# The *Mecyclothorax* beetles (Coleoptera, Carabidae, Moriomorphini) of Haleakala-, Maui: Keystone of a hyperdiverse Hawaiian radiation

**DOI:** 10.3897/zookeys.544.6074

**Published:** 2015-12-11

**Authors:** James K. Liebherr

**Affiliations:** 1 Cornell University Insect Collection, 2144 John H. and Anna B. Comstock Hall, 129 Garden Ave., Cornell University, Ithaca, NY 14853-2601, USA

**Keywords:** Allopatric speciation, biodiversity, biogeography, genitalic evolution, revisionary systematics

## Abstract

The *Mecyclothorax* carabid beetle fauna of Haleakalā volcano, Maui Island, Hawai‘i is taxonomically revised, with 116 species precinctive to Haleakalā recognized, 74 newly described. Species are classified into 14 species groups, with the newly described species arrayed as follows: 1, *Mecyclothorax constrictus* group with *Mecyclothorax perseveratus* **sp. n.**; 2, *Mecyclothorax obscuricornis* group with *Mecyclothorax notobscuricornis* **sp. n.**, *Mecyclothorax mordax* **sp. n.**, *Mecyclothorax mordicus* **sp. n.**, *Mecyclothorax manducus* **sp. n.**, *Mecyclothorax ambulatus* **sp. n.**, *Mecyclothorax montanus* **sp. n.**, *Mecyclothorax waikamoi* **sp. n.**, *Mecyclothorax poouli* **sp. n.**, and *Mecyclothorax ahulili* **sp. n.**; 3, *Mecyclothorax robustus* group with *Mecyclothorax affinis* **sp. n.**, *Mecyclothorax anchisteus* **sp. n.**, *Mecyclothorax consanguineus* **sp. n.**, *Mecyclothorax antaeus* **sp. n.**, *Mecyclothorax cymindulus* **sp. n.**, and *Mecyclothorax haydeni* **sp. n.**; 4, *Mecyclothorax interruptus* group with *Mecyclothorax bradycelloides* **sp. n.**, *Mecyclothorax anthracinus* **sp. n.**, *Mecyclothorax arthuri* **sp. n.**, *Mecyclothorax medeirosi* **sp. n.**, *Mecyclothorax inconscriptus* **sp. n.**, and *Mecyclothorax foveolatus* **sp. n.**; 5, *Mecyclothorax sobrinus* group with *Mecyclothorax foveopunctatus* **sp. n.**; 6, *Mecyclothorax ovipennis* group with *Mecyclothorax subtilis* Britton & Liebherr, **sp. n.**, *Mecyclothorax patulus* **sp. n.**, *Mecyclothorax patagiatus* **sp. n.**, *Mecyclothorax strigosus* **sp. n.**, *Mecyclothorax takumiae* **sp. n.**, *Mecyclothorax parapicalis* **sp. n.**, *Mecyclothorax mauiae* **sp. n.**, *Mecyclothorax subternus* **sp. n.**, *Mecyclothorax flaviventris* **sp. n.**, *Mecyclothorax cordaticollaris* **sp. n.**, and *Mecyclothorax krushelnyckyi* **sp. n.**; 7, *Mecyclothorax argutor* group with *Mecyclothorax ommatoplax* **sp. n.**, *Mecyclothorax semistriatus* **sp. n.**, *Mecyclothorax refulgens* **sp. n.**, *Mecyclothorax argutulus* **sp. n.**, *Mecyclothorax planipennis* **sp. n.**, *Mecyclothorax planatus* **sp. n.**, and *Mecyclothorax argutuloides* **sp. n.**; 8, *Mecyclothorax microps* group with *Mecyclothorax major* **sp. n.**, *Mecyclothorax xestos* **sp. n.**, *Mecyclothorax orbiculus* **sp. n.**, and *Mecyclothorax contractus* **sp. n.**; 9, *Mecyclothorax scaritoides* group with *Mecyclothorax scarites* **sp. n.**, *Mecyclothorax timberlakei* **sp. n.**, *Mecyclothorax crassuloides* **sp. n.**, *Mecyclothorax crassulus* **sp. n.**, *Mecyclothorax gracilicollis* **sp. n.**, and *Mecyclothorax dispar* **sp. n.**; 10, *Mecyclothorax haleakalae* group with *Mecyclothorax reiteratus* **sp. n.**, *Mecyclothorax splendidus* **sp. n.**, *Mecyclothorax bacrionis* **sp. n.**, and *Mecyclothorax simpulum* **sp. n.**; 11, *Mecyclothorax vitreus* group with *Mecyclothorax kipwilli* **sp. n.**, *Mecyclothorax kipahulu* **sp. n.**, *Mecyclothorax kaumakani* **sp. n.**, and *Mecyclothorax kuiki* **sp. n.**; 12, *Mecyclothorax montivagus* group with *Mecyclothorax rex* **sp. n.**; 13, *Mecyclothorax ducalis* group with *Mecyclothorax aquilus* **sp. n.**, *Mecyclothorax invisitatus* **sp. n.**, *Mecyclothorax longidux* **sp. n.**, and *Mecyclothorax brevidux* **sp. n.**; and 14, *Mecyclothorax palustris* group with *Mecyclothorax hephaestoides* **sp. n.**, *Mecyclothorax oculellus* **sp. n.**, *Mecyclothorax bicoloris* **sp. n.**, *Mecyclothorax bicoloratus* **sp. n.**, *Mecyclothorax bilobatus* **sp. n.**, *Mecyclothorax palustroides* **sp. n.**, *Mecyclothorax filipoides* **sp. n.**, *Mecyclothorax nanunctus* **sp. n.**, *Mecyclothorax tauberorum* **sp. n.**, and *Mecyclothorax pau* **sp. n.** *Mecyclothorax integer* Sharp, stat. n. is recognized as a species distinct from *Mecyclothorax interruptus* Sharp. Because type series for species described by Blackburn, Karsch, and Sharp are most often divided among geographically remote collections, lectotypes are designated to stabilize the nomenclature. The radiation includes numerous cryptic sibling species best diagnosed using male genitalia, and photographs are used to represent the male genitalic variability observed among numerous dissected individuals. The large number of new species is based on substantial new collections made from all quarters of the mountain. The dense geographic sampling allows fine-scale discrimination of species boundaries, elucidating the geographic disjunctions that are associated with speciation within this hyperdiverse radiation. Disjunctions between closely related species precinctive to various areas of the mountain are not congruent across the different lineages of the radiation, indicating differential responses by the various lineages to past geological and geographical events. Of the 62 1’ latitude × 1’ longitude grid cells on Haleakalā that are occupied by *Mecyclothorax* beetles, 22 house 10 or more species, and 9 house 20 or more species. This substantial level of sympatry, associated with occupation of diverse microhabitats by these beetles, provides ample information useful for monitoring biodiversity of the natural areas of Haleakalā.

## Introduction

The Hawaiian Islands are home to a remarkable assemblage of carabid beetles, unique in the World for its composition, as well for its inordinate species-level diversity. Like the Hawaiian biota at large (Zimmerman 1970), the native Carabidae are exceedingly disharmonic, being represented by beetles assignable to only three tribes—Bembidiini, Moriomorphini, and Platynini—of the total 110 tribes currently recognized in the family (Bousquet 2012). However each of these tribal taxa are represented by numerous species native to Hawai‘i, all of them geographically restricted to the archipelago. The Platynini are represented by 133 extant and 7 extinct species of the endemic genus *Blackburnia* Sharp, with these species distributed on all of the current high islands; Kaua‘i, O‘ahu, Moloka‘i, Lāna‘i, Maui and Hawai‘i Island (Liebherr and Zimmerman 2000, Liebherr and Porch 2015). The radiation is monophyletic (Liebherr and Zimmerman 1998), with closest relatives in Australia (Liebherr 2005a). The Bembidiini of Hawai‘i include 23 native species of *Bembidion* Latreille (Liebherr 2008a) representing two colonization events, both emanating from New Zealand (Liebherr and Maddison 2013). Several other bembidiine genera also include Hawaiian species—*Tachys* Stephens *sensu lato*, *Lymnastis* Motschulsky, and *Typhlonesiotes* Jeannel (Britton 1948a)—though only one to several species have evolved in each of these colonizing lineages. However it is the Hawaiian representatives of the Moriomorphini that set the standard for diversity. In all, based on this revision, there are 239 native species of *Mecyclothorax* Sharp known from Hawai‘i. Their collective distribution differs from the distributional pattern ascribed to progressive colonization of the Hawaiian Island chain (Gillespie and Roderick 2002), as the greatest diversity, by far, resides on Haleakalā, Maui, where 116 species are known. Conversely, the genus is not represented on Kaua‘i, the oldest subaerial high island. Fitting with that absence, the O‘ahu *Mecyclothorax* fauna is attenuated, with only a few species groups and 20 species occurring on the island (Liebherr 2009a), whereas the present-day fragments of Maui Nui support more species—Moloka‘i with 43 (Liebherr 2007) and West Maui with 27 (Liebherr 2011)—complementing the great diversity of Haleakalā. The *Mecyclothorax* fauna of the geologically youngest island of Hawai‘i (Liebherr 2008b) comprises 30 species, all members of species groups that have diversified on Haleakalā. The Hawaiian radiation is monophyletic based on morphological characters (Britton 1948b, Liebherr 2013), with the most generalized species, *Mecyclothorax montivagus* (Blackburn) residing in open shrubland on the lee slopes of Haleakalā.

**Figure 1.**
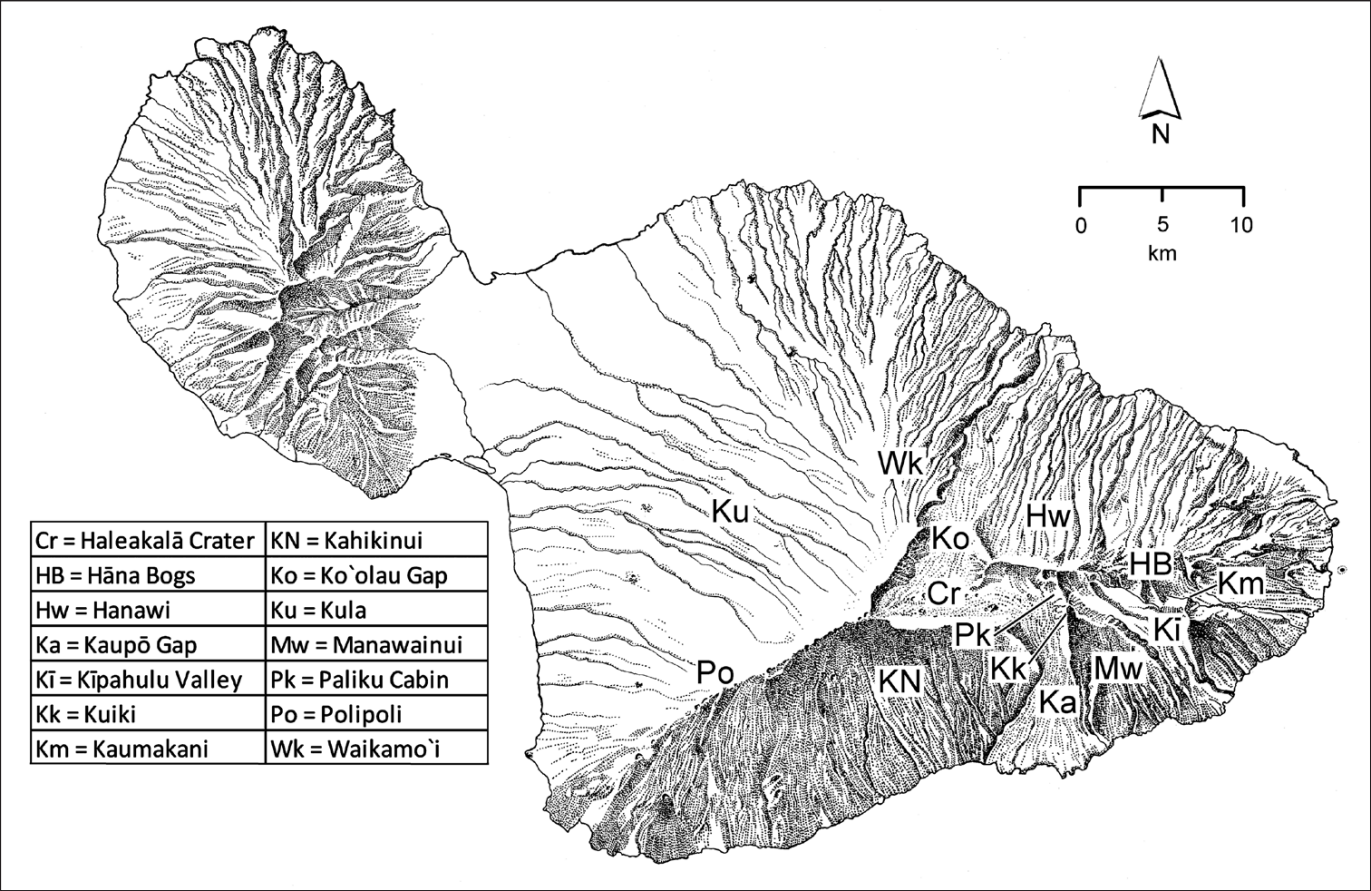
Map of Maui Island, Hawai‘i labeled with regions of Haleakalā volcano mentioned in text.

The *Mecyclothorax* species of Haleakalā are taxonomically revised below. By the numbers, Hawaiian *Mecyclothorax* are predominantly rainforest species, with the highest diversity in the windward forests of Waikamoi, Hanawī, and Kīpahulu Valley (Fig. 1). Thus the 116 known species are concentrated into only a portion of Haleakalā’s 1,440 km^2^ surface area. This biotic concentration is evidenced by extremely high levels of sympatry among the various species, with more than 20 species commonly recorded from a forested area of 1’ latitude × 1’ longitude. This level of diversity has confounded past attempts to identify specimens representing this genus, especially because only about one-third of the Haleakalā species were described, either in the premier *Fauna Hawaiiensis* (Sharp 1903), or the subsequent revision by Britton (1948b). This revision attempts to rectify that problem by providing numerous photographs to document the morphological diversity of this fauna. The amount of biological information inherent in such large radiations is essentially limitless, as it can be viewed in light of phylogenetic relationships and thus become relevant to hypotheses regarding speciation or selection, or be joined with geographical or ecological information to assist conservation management.

## Materials and methods

**Figure 2.**
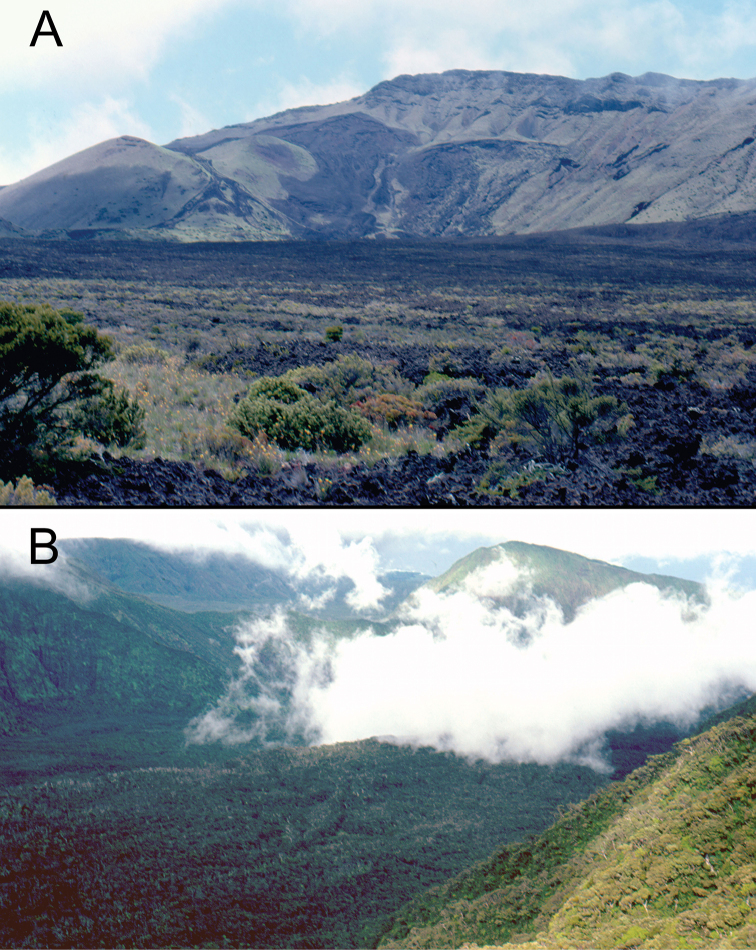
Views of Haleakalā volcano. **A** Haleakalā Crater looking northwest toward Hanakauhi. Photo <br/> taken from Sliding Sands Trail **B** Kīpahulu Valley from Kuiki, with Kaumakani in the right rear distance and Waiho‘i Valley to its left. Ridge within Kīpahulu Valley is Central Pali.

**Haleakalā.** Thomas Blackburn wrote “The eastern end of Maui is, in my opinion, the head-quarters of the insect fauna of the archipelago. It is formed entirely by that gigantic mountain Haleakala ... (Blackburn 1885: 205).” At over 3000 m tall, the mountain supports many habitats that are defined by topographic parameters such as altitude and windward aspect, as well as geological factors such as the recency of volcanic lava flows (Sherrod et al. 2007). From available specimens, both recently and historically collected, we can surmise that *Mecyclothorax* beetles occupied much of the gigantic mountain. There are historical records from as low as 450 m elevation (Fig. 6), and a well-developed fauna in Haleakalā Crater (Fig. 2A) and at the 3000 m summit in areas of *Sophora* shrubland or alpine aeolian desert. Recent records from such marginal carabid habitats are few, and therefore quite valuable, with most beetle collections coming from rainforest habitats on or adjoining the windward, i.e. northeastern and eastern upper slopes of the mountain. Nevertheless, moisture is also available to the leeward south slope either during episodes of southerly, or kona weather, or by uphill convection of moist marine air leading to the development of fog, and condensation of water on plant surfaces (Juvic and Nullet 1995).

In order to place the specimens used as the basis for this revision in an ecological context, an outline of the major habitat formations and biogeographic areas across the windward face is presented. This synopsis necessarily takes a coleopterist’s eye view, but will hopefully allow those who venture into the field the ability to know when they are in the same situations that resulted in the taxonomic material currently in hand. Conversely, any findings made outside the situations presented here will add to what is known about where and how these beetles live. For an overview of the botanical communities in Hawai‘i, with special emphasis on those in Haleakalā, the reader should consult Gagné and Cuddihy (1990). Their classification of botanical formations is followed throughout this revision. The outline below commences with the Waikamoi area, the best collected area as it was the only rainforest accessible to 19^th^ Century collectors such as T. Blackburn and R.C.L. Perkins. This botanically rich and geographically complex area remains accessible and was visited on numerous occasions for this study. The outline then proceeds clockwise through the other identified areas of Haleakalā (Fig. 1).

**Figure 3.**
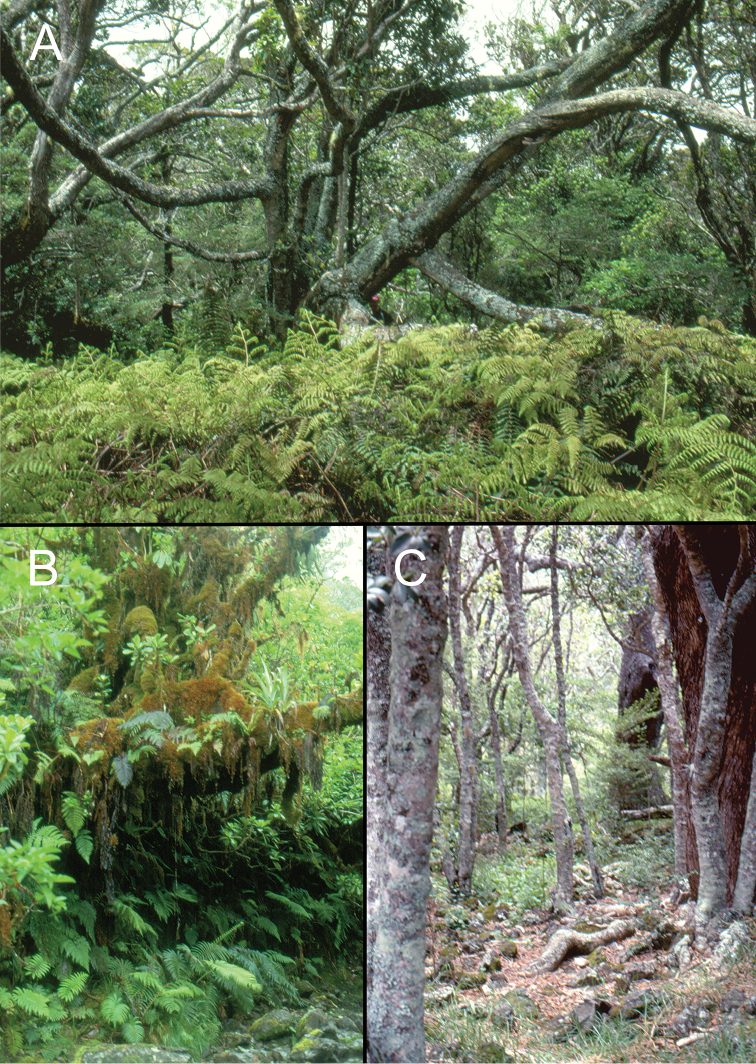
Forests of Haleakalā volcano. **A** *Acacia koa* (koa) tree in mesic montane forest of Waikamoi Nature Conservancy Preserve, Honomanu drainage, 1850 m elevation (photo courtesy D.A. Polhemus) **B** Moss-covered *Metrosideros polymorpha* (‘ōhi‘a) tree next to Kuhiwa Stream, 1600 m elevation **C** Koa Mesic Forest in Kaupō Gap, 1500 m elevation.

*Waikamoi*. Forests of this area are dominated by koa (*Acacia koa*) and ‘ōhi‘a (*Metrosideros polymorpha*), with intact, accessible forest habitats spanning 900–2000 m elevation. Though the stream drainages in this area lie along the western, leeward edge of Haleakalā’s windward face, rainfall is abundant and the gulches may be very deep. The forest is wetter from rainfall at lower elevations, with ‘Ōhi‘a/Hāpu‘u Wet Forest present from 900–1400 m. At higher elevations koa becomes more prevalent, with large trees dominating the forest at 1500 m elevation near the western forest edge (Fig. 3A). The koa forest is well developed near Ukulele Camp (Site) at ~1600 m (5360 ft.) elevation (USGS 1983). This site was carefully collected because it served as R.C.L. Perkins’ base camp (Perkins 1894, 1896a, b). The streams that run through this area within whose gulches *Mecyclothorax* beetles were collected include (west to east): Kahakapao, Opana, Waikamoi, Haipua‘ena, and Honomanu. The *Mecyclothorax* fauna of this area includes several ecologically defined subfaunas. The most generalized component of the fauna is associated with ‘ōhi‘a trees and associated moss growing on trunks, in branch crotches, or on exposed air roots (Fig. 3B). Many of these species, such as *Mecyclothorax consanguineus*, *Mecyclothorax filipoides*, *Mecyclothorax iteratus*, *Mecyclothorax kipwilli*, *Mecyclothorax ovipennis*, *Mecyclothorax perstriatus*, and *Mecyclothorax robustus* may also occur on koa, though the number of collecting occurrences of these species is small. Conversely, there are species in this area that are predominantly associated with koa; e.g. *Mecyclothorax haleakalae*, *Mecyclothorax macrops*, and *Mecyclothorax vitreus*. These beetles occur under bark flaps during daytime, and forage on tree branches during nighttime.

The third component is a terrestrial suite of species that occur predominantly in association with leaf litter on well-drained soil, most often in association with koa forest along the drier western and upper elevational edges of the forest. These species include *Mecyclothorax inaequalis*, *Mecyclothorax longulus*, *Mecyclothorax multipunctatus*, *Mecyclothorax obscuricornis*, *Mecyclothorax sobrinus*, and *Mecyclothorax unctus*. A terrestrial species—e.g. *Mecyclothorax unctus*—may also be found along stream edges, though this habitat is rarely occupied by *Mecyclothorax* beetles. The combined diversity of these different guilds is very large, with an extensive number of species precinctive to this portion of the mountain. In all, 25 species are restricted to forests west of Ko‘olau Gap. Even though Blackburn and Perkins necessarily centered their collecting here, 14 of these endemics are newly described below.

*Ko‘olau Gap/Ke‘anae Valley*. East of the Waikamoi forests and gulches lies this broad Pleistocene erosional feature with secondary volcanic vents (Sherrod et al. 2006). The valley floor is very wet, with standing pools of water surrounded by low-stature ridges bearing open forest vegetation consisting of ‘ōhi‘a and ōlapa (*Cheirodendron* spp.) trees. *Cibotium* tree ferns (hāpu‘u) are also prevalent, often growing on downed ‘ōhi‘a nurse logs. The open understory sampled for *Mecyclothorax* beetles is covered with dense native ‘ākala (*Rubus hawaiensis*). In keeping with the ~1000 year old flows within Ko‘olau Gap (Sherrod et al. 2006), there are no species precinctive to this area, and populations here may represent Waikamoi-centered species, e.g. *Mecyclothorax interruptus* (Fig. 51) or *Mecyclothorax rex* (Fig. 130), widespread windward species such as *Mecyclothorax mauiae* (Fig. 71) or *Mecyclothorax iteratus* (Fig. 106), or western-limital populations of species otherwise known from Hanawī and the Hāna Bogs to the east; e.g. *Mecyclothorax bacrionis* (Fig. 112) and *Mecyclothorax pau* (Fig. 163).

*Hanawī*. As circumscribed in this treatment, Hanawī is geographically broad, including all drainages between Ke‘anae Valley on the west, and Helele‘ike‘oha Stream on the east. The area receives abundant rainfall, with the weather station on Kuhiwa Stream receiving the highest rainfall amounts on the windward face (Stearns and MacDonald 1942). The area is ecologically disparate, with a broad area centered on Kopili‘ula Stream that is devoid of closed forest, the ‘ōhi‘a trees experiencing significant dieback (Holt 1983). *Mecyclothorax* beetles were sampled between 1100–1200 m elevation in an area with extensive, 1.5–2 m tall uluhe fern (*Dicranopteris*) banks covering the ground, and many downed ‘ōhi‘a logs, with scattered emergent koa trees the major standing woody vegetation. This formation best corresponds to the Lowland Wet ‘Ōhi‘a/Uluhe Fern Forest formation of Gagné and Cuddihy (1990: 89), but with the ‘ōhi‘a trees removed by dieback. Further east the country is continuously wooded, with Wet ‘Ōhi‘a/Hāpu‘u (*Metrosideros*/*Cibotium*) Forest at lower elevations, and more dominant ‘ōhi‘a and ōlapa (*Cheirodendron* spp.) at higher elevations. The extensive rainfall and condensed fog and mist provide abundant moisture supporting development of substantial moss mats on ‘ōhi‘a trees (Fig. 3B). Many beetles reside in these mats, with the best means to sample these epiphytic growths being the use of pyrethrin fog. Fewer beetles are associated with ground-level microhabitats due to the extensive runoff over the rocky and muddy surface. The soil surface suffered prior to our visits from the depredations of feral pigs (*Sus scrofa*), with their rooting extensively damaging the forest understory plants. Infrastructure in this area includes two cabins established to support study of the formerly endangered and now extinct Po‘o uli (*Melamprosops phaeosoma* Casey and Jacoby), a distinctive Hawaiian honeycreeper of the avian family Fringillidae. Elevational transects were established to facilitate study of bird population levels in this area (Simon et al. 2002) and these provided the means to move vertically on the mountain while sampling for insects. The Kuhiwa and Helele‘ike‘oha Stream drainages were sampled in this area, the former from its headwaters down to 880 m elevation. Seven *Mecyclothorax* species are known to be precinctive to the Hanawī region as defined here, and another five species have distributions centered here but extending to neighboring areas such as Kīpahulu Valley or the Hāna Bogs.

**Figure 4.**
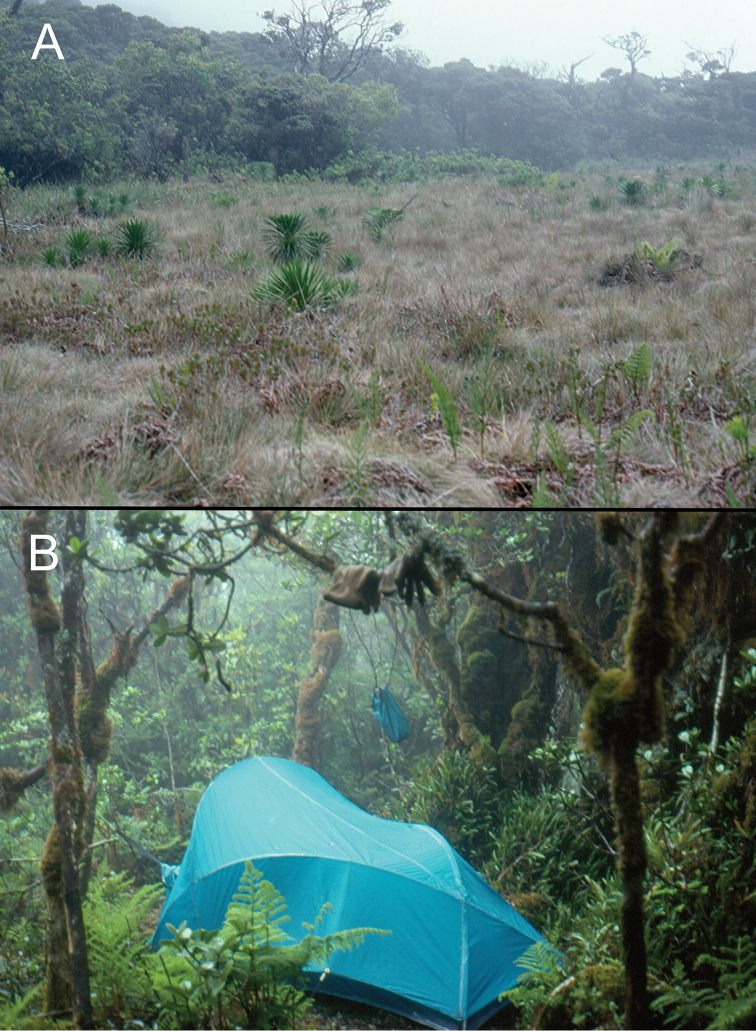
Moist habitats of Haleakalā volcano. **A** New Greensword Bog in the Hāna Bogs area, 1850 m elevation **B** Wet montane forest ESE Kuiki along the western rim of Kīpahulu Valley, 1850 m.

*Hāna Bogs*. This poorly drained tableland lies above and to the east of the eastern Hanawī area, and is bordered to the east by Waiho‘i Valley, to the south by Kīpahulu Valley, and to the southwest by Kalapawili Ridge, the northern summit ridge of Haleakalā Crater. The vegetation is classified as the ‘Ōhi‘a Montane Wet Mixed Community (Gagné and Cuddihy 1990), with open bog surfaces populated with sedges and grasses. These develop hummocks that can support woody vegetation—‘ōhi‘a, ōlapa, and the stunning Lobelias—with the margins of the bogs hemmed in by small stature, 5–7 m tall, ‘ōhi‘a trees (Fig. 4A). Beetles were encountered in epiphytic mosses growing on the emergent woody vegetation. Given the very wet, in some instances flowing bog surfaces, movement of individual brachypterous beetles must occur only during drier periods of the year. Endemism is rather low here for *Mecyclothorax*, with only *Mecyclothorax medeirosi* restricted to this formation. However several species known from Hanawī and Kīpahulu Valley also occur here, elevating *Mecyclothorax* diversity somewhat.

*Kīpahulu Valley*. With its head lying southwest of the Hāna Bogs tableland, Kīpahulu Valley extends broadly southeastwardly to the sea, affording a continuous transect of native forest from Mauka Ridge at 2050 m elevation, to the deterioration of native forest approaching 600 m elevation. The valley floor consists of recent, Hāna volcanic flows (Sherrod et al. 2007) with a flow along the southern half of the valley overlying an older flow that defines the northern half of the valley floor. The Central Pali at their junction can be travelled, allowing access to habitats at different elevations (Fig. 2B). The higher elevations—1900–2000 m elevation—support ‘Ōhi‘a (*Metrosideros*) Montane Wet Forest (Gagné and Cuddihy 1990), a low stature formation that is composed almost exclusively of ‘ōhi‘a, with the 5–8 m tall trees covered abundantly with epiphytic mosses and lichens. At mid-elevations—1200–1500 m elevation—the forests support koa and hāpu‘u (*Cibotium*) tree ferns as well as ‘ōhi‘a, producing a more complex forest mosaic. At the bottom of the transect—600–900 m elevation—the forest is classified as ‘Ōhi‘a Lowland Wet Forest (Gagné and Cuddihy 1990). At and below this elevational level the forest becomes heavily invaded with strawberry guava (*Psidium cattleianum*) and non-native grasses, and native insects are not found. Through collaboration with Drs. A. Medeiros and D. Polhemus, the entire elevational transect was sampled for *Mecyclothorax* beetles. Based on the material in hand, endemism is high, with nine species known only from within Kīpahulu Valley, and seven more species nearly precinctive to the valley, as they also include populations in adjacent areas such as Hanawī, the eastern reaches of Haleakalā Crater, and Kaupō Gap. Species are subdivided by elevation, with high elevation specialists including *Mecyclothorax anchisteus* (Fig. 32), *Mecyclothorax refulgens* (Fig. 85), and *Mecyclothorax kipahulu* (Fig. 121). Conversely *Mecyclothorax dispar* is known only from 1200 m elevation (Fig. 101), and *Mecyclothorax aquilus* only from 900 m (Fig. 135). Other Kīpahulu precinctives—e.g. *Mecyclothorax manducus* (Fig. 21) and *Mecyclothorax cymindulus* (Fig. 40)—are known from a range of elevations in the valley, but have yet to be found in adjacent areas.

**Figure 5.**
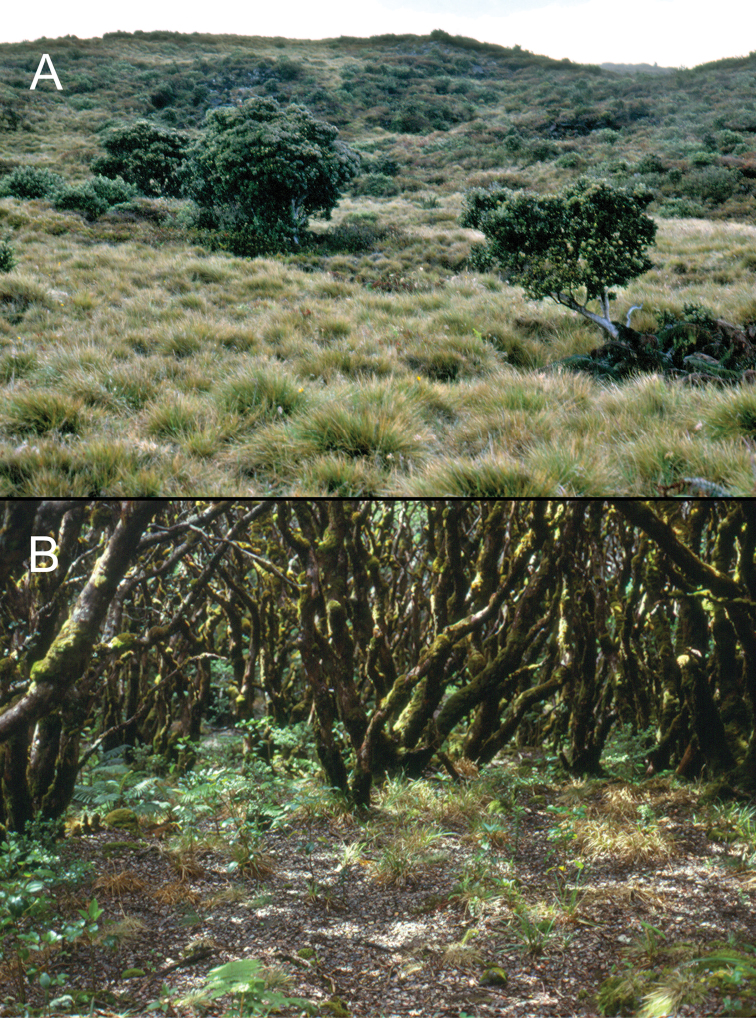
Vegetation in vicinity of Kuiki. **A** Open ‘Ōhi‘a Savannah above timber line near Kuiki summit, 2100 m elevation **B** Elfin ‘Ōhi‘a Forest just below timber line showing well-drained leaf-litter humus layer in “rooms" within the low forest canopy.

*Manawainui Planeze*. Bounded on the north by Kīpahulu Valley and on the west by Kaupō Gap, this pie-slice shaped planeze of Kula Volcanics—0.228 Myr old (Sherrod et al. 2003)—is exceedingly discrete. At 2100 m elevation near its northwest summit of Kuiki, the habitat is open ‘ōhi‘a woodland, with individual trees isolated by grasses (Fig. 5A). Copses of small ‘ōhi‘a trees also occur, with the small-stemmed plants bearing epiphytic mosses, and the ground densely covered with slowly decaying myrtaceous leaf litter (Fig. 5B). *Mecyclothorax* beetles have been collected within these shaded situations, including three species precinctive to Manawainui Planeze; *Mecyclothorax mordax* (Fig. 19), *Mecyclothorax antaeus* (Fig. 36), and *Mecyclothorax gracilicollis* (Fig. 101). A fourth geographically restricted species also occurs here; *Mecyclothorax kuiki* also found in the Hāna Bogs (Fig. 121). Moving downward in elevation brings one into the ‘Ōhi‘a (*Metrosideros*) Montane Wet Forest at 1800–1900 m elevation (Fig. 4B). Here 5–8 m tall ‘ōhi‘a trees dominate with the occasional ōlapa (*Cheirodendron*), and understory shrubs including pilo (*Coprosma*) and pūkiawe (*Leptecophylla*) (Gagné and Cuddihy 1990). *Mecyclothorax strigosus* has only been collected at this elevation on the planeze (Fig. 64). Further downhill—1600–1700 m elevation—this formation is complemented by hāpu‘u (*Cibotium*) tree ferns, with the ‘ōhi‘a trees of larger stature. *Mecyclothorax ahulili* (Fig. 25) and *Mecyclothorax kaumakani* (Fig. 121) have been collected in this elevational zone near Puu Ahulili.

*Haleakalā Crater and Kaupō Gap*. These two areas are floored by very young volcanic deposits, ranging from only about 1000 years within the crater, and about 8000 years along the eastern margin of Kaupō Gap (Sherrod et al. 2006), strongly suggesting that any species currently known only from these areas evolved either somewhere else, or within the bounds of these features, with their species’ members then dispersing to colonize the newer ground upon which they were collected during this study. The western crater is drier, and therefore covered with a dry shrubland formation (Fig. 2A). Few specimens have been collected in this area, though those that have been—e.g. *Mecyclothorax parapicalis* endemic to the western crater—suggest that the area should be sampled more thoroughly. The eastern crater margin at Paliku lies at about 2000 m elevation directly under the headwall of Kīpahulu Valley, with veils of trade-wind moisture spilling onto the area from the eastwardly looming Kīpahulu summit ridge. This moisture source supports a mesic savannah biota, with native *Rubus* (‘ākala) abundant, and copses of ōlapa (*Cheirodendron*) occurring amongst the shrubland plants. This situation is remarkably beneficent to *Mecyclothorax* beetles, with two species known only from this site—*Mecyclothorax takumiae* (Fig. 67) and *Mecyclothorax major* (Fig. 94), and a third found here and also further south in Kaupō Gap; *Mecyclothorax inconscriptus* (Fig. 51). Other species collected at Paliku also live in much wetter locales; e.g. *Mecyclothorax arthuri* also known from Kuiki on the Manawainui Planeze and from the head of Kīpahulu Valley (Fig. 51), and *Mecyclothorax planipennis* from those sites plus Waiho‘i Valley (Fig. 86). Progressing south from Paliku into Kaupō Gap, one enters Koa Mesic Forest near 1500 m elevation. *Mecyclothorax cordaticollaris* (Fig. 77) is known only from this geographically restricted koa forest, and *Mecyclothorax simpulum* is found here and also at lower elevations in Kīpahulu Valley (Fig. 112).

*Kahikinui*.—Everything we know about the *Mecyclothorax* fauna of the immense south face of Haleakalā, much included in the Kahikinui district, comes from the efforts of Drs. Paul Krushelnycky and Robert Peck. Known *Mecyclothorax* diversity in the Koa/‘Ōhi‘a Montane Mesic Forest that occurs on this slope amounts to four species—*Mecyclothorax giffardi* (Fig. 56), *Mecyclothorax krushelnyckyi* (Fig. 79), *Mecyclothorax cordithorax* (Fig. 89), and *Mecyclothorax iteratus* (Fig. 106)—with only *Mecyclothorax krushelnyckyi* restricted to Kahikinui. The koa/‘ōhi‘a forest has been seriously degraded by cattle (*Bos taurus*), goats (*Capra hircus*), and pigs (*Sus scrofa*), though mature koa and ‘ōhi‘a trees remain on the slope. The leeward site offers a possible locale for translocation of individuals of the endangered Maui Parrotbill (*Pseudonestor xanthophrys*: Family Fringillidae) (Mounce et al. 2015). Ongoing efforts to reestablish Koa/‘Ōhi‘a Forest in Kahikinui—specifically the Nakula Natural Area Reserve of the State of Hawai‘i—is intended to ensure the arthropod resource base required by the endangered birds (Peck et al. 2015). Such rehabilitation efforts may offer enhanced opportunities for all of the native fauna to develop populations on this face of Haleakalā.

*Polipoli Springs*. Situated on the southwest rift of Haleakalā, Polipoli Springs is an oasis of mesic forest vegetation at the boundary of the drier Kahikinui and Kula faces of the mountain. The original native Koa/‘Ōhi‘a Mesic Forest was degraded through grazing, with reforestation during the 1930s taking the form of numerous exotic trees planted in large plots as an experimental forest. Exotic tree species include Monterey Pine (*Pinus radiata*), Tropical Ash (*Fraxinus hydei*), and Coast Redwood (*Sequoia sempervirens*). The understory is open and dominated by *Dryopteris wallachiana* fern (laukahi). The exotic pine grows very well in the cloud zone at this elevation, with the trees unstable due to the ashy soil, resulting in extensive lodging, leading to large light gaps covered by downed logs. Even given the alien landscape, native *Mecyclothorax* beetles abound in the leaf litter, under stones near the spring sources, and under loose bark of downed trees (Liebherr 2005b). The species present here often represent southwest outpost populations of species also found in the dry habitats of the northern Kula face to the wetter forest habitats of the mountain’s windward side; e.g. *Mecyclothorax irregularis* (Fig. 48), *Mecyclothorax ovipennis* (Fig. 66), *Mecyclothorax laetus* (Fig. 74), and *Mecyclothorax cordithorax* (Fig. 89). More significantly, the beetles living here are precinctive to Polipoli, their ancestors presumably having survived the ecological holocaust of deforestation during the early 20^th^ Century. These alien forest endemics are closely related to windward forest species found in Waikamoi; e.g. *Mecyclothorax aeneipennis* (Fig. 32), *Mecyclothorax consobrinus* (Fig. 59). A third biogeographic pattern is represented here by *Mecyclothorax giffardi*, a species also found on the drier leeward south face of Haleakalā (Fig. 56). From these patterns it can be concluded that the Polipoli area has continuously supported *Mecyclothorax* populations long enough for speciation to have occurred, and that the area still supports species that persist in the native Koa/‘Ōhi‘a Mesic Forest of Kahikinui.

*Kula*. The dry leeward face of Haleakalā that is accessible for entomological study consists of Montane Dry Shrubland, an open formation from 900–2700 m elevation dominated by small stature ‘ōhi‘a (Gagné and Cuddihy 1990), that grades into open māmane (*Sophora*) shrubland with *Deschampsia* bunchgrass in the higher elevations. *Mecyclothorax* beetles have occurred throughout this zone, with historical records of *Mecyclothorax montivagus* being from rather low elevations (Fig. 132). The higher māmane forest is dominated by *Mecyclothorax cordithorax* (Fig. 89), *Mecyclothorax micans* (Fig. 130), and *Mecyclothorax montivagus* (Fig. 132). Other montane species of the *Mecyclothorax ovipennis* group also occur in this area upwards to the bare, aeolian summit of Pu‘u ‘Ula‘ula; e.g. *Mecyclothorax subconstrictus* and *Mecyclothorax nubicola* (Fig. 79), and *Mecyclothorax pusillus* and *Mecyclothorax rusticus* (Fig. 80). Three of these four—*Mecyclothorax subconstrictus*, *Mecyclothorax pusillus*, and *Mecyclothorax rusticus*—were collected in large numbers only in the weeks after melt of an unusual snowcap during April (Perkins 1894). If populations of these species persist, their individuals may live cryptically within the soil for much of the year, or perhaps spend time in subterranean voids such as burrows of the Hawaiian Dark-rumped Petrel/‘Ua‘u (*Pterodroma sandwichensis*: Family Procellariidae). The upper Kula Face has been invaded by several alien species; Argentine Ant, *Linepithema humile* (Mayr), and a European carabid beetle imported via Oregon, *Trechus obtusus* Erichson (Liebherr and Takumi 2002, Liebherr and Krushelnycky 2007). Both the ant and alien carabid adversely impact native *Mecyclothorax* populations, suggesting another reason for the relative lack of recent collections of the high-altitude *Mecyclothorax ovipennis* group species.

**Figure 6.**
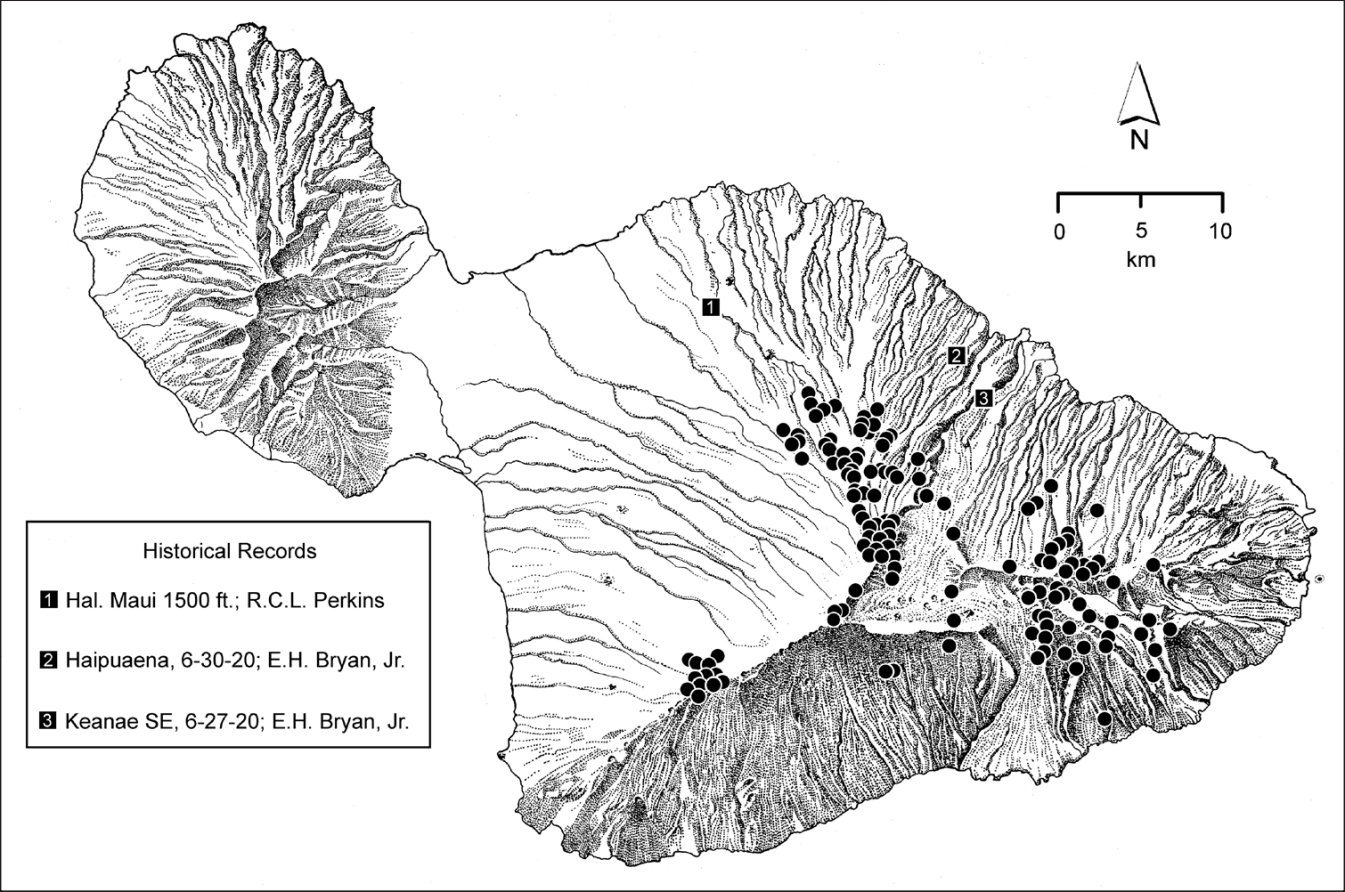
Distribution of localities on Haleakalā volcano from which *Mecyclothorax* beetles have been collected. Numbered localities isolated to the north of the main mass of localities were sites of historical collecting events.

**Taxonomic material.** This revision is based on the study of 7,623 specimens of *Mecyclothorax* collected on Haleakalā (Fig. 6) currently held in 15 institutions (Table 1). Historical material predominantly includes specimens collected by the Rev. Thomas Blackburn (summarized in Blackburn and Sharp 1885) and R. C. L. Perkins (Manning 1986). The bulk of Blackburn’s material was deposited in The Natural History Museum, London (BMNH), although some personally retained syntypes were subsequently deposited in the South Australian Museum, Adelaide. Perkins’ material was collected on behalf of the *Fauna Hawaiiensis* (Sharp 1903), with the collecting series divided between The Natural History Museum, London (BMNH) and the Bernice P. Bishop Museum, Honolulu (BPBM). A third smaller lot of material was collected by Otto Finsch, described by Karsch (1881) and deposited in the Museum für Naturkunde, Berlin (MNHU). Finsch’s specimens remained unlabeled except for lot number until taken on loan by the author. Subsequent to publication of the *Fauna Hawaiiensis*, Blackburn, Finsch, and Perkins material was exchanged with other major institutions in Canberra, Paris, and Milan. Because of this division of syntype series, and some genuine confusion of species in the divided material—some of Blackburn’s syntypes are misidentified—lectotypes have been designated for all species for which a holotype was not previously designated. Most lectotypes were labeled during a brief visit to The Natural History Museum. That visit’s brevity necessitated that the many paralectotypes were not so labeled. For material described in this revision, holotypes were chosen, when possible, from the historically earliest collections of the species. Where cryptic species are newly described here, necessitating use of male genitalia for firm identification, a male specimen, either dissected or with partially everted genitalia, has been chosen as holotype. Holotype data are presented in the text with verbatim transcriptions of the labels, including character spacing and a close approximation of font. Individual lines on labels are separated by slashes ( / ), separate labels by double slashes ( // ). Lectotype label transcription follows that protocol with the exception that label lines for BMNH lectotypes are not denoted. Paratype data are summarized from a specimen database, with redundant fields removed from adjacent records so as to minimize required text space. Holotypes are deposited in the institutions associated with the types’ collectors, except for specimens collected by R. Takumi Kaholoa‘a, Haleakala National Park, which are deposited at the Bishop Museum, Honolulu, where they are held in a secure type collection. Recently collected material forms the bulk of the taxonomic material, with most field specimens generated during expeditions in 1991, 1993, 1998, 1999, 2001, 2003, and 2005. Specimens from 1991 and 1993 were collected in ethyl acetate killing jars, and then held under ethyl acetate atmosphere in scintillation vials holding crumpled Kimwipes® tissues. Prior to preparation the ethyl acetate was allowed to evaporate, and specimens were placed in near boiling water as they were prepared as point-mounted specimens. Specimens from later years were either killed in ethyl acetate, held for a day in the kill jar, and then transferred to 70% ethanol (Liebherr), or collected directly into ethanol (Ewing, Polhemus). These specimens were point-mounted directly from ethanol.

**Table 1.** Specimen repositories with numbers of specimens, and the collectors whose deposited material would be eligible for lectotypification in this revision. Nearly all lectotypes are designated using BMNH and MNHU material. For names of institutions and associated curators, see Acknowledgements.

Institutional Code	No. Specimens	Historical Collectors
ANIC	12	Perkins
BMNH	723	Blackburn, Perkins
BPBM	1,214	Perkins
CAS	392	
CUIC	3,084	
HALE	408	
HDAC	8	
HNHM	4	
MNHN	29	Blackburn, Finsch, Perkins
MNHU	12	Finsch
MSNM	11	Perkins
NMNH	1,502	
SAMA	9	Blackburn
UCRC	59	
UHIM	147	

**Laboratory methods.** Specimen preparation and dissection protocols are the same as those used for prior Hawaiian *Mecyclothorax* revisions (Liebherr 2007, 2008b, 2009a, 2009b, 2011). Briefly, both male and female specimens were relaxed in nearly boiling distilled water held in shell vials placed in a double boiler, the water containing a drop of Kodak Photo-Flo® detergent. For males, the aedeagal median lobe, associated parameres and 9th tergite were disassociated from the abdomen using minute nadeln mounted on wooden dowels. The genitalic apparatus was removed, cleared overnight in cold 10% KOH, deacidified in 10% acetic acid, and then placed in glycerine. If the male internal sac was to be everted using modified minuten nadeln, eversion was done while the dissection was soaking in KOH or acetic acid, as dehydration in glycerine made the sac more brittle and impossible to evert. Female dissections involved removal of the entire abdomen and clearing it overnight in cold 10% KOH. The female reproductive tract was removed from the abdomen by first tearing off the membranous tergites, then gently tearing the 8th laterotergites from the sclerotized abdominal ventrites. The reproductive tract assembly was deacidified briefly in dilute 10% acetic acid, then cleared and stained for approximately 10 minutes in a mixture of Kodak® Chlorazol Black stain suspended in methyl cellosolve. The dissection was placed in glycerine, and the various tracheae and defensive gland assemblies and associated sclerites removed from the gonocoxae and bursa copulatrix complex. This latter was mounted in glycerine on a microscope slide.

All male and female genitalic dissections were photographed using a Microptics (now Visionary Digital®) photographic apparatus employing a Nikon D1 camera, the K2 lens system, and a three-wand photographic strobe fiber-optic light source. All male aedeagal preparations were photographed at the same scale from a right-side view, augmented when appropriate with a ventral view. Aedeagal preparations with internal sac everted were photographed from the right side. In all, 562 male dissections representing 99 species, and 92 female dissections representing 85 species were photographed. For all newly described species at least one male specimen from each collecting series was dissected and photographed, with the photographs used to assist specimen assignment to species. Where necessary, multiple specimens per series were photographed, until all specimens could be assigned to species with confidence using both external and male-genitalic characters. Female dissections were photographed in ventral view however the 3-dimensional complexity of the gonocoxites and associated laterotergites required interpretive line drawings for those structures. To compose the line drawings, the photographed dissections were used to establish the outlines of major features of the gonocoxites, with the finer setational and sensillar features placed on the drawing by hand during examination of the dissection under phase contrast compound microscopy at magnifications of 100–400×. A calibrated ocular grid served to establish scaled dimensions for all photographs and drawings.

**Figure 7.**
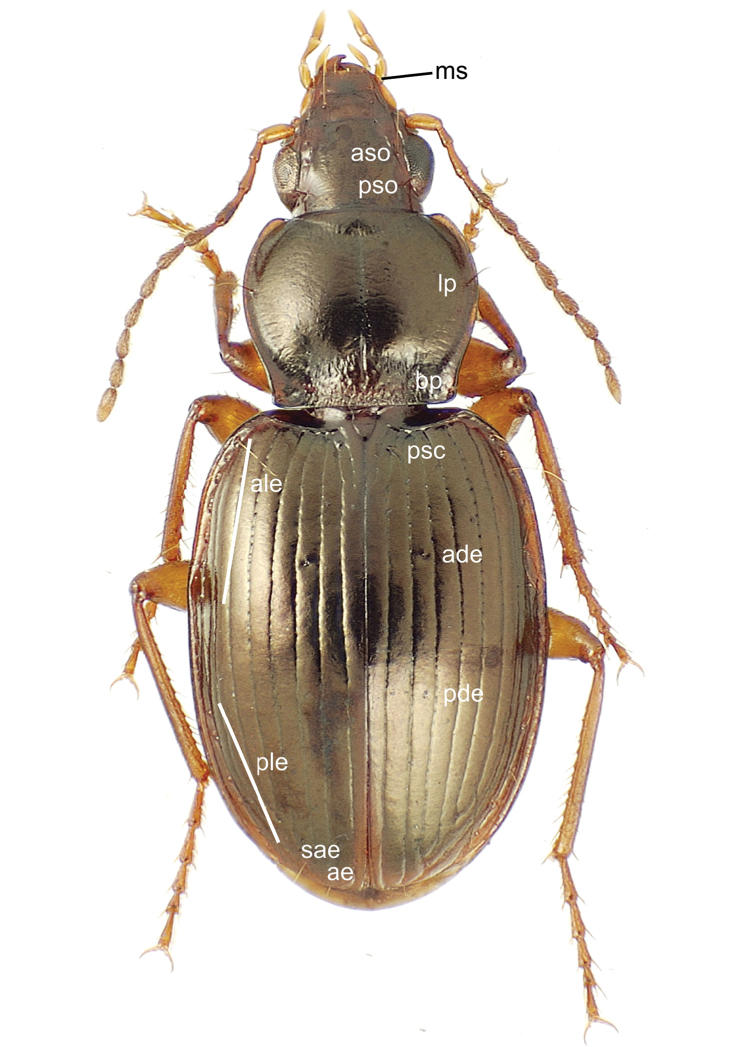
*Mecyclothorax aeneipennis*, dorsal habitus view. Setal positions on the body from anterior to posterior: ms, mandibular scrobe seta; aso, anterior supraorbital seta; pso, posterior supraorbital seta; lp, lateral pronotal seta; bp; basal pronotal seta; psc, parascutellar seta; ale, anterior series of lateral elytral seta; ade, anterior dorsal elytral seta; pde, posterior dorsal elytral seta; ple, posterior series of lateral elytral setae; sae, subapical elytral seta; ae, apical elytral seta.

**Descriptive conventions and characters.** Species descriptions were generated in close consultation with a character matrix developed during extensive examination of specimens and scoring of characters. An initial list of characters, based on experience with the Society Islands *Mecyclothorax* fauna (Liebherr 2012a, 2012b, 2013), was augmented with additional characters as those were discovered among the Hawaiian species. All descriptions include a diagnosis that summarizes the salient characters that may be used to distinguish the species in question from all others. In some instances these characters are the same as those used in the dichotomous identification keys, though other characters also included may diagnose the particular species from those not sequentially adjacent in the key. All diagnoses also include two standard metrics: 1, standardized body length; and 2, the setal formula. Standardized body length is defined as the sum of three linear measurements: 1, the distance from the labral anterior margin to the cervical ridge, a transverse carina posterad the vertex; 2, the medial length of the pronotum; and 3, the elytral length defined as the distance from the base of the scutellum to the elytral apex, measured parallel to the suture. The setal formula was developed and used by Perrault (1984, 1986, 1988, 1989) as a shorthand to diagnose species and species groups for the Society Islands *Mecyclothorax* fauna. The setal formula consists of four numbers, abcd, defined as: a = the number of supraorbital setae each side of the head; b = the number of setae along the lateral margin of the pronotum; c = the number of dorsal elytral setae associated with elytral interval 3 on each elytron; d = the number of setae at the apex of each elytron. There may be either two (Fig. 7) or one supraorbital setae each side; if one it is the posterior seta that is present in Hawaiian species. There may be 0, one, or two lateral pronotal setae; if one it is always the lateral seta, as the basal seta is evolutionarily lost prior to loss of the lateral seta. Most species are characterized by two dorsal elytral setae each side, at approximated 0.3× and 0.6× the elytral length. The posterior seta is absent in some species, and both setae are absent in some others. The setae of the elytral apex include one—termed the apical seta—at the apex of elytral stria 2 just basad the longest point of the elytron, and a 2^nd^ seta—the subapical seta—present in the 7^th^ elytral stria mesad the subapical sinuation (Fig. 7). In species with well-developed elytral striae and cuticular microsculpture, both setae are generally present. In those species with very convex elytra, reduced elytral striation and smooth elytral cuticle, both setae are generally absent. The alternate states of apical seta absent/subapical present and apical seta present/subapical absent also occur. When the number of setae present varies within a species, with several state observed in more than ¼ of the individuals, the value is expressed as x_1_–x_2_. When one configuration is rare—observed in less than 10% of individuals, or only unilaterally—the rare value parenthetically follows the commonly observed value.

Full descriptions of all new species include sections describing the head, pronotum, prosternum, pterothoracic ventrites, abdomen, legs, microsculpture, coloration, male genitalia, and female reproductive tract. Several descriptive ratios are used. For the head these include the ocular ratio and ocular lobe ratio. The ocular ratio is defined as the maximal head width (MHW) across the convex surfaces of the compound eyes, divided by the minimal distance between the eyes across the frons (mFW). The ocular lobe ratio is measured as the length of the eye from anterior to posterior margin measured from dorsal aspect, divided by the distance from anterior eye margin to the posterior margin of the ocular lobe where its projected margin meets the gena. The first ratio provides a measure of eye convexity, whereas the ocular lobe ratio is related to eye diameter. Three descriptive ratios are used to help describe pronotal shape; 1, MPW/BPW, or maximum pronotal width divided by basal pronotal width; 2, MPW/PL, maximum pronotal width divided by pronotal length measured along the midline; and 3, APW/BPW, pronotal width across the front angles divided by basal pronotal width. Basal pronotal width is measured as the distance between the hind angles, whether or not the pronotal lateral margins converge anterad those angles. The degree of humeral development of the elytra varies greatly among these species, as all Hawaiian *Mecyclothorax* are brachypterous, and evolution has proceeded without the functional constraint necessitating maintenance of a fully functional flight apparatus. Thus elytra may be parallel sided and appearing much like those of mainland species that actively fly during their lifetime, or the elytra may be ovoid or ellipsoid with narrowed humeri. This disparity is measured by MEW/HuW, i.e. maximal elytral width divided by humeral width. The latter variable is measured as the transverse distance between the most anterior position of the basal elytral groove, generally where it meets the lateral marginal depression of the elytron. Occasionally two other ratios are used—MEW/MPW, or MEW/MHW—that is the maximal elytral width divided by the maximal pronotal width, or by the maximal head width across the convex surfaces of the compound eyes. These ratios may differentiate species when the degree of elytral “inflation” varies relative to the breadth of the forebody. Elytral setation and striation provide numerous useful characters. The parascutellar seta, present in the base of the sutural stria laterad the parascutellar striole (Fig. 7) may be present or absent, with these states invariant within species. The elytra bear a series of lateral setae associated with the 8^th^ stria. These are arranged in an anterior series that commences just posterad the humeral angle, and a posterior series that lies along the margin of the elytral apical half anterad the subapical sinuation (Fig. 7). In some instances, a single isolated seta may lie between the anterior and posterior series. When available for viewing in dissected individuals, the configuration of the metathoracic flight wings is described. Venation of these variously reduced wing vestigia follows homologies proposed by Kukalová-Peck and Lawrence (1993).

Cuticular microsculpture is largely constant among specimens of the same species, though male specimens may exhibit less well-developed sculpticells compared to females, and they may have sculpticells that are slightly more transversely stretched than in females. The shapes of sculpticells and their aggregate patterns are described using the terminology of Lindroth (1974). When individual sculpticells are described, their length is the dimension along the head-abdomen body axis, and their breadth is the dimension perpendicular to that axis. As the pattern of microsculpture differs greatly on different portions of the major visible sclerites—head capsule, pronotum, elytra, various ventrites—diagnostic presentation of microsculpture specifies the exact position on the body at which it should be assessed.

**Figure 8.**
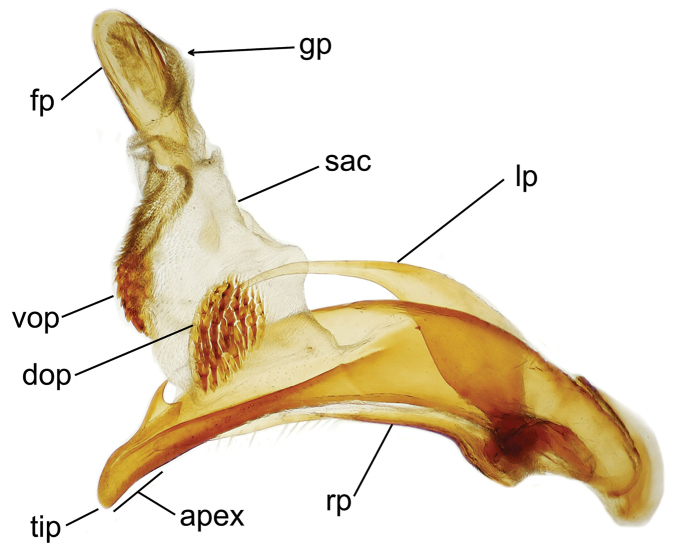
Male aedeagus, right view, *Mecyclothorax aeneipennis*: apex, apical portion of median lobe distad the ostial opening through which the internal sac everts; dop, dorsal ostial microtrichial patch of internal sac; fp, ventrally concave flagellar plate of internal sac; gp, gonopore positioned on membranous dorsal surface overlying flagellar plate; lp, left paramere; rp, right paramere; sac, internal sac; tip, most distal portion of apex; vop, ventral ostial microtrichial patch.

The male aedeagal median lobe and internal sac offer substantial characters for species identification (Table 2). A specimen may be sexed as male by two means. Firstly, all males have the basal three protarsomeres bearing squamose adhesive setae medially on the ventral surface in addition to the trichiform subapical and apical setae laterally near the tarsomere apex. Moreover, in almost all Hawaiian *Mecyclothorax* species, the males have a single seta each side on the apical margin of the apical abdominal ventrite, whereas the females have two setae each side along the apical margin. In two exceptions, males have two setae each side—*Mecyclothorax planipennis* and *Mecyclothorax planatus* (Fig. 87A–B)—but in these species the females have four setae each side. In these rare exceptions to the setal count sex-determination rule, ventral setation of the protarsomere can be used to sex the specimen. When viewing the male aedeagal median lobe, diagnostic differences are often present in the configuration of the apex (Fig. 8), which may vary among the species in length, breadth, and curvature. The depth of the lobe—i.e. dorsoventral breadth—may also vary relative to the length of the aedeagus, with the lobe narrow (Fig. 45M, O) to very broad (Fig. 69). The internal sac bears a flagellar plate (terminology of Maddison 1993) that comprises ventrally a concave sclerotized, cuplike plate, and dorsally an overlying, lightly sclerotized membranous surface that bears the gonopore at its center (Fig. 8). Large spicules may be present on the sac surface, their aggregated structures called ostial microtrichial patches (Maddison 1993). When the patch lies along the midline of the sac between the lobe and the flagellar plate, it is termed the ventral ostial microtrichial patch. A patch may lie along the right side of the sac base, and extend toward the dorsal surface of the sac basad the gonopore. This is termed the dorsal ostial microtrichial patch. Usually the sac is a tubular structure (Fig. 8), although in some species it is divided, with a lobe that bears the flagellar plate—the apical lobe (Figs 120G, 150E–F, 161I)—complemented by a basal lobe situated dorsally closer to the dorsal surface of the median lobe.

**Table 2.** Abbreviations used in male genitalic and female reproductive tract species plates.

Abbreviation	Structure
**Males**	
al	apical lobe of internal sac
bl	basal lobe of internal sac
dop	dorsal ostial microtrichial patch
fp	flagellar plate
gp	gonopore
ms	macrospicules of internal sac
pal	“pineapple” lobe of internal sac
vl	ventral lobe of internal sac
vop	ventral ostial microtrichial patch
**Females**	
abc	apical lobe of bursa copulatrix
bc	bursa copulatrix
bsc	bursal sclerite
co	common oviduct
dgr	defensive gland reservoir
hg	hindgut
lo	lateral oviduct
sg	spermathecal gland
sgd	spermathecal gland duct
sp	spermatheca
v	vagina

**Figure 9.**
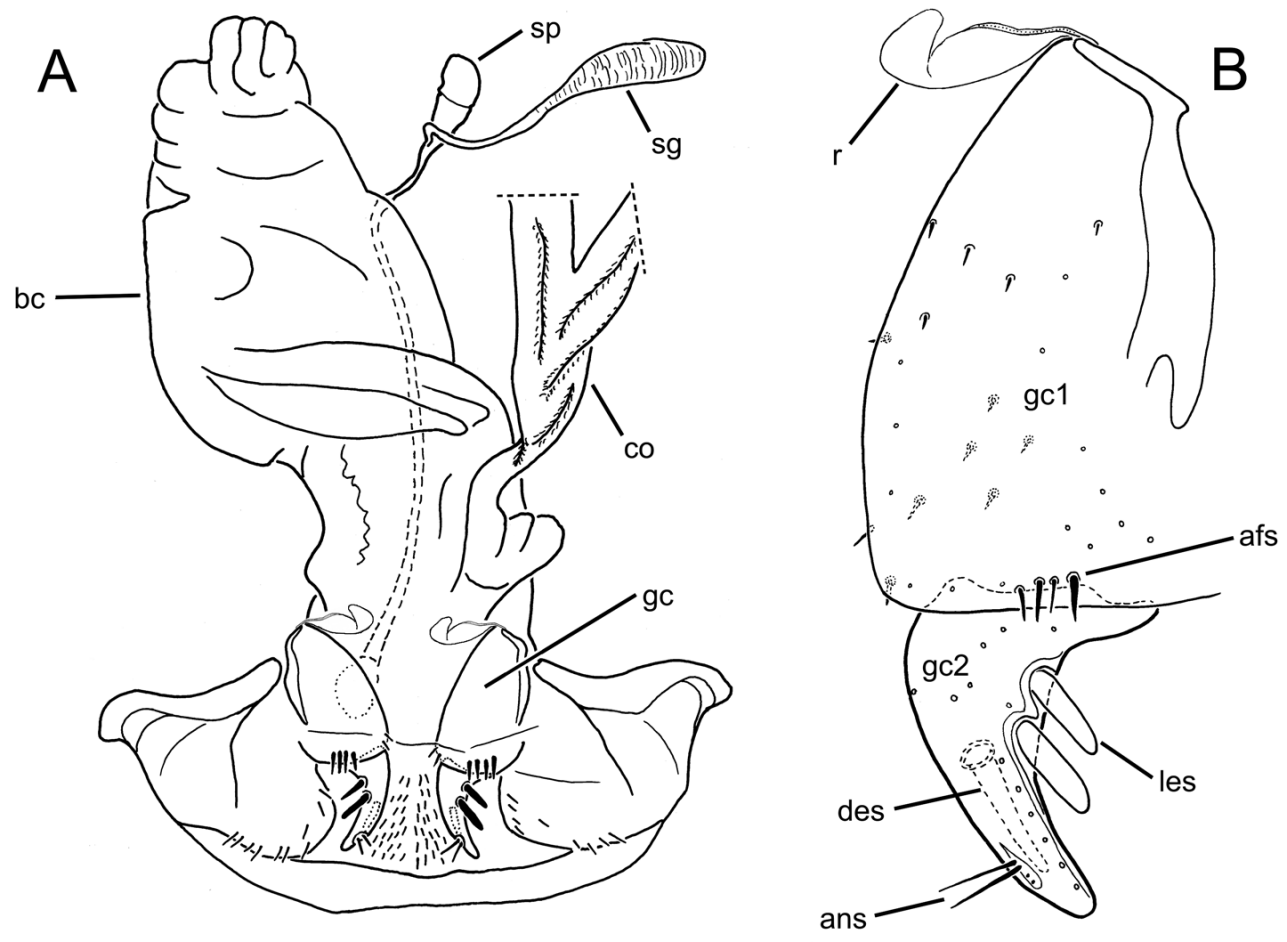
Female reproductive tract structures, *Mecyclothorax aeneipennis*. **A** Female internal genitalia with associated gonocoxae, ventral view: bc, bursa copulatrix; co, common oviduct; gc, gonocoxa; sg, spermathecal gland; sp, spermatheca **B** Left gonocoxa, ventral view: afs, apical fringe setae; ans, apical nematiform setae; des, dorsal ensiform seta of apical gonocoxite; gc1, basal gonocoxite 1 of gonocoxa; gc2, apical gonocoxite 2 of gonocoxa; les, lateral ensiform setae of gonocoxite 2; r, ramus, a membranous or sclerotized lobe associated medially with base of gonocoxa (in this species, apical margin of ramus is narrowly sclerotized).

The female reproductive tract and associated gonocoxal ovipositors are interpreted (Table 2) following the system of Liebherr and Will (1998). The bursa copulatrix is a membranous sac of varying thickness and sclerotization, with the common oviduct entering on the ventral surface, and the spermathecal duct entering medially on the dorsal surface (Fig. 9A). The spermatheca is a fusiform structure of varying configuration, though annular rings are present to varying degrees. The spermatheca has an associated gland whose duct enters at the base of the spermathecal reservoir. The gland is exceedingly membranous and flaccid, with pore ductules visible over its surface. Gland size may vary among individuals of the same species. The underlying cause for this variation remains unstudied. The bursa copulatrix may be sclerotized on its ventral surface distad the juncture with the common oviduct (Fig. 54C). The gonocoxa is bipartite, with a lightly sclerotized basal gonocoxite 1, and a more distinctly sclerotized apical gonocoxite 2 (Fig. 9B). Medial to the base of the basal gonocoxite lies the ramus, a membranous fold of the ventral bursal wall mesad the gonocoxa. The paired rami may be narrowly sclerotized along their anterior margin (Fig. 9B). The basal gonocoxite usually bears a series of larger setae along the apical margin; apical fringe setae (Fig. 9B). These may vary from 1–6 each side, or there may be no setae at this position. The numbers of setae often vary bilaterally by one or two within the same specimen, reducing their utility for diagnosis. There are variable numbers of smaller setae along the medial surface of the basal gonocoxite, and a larger seta may be present at the apicomedial angle of the gonocoxite; the apicomedial seta. The more heavily sclerotized apical gonocoxite bears 1–2 lateral ensiform setae (rarely a third smaller seta), one dorsal ensiform seta, and two apical nematiform setae (Fig. 9B; terminology of Ball and Shpeley 1983). There are campaniform sensilla distributed over the surface of the apical gonocoxite. The presence and position of a series of these sensilla along the lateral margin distad the apical ensiform setae aids in determining how much of the gonocoxal apex has been worn off during ovipositional activity, that wearing down of the coxite especially prevalent in females of the species living in dry shrubland habitats (e.g. *Mecyclothorax subconstrictus*, Fig. 74D).

For previously described species, an initial diagnosis is followed by an identification section that provides additional characters that aid the determination. These include the descriptive ratios defined above, plus any other characters that might assist in the identification.

Hawaiian words, and formal place and animal names have been used as species epithets. Based on The Code (ICZN 1999), all glottal stops, macrons, or other accents must be excluded from words when they are used as formal scientific names. Similarly, in the current presentation of type data, such accents are excluded except when part of a collector’s name. Hawaiian words used in this revision outside of formal taxonomic procedures are presented in their Hawaiian form with included macrons, i.e. kahakō, and glottal stops, i.e. ‘okina. Pukui et al. (1974, 1975) served as reference sources for Hawaiian words and formal place names. Gagné and Cuddihy (1990) and Starr and Starr (2015) were consulted for Hawaiian native plant names.

## Key to the species groups of *Mecyclothorax* Sharp from Haleakalā volcano, Maui, Hawai‘i

(based on the key to groups of Britton [1948b])

**Table:** 

1	All elytral striae uniformly impressed, never fainter nor absent near apex; 7^th^ stria always present and as distinctly impressed as other striae	**2**
1’	Outer elytral striae (except the 8^th^) less deeply impressed than those nearer suture, and usually fainter in apical half than towards base, often disappearing before reaching apex; one or more of the outer striae usually absent; 7^th^ stria, when present, much less deeply impressed than 8^th^ stria and striae on disc	**5**
2(1)	Elytral third interval consistently with two or three dorsal elytral setae each side	**3**
2’	Elytral third interval with one dorsal elytral seta each side (infrequently unilaterally bisetose; examine more than one specimen if possible)	***Mecyclothorax constrictus* group** (001–003)
3(2)	Larger, standardized body length including individuals > 3.9 mm (smaller specimens—3.6–3.9 mm—with irregularly fused elytral striae (Figs 44E, 52B) key to this couplet half)	**4**
3”	Smaller, standardized body length < 3.9 mm (larger specimens—3.9–4.3 mm—with elytral intervals 6–9 contrastedly flavous relative to piceous intervals 1–5 (Figs 15, 20) key to this couplet half)	***Mecyclothorax obscuricornis* group** (004–015)
4(3)	Elytral intervals slightly to moderately convex, striae minutely to distinctly punctate, greater strial punctation associated with more convex intervals; elytral striae regular, linear, adjacent striae united or approaching only where they fuse apically	***Mecyclothorax robustus* group** (016-026)
4’	Elytral intervals moderately to very convex, striae impunctate to minutely punctate, strial punctation and interval convexity not associated; striae may irregularly approach or anastomose on disc; this may involve fusion or approach of adjacent striae at dorsal elytral setae, or fusion or approach of striae 3 and 4, or 5 and 6 on elytral base	***Mecyclothorax interruptus* group** (027–035)
5(1)	First and 2^nd^ elytral striae subequally impressed at apex	**6**
5’	First elytral stria much more markedly impressed near apex than 2^nd^ stria	**9**
6(5)	Setiferous punctures of 3^rd^ elytral interval set in small depressions that are never as wide as the interval	**7**
6’	Setiferous punctures of 3^rd^ elytral interval set in obvious depressions that are as wide as or wider than interval	***Mecyclothorax sobrinus* group** (036–042)
7(6)	Eyes relatively smaller and less convex; beetles either larger, standardized body length 4.7–6.2 mm, with small to moderate eyes, ocular ratio 1.29–1.53, OR smaller, standardized body length 3.2–4.5 mm, with little convex eyes, ocular ratio 1.28–1.39	**8**
7’	Eyes well developed, larger with outer surface distinctly convex, standardized body length 3.3–4.6 mm, eyes moderately to very convex, ocular ratio 1.41–1.61	***Mecyclothorax ovipennis* group** (043–061)
8(7)	Standardized body length > 4.50 mm; elytra with parascutellar seta and both subapical and apical setae present, setal formula 2 2 2 2 or 2 2 3–4 2	***Mecyclothorax argutor* group** (062–069)
8’	Standardized body length < 4.50 mm; elytra without parascutellar, subapical, and apical setae; in some instances also bearing less than 2 dorsal elytral setae, setal formula 2 2 2 0, 2 2 1 0, or 2 2 0 0	***Mecyclothorax microps* group** (070–075)
9(5)	Second elytral stria as deeply impressed (or punctured) as sutural stria, at least in basal half; pronotal lateral marginal depression moderately narrow to broad, the margin upturned	**10**
9’	Second elytral stria less deeply impressed in basal half than sutural stria; pronotal lateral marginal depression very narrow, margin beadlike, especially so in species with evident second stria	***Mecyclothorax scaritoides* group** (076–084)
10(9)	Elytral microsculpture slightly to distinctly transverse, especially at sides (if microsculpture reduced and cuticle glossy, assess sculpticell shape at elytral apex); elytra usually with distinct humeri; lateral margin of pronotum usually with a short sinuation anterad hind angle; elytra reddish to black, usually without metallic reflection	**11**
10’	Elytral microsculpture isodiametric, even at the sides (if microsculpture reduced and cuticle glossy, assess sculpticell shape at elytral apex); elytra obovoid to ellipsoid, humeri narrowly rounded; sides of the pronotum with an elongate sinuation anterad hind angle; elytra dark brown to black, with a metallic blue reflection	***Mecyclothorax haleakalae* group** (085–090)
11(10)	Elytra dark brown to black, sometimes slightly aeneous, but without metallic green or blue reflection, surface often with distinct microsculpture; elytral intervals slightly to moderately convex, discal striae 1–3 to 1–6 well indicated, slightly to distinctly punctate; pronotal lateral margins moderately broad, the lateral margin upturned	**12**
11’.	Elytra dark brown to black but with metallic green or blue reflection, surface highly polished, microsculpture reduced to absent; elytral intervals nearly flat, discal striae little impressed, at most striae 1–2 minutely punctate on disc; pronotal lateral marginal depression very narrow, margin little upturned	***Mecyclothorax vitreus* group** (091–096)
12(11)	Pronotal lateral margins sinuate before hind angles outwards before basal margin distinct from lateral margins at angulate hind angle	**13**
12’	Pronotal lateral margins distinctly curved outwards before hind angles, posterior angles very obtuse, small and toothlike	***Mecyclothorax montivagus* group** (in part, 098–099)
13(12)	Pronotal base narrow, MPW/BPW = 1.43–1.68; discal elytral striae moderately developed, intervals moderately convex to flat	**14**
13’	Pronotum broad basally with explanate hind angles, MPW/BPW = 1.20–1.27; discal elytral striae well developed, deep between the distinct punctures, elytral intervals of striae 2–4 convex basally	***Mecyclothorax montivagus* group** (in part, 097)
14(13)	Elytra glossy, discal intervals without distinct microsculpture; elytra markedly convex, domed, lateral margins depressed relative to disc, lateral surfaces vertical adjoining lateral marginal depression	***Mecyclothorax ducalis* group** (100–105)
14’	Elytra bearing distinct microsculpture, from shallow transverse mesh on glossy elytral surface, to well-developed transverse mesh, sculpticell breadth 2–4× length; elytra moderately convex, lateral surfaces more gently sloped relative to disc, not vertically adjoining lateral margin depression	***Mecyclothorax palustris* group** (106–116)

### *Mecyclothorax constrictus* species group

**Diagnosis.** Species in this group are characterized by deep elytral striae extended from the suture to the lateral margin with only stria 7 slightly shallower, and small to moderate body size (standardized body length 3.8–4.8 mm). Beetles of the Haleakalā species exhibit elytra bearing a single anterior dorsal elytral seta each side (rarely two setae or no seta present), and they lack both apical elytral setae. The pronotum is cordate, with MPW/BPW = 1.52–1.66, and the pronotal lateral margins are parallel or convergent anterad the right to slightly acute hind angles.

**Membership and distribution.** Other than the Haleakalā species, this group is represented by four species on Moloka‘i (Liebherr 2007). Beetles of the Moloka‘i species deviate from those on Haleakalā by the presence of 2–3 dorsal elytral setae, and one of the two apical elytral setae.

#### Key to adults of the *Mecyclothorax constrictus* species group, Haleakalā volcano, Maui, Hawai‘i

**Table:** 

1	Basal portions of discal elytral intervals glossy, transverse sculpticells obsolete, not traceable at least near elytral basal groove, in some instances not visible in basal fifth of elytron; male aedeagal median lobe with short to moderately elongate apical extension (Fig. 11D–E, G, H)	**2**
1’	Basal portions of discal elytral intervals with distinct transverse-mesh microsculpture, the sculpticells evident even near elytral basal groove; male aedeagal median lobe with elongate, spatulate apical extension (Fig. 11A–B)	(001) ***Mecyclothorax perseveratus* sp. n.**
2(1)	Discal elytral striae 1–5 minutely punctate, the punctures longitudinal and not expanding breadth of striae (Fig. 10B); male aedeagal median lobe with parallel-sided apical extension (Fig. 11D–E, G)	(002) ***Mecyclothorax perstriatus* (Sharp)**
2’	Discal elytral striae 1-5 more distinctly punctate, the punctures rounder and slightly expanding breadth of striae (Fig. 10C); male aedeagal median lobe with very short apex, the dorsal and ventral surfaces of extension convergent to tightly rounded tip (Fig. 11H)	(003) ***Mecyclothorax superstriatus* Liebherr**

#### (001) Mecyclothorax perseveratus sp. n.

Taxon classificationAnimaliaColeopteraCarabidae

http://zoobank.org/DDE71786-1F59-4BD0-8AD4-4D82F36648F3

**Figure 10.**
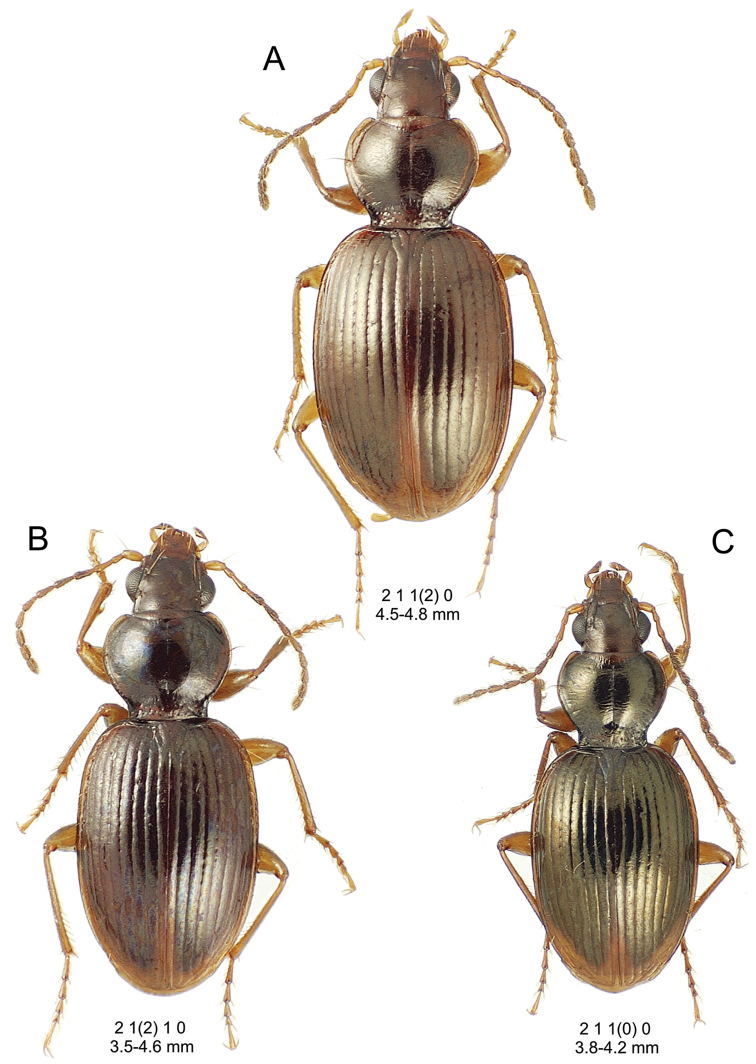
*Mecyclothorax constrictus* group species, dorsal habitus view. **A** *Mecyclothorax perseveratus* (Waikamoi, 1160 m) **B** *Mecyclothorax perstriatus* (Kahakapao, 960 m) **C** *Mecyclothorax superstriatus* (Polipoli, 1730 m).

**Figure 11.**
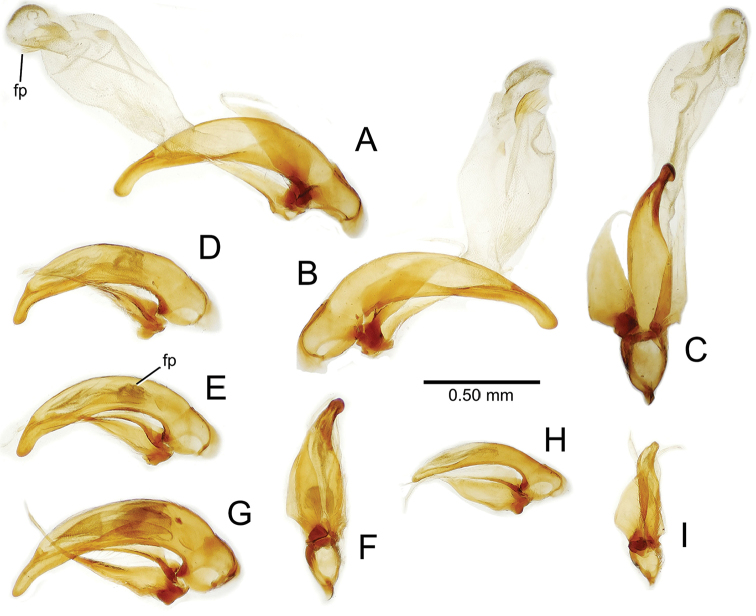
Male aedeagus, *Mecyclothorax constrictus* group species (for abbreviations see Table 2, p. 23). **A–C** *Mecyclothorax perseveratus*, right, left, and ventral views, sac everted (Waikamoi 1160 m) **D–G** *Mecyclothorax perstriatus* **D** Right view (Waikamoi, 1534-1660 m) **E** Right view (Kahakapao, 960 m) **F** Ventral view (Kahakapao, 960 m) **G** Right view (Waikamoi, 1300 m) **H–I** *Mecyclothorax superstriatus*, right and ventral views (Polipoli, 1730 m).

**Figure 12.**
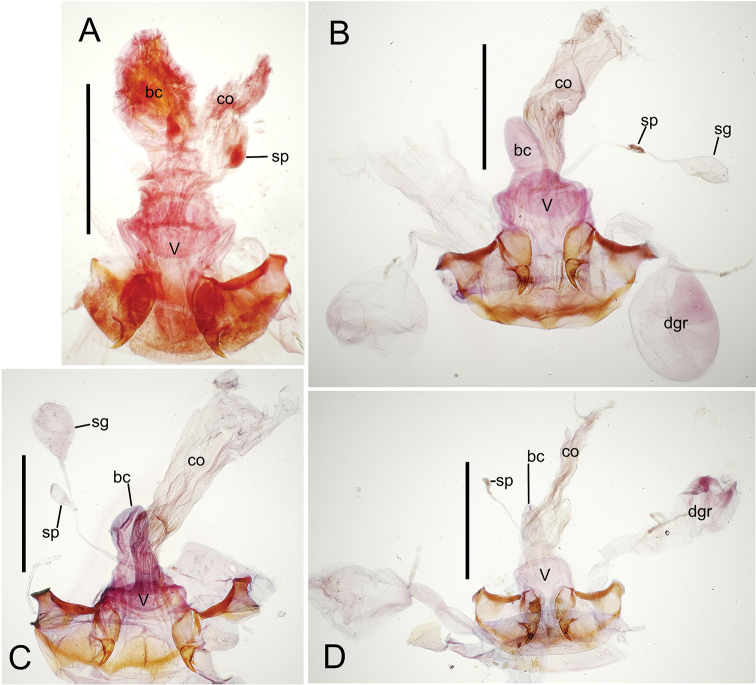
Female bursa copulatrix and associated reproductive structures, *Mecyclothorax constrictus* group species, ventral view (for abbreviations see Table 2, p. 23). **A** *Mecyclothorax perseveratus* (Waikamoi, 1160 m) **B** *Mecyclothorax perstriatus* (Kīpahulu, 1800 m) **C** *Mecyclothorax perstriatus* (Kuhiwa, 1600 m) **D** *Mecyclothorax superstriatus* (Polipoli, 1730 m). Scale bar = 0.50 mm.

**Figure 13.**
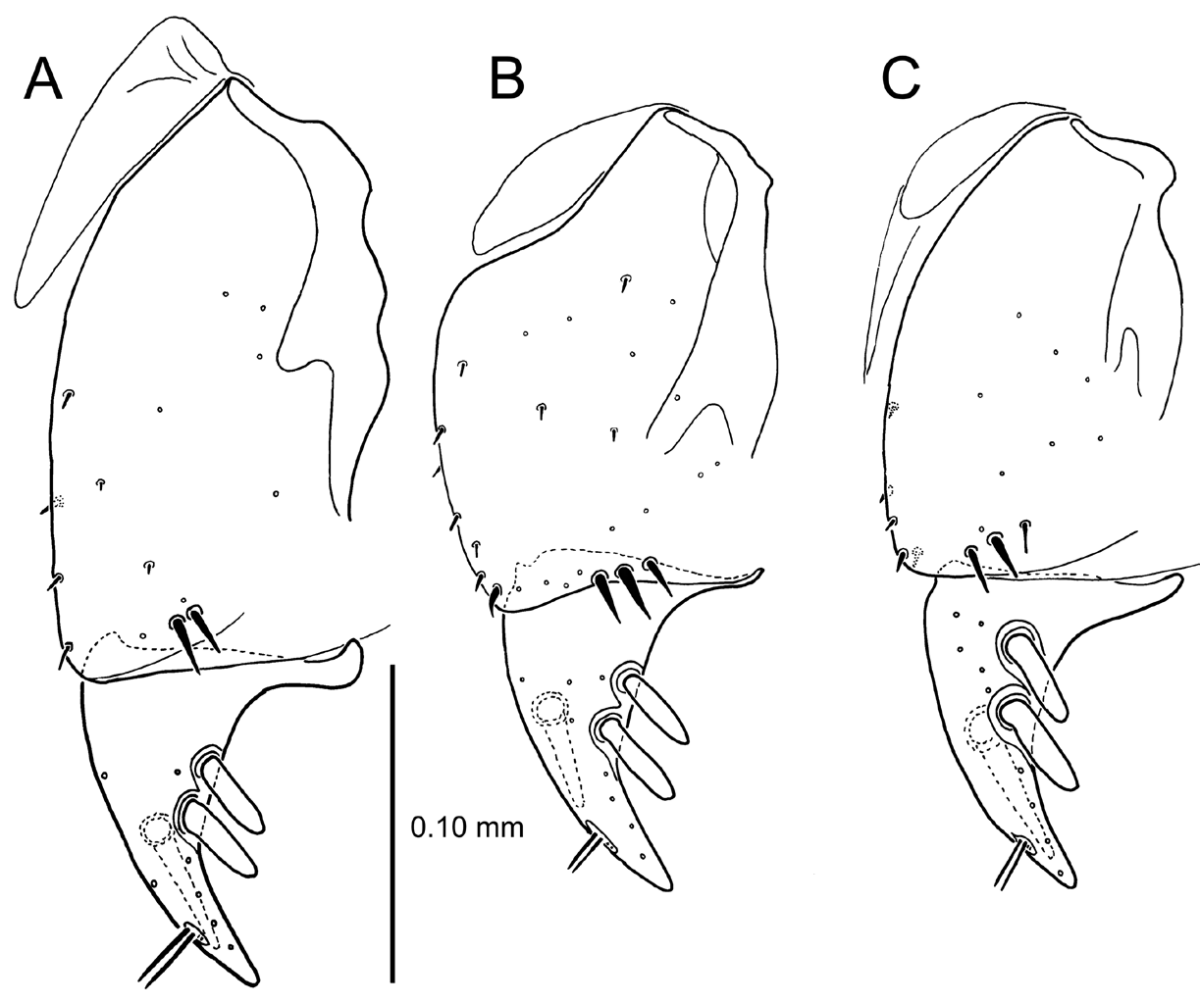
Left female gonocoxa, *Mecyclothorax constrictus* group species, ventral view. **A** *Mecyclothorax perseveratus* (Waikamoi, 1160 m) **B** *Mecyclothorax perstriatus* (Kuhiwa, 1600 m) **C** *Mecyclothorax superstriatus* (Polipoli, 1730 m).

**Figure 14.**
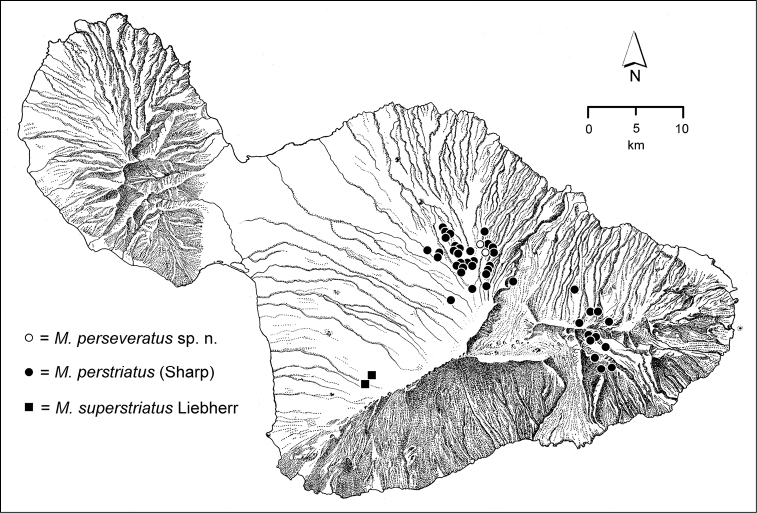
Recorded geographic distributions of *Mecyclothorax constrictus* group species.

Figs 10A , 11A–C , 12A , 13A , 14

##### Diagnosis.

This is the largest bodied of the three Haleakalā species in this group (Fig. 10A); standardized body length 4.5–4.8 mm versus 3.5–4.6 mm for the other two species below. Dorsal microsculpture is more developed in this species, with the elytral disc covered with an elongate transverse mesh and parallel lines, and the pronotal disc bearing an evident transverse mesh. Setal formula 2 1 1(2) 0.

##### Description

(n = 3). *Head capsule* with frontal grooves straight, bordered by a lateral carina and mesal wrinkles; neck flat to slightly concave; eyes convex, largely covering ocular lobe, ocular ratio 1.52–1.55, ocular lobe ratio 0.89–0.94; labral anterior margin very shallowly emarginate; antennae filiform, antennomeres 2-3 covered with sparse pelage of small setae; mentum tooth narrow with acute sides, apex tightly rounded. *Pronotum* with lateral seta present, basal seta absent; MPW/BPW = 1.52–1.66; MPW/PL = 1.11–1.23; hind angle right to slightly acute; lateral margin convergent to subparallel anterad hind angle; median base with small punctures, sparse medially, denser laterally; basal margin broadly, slightly convex between laterobasal depressions; median longitudinal impression finely incised, shallow; anterior transverse impression distinct, slightly punctate in middle half; anterior callosity slightly convex, traversed by shallow wrinkles; front angles slightly protruded, tightly rounded; APW/BPW = 1.0–1.04; lateral marginal depression moderately narrow, flat near front angle, edge upturned; laterobasal depression a continuation of lateral depression, surface irregularly punctate. *Proepisternum* with 6 small punctures along hind marginal groove; prosternal process with narrow median impression, lateral margins a narrow bead anterad. *Elytra* broadly ovoid, convex, suture elevated relative to disc; basal groove nearly straight laterad scutellum, humeral angle subangulate, defined by a hitch at base of lateral depression; humeri broadly rounded, MEW/HuW = 2.11–2.14; elytra broad relative to pronotum and head, MEW/MPW = 1.50–1.59, MEW/MHW = 2.18–2.29; parascutellar seta present; parascutellar striole with 2–4 punctures, striole shallow between punctures; juncture of sutural intervals each side upraised, producing a median callus; depth of sutural or first stria subequal to 2^nd^ stria from base to apex; discal stria finely punctate basally, narrow and smooth apically; lateral striae punctate basally, the punctures small, slightly expanding striae in basal half, punctures absent apically; intervals 2–7 moderately convex; all striae finely incised apically; 8^th^ interval laterad 7^th^ stria not more convex apically than other intervals; either 1 or 2 dorsal elytral setae, if two, then setae at 0.10× and 0.28–0.33× elytral length, if one seta, then situated 0.24–0.30× elytral length; apical and subapical setae absent; lateral elytral setae arranged as 7 + 6 (anterior and posterior series); elytral marginal depression narrow, lateral margin upturned; subapical sinuation broadly excavated, shallow, internal plica visible from dorsal view. *Mesepisternum* punctate, ~13 punctures in 2–3 vertical rows; metepisternal medial length/maximum width = 1.39; metepisternum/metepimeron suture distinct. *Abdomen* with irregular longitudinal wrinkles on ventrites 1–4, suture between ventrites 2 and 3 complete; males with 2 apical abdominal setae, females with 4 equally spaced setae plus 4 short setae arranged in a median trapezoid. *Legs*-metatarsomere 1/metatibial length ratio = 0.195; metatarsomere 4 lobe length 1.5× medial tarsomere length, subapical and apical setae present; metatarsal dorsolateral sulci very narrow, shallow, evident on mt1 and mt2 only. *Microsculpture* of head capsule transverse, vertex with transverse mesh and fine wrinkles; pronotal disc with evident, reflective transverse-mesh microsculpture; pronotal median base with reflective isodiametric mesh in transverse rows; elytral disc with elongate transverse mesh and parallel lines, apex with evident transverse mesh; metasternum covered with transverse mesh; laterobasal abdominal ventrites with swirling isodiametric and transverse sculpticells. *Coloration* of vertex brunneous with a slight piceous cast; antennomeres 1–3 flavous, 4–11 rufobrunneous; pronotal disc rufobrunneous, pronotal margins slightly, broadly paler; proepipleuron rufoflavous, proepisternum brunneous with piceous cast; elytral disc rufobrunneous, darkest behind middle, sutural interval paler, rufous basally, flavous apically; elytral margins concolorous with disc basally, broadly paler apically; elytral apex broadly flavous, flavous coloration extended anterad along suture; elytral epipleuron rufoflavous, metepisternum brunneous; abdominal ventrites 1–3 brunneous medially, more apical ventrites flavous; metafemur flavous, metatibia flavous with brunneous cast.

**Male genitalia** (n = 1). Aedeagal median lobe gracile, elongate, distance from parameral articulation to tip 4.7× depth at midlength (Fig. 11A); median lobe apex parallel sided with rounded tip, apex evenly downcurved distad ostial opening in lateral view (Fig. 11A–B), apex curved to right with top expanded as a knob in ventral view (Fig. 11C); internal sac elongate, flagellar plate small, sac covered with fine spicules only.

**Female reproductive tract** (n = 1). Bursa copulatrix columnar, base broad at vagina, narrower at midlength, bursal length 0.80 mm, breadth 0.40 mm at base (Fig. 12A); bursal surface translucent, wrinkled, not sclerotized; gonocoxite 1 with 2–3 apical fringe setae, 1 small seta at apicomedial angle, 4–5 setae along medial surface (Fig. 13A); gonocoxite 2 subacuminate with lateral panhandle extension; 2 moderately elongate lateral ensiform setae, 0.36× length of gonocoxite; apical nematiform setae on medial surface at 0.80× gonocoxite length.

##### Holotype.

Male (CUIC) labeled: HI: Maui Haleakala Kula / Pipeline Rd. W Waikamoi / Gulch 15-V-1998 lot04 / 1160 m el. pyr. fog mossy / ohia/ logs J.K. Liebherr // HOLOTYPE / Mecyclothorax / perseveratus / Liebherr / det. J.K. Liebherr 2015 (black-margined red label).

##### Paratypes.

HI: Maui: Koolau For. Res., Kula Pipeline Rd., pyrethrin fog *Metrosideros*/moss, 1160 m el., 15-v-1998 lot 06, Polhemus (NMNH, 1), wet forest, yellow pan trap, 1183–1280 m el., vi-viii-2006, Leblanc (CUIC, 1; UHIM, 1), Makawao Flume Rd., ecotone forest, yellow pan trap, 1293 m el., vi-viii-2006, Leblanc (CUIC, 1; UHIM, 2), Waikamoi flume tanks [labeled Waikamoi N.C.P.], 1275 m el., 30-v-1993, Tauber/Tauber (CUIC, 1).

##### Etymology.

The adjectival species epithet perseveratus is based on the verb perseverate; to repeat insistently or redundantly. Such a name could be appropriately applied for any number of Hawaiian *Mecyclothorax*, but it is used here as the name shares the first syllable with the following cryptic sibling species.

##### Distribution and habitat.

*Mecyclothorax perseveratus* is known to occur only in the Waikamoi drainage from 1160–1300 m elevation (Fig. 14). As such, the species is sympatric with lower elevational populations of *Mecyclothorax perstriatus*. Individuals have been collected from the trunks of moss-covered ‘ōhi‘a trees, as well as from mossy downed logs of the same species. The beetles must actively walk across the forest floor, as they have been collected from fluid-filled yellow-pan traps.

#### (002) Mecyclothorax perstriatus

Taxon classificationAnimaliaColeopteraCarabidae

(Sharp)

Figs 10B , 11D–G , 12B–C , 13B , 14

Thriscothorax perstriatus Sharp 1903: 260.
Mecyclothorax perstriatus , Britton 1948b: 158; Liebherr 2005b: 122.
Oopterus plicaticollis Boisduval, Karsch 1881: 1 (misidentification).
Thriscothorax modestus Sharp, Swezey 1954: 53 (misidentification, *Cibotium* associate).

##### Diagnosis.

Individuals of *Mecyclothorax perstriatus* and *Mecyclothorax superstriatus* share reduced microsculpture, the pronotal disc glossy with an obsolete transverse mesh visible over portions of the disc (Fig. 10B–C). However the elytral microsculpture is more developed in this species, being an evident, shallow transverse mesh versus an obsolete transverse mesh on a glossy surface in *Mecyclothorax superstriatus*. Beetles of both species also have smaller eyes that cover less of the ocular lobe than do those of *Mecyclothorax perseveratus*, with *Mecyclothorax perstriatus* exhibiting an ocular lobe ratio = 0.83–0.86. The male genitalia provide certain diagnosis, with the aedeagal median lobe apex of *Mecyclothorax perstriatus* males extended and downturned (Fig. 11D–G), versus very short and not at all downturned in *Mecyclothorax superstriatus* (Fig. 11H–I). Setal formula 2 1(2) 1 0; only one individual was observed within which the basal pronotal seta was unilaterally present. Standardized body length 3.5–4.6 mm.

##### Identification

(n = 5). The full description of *Mecyclothorax perseveratus* serves to describe this species with the exception of characters mentioned in the diagnosis. The eyes are convex; ocular ratio 1.51–1.55. The pronotum is cordate with right to acute hind angles; MPW/BPW = 1.54–1.65. The elytra are narrowly to more broadly ovoid; MEW/HuW = 2.09–2.23.

**Male genitalia** (n = 20). Aedeagal median lobe gracile but shorter than that of *Mecyclothorax perseveratus* males, distance from parameral articulation to tip 3.5–4.3× medial breadth (Fig. 11D–E, G); median lobe apex shorter than that of *Mecyclothorax perseveratus*, ventral margin angulate just distad ostial opening in lateral view; median lobe apex moderately curved to the right with tip slightly expanded in ventral view (Fig. 11F); internal sac with evident, short flagellar plate, but without evident microtrichial patches (uneverted specimens).

**Female reproductive tract** (n = 2). Bursa copulatrix bipartite, apex digitiform and narrower than base at vagina, bursa 0.46 mm long overall, base at vagina 0.42 mm broad, digitiform apical lobe 0.37 mm long, 0.14 mm broad (Fig. 12B–C); bursal surface membranous, translucent, finely wrinkled; gonocoxite 1 with 3 apical fringe setae, a moderate apicomedial seta at apex of medial surface, 5–6 setae basally on medial surface (Fig. 13B); gonocoxite 2 subacuminate with lateral extension, 2 gracile lateral ensiform setae, apical nematiform setae on medial surface at 0.75× gonocoxite length.

##### Lectotype.

Female (BMNH), designated by Liebherr (2005b: 122).

##### Misidentifications.

Karsch (1881) lists *Oopterus plicaticollis* Boisduval, with its collecting information recorded under MNHU lot number 60815 as “Olinda, 1, Dr. O. Finsch.” The single specimen is *Mecyclothorax perstriatus*, and so Karsch’s mention of the species represents a misidentification. Documentation of Swezey’s (1954) misidentification of *Mecyclothorax perstriatus* as *Mecyclothorax modestus* is supported by: 1, specimens that match his collecting date and host information that are; 2, not attributable to *Mecyclothorax cordaticollis*, the senior name synonymous below with *Mecyclothorax modestus*, but instead represent this species.

##### Distribution and habitat.

The recorded distribution of this species spans the western Waikamoi forests, the Hāna Bogs, upper Kīpahulu Valley, and the Manawainui Planeze (Fig. 14). It is a wet forest species, and has been found in association with ‘ōhi‘a, koa, or ‘ōlapa trees, or various ferns; *Asplenium*, *Athyrium* (‘akolea), *Cibotium* (hāpu‘u), or *Sadleria* (‘ama‘u). The beetles are active on vegetation at night, and also may be found by sifting litter. As with *Mecyclothorax perseveratus*, beetles have been collected from yellow-pan traps set on the ground, implying they active walk over the forest floor.

#### (003) Mecyclothorax superstriatus

Taxon classificationAnimaliaColeopteraCarabidae

Liebherr

Figs 10C , 11H–I , 12D , 13C , 14

Mecyclothorax superstriatus Liebherr 2005b: 118.

##### Diagnosis.

Superficially similar to the preceding two species, but individuals tend to be smaller—standardized body length 3.8–4.2 mm—with less developed microsculpture (Fig. 10C). The pronotal disc and discal elytral intervals are glossy, with only a very shallow transverse mesh discernible over portions of the cuticle. The male aedeagus is small (Figs 11H–I) with non-projected apex. Setal formula 2 1 1(0) 0; of 13 individuals assessed, 9 have the anterior dorsal elytral seta present both sides, 3 have the seta present unilaterally, and 1 lacks any dorsal elytral setae.

##### Identification

(n = 5). As *Mecyclothorax perstriatus* above, this species shares most characters with *Mecyclothorax perseveratus*. The eyes are convex, ocular ratio = 1.46–1.55, covering the ocular lobe as in *Mecyclothorax perstriatus*; ocular lobe ratio = 0.81–0.87. Being of smaller body size, the pronotum appears more constricted basally, though the MPW/BPW ratio range of 1.53–1.63 overlaps the values of the other two species. Elytral shape is also variable as in the other two species; MEW/HuW = 2.05–2.23.

**Male genitalia** (n = 1). Aedeagal median lobe short, apex barely extended beyond ostial opening, shaft thin, distance from parameral articulation to tip 5.7× depth at midlength (Fig. 11H); median lobe apex slightly curved to the right, tip not expanded in ventral view (Fig. 11I); internal sac with evident flagellar plate, but without other ornamentation.

**Female reproductive tract** (n = 1). Bursa copulatrix bipartite, apex digitiform and narrower than base at vagina, bursa 0.40 mm long overall, base at vagina 0.26 mm broad, digitiform apical lobe 0.17 mm long, 0.13 mm broad (Fig. 12D); bursal surface membranous, translucent, finely wrinkled; gonocoxite 1 with 3 apical fringe setae, a thin apicomedial seta at apex of medial surface, 4–5 setae more basally on medial surface (Fig. 13C); gonocoxite 2 subacuminate with lateral extension, 2 gracile lateral ensiform setae, apical nematiform setae on medial surface at 0.75× gonocoxite length.

##### Holotype.

Male (NMNH) dissected (Liebherr 2005b: 121). Type locality HI: Maui, Haleakalā, Polipoli S.R.A., 1770 m el.

##### Distribution and habitat.

*Mecyclothorax superstriatus* is restricted to the Polipoli Springs area along the southwest rift of Haleakalā (Fig. 14). They have been found from 1730–1770 m elevation, either in a deep ravine on a moist rock face that was covered with ferns and mosses, or in moss on a moist *Pinus radiata* log on a ravine floor. Both situations were among the moister microhabitats in the immediate area.

### *Mecyclothorax obscuricornis* species group

**Figure 15.**
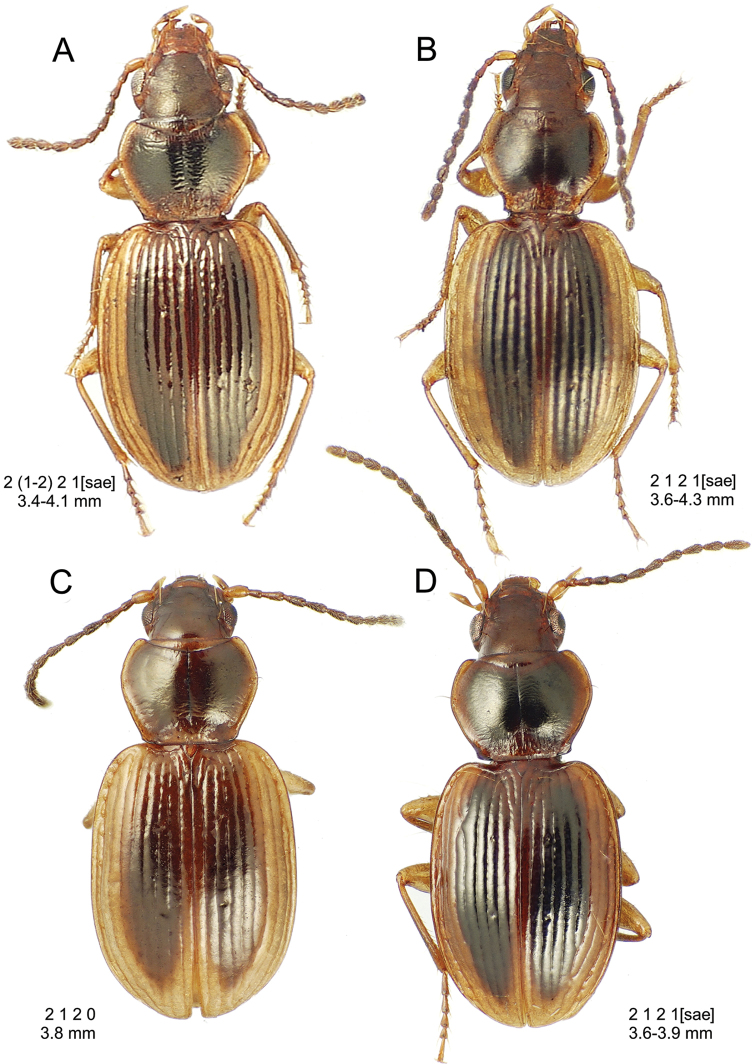
*Mecyclothorax obscuricornis* group species, dorsal habitus view. **A** *Mecyclothorax daptinus* (Ukulele Camp Pipeline, 1510 m) **B** *Mecyclothorax notobscuricornis* (Honomanu, 1700 m) **C** *Mecyclothorax mordax* (ESE Kuiki, 2164 m) **D** *Mecyclothorax mordicus* (Kuhiwa, 1780 m).

**Diagnosis.** Species of this group are characterized by small body size (standardized body length 3.2–4.3 mm), and elytral striae that are deep and equally well-developed from suture to lateral margin. The pronotum is moderately constricted basally, with MPW/BPW = 1.38–1.61. Both anterior and posterior dorsal elytral setae are present. The Haleakalā species exhibit two different coloration patterns: 1, pale pronotal and elytral margins contrasted with piceous discs (Figs 15, 20A); 2, more uniform coloration with margins concolorous to slightly paler (Figs 20B–D, 24).

**Membership and distribution.** This species group is restricted to three of the volcanoes comprising the former Maui Nui; East Moloka‘i with three species (Liebherr 2007), West Maui with four species (Liebherr 2011), and Haleakalā with the 12 species treated below.

#### Key to adults of the *Mecyclothorax obscuricornis* species group, Haleakalā volcano, Maui, Hawai‘i

**Table:** 

1	Elytral intervals 2–5 dark, piceous, and contrasted with rufous sutural interval and pale, testaceous intervals 7–9 (Figs 15, 20A)	**2**
1’	Elytral intervals concolorous, or sutural and lateral intervals only slightly paler than discal striae 2–5 (Figs 20B–D, 24)	**6**
2(1)	Pronotal disc smooth medially, the median longitudinal impression adjoined by at most shallow wrinkles (Figs 15B–D, 20A)	**3**
2’	Pronotal disc covered with rugose transverse wrinkles, pronotal surface adjacent to the median longitudinal impression irregular (Fig. 15A)	(004) ***Mecyclothorax daptinus* Sharp**
3(2)	Pronotal base moderately broad, MPW/BPW = 1.42–1.53, pronotal lateral margins briefly subparallel or divergent anterad hind angles	**4**
3’	Pronotum constricted basally, MPW/BPW = 1.57–1.61, pronotal lateral margins subparallel to slightly convergent anterad hind angles	(005) ***Mecyclothorax notobscuricornis* sp. n.**
4(3)	Pronotal hind angles distinct, lateral margins subparallel to sinuate before angles; pronotal disc pearlaceous due to distinct transverse-mesh microsculpture, sculpticell breadth 2× length	**5**
4’	Pronotal hind angles indistinct, subangulate, lateral margins divergent anterad angles; pronotal disc glossy, covered with shallow transverse-mesh microsculpture, sculpticell breadth 3× length	(006) ***Mecyclothorax mordax* sp. n.**
5(4)	Eyes moderate, ocular lobe ratio = 0.75–0.77, outer surface more convex, ocular ratio = 1.44–1.55; elytra subquadrate, humeri broader relative to maximum breadth, MEW/HuW = 1.81–1.86 (Fig. 15D)	(007) ***Mecyclothorax mordicus* sp. n.**
5’	Eyes smaller, ocular lobe ratio = 0.71–0.74, outer surface less convex, ocular ratio = 1.40–1.43; elytra subovoid, humeri narrower relative to broadly rounded margin behind, MEW/HuW =1.98–2.03 (Fig. 20A)	(008) ***Mecyclothorax manducus* sp. n.**
6(1)	Body moderately broad, eyes convex or not, MEW/MHW = 1.95–2.13 (largest values in species with less convex eyes); pronotum and elytra with margins narrowly paler, femora contrastedly paler, rufoflavous (Figs 20C–D, 24)	**7**
6’	Body broad, robust, eyes convex, MEW/MHW = 2.15; head, pronotum and elytra dark, fuscous, legs hardly paler (Fig. 20B)	(009) ***Mecyclothorax ambulatus* sp. n.**
7(6)	Pronotum bisetose, the lateral setae present and basal setae absent, elytral lateral margins convex near midlength, the elytra variously orbicular (Figs 20D, 24); pronotal base with distinct punctures, which may be accompanied by rugose wrinkles	**8**
7’	Pronotum quadrisetose, both lateral and basal setae present, elytral lateral margins straight, though divergent, near elytral midlength (Fig. 20C); pronotal base slightly rugose but without distinct punctures	(010) ***Mecyclothorax montanus* sp. n.**
8(7)	Vertex with well-developed microsculpture, either isodiametric or transverse mesh mixed with isodiametric sculpticells	**9**
8’	Vertex glossy, with obsolete transverse-mesh microsculpture visible only over portions of surface	**10**
9(8)	Discal elytral striae smooth, intervals broadly convex; elytra orbicular, lateral margins broadly convex (Fig. 20D)	(011) ***Mecyclothorax obscuricolor* (Blackburn)**
9’	Discal elytral striae punctate, the associated elytral intervals moderately convex; elytra elongate, the lateral margins moderately convex (Fig. 24A)	(012) ***Mecyclothorax obscuricornis* Sharp**
10(8)	Discal elytral striae 1–6 moderately punctate, punctures evident but not deep, little expanding strial breadth (Fig. 24C–D); eyes more convex, ocular ratio = 1.43–1.51, larger in diameter, ocular lobe ratio = 0.79–0.86	**11**
10’	Discal elytral striae 1–6 distinctly punctate, punctures deep, expanding strial breadth (Fig. 24B); eyes less convex, ocular ratio = 1.38–1.47, smaller in diameter, ocular lobe ratio = 0.79–0.81	(013) ***Mecyclothorax waikamoi* sp. n.**
11(10)	Pronotal hind angles protruded, lateral margins straight for short distance anterad angles (Fig. 24C); male aedeagal median lobe with parallel-sided apex, terminated ventrally as a narrowly rounded tip (Fig. 26B–D)	(014) ***Mecyclothorax poouli* sp. n.**
11’	Pronotal hind angles less protruded, lateral margins divergent immediately anterad angles (Fig. 24D); male aedeagal median lobe with very short, broad apex terminated ventrally as a subangulate tip (Fig. 26E)	(015) ***Mecyclothorax ahulili* sp. n.**

#### (004) Mecyclothorax daptinus

Taxon classificationAnimaliaColeopteraCarabidae

Sharp

**Figure 16.**
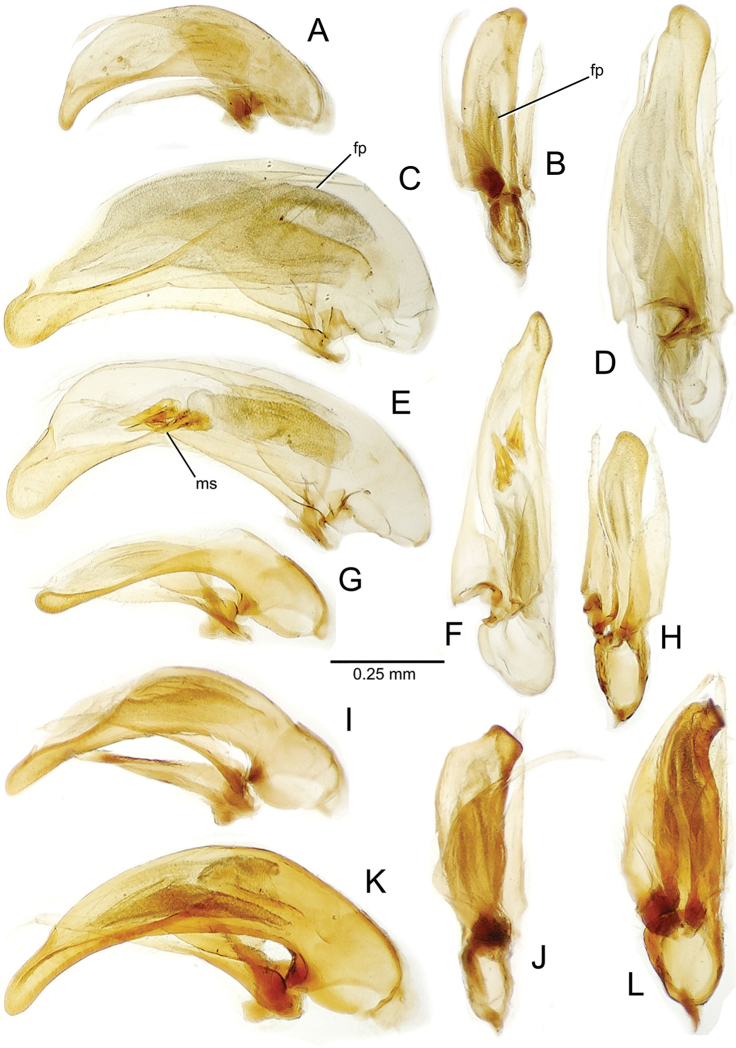
Male aedeagus, *Mecyclothorax obscuricornis* group species (for abbreviations see Table 2, p. 23). **A–B** *Mecyclothorax daptinus*, right and ventral views (Ukulele Camp, 1525 m) **C–D** *Mecyclothorax notobscuricornis*, right and ventral views (Honomanu, 1700 m) **E–F** *Mecyclothorax mordax*, right and ventral views (ESE Kuiki, 2164 m) **G–H** *Mecyclothorax mordicus*, right and ventral views (Kuhiwa, 1780 m) **I–J** *Mecyclothorax manducus*, right and left views (Kīpahulu, 915 m) **K–L** *Mecyclothorax ambulatus*, right and ventral views (Haleakalā, 1500 ft., RCLP).

**Figure 17.**
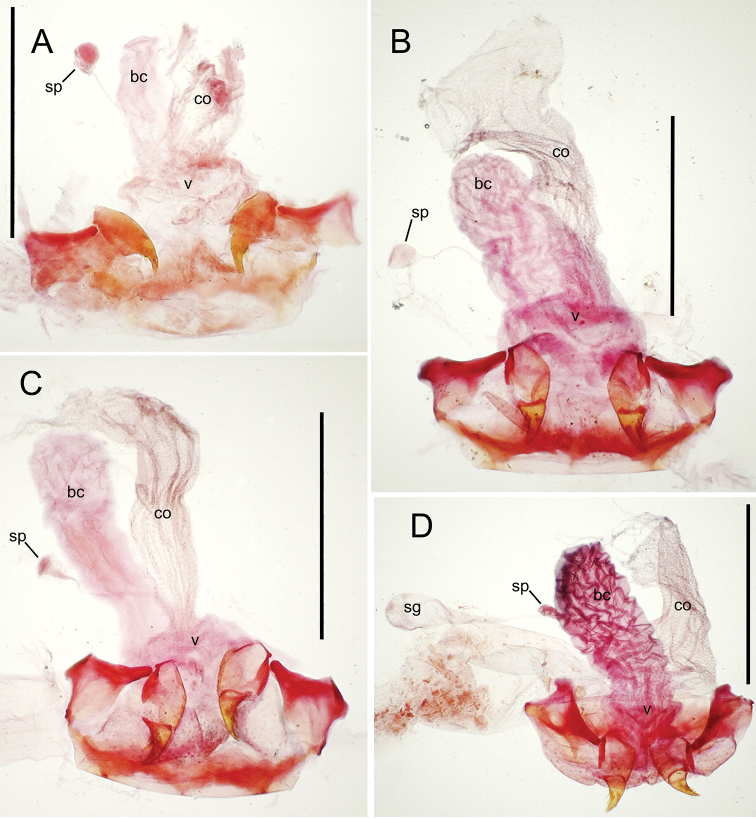
Female bursa copulatrix and associated reproductive structures, *Mecyclothorax obscuricornis* group species, ventral view (for abbreviations see Table 2, p. 23). **A** *Mecyclothorax daptinus* (Ukulele Camp, 1525 m) **B** *Mecyclothorax notobscuricornis* (Haipua‘ena, 455 m) **C** *Mecyclothorax manducus* (Kīpahulu, 910 m) **D** *Mecyclothorax montanus* (ESE Kuiki, 2145 m). Scale bar = 0.50 mm.

**Figure 18.**
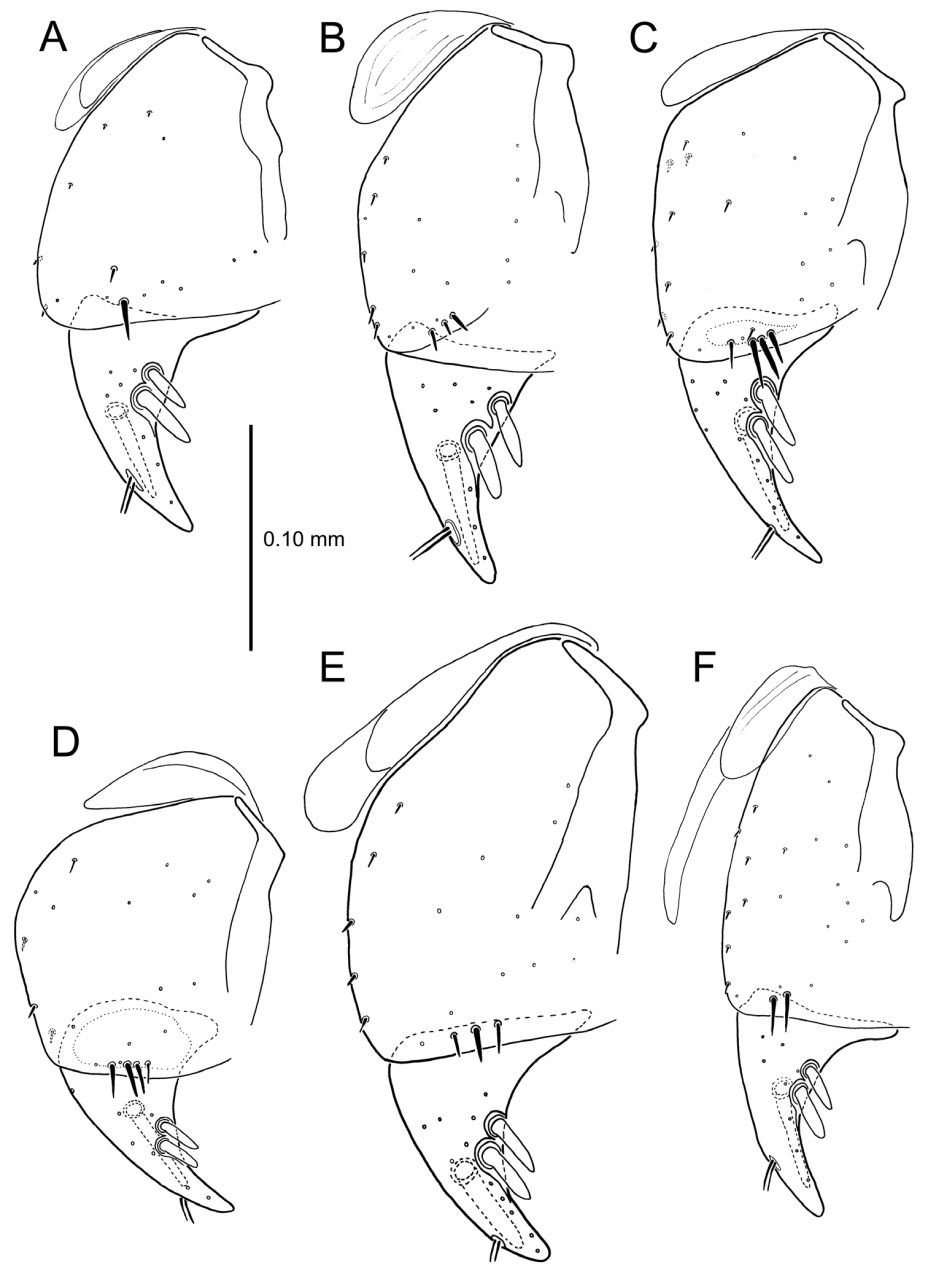
Left female gonocoxa, *Mecyclothorax obscuricornis* group species, ventral view. **A** *Mecyclothorax daptinus* (Ukulele Camp, 1525 m) **B** *Mecyclothorax notobscuricornis* (Haipua‘ena, 455 m) **C** *Mecyclothorax manducus* (Kīpahulu, 910 m) **D** *Mecyclothorax montanus* (ESE Kuiki, 2145 m) **E** *Mecyclothorax obscuricolor* (Ukulele Camp, 1525-1960 m) **F** *Mecyclothorax obscuricornis* (Ukulele Camp, 1525 m).

**Figure 19.**
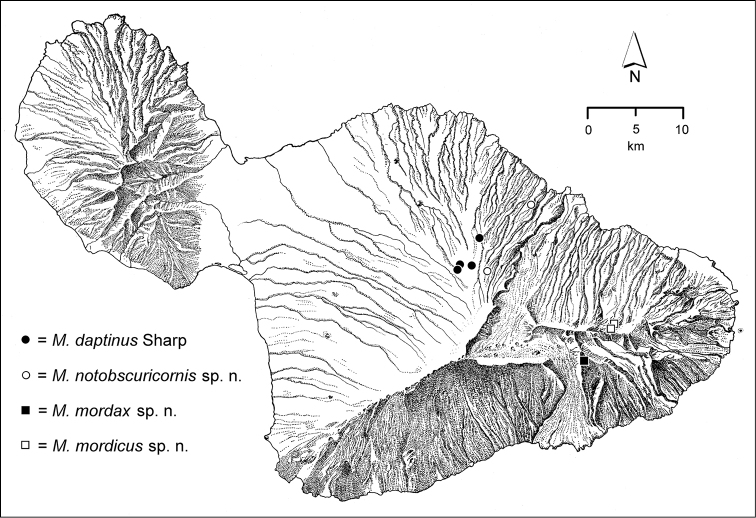
Recorded geographic distributions of *Mecyclothorax obscuricornis* group species.

Figs 15A , 16A–B , 17A , 18A , 19

Mecyclothorax daptinus Sharp 1903: 249; Britton 1948b: 160.

##### Diagnosis.

This species is easily diagnosed by the pale pronotal and elytral margins contrasted with piceous discal areas and the rugose transverse wrinkles of the pronotal disc (Fig. 15A). The pronotum is moderately constricted basally–MPW/BPW = 1.44–1.59–and the forebody dorsal surface bears well-developed microsculpture. The vertex is covered with upraised isodiametric sculpticells in transverse rows, and the pronotal disc is covered with a transverse mesh, sculpticell breadth 2–3× length. Conversely, the elytral discal intervals are glossy, with a transverse mesh to transverse lines toward the lateral elytral margins. Setal formula 2 (1-2) 2 1[sae]; of the five individuals scored, two have both lateral and basal pronotal setae, and three have only the lateral pair of setae. Standardized body length 3.4–4.1 mm.

##### Identification

(n = 5). Beetles of this species are of stocky stature, with short, submoniliform antennal segments and short legs. The eyes are moderately convex, ocular ratio = 1.44–1.52, but they cover only the anterior portion of the protruded ocular lobes; ocular lobe ratio = 0.72–0.80. In addition to the transverse wrinkles on the pronotal disc, the deep anterior transverse impression and elevated and flat anterior callosity are crossed by dense longitudinal wrinkles. The elytra are subquadrate with tightly rounded humeral angles—MEW/HuW = 1.89–1.92—and the discal elytral intervals are convex, the associated striae 2–3 minutely punctate basally, striae 4–8 with only minute irregularities.

**Male genitalia** (n = 2). Aedeagal median lobe robust, short, distance from parameral articulation 2.8× depth at midlength, apex briefly extended and evenly downturned beyond ostial opening (Fig. 16A); median lobe not curved to the right, though left side more broadly curved to rounded tip in ventral view (Fig. 16B); a moderate sized flagellar plate visible in uneverted specimen (Fig. 16B), no other sac ornamentation evident.

**Female reproductive tract** (n = 1). Bursa copulatrix basally broad, apically digitiform, overall length 0.43 mm, basal breadth 0.30 mm, apical lobe breadth 0.11 mm (Fig. 17A); bursal walls membranous, thin, transparent; gonocoxite 1 with 2 apical fringe setae, medial seta smaller, 1–2 small setae on medial surface (Fig. 18A); gonocoxite 2 subtriangular, apex subacuminate, base evenly extended from curved lateral surface, with 2 lateral ensiform setae, apical seta broader and longer, apical nematiform setae on medioventral surface at 0.71× gonocoxite length.

##### Lectotype.

Female (BMNH) hereby designated, labeled: Mecyclothorax daptinus Type D.S. Haleakala Perkins 113 (on mounting platen) // Type // Hawaiian Is. Perkins 1904-336 // Haleakala Maui 5000 ft. Perkins IV 1894 // LECTOTYPE Mecyclothorax daptinus Sharp J.K. Liebherr 1998 (black-margined red label).

##### Distribution and habitat.

*Mecyclothorax daptinus* is distributed along the leeward edge of the Waikamoi forest (Fig. 19), with the only recent records resulting from the application of pyrethrin fog to a mossy koa trunk or a mossy ‘ōhi‘a log. All but one of the collections have come from mesic forest near the Ukulele Camp site—~1500 m elevation—with the exception being a site on the Kula Pipeline Road at 1300 m elevation.

#### (005) Mecyclothorax notobscuricornis sp. n.

Taxon classificationAnimaliaColeopteraCarabidae

http://zoobank.org/F9F2268A-0D17-4403-9ECA-DF564C2A5832

Figs 15B , 16C–D , 17B , 18B , 19

##### Diagnosis.

Among the pale-margined members of this species group, *Mecyclothorax notobscuricornis* (Fig. 15B) stands out due to the basally constricted pronotum; MPW/BPW = 1.57–1.61. This is also the largest-bodied species in the group, with standardized body length 3.6–4.3 mm. Like *Mecyclothorax daptinus*, this species is characterized by well-developed forebody microsculpture and glossy elytra. The vertex is covered with well-developed isodiametric and transverse-mesh microsculpture, the pronotal disc by a well-developed transverse mesh, sculpticell breadth 2–3× length, whereas the discal elytral intervals are glossy, their surface covered with reduced transverse lines. Setal formula 2 1 2 1[sae].

##### Description

(n = 4). *Head capsule* with frontal grooves broad near clypeus, an associated lateral carina extended to anterior supraorbital seta; dorsal impression of neck flat to slightly concave; labral anterior margin broadly, shallowly emarginate; antennomeres 2-3 with sparse pelage of short setae; mentum tooth with sides acute, apex tightly rounded. *Pronotum* with lateral setae present, basal absent; hind angle obtuse to right, to slightly acute, margin rounded posterad angle; lateral margin subparallel to convergent anterad hind angle; median base depressed relative to disc, with fine longitudinal wrinkles; basal margin straight medially, expanded posterad laterobasal depressions; median longitudinal impression moderately deep at midlength, finely incised, extended onto median base; anterior transverse impression broad, shallow, crossed with longitudinal wrinkles, lined with granulate isodiametric microsculpture; anterior callosity slightly convex; front angles slightly projected, tightly rounded, more distant than hind angles, APW/BPW = 1.06–1.12; lateral marginal depression narrow throughout length, edge reflexed, beadlike margin only anterad and posterad hind angle; laterobasal depression smooth, continuous with lateral depression. *Proepisternum* with smooth hind marginal groove; prosternal process medially depressed, sides broadly upraised laterally. *Elytra* subovoid, disc moderately convex, lateral margins more so; basal groove distinctly recurved to subangulate humeral angle, MEW/HuW = 1.95–2.07; parascutellar seta present; parascutellar striole with 3–5 punctures, deep, continuous; sutural interval more convex than lateral intervals, sutural juncture elevated; sutural and 2^nd^ striae of subequal depth from base to apex; all striae deep, associated intervals convex; 7^th^ and 8^th^ interval similarly convex near apex; 2 dorsal elytral setae, anterior at 0.29×, and posterior at 0.58–0.61× elytral length, setae situated in depressions spanning 2/3 of interval 3; subapical seta present, apical seta absent; lateral elytral setae arranged in anterior series of 7 setae, posterior series of 5(6) setae; elytral marginal depression broadly reflexed, translucent posterad humerus, narrowed apically to beadlike margin anterad subapical sinuation; subapical sinuation shallow, more abruptly incurved anteriorly. *Mesepisternum* with 6 shallow punctures, most in 1 row; metepisternal width to length ratio = 0.82; metepisternum/metepimeron suture distinct. *Abdomen* with irregular wrinkles laterally on ventrites 1–5; suture between ventrites 2 and 3 reduced, effaced laterally; apical male ventrite with 2 apical setae, apical female ventrite with 4 equally spaced setae plus a median trapezoid of 4 small setae. *Legs*-metatarsomere 1/metatibial length ratio = 0.19; metatarsomere 4 length along outer lobe 1.25× medial tarsomere length, apical and subapical setae present; metatarsal dorsolateral sulci narrow, lateral, median area broad. *Microsculpture* of vertex well-developed isodiametric mesh in rows; pronotal disc with well-developed transverse mesh, sculpticell breadth 2–3× length; pronotal median base with upraised isodiametric and transverse mesh, transverse sculpticell breadth 2× length; elytral discal surface glossy, with reduced transverse lines; elytral apex with shallow transverse mesh and lines; metasternum with distinct transverse mesh; laterobasal abdominal ventrites with swirling isodiametric and transverse microsculpture. *Coloration* of vertex rufobrunneous; antennomere 1 flavous, antennomeres 2–3 rufous, 4–11 rufobrunneous; pronotal disc rufopiceous, lateral margins flavous, apex and base rufoflavous; proepipleuron rufoflavous, proepisternum rufobrunneous; elytral disc rufopiceous on intervals 2–5 to 2–6, base of interval 6 flavous; sutural interval rufobrunneous basally, rufoflavous apically; elytral intervals 7–9 pale, flavous, apex flavous to apical juncture of intervals 3 and 4; elytral epipleuron flavous dorsally, rufoflavous ventrally, metepisternum rufopiceous; abdominal ventrites 1–5 rufopiceous medially, 3–6 flavous laterally; apical half of apical ventrite 6 pale, flavous; metafemur flavous; metatibia rufoflavous with piceous cast.

**Male genitalia** (n = 1). Aedeagal median lobe broad, robust, distance from parameral articulation to tip 2.5× depth at midlength, apex broadly rounded and little extended beyond ostial opening (Fig. 16C); slightly curved to the right, the tip broadly rounded in ventral view (Fig. 16D); internal sac unornamented, large flagellar plate evident inside dorsal surface of median lobe dorsad parameral articulation (Fig. 11C).

**Female reproductive tract** (n = 1). Bursa copulatrix elongate, broad with rounded apex, length 0.57 mm, breadth at bursal-vaginal juncture 0.23 mm, maximum vagina breadth 0.35 mm (Fig. 17B); bursal walls translucent, thinly wrinkled; gonocoxite 1 with 3 apical fringe setae, 5 setae on medial surface (Fig. 18B); gonocoxite 2 subtriangular, apex tightly rounded, base with short broad lateral extension, 2 lateral ensiform setae, the apical seta broader and longer, apical nematiform setae on medioventral surface at 0.70× gonocoxite length.

##### Holotype.

Male (CUIC) dissected and labeled: HI: Maui Haleakala NW / slope Waikamoi Pres. / trans. 3 @ 1700 m el. / 10-V-1991 sifting / litter J.K. Liebherr // 2 // Mecyclothorax / notobscuricornis / ♂ #5 / det. J.K. Liebherr 2014 // HOLOTYPE / Mecyclothorax / notobscuricornis / Liebherr / det. J.K. Liebherr 2015 (black-margined red label).

##### Allotype.

Female (CUIC) labeled: HI: Maui Haleakala NW / slope Waikamoi Pres. / trans. 3 @ 1700 m el. / 8-V-1991 scraping / ohia w/ moss & dirt // J.K. Liebherr / collector // Mecyclothorax / notobscuricornis / ♀ photo / det. J.K. Liebherr 2014 // ALLOTYPE / Mecyclothorax / notobscuricornis / Liebherr / det. J.K. Liebherr 2015 (black-margined red label).

##### Paratypes.

Koolau F.R., Haipuaena, 455 m el., 30-vi-1920, Bryan (BPBM, 2).

##### Etymology.

The adjectival species epithet notobscuricornis is drawn from the converse name obscuricornis, another species in this group. Why Sharp (1903) used that name is unknown, though it too can be derived from the name of a previously described and related species; *Mecyclothorax obscuricolor* (Blackburn).

##### Distribution and habitat.

*Mecyclothorax notobscuricornis* (Fig. 19) is known from one recent collecting site, the upper Honomanu drainage at 1700 m elevation, and one historical 1920 collecting site near Haipua‘ena Camp along the Koolau Ditch (Wilcox 1996), 425 m elevation (BPBM, E.H. Bryan, Jr.). Recent records were from sifted leaf litter and from moss and associated humus on the trunk of a large ‘ōhi‘a tree.

#### (006) Mecyclothorax mordax sp. n.

Taxon classificationAnimaliaColeopteraCarabidae

http://zoobank.org/62B954AC-2B92-4FA9-B601-5C7918544E8B

Figs 15C , 16E–F , 19

##### Diagnosis.

Among the pale-margined species in this group, this species can be diagnosed by the obtuse, rounded pronotal hind angles, the lateral margins only slightly sinuate anterad the hind angles (Fig. 15C). The elytra are subquadrate, with the lateral margins broadly extended posterad the rounded humeri. The pronotal base is smooth, with the median base moderately depressed and sparsely covered with shallow punctures and longitudinal wrinkles. Setal formula 2120. Standardized body length 3.8 mm.

##### Description

(n = 1). *Head capsule* with frontal grooves broad at clypeus, a lateral carina present to anterior supraorbital seta; dorsal impression of neck flat to slightly concave; eyes little convex, ocular ratio = 1.37, ocular lobe ratio = 0.77; labral anterior margin with broad, shallow emargination; antennae filiform, antennomere 2 sparsely setose, antennomere 3 with well-developed pelage of short setae; mentum tooth with sides acute, apex rounded. *Pronotum* with lateral seta present, basal seta absent; pronotal base little constricted, MPW/BPW = 1.42; basal margin straight medially, expanded posterad laterobasal depressions; median longitudinal impression shallow, finely incised; anterior transverse impression deep, narrow, finely incised; anterior callosity slightly convex, glossy but crossed by indistinct wrinkles; front angles moderately projected, tightly rounded; apical and basal angles at subequal separation, APW/BPW = 0.99; lateral marginal depression narrow but edge reflexed anteriorly, broader at midlength, beaded at hind angle; laterobasal depression smooth, concave, continuous with lateral depression. *Proepisternum* with 5 minute punctures along hind marginal groove; prosternal process medially depressed, broadly upraised laterally. *Elytra* subquadrate, disc moderately convex, sides slightly more sloped; basal groove moderately recurved to meet rounded humeral angle; parascutellar seta present; parascutellar striole with 3–5 punctures, striole shallow between punctures; sutural interval more convex than lateral intervals, sutural juncture elevated; sutural and 2^nd^ striae of subequal depth from base to apex; striae 1–4 distinctly punctate basally, the punctures expanding strial breadth; intervals 2–8 convex to apex; 7^th^ and 8^th^ interval similarly convex mesad subapical sinuation; 2 dorsal elytral setae at 0.29× and 0.59× elytral length, setal impressions spanning 3^rd^ interval; apical and subapical setae absent; lateral elytral setae arranged as anterior series of 7 setae, and posterior series of 5–6 setae; elytral marginal depression narrow at humerus, posteriorly expanded laterally, broad to midlength, a narrow bead at subapical sinuation; subapical sinuation very shallow, nearly obsolete. *Mesepisternum* with ~8 shallow punctures arranged in 2–3 rows; metepisternal width to length ratio = 0.80; metepisternum/metepimeron suture distinct; metathoracic flight wing extended to posterior margin of metanotum. *Abdomen* with irregular lateral wrinkles on ventrites 1–4; suture between ventrites 2 and 3 complete; apical ventrite of male with 2 apical setae. *Legs*-metatarsomere 1/metatibial length ratio = 0.19; metatarsomere 4 length along outer lobe 1.33× medial tarsomere length, apical and subapical setae present; metatarsal dorsolateral sulci narrow, lateral, median area broad. *Microsculpture* of vertex shallow transverse mesh, sculpticell breadth 2× length; pronotal disc with shallow transverse mesh, sculpticell breadth 2× length; pronotal median base with shallow isodiametric and transverse-mesh microsculpture; elytral disc with shallow transverse mesh, sculpticell breadth 2× length; elytral apex with shallow transverse mesh, sculpticell breadth 2–3× length, plus transverse lines; metasternum with distinct transverse mesh; laterobasal abdominal ventrites with swirling isodiametric and transverse microsculpture. *Coloration* of vertex a glossy rufopiceous, antennomere 1 rufoflavous, antennomeres 2–3 rufoflavous, 4–11 rufobrunneous; pronotal disc rufopiceous, pronotal anterior callosity, lateral margins, and median base rufoflavous; proepipleuron flavous, proepisternum rufobrunneous with piceous cast; elytral disc with intervals 2–5 piceous from base to near apex, interval 6 rufous, and 7–9 flavous continuous with broadly flavous apex; sutural interval rufoflavous basally, flavous in apical 1/3; elytral epipleuron pale creamy ivory, metepisternum rufopiceous with piceous cast; abdominal ventrites 1–3 medially, and 4–6 mediobasally piceous, flavous laterally and apically; apical 2/3 of apical abdominal ventrite 6 flavous; metafemur flavous with piceous cloud covering basal half of anterior face; metatibia rufoflavous with brunneous cast.

**Male genitalia** (n = 1). Aedeagal median lobe robust (Fig. 16E), but thinner than that of *Mecyclothorax notobscuricornis* (Fig. 16C), distance from parameral articulation to tip 3.6× depth at midlength, apex extended more than its breadth beyond ostial opening, tip tightly rounded; median lobe narrowed apically and curved to the right in ventral view, tip appearing bluntly rounded (Fig. 16F); internal sac with field of 5 large macrospicules, flagellar plate large and visible in uneverted specimen just basad spicular field.

##### Holotype.

Male (CUIC) dissected and labeled: HI: Maui Haleakala N.P. / Kuiki, below el. 2164 m / N20°42.23', W156°08.00', / 16-V-2001 lot 05 sifting / ohia litter C.P. Ewing // Mecyclothorax / mordax / ♂ #1 / det. J.K. Liebherr 2014 // HOLOTYPE / Mecyclothorax / mordax / Liebherr / det. J.K. Liebherr 2015 (black-margined red label).

##### Etymology.

The adjectival epithet mordax means biting, corroding, or pungent, an appropriate name for a carabid beetle. But here it is also a play on the epithet daptinus—used by Sharp (1903) for a species of similar appearance—derived from the Greek dapto; to devour, or gnaw (Brown 1956).

##### Distribution and habitat.

The lone specimen of this species was found near Kuiki (Fig. 19) in leaf litter taken from below an isolated ‘ōhi‘a tree growing at timberline (e.g. Fig. 5A).

#### (007) Mecyclothorax mordicus sp. n.

Taxon classificationAnimaliaColeopteraCarabidae

http://zoobank.org/5CE64B17-DC53-4351-BE19-2837ECBA9590

Figs 15D , 16G–H , 19

##### Diagnosis.

**Figure 20.**
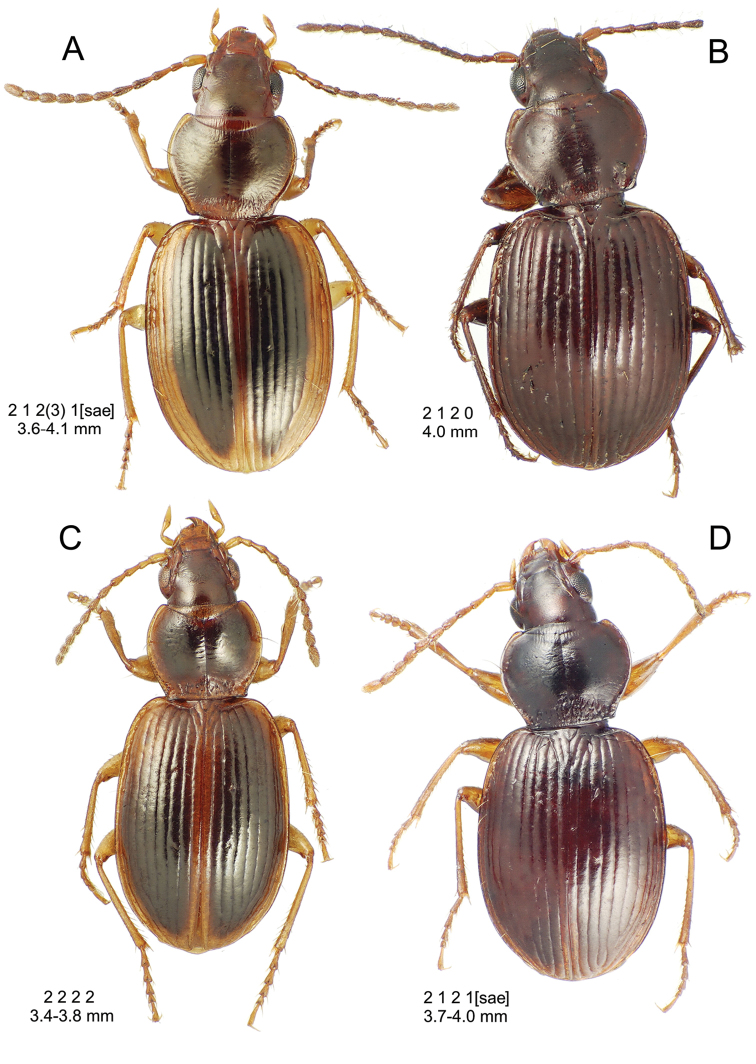
*Mecyclothorax obscuricornis* group species, dorsal habitus view. **A** *Mecyclothorax manducus* (Kīpahulu, 910 m) **B** *Mecyclothorax ambulatus* (Haleakalā, 1500 ft., RCLP) **C** *Mecyclothorax montanus* (ESE Kuiki, 2145 m) **D** *Mecyclothorax obscuricolor* (Ukulele Camp, 1525–1980 m).

Of the pale margined species in this group from Haleakalā that exhibit a smooth pronotal disc, sinuate lateral pronotal margins and a broader pronotal base—MPW/BPW = 1.42–1.52—this species can be diagnosed by more convex eyes—ocular ratio = 1.44–1.55—and the subparallel pronotal lateral margins and protruded hind angles (Fig. 15D). The most similar species is *Mecyclothorax manducus* (Fig. 20A), which deviates by exhibiting smaller eyes—ocular ratio = 1.40–1.43. If a male is available, the aedeagus can settle the matter, with *Mecyclothorax mordicus* exhibiting a median lobe with a very short rounded apex distad the ostial opening (Fig. 16G), whereas the median lobe apex of *Mecyclothorax manducus* is elongate (Fig. 16I). Setal formula 2 1 2 1[sae]. Standardized body length 3.6–3.9 mm.

##### Description

(n = 4). *Head capsule* with frontal grooves broad at clypeus, a lateral carina to anterior supraorbital seta; dorsal impression of neck flat to concave; ocular lobe ratio = 0.75–0.77; labral anterior margin broadly, shallowly emarginate; antennae filiform, antennomere with sparse setae, antennomere 3 with pelage of short setae; mentum tooth with sides acute, apex rounded. *Pronotum* with lateral seta present, basal seta absent; hind angle obtuse, margin rounded posterad; lateral margin subparallel anterad hind angle then divergent; median base moderately depressed, covered with dense longitudinal wrinkles and some punctures; basal margin straight medially, slightly expanded posteriorly mesad laterobasal depressions; median longitudinal impression shallow, finely incised; anterior transverse impression deep, broader medially, lined with granulate isodiametric microsculpture and crossed by longitudinal wrinkles; anterior callosity elevated, flat, crossed by longitudinal wrinkles; front angles little projected, tightly rounded; front angles slightly farther apart than hind angles; APW/BPW = 1.03–1.04; lateral marginal depression moderately narrow, edge upturned; laterobasal depression smooth, concave, surface continuous with lateral depression. *Proepisternum* with 5 minute punctures along hind marginal groove; prosternal process medially depressed, sides broadly upraised. *Elytra* subquadrate, MEW/HuW = 1.81–1.86; disc moderately convex, sides more sloped; basal groove angled anterad from 3^rd^ stria to meet tightly rounded humeral angle; parascutellar seta present; parascutellar striole with 3–5 punctures, striole shallow between punctures; sutural interval more convex than lateral intervals, sutural juncture upraised; sutural and 2^nd^ striae of subequal depth from base to apex; intervals 2-8 convex, associated striae deep, minute irregularities present at base, smooth on disc and at apex; 7^th^ and 8^th^ interval of similar convexity mesad subapical sinuation; 2 dorsal elytral setae situated at 0.30× and 0.60× elytral length, anterior setal impression spanning interval 3, posterior impression spanning ½ of interval width; subapical seta present, apical seta absent; lateral elytral setae arranged as anterior series of 7 setae and posterior series of 6 setae; elytral marginal depression moderately narrow at humerus, broader posterad, beadlike at subapical sinuation; subapical sinuation shallow, more abruptly incurved anteriorly. *Mesepisternum* with ~4 shallow punctures in one row; metepisternal width to length ratio = 0.81; metepisternum/metepimeron suture distinct. *Abdomen* with irregular lateral wrinkles on ventrites 1–5; suture between ventrites 2 and 3 complete; apical ventrite of male with 2 setae. *Legs*-metatarsomere 1/metatibial length ratio = 0.20; metatarsomere 4 length along outer lobe 1.25× medial tarsomere length, apical and subapical setae present; metatarsal dorsolateral sulci narrow, lateral, median area broad. *Microsculpture* of vertex isodiametric sculpticells in transverse rows; pronotal disc with transverse mesh, sculpticell breadth 2× length; pronotal median base with transverse mesh, sculpticell breadth 2× length; elytral disc glossy, transverse mesh to transverse lines present laterally; elytral apex with shallow transverse mesh, sculpticell breadth 2–3× length; metasternum with distinct transverse mesh; laterobasal abdominal ventrites with swirling isodiametric and transverse microsculpture. *Coloration* of vertex rufobrunneous; antennomere 1 flavous, antennomeres 2–3 rufoflavous, 4–11 rufobrunneous; pronotal disc rufopiceous, anterior callosity, lateral margins broadly, and median base rufoflavous; proepipleuron and proepisternum rufoflavous; elytral disc with intervals 2–5 piceous from base to apical 5/6, and interval 6 piceous on disc; sutural interval rufobrunneous basally and on disc, flavous in apical half; elytral intervals 7–9 plus marginal depression flavous, apex broadly flavous to juncture of intervals 3 and 4; elytral epipleuron pale creamy ivory, metepisternum rufobrunneous; abdomen with ventrites 1–3 medially and 4–6 mediobasally piceous; apical 2/3 of apical abdominal ventrite 6 flavous; metafemur flavous; metatibia flavous with brunneous cast.

**Male genitalia** (n = 2). Aedeagal median lobe gracile but with tip very broadly rounded and little extended beyond ostial opening, distance from parameral articulation to tip 4.4× depth at midlength (Fig. 16G); median lobe not curved to the right except near bluntly rounded tip in ventral view (Fig. 16H); internal sac unornamented, lightly sclerotized flagellar plate visible in lateral and ventral views (Figs 16G–H).

##### Holotype.

Male (CUIC) dissected and labeled: HI: Maui Haleakala Hana / For. Res. Ridge E / Heleleikeoha Str. 12-V- / 1998 lot 09 1760 m el. / sifting ohia litter C.P. / Ewing // 2 // Mecyclothorax / mordicus / ♂ #2 / det. J.K. Liebherr 2014 // HOLOTYPE / Mecyclothorax / mordicus / Liebherr / det. J.K. Liebherr 2015 (black-margined red label).

##### Paratypes.

HI: Maui, Haleakala, Hana For. Res., Horseshoe Bog, sifting *Metrosideros* litter, 1830 m el., 12-v-1998 lot 08 (CUIC, 1), Kuhiwa Str., beating *Myrsine lessertiana*, 1780 m el., 12-v-1009 lot 13 (CUIC, 1), same data a holotype (CUIC, 1).

##### Etymology.

The adjectival mordicus means biting in the mandibular sense, continuing the string of epithets that started with daptinus above.

##### Distribution and habitat.

Recorded localities of *Mecyclothorax mordicus* are restricted the Hāna Bogs region, specifically to proximate locales at Horseshoe Bog and near the headwaters of Kuhiwa and Helele‘ike‘oha Streams (Fig. 19). Beetles of this species have been found in ‘ōhi‘a litter, and by beating *Myrsine lessertiana* (kolea lau nui).

#### (008) Mecyclothorax manducus sp. n.

Taxon classificationAnimaliaColeopteraCarabidae

http://zoobank.org/F4C06E91-20FC-4BA2-88EC-9FF07E685A87

**Figure 21.**
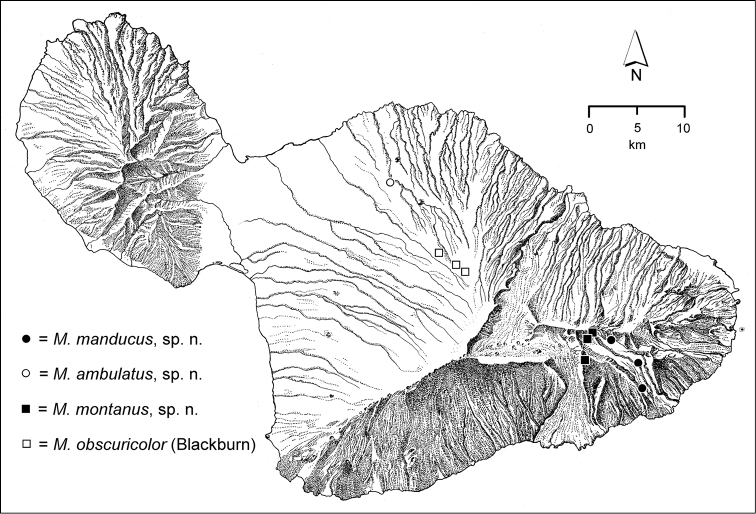
Recorded geographic distributions of *Mecyclothorax obscuricornis* group species.

Figs 16I–J , 17C , 18C , 20A , 21

##### Diagnosis.

Among the pale-margined species of this group, the slightly sinuate pronotal lateral margins associated with the obtuse pronotal hind angles of this species (Fig. 20A) are intermediate to the non-sinuate lateral margins of *Mecyclothorax mordax* (Fig. 15C) and the distinctly sinuate lateral margins of *Mecyclothorax mordicus* (Fig. 15D). This species is also characterized by less convex eyes—ocular ratio = 1.40–1.43—versus the more convex eyes of the other two species. Setal formula 2 1 2(3) 1[sae]; a third unilaterally present dorsal elytral seta was observed in one individual. Standardized body length 3.6–4.1 mm.

##### Description

(n = 5). *Head capsule* with frontal grooves broad near clypeus, a lateral carina present to anterior supraorbital seta; dorsal impression of neck flat to slightly concave; ocular lobe projected posteriorly, eye small, ocular lobe ratio = 0.71–0.74; labral anterior margin broadly, shallowly emarginate, antennomere 2 sparsely setose, antennomere with well-developed pelage of short setae; antennae filiform; mentum tooth with sides acute, apex tightly rounded. *Pronotum* with lateral seta present, basal seta absent; median base moderately depressed relative to disc, minutely punctate, minute wrinkles present on disc; basal margin straight medially, expanded posteriorly mesad laterobasal depressions; median longitudinal impression narrow, shallowly incised, continuous to basal margin; anterior transverse impression deep, narrow, surface behind with granulate isodiametric microsculpture, crossed by fine longitudinal wrinkles; anterior callosity slightly convex, glossy except for longitudinal wrinkles; front angles slightly projected, tightly rounded; APW/BPW= 1.00–1.04; lateral marginal depression narrow, edge upturned anteriorly, slightly broader, reflexed near base; laterobasal depression smooth, transversely wrinkled onto disc, continuous with lateral depression. *Proepisternum* with smooth hind marginal groove; prosternal process medially depressed, sides broadly upraised. *Elytra* subquadrate, disc moderately convex, sides more so; basal groove gently recurved to rounded humeral angle; MEW/HuW = 1.98–2.03; parascutellar seta present; parascutellar striole with three deep punctures, continuous between punctures; sutural interval more convex than lateral intervals, sutural juncture elevated; sutural and 2^nd^ striae of subequal depth from base to apex; striae 2–8 of similar depth, associated intervals convex; striae 1–3 with small punctures that expand stria, striae 4–5 with slight irregularities; 7^th^ and 8^th^ interval similarly convex mesad subapical sinuation; 2 dorsal elytral setae at 0.27× and 0.63× elytral length (unilateral third seta at 0.48× length), setal impressions spanning 2/3 of interval 3; subapical seta present, apical seta absent; lateral elytral setae arranged in anterior series of 7 setae, posterior series of 6 setae; elytral marginal depression narrow at humerus, expanded laterally along sides, narrowly beaded at subapical sinuation; subapical sinuation shallow, more abruptly incurved anteriorly. *Mesepisternum* with ~5 very shallow punctures; metepisternal width to length ratio = 0.76; metepisternum/metepimeron suture distinct. *Abdomen* with irregular lateral wrinkles on ventrites 1–5; suture between ventrites 2 and 3 reduced, effaced laterally; apical male ventrite with 2 marginal setae, female ventrite with 4 equally spaced marginal setae plus a median trapezoid of 4 smaller setae. *Legs*-metatarsomere 1/metatibial length ratio = 0.18; metatarsomere 4 length along outer lobe 1.25× medial tarsomere length, apical and subapical setae present; metatarsal dorsolateral sulci narrow, lateral, median area broad. *Microsculpture* of vertex a well-developed isodiametric mesh in transverse rows; pronotal disc with well-developed transverse mesh, sculpticell breadth 2–3× length; pronotal median base with well-developed, upraised transverse mesh, sculpticell breadth 2× length; elytral disc with transverse mesh, sculpticell breadth 2–3× length; elytral apex with mixture of transverse mesh and transverse lines; metasternum with distinct transverse mesh; laterobasal abdominal ventrites with swirling isodiametric and transverse microsculpture. *Coloration* of vertex rufobrunneous; antennomere 1 flavous, antennomeres 2–3 rufoflavous, 4–11 rufobrunneous; pronotal disc rufopiceous, pronotal margins broadly rufobrunneous, median base rufoflavous; proepipleuron rufoflavous, proepisternum rufobrunneous; elytral intervals 2–6 piceous from base to juncture of striae 3 and 4, outer intervals and apex flavous; sutural interval rufobrunneous basally, flavous in apical 1/3; elytral epipleuron rufoflavous, metepisternum rufobrunneous with piceous cast; abdomen with ventrites 1–5 rufopiceous medially, rufoflavous laterally; basal half of apical ventrite 6 rufopiceous, apical half flavous; metafemur flavous; metatibia flavous with brunneous cast.

**Male genitalia** (n = 2). Aedeagal median lobe moderately elongate, distance from parameral articulation to tip 3.7× depth at midlength (Fig. 16I); apical extension parallel sided, the tip subangulate at its ventral margin; median lobe apex curved to the right just before blunt tip in ventral view (Fig. 16J); internal sac unornamented, elongate flagellar plate visible inside dorsal margin of median lobe in lateral view.

**Female reproductive tract** (n = 1). Bursa copulatrix columnar, 0.57 mm long, 0.15 mm broad (Fig. 17C); bursal walls translucent, thinly wrinkled; gonocoxite 1 with 2–5 apical fringe setae, 8–9 small setae on medial surface (Fig. 18C); gonocoxite 2 subtriangular, apex acuminate; base evenly extended from lateral margin and basally curved at apex; 2 gracile lateral ensiform setae, apical nematiform setae on medial surface at 0.70× gonocoxite length.

##### Holotype.

Female (CUIC) labeled: HI:Maui Haleakala N.P. / Kipahulu Vy. Central / Pali Tr. 910 m el. / 30-IV-1991 sifting / moss and leaf litter // J.K. Liebherr / A.C. Medeiros, / Jr. collectors // Mecyclothorax / manducus / ♀ photo / Det. J.K. Liebherr 2014 // HOLOTYPE / Mecyclothorax / manducus / Liebherr / det. J.K. Liebherr 2015 (black-margined red label).

##### Paratypes.

HI: Maui: Haleakala N.P., Kipahulu Vy., sift litter, 1800 m el., 08-v-1991 lot 04, Jessel/Medeiros (CUIC, 1), above Bravo Camp above, sift litter, 600 m el., 01-v-1991 lot 03, Liebherr/Medeiros (CUIC, 1), Central Pali Tr., sift leaf/moss litter, 915 m el., 30-iv-1991 lot 03, Liebherr/Medeiros (CUIC, 16), under boards/logs/tarps, 915 m el., 30-iv-1991 lot 02, Liebherr/Medeiros (CUIC, 1).

##### Etymology.

This last of the species epithets related to biting, the Latin noun manducus means glutton (Brown 1956).

##### Distribution and habitat.

*Mecyclothorax manducus* is known only from Kīpahulu Valley (Fig. 21), though it occupies habitats from 600–1800 m elevation there. Records are all from ground-level microhabitats. These predominantly include 17 specimens in four Winkler sifter samples of leaf and moss litter (2–3 l each) taken from near large koa and ‘ōlapa trees, but also one beetle from under boards, tarps and logs at an abandoned fence-building camp.

#### (009) Mecyclothorax ambulatus sp. n.

Taxon classificationAnimaliaColeopteraCarabidae

http://zoobank.org/3E6C4AE5-5721-4CC1-826F-5872CF02E568

Figs 16K–L , 20B , 21

Mecyclothorax n. sp. δ, Liebherr 2004: fig. 4.

##### Diagnosis.

This species exhibits the most somber coloration in the group, the dorsal surface dark fuscous, and antennomeres 1–3 and femora only slightly paler (Fig. 20B). Elytral striae 1–5 are punctate basally, the punctures expanding strial breadth, a characteristic shared with the species triplet, *Mecyclothorax waikamoi*, *Mecyclothorax poouli*, and *Mecyclothorax ahulili* (Fig. 24B–D), however *Mecyclothorax ambulatus* exhibits larger body size; standardized body length 4.0 mm versus 2.9–3.4 mm in the other three species. Setal formula 2 1 2 0.

##### Description

(n = 1). *Head capsule* with frontal grooves broad near clypeus, a lateral carina present to anterior supraorbital seta; dorsal impression of neck flat to slightly concave; ocular ratio = 1.43, ocular lobe ratio = 0.81; labral anterior margin broadly, shallowly emarginate; antennae filiform, antennomere 2 sparsely setose, antennomere 3 with well-developed pelage of short setae; mentum tooth with sides acute, apex tightly rounded. *Pronotum* with lateral seta present, basal seta absent; MPW/BPW = 1.43; hind angle obtuse, apex rounded; lateral margin slightly divergent anterad hind angle, then more divergent anteriorly; median base moderately depressed relative to disc, sparsely covered with minute punctures, minute wrinkles present at juncture with disc; basal margin convexly expanded between laterobasal depressions; median longitudinal impression narrow, shallowly incised; anterior transverse impression narrow, finely incised, shallowest medially, crossed by fine wrinkles; anterior callosity elevated, flat, crossed by fine wrinkles; front angles slightly projected, tightly rounded; APW/BPW= 1.01; lateral marginal depression narrow, edge upturned anteriorly, beadlike at lateral sinuation and posterad laterobasal depressions; laterobasal depression smooth, continuous with lateral depression. *Proepisternum* with smooth hind marginal groove; prosternal process medially depressed, sides broadly upraised. *Elytra* broadly subquadrate; disc moderately convex, sides more so; basal groove recurved to subangulate humeral angle defined by a hitch at base of lateral marginal depression; MEW/HuW = 1.93; parascutellar seta present; parascutellar striole with 3 deep punctures, deep, continuous between punctures; sutural interval more convex than lateral intervals, sutural juncture elevated; sutural and 2^nd^ striae of subequal depth base to apex; striae 1–8 deep, interval 8 convex mesad subapical sinuation; 2 dorsal elytral setae at 0.28–0.30× and 0.61–0.63× elytral length, setal impressions moderately deep, spanning 2/3 of interval 3; subapical and apical setae absent; lateral elytral setae arranged in an anterior series of 7 setae and a posterior series of 5 setae; elytral marginal depression broad with upraised margin at humerus, gradually narrowed but still evident before subapical sinuation. *Mesepisternum* with ~7 punctures in 2–3 irregular rows; metepisternal width to length ratio = 0.80; metepisternum/metepimeron suture distinct. *Abdomen* with irregular lateral wrinkles on ventrites 1–5; suture between ventrites 2 and 3 reduced, effaced laterally; apical ventrite of male with 2 marginal setae. *Legs*-metatarsomere 1/metatibial length ratio = 0.19; metatarsomere 4 length along outer lobe 1.33× medial tarsomere length, apical and subapical setae present; metatarsal dorsolateral sulci narrow, lateral, median area broad. *Microsculpture* of vertex a well-developed isodiametric mesh in transverse rows; pronotal disc with well-developed transverse mesh, sculpticell breadth 2–3× length; pronotal median base with well-developed, upraised transverse mesh, sculpticells twice as broad as long; elytral disc with mixture of transverse mesh and transverse lines; elytral apex with mixture of transverse mesh–sculpticell breadth 3× length–and transverse lines; metasternum with distinct transverse mesh; laterobasal abdominal ventrites with swirling isodiametric and transverse microsculpture. *Coloration* of vertex dark rufobrunneous; antennomeres 1–3 rufobrunneous, 4–11 rufopiceous; pronotal disc and margins concolorous, dark rufobrunneous; proepipleuron rufoflavous, proepisternum rufobrunneous; elytral disc dark rufobrunneous, sutural interval concolorous basally, slightly paler, rufoflavous at apex, elytral marginal depression and apex slightly paler, rufoflavous; elytral epipleuron rufoflavous, metepisternum rufobrunneous; abdomen rufobrunneous, apical 1/6 of apical ventrite 6 paler, flavous; metafemur rufobrunneous with piceous cloud covering basal 4/4; metatibia rufoflavous with piceous cast.

**Male genitalia** (n = 1). Aedeagal median lobe broad, long, distance from parameral articulation to tip 3.3× depth at midlength (Fig. 16K); apex evenly curved, extended for 3× its breadth beyond ostial opening, tip slightly flattened on dorsal aspect; median lobe distinctly curved to the right just before blunt apex in ventral view (Fig. 16L); internal sac with dark microspicules over surface, a short flagellar plate visible inside dorsal margin in lateral view.

##### Holotype.

Male (BPBM) dissected, point mounted above original mounting platen, and labeled: Hal. Maui / 1500 ft. (on reverse) // obscuricolor / var. from lower / elevation. RCLP. // red rectangle // T. obscuricolor // Mecyclothorax / ambulatus / ♂ #1 / det. J.K. Liebherr 2014 // HOLOTYPE / Mecyclothorax / ambulatus / Liebherr / det. J.K. Liebherr 2015 (black-margined red label). The elevation of 1500 ft. along the Makawao-Paia road is designated the type locality.

##### Etymology.

The past participle ambulatus means to have travelled or traversed, and is used to signify R.C.L. Perkins’ discovery of the single known specimen at 1500 ft. elevation on Haleakalā. This low elevation collecting site is interpreted to have been along his walk from Makawao, the home base of his mountain collecting, to Paia, the village where he purchased groceries (Manning 1986).

##### Distribution and habitat.

*Mecyclothorax ambulatus* is a biogeographic relict, being labeled from Haleakalā, 1500 ft. by Perkins (Fig. 21). The designated type locality currently lies among agricultural fields and homesites near the town of Kamole, leading to the conclusion that this species is extinct near its only known locality.

#### (010) Mecyclothorax montanus sp. n.

Taxon classificationAnimaliaColeopteraCarabidae

http://zoobank.org/615BCB53-3AD7-49D8-BB62-D78FFE0C6EC5

**Figure 22.**
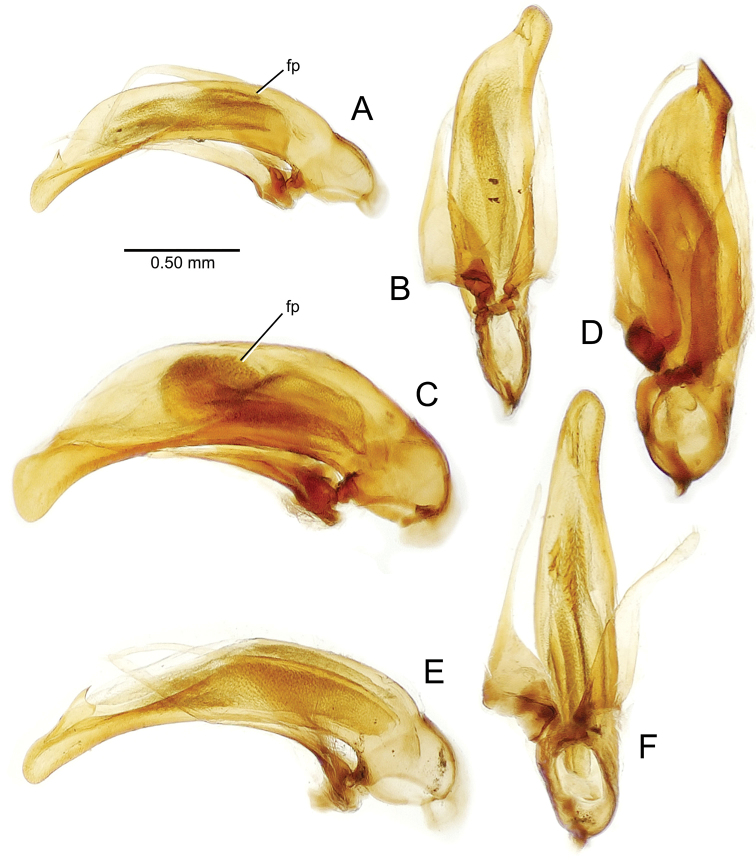
Male aedeagus, *Mecyclothorax obscuricornis* group species (for abbreviations see Table 2, p. 23). **A–B** *Mecyclothorax montanus*, right and ventral views (ESE Kuiki, 2145 m) **C–D** *Mecyclothorax obscuricolor*, right and ventral views (Ukulele Camp, 1525-1980 m) **E–F** *Mecyclothorax obscuricornis*, right and ventral views (Ukulele Camp 1525 m).

Figs 17D , 18D , 20C , 21 , 22A–B

##### Diagnosis.

The combination of quadrisetose pronotum, both lateral and basal setae present, subparallel to subovoid elytra, the lateral margins nearly straight along the anterior lateral setal series, and moderate body size, standardized body length, 3.4–3.8 mm, will diagnose this species. The pronotal and elytral margins are slightly paler than their respective discs (Fig. 20C), but not to the degree as in the paler-margined species treated above (Figs 15, 20A). This is also the only Haleakalā species of the group to exhibit both subapical and apical elytral setae; setal formula 2 2 2 2. Nevertheless, beetles of this species lack the parascutellar seta, and exhibit a reduced lateral elytral setal series, with base numbers of six anterior setae and five posterior setae.

##### Description

(n = 5). *Head capsule* with frontal grooves broad near clypeus, a lateral carina to anterior supraorbital seta; dorsal impression of neck flat to slightly concave; ocular lobe protruded, eyes small, ocular ratio = 1.36–1.38, ocular lobe ratio = 0.66–0.71; labral anterior margin broadly, shallowly emarginate; antennae submoniliform, antennomeres 2–3 sparsely setose; mentum tooth with sides acute, apex rounded. *Pronotum* quadrisetose, both lateral and basal setae present; MPW/BPW = 1.38–1.49; hind angle obtuse due to rounded margin behind; lateral margin parallel just anterad angle, then divergent; median base depressed relative to disc, a few large punctures and wrinkles present; basal margin broadly, slightly convex between laterobasal depressions; median longitudinal impression narrowly, shallowly incised, continuous to basal margin, adjoined by curved wrinkles emanating onto disc; anterior transverse impression deep, narrow, finely incised, crossed by fine longitudinal wrinkles; anterior callosity elevated, flat, crossed by fine wrinkles; front angles slightly projected, tightly rounded; APW/BPW = 0.94–1.03; lateral marginal depression narrow, edge upturned to beadlike at front, broader, less elevated to base; laterobasal depression surface impunctate with transverse wrinkles, continuous with lateral depression. *Proepisternum* with ~5 minute punctures along hind marginal groove; prosternal process medially depressed, sides broadly upraised. *Elytra* subparallel to subovoid, disc moderately convex, sides more so; basal groove evenly recurved to tightly rounded humeral angle; MEW/HuW = 1.80–2.00; parascutellar seta absent; parascutellar striole smooth, sinuous; sutural interval more convex than lateral intervals, sutural juncture upraised; sutural and 2^nd^ striae of subequal depth from base to apex; discal striae 1–7 with small punctures that cause strial irregularities; 7^th^ and 8^th^ interval of similar convexity mesad subapical sinuation; 2 dorsal elytral setae at 0.28–0.35× and 0.59× elytral length; setal impressions moderate, spanning 2/3 of interval 3; apical and subapical setae present; lateral elytral setae arranged in anterior series of 6 setae, and posterior series of 5 setae (rarely 4 or 6 setae); elytral marginal depression narrow from humerus to midlength, gradually narrowed to bead at subapical sinuation; subapical sinuation shallow, more abruptly incurved anteriorly. *Mesepisternum* with ~7 punctures in 2–3 rows; metepisternal width to length ratio = 0.80; metepisternum/metepimeron suture distinct; metathoracic flight wing length to width ratio = 2.2, remnant R and M veins present, wing tip extended 2/3 distance to hind margin of metanotum. *Abdomen* with irregular lateral wrinkles on ventrites 1–5; suture between ventrites 2 and 3 complete; apical male ventrite with 2 marginal setae, apical female ventrite with 4 equally spaced marginal setae and median trapezoid of 4–5 short setae (the 5^th^ seta, when present, shorter). *Legs*-metatarsomere 1/metatibial length ratio = 0.19; metatarsomere 4 length along outer lobe 1.28× medial tarsomere length, apical and subapical setae present; metatarsal dorsolateral sulci narrow, lateral, median area broad. *Microsculpture* of vertex very shallow, transverse, sculpticell breadth 2× length; pronotal disc with shallow transverse mesh, sculpticell breadth 3× length; pronotal median base with shallow isodiametric and transverse mesh; elytral disc with shallow transverse mesh, sculpticells 2–3× length; elytral apex with shallow transverse mesh, sculpticell breadth 2× length; metasternum with distinct transverse mesh; laterobasal abdominal ventrites with swirling isodiametric and transverse microsculpture. *Coloration* of vertex glossy rufopiceous; antennomeres 1–3 flavous, 4–11 only slightly darker; pronotal disc rufopiceous; pronotal lateral margins, anterior callosity and median base rufoflavous to flavous at outer margins; proepipleuron flavous, proepisternum rufobrunneous; elytral intervals 2–8 rufopiceous, sutural interval rufoflavous basally, flavous apically; interval 9, marginal depression, and apical margin rufoflavous; elytral epipleuron flavous dorsally, rufoflavous along ventral margin, metepisternum rufobrunneous; abdomen with ventrites 1–5 rufopiceous medially, rufoflavous laterally; apical ventrite 6 flavous in apical half; metafemur flavous; metatibia flavous with brunneous cast.

**Male genitalia** (n = 1). Aedeagal median lobe gracile, distance from parameral articulation to tip 4.5× depth at midlength (Fig. 22A), apex broad, extended twice its breadth beyond ostial opening, tip flattened on dorsoapical aspect; median lobe slightly curved to the right before rounded tip in ventral view (Fig. 22B); internal sac covered with evident microspicules, a flagellar plate visible inside dorsal margin in lateral view (Fig. 22A).

**Female reproductive tract** (n = 1). Bursa copulatrix columnar, nearly as broad as vagina, length 0.54 mm, breadth 0.23 mm (Fig. 17D); bursal walls translucent, distinctly wrinkled; gonocoxite 1 with 3–4 apical fringe setae, 4–5 smaller setae on medial surface (Fig. 18D); gonocoxite 2 subtriangular, apex acuminate, lateral surface distinctly curved, 2 small lateral ensiform setae, apical nematiform setae on medial surface at 0.75× gonocoxite length.

##### Holotype.

Male (CUIC) dissected and labeled: HI: Maui Haleakala N.P. / Kuiki, below el. 2134 m / N20°42.23', W156°08.00', / 16-V-2001 lot 02 sift. / litter under ohia lehua / J.K. Liebherr // Mecyclothorax / montanus / ♂ #1 / Det. J.K. Liebherr 2014 // HOLOTYPE / Mecyclothorax / montanus / Liebherr / det. J.K. Liebherr 2015 (black-margined red label).

##### Paratypes.

HI: Maui: Haleakala N.P., Kipahulu Vy., sift litter by day, 2100 m el., 07-v-1991 lot 05, Jessel/Medeiros (CUIC, 2), Mauka Ridge, pyrethrin fog *Metrosideros*/moss, 2045 m el., 21-v-1998 lot 01, Polhemus (NMNH, 1), Kuiki, below, sift *Metrosideros* litter, 2145 m el., 16-v-2001 lot 02, Liebherr (CUIC, 6), pyrethrin fog *Metrosideros*/moss, 2145 m el., 16-v-2001 lot 01, Liebherr (CUIC, 1).

##### Etymology.

The adjectival epithet montanus means of the mountains, which aptly fits the habits of this species distributed around the head of Kīpahulu Valley.

##### Distribution and habitat.

*Mecyclothorax montanus* is distributed in the upper slopes surrounding the head of Kīpahulu Valley (Fig. 21). Beetles have been found in sifted leaf litter from the forest floor, and also in leaf and moss litter adhering to trunks of ‘ōhi‘a.

#### (011) Mecyclothorax obscuricolor

Taxon classificationAnimaliaColeopteraCarabidae

(Blackburn)

**Figure 23.**
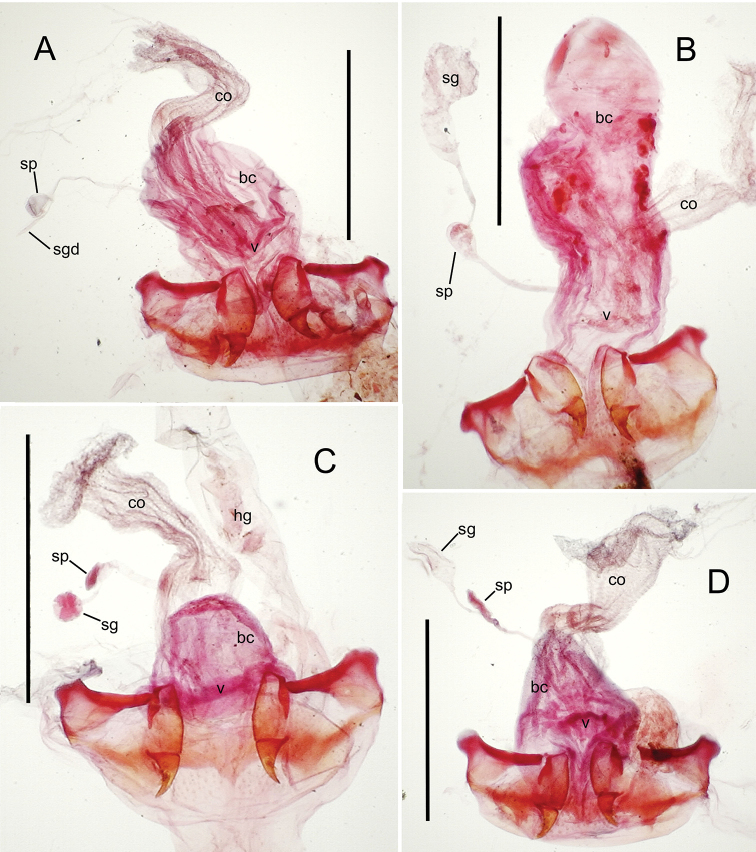
Female bursa copulatrix and associated reproductive structures, *Mecyclothorax obscuricornis* group species, ventral view (for abbreviations see Table 2, p. 23). **A** *Mecyclothorax obscuricolor* (Ukulele Camp, 1525–1980 m) **B** *Mecyclothorax obscuricornis* (Ukulele Camp, 1525 m) **C** *Mecyclothorax waikamoi* (Waikamoi, 1265 m) **D** *Mecyclothorax poouli* (Kuhiwa, 1590 m). Scale bar = 0.50 mm.

Figs 18E , 20D , 21 , 22C–D , 23A

Cyclothorax obscuricolor Blackburn 1878a: 123; Blackburn and Sharp 1885: 215.
Thriscothorax obscuricolor , Sharp 1903: 266.
Mecyclothorax obscuricolor , Britton 1948b: 160.

##### Diagnosis.

Of species in this group with concolorous pronotal discs and margins, this species exhibits the smoothest elytral striae, with only minute punctures basally in striae 1–7 that cause slight irregularities of the strial surface (Fig. 20D). The pronotal median base is moderately depressed relative to the disc, with longitudinal punctures and wrinkles producing a rough surface. The dorsal surface bears well-developed microsculpture, an isodiametric mesh on the vertex, and transverse mesh on pronotal and elytral discs with sculpticell breadth 2–3× length. Setal formula 2 1 2 1[sae]. Standardized body length 3.7–4.0 mm.

##### Identification

(n = 5). The eyes are little convex, ocular ratio = 1.36–1.42, and cover about ¾ of the ocular lobe; ocular lobe ratio = 0.71–0.82. The pronotal hind angles are obtuse, with the lateral margins divergent anteriorly from the angles. The pronotum is broad, MPW/PL = 1.31–1.33, with a moderately broad base, MPW/BPW = 1.40–1.52. The pronotal anterior transverse impression is deep, narrow, and crossed by deep wrinkles. The elytra are subquadrate with broad subangulate humeri, MEW/HuW = 1.78–1.95. Body coloration is quite uniform, with head and elytral disc rufobrunneous, and pronotal disc darker, rufopiceous. The legs are contrastedly paler, with metafemora rufoflavous with a piceous cloud over basal 1/3 of anterior surface, and the tibiae rufoflavous with a piceous cast, especially apically.

**Male genitalia** (n = 1). Aedeagal median lobe broad, robust, distance from parameral articulation to tip 3.2× depth at midlength (Fig. 22C); apex broadly extended beyond ostial opening, the apical face flat; median lobe curved to the right, tip appearing flat in ventral view (Fig. 22D); internal sac covered with fine microspicules, brown, round flagellar plate evident inside midlength of lobe shaft (Fig. 22C–D).

**Female reproductive tract** (n = 1). Bursa copulatrix broadly rounded, length 0.39 mm, breadth 0.34 mm (Fig. 23A); bursal walls thin, diaphanous; gonocoxite 1 with 3 apical fringe setae, the middle seta of series larger, 5 smaller setae on medial surface (Fig. 18E); gonocoxite 2 subtriangular, apex tightly rounded, base evenly extended from lateral margin, 2 lateral ensiform setae, the apical seta broader and longer, apical nematiform setae on medial surface at 0.75× gonocoxite length.

##### Lectotype.

Female (BMNH) hereby designated, labeled: mounting platen with Blackburn Maui code (Zimmerman 1957: 210), C. obscur. (on reverse) // Type // Hawaiian Is. Rev. T. Blackburn 1888-30 // LECTOTYPE Cyclothorax obscuricolor Blackburn J.K. Liebherr 1998 (black-margined red label).

##### Distribution and habitat.

*Mecyclothorax obscuricolor* was collected by R.C.L. Perkins in four different collecting lots (Nos. 112, 371, 372, and 622) that were derived from elevations ranging 1200–1980 m (Anonymous N D). Based upon the elevations and Perkin’s (1894) field notes, these localities spanned Olinda at lower elevations and Ukulele Camp and environs for the upper elevation localities (Fig. 21). No specific microhabitat may be ascribed to this species based on Perkins’ report (Perkins 1896c), and the species has not been recollected since 1896.

#### (012) Mecyclothorax obscuricornis

Taxon classificationAnimaliaColeopteraCarabidae

Sharp

**Figure 24.**
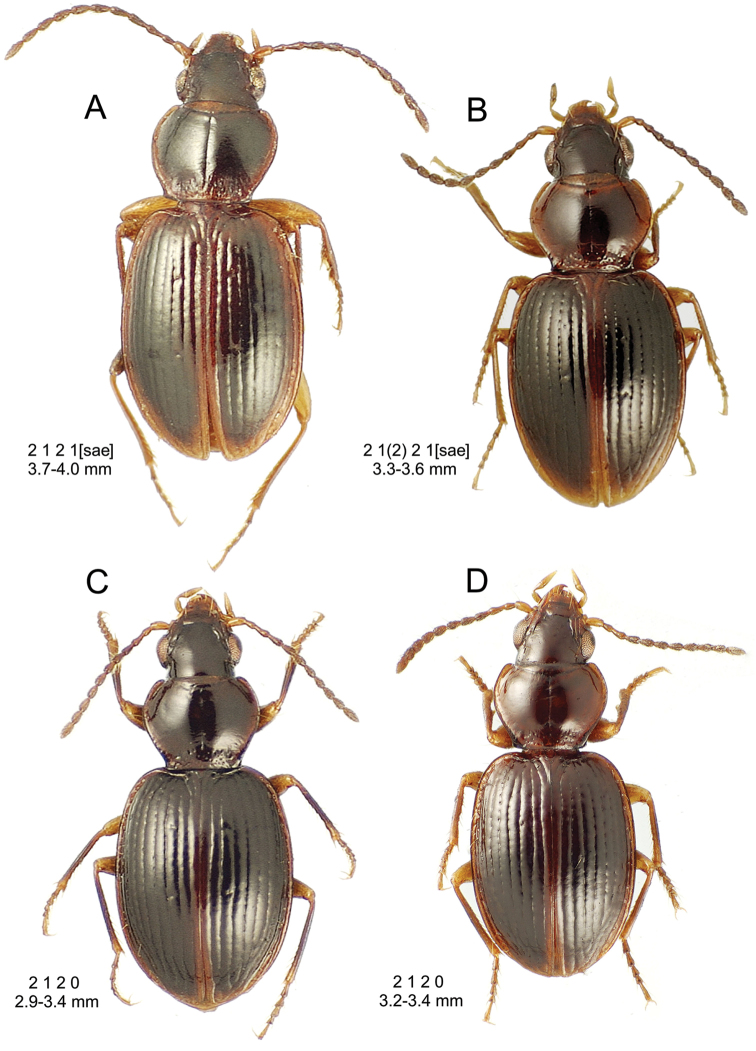
*Mecyclothorax obscuricornis* group species, dorsal habitus view. **A** *Mecyclothorax obscuricornis* (Ukulele Camp, 1525 m) **B** *Mecyclothorax waikamoi* (Waikamoi, 1265 m) **C** *Mecyclothorax poouli* (Kuhiwa, 1590 m) **D** *Mecyclothorax ahulili* (Pu‘u Ahulili, 1600 m).

**Figure 25.**
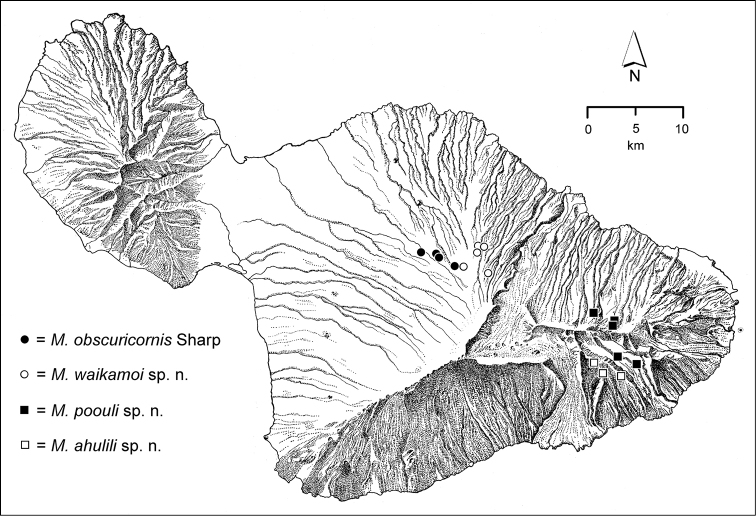
Recorded geographic distributions of *Mecyclothorax obscuricornis* group species.

Figs 18F , 22E–F , 23B , 24A , 25

Mecyclothorax obscuricornis Sharp 1903: 245; Britton 1948b: 160.

##### Diagnosis.

The combination of standardized body length 3.4–3.6 mm, distinctly punctate discal elytral striae (Fig. 24A), convex lateral elytral margins, and narrowly paler pronotal and elytral margins serves to diagnose this species from others in the group. This species shares with *Mecyclothorax obscuricolor* well-developed isodiametric and transverse-mesh microsculpture on the vertex, however the pronotal disc has more transverse sculpticells–breadth 3× length to unconnected transverse lines–and the elytral disc has only a shallow transverse mesh, sculpticell breadth 2× length, with the surface glossy. In common with *Mecyclothorax montanus*, the parascutellar seta is lacking. Setal formula 2 1(2) 2 1[sae]; the species is scored for rare occurrence of both lateral and basal pronotal setae based on one individual with unilateral presence of the basal seta.

##### Identification

(n = 3). The eyes are little convex, ocular ratio = 1.37–1.40, and small, ocular lobe ratio = 0.74–0.79. The pronotum is moderately constricted basally, MPW/BPW = 1.43–1.46, with the lateral margins subparallel for only a short distance anterad the obtuse hind angles. The pronotal median base is moderately depressed and rugose due to the presence of large punctures and longitudinal wrinkles. The elytra are subquadrate with tightly rounded to subangulate humeral angles; MEW/HuW = 1.82–1.89.

**Male genitalia** (n = 2). Aedeagal median lobe (Fig. 22E) more gracile than that of *Mecyclothorax obscuricolor* (Fig. 22C), distance from parameral articulation to tip 4× medial breadth, apex narrow, parallel sided, extended 2.7× its breadth beyond ostial opening, the tip rounded; median lobe nearly straight in ventral view, right margin slightly concave before rounded tip (Fig. 22F); internal sac covered with evident microspicules, flagellar plate elongate, visible just inside dorsal margin in lateral view (Fig. 22E).

**Female reproductive tract** (n = 1). Bursa copulatrix columnar with rounded apex, length 0.83 mm, breadth 0.34 mm, base as broad as vagina (Fig. 23D); bursal walls translucent, thinly wrinkled; gonocoxite 1 with 2–3 apical fringe setae, 7–8 small setae on medial surface (Fig. 18F); gonocoxite 2 subfalcate, apex subacuminate, base extended laterally, 2 lateral ensiform setae, the apical seta broader and longer, apical nematiform setae on medial surface at 0.68× gonocoxite length.

##### Lectotype.

Male (BMNH) hereby designated, labeled: Mecyclothorax obscuricornis Type D.S. Haleakala Perkins 120 (on mounting platen) // Type // Hawaiian Is. Perkins 1904-336 // LECTOTYPE Mecyclothorax obscuricornis Sharp J.K. Liebherr 1998 (black-margined red label).

##### Distribution and habitat.

Just as with *Mecyclothorax obscuricolor* above, *Mecyclothorax obscuricornis* was collected repeatedly by Perkins (Nos. 112, 113, 120, 251, 371, 372, 59 8, 600, 608) with his notes (Perkins 1894, 1896a, 1896b) placing those collecting activities in the vicinities of Olinda and Ukulele Camp (Fig. 25). Also, as with *Mecyclothorax obscuricolor*, this species has not been seen in nature Perkins collected it. In 1894 he collected both species on 31–iii, 1– iv, and 6–iv, supporting their occupation of similar or at least adjacent habitats during the late 19^th^ Century.

#### (013) Mecyclothorax waikamoi sp. n.

Taxon classificationAnimaliaColeopteraCarabidae

http://zoobank.org/2317B4D5-EE29-4F10-9798-F4EB735454F2

**Figure 26.**
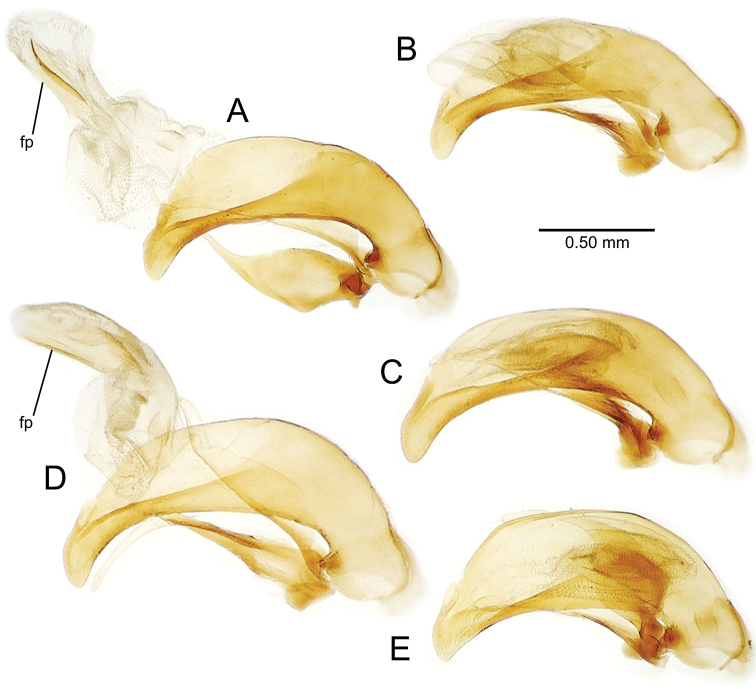
Male aedeagus, *Mecyclothorax obscuricornis* group species (for abbreviations see Table 2, p. 23). **A** *Mecyclothorax waikamoi*, right view, sac everted (Waikamoi, 1305 m). **B–D** *Mecyclothorax poouli*, right view **B** (Kīpahulu, 910 m) **C** (Kuhiwa, 1590 m) **D** Sac everted (Helele‘ike‘oha, 1800 m) **E** *Mecyclothorax ahulili*, right view (Pu‘u Ahulili, 1600 m).

**Figure 27.**
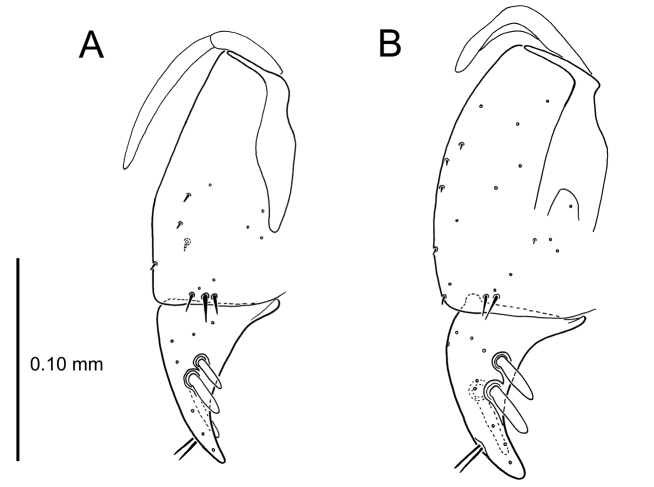
Left female gonocoxa, *Mecyclothorax obscuricornis* group species, ventral view. **A** *Mecyclothorax waikamoi* (Waikamoi, 1265 m) **B** *Mecyclothorax poouli* (Kuhiwa, 1590 m).

Figs 23C , 24B , 25 , 26A , 27A

##### Diagnosis.

This is the first of three very similar species, all characterized by small body size—standardized body length in this species = 2.9–3.4 mm—and subovoid elytra with variously punctate discal striae (Fig. 24B–D). The three species are all of dark coloration, with rufobrunneous head capsules, rufopiceous pronotal discs, and piceous elytral discs with narrowly paler, rufoflavous to flavous margins. *Mecyclothorax waikamoi* deviates from the other two in the well-developed punctation of elytral striae 1–6 in the basal half of the elytra (Fig. 24B). The eyes also tend to be less convex in this species—ocular ratio = 1.38–1.47—though that span overlaps the range of ocular ratios of the other two species at 1.43–1.51. The male aedeagus (Fig. 26A) can diagnose the species, with males of this species (Fig. 26A) exhibiting a broader, more apically flattened apex to the median lobe. Setal formula 2 1 2 0.

##### Description

(n = 5). *Head capsule* with frontal grooves broad near clypeus, a lateral carina to anterior supraorbital seta; dorsal impression of neck flat to slightly concave; labral anterior margin broadly, shallowly emarginate; antennae submoniliform, antennomere 2 sparsely setose, antennomere 3 with well-developed pelage of short setae; mentum tooth with sides acute, apex rounded. *Pronotum* broad, MPW/PL = 1.28–1.34, basally constricted, MPW/BPW = 1.47–1.54, with sinuate lateral margins anterad slightly obtuse hind angles (obtuse due to rounded basal margin inside angle); median base moderately depressed, sparsely punctate, shallow wrinkles at juncture with disc; basal margin broadly, slightly convex between laterobasal depressions; median longitudinal impression extremely shallow, narrowly incised; anterior transverse impression shallow, narrow, discontinuous medially; anterior callosity slightly convex, smooth; front angles slightly projected, rounded; distance between front and hind angles subequal, APW/BPW = 0.97–1.05; lateral marginal depression narrow, edge not upraised from front angle to lateral seta, very narrow and beadlike in basal half; laterobasal depression smooth, narrow, continuous with lateral depression. *Proepisternum* with ~5 minute punctures along hind marginal groove; prosternal process medially depressed, sides broadly upraised. *Elytra* subovoid and convex, sides and apex depressed relative to disc; basal groove evenly recurved to tightly rounded humeral angle; MEW/HuW = 1.95–2.03; parascutellar seta present; parascutellar striole with 3 punctures, discontinuous between punctures; sutural interval more convex than lateral intervals, sutural juncture elevated; sutural and 2^nd^ striae of subequal depth and punctation from base to apex; striae 1–6 and 8 complete, stria 7 shallower, associated intervals convex; 7^th^ and 8^th^ interval of similar convexity mesad subapical sinuation; 2 dorsal elytral setae at 0.33× and 0.50× elytral length, setal impression small, spanning about half of interval 3; both apical and subapical setae absent; lateral elytral setae arranged as anterior series of 7(6) setae, and a posterior series of 5(4) setae; elytral marginal depression slightly broader at humerus, narrowed laterally to a beadlike margin at subapical sinuation; subapical sinuation very shallow, more abruptly incurved anteriorly. *Metepisternum* with ~5 very shallow punctures in 1 row; metepisternal width to length ratio = 0.84; metepisternum/metepimeron suture distinct. *Abdomen* with irregular lateral wrinkles on ventrites 1–5; suture between ventrites 2 and 3 complete; apical male ventrite with 2 marginal setae, female apical ventrite with 4 equally spaced marginal setae and a median trapezoid of 4–6 short setae. *Legs*-metatarsomere 1/metatibial length ratio = 0.18; metatarsomere 4 length along outer lobe 1.33× medial tarsomere length, apical and subapical setae present; metatarsal dorsolateral sulci narrow, lateral, median area broad. *Microsculpture* of vertex an obsolete transverse mesh, sculpticell breadth 2–3× length; pronotal disc with obsolete transverse mesh, sculpticell breadth 3× length, surface glossy; pronotal median base glossy with obsolete transverse sculpticells; elytral disc with shallow transverse mesh, sculpticell breadth 2–3× length, transverse lines present on lateral reaches of elytra; elytral apex with transverse sculpticells, breadth 2–3× length; metasternum with distinct transverse mesh; laterobasal abdominal ventrites with swirling isodiametric and transverse microsculpture. *Coloration* of vertex glossy rufobrunneous to rufopiceous; antennomeres 1–3 flavous, 4–11 rufobrunneous; pronotal disc rufobrunneous, lateral margins and base slightly paler, rufous; proepipleuron flavous, proepisternum rufoflavous to rufobrunneous; elytral disc rufopiceous on intervals 2–9, sutural interval rufoflavous from base, flavous apically; elytral marginal depression narrowly flavous, apex and intervals 8–9 near apex flavous; elytral epipleuron flavous dorsally, rufoflavous along ventral margin, metepisternum rufobrunneous; abdomen with ventrites 1–3 medially rufopiceous, ventrites 4–6 rufobrunneous, apical 1/3 of apical ventrite 6 flavous; metafemur flavous, basal half with brunneous to piceous cloud on anterior surface; metatibia rufoflavous with brunneous cast.

**Male genitalia** (n = 3). Aedeagal median lobe distinctly curved dorsally, robust, distance from parameral articulation to tip 2.7× maximum breadth dorsad ostial opening (Fig. 26A); dorsal surface expanded at midpoint of ostial opening, apex downturned with apical face flat; internal sac unornamented, lightly spiculated, flagellar plate moderately elongate, length of sclerotized ventral face 0.43× distance from parameral articulation to apex.

**Female reproductive tract** (n = 1). Bursa copulatrix a very short, broad pouch, length 0.17 mm, breadth at base 0.25 mm (Fig. 23C); bursal walls thin, transparent, not wrinkled; gonocoxite 1 with 3 apical fringe setae (Fig. 27A), 4 smaller setae on medial surface; gonocoxite 2 subtriangular, apex pointed, base little extended laterally, 2 short lateral ensiform setae, the apical seta distinctly broader, apical nematiform setae on medial surface at 0.70× gonocoxite length.

##### Holotype.

Male (BPBM) dissected, platen mounted and labeled: ♂ (glued to mounting platen) // Waikamoi, Maui / 4000', VII-1956 // R. Namba / Collector // Mecyclothorax / waikamoi / ♂ #5 / det. J.K. Liebherr 2014 // HOLOTYPE / Mecyclothorax / waikamoi / Liebherr / det. J.K. Liebherr 2015 (black-margined red label).

##### Allotype.

Female (CUIC) labeled: HI:Maui Haleakala / Waikamoi N.C.P. Ukulele / Pipeline 7-V-1998 lot05 / 1550 m el. pyrethrum fog / mossy ohia J.K. Liebherr // 4 // Mecyclothorax / waikamoi / ♀ photo // ALLOTYPE / (same labeling as Holotype).

##### Paratypes.

HI: Maui: Koolau For. Res., Kula Pipeline Rd., pyrethrin fog log, 1305 m el., 18-v-2003 lot 09, Polhemus (NMNH, 3), pyrethrin fog *Metrosideros*, 1265 m el., 18-v-2003 lot 08, Polhemus (NMNH, 2); Waikamoi N.C.P., Honomanu drainage transect 3, sift litter, 1700 m el., 10-iv-1991 lot 01, Liebherr (CUIC, 1) scraping *Metrosideros* humus/moss, 1700 m el., 08-v-1991 lot 03, Liebherr (CUIC, 1).

##### Etymology.

The species epithet waikamoi is taken from the Hawaiian Waikamoi, the name of the Maui land section and stream that is translated from Hawaiian as “water of the ruler’s taro (Pukui et al. 1974).”

##### Distribution and habitat.

*Mecyclothorax waikamoi* is a species of ‘Ōhi‘a Montane Mesic to Wet Forest from 1200–1700 m elevation in the Waikamoi and Honomanu drainages (Fig. 25). It has been found in ground litter and in mossy humus on the trunks, and in the crotches of ‘ōhi‘a trees.

#### (014) Mecyclothorax poouli sp. n.

Taxon classificationAnimaliaColeopteraCarabidae

http://zoobank.org/F8166A63-6463-48DF-80A9-7099874B7A9A

Figs 23D , 24C , 25 , 26B–D , 27B

##### Diagnosis.

Distinguished from *Mecyclothorax waikamoi*, above, by the less punctate elytral striae (Fig. 24B, C) and the slightly larger, more convex eyes; ocular ratio = 1.43–1.48, ocular lobe ratio = 0.79–0.86. Distinguishable from *Mecyclothorax ahulili* by the subparallel pronotal lateral margins anterad the obtuse to nearly right hind angles (Fig. 24C), versus the divergent lateral margins and obtuse rounded hind angles of *Mecyclothorax ahulili* (Fig. 24D). The male aedeagal median lobe apex of this species (Fig. 26B–D) differs distinctively from the very short and broad apex characterizing *Mecyclothorax ahulili* (Fig. 26E), and the flagellar plate is larger than observed in *Mecyclothorax waikamoi* (Fig. 26A). Setal formula 2 1 2 0. Standardized body length 3.2–3.4 mm.

##### Description

(n = 5). [The description of *Mecyclothorax waikamoi* serves equally well for this species with the following substitutions.] Eyes moderately developed, ocular ratio = 1.43–1.48, ocular lobe ratio 0.79–0.86; pronotum variably broad, MPW/PL = 1.30–1.37; pronotal hind angles obtuse to nearly right, margin rounded behind; pronotal lateral margins subparallel for short distance anterad hind angles, MPW/BPW = 1.49–1.53; elytra may be narrower across the humeri in some individuals, MEW/HuW = 1.97–2.08; elytral sutural stria with minute irregularities in basal half but without distinct punctures, smooth and deep apically; parascutellar striole with 3 punctures, striole continuous between punctures; lateral elytral setal series arranged as 6-7 setae in anterior series, 5(4) in posterior series; only 4 setae observed in the median trapezoidal setal patch of the female apical abdominal ventrite; metafemora with brunneous cloud on basal anterior surface.

**Male genitalia** (n = 4). Aedeagal median lobe distinctly curved dorsally, robust, distance from parameral articulation to tip 2.7–3.0× maximum breadth dorsad ostial opening (Figs 26B–D); dorsal surface evenly curved at midpoint of ostial opening, apex downturned with apical face convex to briefly flattened, the flat surface shorter than in *Mecyclothorax waikamoi* (Fig. 26A); internal sac unornamented, lightly spiculated, flagellar plate large (Fig. 26D), length of sclerotized ventral face 0.55× distance from parameral articulation to apex.

**Female reproductive tract** (n = 1). Bursa copulatrix a triangular tentlike pouch, length 0.32 mm, basal breadth at vagina 0.33 mm (Fig. 23D); bursal walls thin, transparent; gonocoxite 1 with 2 apical fringe setae, 5–6 small setae on medial surface (Fig. 27B); gonocoxite 2 subtriangular, apex rounded, 2 lateral ensiform setae with apical seta broader and longer, apical nematiform setae on medial surface at 0.72× gonocoxite length.

##### Holotype.

Male (CUIC) labeled: HI:Maui Haleakala / Hanawi N.A.R. Poouli / Cabin 5-V-1998 lot02 / 1590m el. pyr. fog ohia / + Cibotium J.K. Liebherr // Mecyclothorax / poouli / ♂ photo / det. J.K. Liebherr 2014 // HOLOTYPE / Mecyclothorax / poouli / Liebherr / det. J.K. Liebherr 2015 (black-margined red label).

##### Paratypes.

HI: Maui: Haleakala N.P., Kipahulu Vy., Central Pali Tr., sifting leaf/moss litter, 915 m el., 30-iv-1991 lot 03, Liebherr/Medeiros (CUIC, 2), 1200 m el., 29-iv-1991 lot 03, Liebherr/Medeiros (CUIC, 2); Hana For. Res., Heleleikeoha Str. State Fence Camp, pyrethrin fog *Metrosideros*/moss, 1615 m el., 11-v-1998 lot 06, Polhemus (NMNH, 1), 12-v-1998 lot 04, Liebherr (CUIC, 1), 1795 m el., 12-v-1998 lot 11, Polhemus (NMNH, 2); Koolau For. Res., Hanawi N.A.R., Kuhiwa Vy. E rim, pyrethrin fog *Cibotium*, 915 m el., 10-vi-1999 lot 04, Polhemus (NMNH, 2), pyrethrin fog *Metrosideros*, 880 m el., 09-vi-1999 lot 09, Polhemus (NMNH, 1), Kuhiwa Vy., Poouli Cabin, pyrethrin fog *Metrosideros*/*Cibotium*, 1590 m el., 05-v-1998 lot 02, Liebherr (CUIC, 7), pyrethrin fog *Metrosideros*/moss, 1590 m el., 05-v-1998 lot 01, Liebherr (CUIC, 2), lot 03, Polhemus (NMNH, 4).

##### Etymology.

The po‘o uli (*Melamprosops phaeosoma* Casey and Jacoby 1974) was a member of the Hawaiian drepanid finches first discovered in 1973 in the Hanawī rainforest. By 2004 it was extinct (Powell 2008). The Hawaiian species epithet is meant as a memorial to the birds that may have fed on the ancestors of the type series of *Mecyclothorax poouli*.

##### Distribution and habitat.

*Mecyclothorax poouli* occupies ‘Ōhi‘a-Hāpu‘u (*Metrosideros*-*Cibotium*) Wet Forest in the Hāna Bogs, and Kuhiwa and Kīpahulu Valleys (Fig. 25). Known localities span 900–1800 m elevation. The beetles have been discovered in moss and leaf litter at ground level, and in mossy epiphytic growths on ‘ōhi‘a trunks and downed nurse logs.

#### (015) Mecyclothorax ahulili sp. n.

Taxon classificationAnimaliaColeopteraCarabidae

http://zoobank.org/28050B2D-A9DF-4E3E-99B1-0193A8900D0C

Figs 24D , 25 , 26E

##### Diagnosis.

This species exhibits strial punctation intermediate to *Mecyclothorax waikamoi* and *Mecyclothorax poouli* (Fig. 24B–D)—the sutural stria bears minute punctulae associated with irregularities in the strial orientation—plus the briefest sinuation of the pronotal lateral margin anterad the hind angle. The pronotal lateral marginal depression is also somewhat broader, especially at the front angle where it is almost explanate. The male aedeagal median lobe exhibited by males of this species is very different from those present in males of the other two species, with the apex very brief and ventrally subangulate (Fig. 26E). The median lobe is also shorter and more robust overall than those seen in males of the other two species. Setal formula 2 1 2 0. Standardized body length 2.9–3.2 mm.

##### Description

(n = 4). [As for *Mecyclothorax poouli* above, the description of *Mecyclothorax waikamoi* serves for *Mecyclothorax ahulili* with the following substitutions.] Eyes larger, more convex, ocular ratio = 1.46–1.51, ocular lobe ratio = 0.83–0.85; pronotum variably broad, MPW/PL = 1.27–1.35, basally constricted, MPW/BPW = 1.49–1.56; elytra slightly broader across humeri, MEW/HuW = 1.98–2.04; 5 setae observed in the median trapezoidal setal patch of the female apical abdominal ventrite; metafemora with brunneous cloud on basal anterior surface.

**Male genitalia** (n = 1). Aedeagal median lobe very broad, robust, distance from parameral articulation to tip 2.2× depth at midlength (Fig. 26E); apex very briefly extended beyond ostial opening, length of extension subequal to breadth, apical face slightly flattened; internal sac lightly spiculated, flagellar plate visible in uneverted specimen, plate length 0.49× distance from parameral articulation to tip.

##### Holotype.

Male (CUIC) dissected and labeled: HI: Maui Haleakala N.P. / Kekuewa Hill 0.7 km N / Puu Ahulili sift moss & / humus 16-V-1993 lot 02 / el. 1600 m // J.K. Liebherr & / A.C. Medeiros / collectors // 3 // Mecyclothorax / ahulili / ♂ #7 / det. J.K. Liebherr 2014 // HOLOTYPE / Mecyclothorax / ahulili / Liebherr / det. J.K. Liebherr 2015 (black-margined red label).

##### Paratypes.

HI: Maui, Haleakala N.P., Kipahulu west rim ESE Kuiki, sifting *Metrosideros* litter, 1830 m el., 12-v-1998 lot 08, Liebherr/Medeiros (CUIC, 1), Kekuewa Hill 0.7 km N Puu Ahulili, sifting humus/moss, 1600 m el., 16-v-1998 lot 02 (CUIC, 1), lot 03 (CUIC, 1), Kaapahu, 1250 m el., 7-iv-2004 lot 01, Kaholoa‘a (BPBM, 1).

##### Etymology.

The Hawaiian species epithet ahulili is based on the peak Pu‘u Ahulili that is near the type locality for this species. ‘Ahulili means glowing or dazzling (Pukui et al. 1974), though being a Hawaiian word it is to be treated as a noun.

##### Distribution and habitat.

*Mecyclothorax ahulili* is known from three localities on the Manawainui Planeze (Fig. 25) that range 1250–1805 m elevation. All specimens have been found in ‘Ōhi‘a Montane Wet Forest within moss, leaf and humus litter at ground level, though the beetles should also be found on mossy trunks as for the prior two closely related species.

### *Mecyclothorax robustus* species group

**Diagnosis.** Species in this group are characterized by: 1, uniformly darker dorsal body coloration, often with a bronzed or purplish reflection, though pronotal and elytral margins may be somewhat paler, rufobrunneous to rufoflavous; 2, larger body size, standardized body length = 4.1–6.3 mm; 3, regular elytral striation with adjacent striae not approaching or anastomosing except approaching the elytral apex. Among Haleakalā species the setal formula is 2 2 2 2, with the exceptions of *Mecyclothorax consanguineus*, characterized by loss of the apical elytral seta and instability for bilateral presence of the basal pronotal setae, and *Mecyclothorax cognatus* with rare individuals lacking one of the lateral pronotal setae.

**Membership and distribution.** The 11 Haleakalā species are complemented by a lone species—*Mecyclothorax chalcosus* Sharp—from West Maui (Liebherr 2011), two species from Moloka‘i (Liebherr 2007), and four species from the Big Island of Hawai‘i (Liebherr 2008b).

#### Key to adults of the *Mecyclothorax robustus* species group, Haleakalā volcano, Maui, Hawai‘i

**Table:** 

1	Pronotum more cordate, constricted basally, MPW/BPW = 1.31–1.60 (Figs 28B–C, 33, 38)	**2**
1’	Pronotum more quadrate, not constricted basally, MPW/BPW = 1.17–1.29 (Fig. 28A)	(016) ***Mecyclothorax aeneipennis* Liebherr**
2(1)	Elytra short relative to breadth, orbicular, lateral margins convex from humerus to subapical sinuation (Figs 28B–C, 33A–B)	**3**
2’	Elytra longer, lateral margins straight, though perhaps divergent between humerus and subapical sinuation (Figs 33C–D, 38)	**6**
3(2)	Eyes more convex, ocular ratio = 1.39–1.51 (Figs 28C, 33, 38)	**4**
3’	Eyes less convex, ocular ratio = 1.35 (Fig. 28B)	(017) ***Mecyclothorax affinis* sp. n.**
4(3)	Body narrower overall or forebody narrow relative to orbicular elytra (Figs 33, 38), pronotal lateral margin narrowly upraised mesad basal sinuation, the surface evenly elevated from laterobasal depression to margin, lateral marginal depression narrowly upraised at midlength	**5**
4’	Body broader, more robust, pronotal lateral margin explanate at basal sinuation, moderately broad at midlength (Fig. 28C)	(018) ***Mecyclothorax cognatus* Sharp**
5(4)	Pronotum cordate with glabrous hind angles; elytra very narrow basally, ovoid, MEW/HuW = 1.76, elytral basal groove broadly rounded at humerus (Fig. 33A); discal elytral striae finely inscribed, associated intervals convex, bearing transverse-line microsculpture not arranged in a distinct mesh	(019) ***Mecyclothorax anchisteus* sp. n.**
5’	Pronotum broader basally, quadrisetose; elytra moderately narrow basally, subquadrate, MEW/HuW = 1.91–2.04, elytral basal groove angled anteriorly at humerus resulting in hitched humeral angle (Fig. 33B); discal elytral striae more broadly impressed, associated intervals only moderately convex, covered with well-defined transverse-mesh microsculpture, sculpticell breadth 3–4× length	(020) ***Mecyclothorax consanguineus* sp. n.**
6(2)	Discal elytral striae 1-6 smooth to indistinctly punctate in basal half, if punctulae are present at the deepest portions of striae, they are elongate and do not expand strial breadth (Fig. 33C–D)	**7**
6’	Discal elytral striae 1–6 distinctly punctate in basal half (Fig. 38)	**8**
7(6)	Eyes less convex, ocular ratio = 1.42 (Fig. 33C); pronotal disc covered with granulate isodiametric microsculpture	(021) ***Mecyclothorax aeneus* Sharp**
7’	Eyes more convex, ocular ratio = 1.46–1.50 (Fig. 33D); pronotal disc covered with transverse mesh microsculpture, sculpticell breadth 2–3× length	(022) ***Mecyclothorax antaeus* sp. n.**
8(6)	Body size smaller, standardized body length 4.1–4.7 mm	**9**
8’	Body size larger, standardized body length 4.8–6.2 mm	**10**
9(8)	Elytra with isodiametric microsculpture on sutural interval near base, a mixture of distinct isodiametric to transverse-mesh sculpticells basally on intervals 2–6, sculpticell breadth up to 4× length	(023) ***Mecyclothorax cymindicus* Sharp**
9’	Elytra with transverse-mesh microsculpture on sutural interval near base, transverse lines without formation of mesh on lateral intervals	(024) ***Mecyclothorax cymindulus* sp. n.**
10(8)	Discal elytral striae 2–4 distinctly punctate throughout length, or at least to position behind posterior dorsal seta (Fig. 38C–D); eyes larger, more convex, ocular ratio = 1.48–1.54, ocular lobe ratio = 0.76–0.84; male aedeagal median lobe apex dorsally expanded distad ostium (Fig. 41); internal sac ventrally covered with field of elongate, brunneous microsetae, a field of larger spicules may be present (Fig. 41B, E, G)	(025) ***Mecyclothorax robustus* (Blackburn)**
10’	Dorsal elytral striae 2–4 distinctly punctate only in basal portion before anterior dorsal seta, the punctures associated with change in orientation of stria in some instances, or with expansion of strial breadth in others (Fig. 38E); eyes smaller, less convex, ocular ratio = 1.41–1.49, ocular lobe ratio = 0.70–0.78; male aedeagal median lobe apex parallel sided distad ostium, evenly downturned to tightly rounded apex (Fig. 43), internal sac with ventral surface bearing very short, pale microsetae and a small field of larger spicules (Fig. 43B, F, H)	(026) ***Mecyclothorax haydeni* sp. n.**

#### (016) Mecyclothorax aeneipennis

Taxon classificationAnimaliaColeopteraCarabidae

Liebherr

**Figure 28.**
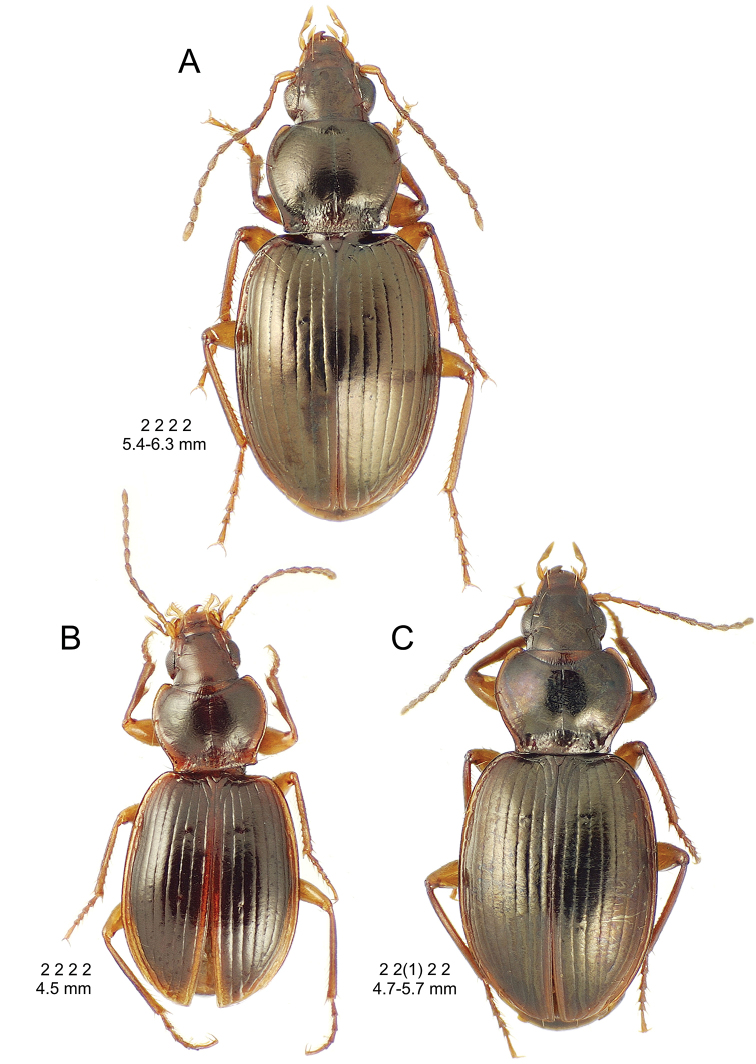
*Mecyclothorax robustus* group species, dorsal habitus view. **A** *Mecyclothorax aeneipennis* (Polipoli, 1775 m) **B** *Mecyclothorax affinis* (West Wailuanui, 1950 m) **C** *Mecyclothorax cognatus* (Ukulele Camp Pipeline, 1510 m).

**Figure 29.**
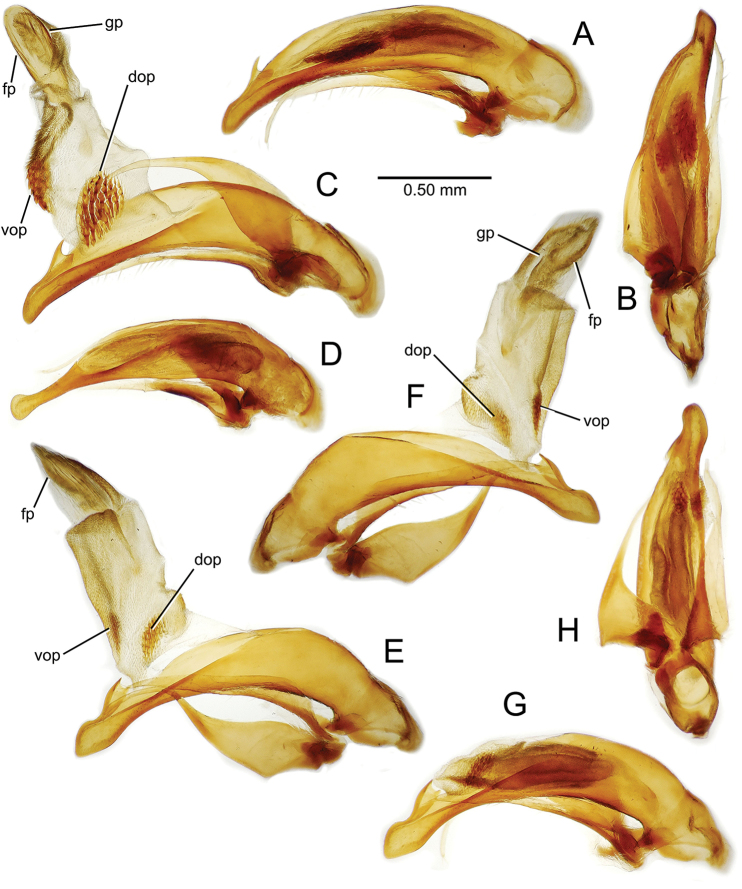
Male aedeagus, *Mecyclothorax robustus* group species (for abbreviations see Table 2, p. 23). **A–C** *Mecyclothorax aeneipennis* **A–B** Right and ventral views (Polipoli, 1878 m) **C** Right view, sac everted (Polipoli, 1776 m) **D** *Mecyclothorax affinis*, right view (West Wailuanui, 1950 m) **E–H** *Mecyclothorax cognatus* (Honomanu, 1850 m) **E** Right view, sac everted **F** Left view, sac everted **G** Right view, sac inverted **H** Ventral view.

**Figure 30.**
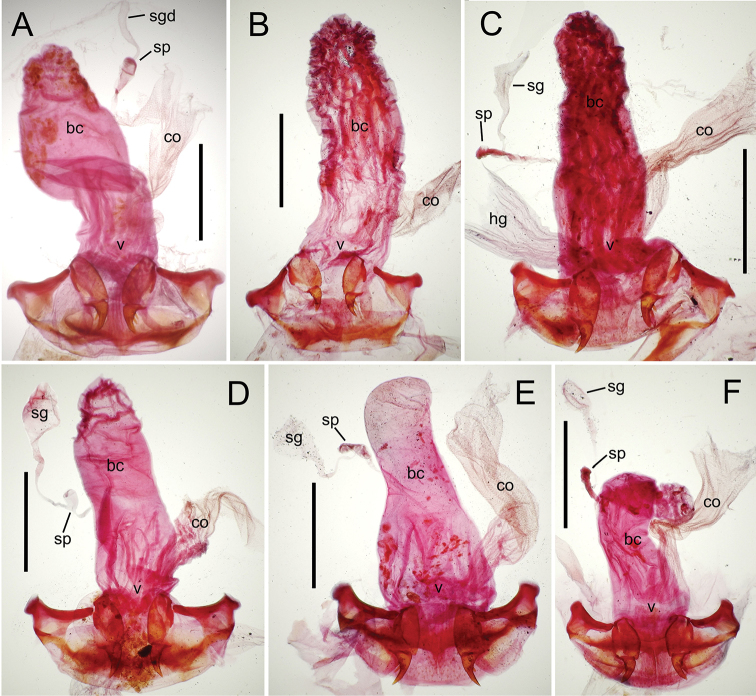
Female bursa copulatrix and associated reproductive structures, *Mecyclothorax robustus* group species, ventral view (for abbreviations see Table 2, p. 23). **A** *Mecyclothorax aeneipennis* (Polipoli, 1890 m) **B** *Mecyclothorax cognatus* (Ukulele Camp, 1525 m) **C** *Mecyclothorax consanguineus* (Honomanu, 1850 m) **D** *Mecyclothorax aeneus* (Honomanu, 1820–1850 m) **E** *Mecyclothorax antaeus* (ESE Kuiki, 1850 m) **F** *Mecyclothorax cymindicus* (Ukulele Camp Pipeline, 1495–1525 m). Scale bar = 0.50 mm.

**Figure 31.**
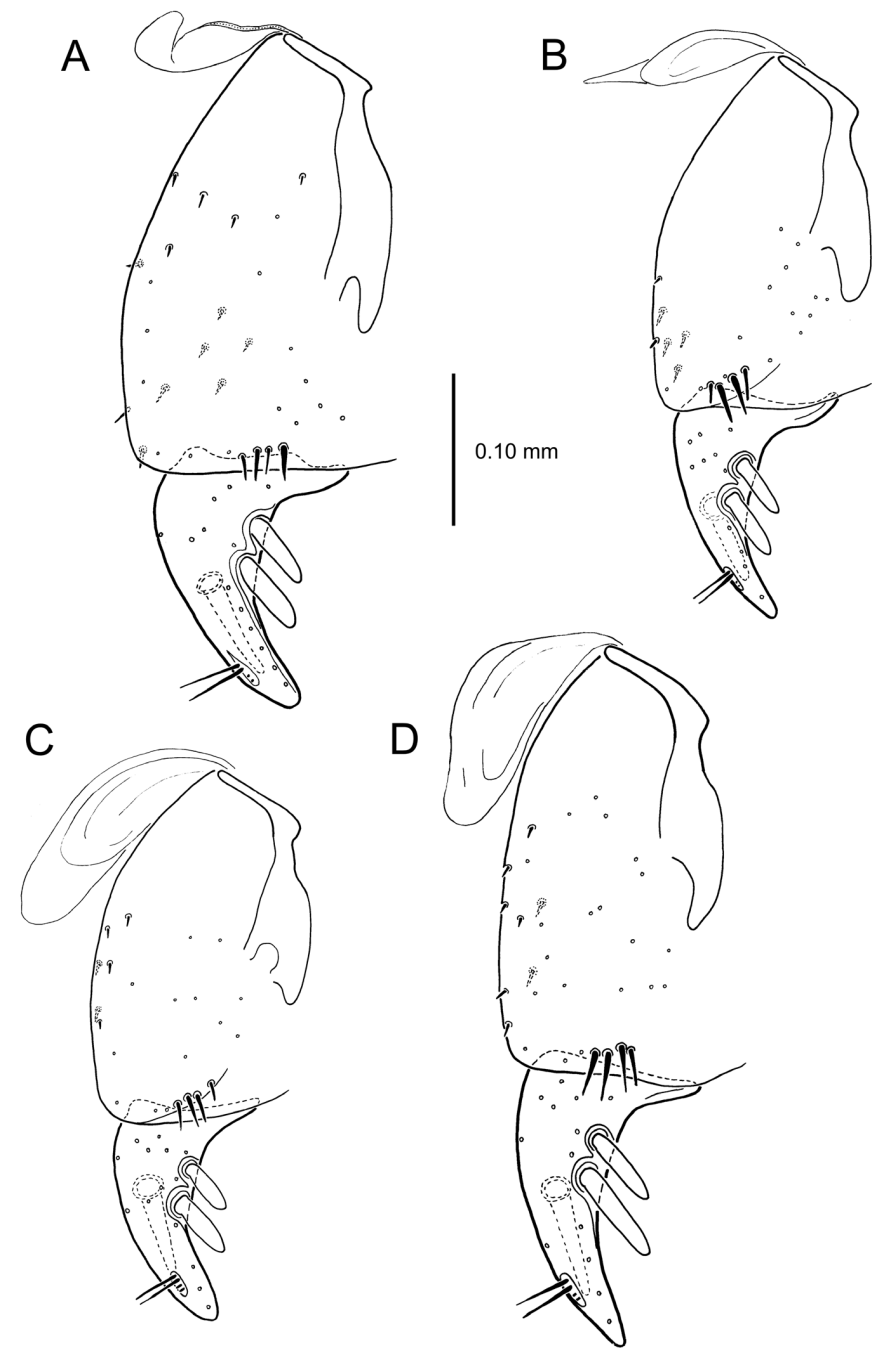
Left female gonocoxa, *Mecyclothorax robustus* group species, ventral view. **A** *Mecyclothorax aeneipennis* (Polipoli, 1890 m) **B** *Mecyclothorax cognatus* (Ukulele Camp, 1525 m) **C** *Mecyclothorax consanguineus* (Honomanu, 1850 m) **D** *Mecyclothorax aeneus* (Honomanu, 1820–1850 m).

**Figure 32.**
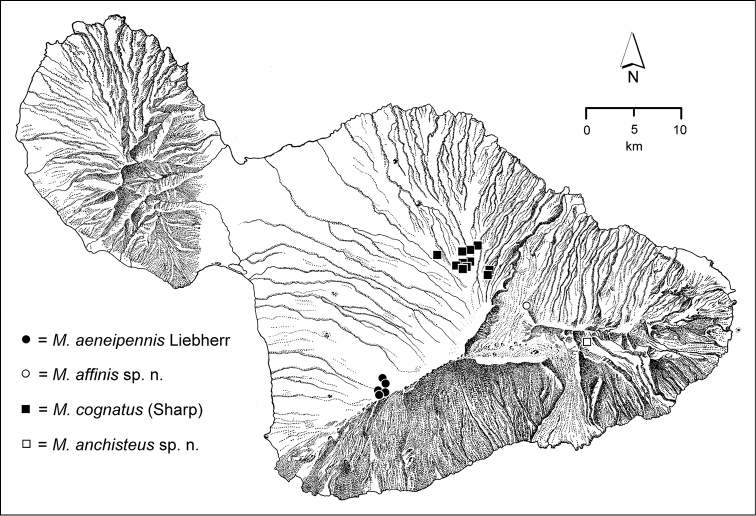
Recorded geographic distributions of *Mecyclothorax robustus* group species.

Figs 28A , 29A–C , 30A , 31A , 32

Mecyclothorax aeneipennis Liebherr 2005b: 123.

##### Diagnosis.

Among Haleakalā species of this group, *Mecyclothorax aeneipennis* exhibits the most quadrate pronotum, with the lateral margins little sinuate outside the laterobasal depressions (Fig. 28A); MPW/BPW = 1.17–1.29, versus MPW/BPW = 1.34–1.57 for all other Haleakalā species in this group. The elytral intervals are slightly convex, with discal striae 1–5 lined with small but distinct punctures in their basal halves to 2/3 of length. At the elytral apex, the 8^th^ interval is more convex than the fused apical portion of intervals 5 + 7. The vertex is rufobrunneous, elytral disc slightly darker rufopiceous, and elytral disc rufopiceous with a cupreous reflection. The legs are contrastedly paler; femora flavous and tibiae flavous with a brunneous cast. Setal formula 2 2 2 2. Standardized body length 5.4–6.3 mm.

##### Identification

(n = 5). The eyes are moderately convex, ocular ratio = 1.41–1.50, ocular lobe ratio = 0.73–0.81. The elytra are quadrate, with the basal groove evenly recurved to the tightly rounded to subangulate humerus, the lateral marginal depression broad with margin upraised behind the humeral angle; MEW/HuW = 1.68–1.78. The dorsal body surface bears well-developed microsculpture: 1, vertex and pronotal disc with transverse mesh, sculpticell breadth 2× length; 2; 2, pronotal median base with mixture of granulate isodiametric and transverse-mesh microsculpture; 3, elytral disc with distinct granulate isodiametric mesh; and 4, elytral apex with a transverse mesh, sculpticell breadth 3–4× length.

**Male genitalia** (n = 2). Aedeagal median lobe gracile, distance from parameral articulation 4.2× median breadth (Fig. 29A), apex narrowly extended beyond ostial opening, the tip flattened on dorsoapical aspect, tightly rounded ventrally; median lobe straight in ventral view, the right margin slightly concave before blunt tip, the left margin curved rightward to meet apical extension (Fig. 29B); internal sac with well developed, dorsal and ventral microtrichial patches, both composed of stout spicules (Fig. 29C); flagellar plate with internal face well sclerotized, length of plate 0.40× distance from parameral articulation to tip.

**Female reproductive tract** (n = 1). Bursa copulatrix columnar with expanded apex, length 1.2 mm, apical breadth 0.57 mm, basal breadth 0.40 mm (Fig. 30A); bursal base translucent with thick wrinkles, apex more transparent, little wrinkled; gonocoxite 1 with 4 apical fringe setae, a small seta unilaterally present at medial apex, otherwise 7–10 small setae on medial surface (Fig. 31A); gonocoxite 2 narrowly subtriangular with broad apex and tightly rounded tip, base broadly extended laterally, 2 lateral ensiform setae with apical seta broader and longer, apical nematiform setae on medioventral surface at 0.71× gonocoxite length.

##### Holotype.

Male (CUIC) designated by Liebherr (2005b). Type locality: HI: Maui, Haleakalā, Polipoli S.R.A., 1890 m el.

##### Distribution and habitat.

*Mecyclothorax aeneipennis* is restricted to the forests near Polipoli Springs on the southwest rift of Haleakalā (Fig. 32). At the time this area was surveyed it was extensively afforested with exotic gymnosperms, especially *Pinus radiata*. Many of these trees had lodged, creating tangles of old logs with loose bark. Beetles were found under loose bark of downed logs, under logs on the ground, or by grubbing the *Pinus* leaf litter; i.e., pushing the litter aside to expose an area of soil and waiting for beetles to run into the arenalike opening. *Mecyclothorax aeneipennis* individuals were also found in more native situations, such as in mossy litter, or among tangles of *Dryopteris* fern stems.

#### (017) Mecyclothorax affinis sp. n.

Taxon classificationAnimaliaColeopteraCarabidae

http://zoobank.org/12C6EBDE-82B9-4521-8ABE-E1FFE0B84C29

Figs 28B , 29D , 32

##### Diagnosis.

Like *Mecyclothorax aeneipennis* in the quadrate elytra with broad humeri—MEW/HuW = 1.76—but with cordate pronotum, the lateral margins slightly convergent anterad the projected, right hind angles (Fig. 28B). The eyes are relatively flat, ocular ratio = 1.35, and the ocular lobes project abruptly from the gena; a slotlike impression at the juncture of gena and ocular lobe. The single specimen, which does not appear teneral, has the pronotal and elytral lateral marginal depressions paler than the discs, the pronotal margins rufobrunneous versus the rufopiceous disc, and the elytral marginal depression narrowly rufoflavous versus the rufopiceous disc. Moreover, this is the only Haleakalā species in the group to be characterized by absence of the parascutellar seta. Setal formula 2 2 2 2. Standardized body length 4.5 mm.

##### Description

(n = 1). *Head capsule* with frontal grooves broad near clypeus, a lateral carina to anterior supraorbital seta, dorsal surface of neck slightly concave; labral anterior margin broadly, shallowly emarginate; antennae filiform, antennomeres 2–3 with sparse pelage of short setae; mentum tooth with sides acute, apex tightly rounded. *Pronotum* moderately transverse, MPW/PL = 1.30, base moderately constricted, MPW/BPW = 1.38; median base depressed relative to disc, surface strigose with fine longitudinal wrinkles; basal margin slightly convex between the laterobasal depressions; median longitudinal impression shallow, very finely incised at depth, joined by irregular transverse wrinkles; anterior transverse impression deep, finely incised at depth, separate from discal intervals; anterior callosity moderately convex, crossed by shallow wrinkles; front angles slightly projected, apex tightly rounded; APW/BPW = 0.99; lateral marginal depression slightly broader at front angle, moderately narrow behind, edge upturned; laterobasal depression smooth, laterally elevated to projected lateral margin. *Proepisternum* with smooth marginal groove; prosternal process medially depressed, upraised laterally to narrow bead anterad coxa. *Elytra* with convex disc, sides progressively sloped laterad; basal groove recurved medially, straight laterally to subangulate humerus; parascutellar striole shallow, with 3–4 punctures; sutural interval flat basally, convex at suture from disc to apex; sutural and 2^nd^ striae of subequal depth from base to apex; intervals 2–5 moderately convex, associated striae with minute punctulae causing strial irregularities, all striae smooth and deep apically; 7^th^ and 8^th^ intervals of similar convexity mesad subapical sinuation; 2 dorsal elytral setae at 0.25× and 0.61–0.63× elytral length, setal impressions extended over 2/3 of interval 3; both apical and subapical setae present; lateral elytral setae arranged as an anterior series of 7 setae and a posterior series of 6 setae; elytral marginal depression moderately broad throughout length until reduced to beadlike margin from subapical sinuation to apex; subapical sinuation shallow, more abruptly incurved anteriorly. *Mesepisternum* with ~8–9 punctures in 2–3 rows; metepisternal width to length ratio = 0.83. *Abdomen* with irregular lateral wrinkles on abdominal ventrites 1–5; suture between ventrites 2 and 3 complete; apical ventrite of male with 2 marginal setae. *Legs*-metatarsomere 1/metatibial length ratio = 0.20, metatarsomere 4 length along outer lobe 1.2× medial tarsomere length, apical and subapical setae present; metatarsal dorsolateral sulci very broad, median area rough to carinate. *Microsculpture* of vertex isodiametric to slightly transversely stretched in rows; pronotal disc with transverse mesh, sculpticell breadth 2–3× length; pronotal median base with isodiametric to transverse mesh; elytral disc with transverse mesh, sculpticell breadth 3× length, to transverse lines; elytral apex with shiny transverse mesh, sculpticell breadth 2–3× length; metasternum with distinct transverse mesh; laterobasal abdominal ventrites with swirling isodiametric and transverse microsculpture. *Coloration* of vertex rufobrunneous; antennomere 1 flavous, antennomeres 2–3 rufoflavous, 4–11 rufobrunneous; pronotal disc rufopiceous; pronotal margins rufobrunneous in depression, lateral bead darker, rufopiceous; proepipleuron rufoflavous, proepisternum dark rufobrunneous; elytral disc rufopiceous; sutural interval basally rufobrunneous, apically rufoflavous to flavous; elytral margins and apex narrowly rufoflavous; elytral epipleuron rufoflavous laterally, rufobrunneous ventrally, metepisternum rufopiceous; abdomen medially rufopiceous, laterally rufobrunneous; abdominal apical ventrite with narrowly paler margin, rufobrunneous; metafemur flavous with piceous cloud on basal 2/3; metatibia rufobrunneous.

**Male genitalia** (n = 1). Aedeagal median lobe gracile, distance from parameral articulation to tip 4× median breadth (Fig. 29D), apex elongate, with narrow extension beyond ostial opening and dorsoventrally expanded, spoonlike tip; internal sac without apparent microtrichial patches, moderately elongate flagellar plate visible dorsad parameral articulation in uneverted specimen, flagellar plate length 0.42× distance from parameral articulation to tip.

##### Holotype.

Male (BPBM) dissected and labeled: HAWAIIAN ISLANDS / Maui, Haleakala / Waikau Cabin 6400', / VI-18-1975 // R. Burkhart / Collector // ACC. NO. 1990.009 / BISHOP Museum // HOLOTYPE / Mecyclothorax / affinis / Liebherr / det. J.K. Liebherr 2015 (black-margined red label).

##### Etymology.

The Latin adjective affinis—meaning related to or neighboring—is used for this species to signify its close relationship to *Mecyclothorax cognatus*, a species named with the Latin word that means kindred or related (Brown 1956).

##### Distribution and habitat.

The lone specimen representing this species was collected at Waikau Cabin (Fig. 32) without any associated ecological information.

#### (018) Mecyclothorax cognatus

Taxon classificationAnimaliaColeopteraCarabidae

Sharp

Figs 28C , 29E–H , 30B , 31B , 32

Mecyclothorax cognatus Sharp 1903: 255; Britton 1948b: 165.
Atelothorax optatus Sharp 1903: 269; Britton 1948b: 165 (synonymy).

##### Diagnosis.

**Figure 33.**
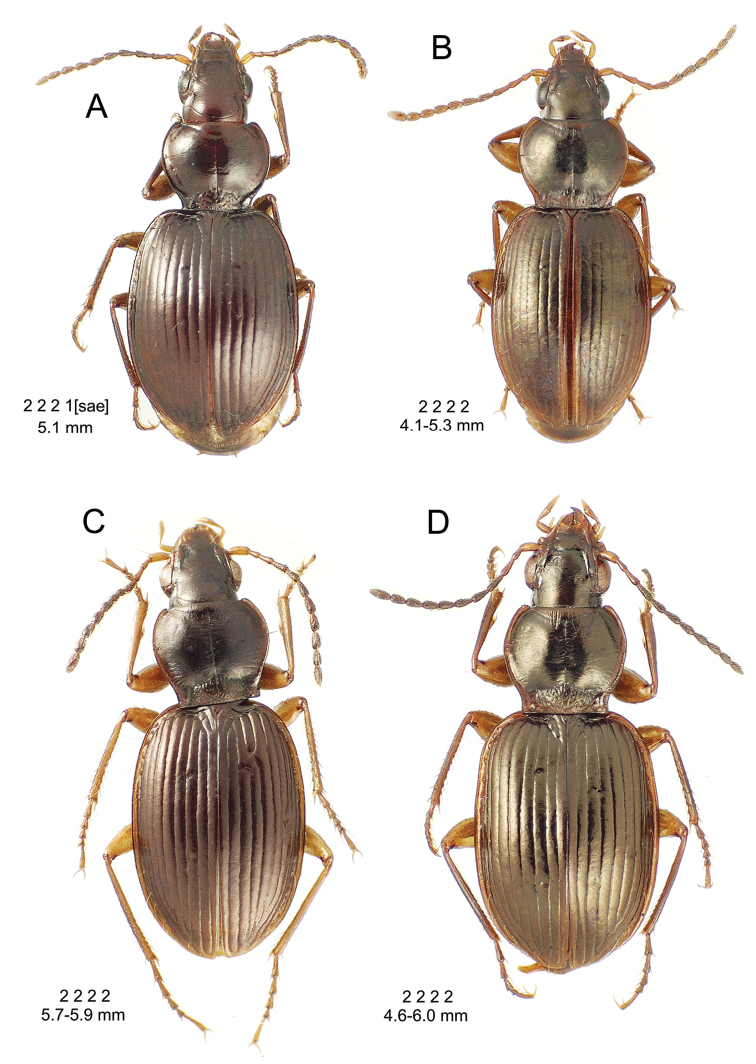
*Mecyclothorax robustus* group species, dorsal habitus view. **A** *Mecyclothorax anchisteus* (Kīpahulu, 1960 m) **B** *Mecyclothorax consanguineus* (Ukulele Camp Pipeline, 1510 m) **C** *Mecyclothorax aeneus* (Honomanu, 1820–1850 m) **D** *Mecyclothorax antaeus* (ESE Kuiki, 2145 m).

**Figure 34.**
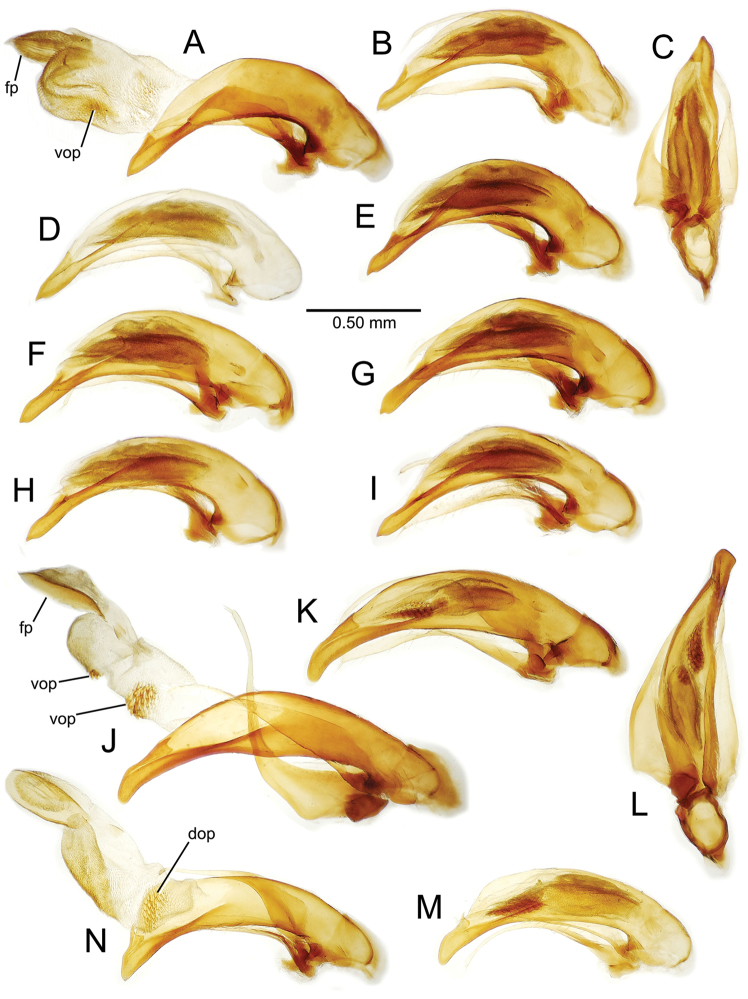
Male aedeagus, *Mecyclothorax robustus* group species (for abbreviations see Table 2, p. 23). **A–I** *Mecyclothorax consanguineus*. **A** Right view, sac everted (Ukulele Camp Pipeline, 1534 m) **B–C** Right and ventral views (Ukulele Camp Pipeline, 1534 m) **D** Right view (Honomanu, 1750 m). **E** Right view (ESE Kuiki, 2164 m) **F** Right view (ESE Kuiki, 2135 m) **G** Right view (Paliku, 1900 m) **H–I** Right view (Kīpahulu, 1960 m) **J–L** *Mecyclothorax antaeus* **J** Right view, sac everted (ESE Kuiki, 2145 m) **K–L** Right and left views (ESE Kuiki, 2105 m) **M** *Mecyclothorax cymindicus*, right view (near Ukulele Camp, 1210–1365 m) **N** *Mecyclothorax cymindulus*, right view, sac everted (Kīpahulu, 1960 m).

Among the mid-sized species in this group—standardized body length 4.7–5.7 mm—this species exhibits the broadest body in both pronotal and elytral dimensions. The pronotum is transverse, MPW/PL = 1.26–1.33, and basally broad, MPW/BPW = 1.31–1.42. This species is broadly sympatric in the Waikamoi area with *Mecyclothorax consanguineus*, the species most similar in appearance and thus likely to cause confusion. *Mecyclothorax cognatus* can be distinguished by the broad elytra (Fig. 28C), with broad humeri, MEW/HuW = 1.83–1.94 versus MEW/HuW = 1.91–2.04 for *Mecyclothorax consanguineus* (Fig. 33B). The pronotum of *Mecyclothorax cognatus* also exhibits more broadly explanate lateral margins just before the hind angles, that area broadly extended from the deep, smooth laterobasal depressions. *Mecyclothorax consanguineus* conversely exhibits less explanate lateral margins at that position. If a male is available, the aedeagal tip is absolutely diagnostic even if extended only slightly from the specimen. Those of *Mecyclothorax cognatus* males have an angulate apex with subangulate tip (Fig. 29E–G), whereas those of *Mecyclothorax consanguineus* males have an elongate apex with a mucronate tip (Fig. 34A–I). Sharp (1903) described *Atelothrus optatus* based his interpretation that the lateral pronotal setae were missing in the unique type specimen. The left lateral seta is indeed absent, but the articulatory socket for the right lateral seta is evident, resulting in the species setal formula being scored here as 2 2(1) 2 2.

##### Identification

(n = 5). The eyes are moderately developed—ocular ratio = 1.41–1.46, ocular lobe ratio = 0.75–0.81—with the ocular lobe smoothly joined to the gena. The pronotal front angles are broadly protruded, subangulate externally, with the anteriorly broad pronotal lateral marginal depression narrowed to the position of the lateral seta, and then broadened toward the back of the pronotum. The elytral striae are present across the width of the elytra, depth of striae 6 and 7 subequal to slightly shallower than striae 1–5 and 8. The discal elytral intervals are only slightly convex, with very fine punctures in the associated striae. The metepisternum bears ~16 punctures in 2–3 rows, about twice as many punctures as seen in *Mecyclothorax consanguineus*. Cuticular microsculpture is essentially identical to that observed in *Mecyclothorax consanguineus*: 1, vertex with transverse mesh, sculpticell breadth 2× length; 2, pronotal disc with transverse mesh, sculpticell breadth 2–3× length; 3, pronotal base with distinct isodiametric and transverse sculpticells; 4, elytral disc with transverse mesh, sculpticell breadth 3–4× length; and 5, elytral apex with transverse mesh, sculpticell breadth 3× length, to transverse lines.

**Male genitalia** (n = 6). Aedeagal median lobe gracile with broad, trapezoidal apex, distance from parameral articulation to tip 4.1× median breadth (Fig. 29G), apex with flat ventral and apical faces, the tip angulate; median lobe nearly straight in ventral view, the right margin concave, and left margin incurved before the apparently rounded tip (Fig. 29H); internal sac broad, parallel sided, with moderate dorsal microtrichial patch and smaller ventral microtrichical patch that is near base of sac (Fig. 29E–F); flagellar plate well sclerotized, length 0.42× distance from parameral articulation to tip.

**Female reproductive tract** (n = 1). Bursa copulatrix columnar with rounded apex, length 1.32 mm, breadth 0.46 mm, base as broad as vagina (Fig. 30B); bursal walls translucent with thick wrinkles; gonocoxite 1 with 4 apical fringe setae, 6–7 smaller setae on medial surface (Fig. 31B); gonocoxite 2 narrowly subtriangular with subacuminate apex, base narrowly extended laterally, 2 lateral ensiform setae with apical seta broader, apical nematiform setae on medioventral surface at 0.73× gonocoxite length.

##### Types.

For *Mecyclothorax cognatus* Sharp holotype female (BMNH) labeled: Mecyclothorax cognatus Type D.S. Haleakala Perkins 111 // Type // Hawaiian Is. Perkins 1904-336. // Haleakala Maui 5000 ft. Perkins IV 1894 // Atelothorax optatus Sharp compared with type E.B.B. // HOLOTYPE Mecyclothorax cognatus Sharp J.K. Liebherr 1998 (black-margined red label). For *Atelothorax optatus* Sharp holotype male (BPBM) platen mounted and labeled: Atelothorax / optatus / Type / D.S. / Haleakala / 1902 (written on obverse of mounting card) // Mecyclothorax / cognatus Sharp / Compared / with type E.B.B. // HSPA # / 1960 // HOLOTYPE / Atelothorax / optatus / Sharp / J.K. Liebherr 1998 (black-margined red label).

##### Distribution and habitat.

*Mecyclothorax cognatus* is restricted to forests in the Waikamoi region (Fig. 32) from 1200–1850 m elevation. The only recorded Perkins lot (No. 111) was collected iv–1894 near Ukulele Camp, with modern collections centered on the Ukulele Pipeline Koa-‘Ōhi‘a Mesic Forest at ~1500 m elevation northeast of the Ukulele Camp site. Beetles occur on and under bark of koa, in moss on trunks of ‘ōhi‘a, and on the ground in the leaf litter or under logs. Several have been collected in yellow-pan traps set on the ground.

#### (019) Mecyclothorax anchisteus sp. n.

Taxon classificationAnimaliaColeopteraCarabidae

http://zoobank.org/FF5D8759-E3F4-45D6-9FE2-67EEFE7B39B6

Figs 32 , 33A

##### Diagnosis.

This species is easily diagnosed by the distinctly cordate pronotum, MPW/BPW = 1.57, with glabrous hind angles. The pronotal lateral margins are distinctly convergent anterad the acute and acuminately projected hind angles (Fig. 33A). The head, pronotum, and elytra are a uniformly dark rufous. The eyes are moderately convex, ocular ratio = 1.43, but rather small, covering only ¾ of the little protruded ocular lobe; ocular lobe ratio = 0.76. The elytra are broadly subovoid, with the humeri narrowly rounded; MEW/HuW = 2.0. The discal elytral intervals are covered with irregular transverse-line microsculpture, the lines not joined into a mesh. Setal formula 2 1 2 1[sae]. Standardized body length 5.1 mm.

##### Description

(n = 1). *Head capsule* with frontal grooves broad near clypeus, a lateral carina to anterior supraorbital seta; dorsal surface of neck slightly concave; labral anterior margin broadly, shallowly emarginate, antennae filiform, sparse pelage of short setae present on antennomeres 2–3; mentum tooth with sides acute, apex tightly rounded. *Pronotum* moderately transverse, MPW/PL = 1.26; median base depressed relative to disc, with small punctures and lateral wrinkles; basal margin straight, slightly indented posterad laterobasal depressions; median longitudinal impression shallow, very finely incised at depth; anterior transverse impression very shallow, narrow, crossed medially by longitudinal wrinkles; anterior callosity nearly flat, crossed by indistinct wrinkles; front angles slightly projected, tightly rounded; anterior width greater than basal width, APW/BPW = 1.07; lateral marginal depression narrow throughout, margin upturned to finely beaded before sinuation; laterobasal depression smooth, laterally elevated to projected lateral margin. *Proepisternum* with smooth hind marginal groove; prosternal process medially depressed, a broad lateral marginal bead that is narrowed anteriorly. *Elytra* with convex disc, sides depressed; basal groove slightly recurved to broadly rounded humeral angle; parascutellar seta present; parascutellar striole continuous, with 4–5 punctures; sutural interval more convex than intervals 2–4, sutural juncture upraised; sutural and 2^nd^ striae of subequal depth from base to apex; striae 1–8 complete, stria 7 slightly shallower, associated intervals moderately convex; discal striae with slightly irregular punctulae basally, smooth and deep apically; 7^th^ and 8^th^ intervals of similar convexity mesad subapical sinuation; 2 dorsal elytral setae at 0.27× and 0.49–0.52× elytral length; setal impressions extended over 2/3 width of interval 3; subapical seta present, apical seta absent; lateral elytral setae arranged in anterior series of 7 setae and posterior series 6 setae; elytral marginal depression narrow throughout, margin slightly upraised at humerus; subapical sinuation shallow, broad. *Mesepisternum* with ~5 punctures in 1 row; metepisternal width to length ratio = 0.81; metepisternum/metepimeron suture distinct. *Abdomen* with irregular lateral wrinkles on ventrites 1–5; suture between ventrites 2 and 3 complete; apical ventrite of female with 4 equally spaced marginal setae and a median trapezoid of 4 setae, the basal pair longer. *Legs*-metatarsomere 1/metatibial length ratio = 0.22; metatarsomere 4 length along outer lobe 1.37× medial tarsomere length, apical and subapical setae present; metatarsal dorsolateral sulci very broad, median area rough to carinate. *Microsculpture* of vertex with isodiametric to slightly stretched isodiametric sculpticells in transverse rows; pronotal disc with transverse mesh, sculpticell breadth 3–4× length; pronotal median base with isodiametric to transverse sculpticells; elytral disc with irregular transverse lines, apex with more regular transverse lines; metasternum with distinct transverse mesh; laterobasal abdominal ventrites with swirling isodiametric and transverse microsculpture. *Coloration* of antennomere 1 flavous, antennomeres 2–3 rufoflavous, 4–11 rufobrunneous; proepipleuron and proepisternum rufobrunneous; elytral apex paler than disc, rufoflavous to position of subapical seta; elytral epipleuron rufoflavous laterally, rufobrunneous ventrally, metepisternum rufobrunneous; abdomen medially rufobrunneous, laterally rufoflavous; abdominal apical ventrite 6 with apical 1/3 paler, flavous; metafemur flavous with piceous cloud covering basal half; metatibia rufoflavous with brunneous cast.

**Female reproductive tract.** The lone female holotype was not dissected.

##### Holotype.

Female (NMNH) labeled: HI:Maui Haleakala N.P. / Kipahulu Vy. West Camp / 20-V-1998 lot01 1950 m / el. pyrethrum fog mossy / ohia D.A. Polhemus // HOLOTYPE / Mecyclothorax / anchisteus / Sharp / J.K. Liebherr 2015 (black-margined red label).

##### Etymology.

The species epithet anchisteus is Greek for next of kin (Jaeger 1955), signifying this species’ close affinities to *Mecyclothorax cognatus*, *Mecyclothorax affinis*, and *Mecyclothorax consanguineus*.

##### Distribution and habitat.

This species is known from a single specimen collected at the head of Kīpahulu Valley (Fig. 32) after application of pyrethrin fog to a mossy ‘ōhi‘a tree.

#### (020) Mecyclothorax consanguineus sp. n.

Taxon classificationAnimaliaColeopteraCarabidae

http://zoobank.org/B1A4F936-4449-4C02-A049-034860DE3022

**Figure 35.**
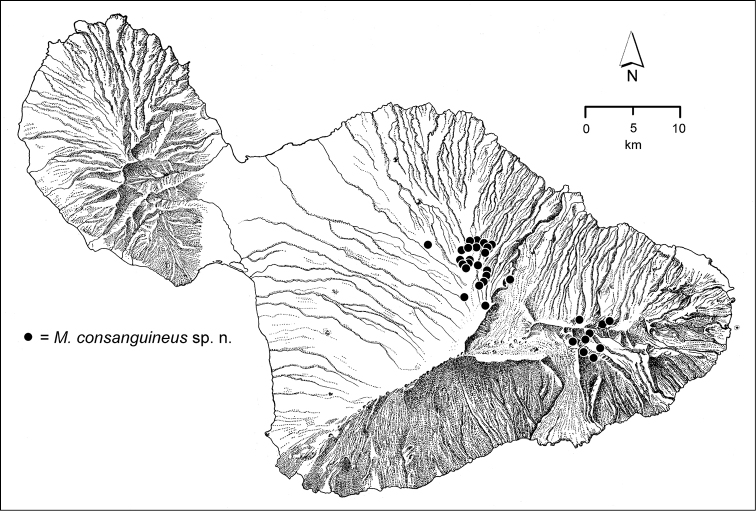
Recorded geographic distribution of *Mecyclothorax consanguineus*.

Figs 30C , 31C , 33B , 34A–I , 35

##### Diagnosis.

Of species in this group characterized by broader, shorter elytra and a basally constricted, quadrisetose pronotum (Figs 28B–C, 33B), this species can be diagnosed by the narrowed humeri, MEW/HuW = 1.91–2.04. As in the morphologically similar and partially sympatric *Mecyclothorax cognatus*, the pronotum is moderately constricted basally—MPW/BPW = 1.37–1.60—and the discal elytral striae are finely impressed and lined with minute punctures, the associated intervals slightly convex. The male aedeagal median lobe configuration is unique in the mucronate apex (Fig. 34A–I). Setal formula 2 2(1) 2 2; the basal pronotal seta may be unilaterally present, though at least one seta was observed in all examined specimens. Standardized body length 4.1–5.3 mm.

##### Description

(n = 5). *Head capsule* with frontal grooves broad near clypeus, a lateral carina to anterior supraorbital seta; dorsal surface of neck flat to slightly concave; eyes moderately developed, ocular ratio = 1.39–1.51, ocular lobe ratio = 0.72–0.77; labral anterior margin with subangulate emargination, excavated 0.2× length medially; antennae filiform, antennomeres 2-3 with sparse pelage of short setae; mentum tooth with sides acute, apex tightly rounded. *Pronotum* moderately narrow, MPW/PL = 1.15–1.25, and moderately to rather constricted basally, MPW/BPW = 1.37–1.60; hind angle right to slightly acute, projected, the lateral margin convergent to parallel just anterad the angle; median base very depressed relative to disc, shallow longitudinal wrinkles and small punctures covering surface; basal margin straight medially, slightly indented posterad laterobasal depressions; median longitudinal impression very shallow, indistinct; anterior transverse impression very shallow, broad, crossed by longitudinal wrinkles; anterior callosity nearly flat with wrinkles on the posterior half; front angles projected, tightly rounded; front and basal pronotal angles variably subequal, APW/BPW = 0.92–1.03; lateral marginal depression slightly broader at front angle, moderately narrow behind, edge upturned; laterobasal depression smooth with median extension from disc as a tubercle. *Proepisternum* with 6 punctures along hind marginal groove; prosternal process medially depressed, with broad lateral marginal bead. *Elytra* with moderately narrow humeri, the disc convex and side moderately sloped; basal groove slightly recurved to hitched humeral angle at base of elytral lateral depression; parascutellar seta present; parascutellar striole shallow, smooth anteriorly with 3 punctures in posterior portion; sutural interval equally convex as intervals 2–4 basally, more convex apically; sutural and 2^nd^ striae of subequal depth from base to apex; sutural stria finely impressed, irregularly punctate basally, smooth and deep apically, striae 2–5 with minute punctulae on disc, striae slightly irregular along length; 7^th^ and 8^th^ interval of similar convexity mesad subapical sinuation; two dorsal elytral setae at 0.26–0.28× and 0.54–0.56× elytral length, setal impressions small, extended over ½ width of interval 3; apical and subapical setae present; lateral elytral setae arranged as an anterior series of 7 setae, a posterior series of 6 setae; elytral marginal depression moderately broad at humerus, gradually narrowed to beadlike margin at subapical sinuation; subapical sinuation shallow, nearly obsolete. *Mesepisternum* with ~8–9 punctures in 1–2 rows; metepisternal width to length ratio = 0.84; metepisternum/metepimeron suture distinct. *Abdomen* with irregular lateral wrinkles on ventrites 1–5; suture between ventrites 2 and 3 complete; apical ventrite of male with 2 marginal setae, apical ventrite of female with 4 equally spaced marginal setae plus a median trapezoid of 4 smaller, subequal setae. *Legs*-metatarsomere 1/metatibial length ratio = 0.21; metatarsomere 4 length along outer lobe 1.4× medial tarsomere length, apical and subapical setae present; metatarsal dorsolateral sulci deep, broad, median area strigose to carinate. *Microsculpture* of vertex a transverse mesh, sculpticell breadth 2× length; pronotal disc with reduced transverse mesh, sculpticell breadth 2–3× length; pronotal median base with distinct isodiametric and transverse sculpticells; elytral disc with transverse mesh, sculpticell breadth 3–4× length; elytral apex with transverse mesh, sculpticell breadth 3× length, to transverse lines; metasternum with distinct transverse mesh; laterobasal abdominal ventrites with swirling isodiametric and transverse microsculpture. *Coloration* of vertex rufobrunneous; antennomere 1 flavous, antennomeres 2–3 rufoflavous, 4–11 rufobrunneous; pronotal disc rufobrunneous, pronotal apical and lateral margins rufoflavous; proepipleuron rufoflavous, proepisternum rufobrunneous; elytral disc rufobrunneous, sutural interval concolorous to rufoflavous basally, rufoflavous to flavous apically; elytral marginal depression concolorous with disc to paler, rufoflavous basally, apex slightly paler, rufoflavous to position of subapical seta; elytral epipleuron and metepisternum rufobrunneous; abdomen with ventrites medially rufopiceous, ventrites 3–6 rufoflavous laterally; abdominal apical ventrite with apical half paler, flavous; metafemur flavous; metatibia rufobrunneous.

**Male genitalia** (n = 15). Aedeagal median lobe distinctly curved, variably robust, with ratio of distance from parameral articulation to tip versus depth at midlength ranging 3.1–4.2 (Fig. 34A, H), but always with apex narrowly extended well beyond ostial opening, and tip denticulate; a brief indentation along apicoventral surface (Fig. 34A–B, D–I); median lobe straight in ventral view, right margin slightly concave, left margin incurved before apparently blunt tip (Fig. 34C); internal sac generally lightly spiculated, but with indistinct ventral microtrichial patch comprised of slightly larger microtrichia (Fig. 34A); flagellar plate relatively large, length 0.45× distance from parameral articulation to tip.

**Female reproductive tract** (n = 1). Bursa copulatrix columnar with apex rounded, bursa narrowed apically, length 1.05 mm, apical breadth 0.29 mm, basal breadth 0.36 mm equal to vagina breadth (Fig. 30C); bursal walls translucent with thick wrinkles; gonocoxite 1 with 3–4 apical fringe setae and 6–7 small setae on medial surface (Fig. 31C); gonocoxite 2 narrowly subtriangular with broad apex, base broadly extended laterally, 2 lateral ensiform setae with apical seta broader and longer, apical nematiform setae on medioventral surface at 0.74× gonocoxite length.

##### Holotype.

Male (CUIC) labeled: HI: Maui Haleakala NW / slope Waikamoi Pres. / trans. 3 @ 1700 m el. / 10-IV-1991 sifting / litter J.K. Liebherr // HOLOTYPE / Mecyclothorax / consanguineus / Liebherr / J.K. Liebherr 2015 (black-margined red label).

##### Paratypes.

261 specimens (see Appendix).

##### Etymology.

The Latin adjectival consanguineus means related by blood, or kindred (Brown 1956), and signifies this species’ close relationship to the previous three species.

##### Distribution and habitat.

*Mecyclothorax consanguineus* has an elevationally broad windward distribution that spans the Waikamoi Koa-‘Ōhi‘a Mesic Forest to the lower elevation ‘Ōhi‘a-‘Ōlapa Wet Forest, and then disjunctly extends through the Hāna Bogs to the head of Kīpahulu Valley (Fig. 35). Collection localities range 1210–2438 m elevation. A population is known from the western wall of Ke‘anae Valley, 1325 m elevation. Another marginal population occurs at Paliku, 1830 m elevation, in the eastern end of Haleakalā Crater where windward fog and moisture spill over the headwall of Kīpahulu Valley creating a lens of mesic habitat. Beetles occur in forests composed of varying mixtures of koa and ‘ōhi‘a, as well as more open, higher elevation habitats including *Dubautia* (kupaoa) and *Leptecophylla* (pūkiawe). That the species can occur in such open habitats begs the question of why it has not been recorded from the Hanawī face of Haleakalā, an area of open koa Forest with extensive *Dicranopteris* (uluhe) fern understory. However our sampling of the Hanawī Koa-Uluhe Formation was very cursory, leaving the question for a more intensive biotic survey.

#### (021) Mecyclothorax aeneus

Taxon classificationAnimaliaColeopteraCarabidae

Sharp

**Figure 36.**
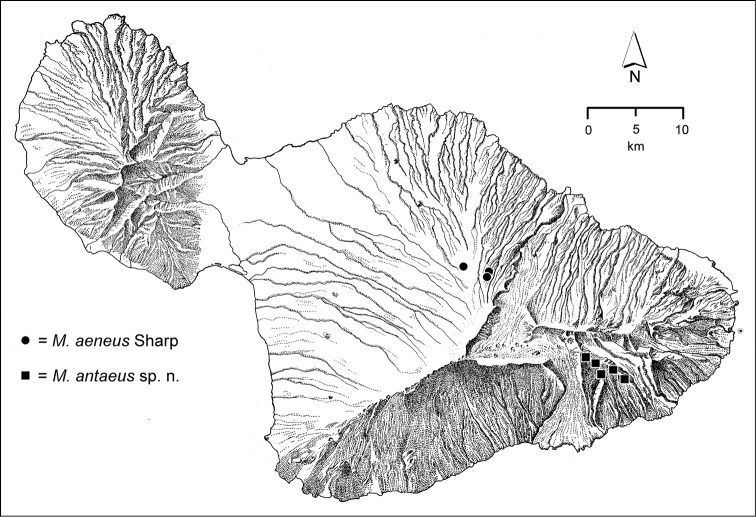
Recorded geographic distributions of *Mecyclothorax robustus* group species.

Figs 30D , 31D , 33C , 36

Mecyclothorax aeneus Sharp 1903: 255; Britton 1948b: 164.

##### Diagnosis.

The narrow body and moderately convex elytral intervals with nearly smooth striae set this species apart. Individuals of this species can be diagnosed by the narrow pronotum–MPW/PL = 1.15–1.19–with its lateral margins convergent for 0.1× the pronotal length anterad the acute, projected hind angles (Fig. 33C). The elytra are narrowly ellipsoid, with distinctly recurved basal grooves leading to proximate, subangulate humeri; MEW/HuW = 2.04–2.15. The elytral disc is rufobrunneous, with a cupreous reflection enhanced by a distinct granulate isodiametric mesh. At the elytral apex, the 8^th^ interval is more convex than the fused apical portion of intervals 5 + 7. Setal formula 2 2 2 2. Standardized body length 5.7–5.8 mm for specimens available to the author, though Britton (1948b) listed the size range as 4.8–5.8 mm.

##### Identification

(n = 2). The eyes are moderately convex, ocular ratio = 1.42, covering ¾ of the protruded ocular lobe, ocular lobe ratio = 0.76. There is a deep carinate groove at the juncture of lobe and gena. The pronotal median base is depressed relative to the disc, and irregularly covered with fine punctures, longitudinal wrinkles lining the juncture of base and disc. The pronotal anterior transverse impression is shallow and broad, not incised, and crossed by dense, deep longitudinal wrinkles that extend across the anterior callosity. The pronotal laterobasal depressions are broad and smooth, with a well-developed median tubercle. The mesepisternum bears ~16 punctures in 2–3 rows. Microsculpture is well developed, with the vertex covered by an isodiametric mesh in transverse rows, and the pronotal base covered with mixture of granulate isodiametric and transverse sculpticells.

**Female reproductive tract** (n = 1). Bursa copulatrix columnar with rounded apex, length 1.14 mm, breadth 0.39 mm, base as broad as vagina (Fig. 30D); bursal walls translucent with thick wrinkles in basal half, more transparent and little wrinkled apically; gonocoxite 1 with 3–4 apical fringe setae and 8–9 small setae on medial surface (Fig. 31D); gonocoxite 2 narrowly subtriangular with broad apex, tightly rounded tip, 2 lateral ensiform setae with apical seta broader and longer, apical nematiform setae on medioventral surface at 0.72× gonocoxite length.

##### Lectotype.

Male (BMNH) hereby designated, labeled: Thriscothorax aeneus D.S. Type Haleakala Perkins 383 // Type // Hawaiian Is. Perkins 1904-336 // LECTOTYPE Mecyclothorax aeneus Sharp J.K. Liebherr 1998 (black-margined red label).

##### Distribution and habitat.

*Mecyclothorax aeneus* is a species of the mesic Waikamoi forests (Fig. 36). In 1894 Perkins collected two specimens near the leeward forest edge at Ukulele Camp, with two more recently collected specimens from 1700–1850 m elevation in the Honomanu drainage complementing the original type series. The two recent collections were made from moss and humus adhering to ‘ōhi‘a trunks.

#### (022) Mecyclothorax antaeus sp. n.

Taxon classificationAnimaliaColeopteraCarabidae

http://zoobank.org/F8551848-5C97-426E-940D-31B4C1AEB4E8

**Figure 37.**
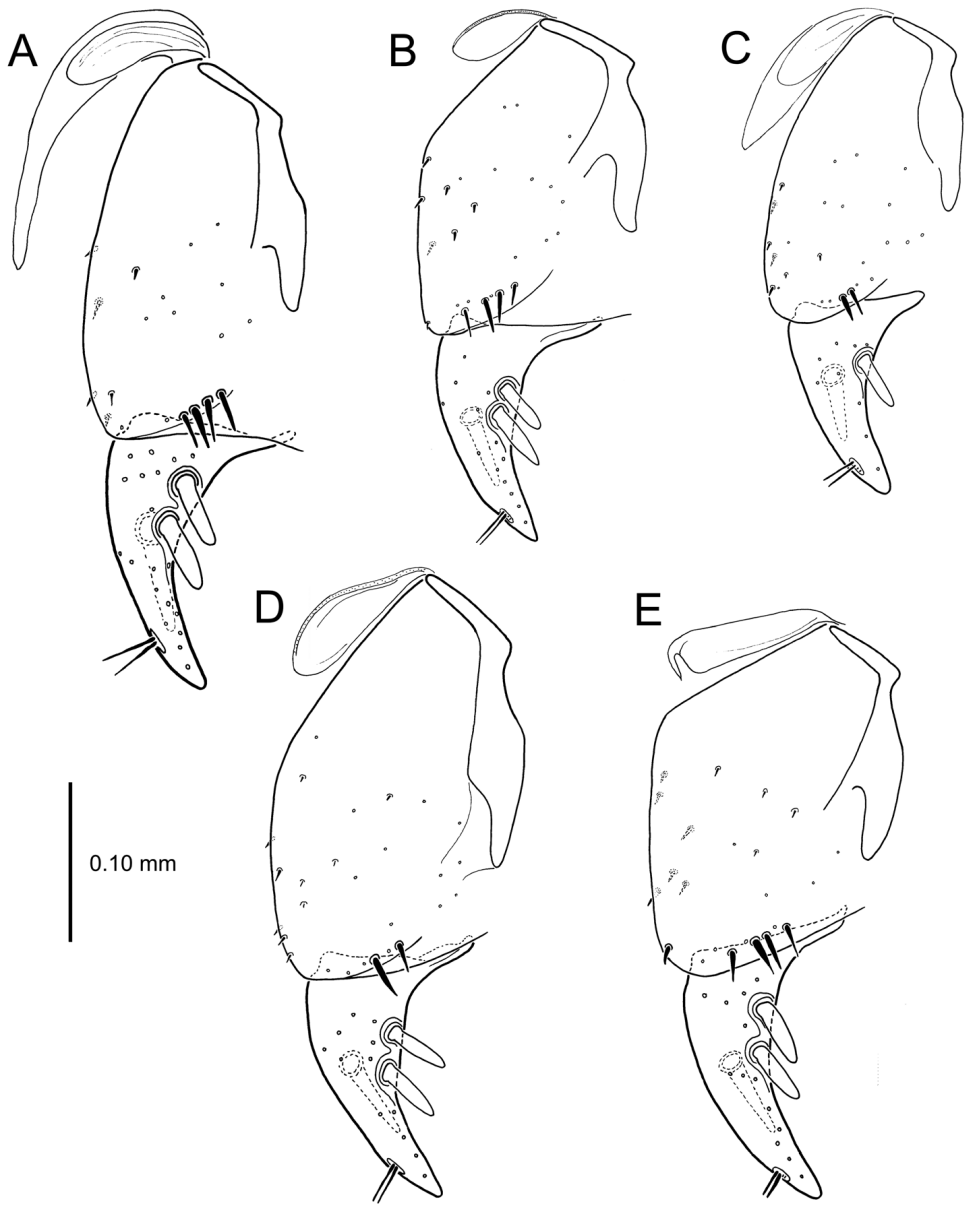
Left female gonocoxa, *Mecyclothorax robustus* group species, ventral view. **A** *Mecyclothorax antaeus* (Kīpahulu west rim, 1850 m) **B** *Mecyclothorax cymindicus* (Ukulele Camp Pipeline, 1525 m) **C** *Mecyclothorax cymindulus* (Kīpahulu, 1950 m) **D** *Mecyclothorax robustus* (Waikamoi, 1310 m) **E** *Mecyclothorax haydeni* (Kīpahulu, 1500).

Figs 30E , 33D , 34J–L , 36 , 37A

##### Diagnosis.

**Figure 38.**
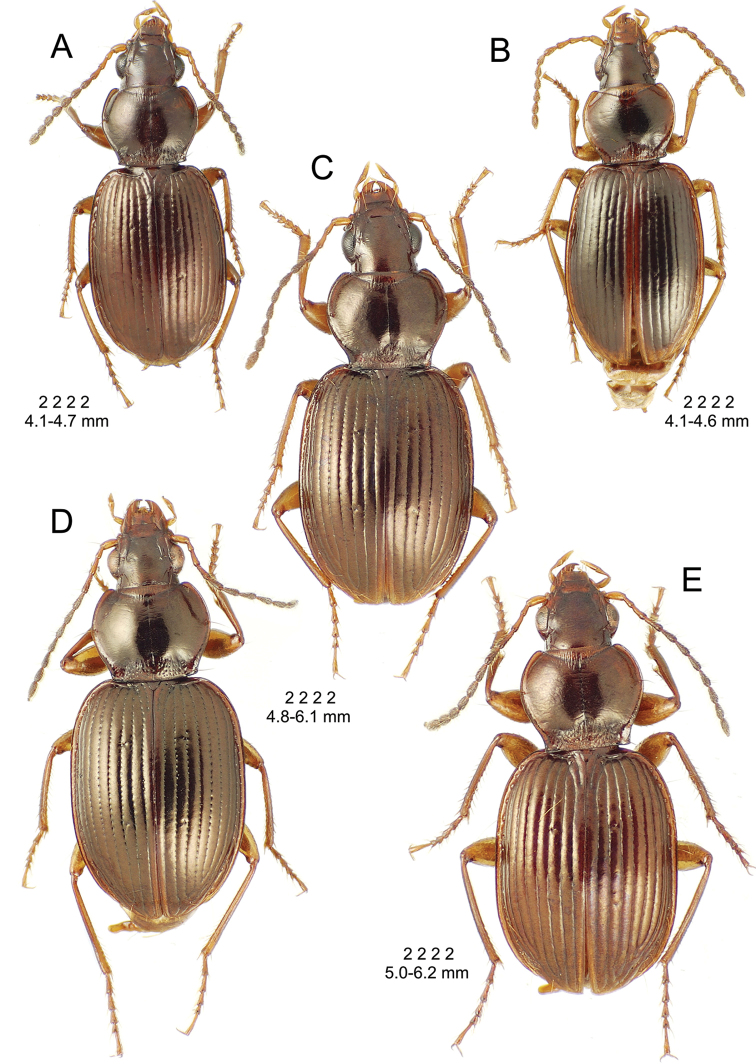
*Mecyclothorax robustus* group species, dorsal habitus view. **A** *Mecyclothorax cymindicus* (Ukulele Camp Pipeline, 1525 m) **B** *Mecyclothorax cymindulus* (Kīpahulu, 1950 m) **C** *Mecyclothorax robustus* (Ke‘anae, 1325 m) **D** *Mecyclothorax robustus* (Waikamoi, 1170 m). **E** *Mecyclothorax haydeni* (Helele‘ike‘oha, 1615 m).

This species can be diagnosed by the broad elytra with sides subparallel at midlength (Fig. 33D), a configuration shared with *Mecyclothorax robustus* and *Mecyclothorax haydeni* (Fig. 38C–E), however the discal elytral striae are smooth to only minutely punctate instead of distinctly punctate, and the associated intervals are only moderately convex instead of convex. The pronotum has the lateral margin parallel to slightly convergent anterad the right hind angles, and the pronotal median base is covered with fine punctures. Beetles of the other two species have pronota with parallel to divergent basal lateral margins. The male aedeagal median lobe of *Mecyclothorax antaeus* males has the apex elongate and parallel sided with a downturned tip (Fig. 34J–K), versus the apically flattened median lobe of *Mecyclothorax robustus* (Fig. 41) and the elongate, evenly curved apex observed in males of *Mecyclothorax haydeni* (Fig. 43). Setal formula 2 2 2 2. Standardized body length 4.6–5.9 mm.

##### Description

(n = 5). *Head capsule* with frontal grooves broad near clypeus, a lateral carina to anterior supraorbital seta; dorsal surface of neck flat to slightly concave; eyes moderately convex, ocular ratio = 1.46–1.50, ocular lobe distinctly protruded from gena, ocular lobe ratio = 0.71–0.78, an abruptly depressed slot at juncture of lobe and gena; labral anterior margin broadly shallowly emarginate; antennae filiform, antennomeres 2–3 with sparse pelage of short setae; mentum tooth with sides acute, apex tightly rounded. *Pronotum* subcordate, quadrate to slightly transverse, MPW/BPW = 1.38–1.45, MPW/PL = 1.18–1.25; hind angle right, projected, the margin behind convex; basal margin slightly convex between hind angles; median longitudinal impression shallow, finely incised; anterior transverse impression broad, shallow, crossed by longitudinal wrinkles, lined with granulate isodiametric microsculpture; anterior callosity slightly elevated, covered with dense longitudinal wrinkles, strigose; front angles slightly projected, rounded; base broader than distance between front angles, APW/BPW = 0.86–0.98; lateral marginal depression moderately broad, explanate, edge upturned; laterobasal depression broad, smooth, with median tubercle. *Proepisternum* with 6 indistinct punctures along hind marginal groove; prosternal process medially depressed, with broad lateral marginal bead. *Elytra* subquadrate, humeri moderately narrow, rounded, sides subparallel at midlength; elytral disc convex, sides progressively sloped laterally; basal groove recurved to subangulate humeri, the lateral margin upraised, and humeral angle defined by hitch at base of lateral marginal depression, MEW/HuW = 2.02–2.13; parascutellar seta present; parascutellar striole deep with 5 punctures; sutural interval more convex than intervals 2–4, sutural juncture upraised; sutural and 2^nd^ striae of subequal depth from base to apex; sutural and lateral discal striae 2–6 minutely punctate, striae slightly irregular along length; 7^th^ and 8^th^ intervals of similar convexity mesad subapical sinuation; 2 dorsal elytral setae at 0.29–0.34× and 0.55–0.60× elytral length, setal impressions small, extended over ½ width of interval 3; apical and subapical setae present; lateral elytral setae arranged in an anterior series of 7(6) setae and a posterior series of 6 setae; elytral marginal depression moderately broad in anterior half, narrowed anterad subapical sinuation; subapical sinuation shallow, broad. *Mesepisternum* with ~14 punctures in 2–3 rows; metepisternal width to length ratio = 0.81; metepisternum/metepimeron suture distinct. *Abdomen* with irregular lateral wrinkles on ventrites 1–5; suture between ventrites 2 and 3 complete; apical ventrite of male with 2 marginal setae, apical ventrite of female with 4 equally spaced setae and median patch of 4(5) short, subequal setae. *Legs*-metatarsomere 1/metatibial length ratio = 0.20; metatarsomere 4 length along outer lobe 1.33× medial tarsomere length, apical and subapical setae present; metatarsal dorsolateral sulci deep, broad, median area strigose to carinate. *Microsculpture* of vertex an isodiametric mesh; pronotal disc with transverse mesh, sculpticell breadth 2–3× length; pronotal median base with distinct to granulate isodiametric mesh; elytral disc and apex with transverse mesh, sculpticell breadth 3× length, to transverse lines; metasternum with distinct transverse mesh; laterobasal abdominal ventrites with swirling isodiametric and transverse microsculpture. *Coloration* of vertex rufobrunneous with a purplish reflection; antennomere 1 flavous, 2–3 rufoflavous, 4–11 rufobrunneous; pronotal disc rufobrunneous with purplish reflection; pronotal margins rufoflavous inside front angles, otherwise concolorous with disc; proepipleuron rufoflavous, proepisternum rufobrunneous; elytral disc rufobrunneous with purplish reflection; sutural interval concolorous with disc basally, rufoflavous apically; elytral lateral marginal depression rufoflavous, apex slightly paler than disc, rufoflavous to position of subapical seta; elytral epipleuron rufoflavous laterally, rufobrunneous ventrally, metepisternum rufobrunneous; abdomen with ventrites medially rufobrunneous, marginally rufoflavous; apical half of ventrite 6 paler, flavous; metafemur flavous; metatibia rufoflavous.

**Male genitalia** (n = 4). Aedeagal median lobe gracile, distance from parameral articulation to tip 4.1× depth at midlength (Fig. 34J–K), apex narrowly extended beyond ostial opening, tip downturned with flat apical face; median lobe broadly curved rightward in ventral view, the apex parallel sided and tip blunt (Fig. 34L); internal sac tubular, elongate, with two ventral microtrichial patches, a larger basal patch and a smaller apical patch situated just distad a median constriction (Fig. 34J); flagellar plate of moderate size, length of sclerotized internal face 0.38× distance from parameral articulation to tip.

**Female reproductive tract** (n = 1). Bursa copulatrix columnar with rounded apex, bursa narrower in apical half, length 1.10 mm, apical breadth 0.32 mm, basal breadth 0.51 mm, broader than width of vagina (Fig. 30E); bursal walls translucent basally with think wrinkles, apical surface shagreened but not wrinkled; gonocoxite 1 with 3–4 apical fringe setae and 6–9 smaller setae on medial surface (Fig. 37A); gonocoxite 2 narrowly subtriangular with subacuminate apex, base extended laterally as a thin extension, 2 lateral ensiform setae, the apical seta slightly broader, apical nematiform setae on medioventral surface at 0.74× gonocoxite length.

##### Holotype.

Male (CUIC) labeled: HI: Maui / Haleakala N.P. / Kipahulu west rim ESE / Kuiki sift humus ex ohia 15-V-1993 lot 03 / el. 1850 m // J.K. Liebherr & / A.C. Medeiros / Collectors // HOLOTYPE / Mecyclothorax / antaeus / Liebherr / J.K. Liebherr 2015 (black-margined red label).

##### Paratypes.

39 paratype specimens plus 1 non-type specimen (see Appendix).

##### Etymology.

Antaeus was a “giant Libyan wrestler whose strength was renewed when he touched the earth (Brown 1956),” an apt epithet for this close relative of *Mecyclothorax robustus*. The name is treated as a noun.

##### Distribution and habitat.

*Mecyclothorax antaeus* is a species of the Manawainui Planeze, with known collection localities lining the western rim of Kīpahulu Valley (Fig. 36) at 1200–2145 m elevation. Specimens have been collected in association with ‘ōhi‘a, either from humus around tree bases, or from humus on tree trunks.

#### (023) Mecyclothorax cymindicus

Taxon classificationAnimaliaColeopteraCarabidae

Sharp

Figs 30F , 34M , 37B , 38A , 40

Mecyclothorax cymindicus Sharp 1903: 248; Britton 1948b: 164.

##### Diagnosis.

Along with *Mecyclothorax cymindulus*, the smallest-bodied beetles in the group (Fig. 38A–B) excepting the smallest individuals of *Mecyclothorax antaeus* (Fig. 33D); standardized body length for this species 4.1–4.7 mm. This and *Mecyclothorax cymindulus* are also the most narrow-bodied species, with subquadrate elytra and subangulate, laterally extended humeri (Figs 38A–B). This species deviates from *Mecyclothorax cymindulus* by the distinctly punctate discal elytral striae, the punctures in basal portions of striae 1–5 expanding strial breadth, and by the less transverse microsculpture; The elytral sutural interval is covered with isodiametric microsculpture, with the lateral intervals bearing a transverse mesh, sculpticell breadth 3–4× length, versus the transverse mesh on the sutural interval and transverse lines laterally observed in individuals of *Mecyclothorax cymindulus*. The eyes also tend to be more convex in this species—ocular ratio = 1.41–1.45—versus the ocular ratio = 1.34–1.43 of *Mecyclothorax cymindulus*. Setal formula 2 2 2 2.

##### Identification

(n = 5). The pronotum is little transverse, MPW/PL = 1.17–1.22, and the pronotal hind angles are acute and moderately projected, with the pronotal lateral margins slightly convergent anterad the angles; MPW/BPW = 1.36–1.54. The elytral humeral angles are defined by a hitch in the recurved basal groove at its juncture with the lateral marginal depression; MEW/HuW = 1.83–1.95. The vertex is covered with a transverse mesh, sculpticell breadth 2× length; pronotal disc with transverse mesh, sculpticell breadth 3–4× length, and pronotal base with distinct isodiametric sculpticells medially and a transverse mesh laterally.

**Male genitalia** (n = 1). Aedeagal median lobe gracile, distance from parameral articulation to tip 3.6× depth at midlength (Fig. 34M); apex moderately extended beyond ostial opening, apical face flat, tip rounded at juncture of apical face and ventral margin; internal sac with sclerotized dorsal microtrichial patch (based on distal position in uneverted specimen); flagellar plate length 0.47× parameral articulation-tip distance.

**Female reproductive tract** (n = 1). Bursa copulatrix columnar with rounded apex, length 1.03 mm, breadth 0.29 mm (Fig. 30F); bursal walls translucent, thickly wrinkled; gonocoxite 1 with 4 apical fringe setae and 7 smaller setae on medial surface (Fig. 37B); gonocoxite 2 narrowly subtriangular with broad apex, tip tightly rounded, 2 lateral ensiform setae, the apical seta broader and longer, apical nematiform setae on medioventral surface at 0.77× gonocoxite length.

##### Lectotype.

Female (BMNH) hereby designated, labeled: Mecyclothorax cymindicus Type D.S. Haleakala Perkins 680 // Type // Hawaiian Is. Perkins 1904-336 // LECTOTYPE Mecyclothorax cymindicus Sharp J.K. Liebherr 1998 (black-margined red label).

##### Distribution and habitat.

*Mecyclothorax cymindicus* is geographically restricted to the Waikamoi area (Fig. 40), with localities ranging 1210–1740 m elevation. Perkins’ collections were made in the vicinity of Ukukule Camp along the then leeward edge of the Koa-‘Ōhi‘a Mesic Forest, with more recent records from the Honomanu drainage to the east and Waikamoi Gulch from 1210–1435 m elevation. Specimens have been recorded in association with ‘ōhi‘a trunks, logs, or leaf litter, and *Cibotium* (hāpu‘u).

#### (024) Mecyclothorax cymindulus sp. n.

Taxon classificationAnimaliaColeopteraCarabidae

http://zoobank.org/2F914232-3664-4139-AB0A-AC7ADA9DBC8C

**Figure 39.**
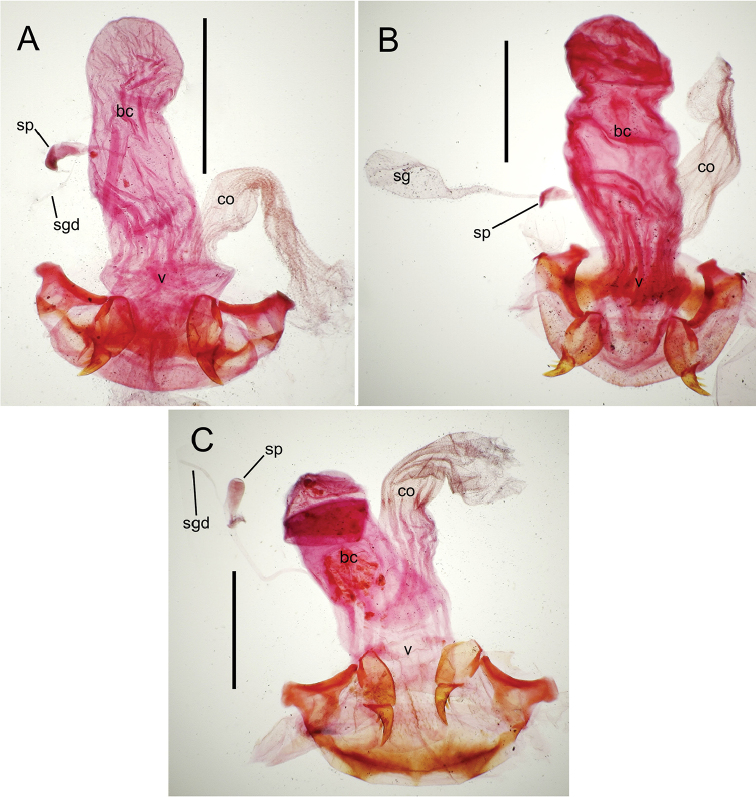
Female bursa copulatrix and associated reproductive structures, *Mecyclothorax robustus* group species, ventral view (for abbreviations see Table 2, p. 23). **A** *Mecyclothorax cymindulus* (Kīpahulu, 1950 m) **B** *Mecyclothorax robustus* (Waikamoi, 1310 m) **C** *Mecyclothorax haydeni* (Kīpahulu, 1500 m). Scale bar = 0.50 mm.

**Figure 40.**
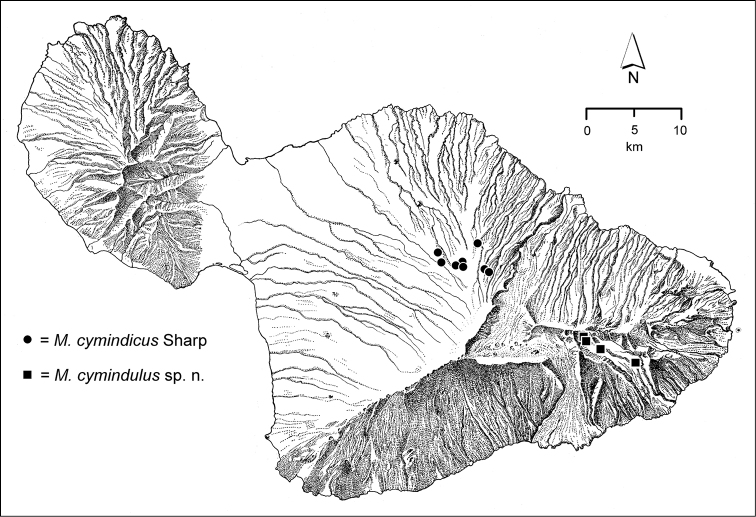
Recorded geographic distributions of *Mecyclothorax robustus* group species.

Figs 34N , 37C , 38B , 39A , 40

##### Diagnosis.

Differing from its most similar species group member *Mecyclothorax cymindicus* (Fig. 38A–B) by: 1, pronotal hind angles obtuse due to rounded basal margin posterad angle, the pronotal lateral margin subparallel anterad angle; 2, broad, minutely punctate discal elytral striae over basal half of length; 3, elytra with transverse-mesh microsculpture on the sutural interval, and parallel-lined microsculpture laterally on disc and at apex. If a male is available, the aedeagal median lobe of *Mecyclothorax cymindulus* has a more flattened apex with a subangulate, ventrally angled tip (Fig. 34N), versus the more rounded apex with ventrally expanded tip of male *Mecyclothorax cymindicus* (Fig. 34 M). Setal formula 2 2 2 2. Standardized body length 4.1–4.6 mm.

##### Description

(n = 5). *Head capsule* with frontal grooves straight, a lateral carina to anterior supraorbital seta; dorsal surface of neck flat to slightly concave; ocular lobe moderately extended from gena, eyes not covering posterior portion of lobe, ocular ratio = 1.34–1.41, ocular lobe ratio = 0.70–0.76; labral anterior margin subangulately excavated medially to 0.2× length; antennae broader in apical half, submoniliform, antennomeres 2–3 with sparse pelage of short setae; mentum tooth with sides acute, apex tightly rounded. *Pronotum* moderately transverse, MPW/PL = 1.21–1.25, base broad, MPW/BPW = 1.41–1.49; median base depressed relative to disc, covered with irregular punctures and longitudinal wrinkles; basal margin slightly convex between laterobasal depressions; median longitudinal impression shallow, very finely incised at depth; anterior transverse impression very shallow, broad, crossed by fine longitudinal wrinkles; anterior callosity slightly convex, crossed by numerous wrinkles; front angles not projected, rounded; anterior and basal widths subequal, APW/BPW = 0.95–1.04; lateral marginal depression broader, beaded at front angle, evenly expanded along midlength, moderately broad and upturned toward basal angle; laterobasal depression smooth, broad, with or without convex median extension from disc. *Proepisternum* with 6 punctures along hind marginal groove; prosternal process medially depressed, a broad marginal bead laterally. *Elytra* with convex disc, sides more sloped; basal groove straight from sutural stria laterally to subangulate humeri; elytral slightly narrowed basally, subquadrate, MEW/HuW = 1.83–1.95; parascutellar seta present; parascutellar striole with 4–5 punctures, continuous between punctures; sutural interval more convex than lateral intervals; sutural and 2^nd^ striae of subequal depth from base to apex; discal striae 1–5 and base of 6 minutely punctate, striae slightly irregular, associated intervals slightly convex; 7^th^ and 8^th^ interval of similar convexity mesad subapical sinuation; 2 dorsal elytral setae at 0. 25–0.27× and 0.57–0.59× elytral length, setal impressions extended over 2/3 width of interval 3; apical and subapical setae present; lateral elytral setae arranged as anterior series of 7 setae, posterior series of 6 setae; elytral marginal depression broadest at humerus, gradually narrowed to a beadlike margin at subapical sinuation; subapical sinuation shallow, abruptly incurved anteriorly. *Mesepisternum* with ~10 punctures in 2 rows; metepisternal width to length ratio = 0.79; metepisternum/metepimeron suture distinct. *Abdomen* with irregular lateral wrinkles on ventrites 1–5; suture between ventrites 2 and 3 reduced, effaced laterally; apical male ventrite with 2 marginal setae, apical female ventrite with 4 equally spaced marginal setae and a median trapezoid of 4 subequally short setae. *Legs*-metatarsomere 1/metatibial length ratio = 0.17; metatarsomere 4 length along outer lobe 1.35× medial tarsomere length, apical and subapical setae present; metatarsal dorsolateral sulci deep, broad, basal tarsomeres medially carinate. *Microsculpture* of vertex transverse, sculpticell breadth 2× length; pronotal disc with transverse mesh, sculpticell breadth 3–4× length; pronotal median base with distinct isodiametric and transverse sculpticells; metasternum with distinct transverse mesh; laterobasal abdominal ventrites with swirling isodiametric and transverse microsculpture. *Coloration* of vertex rufobrunneous; antennomeres 1–3 flavous, 4–11 rufobrunneous; pronotal disc rufobrunneous, pronotal margins slightly paler; proepipleuron and proepisternum rufoflavous; elytral disc rufobrunneous, metallic reflection present due to microsculpture; sutural interval rufous basally, flavous apically; elytral marginal depression narrowly flavous, apex flavous to juncture of intervals 3 and 4; elytral epipleuron flavous, metepisternum rufoflavous; abdomen with ventrites 1–6 medially rufoflavous, laterally flavous, ventrite 6 with apical half flavous; metafemur flavous; metatibia rufoflavous.

**Male genitalia** (n = 1). Aedeagal median lobe gracile, distance from parameral articulation to tip 4.0× depth at midlength (Fig. 34N); apex moderately extended beyond ostial opening, apical face flat, tip distinctly angulate at juncture of apical face and ventral margin; internal sac with lightly sclerotized dorsal microtrichial patch, and ventral face covered with fine spicules; flagellar plate length 0.47× parameral articulation-tip distance.

**Female reproductive tract** (n = 1). Bursa copulatrix columnar with rounded apex, narrowed along midlength, length 0.93 mm, medial breadth 0.31 mm, basal breadth at vagina 0.43 mm (Fig. 39A); bursal walls translucent with thin wrinkles; gonocoxite 1 with 2–3 apical fringe setae and 6–7 smaller setae on medial surface (Fig. 37C); gonocoxite 2 narrowly subtriangular with broad apex, tip tightly rounded, 1–2 lateral ensiform setae, apical nematiform setae on medioventral surface at 0.76× gonocoxite length.

##### Holotype.

Female (BPBM) labeled: Kipahulu Valley / Maui Camp 1 / 945 m, 6–12.VIII.67 // N. Wilson / Collector / BISHOP // ? cymindicus (E.C.Z. handwriting) // HOLOTYPE / Mecyclothorax / cymindulus / Liebherr / J.K. Liebherr 2015 (black-margined red label).

##### Paratypes.

HI: Maui: Haleakala N.P., Kipahulu Vy., sift litter, 1500 m el., 09-v-1991 lot 03, Jessel/Medeiros (CUIC, 1), Mauka Ridge, pyrethrin fog *Metrosideros*/moss, 2055 m el., 21-v-1998 lot 01, Polhemus (NMNH, 1), West Camp, pyrethrin fog *Metrosideros*/moss, 1960 m el., 20-v-1998 lot 01, Polhemus (CUIC, 1; NMNH, 2).

##### Etymology.

This epithet is taken from the Latin noun cymindis, meaning night hawk, modified with the diminutive ending -ule. Beetles of this species are the same size as those of *Mecyclothorax cymindicus*, but use of the common stem for the epithet is meant to connote the two species’ affinities; a convention used by Perrault (1984, 1986, 1988, 1989) to deal with the rampant *Mecyclothorax* diversity of Tahiti.

##### Distribution and habitat.

Beetles of *Mecyclothorax cymindulus* are recorded only from Kīpahulu Valley, 945–2055 m elevations (Fig. 40). They have been discovered in sifted leaf litter or on mossy ‘ōhi‘a trunks.

#### (025) Mecyclothorax robustus

Taxon classificationAnimaliaColeopteraCarabidae

(Blackburn)

**Figure 41.**
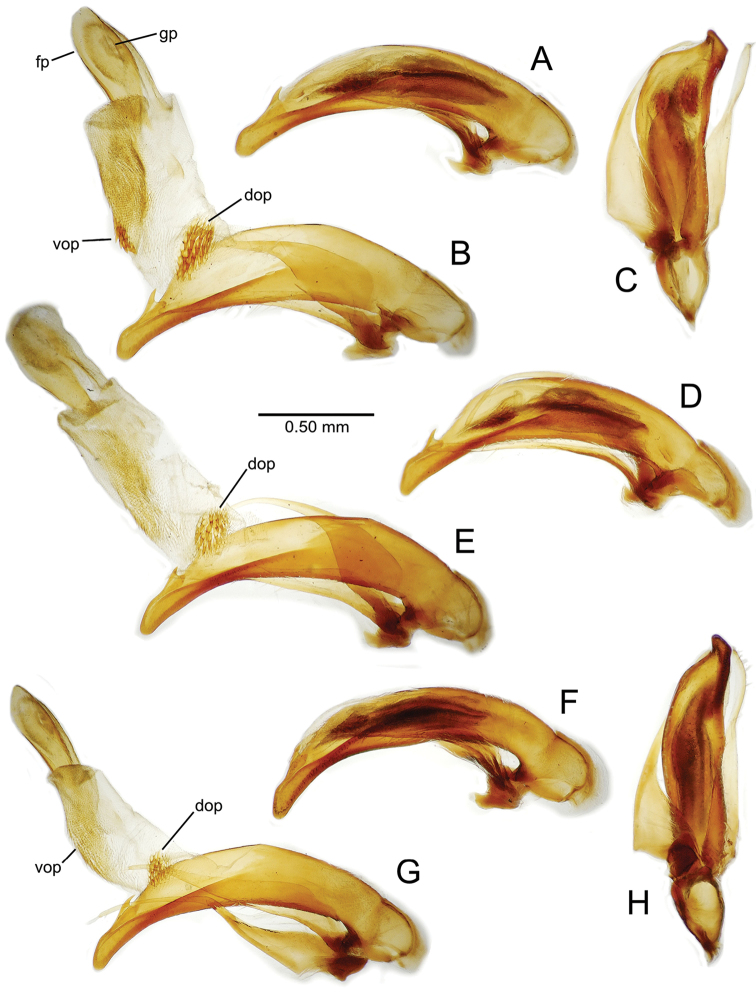
Male aedeagus, *Mecyclothorax robustus* (for abbreviations see Table 2, p. 23). **A–C** (Ukulele Camp Pipeline, 1495 m). **A** Right view **B** Right view, sac everted **C** Ventral view **D–E** (Ko‘olau Gap, 1325 m) **D** Right view **E** Right view, sac everted **F–H** (Kuhiwa, 1590 m) **F** Right view **G** Right view, sac everted **H** Ventral view.

**Figure 42.**
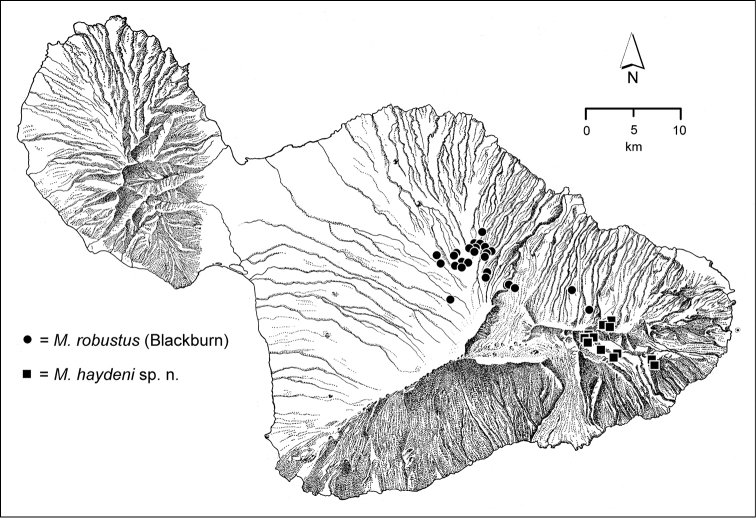
Recorded geographic distributions of *Mecyclothorax robustus* group species.

Figs 37D , 38C–D , 39B , 41 , 42

Cyclothorax robustus Blackburn 1881: 228; Blackburn and Sharp 1885: 215.
Thriscothorax robustus , Sharp 1903: 268; Swezey 1954: 7 (koa associate).
Mecyclothorax robustus , Britton 1948b: 166.
Mecyclothorax robustus Sharp 1903: 255.
Mecyclothorax robustus , Britton 1948b: 166 (synonymy).

##### Diagnosis.

**Figure 43.**
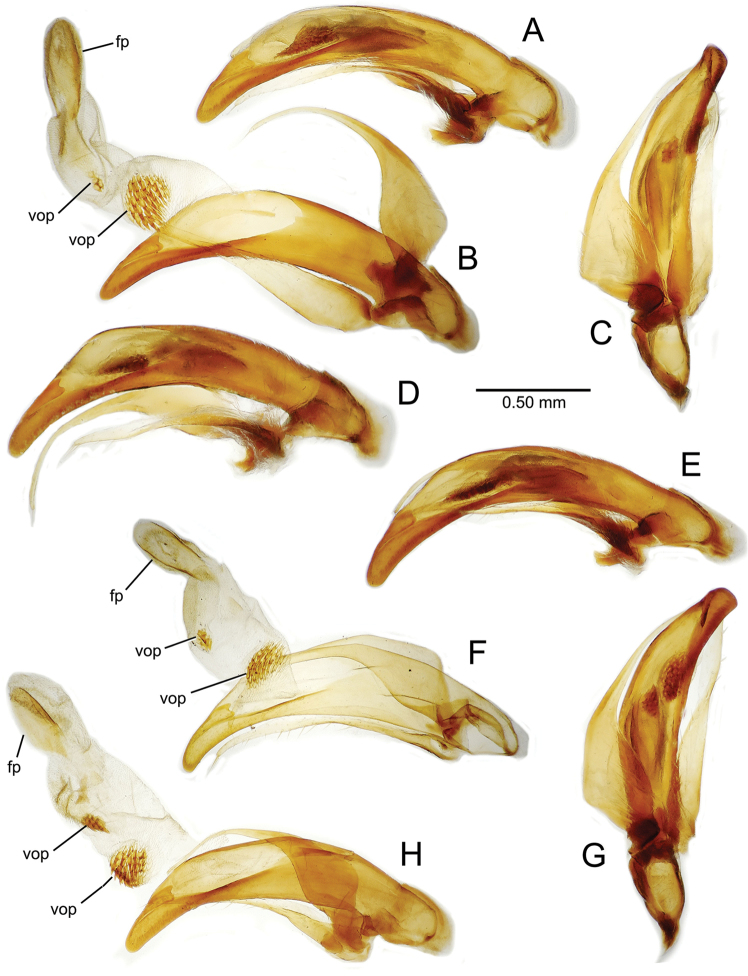
Male aedeagus, *Mecyclothorax haydeni* (for abbreviations see Table 2, p. 23). **A–C** (Helele‘ike‘oha, 1615 m). **A** Right view **B** Right view, sac everted **C** Ventral view. **D–G** (Kīpahulu, 1845-1960 m) **D–E** Right view **F** Right view, sac everted **G** Ventral view. **H** Right view, sac everted (Kaumakani, 1127 m).

This and *Mecyclothorax haydeni* represent the two larger bodied species in this group with subquadrate elytra, the discal elytral striae lined with distinct, round punctures (Figs 38C–E). Of the two species, the discal elytral striae are more distinctly and regularly punctate in *Mecyclothorax robustus*, though this characteristic is variable. In aggregate, individuals of *Mecyclothorax robustus* have larger eyes, ocular ratio = 1.48–1.54, that cover most of the ocular lobe, ocular lobe ratio = 0.76–0.84, in contrast to beetles of *Mecyclothorax haydeni* with ocular ratio = 1.41–1.49, and ocular lobe ratio = 0.70–0.78. Male genitalia are diagnostic, with the aedeagal median lobe of *Mecyclothorax robustus* males exhibiting an apex with a flattened apical surface and tightly rounded, ventrally directed tip (Fig. 41), versus a median lobe with an evenly downcurved and narrowed apex for *Mecyclothorax haydeni* (Fig. 43). Setal formula 2 2 2 2. Standardized body length 4.8–6.1 mm.

##### Identification

(n = 5). In keeping with the larger eyes, the posterior portion of the ocular lobe joins the gena at nearly a right angle in this species, with a shallow groove at the juncture of lobe and gena. The pronotal median base is covered with distinct punctures, and longitudinal wrinkles line the juncture with the disc; the anterior transverse impression is deep, narrow, and crossed by deep wrinkles, its posterior surface lined with isodiametric microsculpture. The pronotum is variably transverse, MPW/PL = 1.20–1.28, with variably moderate basal constriction, MPW/BPW = 1.34–1.50. The parascutellar striole is distinctly 5-punctate, and striae 1–6 are distinctly punctate basally, and slightly irregular to smooth apically. The elytral disc is rufobrunneous, often with a purplish reflection due to the transverse-mesh to transverse-line microsculpture covering the intervals.

**Male genitalia** (n = 36). Aedeagal median lobe gracile, distance from parameral articulation to tip varies 3.5–4.8× depth at midlength (Fig. 41B, F); apex moderately extended beyond ostial opening, but always with apical face flat and tip rounded at juncture of apical face and ventral margin; median lobe slightly curved rightward in ventral view, the curvature of left margin more exaggerated in more robust aedeagi (e.g., Fig. 41C versus 41H, which is same specimen as 41F); internal sac parallel sided, dorsal and ventral microtrichial patches variably developed (Fig. 41B, E, G); flagellar plate moderately large, length of sclerotized ventral face 0.44–0.48× parameral articulation-tip distance.

**Female reproductive tract** (n = 2). Bursa copulatrix columnar with expanded apex, length 1.25–1.28 mm, breadth 0.48–0.51 mm, basal breadth 0.34 mm in one specimen (Fig. 39B), basal breadth subequal to apical breadth in second specimen; bursal walls translucent with thick wrinkles; gonocoxite 1 with 2–3 apical fringe setae and 7–10 smaller setae on medial surface (Fig. 37D); gonocoxite 2 narrowly subtriangular with subacuminate apex, base narrowly extended laterally, 2 lateral ensiform setae with apical seta broader and longer, apical nematiform setae on medioventral surface at 0.74× gonocoxite length.

##### Types.

For *Cyclothorax robustus* Blackburn, holotype female (BMNH) labeled: mounting platen with Blackburn Maui code (Zimmerman 1957: 210), Cyc robustus (on reverse) // Type // Rev. T. Blackburn 1888-30. // HOLOTYPE Cyclothorax robustus Blackburn J.K. Liebherr 1998 (black-margined red label). For *Mecyclothorax robustus* Sharp, lectotype male (BMNH) labeled: Mecyclothorax robustus D.S. Type Haleakala Perkins 622 // Type // Hawaiian Is. Perkins 1904-336 // LECTOTYPE Mecyclothorax robustus Sharp J.K. Liebherr 1998 (black-margined red label).

##### Distribution and habitat.

The distributions of *Mecyclothorax robustus* and *Mecyclothorax haydeni* subdivide the windward face of Haleakalā, with this species found from Kuhiwa Valley on the east to the leeward forest edge near Makawao on the west (Fig. 42) at elevations spanning 1137–1830 m. Historical Perkins records can be predominantly assigned to Ukulele Camp (Perkins 1894, 1896a, b). The species is distributed in ‘Ōhi‘a and Koa Forest formations, though most direct associations involve beetles being found in moss on ‘ōhi‘a, and secondarily on hāpu‘u (*Cibotium*) or on ‘ōhi‘a nurse logs. This is an abundant species within its habitats. Beetles can be beaten from vegetation at night, or found under boards or logs either during day or at night.

#### (026) Mecyclothorax haydeni sp. n.

Taxon classificationAnimaliaColeopteraCarabidae

http://zoobank.org/03C7D321-9E41-49D0-A968-70BA883E82E8

Figs 37E , 38E , 39C , 42 , 43

##### Diagnosis.

Larger bodied beetles within the species group, standardized body length = 5.0–6.2 mm, with broad, subquadrate elytra. The discal elytral striae are lined with small though distinct punctures that at most expand strial breadth, or at least introduce irregularities to the strial orientation (Fig. 38E). The eyes are smaller than those observed in *Mecyclothorax robustus* individuals (see diagnosis above), though the male genitalic configuration is the only certain means to diagnose the two species. The male median lobe of *Mecyclothorax haydeni* males exhibits an evenly downcurved apex (Fig. 43), though the degree of narrowing toward the tip and extension beyond the ostial opening varies among individuals (Fig. 43B versus 43D). The male aedeagal internal sac is shorter and narrower in males of *Mecyclothorax haydeni* (Fig. 43B, F, H) than in males of *Mecyclothorax robustus* (Fig. 41B, E, G). Setal formula 2 2 2 2.

##### Description

(n = 5). *Head capsule* with frontal grooves broad near clypeus, with broad lateral carina to anterior supraorbital seta; dorsal surface of neck flat to slightly concave; labral anterior margin subangulate, medially excavated 0.1× length; antennae robustly filiform, antennomeres 2–3 with sparse pelage of short setae; mentum tooth with sides acute, apex tightly rounded. *Pronotum* slightly transverse, MPW/PL = 1.20–1.24, variably constricted basally, MPW/BPW = 1.37–1.50; hind angle slightly obtuse, basal margin rounded posterad angle, lateral margin slightly divergent anterad hind angle; median base depressed relative to disc, covered with dense punctures and wrinkles, surface strigose; basal margin broadly, moderately convex between laterobasal depressions; median longitudinal impression very shallow, finely incised; anterior transverse impression broad, shallow, crossed by wrinkles and lined by granulate isodiametric microsculpture; anterior callosity slightly elevated, crossed by dense longitudinal wrinkles, strigose; front angles slightly projected, rounded; pronotum broader basally than apically, APW/BPW = 0.90–0.95; lateral marginal depression only slightly broader at front angle, moderately narrow, edge upturned; laterobasal depression broad, smooth, with median tubercle. *Proepisternum* with 6 indistinct punctures along hind marginal groove; prosternal process medially depressed, with broad lateral marginal bead. *Elytra* subquadrate, disc convex, sides more sloped; basal groove recurved to subangulate humerus, the angle defined by hitch in groove at juncture with lateral marginal depression, MEW/HuW = 1.81–2.08; parascutellar seta present; parascutellar striole continuous, with 5 small punctures or irregularities along length; sutural interval more convex than lateral intervals, sutural juncture upraised; sutural and 2^nd^ striae of subequal depth from base to apex; discal striae minutely punctured in basal 1/3, smooth and deep apically, associated intervals convex; 7^th^ and 8^th^ intervals of similar convexity mesad subapical sinuation; 2 dorsal elytral setae at 0.31× and 0.63× elytral length, setal impressions small, extended over ½ width of interval 3; apical and subapical setae present; lateral elytral setae arranged as anterior series of 7 setae and posterior series of 6 setae; elytral marginal depression moderately narrow throughout length, flat bottomed until subapical sinuation; subapical sinuation evident, moderately deep. *Mesepisternum* with ~ 14 punctures in two rows; metepisternal width to length ratio = 0.79; metepisternum/metepimeron suture distinct. *Abdomen* with irregular lateral wrinkles on ventrites 1–5; suture between ventrites 2 and 3 complete; apical male ventrite with 2 marginal setae, and apical female ventrite with 4 equally spaced setae and a median trapezoid of 4 subequal, short setae. *Legs*-metatarsomere 1/metatibial length ratio = 0.19; metatarsomere 4 length along outer lobe 1.4× medial tarsomere length, apical and subapical setae present; metatarsal dorsolateral sulci deep, broad, median area medially strigose. *Microsculpture* of vertex an isodiametric mesh; pronotal disc with transverse mesh, sculpticell breadth 2–3× length; pronotal median base with distinct to granulate isodiametric mesh; elytral disc and apex with transverse mesh, sculpticell breadth 3× length, to transverse lines unconnected into a mesh, the apex with more transverse lines; metasternum with distinct transverse mesh; laterobasal abdominal ventrites swirling isodiametric and transverse microsculpture. *Coloration* of vertex rufobrunneous; antennomere 1 flavous, 2–3 rufoflavous, 4–11 rufobrunneous; pronotal disc rufobrunneous, margins and apex slightly paler, base concolorous; proepipleuron rufoflavous, proepisternum rufobrunneous; elytral disc rufobrunneous; sutural interval rufous basally, rufoflavous apically; elytral marginal depression slightly paler, elytral apex concolorous with disc; elytral epipleuron rufoflavous, metepisternum rufobrunneous; abdomen with ventrites 1–6 medially rufobrunneous, laterally rufoflavous; apical abdominal ventrite 6 with apical half paler, flavous; metafemur flavous; metatibia rufoflavous.

**Male genitalia** (n = 26). Aedeagal median lobe slender, elongate, distance from parameral articulation to tip 4.1–5.5× depth at midlength (Fig. 43E, H), but apical extension always smoothly curved relative to median lobe shaft, the apex evenly narrowed to a rounded tip; median lobe broadly curved rightward, the apex blunt in ventral view (Fig. 43C, G); internal sac of variable length (Fig. 43B, F, H), with consistent presence of two ventral microtrichial patches—basal and apical as in *Mecyclothorax antaeus* (Fig. 34J)—the apical patch of variable size; flagellar plate relatively small, length of sclerotized ventral face 0.33–0.36× parameral articulation-tip distance.

**Female reproductive tract** (n = 1). Bursa copulatrix columnar with narrow, rounded apex, length 1.17 mm, apical breadth 0.31 mm, basal breadth 0.47 mm subequal to vagina breadth (Fig. 39C); bursal walls translucent with thin wrinkles basally, apical surface shagreened but not wrinkled; gonocoxite 1 with 2–4 apical fringe setae, a moderately sized seta just basad medioapical angle and 7–8 small setae on medial surface (Fig. 37E); gonocoxite 2 narrowly subtriangular with broad apex and tightly rounded tip, 2 lateral ensiform setae, apical nematiform setae on medioventral surface at 0.78× gonocoxite length.

##### Holotype.

Female (BPBM) labeled: Kipahulu Valley / Maui Camp 2 / 1250 m, 13-17.VIII.67 // N. Wilson / Collector / BISHOP // ? cognatus (E.C.Z. handwriting) // HOLOTYPE / Mecyclothorax / haydeni / Liebherr / J.K. Liebherr 2015 (black-margined red label).

##### Paratypes.

176 specimens (see Appendix).

##### Etymology.

This species is named to honor the contributions of Dr. James E. Hayden, Jr. to this project, wherein he dissected numerous male specimens of Hawaiian *Mecyclothorax* in order to delineate species boundaries.

##### Distribution and habitat.

*Mecyclothorax haydeni* occupies ‘Ōhi‘a Forest formations from 1127–2145 m elevation on the wetter, eastern end of the Haleakalā windward forest (Fig. 42). Beetles may be found in leaf litter via sifting, or they may be observed actively running on tree trunks and ferns at night. To date, equivalent samples derived from leaf litter sampling or pyrethrin fogging at 1500–1960 m in Kīpahulu Valley include many more individuals of this species than do samples from 1200 m elevation.

### *Mecyclothorax interruptus* species group

**Figure 44.**
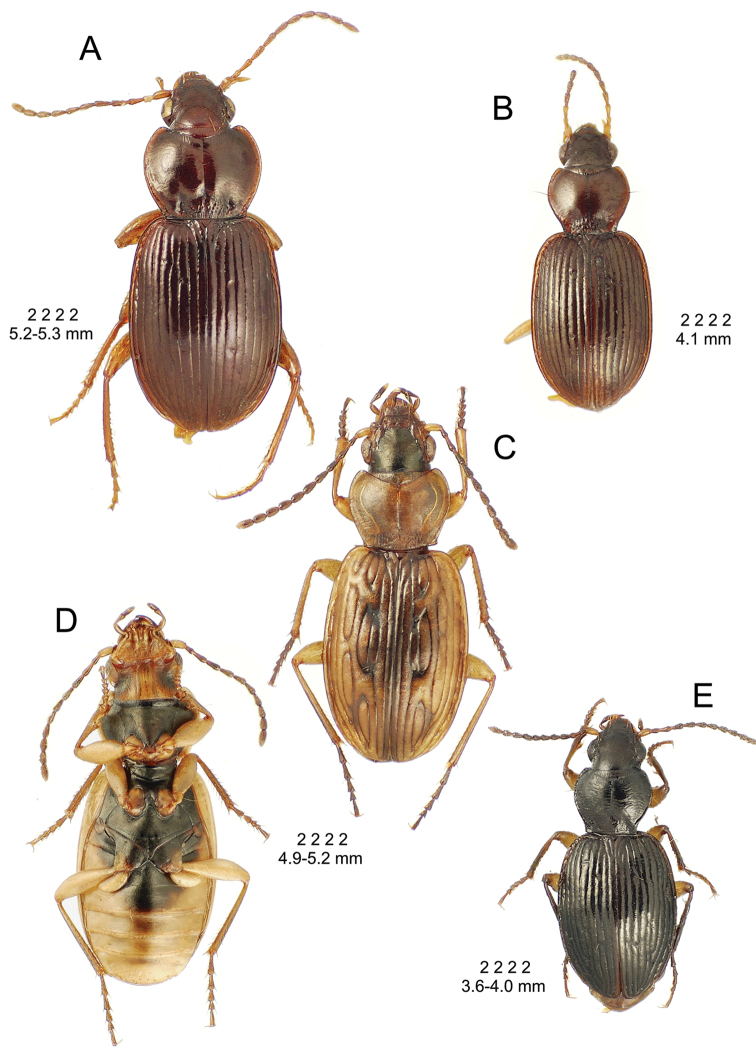
*Mecyclothorax interruptus* group species, dorsal habitus view. **A** *Mecyclothorax integer* (Olinda, 1210 m) **B** *Mecyclothorax bradycelloides* (Ukulele Camp Pipeline, 1465–1495 m) **C–D** *Mecyclothorax irregularis* (Polipoli, 1738 m) **C** Dorsal view **D** Ventral view **E** *Mecyclothorax anthracinus* (Kuhiwa, 2070-2100 m).

**Diagnosis.** Specimens representing this species group can be diagnosed by the fully developed, impunctate elytral striae from suture to lateral margin, with adjacent striae at least connected by the depressions surrounding the dorsal elytral setae—*Mecyclothorax bradycelloides* (Fig. 44B)—or more tortuously distorted and variously anastomosed along their length (Figs 44A, C, E, 49A–C). Setal formulae for the Haleakalā species vary, including 2 2 2 2, 2 1(2) 2 2, 2 1 3 2, or the infraspecifically variable formula of 2 1 2 (0-1(2)) in *Mecyclothorax arthuri*.

**Membership and distribution.** This group is restricted to Maui Nui, with four species distributed in West Moloka‘i (Liebherr 2007), and two in West Maui (Liebherr 2011) complementing the nine Haleakalā species. Liebherr (2007), following Britton (1948b), placed *Mecyclothorax perkinsi* (Sharp) in the *Mecyclothorax constrictus* species group based on the basally constricted prothorax with glabrous hind angles (setal formula 2 1 2-3 1[ae]). Male *Mecyclothorax perkinsi* exhibit a unique accessory apical aedeagal projection (Liebherr 2007, fig. 95) not seen in any other *Mecyclothorax* species, though male *Mecyclothorax constrictus* (Sharp) exhibit an anatomically similar accessory subapical aedeagal projection (Liebherr 2007, fig. 97). Nevertheless, *Mecyclothorax perkinsi* exhibits tortuously anastomosing elytral striae as seen in species of this group; the only other Hawaiian species not placed in the *Mecyclothorax interruptus* group to exhibit this condition (Liebherr 2007, fig. 30). These conflicting character-based data suggest that current placement of this species should be revisited pending additional information.

#### Key to adults of the *Mecyclothorax interruptus* species group, Haleakalā volcano, Maui, Hawai‘i

**Table:** 

1	Adjacent elytral striae not conjoined—though they may approach (Fig. 44A)—except in close association with a dorsal elytral seta, in which instance the impression surrounding seta will connect striae 2 and 3 (Fig. 44B)	**2**
1’	Adjacent elytral striae variously conjoined along their length not in close association with a dorsal elytral seta, these fusions variously involving striae 1 and 2, 3 and 4, or 5 and 6 (Figs 44C, E, 49, 50)	**3**
2(1)	Pronotum broad, MEW/MPW = 1.30–1.32 (Fig. 44A); standardized body length = 5.2–5.3 mm	(027) ***Mecyclothorax integer* Sharp**
2’	Pronotum narrow, MEW/MPW = 1.47 (Fig. 44B); standardized body length = 4.1 mm	(028) ***Mecyclothorax bradycelloides* sp. n.**
3(1)	Vertex, pronotum, elytra and ventral surface concolorous, brunneous to piceous, legs paler, femora pale brunneous to flavous at midlength (Figs 44E, 49, 50)	**4**
3’	Vertex iridescent piceous, contrasted to flavous pronotum and elytral disc, though elytra with melanic areas associated with strial fusions (Fig. 44C), thoracic ventrites and mediobasal area of abdominal venter piceous versus flavous abdominal apex (Fig. 44D)	(029) ***Mecyclothorax irregularis* Britton**
4(3)	Body broader, brunneous to dark brunneous, legs paler, femora may be darker but not markedly so (Figs 49, 50); striae 3 and 4 irregularly fused near dorsal elytral setae, striae 5 and 6 may also be fused	**5**
4’	Body narrow, piceous, legs paler, base color flavous though tibiae distinctly, contrastedly piceous (Fig. 44E); elytral striation unstable, bases of striae 5 and 6 often fused or approaching resulting in varying convexity of associated intervals, otherwise striae generally independent along length	(030) ***Mecyclothorax anthracinus* sp. n.**
5(4)	Elytral striae intensely anastomosed, bulbous calli asymmetrically formed from fusions of all striae (Fig. 49A–C)	**6**
5’	Fusion of elytral striae limited to anastomosed striae 3 and 4 near dorsal elytral setae, and possibly striae 5 and 6 basally	**7**
6(5)	Elytra narrower relative to pronotal width, MEW/MPW = 1.47 (Figs 49A–B); pronotal disc with granulate isodiametric microsculpture, deep lateral plaquelike depressions complement deep median longitudinal impression	(031) ***Mecyclothorax arthuri* sp. n.**
6’	Elytra broader relative to pronotal width, MEW/MPW = 1.63–1.67 (Fig. 49C); pronotal disc with shallow transverse-mesh microsculpture, surface glossy	(032) ***Mecyclothorax medeirosi* sp. n.**
7(5)	Pronotum broad, MPW/BPW = 1.35–1.47, maximum elytral width: maximum pronotal width = 1.34–1.45; standardized body length 4.8–5.9 mm	**8**
7’	Pronotum narrower, more constricted basally, MPW/BPW = 1.49–1.53, MEW/MPW = 1.45–1.49; standardized body length 3.7–4.0 mm	(033) ***Mecyclothorax inconscriptus* sp. n.**
8(7)	Pronotal disc with distinct isodiametric sculpticells arranged in transverse rows, surface coriaceous; male aedeagal median lobe with acuminate dorsal expansion (Fig. 45M–N)	(034) ***Mecyclothorax foveolatus* sp. n.**
8’	Pronotal disc with swirling transverse-mesh microsculpture, sculpticell breadth 2–3× length; male aedeagal median lobe apex with bluntly rounded dorsal expansion (Fig. 45O–P)	(035) ***Mecyclothorax interruptus* Sharp**

#### (027) Mecyclothorax integer

Taxon classificationAnimaliaColeopteraCarabidae

Sharp stat. n.

Figs 44A , 45A , 48

Mecyclothorax interruptus var. integer Sharp 1903: 252; Britton 1948b: 163.

##### Diagnosis.

This is the broadest-bodied species in the Haleakalā *Mecyclothorax* fauna, exhibiting a large prothorax and broadly subquadrate elytra, MEW/MPW = 1.30–1.32 (Fig. 44A). The pronotal lateral margins are only slightly sinuate anterad the obtuse-rounded hind angles; and the linear laterobasal depression is separated from the pronotal lateral margin by a broad tubercle. The elytral striae are nearly regular, though striae 2 and 3 may approach unilaterally. The dorsal elytral setal impressions are foveate anteriorly, crossing interval 3 at the anterior seta, and smaller posteriorly, impressing half the interval width at the posterior seta. The dorsal body coloration is uniformly rufobrunneous, with the legs paler, rufoflavous. Setal formula 2 2 2 2. Standardized body length 5.2–5.3 mm.

##### Identification

(n = 3). The eyes are moderately convex, ocular ratio = 1.43–1.46, but cover much of the ocular lobe, ocular lobe ratio = 0.80–0.84. The pronotum is large, MPW/PL = 1.28–1.31, and modestly constricted basally, MPW/BPW = 1.36–1.40. The elytral disc is flat medially, sides moderately sloped, and the subangulate humeri are defined by juncture of the slightly recurved basal groove and lateral marginal depression. The discal elytral intervals, including the sutural interval, are moderately convex. The vertex is covered with isodiametric microsculpture, the sculpticells transversely stretched on the neck, pronotal disc with distinct transverse mesh, the base glossy in parts with an elongate transverse mesh; elytral disc covered with elongate transverse mesh, a loose transverse mesh and parallel lines on the elytral apex; metasternum covered with an obsolete transverse mesh.

**Figure 45.**
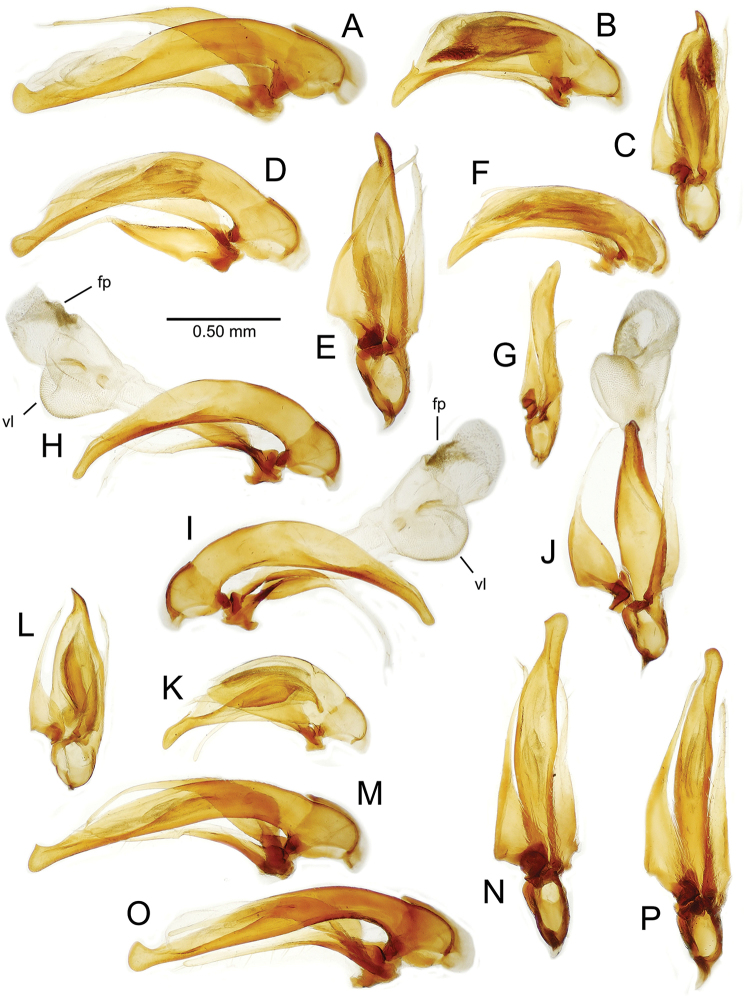
Male aedeagus, *Mecyclothorax interruptus* group species (for abbreviations see Table 2, p. 23). **A** *Mecyclothorax integer*, right view (Olinda, 1210 m). **B–C** *Mecyclothorax bradycelloides*, right and ventral views (Ukulele Camp Pipeline, 1465-1495 m) **D–E** *Mecyclothorax irregularis*, right and ventral views (Polipoli, 1738 m) **F–G** *Mecyclothorax anthracinus*, right and ventral views (Kuhiwa, 2070-2100 m) **H–J** *Mecyclothorax arthuri*, Right, left, and ventral views, sac everted (ESE Kuiki, 2145 m) **K–L** *Mecyclothorax inconscriptus*, right and ventral views (Kaupō Gap, 1495 m) **M–N** *Mecyclothorax foveolatus*, right and ventral views (Kuhiwa, 880 m) **O–P** *Mecyclothorax interruptus*, right and ventral views (Olinda, 1210–1365 m).

**Male genitalia** (n = 1). Aedeagal median lobe slender, distance from parameral articulation to tip 5.6× depth at midlength; shaft slightly curved basally, straight to slightly recurved apically, the apex broadly rounded with blunt dorsal projection (Fig. 45A); internal sac without evident ornamentation.

##### Lectotype.

Male (BPBM) hereby designated, dissected and labeled: 622 (on reverse of mounting platen) // Haleakala / Maui 6000 ft. / Perkins V 1896 // Mecyclothorax interruptus / var. integer Sharp / [number OK but date / on label possibly wrong → as Oct. 1896 in orig. / description / SYNTYPE / G/A. Samuelson det. 196 // LECTOTYPE ♂ / Mecyclothorax / interruptus var. / integer Sharp / J.K. Liebherr 2011 (black-margined red label).

##### Paralectotype.

Female (BPBM) labeled: Mecyclothorax interruptus / var. integer / Haleakala / Perkins (on obverse of mounting platen), 680. (on reverse of mounting platen) // Hawaiian Is. / R.C.L. Perkins // Sharp Coll / 1905-313. // PARALECTOTYPE ♀ / (same labelling as Lectotype).

##### Distribution.

*Mecyclothorax integer* is known only from the lectotype and paralectotype collected by Perkins in his lots 622 and 680; “Haleakala 4000 ft., v-1896”, and “Haleakala 4000+ ft., x-1896” (Anonymous N D), respectively (Fig. 48). Of the October visit, Perkins (1896b) wrote: “In October 1896 I camped for a considerable time at about 5,000 ft. on Haleakala and did a good deal of work at various points Eastward in the windward forest entering this at various points from the upper edge towards the forest that lies above Wailua (p. 1).” This locality would lie about 200 ft. elevation below the Ukulele Camp (site) of the USGS (1983) Kilohana, Hawaii quadrangle. Recent collections near this site did not result in rediscovery of this species.

#### (028) Mecyclothorax bradycelloides sp. n.

Taxon classificationAnimaliaColeopteraCarabidae

http://zoobank.org/D1181A92-D4AD-4B9B-8E1F-3D941DB398C0

Figs 44B , 45B–C , 48

##### Diagnosis.

This species (Fig. 44B), *Mecyclothorax anthracinus* (Fig. 44E), and *Mecyclothorax inconscriptus* (Fig. 50A) represent the three smallest-bodied species in this group, with all individuals equal to or less than 4.1 mm length. Of these, *Mecyclothorax bradycelloides* is the only species without fused elytral striae, though the impressions of the dorsal elytral setae are large, crossing most of the width of interval 3. The pronotum is also the most constricted basally, with MPW/BPW = 1.56 versus a collective span of 1.42–1.53 for the other two species. The elytral margins are straight and nearly parallel at elytral midlength, though the tightly rounded humeral angles are narrowly separated relative to elytral width due to the broadly rounded elytral margins laterad the angles; MEW/HuW = 2.0. Setal formula 2 2 2 2. Standardized body length 4.1 mm.

##### Description

(n = 1). *Head capsule* with frontal grooves deep and broad near clypeus, narrowed toward anterior supraorbital seta; dorsal surface of neck slightly convex; ocular ratio = 1.49, ocular lobe ratio = 0.81; antennae filiform, antennomere 3 with sparse pelage of short setae; mentum tooth with sides acute, apex tightly rounded. *Pronotum* cordate, hind angle obtuse, rounded behind; lateral margin sinuate for short distance anterad hind angle; median base covered with punctures and wrinkles isolated by granulate microsculpture; basal margin nearly straight, slightly convex medially; median longitudinal impression shallow, finely incised; anterior transverse impression deeply incised, complete, crossed by longitudinal wrinkles; anterior callosity broadly, slightly convex; front angles projected, tightly rounded, APW/BPW = 1.07; lateral marginal depression narrow, edge upturned to beaded; laterobasal depression broad, irregular, slightly convex medially. *Proepisternum* with smooth hind marginal groove; prosternal process medially depressed, with lateral marginal bead. *Elytra* subquadrate, disc slightly convex; parascutellar seta present on left side, absent on right; parascutellar striole shallow with 6 punctures; sutural interval moderately convex, appearing broader than intervals 2–4 due to elevated juncture at suture; sutural and 2^nd^ striae of subequal depth from base to apex; discal striae with small punctures that cause strial irregularities along length, intervals convex; 7^th^ and 8^th^ intervals of similar convexity mesad subapical sinuation; 2 dorsal elytral setae at 0.23–0.27× and 0.60–0.63× elytral length; apical and subapical setae present; lateral elytral setae arranged as anterior series of 7 setae, and posterior series of 4–5 setae; elytral marginal depression moderately broad, lined with sculpticells, margin upturned; subapical sinuation very shallow, broad. *Mesepisternum* with ~9 shallow punctures in 1–2 rows; metepisternal width to length ratio = 0.81; metepisternum/metepimeron suture distinct. *Abdomen* with nearly smooth ventrites; suture between ventrites 2 and 3 complete; apical male ventrite with 2 marginal setae. *Legs*-metatarsomere 1/metatibial length ratio = 0.17; metatarsomere 4 length along outer lobe 1.2× medial tarsomere length, apical and subapical setae present; metatarsal dorsolateral sulci broad, deep, median area carinate. *Microsculpture* of vertex isodiametric sculpticells arranged in transverse rows; pronotal disc with transverse mesh, the microsculpture parallel in part; pronotal median base with granulate isodiametric sculpticells; elytral disc with irregular elongate transverse mesh and parallel lines; elytral apex with upraised transverse mesh; metasternum with indistinct transverse mesh; laterobasal abdominal ventrites with indistinct transverse mesh. *Coloration* of vertex rufobrunneous; antennomeres 1–3 flavous, 4–11 rufobrunneous; pronotal disc rufobrunneous, margins narrowly paler, rufoflavous; proepipleuron flavous, proepisternum brunneous; elytral disc rufobrunneous, sutural interval concolorous basally, rufoflavous apically, margins narrowly rufoflavous in lateral depression, apex broadly flavous; elytral epipleuron flavous with rufous cast, metepisternum rufobrunneous; abdominal ventrites 1–5 rufobrunneous, 6 basally rufoflavous, flavous in apical half; metafemur flavous; metatibia flavous with brunneous cast.

**Male genitalia** (n = 1). Aedeagal median lobe robust, distance from parameral articulation to tip 3× depth at midlength (Fig. 45B); apex narrowly extended twice its depth beyond ostial opening, tip subangulate where flattened apical face and ventral margin meet; median lobe straight in ventral view, the left margin distinctly incurved to meet blunt tip (Fig. 45C); internal sac with well-developed, heavily sclerotized dorsal ostial microtrichial patch (based on position near apex of ostium (Fig. 45B), and separate ventral ostial microtrichial patch (Fig. 45C); flagellar plate well sclerotized, visible just inside dorsal margin of lobe (Fig. 45B).

##### Holotype.

Male (NMNH) dissected and labeled: HI:Maui Haleakala / Waikamoi N.C.P. Ukulele / Pipeline 7-V-1998 lot07 / 1465-1495m el. / pyrethrum fog mossy ohia / log D.A. Polhemus // HOLOTYPE / Mecyclothorax / bradycelloides / Liebherr / det. J.K. Liebherr 2015 (black-margined red label).

##### Etymology.

The adjectival epithet bradycelloides is based on Moloka‘i’s *Mecyclothorax bradycellinus* Sharp, with the -oides suffix signifying the similarity between the two species.

##### Distribution and habitat.

The type locality for *Mecyclothorax bradycelloides* lies at ~1500 m elevation near Ukulele Camp (Site) of the USGS (1983) Kilohana, Hawaii quadrangle (Fig. 48). This species was found in a pyrethrin fog sample of a mossy ‘ōhi‘a log.

#### (029) Mecyclothorax irregularis

Taxon classificationAnimaliaColeopteraCarabidae

Britton

**Figure 46.**
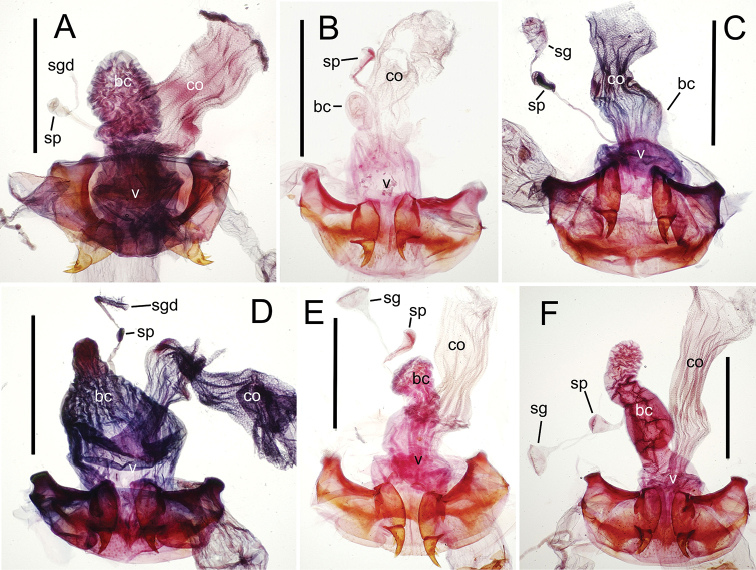
Female bursa copulatrix and associated reproductive structures, *Mecyclothorax interruptus* group species, ventral view (for abbreviations see Table 2, p. 23). **A** *Mecyclothorax irregularis* (Polipoli, 1738 m) **B** *Mecyclothorax anthracinus* (Kuhiwa, 2070–2100 m) **C** *Mecyclothorax arthuri* (ESE Kuiki, 2145 m) **D** *Mecyclothorax inconscriptus* (Kaupō Gap, 1495 m). **E** *Mecyclothorax foveolatus* (Kopili‘ula, 1127 m) **F** *Mecyclothorax interruptus* (Waikamoi, 1310 m). Scale bar = 0.50 mm.

**Figure 47.**
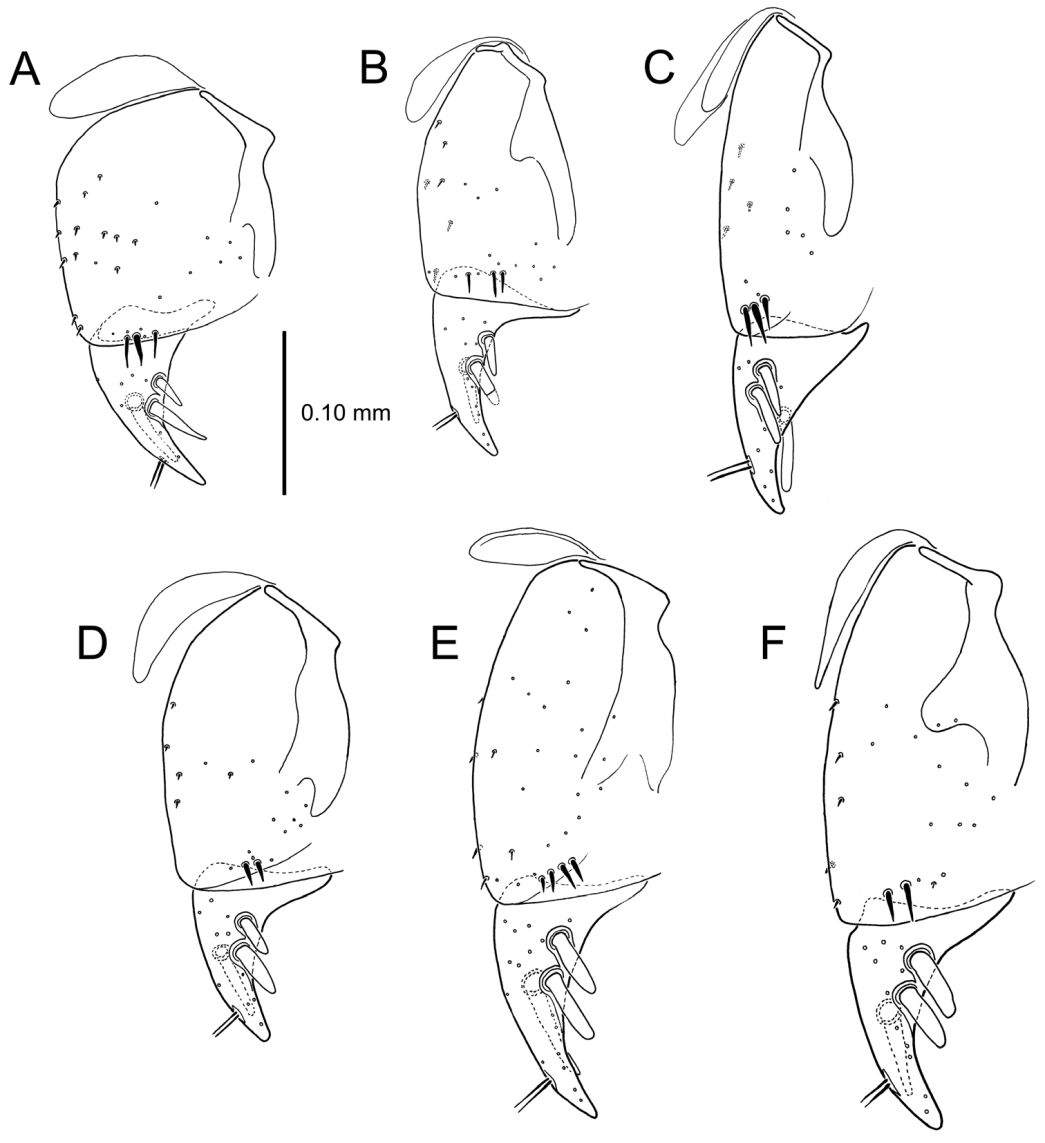
Left female gonocoxa, *Mecyclothorax interruptus* group species, ventral view. **A** *Mecyclothorax irregularis* (Polipoli, 1738 m) **B** *Mecyclothorax anthracinus* (Kuhiwa, 2070–2100 m) **C** *Mecyclothorax arthuri* (ESE Kuiki, 2145 m) **D** *Mecyclothorax inconscriptus* (Kaupō Gap, 1495 m) **E** *Mecyclothorax foveolatus* (Kopili‘ula, 1127 m) **F** *Mecyclothorax interruptus* (Waikamoi, 1310 m).

**Figure 48.**
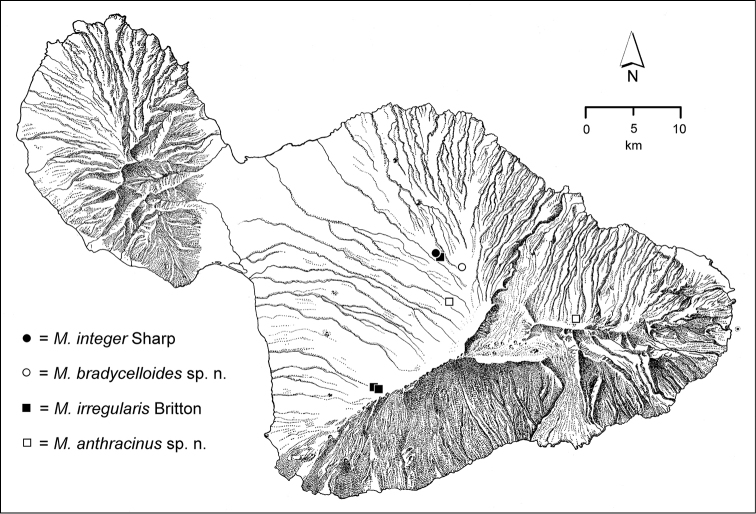
Recorded geographic distributions of *Mecyclothorax interruptus* group species.

Figs 44C–D , 45D–E , 46A , 47A , 48

Cyclothorax multipunctatus , Blackburn 1878a: 122 (misidentification).
Mecyclothorax irregularis Britton 1948b: 161; Liebherr 2005b, 122.

##### Diagnosis.

The irregularly anastomosed elytral striae 2–6, combined with the bicolored dorsal surface—piceous head, testaceous pronotum, plus testaceous elytral convexities versus piceous striae (Fig. 44C)—uniquely diagnose this species within the Hawaiian *Mecyclothorax* fauna. The ventral body coloration is equally distinctive, with the piceous genae, thoracic ventrites, metacoxae, and mediobasal portions of the basal abdominal ventrites distinctly contrasted to the pale mentum and gula, pronotal and elytral epipleura, and abdominal apex (Fig. 44D). Setal formula 2 2 2 2. Standardized body length 4.7–5.2 mm.

##### Identification

(n = 5). The head is broader relative to the hindbody in this species, MEW/MHW = 1.78–1.90, than in any other species of the group. The broad head is based on a broad head capsule as the eyes are small, ocular lobe ratio = 0.70–0.81, and not very convex, ocular ratio = 1.30–1.39. The pronotal median base is smooth, with the small punctures isolated by areas of granulate isodiametric microsculpture. The laterobasal pronotal depressions are also smooth, shallow, with a broad median tubercle. Microsculpture across the body is well developed, with: 1, vertex, and pronotal disc and base with granulate isodiametric sculpticells, the surface appearing dull; 2, elytral disc and apex with isodiametric sculpticells; 3, metasternum covered with an upraised transverse mesh; and 4, basal abdominal ventrites with lateral areas covered with swirling isodiametric and transverse sculpticells.

**Male genitalia** (n = 1). Aedeagal median lobe slender, distance from parameral articulation to tip 4.8× depth at midlength (Fig. 45D); shaft curved basally, nearly straight apically, apex slightly expanded dorsoventrally with rounded tip; median lobe straight in ventral view, left margin distinct incurved to blunt apex, right margin angled leftward to tip (Fig. 45E); internal sac without distinct ornamentation, but surface covered with melanic spicules, short flagellar plate evident in repose, length 0.24× parameral articulation-tip distance.

**Female reproductive tract** (n = 1). Bursa copulatrix columnar with rounded apex, length 0.68 mm, breadth 0.23 mm (Fig. 46A); bursal walls translucent, densely wrinkled; gonocoxite 1 with 3 apical fringe setae, and 6–7 smaller setae on medial surface (Fig. 47A); gonocoxite 2 subtriangular, apex acuminate, base evenly extended laterally from lateral margin, 2 lateral ensiform setae with the apical seta longer and broader, apical nematiform setae on medial surface at 0.73× gonocoxite length.

##### Holotype.

Female (BMNH) labeled: mounting platen with Blackburn Maui code (Zimmerman 1957: 210), multip (on reverse) // Type // Hawaiian Is. Rev. T. Blackburn 1883-30 // Cyclothorax irregularis sp. n. E.B. Britton det. 1939.

##### Distribution and habitat.

This species was described by Britton (1948b) from a single Blackburn specimen originally considered by Blackburn to represent *Mecyclothorax multipunctatus*. The type locality for *Mecyclothorax multipunctatus* was listed as “Haleakala, 4000 ft.”; i.e. near Olinda. The only modern records derive from the ecologically disjunct forest near Polipoli Springs on Haleakalā’s southwest rift (Fig. 48). Microhabitats at Polipoli where this species has been collected include *Dryopteris wallichiana* (laukahi) fern litter, deep leaf litter at the bottom of a small rocky face, and the mossy surface of a small, downed *Pinus radiata* trunk lying in deep leaf litter at the bottom of a shallow ravine.

#### (030) Mecyclothorax anthracinus sp. n.

Taxon classificationAnimaliaColeopteraCarabidae

http://zoobank.org/C9A8CE93-6268-45EE-8084-97F101E823A7

Figs 44E , 45F–G , 46B , 47B , 48

##### Diagnosis.

The small, dark-bodied beetles that comprise this species look ever so like small bits of anthracite coal, their dorsal body surface a reflective black (Fig. 44E). Also, this is the only species in the group that is characterized by absence of the parascutellar seta. The pronotum is narrow and basally constricted, MPW/PL = 1.18–1.24, MPW/BPW = 1.42–1.50, the disc covered with well-developed transverse wrinkles. The elytral intervals are convex and striation irregular, with striae 5 and 6 fused near the basal groove, and the dorsal setal impressions foveate and of diameter equal to the width of interval 3, these impressions associated with longitudinal irregularities of striae 2 and 3. The femora are flavous apically and covered with a piceous cast over their basal third; tibiae piceous. Setal formula 2 2 2 2. Standardized body length 3.6–4.0.

##### Description

(n = 5). *Head capsule* with frontal grooves deep near clypeus, straight with external carina to anterior supraorbital seta; dorsal surface of neck convex, eyes moderately convex, ocular ratio = 1.42–1.50, ocular lobe ratio 0.77–0.85; labral anterior margin broadly, shallowly emarginate, antennae filiform, antennomere 3 with sparse pelage of short setae; mentum tooth with orthogonal sides, apex pointed. *Pronotum* with lateral margin subparallel to convergent anterad right to acute hind angle; median base depressed relative to disc, covered with rugose wrinkles; basal margin nearly straight, slightly convex medially; median longitudinal impression shallow, finely incised, crossed by wrinkles; anterior transverse impression broad, evident, bordered anteriorly by slightly convex anterior callosity that is crossed by fine wrinkles; front angles slightly projected, rounded; anterior width subequal to broader than basal width, APW/BPW = 1.00–1.06; lateral marginal depression moderately narrow, edge upturned to beaded; laterobasal depression broad, depressed with wrinkled surface. *Proepisternum* with 6 minute punctures along hind marginal groove; prosternal process medially depressed, without marginal bead. *Elytra* subellipsoid, disc flat, sides moderately sloped; basal groove slightly recurved to tightly rounded humeral angle; humeri narrow, MEW/HuW = 2.06–2.10; parascutellar striole finely incised, continuous; sutural interval moderately convex, slightly more upraised than intervals 2–4; sutural and 2^nd^ striae of subequal depth from base to apex; discal striae 1–8 complete and deep to apex, smooth with minute irregularities along striae suggesting punctulae; 7^th^ and 8^th^ intervals of similar convexity mesad subapical sinuation; 2 dorsal elytral setae at 0.32× and 0.65–0.73× elytral length; apical and subapical setae present; lateral elytral setae arranged in anterior series of 7 setae and posterior series of 4(5) setae; elytral marginal depression narrow, edge upturned; subapical sinuation very shallow, nearly obsolete. *Mesepisternum* with 8 shallow punctures in 2 rows; metepisternal width to length ratio = 0.71; metepisternum/metepimeron suture distinct, metathoracic flight wing an ovoid flap, length 2.5× breadth, with reduced R and M veins, the flap extended 2/3 distance to hind margin of metanotum. *Abdomen* with indistinct lateral wrinkles on ventrites 1–3; suture between ventrites 2 and 3 complete; apical male ventrite with 2 marginal setae, apical female ventrite with 4 equally spaced marginal setae and a median trapezoid of 4, subequal short setae. *Legs*-metatarsomere 1/metatibial length ratio = 0.17; metatarsomere 4 length along outer lobe 1.2× medial tarsomere length, apical and subapical setae present; metatarsal dorsolateral sulci broad, shallow. *Microsculpture* of vertex of granulate isodiametric sculpticells; pronotal disc covered with distinct transverse mesh, median base with granulate isodiametric sculpticells; elytral disc covered with well-developed transverse mesh, apex with well-developed isodiametric mesh in transverse rows; metasternum with upraised transverse mesh; laterobasal abdominal ventrites with swirling isodiametric and transverse microsculpture. *Coloration* of vertex granulate rufopiceous; antennomere 1 flavous, antennomeres 2–3 with piceous cast, 4–11 piceous; pronotal disc granulate rufopiceous, margins narrowly paler, rufobrunneous; proepipleuron rufobrunneous with piceous upper margin, proepisternum rufobrunneous with piceous cast; elytral disc rufopiceous, sutural interval paler, dark rufous throughout, margins narrowly paler basally, concolorous with disc apically; elytral epipleuron rufobrunneous, metepisternum rufopiceous; abdomen rufopiceous across width of ventrites 1–5, apical ventrite 6 with apical 1/3 paler, rufobrunneous.

**Male genitalia** (n = 1). Aedeagal median lobe curved, gracile, distance from parameral articulation to tip 4× depth at midlength (Fig. 45F); apex angularly narrowed to tightly rounded tip formed at juncture of flat apical face and ventral margin; median lobe sinuously recurved left then right in ventral view (Fig. 45G), tip tightly rounded; internal sac with apparent dorsal ostial microtrichial patch (based on uneverted specimen; Fig. 45F), sac surface covered with microspicules; flagellar plate evident just inside dorsal margin of median lobe.

**Female reproductive tract** (n = 1). Bursa copulatrix a narrow digitiform lobe attached to broader vagina, lobe length 0.26 mm, lobe apical breadth 0.10 mm, vagina breadth 0.25 mm (Fig. 46B); bursal walls thin, transparent; gonocoxite 1 with 3 apical fringe setae and 5–6 smaller setae on medial surface (Fig. 47B); gonocoxite 2 falcate, narrow apically with base broadly extended laterally, 2 lateral ensiform setae with apical seta broader and longer, apical nematiform setae on medial surface at 0.75× gonocoxite length.

##### Holotype.

Female (BPBM) labeled: NW 6000´-6500', / Haleakala / VIII-18-37 Maui // Beating // ECZimmerman / Collector // 3 // HOLOTYPE / Mecyclothorax / anthracinus / Liebherr / det. J.K. Liebherr 2015 (black-margined red label).

##### Paratypes.

HI: Maui: Haleakala N.P., NW upper slope, beating 1830–1980 m el., 18-viii-1937, Zimmerman (BPBM, 2); Koolau For. Res., Hanawi N.A.R., Frisbee Meadow Camp, woods below, sift litter *Dubautia*/tree, 2072–2099 m el., 19-v-1993 lot 01, Liebherr/Medeiros (CUIC, 6).

##### Etymology.

The shiny coal black color of the dorsal surface of these beetles begs for use of the Latin adjective anthracinus.

##### Distribution and habitat.

*Mecyclothorax anthracinus* is known from two isolated, high-elevation localities near the upper limits of the windward forest. E.C. Zimmerman beat three specimens from vegetation at 1830–1980 m elevation along the NW upper slope, and six specimens were taken from leaf litter samples of *Dubautia reticulata* litter at “Frisbee Meadow Camp” in the headwaters of Hanawī Stream to the east (Fig. 48). Whether the unusual coal-black color and ridged dorsal body surface of *Mecyclothorax anthracinus* beetles serve to enhance crypsis on the dark, fissured bark of the tree *Dubautia* seems a question worthy of study.

#### (031) Mecyclothorax arthuri sp. n.

Taxon classificationAnimaliaColeopteraCarabidae

http://zoobank.org/0FF1F2CC-836D-46A6-A04E-4AF8256C032B

**Figure 49.**
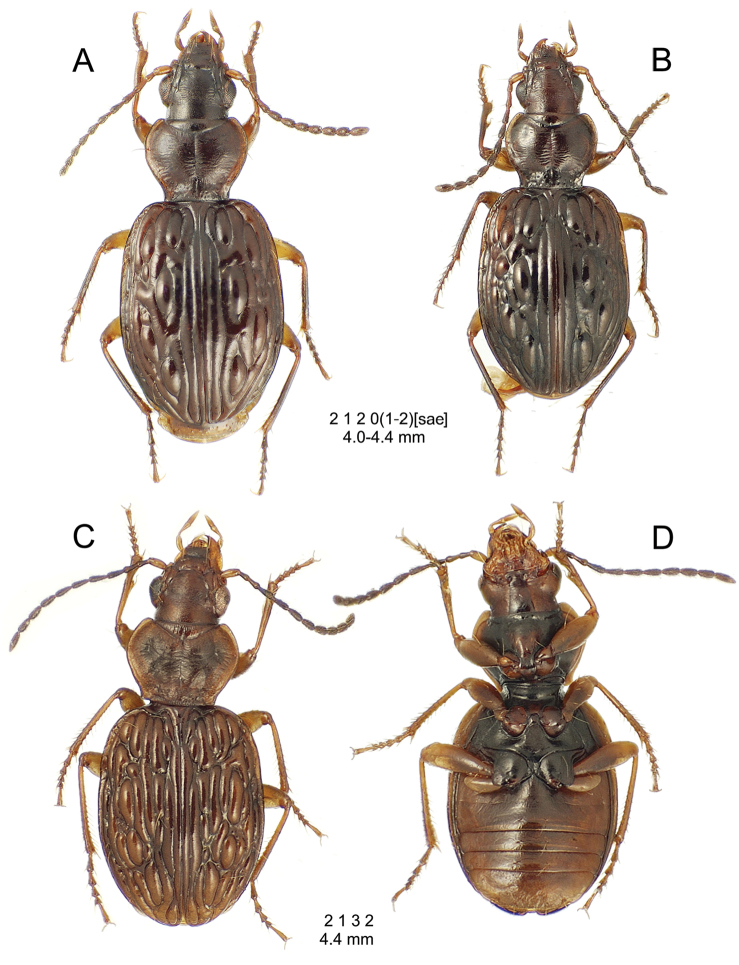
*Mecyclothorax interruptus* group species, habitus view. **A–B** *Mecyclothorax arthuri* (ESE Kuiki, 2145m). **A** Female specimen **B** Male specimen **C–D** *Mecyclothorax medeirosi* (New Greensword Bog, 1850 m) **C** Dorsal view **D** Ventral view.

Figs 45H–J , 46C , 47C , 49A–B , 51

##### Diagnosis.

This species can be diagnosed by the narrow pronotum relative to the elytra, MEW/MPW = 1.64–1.67, and the irregularly anastomosing striae 2–7, resulting in massive convex warts that may or may not be bilaterally symmetrical (Fig. 49A–B). These characters set this species apart from all others from Haleakalā except *Mecyclothorax medeirosi* below. That species differs by a relatively broader pronotum (Fig. 49C); MEW/MPW = 1.47. A third species, *Mecyclothorax oppenheimeri* Liebherr from West Maui, shares the warty elytral condition of *Mecyclothorax arthuri* and *Mecyclothorax medeirosi* while exhibiting a pronotum of relative width intermediate to that of those two species; i.e. MEW/MPW = 1.52–1.57 (Liebherr 2011, fig. 36). Setal formula: 2 1 2 0(1-2)[sae]. The variation in the apical elytral setae is distributed as: 5 individuals with both setae absent; 1 individual with apical seta absent and subapical setae present; and 1 individual with both apical and subapical setae present. Standardized body length 4.0–4.4 mm.

##### Description

(n = 5). *Head capsule* with frontal grooves deep near clypeus, straight, with external carina; dorsal surface of neck flat to convex; eyes moderately convex, ocular ratio = 1.43–1.47, covering ¾ of slightly protruded ocular lobe, ocular lobe ratio = 0.72–0.80; labral anterior margin broadly, moderately deeply emarginate; antennae filiform, antennomeres 2–3 with sparse pelage of short setae; mentum tooth with sides acute, apex rounded. *Pronotum* bisetose, lateral seta present, basal seta absent, basally constricted, MPW/BPW = 1.46–1.53; hind angle acute, apex acuminate, lateral margin broadly convergent anterad angle; median base depressed relative to disc, with sparsely distributed punctures near basal margin, longitudinal wrinkles at juncture with disc; basal margin straight between laterobasal depressions; median longitudinal impression shallow, indistinct, to deep, distinct, always crossed by transverse wrinkles emanating onto disc; anterior transverse impression deeply incised, complete, short wrinkles extended from impression posteriorly onto disc; anterior callosity broadly convex, smooth; front angles slightly produced, tightly rounded; width between front angles greater than basal width, APW/BPW = 1.01–1.08; lateral marginal depression narrow, edge beaded; laterobasal depression broad, a depressed expansion of lateral depression. *Proepisternum* with 6 minute punctures along hind marginal groove; prosternal process medially depressed, with broad marginal bead. *Elytra* broadly subquadrate, lateral margins convex from humerus to subapical sinuation; basal groove incrementally recurved, bordering 4 basal convexities mesad tightly rounded to subangulate humeral angle; humeri narrow relative to broadest portion of elytra behind midlength; MEW/HuW = 2.07–2.16; parascutellar seta present; parascutellar striole narrow, deep, directly connected to isolated basal portion of sutural stria; sutural interval as convex as interval 2, though less convex than warty protuberances associated with intervals 3–7; discal striae lined with sculpticells, smooth; sutural and 2^nd^ striae of subequal depth in apical half of elytra; discal striae 2–6 joined irregularly at positions of dorsal elytral setae, larger convexities associated with striae 2–4, smaller more irregular convexities laterad; warty convexities vary among individuals, as well as bilaterally (Fig. 49A–B); 7^th^ and 8^th^ interval of similar convexity mesad subapical sinuation; 2 dorsal elytral setae at 0.31× and 0.69× elytral length, setal impressions very small, shallow, not distinctly associated with a depressed stria; lateral elytral setae arranged in anterior series of 5 setae, an isolated intermediate seta, and 4 posterior setae; elytral marginal depression broad laterally, narrow behind; subapical sinuation shallow, broad. *Mesepisternum* with 8 shallow punctures in 2 rows; metepisternal width to length ratio = 0.72; metepisternum/metepimeron suture distinct; metathoracic flight wing an ovoid flap 3.3× long as broad, remnant R and M veins present, flap extended to hind margin of metanotum. *Abdomen* with indistinct lateral wrinkles on ventrites 1–3; suture between ventrites 2 and 3 complete; apical male ventrite with 2 marginal setae, apical female ventrite with 4 equally spaced marginal setae and median trapezoid of 4 subequal, short setae. *Legs*-metatarsomere 1/metatibial length ratio 0.20; metatarsomere 4 length along outer lobe 1.2× medial tarsomere length, apical and subapical setae present; metatarsal dorsolateral sulci broad, shallow. *Microsculpture* of vertex a granulate isodiametric mesh; pronotal disc with shallow transverse mesh median base with isodiametric sculpticells and glossy portions; elytral disc and apex with very shallow isodiametric sculpticells in transverse rows; metasternum with distinct transverse mesh; laterobasal abdominal ventrites with swirling isodiametric and transverse microsculpture. *Coloration* of vertex rufobrunneous with piceous cast; antennomere 1 rufoflavous, antennomeres 2–11 rufopiceous; pronotal disc rufobrunneous, pronotal margins broadly paler, rufoflavous; proepipleuron flavous, proepisternum rufous; elytral disc rufobrunneous, sutural interval concolorous basally, rufous apically; elytral marginal depression narrowly rufoflavous, apex concolorous with disc; elytral epipleuron rufoflavous, metepisternum rufous; abdomen rufobrunneous mediobasally, all ventrites flavous laterally, the apical ventrite with apex broadly flavous; metafemur flavous with piceous basal cloud; metatibia rufobrunneous, piceous cast medially.

**Male genitalia** (n = 1). Aedeagal median lobe slender, distance from parameral articulation to tip 5.1× depth at midlength (Fig. 45H); apex extended 4× its depth beyond ostial opening, gradually narrowed to narrowly rounded tip; median lobe constricted laterally toward apex in ventral view, right margin distinctly concave, left margin more gradually narrowed, tip blunt (Fig. 45J); internal sac with bulbous ventral lobe near midlength, apex broadly rounded with small sclerotized flagellar plate visible along dorsal surface of apical lobe (Fig. 45H–I), sac surface uniformly covered with microspicules.

**Female reproductive tract** (n = 1). Bursa copulatrix very short, broad, little extended from broad vaginal base, length 0.23 m, breadth 0.40 mm (Fig. 46C); bursal walls translucent, thinly wrinkled; gonocoxite 1 with 3–4 apical fringe setae in oblique series, 4–6 smaller setae on medial surface (Fig. 47C); gonocoxite 2 subtriangular, apex evenly narrowed, base broadly extended laterally, 2 narrow lateral ensiform setae and broad dorsal ensiform seta with rounded apex, apical nematiform setae on medioventral surface at 0.75× gonocoxite length.

##### Holotype.

Male (CUIC) dissected and labeled: HI: Maui Haleakala N.P. / Kuiki, below at 2134 m / N20°42.23', W156°08.00', / 16-V-2001 lot 02 sift / litter under ohia lehua / J.K. Liebherr // HOLOTYPE / Mecyclothorax / arthuri / Liebherr / det. J.K. Liebherr 2015 (black-margined red label).

##### Paratypes.

HI: Maui: Haleakala N.P., Haleakala Crater, Paliku, 2134 m el., 23-vi-1975, Burkhart (BPBM, 1), Kipahulu Vy., sift litter by day, 2100 m el., 07-v-1991 lot 05, Jessel/Medeiros (CUIC, 3), Kuiki, below, sift *Metrosideros* litter, 2145 m el., 16-v-2001 lot 02, Liebherr (CUIC, 2).

##### Etymology.

Patronyms used in this revision honor the contributions of colleagues to the work in hand. The immense contributions of Dr. Arthur Medeiros in teaching the author how to conduct operative science in the Hawaiian rainforest made this entire work possible. Thus this is the first of two patronyms to honor him.

##### Distribution and habitat.

The known distribution of *Mecyclothorax arthuri* straddles the head of Kīpahulu Valley, including Paliku in the eastern end of Haleakalā Crater, Kuiki high along the southwest valley rim, and the upper Kīpahulu Camp sampled by Medeiros and Jessel, 2100 m elevation (Fig. 51). Specimens have been collected by sifting leaf litter.

#### (032) Mecyclothorax medeirosi sp. n.

Taxon classificationAnimaliaColeopteraCarabidae

http://zoobank.org/AB988313-5E6A-4220-8D7D-52F7A9CB4763

Figs 49C–D , 51

##### Diagnosis.

Like *Mecyclothorax arthuri* in exhibiting tortuously anastomosed elytral striae, though in this species all striae from the sutural to 7^th^ stria are involved (Fig. 49C). This species also differs in the relatively broader pronotum; MEW/MPW = 1.47 versus a ratio of 1.64–1.67 for the former species. The pronotum itself is broader, more transverse, MPW/PL = 1.38, versus MPW/PL = 1.21–1.26 in *Mecyclothorax arthuri*. The elytra are also broader basally in this species, with the lateral elytral margins rounded more broadly laterad the angulate humeri (Fig. 49C) than observed in individuals of *Mecyclothorax arthuri* (Fig. 49A–B); MEW/HuW = 1.98 versus values of 2.07–2.16 for the other species. Finally, elytral setation differs between the species, with *Mecyclothorax medeirosi* characterized by three dorsal elytral setae and presence of both apical and subapical setae, producing a setal formula of 2 1 3 2. Standardized body length 4.35 mm.

##### Description

(n = 1). *Head capsule* with frontal grooves sinuous, broad near clypeus and with lateral carina posteriorly; dorsal surface of neck slightly concave; eyes convex, ocular lobe protruded from gena, ocular ratio = 1.48, ocular lobe ratio = 0.75–0.79; labral anterior margin broadly, moderately emarginate, antennae filiform, antennomeres 2–3 with sparse pelage of short setae; mentum tooth with sides acute, apex rounded. *Pronotum* bisetose, hind angles glabrous; base moderately constricted, MPW/BPW = 1.51; hind angle right to obtuse with rounded apex, lateral margin subparallel to slightly divergent anterad angle; median base smooth medially, 5–6 punctures mesad laterobasal depressions; basal margin slightly convex medially; median longitudinal impression deep, broad, medially incised, crossed by transverse wrinkles; anterior transverse impression deeply incised, complete, slightly irregular; anterior callosity broadly convex, crossed by many fine wrinkles; front angles slightly projected, tightly rounded; apical and basal widths subequal, APW/BPW = 1.02; lateral marginal depression moderate, edge upturned to beaded; laterobasal depression broad, flat to slightly upraised by low tubercle. *Proepisternum* with 5 minute punctures along hind marginal groove; prosternal process medially depressed, with narrow marginal bead. *Elytra* broadly subquadrate, disc moderately convex along entire length; basal groove distinctly recurved to subangulate humeral angle; parascutellar seta present; parascutellar striole narrow, deep, isolated from sutural stria; sutural and 2^nd^ striae of subequal depth from base to apex; adjacent striae from the sutural stria to stria 7 confusedly fused, producing numerous, approximately symmetrical wartlike protuberances, all intervals convex, the warts incorporating portions of more than one interval; discal striae smooth, lined with sculpticells only; 7^th^ and 8^th^ interval of similar convexity mesad subapical sinuation; 3 dorsal elytral setae at 0.29×, 0.51×, and 0.64× elytral length, setal impressions very small, shallow, but associated with depressed discal strial fusions; apical and subapical setae present; lateral elytral setae arranged as anterior series of 7 setae and a posterior series of 5–6 setae, with an isolated intermediate seta present on left side; elytral marginal depression broad along anterior setal series, moderate at midlength, narrower behind; subapical sinuation shallow, broad. *Mesepisternum* with ~9 shallow punctures in 1–2 rows; metepisternum nearly quadrate, width to length ratio = 0.93; metepisternum/metepimeron suture distinct. *Abdomen* with indistinct lateral wrinkles on ventrites 1–3; suture between ventrites 2 and 3 complete; apical female ventrite with 4 equally spaced marginal setae, a median trapezoid of 4 setae, the basal setae shorter, a 5^th^ seta on right side. *Legs*-metatarsomere 1/metatibial length ratio = 0.15; metatarsomere 4 length along outer lobe 1.67× median base, the tarsomere broad, robust, apical and subapical setae present; metatarsal dorsolateral sulci broad, shallow. *Microsculpture* of vertex a granulate isodiametric mesh; pronotal disc with granulate isodiametric mesh, some sculpticells in transverse rows; pronotal median base with granulate isodiametric mesh; elytral disc and apex with very shallow isodiametric sculpticells in shallow rows; metasternum with upraised transverse mesh; laterobasal abdominal ventrites with swirling isodiametric and transverse microsculpture. *Coloration* of vertex rufobrunneous with piceous cast; antennomere 1 flavous, antennomeres 2–11 piceous; pronotal disc rufobrunneous, margins broadly paler, rufoflavous; proepipleuron flavous, proepisternum piceous (Fig. 49D); elytral disc with rufobrunneous intervals and darker brunneous depressions and striae, sutural interval concolorous with lateral intervals; elytral margins rufoflavous, apex broadly flavous; elytral epipleuron flavous, metepisternum piceous; abdomen broadly brunneous, a piceous cast mediobasally, abdominal apical ventrite broadly flavous (Fig. 49D); metafemur flavous with indistinct median rufous cloud; metatibia rufobrunneous.

**Female reproductive tract.** The lone female holotype was not dissected.

##### Holotype.

Female (CUIC) labeled: HI: Maui Haleakala N.P. / Northeast Rift New / Greensword Bog sift ex / larger ohias / 17-V-1993 / lot 04 el. 1850 m // J.K. Liebherr & / A.C. Medeiros / Collectors // HOLOTYPE / Mecyclothorax / medeirosi / Liebherr / det. J.K. Liebherr 2015 (black-margined red label).

##### Etymology.

This is the second patronym to honor the contributions of Dr. Art Medeiros, most specifically for field collecting efforts validated in this revision, and more broadly for his leadership in Hawaiian conservation biology (e.g., Perkins et al. 2014). Both *Mecyclothorax arthuri* and *Mecyclothorax medeirosi* occupy distributional ranges in Kīpahulu Valley and the Hāna Bogs region of Haleakalā (Fig. 51). Based on the species known to science to date, the species’ hypothesized sister-taxon relationship can be signified by the convention, *Mecyclothorax arthuri* + *Mecyclothorax medeirosi*.

##### Distribution and habitat.

The single specimen used as the basis for this species description was collected by sifting humus and leaf litter from under an ‘ōhi‘a tree adjacent to New Greensword Bog (Fig. 51).

#### (033) Mecyclothorax inconscriptus sp. n.

Taxon classificationAnimaliaColeopteraCarabidae

http://zoobank.org/D1A93F43-4DAE-4700-B809-E2C57078CBFB

**Figure 50.**
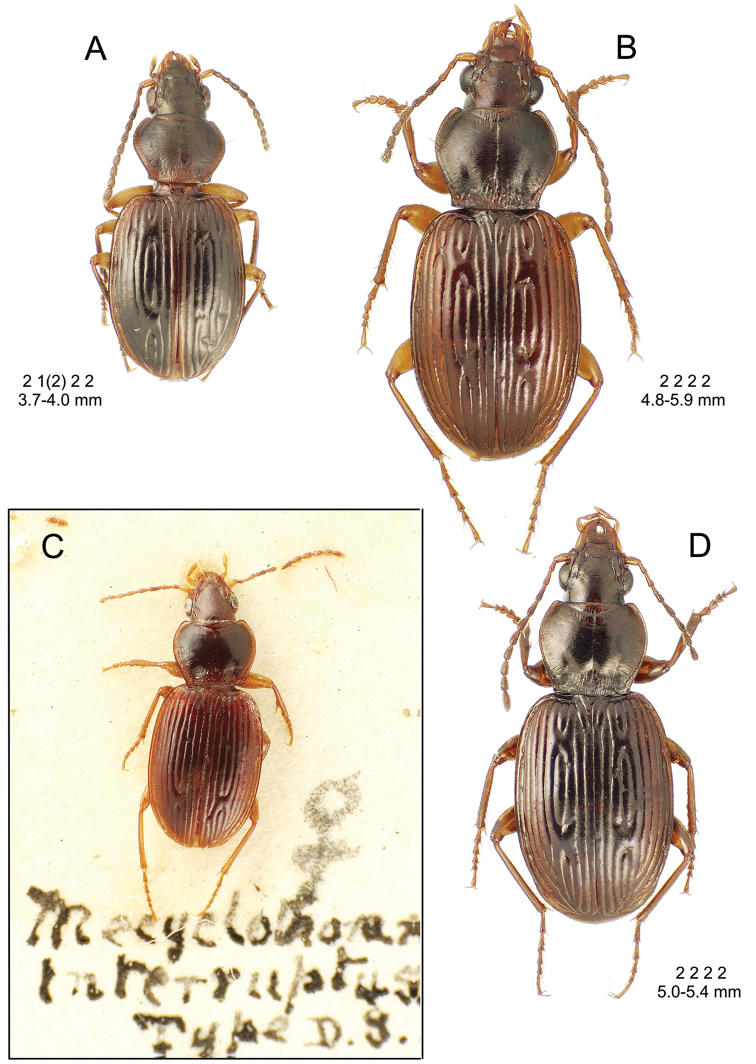
*Mecyclothorax interruptus* group species, dorsal habitus view. **A** *Mecyclothorax inconscriptus* (Kaupō Gap, 1495 m) **B** *Mecyclothorax foveolatus* (Kuhiwa E rim, 880 m) **C** *Mecyclothorax interruptus* lectotype on *Fauna Hawaiiensis* mounting card; “Mecyclothorax interruptus Type D. S.” **D** *Mecyclothorax interruptus* (Honomanu, 1680 m).

**Figure 51.**
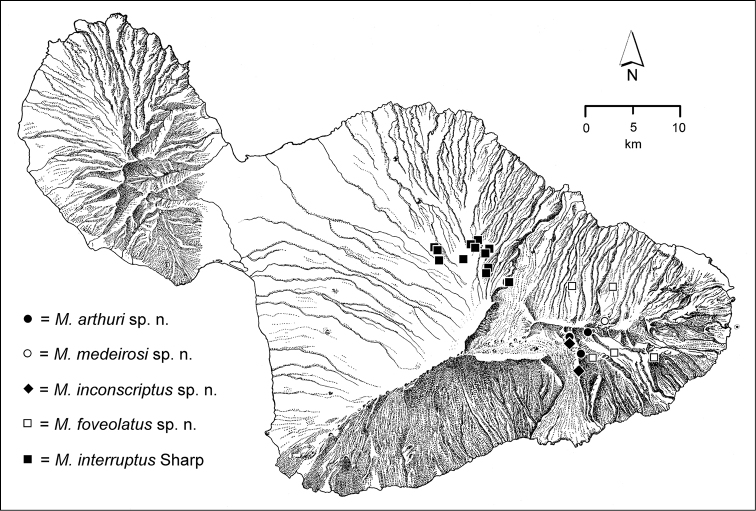
Recorded geographic distributions of *Mecyclothorax interruptus* group species.

Figs 45K–L , 46D , 47D , 50A , 51

##### Diagnosis.

This is one of the three species in the group with body lengths of 4.1 mm or less, accompanying *Mecyclothorax bradycelloides* and *Mecyclothorax anthracinus* in that distinction, however *Mecyclothorax inconscriptus* (Fig. 50A) incongruently differs from those species by sharing with *Mecyclothorax foveolatus* and *Mecyclothorax interruptus* below (Fig. 50B–D), the state of elytral striae 2–4 fused in association with the dorsal elytral setae. The pronotum is basally constricted, MPW/BPW = 1.49–1.52, versus values of 1.38–1.47 for *Mecyclothorax foveolatus* and *Mecyclothorax interruptus*. Pronotal setation also differs from all four species mentioned above in that the basal setae are absent from a slight majority of available specimens, and in the minority of specimens that exhibit the basal setae, they are small and underdeveloped; therefore setal formula 2 1(2) 2 2. Standardized body length 3.7–4.0 mm.

##### Description

(n = 5). *Head capsule* with frontal grooves deep near clypeus, straight with lateral carina; dorsal surface of neck convex; ocular lobe obtusely projected from genal surface, eyes little convex, ocular ratio = 1.36–1.44, ocular lobe ratio = 0.72–0.77; labral anterior margin broadly, shallowly emarginate; antennae filiform, antennomere 3 with sparse pelage of short setae; mentum tooth with sides acute, apex tightly rounded. *Pronotum* transverse, MPW/PL = 1.31–1.37; hind angle obtuse rounded to denticulate, lateral margin straight for short distance to immediately sinuate anterad angle; median base with dense elongate punctures and wrinkles; basal margin nearly straight, slightly convex medially; median longitudinal impression shallow, finely incised, crossed by fine wrinkles; anterior transverse impression shallow, broad, crossed by fine wrinkles; anterior callosity slightly convex, crossed by fine wrinkles; front angles slightly projected, tightly rounded; apical width subequal to slightly larger than basal width, APW/BPW = 1.0–1.04; lateral marginal depression moderate, edge upturned to beaded; laterobasal depression broad, surface wrinkled. *Proepisternum* with 6 minute punctures along hind marginal groove; prosternal process medially depressed with broad marginal bead. *Elytra* subquadrate, disc flat, sides moderately sloped; basal groove slightly recurved to rounded humeral angle, the humeri moderately narrowed relative to greatest width behind midlength, MEW/HuW = 1.93–2.05; parascutellar seta present; parascutellar striole fine, continuous, with 6 small punctures; sutural interval moderately convex, slightly more elevated than intervals 2–4; sutural and 2^nd^ striae of subequal depth from base to apex; discal striae deep, impunctate, continuous to apex with exception of strial fusions; strial fusions include those of striae 3 and 4 in association with dorsal elytral setae, striae 5 and 6 posterad humerus, and irregularly striae 3 and 4, 4 and 5, and 5 and 6 near apex (Fig. 50A); 7^th^ and 8^th^ interval of similar convexity mesad subapical sinuation; 2 dorsal elytral setae at 0.29× and 0.61–0.65× elytral length, setal impressions foveate, spanning interval 3; apical and subapical setae present; lateral elytral setae arranged in anterior series of 7 setae and posterior series of 5 setae; elytral marginal depression narrow, lateral margin upturned; subapical sinuation very shallow, nearly obsolete. *Mesepisternum* with 8 shallow punctures in 2 rows; metepisternal width to length ratio = 0.86; metepisternum/metepimeron suture distinct; metathoracic flight wing an ovoid flap 2.5× long as wide, remnant R plus M veins present, vestige extends ¾ distance to posterior margin of metanotum. *Abdomen* with indistinct lateral wrinkles on ventrites 1–3; suture between ventrites 2 and 3 complete; apical male ventrite with 2 marginal setae, apical female ventrite with 4 equally spaced marginal setae and median trapezoid of 4 subequal, short setae. *Legs*-metatarsomere 1/metatibial length ratio = 0.17; metatarsomere 4 length along outer lobe 1.3× medial tarsomere length, apical and subapical setae present; metatarsal dorsolateral sulci broad, shallow. *Microsculpture* of vertex granulate isodiametric; pronotal disc with granulate transverse mesh, median base with granulate isodiametric mesh; elytral disc with elongate transverse mesh, apex with well-developed isodiametric mesh in transverse rows; metasternum with upraised transverse mesh; laterobasal abdominal ventrites with swirling isodiametric and transverse microsculpture. *Coloration* of vertex rufobrunneous; antennomere 1 flavous, antennomeres 2–3 rufopiceous, 4–11 piceous; pronotal disc rufobrunneous with piceous cast, margins concolorous; proepipleuron rufoflavous, proepisternum rufobrunneous with piceous cast; elytral disc rufopiceous, sutural interval paler, rufous throughout length, lateral marginal depression paler at base, 8^th^ stria and depression paler at apex; elytral epipleuron rufoflavous, metepisternum rufobrunneous; abdomen rufopiceous across width of ventrites 1–5, apical ventrite 6 with apical 1.3 paler, rufobrunneous; metafemur flavous with piceous cast in basal 1/3; metatibia flavous with piceous cast.

**Male genitalia** (n = 1). Aedeagal median lobe short, robust, distance from parameral articulation to tip 2.2× depth at midlength (Fig. 45K); apex narrowly extended beyond ostial opening 3× its depth, tip tightly rounded at juncture of flattened apical face and downturned ventral margin; median lobe straight in ventral view, right and left margins convergent to blunt tip (Fig. 45L); internal sac unornamented, flagellar plate large, sclerotized plate visible in lateral view, plate length 0.58× parameral articulation-tip distance.

**Female reproductive tract** (n = 1). Bursa copulatrix very broad basally, narrowed to a nipplelike apical lobe, overall length 0.51 mm, apical lobe width 0.09 mm, basal width at vagina 0.44 mm (Fig. 46D); bursal walls transparent at base, wrinkled in extension apicad juncture with common oviduct, apical lobe translucent, thinly wrinkled; gonocoxite 1 with 2 subequal apical fringe setae, 4 smaller setae on medial surface (Fig. 47D); gonocoxite 2 broadly subtriangular, apex tightly rounded, base broadly extended, 2 lateral ensiform setae with apical seta broader and longer, apical nematiform setae on medioventral surface at 0.72× gonocoxite length.

##### Holotype.

Male (CUIC) dissected and labeled: HI: Maui Haleakala N.P. / Kaupo Gap el. 1495 m / N20°41'48", W156°08'22" / 17-18-V-2001 lot03 koa/ / fern/moss litter J. Liebherr / 2 / HOLOTYPE / Mecyclothorax / inconscriptus / Liebherr / det. J.K. Liebherr 2015 (black-margined red label).

##### Paratypes.

HI: Maui: Haleakala N.P., Haleakala Crater, Paliku, pitfall, 1900 m el., 14-15-iii-2002, Takumi (BPBM, 1), Kaupo Gap, sift litter *Acacia koa*/fern/moss, 1495 m el., 17–18-v-2001 lot 03, Liebherr (CUIC, 5).

##### Etymology.

The Latin participle conscriptus is used as the stem of this epithet, with the converse inconscriptus signifying this species’ membership in the *Mecyclothorax interruptus* species group.

##### Distribution and habitat.

*Mecyclothorax inconscriptus* exhibits a Kaupō Gap distribution (Fig. 51) biogeographically congruent with that of *Mecyclothorax cordaticollaris* (Fig. 77). The two collecting records indicate occupation of mesic to dry ground-level microhabitats, with one specimen collected in a pitfall trap at Paliku, and a series of four individuals found in a sift sample of *Acacia koa* leaves, mosses, and dead fern fronds.

#### (034) Mecyclothorax foveolatus sp. n.

Taxon classificationAnimaliaColeopteraCarabidae

http://zoobank.org/CB0B5A98-BE78-4139-8956-B8B879B0013F

Figs 45M–N , 46E , 47E , 50B , 51

##### Diagnosis.

This and the following species, *Mecyclothorax interruptus*, are cryptic sibling species with only subtle differences both externally (Fig. 50B, D) and in the male genitalia (Fig. 45M–P), but nevertheless they may be consistently diagnosed morphologically. This species differs from its sibling in its more upraised cuticular microsculpture; 1, vertex covered by a granulate isodiametric mesh, the surface appearing coriaceous, matte and non-reflective; 2, pronotal disc with coriaceous isodiametric and transverse sculpticells in transverse rows, the pronotal base with granulate isodiametric mesh, again the surface with a matte finish. The corresponding microsculpture in *Mecyclothorax interruptus* individuals is: 1, vertex with isodiametric mesh, the sculpticell surfaces shiny in part; 2, pronotal disc with mixture of isodiametric and transverse sculpticells, the surface iridescent, and pronotal base with flattened isodiametric sculpticells, the surface shiny in part. The discal elytral striae are also smoother in this species, with minute punctulae at the deepest parts of the striae associated with longitudinal irregularities in the strial orientation, whereas in *Mecyclothorax interruptus* individuals the striae, especially the sutural and 2^nd^ stria, are minutely but clearly punctate in their deepest portions, with those small punctures slightly expanding the stria at its deepest point. The male median aedeagal lobe apex in this species has a distinct obtuse tooth on its dorsal surface (Fig. 45M–N) versus the lobe apex of *Mecyclothorax interruptus* males with a rounded dorsal projection (Fig. 45O–P). The median lobe tip is also flattened apically in this species, versus more evenly rounded in *Mecyclothorax interruptus*. Setal formula 2 2 2 2. Standardized body length 4.8–5.9 mm.

##### Description

(n = 5). *Head capsule* with frontal grooves deep and broad near clypeus, sinuously directed to terminus mesad anterior supraorbital seta; dorsal surface of neck flat to convex; eyes moderately convex, ocular ratio = 1.42–1.50, ocular lobe distinctly projected from gena; ocular lobe ratio 0.79–0.88; labral anterior margin with broad, moderately deep emargination; antennae filiform, antennomere 3 with sparse pelage of short setae; mentum tooth with side acute, apex broadly rounded. *Pronotum* moderately transverse, MPW/PL = 1.19–1.29, moderately constricted basally, MPW/BPW = 1.38–1.46; hind angle variably obtuse, to right, to slightly acute, apex tightly rounded, lateral margin subparallel anterad angle; median base with dense elongate punctures and longitudinal wrinkles; basal margin nearly straight, slightly convex medially; median longitudinal impression broad, shallow, crossed by wrinkles; anterior transverse impression deeply incised, complete, crossed by longitudinal wrinkles that extend across broadly, slightly convex anterior callosity; front angles slightly projected, rounded; pronotal apical and basal widths subequal, APW/BPW = 0.95–1.06; lateral marginal depression moderate, edge broadly upturned; laterobasal depression broad, smooth, with median tubercle. *Proepisternum* with 6 small, elongate punctures along hind marginal groove; prosternal process broad, medially depressed with lateral marginal bead. *Elytra* subquadrate, disc slightly convex; basal groove evenly recurved to join lateral marginal depression at rounded humerus; MEW/HuW = 1.79–2.02; parascutellar seta present (on 1 specimen near base of stria 2); parascutellar striole deep, continuous; sutural interval moderately convex, convexity similar to that of lateral intervals of similar breadth; sutural and 2^nd^ striae of subequal depth from base to apex; discal striae 2–4 fused in association with dorsal elytral setae, striae 5 and 6 may be fused behind humerus, intervals moderately convex to convex; 7^th^ and 8^th^ intervals of similar convexity mesad subapical sinuation; 2 dorsal elytral setae at 0.29× and 0.60× elytral length, setal impressions foveate, placed within depressed areas associated with strial fusions; apical and subapical setae present; lateral elytral setae arranged as anterior series of 7 setae (or anterior series of 6 with isolated 7^th^ intermediate seta) and a posterior series of 6 setae; elytral marginal depression narrow, lateral margin upturned; subapical sinuation broad and very shallow. *Mesepisternum* with ~22 distinct punctures 3–4 rows; metepisternal width to length ratio = 0.76; metepisternum/metepimeron suture distinct; metathoracic flight wing configuration an ovoid flap, length 2.1× breadth, remnant R and M veins present, the flap extended 1/3 length beyond posterior margin of metanotum. *Abdomen* with indistinct lateral wrinkles on ventrites 1–3; suture between ventrites 2 and 3 complete; apical male ventrite with 2 marginal setae, apical female ventrite with 4 equally spaced marginal setae and median trapezoid of 4 short setae, the basal pair slightly longer. *Legs*-metatarsomere 1/metatibial length ratio = 0.22; metatarsomere 4 length along outer lobe 1.4× medial tarsomere length, apical and subapical setae present; metatarsal dorsolateral sulci broad, deep, median area irregular. *Microsculpture* of elytral disc an elongate transverse mesh, apex with upraised transverse mesh; metasternum with obsolete transverse mesh; laterobasal abdominal ventrites with elongate transverse mesh and glossy areas. *Coloration* of vertex brunneous; antennomere 1 flavous, antennomeres 2–3 flavous with a piceous cast, 4–11piceous; pronotal disc brunneous with piceous cast, margins broadly rufoflavous; proepipleuron flavous with rufous cast, proepisternum rufopiceous; elytral disc rufobrunneous with piceous cast, sutural interval concolorous, margins concolorous to slightly darker; elytral epipleuron flavous with rufous cast, metepisternum piceous; abdominal ventrites 1–6 piceous medially, flavous laterally, the apical ventrite flavous in apical half; metafemur flavous; metatibia flavous with brunneous cast.

**Male genitalia** (n = 2). Aedeagal median lobe slender, distance from parameral articulation to tip 6.6× depth at midlength (Fig. 45M), shaft slightly curved basally, ventral margin straight apically, apex with acute dorsal projection, tip tightly rounded at juncture of flat apical face and ventral margin; in ventral view median lobe slightly curved rightward toward apex, right margin concave, left margin more incurved to apex, apical denticle visible to left of rounded tip (Fig. 45N); internal sac without ornamentation.

**Female reproductive tract** (n = 1). Bursa copulatrix elongate with broad base at vagina, length 0.60 mm, apical width 0.23 mm, basal width 0.34 mm (Fig. 46E); bursal walls translucent, thinly wrinkled; gonocoxite 1 with 4 apical fringe setae and 4–6 smaller setae on medial surface (Fig. 47E); gonocoxite 2 subtriangular, apex acuminate, base evenly extended from lateral margin, 2 parallel-sided lateral ensiform setae, apical nematiform setae on medioventral surface at 0.79× gonocoxite length.

##### Holotype.

Male (CUIC) dissected and labeled: HI: Maui Haleakala N.P. / Kipahulu west rim ESE / Kuiki, sift humus ex ohia / 15-V-1993 lot 03 / el. 1850 m // J.K. Liebherr / A.C. Medeiros, / Jr. collectors // HOLOTYPE / Mecyclothorax / foveolatus / Liebherr / det. J.K. Liebherr 2015 (black-margined red label).

##### Paratypes.

HI: Maui: Haleakala N.P., Kipahulu Vy., Central Pali Tr., sift leaf/moss litter, 1200 m el., 29-iv-1991 lot 03, Liebherr/Medeiros (CUIC, 1), Kipahulu west rim ESE Kuiki, sift *Metrosideros* humus, 1850 m el., 15-v-1993 lot 05, Liebherr/Medeiros (CUIC, 1); Hana For. Res., Kaumakani Peak, pyrethrin fog vegetation, 1165 m el., 08-vi-1999 lot 04, Polhemus (NMNH, 1); Koolau For. Res., Hanawi N.A.R., Kopiliula Str., pyrethrin fog *Acacia koa* trunk, 1127 m el., 03-v-1998 lot 02, Liebherr (CUIC, 1), Kuhiwa Vy. E rim, pyrethrin fog *Metrosideros*, 880 m el., 09-vi-1999 lot 04, Polhemus (NMNH, 1), lot 07, Polhemus (NMNH, 2), lot 09, Polhemus (NMNH, 1).

##### Etymology.

The foveae surrounding dorsal elytral setae of this species are the basis for use of the Latin adjectival foveatus as this species’ epithet.

##### Distribution and habitat.

*Mecyclothorax foveolatus* exhibits a highly fragmented distribution comprising five localities spanning the Manawainui Planeze, Kīpahulu Valley, Kaumakani Peak, and Kuhiwa and Kopili‘ula drainages in Hanawī (Fig. 51). The Kopili‘ula record alone is associated with koa, whereas the other records are associated with ‘ōhi‘a; either through sifting leaf litter, humus, or moss, or by using pyrethrin fog on mossy trunks and logs.

#### (035) Mecyclothorax interruptus

Taxon classificationAnimaliaColeopteraCarabidae

Sharp

Figs 45O–P , 46F , 47F , 50C–D , 51

Mecyclothorax interruptus Sharp 1903: 252; Britton 1948b: 163.
Mecyclothorax interruptus var. dubius Sharp 1903: 252; Britton 1948b: 163 (synonymy).

##### Diagnosis.

This species (Fig. 50D) can be diagnosed from its cryptic sibling species, *Mecyclothorax foveolatus* (Fig. 50B), using the criteria presented under that species (above). These two are the only Haleakalā species exhibiting: 1, strial fusion implicating striae 2–4 at the positions of the dorsal elytral setae; and 2, body sizes above 5 mm. Setal formula 2 2 2 2. Standardized body length 5.0–5.4 mm.

##### Identification

(n = 5). The eyes are moderately convex, ocular ratio = 1.37–1.50, and they cover much of the ocular lobe (Fig. 50D), ocular lobe ratio = 0.79–0.88. The pronotum is little constricted basally, MPW/BPW = 1.35–1.43, with the hind angle right to slightly obtuse, and the lateral margin briefly sinuate before the angle. The pronotal disc is smooth, glossy, with fine transverse wrinkles that extend little from the shallow, finely incised median longitudinal impression. The pronotal median base is covered with small punctures and fine longitudinal wrinkles. The elytra are subquadrate, with the humeri broadly rounded; MEW/HuW = 1.68–1.94. The dorsal body coloration is dark, with the vertex and pronotal disc rufopiceous, elytral disc rufobrunneous, and metafemur and metatibia flavous with a brunneous cast.

**Male genitalia** (n = 3). Aedeagal median lobe very slender, distance from parameral articulation to tip 6.8× depth at midlength (Fig. 45O), shaft slightly curved basally, ventral margin dorsally recurved apically, apex with bluntly rounded dorsal projection, tip broadly tightly rounded, apical face convex; in ventral view median lobe slightly curved rightward toward apex, right margin slightly concave, left margin more incurved to rounded apex, blunt projection visible as apical expansion of rounded tip (Fig. 45P); internal sac without ornamentation.

**Female reproductive tract** (n = 1). Bursa copulatrix a narrow tube with apical lobe, medial bulge, and basal constriction (Fig. 46F), overall length 0.80 mm, apical breadth 0.17 mm, medial bulge breadth 0.23 mm; bursal walls translucent, thinly wrinkled; gonocoxite 1 with 2–3 apical fringe setae and 5–6 smaller setae on medial surface (Fig. 47F); gonocoxite 2 subtriangular, apex broad, base evenly extended laterally from curved lateral margin, 2 broad lateral ensiform setae, apical nematiform setae on medioventral surface at 0.73× gonocoxite length.

##### Lectotypes.

For *Mecyclothorax interruptus Sharp*, female (BMNH) hereby designated, labeled: ♀ (in pencil) / Mecyclothorax / interruptus / Type D.S. / Haleakala / Perkins 597 (on obverse of mounting platen) // Type (round, red-margined label) // Hawaiian Is. / Perkins / 1904–336. / LECTOTYPE / Mecyclothorax / interruptus / Sharp / J.K. Liebherr 1998 (black-margined red label). For Mecyclothorax interruptus var. dubius Sharp, female (BMNH) hereby designated, labeled: M. in- / terruptus. var / dubius. D.S. / Haleakala / Perkins (on obverse of mounting platen), 623. (on reverse of platen) // Hawaiian Is. / Perkins / 1904–336. // LECTOTYPE / Mecyclothorax / interruptus var. dubius / Sharp / J.K. Liebherr 1998 (black-margined red label).

##### Distribution and habitat.

*Mecyclothorax interruptus* is broadly distributed across the Waikamoi block of forest from 1170–1860 m elevation (Fig. 51). This species has been found by sifting ‘ōhi‘a leaf litter, searching under boards or logs on the ground, and by pyrethrin sampling moss-covered standing tree trunks and horizontal nurse logs.

### *Mecyclothorax sobrinus* species group

**Diagnosis.** Species placed in this group display dorsal elytral setae set in foveate impressions spanning the third interval, plus well-developed, isodiametric dorsal microsculpture—just as do species in the *Mecyclothorax interruptus* group. But this group’s species are also characterized by shallower lateral elytral striae 6 and 7, whereas in the *Mecyclothorax interruptus* group species all striae—sutural to 8^th^—are of subequal depth. This distinction was established in Britton’s (1948b) classification. In *Mecyclothorax sobrinus* group species, the 8^th^ elytral interval is convexly raised mesad the subapical sinuation so that it is more convex than both the 9^th^ interval bordering the lateral marginal depression and the apical portion of fused striae 5 + 7. In association with this convexity, stria 7 is markedly depressed apicolaterad the narrowed termination of interval 6 associated with fusion of intervals 5 and 7. These characters represent synapomorphies for this group. *Mecyclothorax sobrinus* group species exhibit both anterior and posterior supraorbital setae, lateral and basal pronotal setae, apical and subapical elytral setae, and at least 2 dorsal elytral setae, leading to a base setal formula of 2 2 2 2. *Mecyclothorax multipunctatus* and *Mecyclothorax inaequalis* deviate from this formula by including individuals that exhibit additional setae on the 5^th^, or 5^th^ and 7^th^ elytral intervals. For these species, the impressions that connect the outward adjacent striae are associated with setae; a condition not observed in *Mecyclothorax interruptus* group species. As all species in both groups are predominantly encountered at the soil level within leaf litter and humus, the possibility that foveate setal impressions and strial fusions converge on some sort of cryptic litter-running facies that can confuse visual predators—presumably avian first, but presently also including the entomologist—should not be discarded out of hand. Conversely, dimpled elytra may serve better to reduce drag during a beetle’s wedge-pushing (Evans 1977) through the leaf and humus layers, at the same time positioning dorsal sensory setae within protective fossae.

**Membership and distribution.** This monophyletic species group comprises seven species restricted to Haleakalā. As West Maui, Moloka‘i, and Haleakalā lost terrestrial connections on the order of 700,000 years ago (Price and Elliott-Fisk 2004), several competing hypotheses present themselves regarding this distributional pattern: 1, the group has radiated on Haleakalā within the past 700,000 years; 2, some biogeographic barrier or ecological criterion restricted this group to Haleakalā during earlier times when the present-day fragments of Maui Nui were terrestrially connected; 3, representatives of this group remain undiscovered, or underwent extinction in West Maui or Moloka‘i. Data currently in hand support a narrow set of ecological preferences for these species, as five of them are sympatrically distributed in Koa-‘Ōhi‘a Mesic Forest within the Waikamoi area, whereas the remaining two are restricted to the mesic forest at Polipoli (Figs 56, 59).

#### Key to adults of the *Mecyclothorax sobrinus* species group, Haleakalā volcano, Maui, Hawai‘i

**Table:** 

1	Dorsal elytral setae in intervals 3 and 5, or 3, 5, and 7 (Fig. 52A–B)	**2**
1’	Two dorsal elytral setae restricted to third interval (Figs 52C–D, 57)	**3**
2(1)	Dorsal elytral setae in striae 3 and 5, unilaterally in interval 7 in some specimens, the elytral striae regular, not fused in association with the setae (Fig. 52A); elytral intervals with regular transverse-mesh microsculpture	(036) ***Mecyclothorax multipunctatus* (Blackburn)**
2’	Dorsal elytral setae situated in intervals 3, 5, and 7, adjacent elytral striae fused in association with setae (Fig. 52B); elytral intervals with coriaceous isodiametric microsculpture, the surface pearlaceous	(037) ***Mecyclothorax inaequalis* (Blackburn)**
3(1)	Elytra subquadrate to subellipsoid, lateral margins convex, greatest width may be behind midlength (Figs 52D, 57)	**4**
3’	Elytra quadrate, broad basally with subparallel lateral margins (Fig. 52C)	(038) ***Mecyclothorax longulus* Sharp**
4(3)	Larger beetles, standardized body length 5.5–6.6 mm; vertex and pronotal disc with upraised isodiametric to transverse microsculpture, the surface pearlaceous (Fig. 57)	**5**
4’.	Smaller beetles, standardized body length 4.5–4.9 mm; vertex and pronotal disc with transverse mesh microsculpture, the surface glossy (Fig. 52D)	(039) ***Mecyclothorax giffardi* Liebherr**
5(4)	Elytral humeri broad, lateral margins evenly convex with maximum width at position between anterior and posterior dorsal elytral setae (Fig. 57B–C); male aedeagal median lobe with a spatulate apex and a projected parapical extension (Fig. 58)	**6**
5’	Elytral humeri narrow, maximum elytral width at position of posterior dorsal elytral setae (Fig. 57A); male aedeagal median lobe a simple shaft without parapical extension (Fig. 53H)	(040) ***Mecyclothorax foveopunctatus* sp. n.**
6(5)	Dorsal surface with exceedingly upraised microsculpture, the isodiametric sculpticells of vertex, pronotal disc, and elytral disc causing intense pearlaceous reflection (Fig. 57B); 2–4 dorsal elytral setae each side in third interval, their positions often unilaterally asymmetrical; elytral intervals 3–6 distinctly and irregularly depressed posterad humerus; male aedeagal median lobe with broad, spatulate apex and robust parapical extension (Fig. 58A–B)	(041) ***Mecyclothorax consobrinus* Liebherr**
6’	Dorsal surface with regular upraised isodiametric microsculpture, the sculpticells in transverse rows on pronotal and elytral discs, surface evenly pearlaceous (Fig. 57C); 2 dorsal elytral setae each side, nearly always symmetrically paired; elytral intervals 4–6 moderately and evenly depressed posterad humerus; male aedeagal median lobe with narrow, hooked apex and digitiform parapical extension (Fig. 58D–E)	(042) ***Mecyclothorax sobrinus* Sharp**

#### (036) Mecyclothorax multipunctatus

Taxon classificationAnimaliaColeopteraCarabidae

(Blackburn)

**Figure 52.**
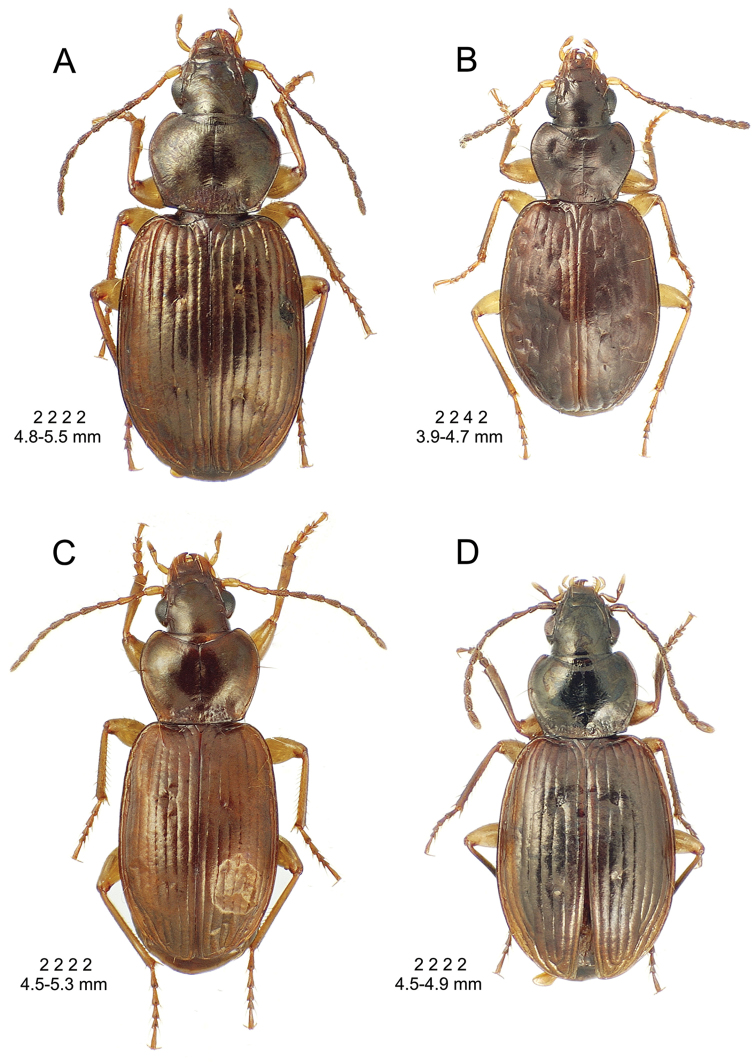
*Mecyclothorax sobrinus* group species, dorsal habitus view. **A** *Mecyclothorax multipunctatus* (nr. Ukulele Camp, 1615 m) **B** *Mecyclothorax inaequalis* (nr. Ukulele Camp, 1615 m) **C** *Mecyclothorax longulus* (nr. Ukulele Camp, 1615 m) **D** *Mecyclothorax giffardi* (Kahikinui, 1616 m).

**Figure 53.**
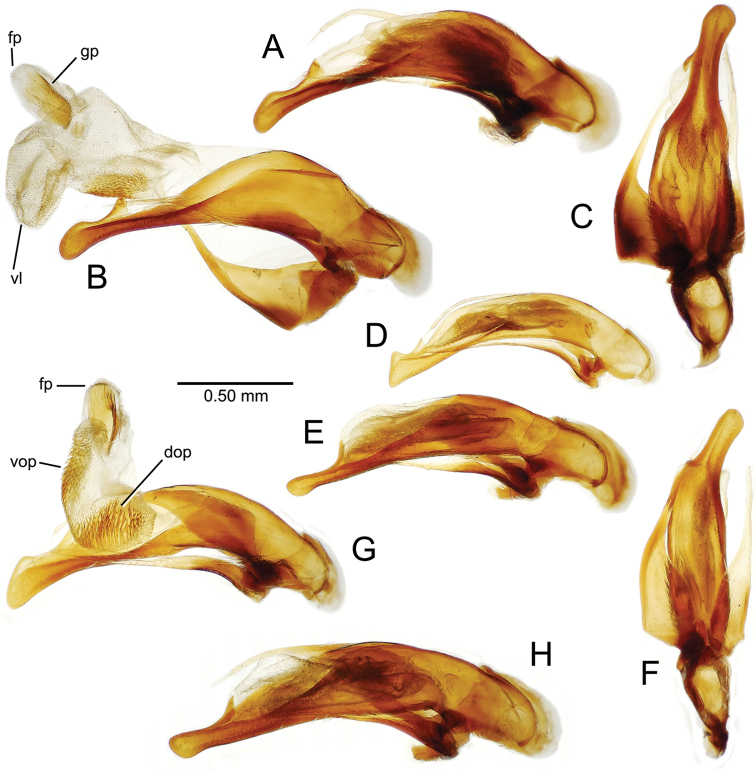
Male aedeagus, *Mecyclothorax sobrinus* group species (for abbreviations see Table 2, p. 23). **A–C** *Mecyclothorax multipunctatus* (nr. Ukulele Camp, 1615 m). **A** Right view **B** Right view, sac everted **C** Ventral view **D** *Mecyclothorax inaequalis*, right view (nr. Ukulele Camp, 1615 m) **E–F** *Mecyclothorax longulus*, right and ventral views (nr. Ukulele Camp, 1615 m) **G** *Mecyclothorax giffardi*, right view, sac everted (Kahikinui, 1616 m) **H** *Mecyclothorax foveopunctatus*, right view (Ukulele Camp Pipeline, 1495–1525).

**Figure 54.**
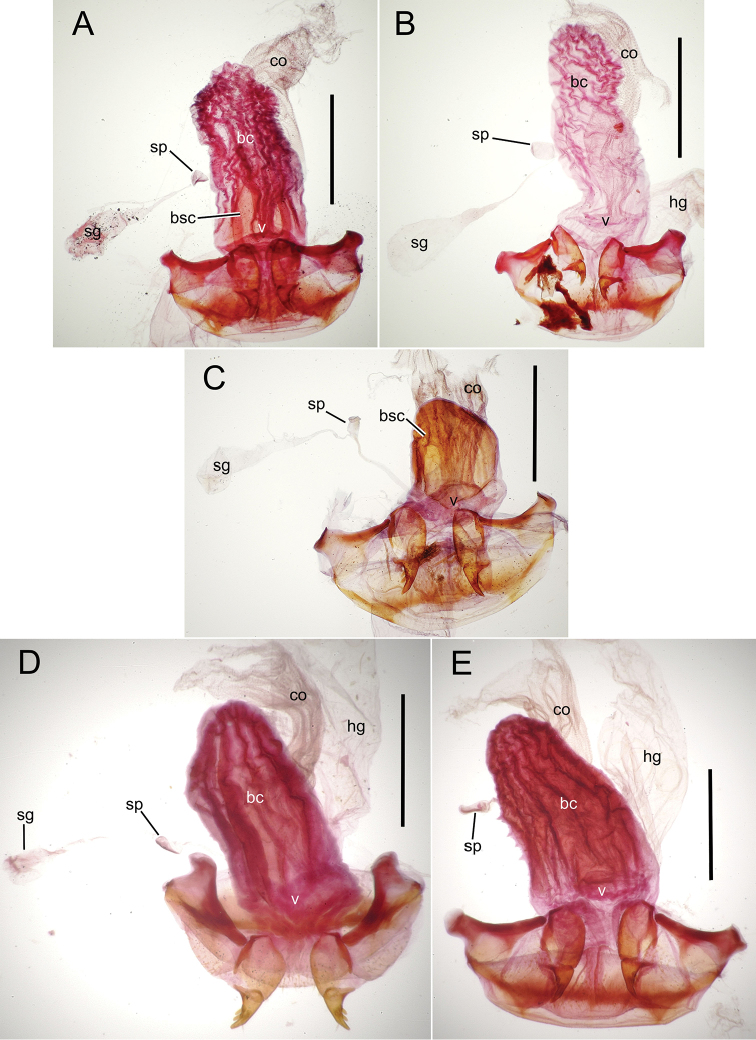
Female bursa copulatrix and associated reproductive structures, *Mecyclothorax sobrinus* group species, ventral view (for abbreviations see Table 2, p. 23). **A** *Mecyclothorax multipunctatus* (nr. Ukulele Camp, 1615 m) **B** *Mecyclothorax inaequalis* (nr. Ukulele Camp, 1615 m) **C** *Mecyclothorax longulus* (nr. Ukulele Camp, 1534–1660 m) **D** *Mecyclothorax consobrinus* (Polipoli, 1890 m). **E** *Mecyclothorax sobrinus* (Honomanu, 1830–1860 m). Scale bar = 0.50 mm.

**Figure 55.**
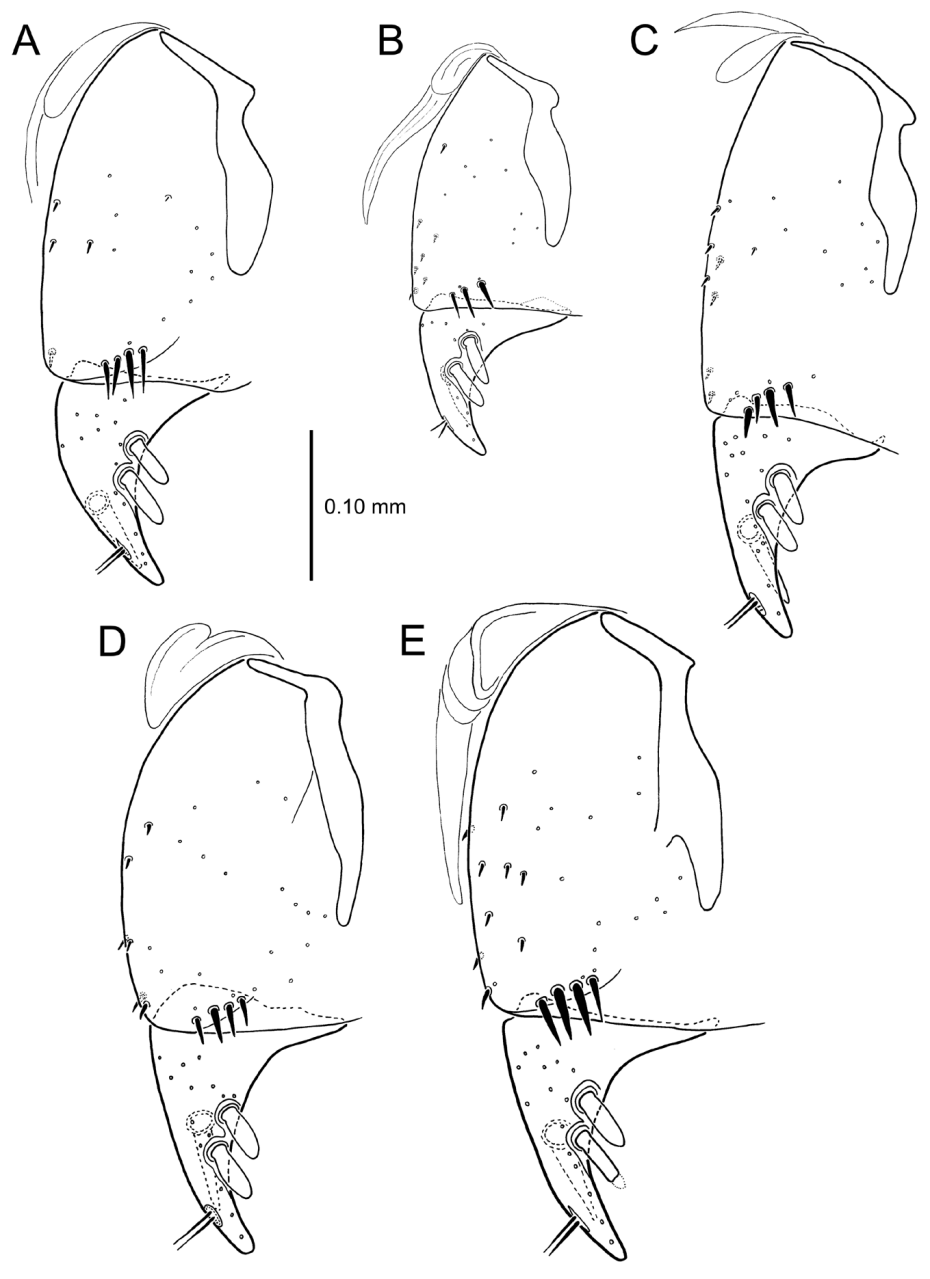
Left female gonocoxa, *Mecyclothorax sobrinus* group species, ventral view. **A** *Mecyclothorax multipunctatus* (nr. Ukulele Camp, 1615 m) **B** *Mecyclothorax inaequalis* (nr. Ukulele Camp, 1615 m) **C** *Mecyclothorax longulus* (nr. Ukulele Camp, 1534–1660 m) **D** *Mecyclothorax consobrinus* (Polipoli, 1890 m). **E** *Mecyclothorax sobrinus* (Honomanu, 1830–1860 m).

**Figure 56.**
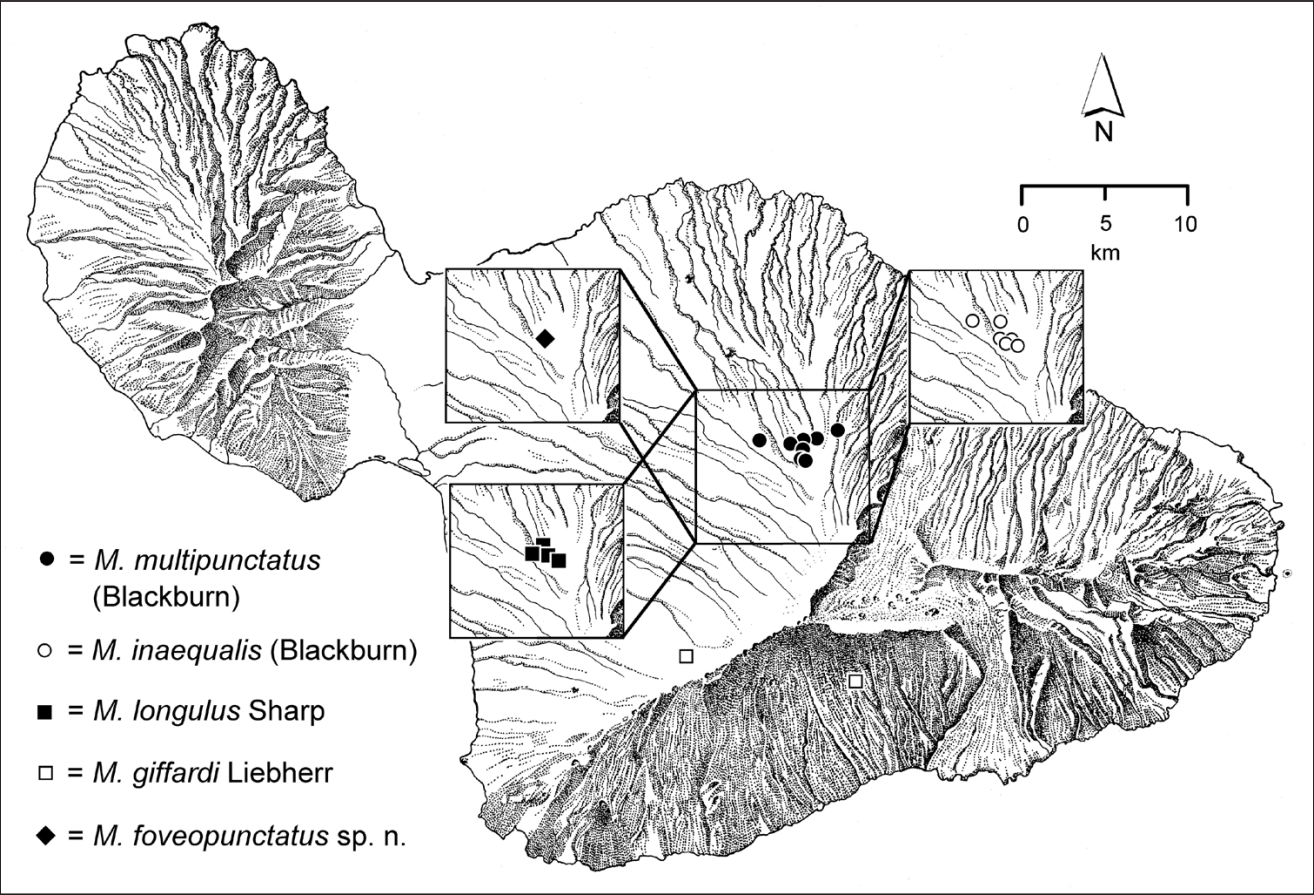
Recorded geographic distributions of *Mecyclothorax sobrinus* group species.

Figs 52A , 53A–C , 54A , 55A , 56

Cyclothorax multipunctatus Blackburn 1878a: 122; Blackburn and Sharp 1885: 214.
Mecyclothorax multipunctatus , Sharp 1903: 252; Britton 1948b: 156.

##### Diagnosis.

This species is uniquely diagnosed by the presence of 1–3 setae in interval 5 accompanying the usual two dorsal elytral setae in interval 2, and broad subquadrate elytra with intervals of consistent breadth that bear regular, transverse-mesh microsculpture. When only one supplementary 5^th^ interval seta is present, it is situated behind the position of the posterior dorsal elytral seta of the 3^rd^ interval (Fig. 52A). When two extra 5^th^ interval setae are present, they include the posterior trailing seta, plus a 2^nd^ seta situated between the positions of the anterior and posterior dorsal elytral setae. A third 5^th^ interval seta may be present, situated at a position immediately posterad or anterad the anterior dorsal elytral seta of interval 3. Setal formula 2 2 2 2. Standardized body length 4.5–5.5 mm.

##### Identification

(n = 5). The pronotum is transverse, MPW/PL = 1.32–1.42, with base broad, MPW/BPW = 1.41–1.44, and hind angles obtuse, with the lateral margins only slightly and briefly sinuate before the angles. The pronotal median base is slightly depressed relative to the disc, and covered with distinct longitudinal punctures and wrinkles. The elytra are broad basally, with the basal groove distinctly recurved to meet the lateral marginal depression at the subangulate humerus; MEW/HuW = 1.83–1.98. The sutural stria is deep with minute punctures to slight irregularities basally, deep and smooth apically. The mesepisternum bears ~8 punctures in 1–2 rows. The cuticular microsculpture is similar to all other species in the group, but the following combination is unique among those species: 1, vertex with upraised isodiametric sculpticells in transverse rows; 2, pronotal disc with transverse mesh, sculpticell breadth 2× length; 3, pronotal median base with granulate isodiametric mesh; 4, elytral disc covered with transverse mesh, sculpticell breadth 2× length; 5, elytral apex with transverse mesh, sculpticell breadth 3× length, to transverse lines.

**Male genitalia** (n = 4). Aedeagal median lobe moderately robust, distance from parameral articulation to tip 3.8× depth at midlength (Fig. 53A); apical extension beyond ostial opening with broadly convex dorsal projection, the tip slightly downturned and rounded; median lobe broad basally, thinly curved rightward apically in ventral view (Fig. 53C), the apex expanded slightly at tip; internal sac with ventral lobe at midlength (Fig. 53B; similar to ventral lobe of *Mecyclothorax arthuri*, Fig. 45H–I), the apical lobe bearing the flagellar plate smaller than the ventral lobe; flagellar plate short, the sclerotized ventral face 0.27× as long as parameral articulation-tip distance; sac with broad, diffuse ventral ostial microtrichial patch, otherwise covered with fine microspicules.

**Female reproductive tract** (n = 1). Bursa copulatrix columnar, broad with rounded apex, length 0.82 mm, breadth 0.43 mm, a heavily sclerotized plate dorsad the bursa copulatrix-common oviduct juncture, this hemi-elliptical bursal sclerite with both basal breadth and medial tarsomere length = 0.30 mm (Fig. 54A); bursal walls translucent with thick wrinkles; gonocoxite 1 with 3–4 apical fringe setae, a curved seta just basad medioapical angle and 3–5 smaller setae on medial surface (Fig. 55A); gonocoxite 2 subfalcate with a tightly rounded apex, broadly extended laterally at base, 2 lateral ensiform setae with apical seta broader and longer, apical nematiform setae on medioventral surface at 0.70× gonocoxite length.

##### Lectotype.

Female (BMNH) hereby designated, labeled: Mounting platen with Blackburn Maui code (Zimmerman 1957: 210), Cyc multipunc (on reverse) // Type // Hawaiian Is. Rev. T. Blackburn 1888-30. // LECTOTYPE Cyclothorax multipunctatus Blackburn J.K. Liebherr 1998 (black-margined red label).

##### Distribution and habitat.

*Mecyclothorax multipunctatus* is distributed in the forests of the Waikamoi and Honomanu drainages from 1210–1615 m elevation (Fig. 56). Most specimens have been collected in more mesic Koa-‘Ōhi‘a Forest, most often on the ground; under logs or in sifted leaf litter. It has also been found on koa trunks, or associated with *Cibotium* (hāpu‘u) tree ferns. On 7-v-1998, specimens of this species were collected from under large ohia logs in the mesic forest at Ukulele Pipeline along with *Mecyclothorax foveopunctatus* and *Mecyclothorax sobrinus* of this species group, plus the less closely related species *Mecyclothorax cognatus*, *Mecyclothorax consanguineus*, *Mecyclothorax cymindicus*, and *Mecyclothorax filipoides*.

#### (037) Mecyclothorax inaequalis

Taxon classificationAnimaliaColeopteraCarabidae

(Blackburn)

Figs 52B , 53D , 54B , 55B , 56

Cyclothorax inaequalis Blackburn 1878b: 157; Blackburn and Sharp 1885: 216.
Mecyclothorax inaequalis , Sharp 1903: 249; Britton 1948b: 142.

##### Diagnosis.

Individuals of this species are instantly recognizable by the lustrous, shimmery dorsal surface resulting from the well-developed isodiametric and transverse microsculpture, and the presence of elytral setae on intervals 3, 5, and 7 (Fig. 52B). The dorsal surface appears similar in reflective pattern to that of the fire-adapted *Sericoda* species (Liebherr 1991). In *Mecyclothorax inaequalis*, there are 4–6 setae on elytral interval 3, 3–5 setae on interval 5, and 3 on interval 7, each seta associated with partial convergence of the adjacent elytral striae. The dorsal microsculpture is arrayed as: 1, vertex with upraised isodiametric mesh, the sculpticells in transverse rows on the neck; 2, pronotal disc with upraised, slightly transversely stretched sculpticells in transverse rows, the median base with granulate isodiametric sculpticells, some in rows; 3, elytral disc with irregular, upraised isodiametric sculpticells plus a transverse mesh, sculpticell breadth 2× length, the apex with a shiny transverse mesh, sculpticell breadth 2–3× length. Setal formula 2 2 4 2. Standardized body length 3.9–4.7 mm.

##### Identification

(n = 5). The eyes are moderately convex, ocular ratio = 1.39–1.45, and situated on protruded ocular lobes, ocular lobe ratio = 0.73–0.79. The pronotum is moderately constricted basally, MPW/BPW = 1.45–1.52, and transverse, MPW/PL = 1.34–1.40. The pronotal hind angles are nearly right with an obtuse-rounded apex, and the lateral margins are subparallel for a short distance anterad the angles. The elytra are subquadrate, with rounded humeri extended laterally on the broadly rounded elytral base; MEW/HuW = 1.91–1.96. The variable dorsal reflective pattern belies monotonous dorsal coloration, with the vertex and pronotal disc brunneous with a slight piceous cast, the elytra rufobrunneous to brunneous, the apex slightly darker due to a piceous cast. Only the antennal base—antennomeres 1–3 and the base of 4—and legs deviate by their flavous coloration; the femora with a broad piceous cloud across their basal third, and the tibiae with an apically more developed piceous cast

**Male genitalia** (n = 1). Aedeagal median lobe gracile, distance from parameral articulation to tip 4.7× depth at midlength (Fig. 53D); apex broadly flat with subangulate ventral tip, blunt dorsal projection; internal sac with dark fields of spicules, flagellar plate short, 0.29× as long as parameral articulation-tip distance.

**Female reproductive tract** (n = 1). Bursa copulatrix columnar, elongate with rounded apex, length 0.91 mm, breadth 0.34 mm (Fig. 54B); bursal walls translucent with thin wrinkles basally, wrinkles thicker apically; gonocoxite 1 with 3 apical fringe setae and 6–7 smaller setae on medial surface (Fig. 55B); gonocoxite 2 falcate with tightly rounded apex, base broadly extended laterally, 2 lateral ensiform setae with apical seta longer and broader, apical nematiform setae on medioventral surface at 0.65× gonocoxite length.

##### Lectotype.

Male (BMNH) hereby designated, labeled: Mounting platen with Blackburn Maui code (Zimmerman 1957: 210), inaequalis (on reverse) // Type // Hawaiian Is. Rev. T. Blackburn 1888-30. // LECTOTYPE Cyclothorax inaequalis Blackburn J.K. Liebherr 1998 (black-margined red label).

##### Distribution and habitat.

*Mecyclothorax inaequalis* is a species recorded only from ground-level microhabitats in the mesic forests west of Waikamoi Gulch (Fig. 56), with collecting localities ranging 1210–1615 m elevation. Anonymous (N D) lists the species in Perkins’ lot 251: “all the small Carabids and Hem[e]iptera by grubbing.” In a modern exercise of the grubbing method on 14-v-1998 at Ukulele Pipeline, this species was collected along with individuals of *Mecyclothorax cognatus*, *Mecyclothorax longulus*, and *Mecyclothorax sobrinus*. In a subsequent grubbing attempt—16-v-2003 along the Sugi Ridge Trail, Waikamoi Nature Conservancy Preserve—the species list added *Mecyclothorax multipunctatus* and *Mecyclothorax perstriatus* to the above.

#### (038) Mecyclothorax longulus

Taxon classificationAnimaliaColeopteraCarabidae

Sharp

Figs 52C , 53E–F , 54C , 55C , 56

Mecyclothorax longulus Sharp 1903: 251; Britton 1948b: 143.

##### Diagnosis.

**Figure 57.**
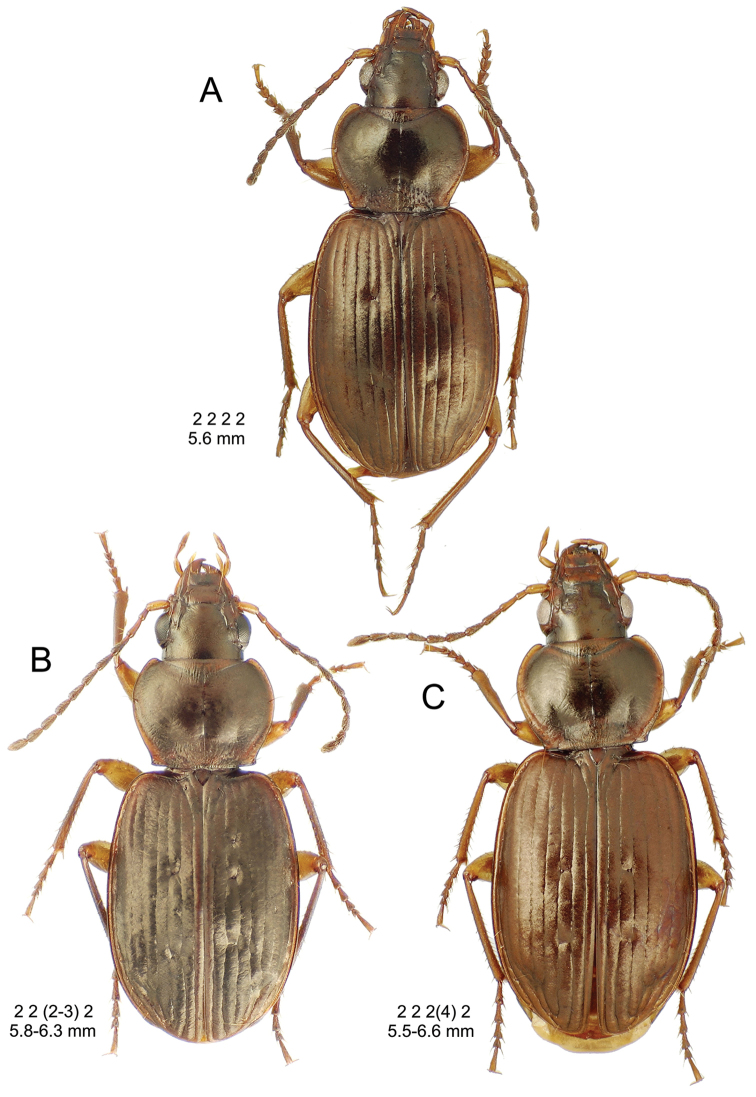
*Mecyclothorax sobrinus* group species, dorsal habitus view. **A** *Mecyclothorax foveopunctatus* (Ukulele Camp Pipeline, 1495–1525 m) **B** *Mecyclothorax consobrinus* (Polipoli, 1500 m) **C** *Mecyclothorax sobrinus* (Honomanu, 1830–1860 m).

It is the narrow, parallel-sided body shape (Fig. 52C) that diagnoses this species. The quadrate, elongate elytra exhibit only slightly convex lateral margins and relatively broad humeri, MEW/HuW = 1.83–1.93, and the pronotum is very broad relative to the elytra, MEW/MPW = 1.33–1.36. In this group, only *Mecyclothorax consobrinus* (Fig. 57B) and *Mecyclothorax sobrinus* (Fig. 57C) exhibit such quadrate elytra, but their body sizes are much larger; standardized body length for these two species spans 5.5–6.6 mm versus a standardized body length of 5.1–5.3 for *Mecyclothorax longulus*. Setal formula 2 2 2 2.

##### Identification

(n = 4). As above for the pronotum, head broad, MEW/MHW = 2.00–2.06, the eyes moderately convex, ocular ratio = 1.40–1.46. The lateral margins of the pronotum are only slightly sinuate anterad the obtuse, non-projected hind angles. The pronotal median base is indistinctly punctate, with 8–11 distinct rounded punctures or elongate longitudinal wrinkles each side, and the smooth laterobasal depression has a broad median tubercle. The discal elytral striae are minutely punctate, with associated intervals 1–5 slightly convex. The elytral setae consist of the parascutellar seta, 2 dorsal elytral setae at 0.33–0.35× and 0.60–0.62 and 0.60–0.62× elytral length, apical and subapical setae, and lateral setae arranged in an anterior series of 7 setae plus a posterior series of 6 setae. The dorsal microsculpture includes: 1, vertex with an upraised isodiametric mesh in transverse rows; 2, pronotal disc with upraised transverse mesh, sculpticell breadth 2× length, the median base with granulate isodiametric sculpticells intermixed with some transverse sculpticells; 3, elytral disc with an upraised isodiametric mesh, the apex with the isodiametric and slightly transversely stretched sculpticells in transverse rows.

**Male genitalia** (n = 1). Aedeagal median lobe gracile, distance from parameral articulation to tip 4.3× depth at midlength (Fig. 53E); apex extended 3× its depth beyond ostial opening, apex expanded dorsoventrally with ventral margin convex before rounded tip; median lobe distinctly curved to right in ventral view distad apex of ostial opening (Fig. 53F); internal sac covered with dark fields of microspicules, short flagellar plate visible inside dorsal margin of lobe, length 0.26× parameral articulation-tip distance.

**Female reproductive tract** (n = 1). Bursa copulatrix columnar, short and broad, the dimensions dictated by a heavily sclerotized, hemi-elliptical plate dorsad bursa copulatrix-median oviduct juncture, bursal length 0.48 mm, breadth 0.40 mm, the same dimensions as bursal sclerite (Fig. 54C); bursal walls translucent with thin wrinkles where not sclerotized into bursal sclerite; gonocoxite 1 with 3–4 apical fringe setae, 6–7 smaller setae on medial surface (Fig. 55C); gonocoxite 2 subfalcate with tightly rounded apex, 2 lateral ensiform setae, apical nematiform setae on medioventral surface at 0.77× gonocoxite length.

##### Holotype.

Male (BMNH) labeled: Mecyclothorax longulus Type D.S. Haleakala Perkins 120 // Type // Hawaiian Is. Perkins 1904-336. // Haleakala Maui 5000 ft. 6 IV 1894 // HOLOTYPE Mecyclothorax longulus Sharp J.K. Liebherr 1998 (black-margined red label).

##### Distribution and habitat.

*Mecyclothorax longulus* is known from Koa-‘Ōhi‘a Mesic Forest across a very limited geographic area west of Pu‘u o Kakae—elevations 1425–1615 m—in the Waikamoi area (Fig. 56). It was described by Sharp (1903) from a unique specimen from Ukulele Camp, and it has been recollected on four occasions since. It has been found in leaf siftate, by grubbing in leaf litter, under the rotten bark of *Cheirodendron* (‘ōlapa), and in a yellow-pan trap.

#### (039) Mecyclothorax giffardi

Taxon classificationAnimaliaColeopteraCarabidae

Liebherr

Figs 52D , 53G , 56

Mecyclothorax giffardi Liebherr 2005b: 108.

##### Diagnosis.

Of the beetles comprising this taxonomic group, only individuals of this species simultaneously exhibit: 1, two dorsal elytral setae on interval 3 but no additional setae on intervals 5 or 7; 2, laterally convex elytra with the greatest width behind midlength in combination with slightly narrowed humeri, MEW/HuW = 1.98; and 3, moderately smaller body size, standardized body length 4.5–4.9 mm. The dorsal body surface is reflective due to largely transverse microsculpture: 1, vertex and pronotal disc covered with a distinct transverse mesh, sculpticell breadth subequal to twice sculpticell length; and 2, elytra with a transverse mesh, sculpticell breadth 2–3× length. Only the pronotal median base exhibits the upraised isodiametric sculpticells–irregularly swirling based on the orientation of the cuticular surface–characteristic of other species in the group. Setal formula 2 2 2 2.

##### Identification

(n = 2). The eyes are smaller, covering only ¾ of the protruded ocular lobe, and narrowly convex, ocular ratio = 1.41–1.42. The forebody is narrower relative to the elytra than in the other species with only the 2 dorsal elytral setae; MEW/MWH = 2.14, MEW/MPW = 1.46. The discal elytral striae are minutely punctate, and the dorsal elytral setae are in broad, foveate depressions that span interval 3 plus the adjacent halves of intervals 2 and 4. The forebody is distinctly darker than the elytra, with frons and vertex plus pronotal disc rufopiceous, contrasted to the rufobrunneous elytra. The apex of the elytral sutural stria and the adjoining elytral apex are paler, rufoflavous. Like related species, the femora are flavous with a basal piceous cloud, and the tibiae are rufobrunneous with a more pronounced piceous cast apically.

**Male genitalia** (n = 1). Aedeagal median lobe gracile, distance from parameral articulation to tip 4.1× depth at midlength (Fig. 53G); apex broadly expanded with flat apical face, the ventral tip rightly rounded; internal sac short and broad, with broad dorsal ostial microtrichial patch at base, and ventral surface broadly spiculate as a ventral patch; flagellar plate short, length 0.30× parameral articulation-tip distance.

##### Holotype.

Male (BPBM) dissected and designated by Liebherr (2005b: 109). Type locality is: HI: Maui, Haleakalā, Polipoli Springs area, 5000 ft. el.

##### Distribution and habitat.

*Mecyclothorax giffardi* is known from only two specimens, the holotype collected by W.M. Giffard at 1525 m elevation in the Kula Forest Reserve below Polipoli Springs, and a second male specimen collected by P.D. Krushelnycky in the Kahikinui Forest Reserve on Haleakalā’s south slope (Fig. 56). The Kahikinui specimen was collected in koa-‘ōhi‘a leaf litter.

#### (040) Mecyclothorax foveopunctatus sp. n.

Taxon classificationAnimaliaColeopteraCarabidae

http://zoobank.org/F8151F07-D502-4FC2-8B26-58A66D9864F3

Figs 53H , 56 , 57A

##### Diagnosis.

**Figure 58.**
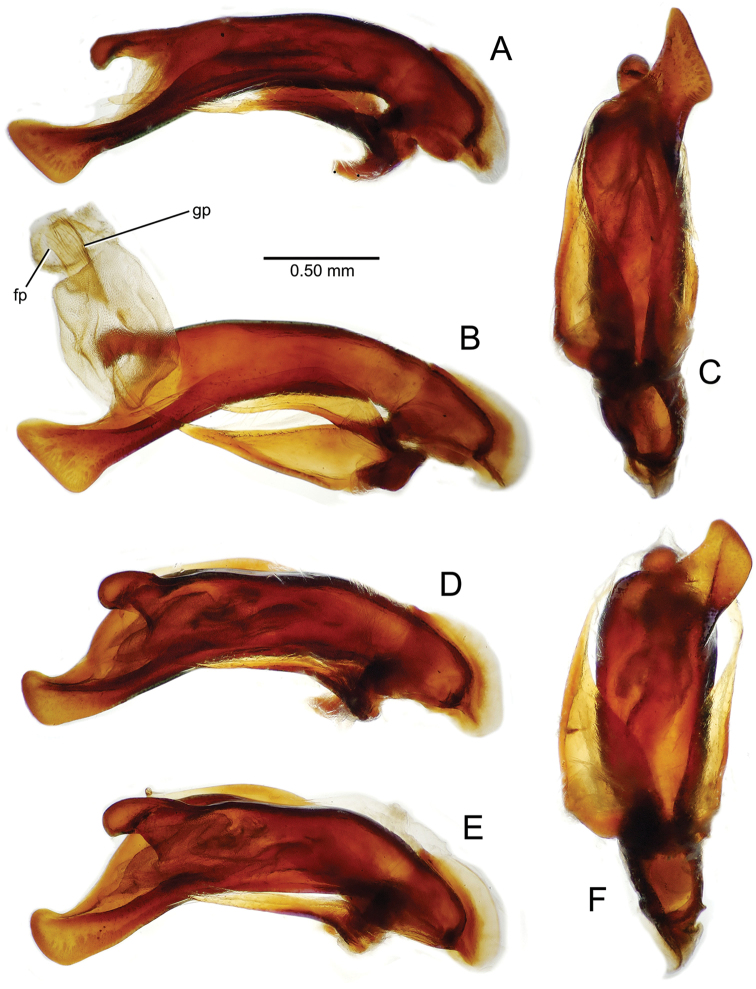
Male aedeagus, *Mecyclothorax sobrinus* group species (for abbreviations see Table 2, p. 23). **A–C** *Mecyclothorax consobrinus*. **A** Right view (Polipoli, 1890 m) **B** Right view, sac everted (Polipoli, 1500 m) **C** Ventral view (Polipoli, 1890 m) **D–F** *Mecyclothorax sobrinus* **D** Right view (Ukulele Camp Pipeline, 1540 m) **E** Right view (nr. Ukulele Camp, 1615 m) **F** Ventral view (Ukulele Camp Pipeline, 1540 m).

This along with *Mecyclothorax sobrinus* and *Mecyclothorax consobrinus* represent the large-bodied species of the group; in this species standardized body length 5.6 mm. This species differs from the other two by the basally more constricted elytra (Fig. 57), and more punctate discal elytral striae, the striae themselves deeper and more regular. The pronotal median base is more discretely punctate, with ~ 6 distinct, rounded punctures plus 3–4 longitudinal wrinkles each side. The male aedeagal median lobe (Fig. 53H) lacks the robust, accessory subapical projection shared by males of *Mecyclothorax consobrinus* and *Mecyclothorax sobrinus* (Fig. 58). Setal formula 2 2 2 2.

##### Description

(n = 1). *Head capsule* with frontal grooves deep near clypeus, angled laterally at midpoint to terminate mesad anterior supraorbital setae; dorsal impression of neck slightly concave; eyes moderate in size and convexity, ocular ratio = 1.44, ocular lobe ratio = 0.76; labral anterior margin moderately emarginate; antennae filiform, antennomeres 2–3 with sparse pelage of short setae; mentum tooth with sides right, apex broadly rounded. *Pronotum* transverse, MPW/PL = 1.33, broad basally, MPW/BPW = 1.36, hind angles obtuse, little projected, with lateral margins subparallel for short distance anterad angles; basal margin straight medially, extended slightly between laterobasal depressions; median longitudinal impression shallow, finely incised at depth, extended onto median base; anterior transverse impression deep, finely incised at depth, anterior callosity elevated, flat, both crossed by longitudinal wrinkles; front angles projected, rounded; pronotal base broader than apex, APW/BPW = 0.90; lateral marginal depression broad at front angle, moderately narrow at midlength, broadened near laterobasal depression, edge upturned; laterobasal depression broad, smooth, with median tubercle. *Proepisternum* with 5 minute punctures along hind marginal groove; prosternal process medially depressed, margin upraised with bead only anterad procoxae. *Elytra* subovoid, disc flat, sides abruptly sloped; basal groove slightly recurved to subangulate humeral angle; humeri narrowed, MEW/HuW = 1.90, lateral margin narrowly curved posterad outside angle; parascutellar seta present; parascutellar striole smooth anteriorly, 3 punctures posteriorly near apex; sutural interval of same convexity as lateral intervals basally, upraised as a callous apically; sutural and 2^nd^ striae of subequal depth from base to apex; 8^th^ interval convex laterad 7^th^ stria near subapical sinuation, 7^th^ stria and nearby portion of 7^th^ interval depressed just apicad diminution of interval 6 at fusion of intervals 5 + 7; 2 dorsal elytral setae at 0.34× and 0.64–0.67× elytral length, setae in foveate depressions that span interval 3 plus lateral half of interval 4; apical and subapical setae present; lateral elytral setae arranged in anterior series of 7 setae, posterior series of 6 setae; elytral marginal depression moderately narrow throughout length, beadlike near subapical sinuation. *Mesepisternum* with ~18 punctures in 3 rows; metepisternal width to length ratio 0.86. *Abdomen* with irregular lateral wrinkles on ventrites 1–5; suture between ventrites 2 and 3 complete; apical male ventrite with 2 marginal setae. *Legs*-metatarsomere 1/metatibial length ratio = 0.18; metatarsomere 4 length along outer lobe 1.3× medial tarsomere length, apical and subapical setae present; metatarsal dorsolateral sulci moderately deep, basal tarsomeres medially convex. *Microsculpture* of vertex upraised isodiametric sculpticells in transverse rows; pronotal disc with isodiametric sculpticells in transverse rows to a transverse mesh, sculpticell breadth 2× length, median base with granulate isodiametric mesh; elytral disc with upraised isodiametric sculpticells, the apex with upraised transverse mesh, sculpticell breadth 2× length; metasternum with distinct transverse mesh; laterobasal abdominal ventrites with swirling transverse and isodiametric microsculpture. *Coloration* of vertex dark rufobrunneous; antennomere 1 flavous, antennomeres 2–3 rufoflavous, 4–11 rufobrunneous; pronotal disc dark rufobrunneous, margins rufoflavous mesad front angles, lateral marginal depressions and median base rufobrunneous; proepipleuron rufoflavous, proepisternum rufopiceous; elytral disc rufobrunneous, sutural interval concolorous basally, slightly paler, rufoflavous apically; elytral marginal depression and apex slightly paler than disc, rufoflavous; elytral epipleuron flavous dorsally, rufobrunneous on ventral margin, metepisternum piceous; abdomen piceous, apical 1/5 of apical ventrite 6 flavous; metafemur with ground color flavous, basal half with broad piceous cloud; metatibia rufoflavous with piceous cast.

**Male genitalia** (n = 1). Aedeagal median lobe (Fig. 53H) much like a more robust version of *Mecyclothorax longulus* (Fig. 53E), gracile, distance from parameral articulation to tip 4.1× depth at midlength, apex extended 3× depth beyond apex of ostial opening, expanded dorsoventrally at tip, with apical face of tip obliquely flattened; internal sac unornamented, flagellar plate short, length 0.30× parameral articulation-tip distance.

##### Holotype.

Male (CUIC) dissected and labeled: HI: Maui Haleakala / Waikamoi N.C.P. Ukulele / Pipeline 7-V-1998 lot 03 / 1495-1525m el. under / logs J.K. Liebherr // Hawaiian Is. / Perkins / 1904–336. / HOLOTYPE / Mecyclothorax / foveopunctatus / Liebherr / det. J.K. Liebherr 2015 (black-margined red label).

##### Etymology.

The Latin adjectival foveopunctatus signifies the foveate depressions surrounding the dorsal elytral setae.

##### Distribution and habitat.

*Mecyclothorax foveopunctatus* is known only from the holotype collected under an ‘ōhi‘a log in mesic forest near Ukulele Pipeline (Fig. 56). The type specimen was collected along with specimens of *Mecyclothorax cognatus*, *Mecyclothorax consanguineus*, *Mecyclothorax cymindicus*, *Mecyclothorax filipoides*, *Mecyclothorax multipunctatus*, and *Mecyclothorax sobrinus*.

#### (041) Mecyclothorax consobrinus

Taxon classificationAnimaliaColeopteraCarabidae

Liebherr

**Figure 59.**
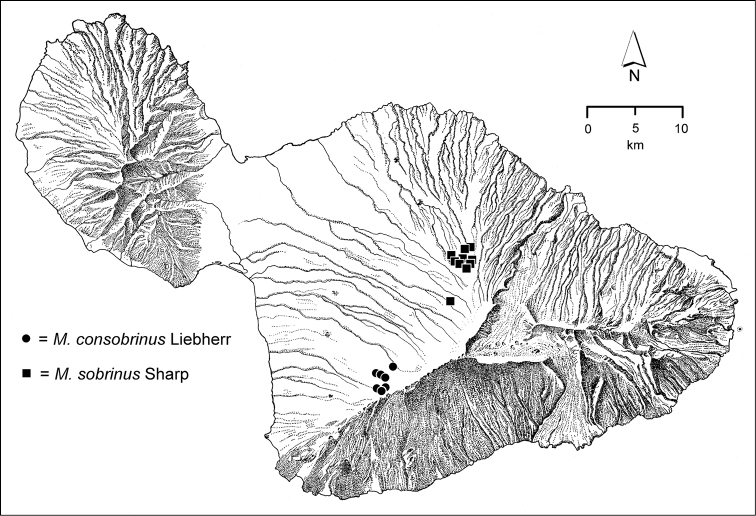
Recorded geographic distributions of *Mecyclothorax sobrinus* group species.

Figs 54D , 55D , 57B , 58A–C , 59

Mecyclothorax consobrinus Liebherr 2005b: 101.

##### Diagnosis.

This and *Mecyclothorax sobrinus* share the conditions of subquadrate elytra with dorsal setae restricted to interval 3 (Fig. 57B–C), and large body size; in this species standardized body length 5.8–6.3 mm. The two species are considered adelphotaxa based on shared possession of a very distinctive male aedeagal median lobe that exhibits a robust, apically directed process situated immediately basad the ostial opening (Fig. 58). *Mecyclothorax consobrinus* can be diagnosed by the more developed dorsal microsculpture: 1, vertex with upraised isodiametric sculpticells in transverse rows; 2, pronotal disc with upraised slightly transversely stretched sculpticells, pronotal median base with granulate isodiametric and transverse sculpticells; 3, elytral disc with upraised isodiametric mesh, the apex with isodiametric mesh and swirling transverse mesh in large depression associated with 7^th^ stria. The elytra are also more irregularly depressed in this species, in some individuals due to presence of a third dorsal elytral seta in interval 3 (Fig. 57B), and in all individuals due to the deep and irregular depression associated with the fused apical portion of striae 5 + 7, this depression in some instances also involving the apical termination of interval 6. Setal formula 2 2 2-3 2.

##### Identification

(n = 5). The elytral striae are shallow, with the surfaces of the discal intervals irregularly undulated along their length. These undulations in concert with the upraised microsculpture lead to a satiny appearance to the elytral cuticle. The pronotal median base tends to have an irregular surface as well, due to the presence of distinct punctures in company with many longitudinal wrinkles. The mesepisternum is profoundly punctate in this species, with ~22 punctures arrayed in 2–3 longitudinal rows across its surface. Access to a male will cement the identification of male members of a series representing this species (Fig. 58A–C) (see below).

**Male genitalia** (n = 2). Aedeagal median lobe heavily sclerotized yet shaft dimensions gracile relative to length, distance from parameral articulation to tip 4.6× depth at midlength (Fig. 58A); apex broadly flattened along apical face, adze-shaped, along with large dorsal subapical projection at basal margin of ostium (a character shared only with *Mecyclothorax sobrinus*; Fig. 58D–E) that results in a paired structure surrounding the ostium that appears not unlike a bottle opener; median lobe with apex curved rightward in ventral view, the dorsal subapical projection curved leftward (Fig. 58C); internal sac very small, short, length 0.47× parameral articulation-tip distance, sac surface unornamented; flagellar plate very small, sclerotized ventral surface 0.24× parameral articulation-tip distance.

**Female reproductive tract** (n = 1). Bursa copulatrix broad, columnar, apex rounded, ventral surface dorsad bursa copulatrix-common oviduct juncture with brownish coloration, lightly sclerotized, length 0.95 mm, breadth 0.46 mm (Fig. 54D); bursal walls translucent with thick wrinkles; gonocoxite 1 with 3–4 apical fringe setae, 1–2 thick, curved setae at apicomedial angle, and 4–5 smaller setal on medial surface (Fig. 55D); gonocoxite 2 narrowly subtriangular with narrowly rounded apex, base broadly extended laterally, 2 lateral ensiform setae, apical nematiform setae on medioventral surface at 0.65× gonocoxite length.

##### Holotype.

Male (BPBM) designated by Liebherr (2005b: 107). Type locality is HI: Maui, Haleakala, Polipoli Springs area, 5000 ft. el.

##### Distribution and habitat.

*Mecyclothorax consobrinus* is known only from the Polipoli Springs area along the southwest rift of Haleakalā (Fig. 59). It has been collected from leaf litter, the sift samples often taken from areas covered with *Dryopteris wallichiana* (Laukahi) ferns. It is also commonly found under stones on moist ground, and one specimen was found under loose *Pinus radiata* bark.

#### (042) Mecyclothorax sobrinus

Taxon classificationAnimaliaColeopteraCarabidae

Sharp

Figs 54E , 55E , 57C , 58D–F , 59

Mecyclothorax sobrinus Sharp 1903: 253; Britton 1948b: 143.

##### Diagnosis.

Individuals of this species exhibit overlapping head, prothoracic, and elytral ratios and standardized body length—5.5–6.6 mm—with *Mecyclothorax consobrinus*, reducing any diagnosis of the two species to qualitative characters associated with elytral setation and punctation, and cuticular microsculpture. The discal elytral striae are deeper and of more uniform depth in this species than in *Mecyclothorax consobrinus*. Striae 1–6 are closely punctured in the basal 2/3 of their length. The setal impressions associated with the two dorsal elytral setae are shallower and less broad than seen in *Mecyclothorax consobrinus*, depressing interval 3 plus less than half of interval 4. Also, the apical depression associated with interval 7 is shallower and more regularly depressed among individuals of this species. Finally, the microsculpture is less upraised overall: 1, vertex with well-developed isodiametric sculpticells in transverse rows; 2, pronotal disc with well-developed slightly transversely stretched mesh, the median base covered with a mixture of isodiametric and transverse sculpticells; 3, elytral disc with a transverse mesh, sculpticell breadth 2× length, apex with mesh more transverse, breadth 2–3× length. Also, no individuals representing this species have been observed to have more than two dorsal elytral setae. Setal formula 2 2 2 2.

##### Identification

(n = 5). Although the ratios overlap with *Mecyclothorax consobrinus*, eyes tend to be less developed in individuals of this species; ocular ratio = 1.37–1.46 versus 1.41–1.46 in *Mecyclothorax consobrinus*, ocular lobe ratio = 0.71–0.77 versus 0.74–0.76. The pronotal median base is relatively smooth, with only sparse fine punctures and a few longitudinal wrinkles. The pronotal lateral marginal depression is only slightly broader at the front angles, the angles tightly rounded. The mesepisternum is moderately punctate, with ~9 punctures in 1–2 longitudinal rows. Finally, the male median aedeagal lobe has a less developed dorsal projection and narrower, more rounded apex (Fig. 58D–F) than that of the adelphotaxon’s males.

**Male genitalia** (n = 2). Aedeagal median lobe (Fig. 58D) a slightly less exaggerated version than that characterizing *Mecyclothorax consobrinus* (Fig. 58A), distance from parameral articulation to tip 4.2× depth at midlength, apex broadly rounded in company with blunt, broad dorsal projection, the dorsal projection varying in curvature and apical shape (Fig. 58D–E), apex of median lobe curved rightward, dorsal projection curved leftward (Fig. 58F).

**Female reproductive tract** (n = 1). Bursa copulatrix broad, columnar apex rounded, ventral surface dorsad bursa copulatrix-common oviduct juncture with brownish coloration, lightly sclerotized, bursal length 1.0 mm, breadth 0.51 mm (Fig. 54E); bursal walls translucent with thick wrinkles outside area of sclerotization; gonocoxite 1 with 3–4 apical fringe setae, thicker apicomedial seta present at apical angle, 7–8 smaller setae basally along median margin (Fig. 55E); gonocoxite 2 narrowly subtriangular with tightly rounded apex, base broadly extended laterally, 2 lateral ensiform setae, apical nematiform setae on medioventral surface at 0.71× gonocoxite length.

##### Lectotype.

Male (BMNH) hereby designated, labeled: Mecyclothorax sobrinus Type D.S. Haleakala Perkins 350 // Type // Hawaiian Is. Perkins 1904-336. // Haleakala Maui 4500 ft. Perkins 28 III 1894 // LECTOTYPE Mecyclothorax sobrinus Sharp J.K. Liebherr 1998 (black-margined red label).

##### Distribution and habitat.

*Mecyclothorax sobrinus* inhabits mesic forest west of Waikamoi Gulch (Fig. 59) from 1280–1980 m elevation. E.C. Zimmerman collected two specimens by beating at 6000–6500 ft. elevation on the NW upper slope; these specimens representing outliers to modern collections in the Koa-‘Ōhi‘a Mesic Forest near Ukulele Pipeline. Forest-inhabiting specimens have been found in sifted litter associated with ‘ōhi‘a and *Cibotium* (hāpu‘u) tree fern. The largest collections have come from yellow-pan traps set in ecotone forest situations (vi-viii-2006, L. Leblanc, UHIM).

### *Mecyclothorax ovipennis* species group

**Diagnosis.** Species classified in this group are characterized by: 1, lateral elytral striae, at least interval 7, reduced in depth relative to the more medial striae; 2, the sutural and 2^nd^ striae of subequal depth at the elytral apex; and 3, eyes well developed, the outer surface convex. This last character is broadly defined, with ocular ratios in Haleakalā species ranging 1.41–1.61, and ocular lobe ratios spanning 0.75–0.89. Beetle bodies of the included species appear gracile, with a basally constricted pronotum and gracile legs. Body size is small to moderate; standardized body length 3.3–4.9 mm. The elytra vary in shape, from subquadrate, to subovoid, to ellipsoid. Dorsal microsculpture also varies dramatically among the species placed here, with elytral disc microsculpture, for example, ranging from a distinct or shallow isodiametric mesh to a transverse mesh, to even a glossy surface with microsculpture apparent only on the lateral elytral intervals. As per the characters listed above, there is great disparity in setal formula across this assemblage, including 2 2 2 2, 2 2 2 1, 2 1 2 1, 2 1 2 0, and 2 1 1 0. When there is only one seta at the elytral apex, it may be the subapical seta or the apical seta.

**Membership and distribution.** This group comprises 37 species from across Maui Nui and Hawai‘i Island, or 15% of the entire Hawaiian *Mecyclothorax* fauna. The group is represented on Maui with the 19 Haleakalā species treated below plus four others in West Maui (Liebherr 2011). Species representation on the other islands include nine species on Moloka‘i (Liebherr 2007), one species on Lāna‘i (Liebherr 2009b), and four species on Hawai‘i Island (Liebherr 2008b). Species on different islands are characterized by identical setal formulae—e.g., West Maui, Haleakalā, and Hawai‘i house species with 2 2 2 2, and on Haleakalā and Moloka‘i reside species exhibiting 2 2 2 1[ae] and 2 1 2 0 setal patterns. Two of these species include the cryptic sibling species pair *Mecyclothorax ovipennis* Sharp of Haleakalā and *Mecyclothorax ferovipennis* Liebherr of West Maui, both of which fit the 2 2 2 2 setal formula. Conversely, the only three species characterized by the setal formula 2 1 2 2 reside in West Maui; *Mecyclothorax exilioides* Liebherr, *Mecyclothorax allostriatus* Liebherr, and *Mecyclothorax geminatus* Liebherr. To the degree that setal formula is congruent with phylogenetic relationships—in the triplet above, *Mecyclothorax allostriatus* and *Mecyclothorax geminatus* are cryptic sibling species—the diversification history of *Mecyclothorax ovipennis* group species was biogeographically complex, involving both within- and between-volcano speciation events. Therefore elucidating their collective history will require comprehensive phylogenetic analysis.

#### Key to adults of the *Mecyclothorax ovipennis* species group, Haleakalā volcano, Maui, Hawai‘i

**Table:** 

1	Elytral disc piceous, contrasted with flavous lateral intervals 7–9 or 8–9, the lateral flavous band extended from humerus to apex (Fig. 60A–C)	**2**
1’	Elytral intervals 2–9 concolorous basally, rufous to piceous, with at most apex contrastedly flavous, lateral marginal depression outside stria 9 may be flavous (Figs 60D, 65, 68, 73, 78)	**4**
2(1)	Pronotal hind angles slightly obtuse due to rounded hind margin, lateral margins divergent very close to angles (Fig. 60B–C); vertex with shallow transverse-mesh microsculpture, the surface glossy	**3**
2’	Pronotal hind angles right, lateral margins parallel before angles (Fig. 60A); vertex with distinct isodiametric sculpticells arranged in transverse rows	(043) ***Mecyclothorax subtilis* Britton & Liebherr, sp. n.**
3(2)	Elytra narrowly flavous marginally, intervals 8–9 flavous versus piceous disc (Fig. 60B); discal elytral intervals with shallow transverse-mesh microsculpture, the sculpticells not visible in areas of reflected light, the surface appearing glossy	(044) ***Mecyclothorax patulus* sp. n.**
3’	Elytra more broadly flavous marginally, interval 6 slightly fuscous and intervals 7-9 flavous (Fig. 60C); discal elytral intervals with evident transverse-mesh microsculpture, the sculpticells visible in areas of reflected ligh	(045) ***Mecyclothorax patagiatus* sp. n.**
4(1)	Elytral striae 3–5 impunctate, discal striae may be developed or evanescent, and striae 1–2 may be indistinctly punctate near base	**5**
4’	Elytral striae 3–5 indistinctly to markedly punctate on disc, striae may be well developed or evanescent, if evanescent always indicated by a line of punctures	**9**
5(4)	Pronotal lateral margins parallel or convergent anterad hind angles which are right to acute (Figs 60D, 65A)	**6**
5’	Pronotal lateral margins divergent anterad hind angles which are obtuse (Figs 65B–D, 68, 73, 78)	**7**
6(5)	Elytra narrow relative to forebody, MEW/MHW = 1.96–2.0; elytral basal groove recurved anteriorly to join lateral marginal depression, the humerus subangulate (Fig. 60D)	(046) ***Mecyclothorax strigosus* sp. n.**
6’	Elytra broad relative to forebody, MEW/MHW = 2.08–2.24; elytral basal groove not recurved anteriorly, juncture with lateral marginal depression rounded (Fig. 65A)	(047) ***Mecyclothorax ovipennis* Sharp**
7(6)	Vertex with evident isodiametric to transverse-mesh microsculpture, the sculpticell margins visible in reflected light	**8**
7’	Vertex and pronotal disc with obsolete transverse-mesh microsculpture, the surface glossy in reflected light	(048) ***Mecyclothorax takumiae* sp. n.**
8(7)	Pronotal base moderately constricted, MPW/BPW = 1.46–1.54; elytral basal groove evenly curved at humerus, elytral lateral marginal depression narrow posterad humerus	(049) ***Mecyclothorax apicalis* (Sharp)** (in part)
8’	Pronotal base broad, MPW/BPW = 1.41–1.45; elytral basal groove curved anterad at humerus, the juncture of basal groove and lateral marginal depression indicated by a hitch, elytral lateral marginal depression broader, flavous posterad humerus	(050) ***Mecyclothorax parapicalis* sp. n.**
9(4)	Elytra broadly ellipsoid, lateral margins markedly convex and humeri narrowly rounded, MEW/HuW = 2.23–2.51 (Figs 68A–B)	**10**
9’	Elytra broader basally, lateral margins subparallel to convex, but humeri not constricted, MEW/HuW = 1.84–2.18 (Figs 68C, 73, 78)	**11**
10(9)	Vertex and pronotal disc with evident transverse-mesh microsculpture, sculpticell breadth 2–3× length; male aedeagal median lobe apex broadly rounded (Fig. 69)	(051) ***Mecyclothorax mauiae* sp. n.**
10’	Vertex with elongate transverse-mesh microsculpture, sculpticell breadth 3–4× length, pronotal disc covered with indistinct transverse microsculpture, much of the surface glossy; male aedeagal median lobe apex narrow with pointed tip (Fig. 70A–C)	(052) ***Mecyclothorax subternus* sp. n.**
11(9)	Pronotal lateral margins parallel or convergent anterad right or acute hind angles (Figs 68C, 73A–C)	**12**
11’	Pronotal lateral margins divergent anterad obtuse hind angles (Figs 73D, 78)	**15**
12(11)	Eyes moderately convex, ocular ratio = 1.41–1.50 (Fig. 73A–C); elytra ellipsoid, lateral margins more evenly curved so that elytra are widest at middle or in anterior half; abdominal ventrites 4–6 concolorous with or only slightly paler than basal ventrites	**13**
12’	Eyes convex, ocular ratio = 1.51–1.56 (Fig. 68C); elytra obovoid, widest behind middle; abdominal ventrites 4–6 flavous, contrasted to piceous basal ventrites	(053) ***Mecyclothorax flaviventris* sp. n.**
13(12)	Elytra broad, lateral margins convex (Fig. 73B–C), MEW/HuW = 2.11–2.16; discal elytral intervals with transverse-mesh microsculpture	**14**
13’	Elytra narrow, elongate (Fig. 73A), MEW/HuW = 2.02–2.06; discal elytral intervals with well-developed isodiametric microsculpture	(054) ***Mecyclothorax laetus* Sharp**
14(13)	Elytral striae 1–2 moderately impressed, striae 3-6 progressively shallower, strial punctation indistinct especially on lateral striae (Fig. 73B); male aedeagal median lobe apex short and broad, with minute nipplelike protuberance (Fig. 76A)	(055) ***Mecyclothorax cordaticollis* (Blackburn)**
14’	Elytral striae 1–4 impressed, distinctly punctate, striae 5–6 shallower with less evident punctures (Fig. 73C); male aedeagal median lobe narrowly extended distad ostium, the narrowly rounded tip bent downward (Fig. 76C)	(056) ***Mecyclothorax cordaticollaris* sp. n.**
15(11)	Pronotal lateral seta present, basal seta absent, the hind angles glabrous	**16**
15’	Both pronotal lateral and basal setae present, the pronotum quadrisetose (if basal seta appears absent, articulatory socket will be visible)	**17**
16(15)	Vertex with well-developed isodiametric sculpticells arranged in transverse rows, pronotal disc with isodiametric to slightly transverse sculpticells in transverse rows; pronotal base broader, MPW/BPW = 1.46–1.54 (Fig. 65C)	(049) ***Mecyclothorax apicalis* (Sharp)** (in part)
16’	Vertex with shallow transverse-mesh microsculpture, sculpticell breadth 2–3× length, pronotal disc with obsolete transverse-mesh microsculpture, glossy medially; pronotal base narrower, MPW/BPW = 1.58–1.69 (Fig. 73D)	(057) ***Mecyclothorax subconstrictus* (Sharp)**
17(15)	Pronotal hind angle defined by small but distinct toothlike projection, the lateral margin briefly sinuate before the obtuse angle (Fig. 78B–D)	**18**
17’	Pronotal hind angles rounded, not projected (Fig. 78A)	(058) ***Mecyclothorax nubicola* (Blackburn)**
18(17)	Pronotal base broader (Fig. 78C–D), MPW/BPW = 1.42–1.51; elytra subquadrate, lateral margins straighter posterad humeri	**19**
18’	Pronotal base narrower (Fig. 78B), MPW/BPW = 1.54; elytra subellipsoid, lateral margins evenly convex posterad the broadly rounded humeri	(059) ***Mecyclothorax krushelnyckyi* sp. n.**
19(18)	Vertex with shallow but evident transverse-mesh microsculpture, sculpticell breadth 2–3× length; elytra more quadrate, MEW/HuW = 1.84–1.90; elytral striae not impressed, indicated by series of isolated punctures	(060) ***Mecyclothorax pusillus* Sharp**
19’	Vertex with obsolete transverse-mesh microsculpture, the surface glossy; elytra subquadrate, MEW/HuW = 1.98–2.19; elytral striae 1–4 impressed, the punctures connected by striae, stria 5 consisting of isolated punctures	(061) ***Mecyclothorax rusticus* Sharp**

#### (043) Mecyclothorax subtilis

Taxon classificationAnimaliaColeopteraCarabidae

Britton & Liebherr sp. n.

http://zoobank.org/6B5112DF-97C4-4300-9033-808C9DC9331E

Figs 60A , 64

Mecyclothorax n. sp. α, Liebherr 2004, fig. 4.

##### Diagnosis.

**Figure 60.**
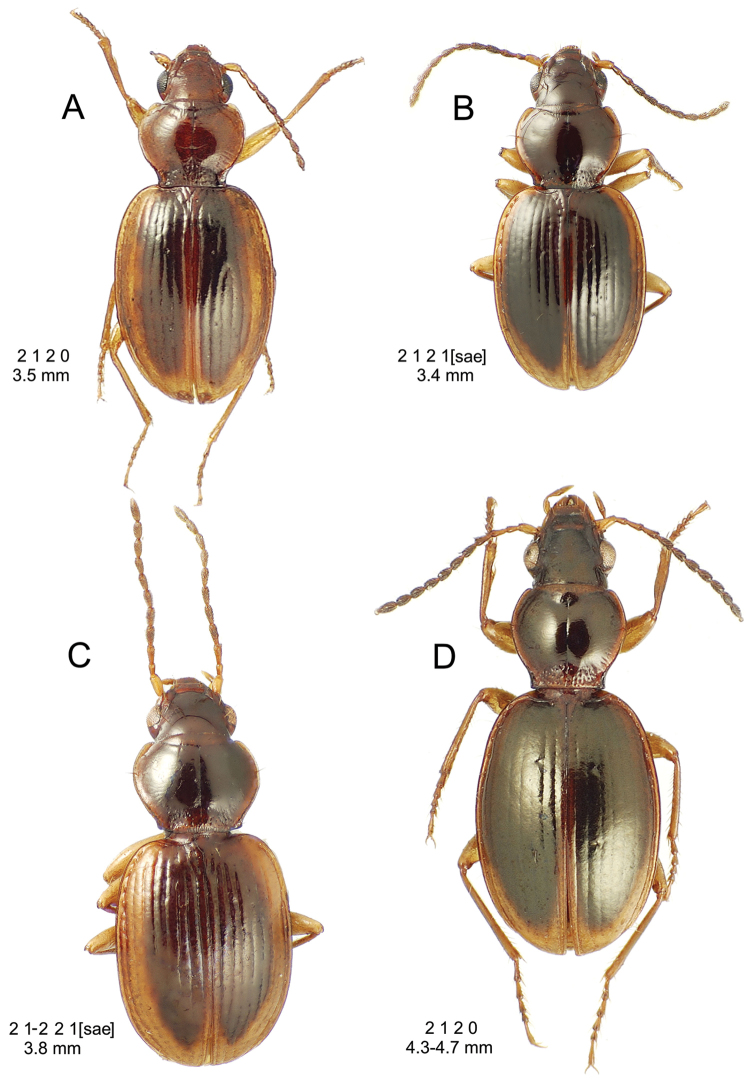
*Mecyclothorax ovipennis* group species, dorsal habitus view. **A** *Mecyclothorax subtilis* (nr. Ukulele Camp, 1525 m) **B** *Mecyclothorax patulus* (Waikamoi, 1305 m) **C** *Mecyclothorax patagiatus* (Kuhiwa E rim, 900 m) **D** *Mecyclothorax strigosus* (Kīpahulu W rim, 1850 m).

This is one of three species in the group that is characterized by bicolored elytra; the lateral elytral intervals flavous, contrasted to the piceous disc (Fig. 60A–C). In this species intervals 7–9 and the apex of the sutural stria are rufobrunneous to flavous, whereas the basal portions of intervals 2–5 are rufopiceous. The dorsal surface of the head and the pronotum are rufous with a flavous cast. The pronotum is moderately transverse, MPW/PL = 1.26, and basally constricted, MPW/BPW = 1.58. The dorsal surface of the head bears well-developed isodiametric sculpticells arranged in transverse rows. The setal formula is 2 1 2 0; the other two bicolored species—*Mecyclothorax patulus* and *Mecyclothorax patagiatus*—are characterized by presence of the subapical seta. Moreover, this species lacks the parascutellar seta, whereas it is present in the other two species. Standardized body length 3.45 mm.

##### Description

(n = 1). *Head capsule* with frontal grooves broad near clypeus, convexity present laterad groove, a narrow carina extended to supraorbital seta; dorsal surface of neck flat; eyes large, moderately convex, ocular ratio = 1.46, ocular lobe ratio = 0.84; labral anterior margin very shallowly emarginate medially; antennae filiform, antennomeres 2–3 glabrous except for 1 or 2 small setae on shafts; mentum tooth with sides acute, apex tightly rounded. *Pronotum* with glabrous hind angles, the base constricted, and lateral margins subparallel anterad the right, projected hind angles; median base moderately depressed, strigose due to long punctures and wrinkles; basal margin straight medially, margin expanded posterad laterobasal depressions; median longitudinal impression shallow, finely incised; anterior transverse impression shallow, broad, crossed by indistinct wrinkles; anterior callosity elevated, flat, crossed by indistinct longitudinal wrinkles; front angles projected, tightly rounded; pronotal apex broader than base, APW/BPW = 1.06; lateral marginal depression narrow, edge slightly upturned, broader at front angle, beaded anterad basal sinuation; laterobasal depression smooth, continuous with lateral depression; slight tubercle mesad lateral margin. *Proepisternum* with 5 minute punctures along hind marginal groove; prosternal process with narrow median impression, lateral margins broadly beaded between coxae. *Elytra* subellipsoid, disc flat, sides moderately sloped; basal groove slightly recurved to tightly rounded humeral angle; greatest width near midlength, MEW/HuW = 2.09; parascutellar striole discontinuous along length, with 3–4 punctures; sutural interval coplanar with lateral striae basally, upraised in apical half; sutural and 2^nd^ striae of subequal depth from base to apex; discal striae 1–5 broad but defined, lined with minute elongate punctulae in basal 1/3 of length, 6–7 discontinuous, represented by serial punctures, discal intervals moderately convex; 8^th^ interval slightly more convex than fused apical portion of fused striae 5 + 7; 2 dorsal elytral setae at 0.28× and 0.64× elytral length, setal impression small, spanning 1/3–1/2 of interval 3; apical and subapical setae absent; lateral elytral setae arranged in an anterior series of 6 setae and a posterior series of 5 setae; elytral marginal depression narrow, margin little upturned in basal half, narrowly beadlike near subapical sinuation; subapical sinuation very shallow, nearly obsolete. *Mesepisternum* with ~8 punctures in 1–2 rows; metepisternal width to length ratio = 0.71; metepisternum/metepimeron suture distinct. *Abdomen* with irregular lateral wrinkles on ventrites 1–5; suture between ventrites 2 and 3 complete; apical female ventrite with 4 equally spaced marginal setae plus median trapezoid of 4 subequal, short setae. *Legs*-metatarsomere 1/metatibial length ratio = 0.20; metatarsomere 4 length along outer lobe 1.33× medial tarsomere length, apical and subapical setae present; metatarsal dorsolateral sulci narrow, shallow, median surface broad. *Microsculpture* of pronotal disc transverse mesh, sculpticell breadth 2× length, median base with mixture of isodiametric and transverse sculpticells; elytral disc with transverse mesh, sculpticell breadth 2–4× length, apex with shallow transverse mesh of the same dimensions; metasternum with shallow transverse mesh; laterobasal abdominal ventrites with swirling isodiametric and transverse microsculpture. *Coloration* of antennomere 1 flavous, antennomeres 2–3 rufoflavous, 4–11 rufobrunneous; proepipleuron and proepisternum rufoflavous; elytral epipleuron rufoflavous, metepisternum rufobrunneous; abdomen with ventrites 1–3 medially pale rufobrunneous, lateral portions and ventrites 4–6 rufoflavous; femur rufoflavous; tibia rufoflavous with rufous cast.

**Female reproductive tract.** The lone female holotype was not dissected.

##### Holotype.

Female (BPBM) labeled: Haleakala / 5000 ft. / April / RCLP (obverse of mounting platen) / 369 (on reverse of platen) // Type (round red-margined label) // sp. n. near Mecyclothorax ovipennis // 1323 // HOLOTYPE / Mecyclothorax / subtilis / E.B. Britton / det. 1940 // HOLOTYPE / Mecyclothorax / subtilis Britton / & Liebherr / det. J.K. Liebherr 2015 (black-margined red label).

##### Etymology.

Britton’s choice of the Latin adjective subtilis to signify the minute, slender body of this beetle was extremely appropriate, and that choice is hereby validated.

##### Distribution and habitat.

Perkins’ lot 369 was collected on 10-iv-1894 at 5000 ft. (1524 m) elevation (Anonymous N D). The only relevant field note from that day (Perkins, 1894) states “Picked up a good number of Carabids, …” As Perkins made no mention of straying far from camp, Ukulele Camp (Site) is designated the type locality (Fig. 64).

#### (044) Mecyclothorax patulus sp. n.

Taxon classificationAnimaliaColeopteraCarabidae

http://zoobank.org/F43541E5-4856-4FF3-858D-73BE0BBBB639

Figs 60B , 64

##### Diagnosis.

This second of the three bicolored species (Fig. 60B) exhibits more narrowly flavous elytral margins—only intervals 8–9—versus piceous discal intervals 2–7. The sutural interval is rufous basally, flavous apically. The bisetose pronotum is more transverse than that of *Mecyclothorax subtilis*; MPW/PL = 1.28, and less constricted basally, MPW/BPW = 1.53. As in the next species below, *Mecyclothorax patagiatus*, the parascutellar and subapical elytral setae are present, but the elytral humeri are narrower in this species—MEW/HuW = 2.06 versus 2.0—and the elytra more ellipsoid. The setal impressions of the dorsal elytral setae are larger in beetles of this species, as they span interval 3. Setal formula 2 1 2 1[sae]. Standardized body length 3.4 mm.

##### Description

(n = 1). *Head capsule* with frontal grooves broad near clypeus, a lateral carina extended to mesad anterior supraorbital seta; dorsal surface of neck flat; eyes moderately convex, ocular ratio = 1.47, not extended onto posterior portion of ocular lobe, ocular lobe ratio = 0.75; labral anterior margin broadly emarginate to 1/6 of length; antennae filiform, sparse setae on apex of antennomere 1 and shafts of antennomeres 2–3; mentum tooth with sides acute, apex tightly rounded. *Pronotum* transverse, MPW/PL = 1.28, bisetose, glabrous hind angles obtuse, rounded behind, lateral margin subparallel for short distance anterad angle; base moderately broad, MPW/BPW = 1.53; median base nearly coplanar with disc, ~10 sparsely distributed, isolated punctures each side; basal margin convexly expanded between laterobasal depressions; median longitudinal impression shallow, finely incised, crossed by fine longitudinal wrinkles; anterior transverse impression deep, finely incised, minute irregularities in deepest part; anterior callosity convex, glossy surface with minute longitudinal wrinkles; front angles slightly projected, tightly rounded; pronotal apical width greater than basal width, APW/BPW = 1.10; lateral marginal depression narrow, edge upturned, slightly broader at front angle; laterobasal depression with slightly irregular surface, continuous with lateral depression. *Proepisternum* with 5 minute punctures along hind marginal groove; prosternal process with narrow median impression, lateral margins broadly beaded between coxae. *Elytra* broadly subellipsoid, disc flat, sides moderately sloped; basal groove evenly and distinctly recurved to tightly rounded humeral angle, MEW/HuW = 2.06; parascutellar seta present; parascutellar striole with 3–4 punctures, very shallow between punctures; sutural interval more convex than lateral intervals, sutural juncture upraised; sutural and 2^nd^ striae of subequal depth from base to disc, 2^nd^ stria slightly shallower at apex; discal striae 1–5 moderately broad, evident, stria 6 shallower and stria 7 shallower still, interrupted along length; striae 1–4 with minute elongate punctures, stria 5 with shallower punctures and stria 6 with only irregularities along length; intervals 2–5 moderately convex, lateral intervals less so; 8^th^ interval slightly more convex than fused apical portion of striae 5 + 7; 2 dorsal elytral setae at 0.28–0.32× and 0.62× elytral length, setal impressions evident, spanning interval 3; apical elytral seta absent, subapical elytral seta present; lateral elytral setae arranged as anterior series of 6 setae and posterior series of 5 setae; elytral marginal depression narrow, edge little upturned in basal half, margin beadlike near subapical sinuation; subapical sinuation very shallow, nearly obsolete. *Mesepisternum* with ~6 punctures in 1–2 rows; metepisternal width to length ratio = 0.80; metepisternum/metepimeron suture distinct. *Abdomen* with irregular lateral wrinkles on ventrites 1–5, lateral depressions on ventrites 3–6; suture between ventrites 2 and 3 complete; apical female abdominal ventrite with 4 equally spaced setae plus trapezoid of 4 subequal, short setae. *Legs*-metatarsomere 1/metatibial length ratio = 0.20; metatarsomere 4 length along outer lobe 1.3× medial tarsomere length, apical and subapical setae present; metatarsal dorsolateral sulci narrow, shallow, median surface broad. *Microsculpture* of vertex shallow isodiametric sculpticells in rows; pronotal disc with shallow transverse mesh, sculpticell breadth 3× length, to transverse lines not connected into mesh; pronotal median base glossy, obsolete transverse mesh between punctures; elytral disc and apex with shallow transverse mesh, sculpticell breadth 2–4× length; metasternum with transverse mesh; laterobasal abdominal ventrites with swirling isodiametric and transverse microsculpture. *Coloration* of vertex rufobrunneous; antennomere 1 flavous, antennomeres 2–3 rufoflavous, 4–11 rufobrunneous; pronotal disc rufobrunneous with piceous cast, lateral margins, apex, and base rufoflavous; proepipleuron flavous, proepisternum rufobrunneous with piceous cast; elytral epipleuron dorsally flavous, ventrally rufoflavous, metepisternum rufobrunneous with piceous cast; abdomen with ventrites 1–2 medially, and 3–5 mediobasally rufopiceous, ventrites 3–6 apical and marginally rufoflavous; metafemur flavous; metatibia flavous with brunneous cast.

**Female reproductive tract.** The lone female holotype was not dissected.

##### Holotype.

Female (NMNH) labeled: HI:Maui Koolau / F.R. Kula Pipeline Rd. / N20°48.58', W156°14.30', / 18-V-2003 lot09 el. 1305m / pyr. fog log D.A. Polhemus // HOLOTYPE / Mecyclothorax / patulus / Liebherr / det. J.K. Liebherr 2015 (black-margined red label).

##### Etymology.

The Latin adjective patulus means open, spread out, or broad (Brown 1956), signifying the short, broad body of these beetles.

##### Distribution and habitat.

*Mecyclothorax patulus* is known only from an ‘Ōhi‘a Wet Forest site uphill from the junction of the Kula Pipeline and Waikamoi Flume Roads (Fig. 64). The type specimen was found in a pyrethrin fog sample of a downed ‘ōhi‘a log covered with moss.

#### (045) Mecyclothorax patagiatus sp. n.

Taxon classificationAnimaliaColeopteraCarabidae

http://zoobank.org/3B4C8BE9-2324-4C8A-88C1-A5BE2729CBED

**Figure 61.**
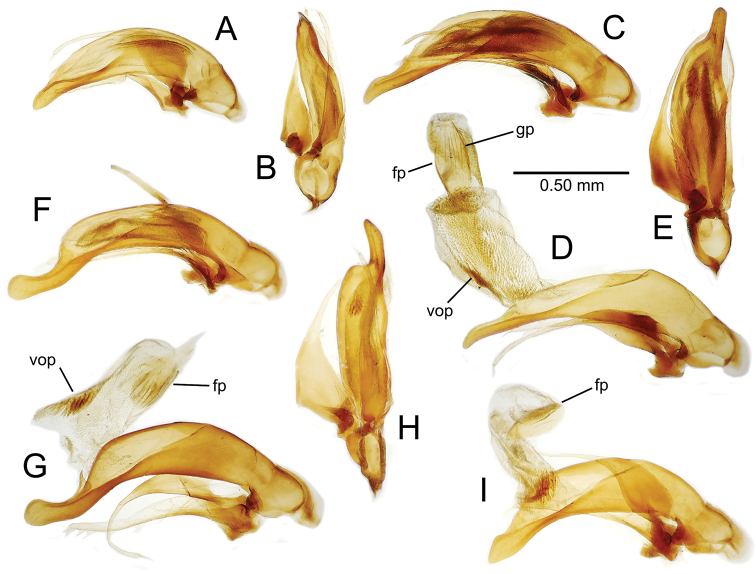
Male aedeagus, *Mecyclothorax ovipennis* group species (for abbreviations see Table 2, p. 23). **A–B** *Mecyclothorax patagiatus* right and ventral views (Kuhiwa E rim, 900 m) **C–E** *Mecyclothorax ovipennis* **C** Right view (Kopili‘ula, 1170 m) **D** Ventral view (Helele‘ike‘oha, 1615 m). **E** Ventral view (Kopili‘ula, 1170 m) **F–H** *Mecyclothorax apicalis*. **F** Right view (NW upper slope, 2745 m). **G** Right view, sac everted (summit, 2895–3050 m) **H** Ventral view (NW upper slope, 2745 m) **I** *Mecyclothorax parapicalis*, right view, sac everted (Holua Cabin, 2134 m).

Figs 60C , 61A–B , 64

##### Diagnosis.

This, the third Haleakalā *Mecyclothorax ovipennis* group species to display bicolored elytra (Fig. 60C), can be diagnosed by the following combination: 1, discal elytral striae 1–6 rufobrunneous with piceous cast, intervals 7–9 contrastedly rufoflavous; 2, pronotal disc also rufobrunneous with piceous cast, pronotal margins rufoflavous; 3, parascutellar seta present; 4, subapical elytral seta present, apical seta absent; 5, pronotal lateral margins slightly divergent from hind obtuse hind angles; 6, elytral humeri broadly rounded, the elytra broadly subquadrate, MEW/HuW = 2.0. Criteria 1, 5, and 6 diagnose this species from both *Mecyclothorax subtilis* and *Mecyclothorax patulus*, whereas criteria 2, 3, and 4 diagnose this species from *Mecyclothorax subtilis*. The pronotum of the unique holotype has the basal pronotal seta present on the right side, with the left hind angle glabrous (specimen examined at 125×), obviating use of this character in the diagnosis. Setal formula 2 1-2 2 1[sae]. Standardized body length 3.8 mm (slightly larger than both preceding species).

##### Description

(n = 1). *Head capsule* with frontal grooves broad near clypeus, lateral carina to anterior supraorbital seta; dorsal surface of neck flat; eyes moderately convex, ocular ratio = 1.43, ocular lobe ratio 0.78; labral anterior margin broadly emarginate to 1/6 of length; antennae filiform, antennomeres 2–3 with sparse pelage of short setae; mentum tooth with sides acute, apex tightly rounded. *Pronotum* transverse, MPW/PL = 1.30, base moderately constricted, MPW/BPW = 1.50; hind angle obtuse, margin behind rounded; median base moderately depressed, ~20 punctures or strigose wrinkles each side; basal margin convexly expanded between laterobasal depressions; median longitudinal impression evident, finely incised, joined by fine transverse wrinkles; anterior transverse impression deep, finely incised, minute irregularities in deepest part; anterior callosity elevated, flat, crossed by indistinct longitudinal wrinkles; front angles projected, broadly rounded; apical and basal pronotal widths subequal, APW/BPW = 1.03; lateral marginal depression moderate, edge upturned, broader at front angle; laterobasal depression narrowly concave, continuous with lateral depression. *Proepisternum* with 5 minute punctures along hind marginal groove; prosternal process narrowly impressed medially, lateral margins broadly beaded between coxae. *Elytra* with disc flat, sides moderately sloped; basal groove slightly recurved to broadly rounded humerus; parascutellar striole with 3–4 punctures, shallow between punctures; sutural interval coplanar with lateral intervals basally, upraised in apical half; sutural and 2^nd^ striae of subequal depth and breadth from base to apex; discal striae 1–5 broad, deep, stria 6 shallower and more irregular, stria 7 shallower still; sutural stria deep, finely punctate basally, deep, narrow, and smooth apically; striae 2–4 with minute punctures on disc, punctures shallower in stria 5, linear irregularities in stria 6; discal intervals 2–4 convex, lateral intervals less so; 8^th^ interval slightly more convex than fused apical portion of striae 5 + 7; 2 dorsal elytral setae at 0.27× and 0.54× elytral length, setal impressions small, spanning ½ width of interval 3; lateral elytral setae arranged in anterior series of 6 setae, posterior series of 5 setae; elytral marginal depression narrow, margin little upturned in basal half, beadlike near subapical sinuation; subapical sinuation very shallow, nearly obsolete. *Mesepisternum* with ~6 punctures in 1–2 rows; metepisternal width to length ratio = 0.80; metepisternum/metepimeron suture distinct. *Abdomen* with irregular lateral wrinkles in ventrites 1–5, lateral depressions in ventrites 3–6; suture between ventrites 2 and 3 complete; apical male ventrite with 2 marginal setae. *Legs*-metatarsomere 1/metatibial length ratio = 0.19; metatarsomere 4 length along outer lobe 1.3× medial tarsomere length, apical and subapical setae present; metatarsal dorsolateral sulci narrow, shallow, median surface broad. *Microsculpture* of vertex with evident isodiametric sculpticells in transverse rows; pronotal disc with shallow transverse mesh, sculpticell breadth 3× length, to transverse lines; pronotal median base with shallow transverse mesh laterally, median area glossy; elytral disc with evident transverse mesh, sculpticell breadth 2–4× length, apex with more distinct transverse mesh of same dimensions; metasternum with transverse mesh; laterobasal abdominal ventrites with swirling isodiametric and transverse microsculpture. *Coloration* of vertex rufobrunneous; antennomere 1 flavous, antennomeres 2–3 rufoflavous; 4–11 rufobrunneous; pronotal disc rufobrunneous with piceous cast, margins rufoflavous; proepipleuron rufoflavous, proepisternum rufobrunneous with piceous cast; elytral sutural interval concolorous with disc basally, rufoflavous apically; elytral epipleuron dorsally flavous, ventrally rufoflavous; metepisternum rufobrunneous; abdomen with ventrites 1–2 medially, 3–5 mediobasally rufopiceous, 3–6 apically and marginally rufoflavous, apical ventrite 6 with apical ¾ paler, flavous; metafemur flavous; metatibia flavous with brunneous cast.

**Male genitalia** (n = 1). Aedeagal median lobe robust, distance between parameral articulation and tip 3.2× depth at midlength (Fig. 61A); extension of apex beyond ostial opening parallel sided at base and evenly narrowed dorsoventrally to tightly rounded tip; median lobe curved sinuously leftward toward apex in ventral view, the right margin distinctly concave, left margin convex (Fig. 61B); internal sac without ornamentation, flagellar plate elongate, length of sclerotized plate 0.54× parameral articulation-tip distance.

##### Holotype.

Male (CUIC) dissected and labeled: HI: E.Maui, Kuhiwa / 2950 ft.,10June1999 / *Cibotium chamissois* / Dead fronds // C. Ewing Coll. / 20°46'25˝N / 156°06'04˝W // HOLOTYPE / Mecyclothorax / patagiatus / Liebherr / det. J.K. Liebherr 2015 (black-margined red label).

##### Etymology.

The Latin stem for this species epithet is patagium; i.e. a gold edging or border (Brown 1956). The adjectival form patagiatus signifies the pale elytral border of beetles of this species.

##### Distribution and habitat.

*Mecyclothorax patagiatus* is known from 900 m elevation in the Kuhiwa Valley of the Hanawī windward face of Haleakalā (Fig. 64). The type specimen was collected from dead *Cibotium* (hāpu‘u) tree fern fronds along with one specimen each of *Bembidion haleakalae* Liebherr, *Mecyclothorax mauiae* (Fig. 71), and *Mecyclothorax bacrionis* (Fig. 112).

#### (046) Mecyclothorax strigosus sp. n.

Taxon classificationAnimaliaColeopteraCarabidae

http://zoobank.org/8318299A-5224-4736-84FD-B2E2AB8B09F1

**Figure 62.**
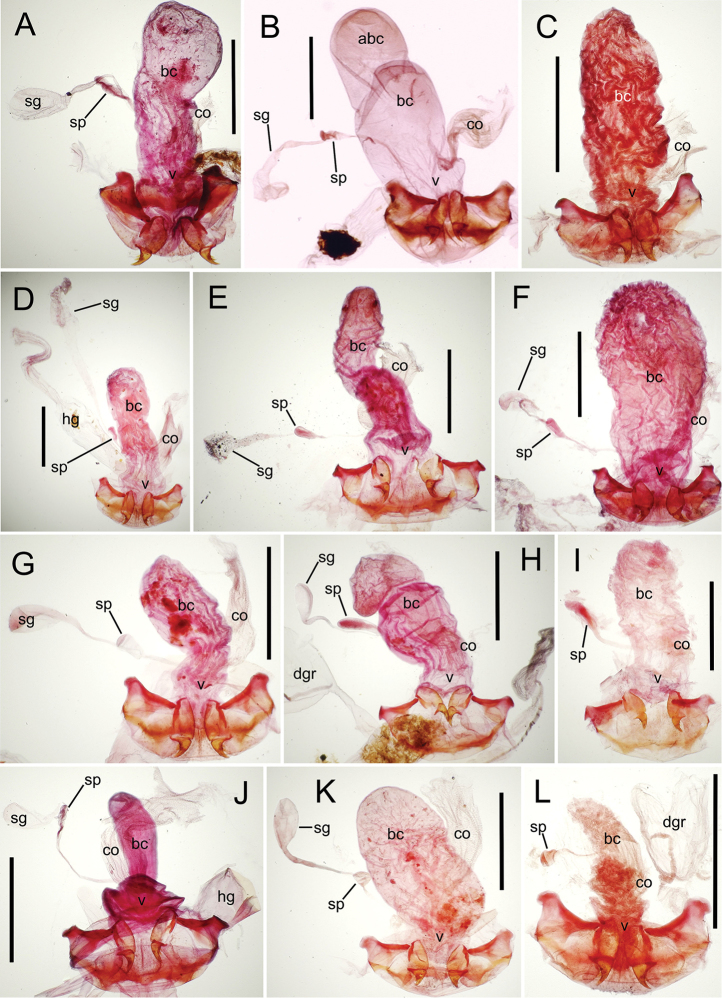
Female bursa copulatrix and associated reproductive structures, *Mecyclothorax ovipennis* group species, ventral view (for abbreviations see Table 2, p. 23). **A** *Mecyclothorax strigosus* (Honomanu, 1860 m) **B** *Mecyclothorax ovipennis* (Kopili‘ula, 1170 m) **C** *Mecyclothorax apicalis* (summit, 2895–3050 m) **D** *Mecyclothorax mauiae* (Waikamoi, 1305 m) **E** *Mecyclothorax mauiae* (Waikamoi, 1310 m). **F** *Mecyclothorax subternus* (Kuhiwa, 1030 m) **G** *Mecyclothorax flaviventris* (Kuhiwa, 2070–2100 m) **H** *Mecyclothorax laetus* (Ukulele Camp Pipeline, 1495-1525 m) **I** *Mecyclothorax cordaticollis* (nr. Ukulele Camp, 1525 m) **J** *Mecyclothorax cordaticollaris* (Kaupō Gap, 1495 m) **K** *Mecyclothorax subconstrictus* (summit, 2895–3050 m) **L** *Mecyclothorax pusillus* (summit, 2895–3050 m). Scale bar = 0.50 mm.

**Figure 63.**
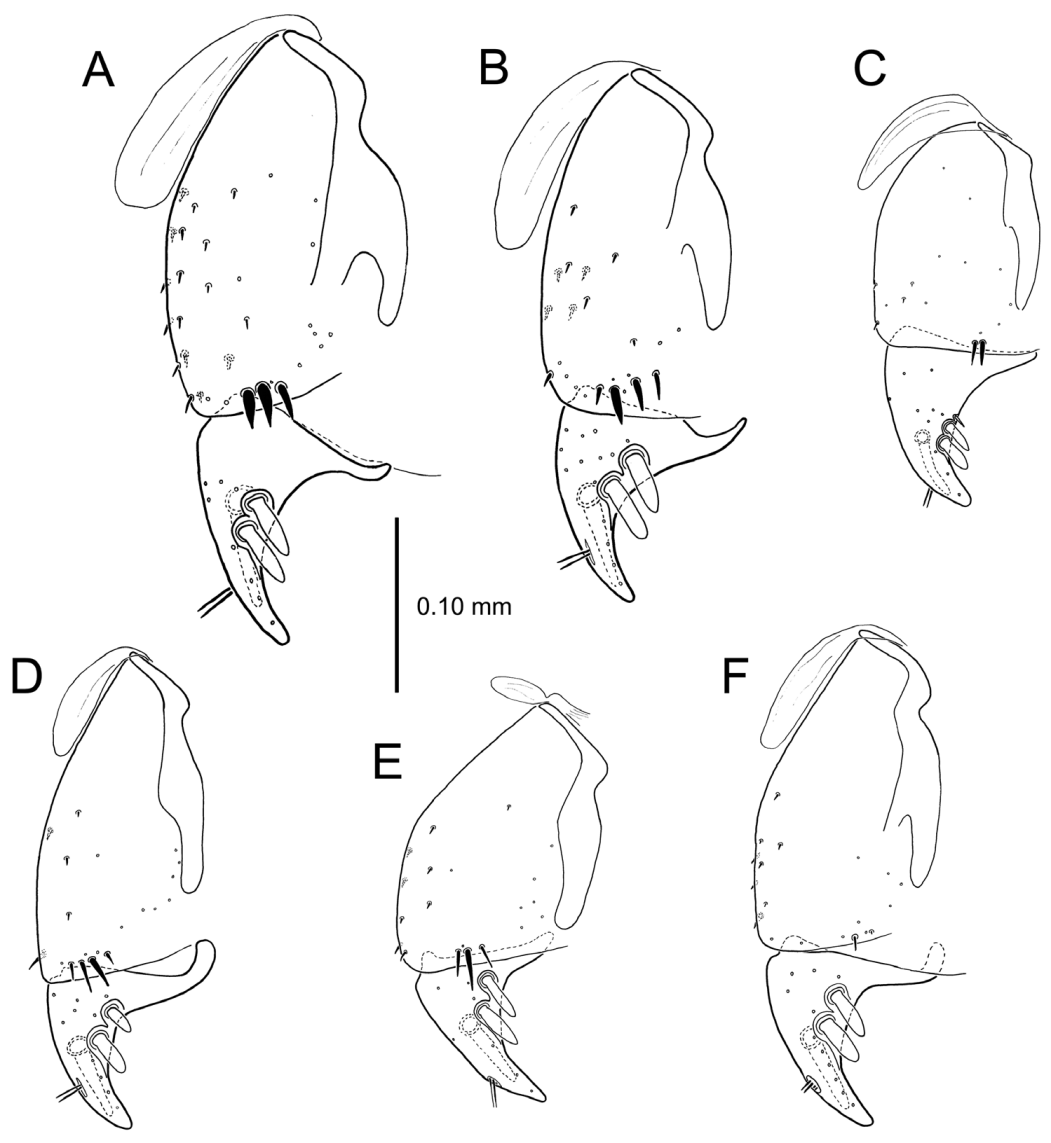
Left female gonocoxa, *Mecyclothorax ovipennis* group species, ventral view. **A** *Mecyclothorax strigosus* (Honomanu, 1860 m) **B** *Mecyclothorax ovipennis* (Kopili‘ula, 1170 m) **C** *Mecyclothorax apicalis* (summit, 2895–3050 m) **D** *Mecyclothorax mauiae* (Waikamoi, 1305 m) **E** *Mecyclothorax subternus* (Kuhiwa, 1030 m). **F** *Mecyclothorax flaviventris* (Kuhiwa, 2070–2100 m).

**Figure 64.**
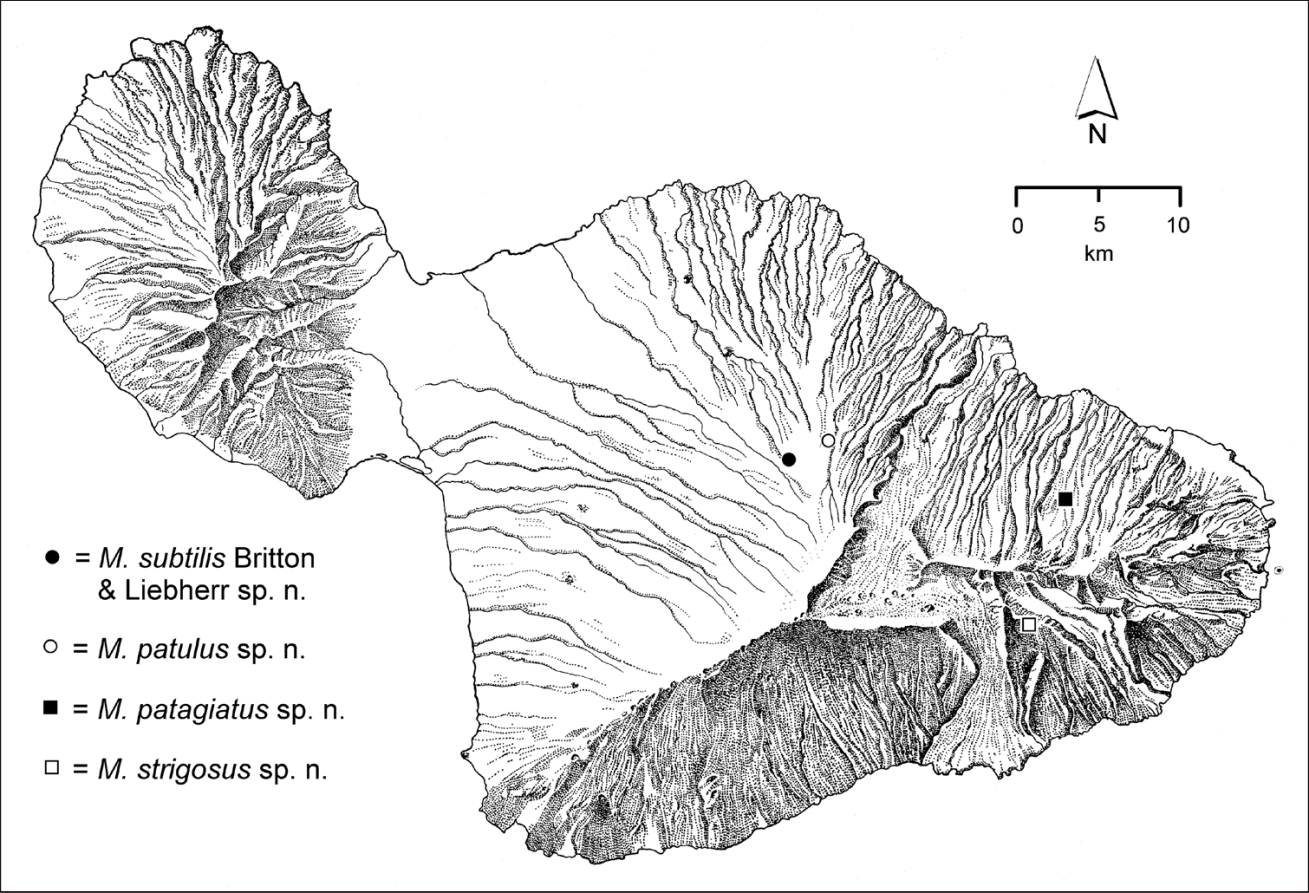
Recorded geographic distributions of *Mecyclothorax ovipennis* group species.

Figs 60D , 62A , 63A , 64

##### Diagnosis.

**Figure 65.**
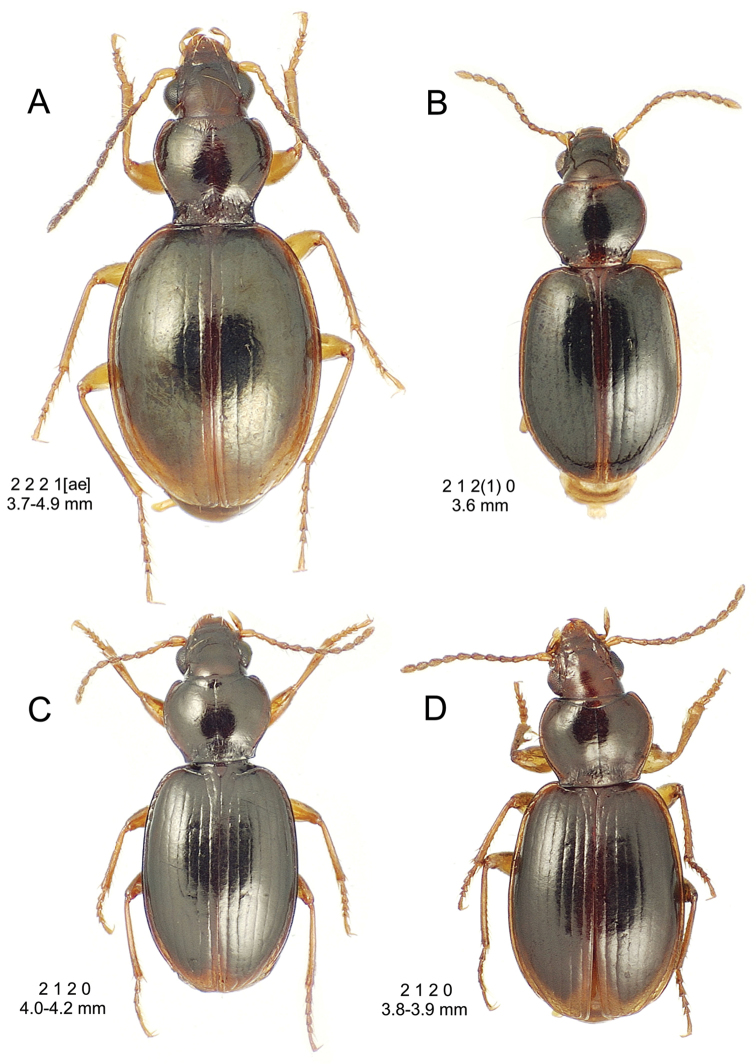
*Mecyclothorax ovipennis* group species, dorsal habitus view. **A** *Mecyclothorax ovipennis* (Kīpahulu, 1800 m) **B** *Mecyclothorax takumiae* (Paliku, 1950 m) **C** *Mecyclothorax apicalis* (summit, 2895–3050 m) **D** *Mecyclothorax parapicalis* (Holua, 2135 m).

This species (Fig. 60D) plus *Mecyclothorax ovipennis* (Fig. 65A) and *Mecyclothorax flaviventris* (Fig. 68C) comprise the three species in this group with the largest, most convex eyes; ocular ratio = 1.55–1.57 in this species. Of these, both *Mecyclothorax ovipennis* and this species are characterized by impunctate discal striae 1–4. This species (Fig. 60D) can be told from *Mecyclothorax ovipennis* (Fig. 65A) by the narrower body with more basally constricted pronotum, MPW/BPW = 1.56–1.67, and more narrowly ellipsoid elytra. Moreover, this is the only one of the three with glabrous hind pronotal angles, and without any apical elytral setae; setal formula 2 1 2 0. Standardized body length 4.3–4.7 mm.

##### Description

(n = 2). *Head capsule* with frontal grooves broad near clypeus, lateral carina to anterior supraorbital seta; dorsal surface of neck flat; ocular lobe distinctly protruded from gena, eyes large, ocular lobe ratio = 0.79–0.80; labral anterior margin very shallowly emarginate medially; antennae filiform, antennomeres 2–3 with sparse pelage of short setae; mentum tooth with sides acute, apex broadly rounded. *Pronotum* appearing elongate, MPW/PL = 1.11–1.18; hind angle right, lateral margin straight, subparallel to slightly convergent anterad hind angle; median base only slightly depressed, ~15 densely distributed punctures each side extended to laterobasal depression; basal margin nearly straight across base; median longitudinal impression very shallow, finely incised; anterior transverse impression very shallow, broad, crossed by longitudinal wrinkles; anterior callosity nearly flat, crossed by indistinct wrinkles; front angles not projected, tightly rounded; pronotal apical width greater than basal width, APW/BPW = 1.09–1.16; lateral marginal depression very narrow throughout length, edge tightly upturned; laterobasal depression depressed, punctate surface continuous with median base. *Proepisternum* with 5 minute punctures along hind marginal groove; prosternal process with narrow median impression, lateral margins broadly beaded between coxae. *Elytra* with disc flat, sides steeply sloped to depressed lateral margins and apex; basal groove briefly, distinctly recurved to angulate humerus; parascutellar seta present; parascutellar striole with 4–5 punctures, shallow, continuous between punctures; sutural interval more convex than intervals 2–4, but sutural juncture still depressed; sutural stria shallow, with minute punctulae basally, slightly deeper and more well defined than 2^nd^ stria on disc, the two of subequal depth apically; discal striae 2–4 shallow, smooth, striae 5–6 obsolete but traceable, stria 7 absent; discal intervals 2–4 only slightly convex to nearly flat, lateral intervals flat; 8^th^ interval of similar convexity to fused apical portion of striae 5 + 7; 2 dorsal elytral setae at 0.30–0.31× and 0.61–0.66× elytral length, setal impressions very small, spanning 1/3 of interval 3; lateral elytral setae arranged in anterior series of 7 setae, and posterior series of 6 setae; elytral marginal depression narrow, margin upturned, beaded only at subapical sinuation; subapical sinuation shallow, more abruptly incurved anteriorly. *Mesepisternum* with ~7 punctures in 2 rows; metepisternal width to length ratio = 0.72; metepisternum/metepimeron suture distinct. *Abdomen* with irregular lateral wrinkles on ventrites 1–3, lateral depressions on ventrites 3–6; suture between ventrites 2 and 3 reduced laterally, effaced; apical female ventrite with 4 equally spaced setae plus median trapezoid of 4 setae, the basal pair longer. *Legs*-metatarsomere 1/metatibial length ratio = 0.18; metatarsomere 4 length along outer lobe 1.25× medial tarsomere length, apical and subapical setae present; metatarsal dorsolateral sulci narrow, shallow, median area broad. *Microsculpture* of vertex and pronotal disc a transverse mesh, sculpticell breadth 2–3× length; pronotal median base with transverse mesh, sculpticell breadth 2× length, between punctures; elytral disc and apex with isodiametric to transverse sculpticells, sculpticell breadth 2× length, in transverse rows; metasternum with distinct transverse mesh; laterobasal abdominal ventrites with swirling isodiametric and transverse microsculpture. *Coloration* of vertex rufobrunneous; antennomere 1–3 flavous, 4–11 darker, more brunneous; pronotal disc rufobrunneous with piceous cast, the lateral margins moderately, and base and apex broadly, rufoflavous; proepipleuron rufoflavous, proepisternum rufobrunneous with piceous cast; elytral disc dark, rufobrunneous to rufopiceous; sutural interval rufobrunneous basally, flavous in apical 1/3; elytral intervals 7–9 slightly paler than disc, rufoflavous basally, flavous apically; elytral epipleuron flavous, metepisternum rufobrunneous with piceous cast; abdomen with ventrite 1 (plus metepimeron) rufobrunneous, ventrites 2–3 and middle of ventrites 4–5 rufopiceous; abdominal apical ventrite with apical 2/3 flavous; metafemur flavous; metatibia flavous with brunneous cast.

**Female reproductive tract** (n = 1). Bursa copulatrix columnar with apical expansion, length 1.0 mm, apical expansion breadth 0.46 mm, basal breadth 0.34 mm (Fig. 62A); bursal shaft translucent, thinly wrinkled, apex more transparent, less wrinkled; gonocoxite 1 with 3 apical fringe setae, the middle seta more robust, a larger seta at apicomedial angle and 12–13 smaller setae basally on medial surface (Fig. 63A); gonocoxite 2 falcate with subacuminate apex, base extended laterally as sinuous panhandle, 2 lateral ensiform setae, apical nematiform setae on medial surface at 0.75× gonocoxite length.

##### Holotype.

Female (CUIC) labeled: HI: Maui Haleakala N.P. / Kipahulu west rim ESE / Kuiki, sift humus ex ohia / 15-V-1993 lot 02 / el. 1850 m / J.K. Liebherr & / A.C. Medeiros / Collectors // 2 // HOLOTYPE / Mecyclothorax / strigosus / Liebherr / det. J.K. Liebherr 2015 (black-margined red label).

##### Paratype.

Female (CUIC) dissected, with same label as holotype except “1” instead of “2.”

##### Etymology.

The Latin adjectival strigosus, meaning thin, signifies the narrow body shape characteristic of this species.

##### Distribution and habitat.

*Mecyclothorax strigosus* has only been encountered in ‘Ōhi‘a Montane Wet Forest ESE of Kuiki at 1850 m elevation (Fig. 64). The two specimens were found in a litter sample sifted from humus surrounding the bases of large ‘ōhi‘a trees. The forest also included *Cheirodendron* (‘ōlapa) and *Leptecophylla tameiameiae* (pūkiawe). The sample containing specimens of *Mecyclothorax strigosus* also included specimens of *Mecyclothorax antaeus*, *Mecyclothorax consanguineus*, *Mecyclothorax mauiae*, *Mecyclothorax ovipennis*, and *Mecyclothorax pau*.

#### (047) Mecyclothorax ovipennis

Taxon classificationAnimaliaColeopteraCarabidae

Sharp

**Figure 66.**
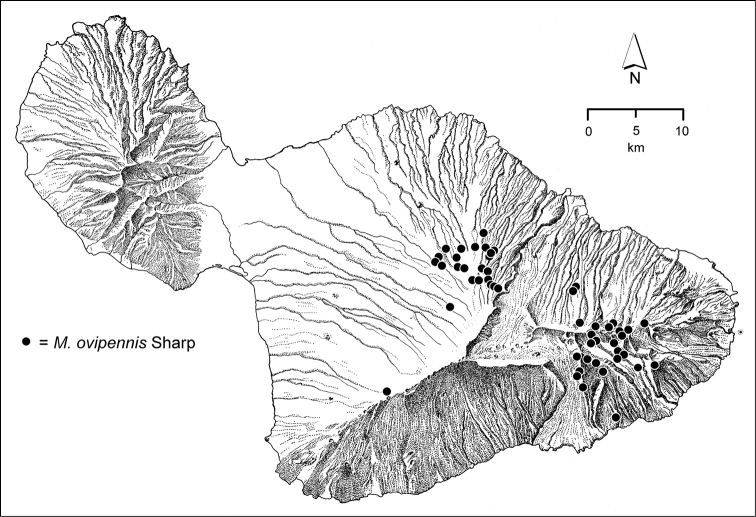
Recorded geographic distribution of *Mecyclothorax ovipennis*.

Figs 61C–E , 62B , 63B , 65A , 66

Mecyclothorax ovipennis Sharp 1903: 250; Britton 1948b: 145; Swezey 1954: 27, 53 (biology); Liebherr 2005b: 109.

##### Diagnosis.

Among Haleakalā *Mecyclothorax* (Fig. 65A), this is most similar to the preceding, *Mecyclothorax strigosus* (Fig. 60D) based on the well-developed eyes, ocular ratio = 1.55–1.61 and ocular lobe ratio = 0.84–0.89, plus basally constricted pronotum and ellipsoid elytra. They can be separated by the setal conformation, with this species characterized by a quadrisetose pronotum, and presence of the apical elytral seta; setal formula 2 2 2 1[ae]. This species is characterized by the same setal formula as *Mecyclothorax flaviventris*, but individuals of that species exhibit punctate discal striae (Fig. 68C). Standardized body length 3.7–4.9 mm.

##### Identification

(n = 5). The pronotum is very cordate in this species, MPW/BPW = 1.46–1.59, with the lateral margin subparallel to convergent for 0.2× the pronotal length anterad the projected, right to acute hind angles. The pronotum appears elongate, but is actually slightly transverse; MPW/PL = 1.08–1.21. The surface of the pronotal disc, and anterior transverse impression and associated callosity are irregularly wrinkled. Of the elytral striae, only sutural stria 1 is moderately deep basally, with elongate punctures that expand the stria basally, the stria smooth and deep apically. Striae 2–4 are shallower on the disc, striae 5–7 progressively shallower, and striae 6–7 discontinuous. At the elytral apex, stria 2 is of subequal depth to the sutural stria, fused striae 3 + 4 and 7 are present, and the apices of striae 5 and 6 are shallow but traceable. The vertex bears isodiametric and transverse sculpticells in transverse rows, the transverse sculpticells 2–3× broad as long. The pronotal and elytral discs are covered with transverse-mesh microsculpture, sculpticell breadth 1.5–3× length; the pronotal base has a shallow transverse mesh between glossy portions of the cuticle.

**Male genitalia** (n = 8). Aedeagal median lobe gracile, distance between parameral articulation and tip 4× depth at midlength (Fig. 61C); apex well extended beyond apex of ostial opening, dorsal surface of projection broadly convex, then flattened dorsad tightly rounded tip, ventral margin slightly concave due to downward curvature of tip (Figs 61C–D); median lobe not curved in ventral view, though left margin distinctly incurved to apical extension, and right margin concave before apex (Fig. 61E); internal sac broader near flagellar plate, covered with well-developed pelage of microspicules, the spicules densest on ventral surface forming a poorly developed ventral ostial microtrichial patch (Fig. 61D); flagellar plate moderately elongate, length 0.42× parameral articulation-tip distance.

**Female reproductive tract** (n = 1). Bursa copulatrix columnar, subdivided into broader basal portion and slightly narrower apical lobe, overall length 0.91 mm, breadth 0.31 mm (Fig. 62B); bursal walls diaphanous, very thin and with indistinct wrinkles; gonocoxite 1 with 4–5 apical fringe setae, a curved seta just basad apicomedial angle, and 8–10 smaller setae on medial margin, setae subequally divided between ventral and dorsal surfaces (Fig. 63B); gonocoxite 2 falcate with tightly rounded apex, base extended laterally into sinuous panhandle, 2 subequal lateral ensiform setae, apical nematiform setae on medial surface at 0.68× gonocoxite length.

##### Lectotype.

Female (BMNH) designated by Liebherr (2005b: 110). Type locality Haleakala, 4500–6000 ft., III-1894 (R.C.L.P. lot 383; Anonymous N D).

##### Distribution and habitat.

*Mecyclothorax ovipennis* is among the most broadly distributed *Mecyclothorax* species on Haleakalā (Fig. 66). It requires, at the minimum, mesic forest conditions, being isolated at Polipoli Springs on the Kula face. It occurs along the eastern margin of Haleakalā Crater where mesic forest occurs. It is also at home in wetter forest situations throughout Waikamoi, Hanawī, Hāna Bogs, Kīpahulu Valley, and the Manawainui Planeze. It has been collected in habitats ranging 880–2134 m elevation. It has been found in association with a great diversity of plant species, including ferns (*Asplenium*, *Cibotium*, *Dicranopteris*, and *Sadleria*), herbaceous secondarily woody shrubs (*Coprosma*, *Cyanea*, *Myrsine*, *Rubus*, and *Vaccinium*) and emergent trees (koa and ‘ōhi‘a). It is also commonly encountered in sifted litter.

#### (048) Mecyclothorax takumiae sp. n.

Taxon classificationAnimaliaColeopteraCarabidae

http://zoobank.org/A9866865-1B53-4DC7-AD1C-AD08858BD0D8

Figs 65B , 67

##### Diagnosis.

Within the *Mecyclothorax ovipennis* group, this species (Fig. 65B) forms part of a triplet of species also including *Mecyclothorax apicalis* (Fig. 65C) and *Mecyclothorax parapicalis* (Fig. 65D) that are collectively diagnosed by glabrous, obtuse pronotal hind angles, shallow impunctate discal elytral striae, and concolorous elytral disc and margins. Of these, *Mecyclothorax takumiae* is characterized by the smallest body size, standardized body length 3.6 mm, and least developed microsculpture. The pronotal disc and median base are glossy with obsolete transverse-mesh microsculpture and parallel lines over portions of the cuticle, whereas the elytral disc has a shallow mesh of isodiametric and transverse sculpticells, breadth 2–3× length. Both *Mecyclothorax apicalis* and *Mecyclothorax parapicalis* have well-developed sculpticells covering those areas. Setal formula 2 1 2(1) 0; the right elytron has both dorsal elytral setae, whereas the left has only a seta at the anterior position of the right elytron. As the bisetose condition is shared with *Mecyclothorax apicalis* and *Mecyclothorax parapicalis*, the unisetose elytron is considered a variant.

##### Description

(n = 1). *Head capsule* with frontal grooves broad near clypeus, lateral carina to anterior supraorbital seta; dorsal surface of neck flat; eyes moderately developed, ocular ratio = 1.45, ocular lobe ratio = 0.80; labral anterior margin medially emarginate, medially excavated 1/6 of length; antennae filiform, antennomeres 2–3 with only 1 or 2 short setae on shafts; mentum tooth with sides acute, apex rounded. *Pronotum* slightly transverse, MPW/PL = 1.21, moderately constricted basally, MPW/BPW = 1.53; hind angle obtuse, margin behind rounded; median base markedly depressed versus disc, ~9 punctures each side, surface glossy between; basal margin convexly expanded between laterobasal depressions; median longitudinal impression shallow, finely incised, crossed by fine transverse wrinkles; anterior transverse impression narrow, shallowly incised laterally, obsolete medially; anterior callosity slightly convex, smooth, glossy; front angles not to slightly projected, tightly rounded; pronotal apical and basal width equal, APW/BPW = 1.0; lateral marginal depression narrow through apical 1/3 of length, edge upturned, widened in basal 1/3; laterobasal depression narrow, surface irregular, continuous with lateral depression. *Proepisternum* with 5 minute punctures along hind margin; prosternal process with narrow median impression, lateral margins broadly beaded between coxae. *Elytra* subquadrate, disc flat, sides distinctly sloped; basal groove evenly recurved to subangulate (left) to tightly rounded (right) humerus, MEW/HuW = 2.04; parascutellar seta present; parascutellar striole with 4 punctures, shallow between punctures; sutural interval slightly more convex than lateral intervals, upraised at sutural juncture; sutural stria shallow, continuous basally, punctate on disc, smooth and deep apically; striae 2–6 progressively shallower, smooth, stria 7 obsolete; stria 1 subequal to slightly deeper than stria 2 at elytral apex; elytral interval 2 slightly convex, intervals 3–7 progressively flatter; 8^th^ interval subcarinate laterad fused apical portion of striae 5 + 7; 2 dorsal elytral setae at 0.31× and 0.63× elytral length (right elytron), setal impressions moderate, spanning 2/3 of interval 3; apical and subapical setae absent; lateral elytral setae arranged in anterior series of 6 setae and posterior series of 6 setae; elytral marginal depression slightly broader at humerus, narrowed laterally, beadlike at subapical sinuation; subapical sinuation shallow, more abruptly incurved anteriorly. *Mesepisternum* with ~8 punctures in 1–2 rows; metepisternal width to length ratio = 0.65; metepisternum/metepimeron suture distinct. *Abdomen* with irregular lateral wrinkles on ventrites 1–6; suture between ventrites 2 and 3 complete; apical female ventrite with 4 equally spaced setae plus a median trapezoid of 4 subequal, short setae. *Legs*-metatarsomere 1/metatibial length ratio = 0.18; metatarsomere 4 length along outer lobe 1.33× medial tarsomere length, apical and subapical setae present; metatarsal dorsolateral sulci narrow, shallow, median area broad. *Microsculpture* of vertex a shallow transverse mesh, sculpticell breadth 2–3× length; elytral apex with transverse mesh, sculpticell breadth 2–3× length; metasternum with transverse mesh; laterobasal abdominal ventrites with swirling isodiametric and transverse microsculpture. *Coloration* of vertex rufobrunneous with piceous cast; antennomeres 1–3 flavous, 4–11 darker, more brunneous; pronotal disc rufopiceous, lateral margins concolorous, base and apex paler, rufoflavous; proepipleuron rufoflavous, proepisternum rufobrunneous with piceous cast; elytral disc rufopiceous, sutural interval rufous in basal half, flavous in apical half; 9^th^ elytral interval and lateral marginal depression rufous, apex of intervals 8 and 9 flavous; elytral epipleuron dorsally flavous, ventrally rufoflavous, metepisternum rufopiceous; abdomen with ventrites 1–5 and the base of 6 rufopiceous, apical 1/3 of apical ventrite 6 flavous; metafemur flavous; metatibia flavous with brunneous cast.

**Female reproductive tract.** The lone female holotype was not dissected.

##### Holotype.

Female (BPBM) labeled: HAWAII: Maui I. / Haleakala Crater / Paliku, 1950 m / 1 JUL 1998 // R. Takumi, coll. / HALE-RM / ex. pitfall trap // HOLOTYPE / Mecyclothorax / takumiae / Liebherr / det. J.K. Liebherr 2015 (black-margined red label).

##### Etymology.

Raina Takumi Kahaloa‘a has provided numerous specimens for this revision, most often from difficult to access natural areas, and from months of the year with very few other records. Thus it is a pleasure to honor her contributions to *Mecyclothorax* diversity by naming this species in her honor.

##### Distribution and habitat.

**Figure 67.**
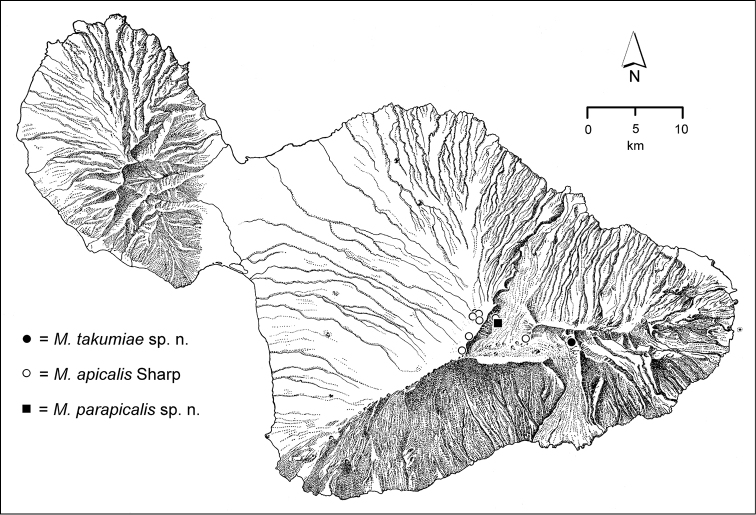
Recorded geographic distributions of *Mecyclothorax ovipennis* group species.

The type specimen was collected at 1950 m elevation in a pitfall trap near Paliku Cabin. The site lies in the mesic eastern end of Haleakalā Crater, and it receives windward moisture wafting over the highest elevations of Kīpahulu Valley to the east (Fig. 67).

#### (049) Mecyclothorax apicalis

Taxon classificationAnimaliaColeopteraCarabidae

(Sharp)

Figs 61F–H , 62C , 63C , 65C , 67

Thriscothorax apicalis Sharp 1903: 264; Britton 1948b: 150.

##### Diagnosis.

This species and *Mecyclothorax parapicalis* share: 1, evident microsculpture, isodiametric to transverse, on the vertex and pronotal disc; 2, divergent pronotal lateral margins anterad obtuse, glabrous hind angles; 3, shallow, impunctate discal elytral intervals that are of similar color to lateral elytral intervals 7–9. The pronotum is more basally constricted in individuals of this species—MPW/BPW = 1.46–1.54—and more transverse—MPW/PL = 1.26–1.33—than in beetles comprising *Mecyclothorax parapicalis* (Fig. 65C–D). The elytra are also narrower basally, with the lateral margins little extended laterally behind the tightly rounded humeri (Fig. 65C). The male aedeagal median lobe is distinctively different from that of *Mecyclothorax parapicalis*, with the apex sinuously recurved with an expanded, spoonlike tip (Figs 61F, G). Individuals of this species vary in the degree of punctation in the discal elytral striae. The striae vary from smooth, impunctate, to more irregular due to the presence of elongate punctulae (Fig. 65C). This variation is taken into account in the dichotomous key above. Setal formula 2 1 2 0. Standardized body length 4.0–4.2 mm.

##### Identification

(n = 5). Characters of the pronotum can assist identification, with the median base depressed relative to the disc, the surface strigose laterally and sparsely punctate medially. The anterior transverse impression is shallow, broad, with sparse longitudinal wrinkles behind. The laterobasal depression is slightly convex medially, and depressed laterally and basally along the beaded pronotal margin. On the elytra, the narrower elytral base is associated with a very narrow marginal depression at the humeri, with the narrow marginal depression concolorous with the elytral intervals. Overall, the body coloration is dark: 1, vertex rufobrunneous with piceous cast; 2, pronotal and elytral disc rufopiceous; 3, elytral apex narrowly flavous along margins (Fig. 65C). The frons and vertex bear well-developed isodiametric sculpticells in transverse rows. The pronotal disc is covered with isodiametric to slightly transverse sculpticells in transverse rows, the isodiametric microsculpture on the median base more upraised, again arranged in transverse rows.

**Male genitalia** (n = 4). Aedeagal median lobe slender, distance between parameral articulation and tip 5× depth at midlength (Fig. 61F); apex sinuously extended beyond apex of ostial opening, dorsoventrally expanded near bluntly rounded tip; median lobe straight overall in ventral view, the apex offset to right relative to the shaft, tip blunt (Fig. 61H); internal sac with ventral ostial microtrichial patch, flagellar plate very small, length 0.24× parameral articulation-tip distance (Fig. 61G).

**Female reproductive tract** (n = 1). Bursa copulatrix columnar, elongate, length 0.91 mm, breadth 0.39 mm (Fig. 62C); bursal walls moderately thick, wrinkled; gonocoxite 1 with 1–2 apical fringe setae, and only 2–4 very small setae on medial half of gonocoxite (Fig. 63C); gonocoxite 2 broadly triangular with laterally curved apex, base with broad lateral extension, 2 lateral ensiform setae with apical seta broader and longer (a minute third, basal ensiform seta present unilaterally), apical nematiform setae on medial surface at 0.68× gonocoxite length.

##### Lectotype.

Female (BMNH) hereby designated, labeled: Thriscothorax apicalis Type D.S. Haleakala Perkins 254 // Type // Hawaiian Is. Perkins 1904-336. // LECTOTYPE Thriscothorax apicalis Sharp J.K. Liebherr 1998 (black-margined red label).

##### Distribution and habitat.

*Mecyclothorax apicalis* is a species of open *Deschampsia nubigena* (hairgrass) grasslands, both along the upper northwest slope and in Haleakalā Crater (Fig. 67). Beetles have been found in the moist soil of bunchgrass tufts, and by pitfall trapping. Although the species has been collected recently across its range, the Argentine Ant, *Linepitheme humile* (Mayr), represents a threat to persistence of its populations when the two are sympatric, as they are on the northwest slope. Ant presence has been shown to have a statistically significant negative effect on beetle abundance in that situation (Liebherr and Krushelnycky 2007).

#### (050) Mecyclothorax parapicalis sp. n.

Taxon classificationAnimaliaColeopteraCarabidae

http://zoobank.org/FA8A66EB-7FD6-4D80-A801-1A16FF0B1D79

Figs 61I , 65D , 67

##### Diagnosis.

Like *Mecyclothorax apicalis*, but the body is broader at the elytral base, and the elytral lateral margins are slightly extended laterally behind the humeri, the humeral angle defined by a slight hitch at the base of the moderately broad lateral marginal depression (Fig. 65D). The microsculpture on the forebody is more transverse, with: 1, a shallow transverse mesh, sculpticell breadth 2–3× length on the vertex; 2, transverse mesh, sculpticell breadth 3× length, to transverse lines on the pronotal disc; and 3, evident isodiametric and transverse sculpticells over the pronotal median base. The male aedeagal median lobe exhibits a rhomboidal apex, with the apical and ventral margins meeting at an acute angle (Fig. 61I).

Setal formula 2 1 2 0. Standardized body length—3.8–3.9 mm—is slightly smaller than that of *Mecyclothorax apicalis*; length 4.0–4.2 for that species.

##### Description

(n = 3). *Head capsule* with frontal grooves broad near clypeus, a broad lateral carina to anterior supraorbital seta; dorsal surface of neck flat to slightly concave; eyes moderately convex but covering much of ocular lobe, ocular ratio = 1.41–1.46, ocular lobe ratio = 0.82–0.84; labral anterior margin medially emarginate 1/6 of length; antennae submoniliform, antennomeres 2–3 with sparse pelage of short setae; mentum tooth with sides acute, apex tightly rounded. *Pronotum* transverse, MPW/PL = 1.28–1.36, broad basally, MPW/BPW = 1.41–1.45; hind angle slightly obtuse, margin rounded behind or not, lateral margin subparallel or slightly divergent immediately anterad angle; median base slightly depressed relative to disc, smoother medially, laterally punctate, longitudinal wrinkles lining juncture with disc; basal margin broadly, slightly convex between laterobasal depressions; median longitudinal impression very shallow, finely incised; anterior transverse impression deep, finely incised, shallower at midline; anterior callosity slightly convex, smooth, glossy; front angles slightly produced, tightly rounded; pronotal apical width slightly narrower than basal width, APW/BPW = 0.93–0.97; lateral marginal depression narrow throughout, edge beaded except where slightly broader at front angles; laterobasal depression broadly convex between median base and hind angle. *Proepisternum* with 5 minute punctures along hind marginal groove; prosternal process with narrow median impression, margins beaded between coxae. *Elytra* subquadrate, disc flat, sides distinctly sloped; basal groove distinctly recurved inside humeral angle; elytra broadest behind midlength and humeri extended laterally in concert with broad pronotal base, MEW/HuW = 1.95–2.0; parascutellar seta present; parascutellar striole with 5 punctures, shallow but continuous between punctures; sutural interval flat in basal half, progressively elevated along suture to apex; sutural and 2^nd^ striae of subequal depth from base to apex; discal striae 1–4 smooth, shallow, striae 5–6 shallower, traceable, stria 7 absent; discal elytral intervals 2–6 only slightly convex to nearly flat on lateral intervals; 8^th^ interval slightly more convex than apical fused portion of striae 5 + 7; 2 dorsal elytral setae at 0.32× and 0.67× elytral length, setal impressions small, spanning ½ width of interval 3; apical and subapical setae absent; lateral elytral setae arranged in anterior series of 7 setae and posterior series of 6 setae; elytral marginal depression of moderate breadth at humerus, narrowed laterad posterior setal series, margin upturned except beaded at subapical sinuation; subapical sinuation shallow, more abruptly incurved anteriorly. *Mesepisternum* with ~6 punctures in 1–2 rows; metepisternal width to length ratio = 0.75; metepisternum/metepimeron suture distinct. *Abdomen* with irregular lateral wrinkles on ventrites 1–5, and lateral depressions on ventrites 3–6; suture between ventrites 2 and 3 reduced laterally, effaced; apical male ventrite with 2 marginal setae. *Legs*-metatarsomere 1/metatibial length ratio = 0.19; metatarsomere 4 length along outer lobe 1.33× medial tarsomere length, apical and subapical setae present; metatarsal dorsolateral sulci narrow, shallow, median area broad. *Microsculpture* of elytral disc consisting of distinct isodiametric and transverse sculpticells arranged in a mesh, elytral apex with transverse mesh, sculpticell breadth 2× length; metasternum with distinct transverse mesh; laterobasal abdominal ventrites with swirling isodiametric and transverse microsculpture. *Coloration* of vertex rufobrunneous; antennomeres 1–3 rufoflavous, 4–11 darker, brunneous; pronotal disc rufobrunneous with piceous cast, lateral margins, base, and apex narrowly rufobrunneous; proepipleuron rufoflavous, proepisternum rufobrunneous; elytral disc rufobrunneous with slightly metallic reflection, sutural interval concolorous basally, rufoflavous apically; intervals 7–8 inside humeral angle plus lateral marginal depression rufoflavous; elytral apex contrastedly flavous from apical terminus of interval 4; elytral epipleuron dorsally rufoflavous, ventrally rufobrunneous, metepisternum rufobrunneous; abdomen with ventrites 1–6 medially rufopiceous, ventrites 3–6 laterally rufoflavous, abdominal apical ventrite with apical 1/6 paler, flavous; metafemur flavous; metatibia flavous with brunneous cast.

**Male genitalia** (n = 1). Aedeagal median lobe robust, distance between parameral articulation and tip 3.8× depth at midlength (Fig. 61I); apex trapezoidal, with flat apical face and angled ventral margin meeting at tightly rounded tip; internal sac with diffusely developed dorsal ostial microtrichial patch, flagellar plate short, length 0.3× parameral articulation-tip distance.

##### Holotype.

Male (BPBM) labeled: HAWAII: E. Maui I: / Haleakala Nat. Park / Haleakala Crater / 2134 m 1.VIII.1973 / *Deschampsia nubigena* / W.C. Gagné Coll. / BISHOP Museum // Mecyclothorax / parapicalis / ♂ photo / det. J.K. Liebherr 2014 // HOLOTYPE / Mecyclothorax / parapicalus / Liebherr / det. J.K. Liebherr 2015 (black-margined red label).

##### Paratypes.

Same data as holotype (BPBM, 1; CUIC, 1)

##### Etymology.

The similarity of this species to *Mecyclothorax apicalis* makes the species epithet parapicalis appropriate; the adjectival epithet meaning like apicalis

##### Distribution and habitat.

The distribution of *Mecyclothorax parapicalis* is completing subsumed by that of the very similar appearing species *Mecyclothorax apicalis* (Fig. 67). This species is known only from three specimens collected by Dr. Wayne Gagné in association with *Deschampsia nubigena* (hairgrass) in the vicinity of Holua Cabin. The specimens were collected during August, whereas all specimens of the closely related *Mecyclothorax apicalis* have been collected in March, April, June, July, and October.

#### (051) Mecyclothorax mauiae sp. n.

Taxon classificationAnimaliaColeopteraCarabidae

http://zoobank.org/1FF2BB69-8C7A-47EC-BCF0-8C4B32C5B422

**Figure 68.**
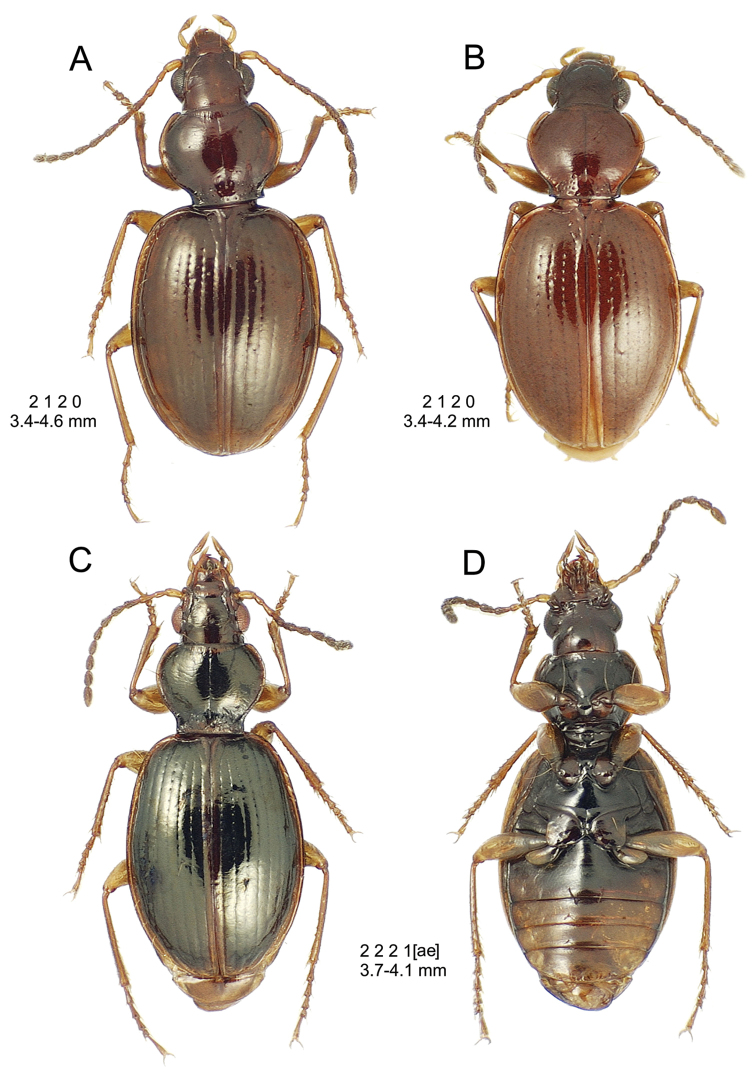
*Mecyclothorax ovipennis* group species, habitus view. **A** *Mecyclothorax mauiae* (Ke‘anae, 1325 m) **B** *Mecyclothorax subternus* (Kuhiwa E rim, 880 m) **C–D** *Mecyclothorax flaviventris* (Kīpahulu, 1500 m) **C** Dorsal view **D** Ventral view.

**Figure 69.**
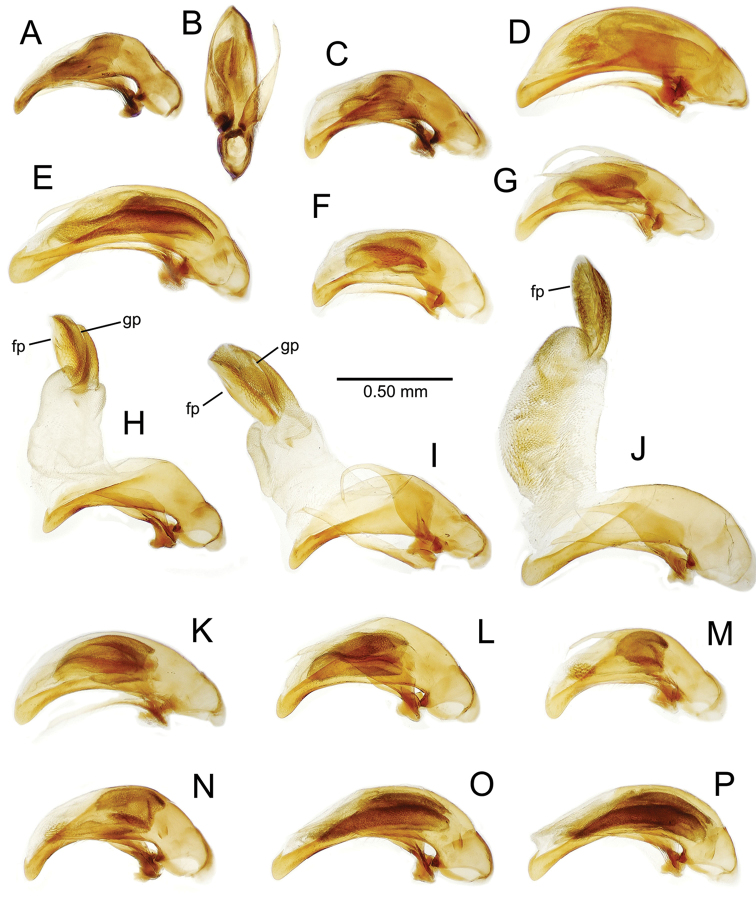
Male aedeagus, *Mecyclothorax mauiae* (for abbreviations see Table 2, p. 23). **A–B** Right and ventral view (Waikamoi, 1305 m) **D–G** Right view **D** (Waikamoi, 1160 m). **E** (Ke‘anae, 1325 m) **F** (Kopili‘ula, 1127 m). **G** (Kuhiwa, 1590 m) **H–J** Right view, sac everted **H** (Kuhiwa, 1580 m) **I** (Helele‘ike‘oha, 1615 m) **J** (Kīpahulu, 910 m) **K–P** Right view. **K** (Kīpahulu, 1500 m) **L** (Kīpahulu W rim, 1850 m) **M** (Pu‘u Ahulili, 1600 m) **N** (Ka‘āpahu, 1250 m) **O** (Midcamp Bog, 1665 m) **P** (Kuhiwa E rim, 880 m).

Figs 62D–E , 63D , 68A , 69 , 71

##### Diagnosis.

**Figure 70.**
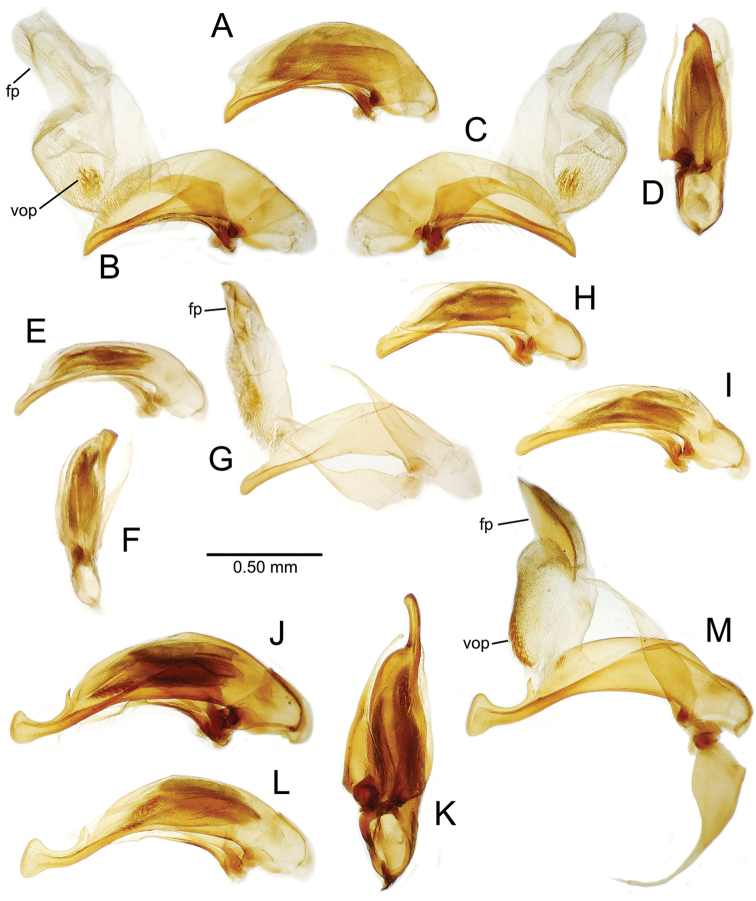
Male aedeagus, *Mecyclothorax ovipennis* group species (for abbreviations see Table 2, p. 23). **A–D** *Mecyclothorax subternus* (Kuhiwa E rim, 880 m). **A** Right view **B–C** Right and left views, sac everted **D** Ventral view **E–I** *Mecyclothorax flaviventris* **E** Right view (Kīpahulu, 1845 m) **F** Ventral view (Kīpahulu, 1845 m) **G** Right view, sac everted (Kīpahulu, 1845 m) **H** Right view (Kīpahulu, 1500 m) **I** Right view (Paliku, 1950m) **J–M** *Mecyclothorax laetus* **J–K** Right and ventral views (Kīpahulu, 1900 m) **L** Right view (Kīpahulu, 1960 m) **M** Right view, sac everted (NW upper slope, 2072 m).

Among the assemblage of *Mecyclothorax ovipennis* group species with punctate discal elytral striae and concolorous elytral intervals, this and the next species, *Mecyclothorax subternus*, stand out due to their broadly ellipsoid to obovoid elytra (Fig. 68A–B). The pronotum characterizing these species is also very constricted basally—MPW/BPW = 1.58–1.72 for this species—with a minutely punctate, glossy median base. *Mecyclothorax mauiae* can be diagnosed from *Mecyclothorax subternus* by the better developed microsculpture: 1, vertex with evident transverse mesh, sculpticell breadth 2× length; and 2, pronotal and elytral discs and elytral apex with transverse mesh, sculpticell breadth 2–3× length. The eyes also tend to be less convex and slightly smaller than in *Mecyclothorax subternus*, with ocular ratio = 1.43–1.50, and ocular lobe ratio = 0.75–0.79 in this species. The final arbiter for any identification involving a male specimen is the apex of the aedeagal median lobe, with the apex always rounded in male of this species (Fig. 69) versus acuminate in *Mecyclothorax subternus* (Fig. 70A–D). The degree of strial development varies among individuals of this species, potentially allowing specimens of this species to be confused with those of *Mecyclothorax nanunctus* of the *Mecyclothorax palustris* group. That group is characterized by a less depressed apex on elytral stria 2; a character exhibiting some infraspecific variation. Specimens of *Mecyclothorax mauiae* always exhibit broader pronotal lateral marginal depressions, and more ellipsoid elytra than observed in individuals of *Mecyclothorax nanunctus*. As with *Mecyclothorax subternus*, examination of male genitalia will finalize the diagnosis, as the male aedeagal median lobe of *Mecyclothorax nanunctus* is much more elongate and gracile, with the apex terminated in narrowly projected tip (Fig. 153D–J). Setal formula 2 1 2 0. Standardized body length 3.4–4.5 mm.

##### Description

(n = 5). *Head capsule* with frontal grooves broad near clypeus, lateral carina to supraorbital seta; dorsal surface of neck flat to slightly concave; labral anterior margin very shallowly emarginate medially; antennae filiform, antennomeres 2–3 with sparse pelage of short setae; mentum tooth with sides acute, apex rounded. *Pronotum* cordate, moderately transverse, MPW/PL = 1.23–1.27; hind angle right to slightly acute, lateral margin convergent just before angle; median base almost coplanar to slightly depressed relative to disc, ~12 sparsely distributed punctures each side; basal margin convexly expanded between laterobasal depressions; median longitudinal impression shallow, finely incised, crossed by fine transverse wrinkles; anterior transverse impression narrow, shallowly incised laterally, obsolete medially; anterior callosity slightly convex, smooth to slightly irregular due to fine wrinkles, surface glossy; front angles slightly projected, rounded; pronotal apical width greater than basal width, APW/BPW = 1.03–1.11; lateral marginal depression moderately narrow, edge beaded anterad lateral seta, broader at front angle, edge little upturned in basal half; laterobasal depression narrow and deep, continuous with lateral depression. *Proepisternum* with 5 minute punctures along hind marginal groove; prosternal process with narrow median impression, lateral margins broadly beaded between coxae. *Elytra* broadly obovoid, disc narrowly flat medially, sides steeply sloped; basal grooves briefly recurved to proximate humeral angles, the tightly rounded to subangulate angles defined by hitch at the base of the lateral marginal depression, MEW/HuW = 2.28–2.51; parascutellar seta present; parascutellar striole with 4 isolated punctures, striole may be discontinuous between adjacent punctures; sutural interval slightly more convex than lateral intervals, sutural juncture upraised; sutural stria shallow between deep, round punctures basally, smooth, moderately deep apically, 2^nd^ stria shallower but also with rounded punctures on disc, shallower and broader apically, the two striae of subequal depth at elytral apex; discal striae 3–4 very shallow, punctate on disc, stria 5 very shallow, traceable, striae 6–7 obsolete, associated inner intervals slightly convex, lateral intervals flat; 8^th^ interval convex, though striae are obsolete in that area of elytra; 2 dorsal elytral setae at 0.31× and 0.56–0.58× elytral length, setal impressions shallow, spanning ½ to 2/3 width of interval 3; apical and subapical setae absent; lateral elytral setae arranged in anterior series of 7 setae and posterior series of 6 setae; elytral marginal depression slightly broader at humerus, narrowed laterally and posteriorly to beadlike at subapical sinuation; subapical sinuation shallow, concavity symmetrical. *Mesepisternum* with ~8 punctures in 1–2 rows; metepisternal width to length ratio = 0.70; metepisternum/metepimeron suture distinct. *Abdomen* with irregular lateral wrinkles on ventrites 1–5, lateral depressions on ventrites 3–6; suture between ventrites 2 and 3 complete; apical male ventrite with 2 marginal setae; apical female ventrite with 4 equally spaced marginal setae and median trapezoid of 4, subequal, short setae. *Legs*-metatarsomere 1/metatibial length ratio = 0.18; metatarsomere 4 length along outer lobe 1.4× medial tarsomere length, apical and subapical setae present; metatarsal dorsolateral sulci narrow, shallow, median area broad. *Microsculpture* of pronotal median base obsolete medially, the surface glossy, a transverse mesh present laterally between punctures; metasternum with shallow transverse mesh; laterobasal abdominal ventrites with swirling isodiametric and transverse microsculpture. *Coloration* of vertex rufobrunneous; antennomere 1 flavous, antennomeres 2–3 brunneous, 4–11 with piceous cast; pronotal disc rufobrunneous, lateral margins narrowly, and base and apex rufous; proepipleuron rufoflavous, proepisternum rufobrunneous; elytral disc rufobrunneous basally, apical half the same with piceous cast, sutural interval rufous throughout, intervals 8–9 rufoflavous, paler apically; elytral epipleuron dorsally rufoflavous, ventrally darker, metepisternum rufobrunneous; abdomen with ventrites 1–3 rufobrunneous, ventrites 4–5 darker, rufopiceous, apical ventrite 6 rufoflavous mediobasally, apical half flavous; metafemur flavous; metatibia flavous with brunneous cast.

**Male genitalia** (n = 50). Aedeagal median lobe variably robust, distance between parameral articulation and tip 2.2–3.3× depth at midlength (Fig. 69E, M), but apex always little extended beyond ostial opening, with tip broadly rounded (Fig. 69); median lobe symmetrical in ventral view, broadest at midlength of lobe shaft, evenly narrowed to blunt tip which lies on right side of apex (Fig. 69B); internal sac broad, of variable length (Fig. 69H–J), sac length from ostium to base of flagellar plate 0.77–1.1× parameral articulation-tip distance, surface unornamented except denser microspicules may occur on ventral surface (Fig. 69J); flagellar plate very large, length 0.60–0.67× parameral articulation-tip distance.

**Female reproductive tract** (n = 2). Bursa copulatrix columnar, elongate, length 1.08–1.16 mm, breadth 0.33–0.36 mm, an apical lobe offset from basal portion by curved constriction, apical lobe 0.49 mm long (Fig. 62D–E); bursal walls translucent with thick wrinkles; gonocoxite 1 with 3–4 apical fringe setae, the outermost setae of series smallest, and 5–8 smaller setae on medial surface (Fig. 63D); gonocoxite 2 falcate with tightly rounded tip, base with lateral panhandle extension with 90° bend at terminus, 2 lateral ensiform setae with apical seta longer and broader, apical nematiform setae on medial surface at 0.68× gonocoxite length.

##### Holotype.

Male (CUIC) labeled: HI: Maui Haleakala NW / slope Waikamoi Flume / Waikamoi to Haipuaena / Gulches 11-IV-1991 / el. 1300 m J. Liebherr // under boards in / wet ohia rain / forest // HOLOTYPE / Mecyclothorax / mauiae / Liebherr / det. J.K. Liebherr 2015 (black-margined red label).

##### Paratypes.

817 specimens (see Appendix).

##### Etymology.

This species’ similarity to *Mecyclothorax molokaiae* of Moloka‘i, suggests use of mauiae as the species epithet, the first declension genitive singular form to mean Maui’s *Mecyclothorax*.

##### Distribution and habitat.

**Figure 71.**
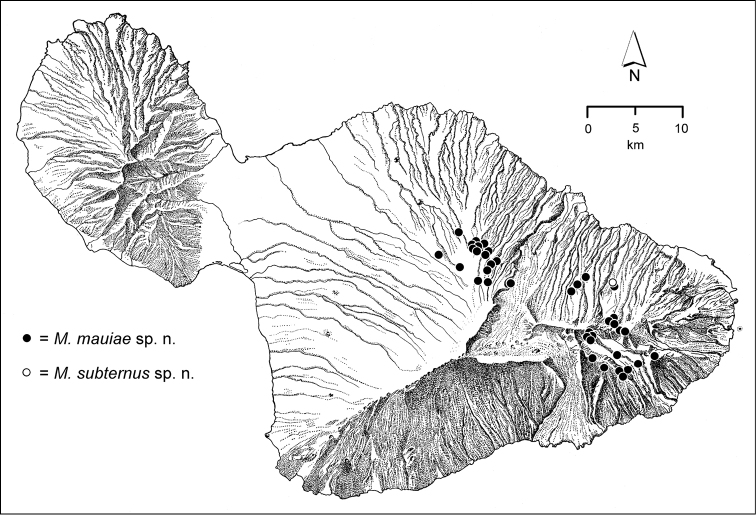
Recorded geographic distributions of *Mecyclothorax ovipennis* group species.

*Mecyclothorax mauiae* is broadly distributed across the windward face of Haleakalā, including Kīpahulu Valley, Kaumakani Peak and the Manawainui Planeze (Fig. 71). Collection localities range 880–1830 m elevation. This species is found in association with a variety of plant substrates, with ‘ōhi‘a and *Cibotium* (hāpu‘u) tree ferns most commonly associated with collections. Beetles have also been collected infrequently on koa trunks, and very commonly in sift samples of ‘ōhi‘a litter. Whereas the predominantly ground-dwelling species of the *Mecyclothorax robustus* and *Mecyclothorax sobrinus* species groups were trapped extensively in yellow-pan traps in the Waikamoi area (see above), only six specimens of *Mecyclothorax mauiae* were so trapped at one wet forest site (Kula Pipeline Road, 1183–1280 m elevation, vi-viii-2006, L. LeBlanc, UHIM).

#### (052) Mecyclothorax subternus sp. n.

Taxon classificationAnimaliaColeopteraCarabidae

http://zoobank.org/85C310CB-52E4-4042-9FF3-661479FBF112

Figs 62F , 63E , 68B , 70A–D , 71

##### Diagnosis.

Individuals of this species (Fig. 68B) are nearly identical externally to those of *Mecyclothorax mauiae* (Fig. 68A), though the eyes tend to be larger and more convex; ocular ratio = 1.48–1.54, ocular lobe ratio = 0.79–0.82, and the dorsal surface is less sculptured; 1, vertex, pronotal disc and median base, and elytral disc glossy, microsculpture obsolete; 2, elytral apex with shallow to obsolete transverse mesh, the surface glossy; 3, metasternum with shallow transverse-mesh microsculpture. The male aedeagal median lobe certifies the identification, as the apex terminates in a pointed tip (Fig. 70A–C) instead of the broadly rounded apex of *Mecyclothorax mauiae* (Fig. 69). Setal formula 2 1 2 0. Standardized body length 3.4–4.2 mm.

##### Description

(n = 5). The description of *Mecyclothorax mauiae* also serves for this species with the following substitutions. Pronotum slightly broader basally and more transverse than that of *Mecyclothorax mauiae*; MPW/BPW = 1.57–1.65, MPW/PL = 1.23–1.32. Pronotal lateral margin narrow, beaded, not wider at front angles. Discal elytral striae shallow between deep, rounded and nearly isolated punctures; discal elytral interval 2 slightly convex, intervals 3–4 nearly flat. Mesepisternum smooth, with 3–4 punctures arrayed in 1 row. Coloration pale (though single topotypic type series may include exclusively somewhat teneral individuals); vertex rufous; antennomere 1 flavous, antennomeres 2–3 rufoflavous, 4–11 brunneous; pronotal disc and margins pale rufous; elytral disc basally rufous, apically rufobrunneous, sutural interval basally rufoflavous, apically flavous; abdominal ventrites 1–5 mediobasally rufous, laterally and apically rufobrunneous; metafemur flavous with medial brunneous cloud.

**Male genitalia** (n = 2). Aedeagal median lobe robust, distance between parameral articulation and tip 2.5× depth at midlength (Fig. 70A–C); apex little extended beyond ostial opening, tip acutely pointed; median lobe symmetrical in ventral view, right margin broadly concave, left margin incurved to blunt tip which is on right side of apex (Fig. 70D); internal sac very broad basally, with oblong ventral ostial microtrichial patch on right side (Fig. 70B); flagellar plate very large, length 0.67× parameral articulation-tip distance.

**Female reproductive tract** (n = 1). Bursa copulatrix a broad sac, length 1.23 mm, maximum breadth in apical half 0.65 mm, breadth at vagina 0.34 mm (Fig. 62F); bursal walls translucent with thick wrinkles; gonocoxite 1 with 3 apical fringe setae, middle seta of series largest, 8 smaller setae along medial margin (Fig. 63E); gonocoxite 2 falcate with acuminate tip, base with sinuous lateral extension, 2 lateral ensiform setae with apical seta broader and longer, apical nematiform setae on medial surface at 0.72× gonocoxite length.

##### Holotype.

Male (NMNH) labeled: HI:Maui Haleakala Hana- / wi NAR Kuhiwa Vy. E rim / 9-VI-1999 lot 07 880 m el. / N20°46'25", W156°06'04" / D.A. Polhemus pyr. fog / *Cibotium* // Mecyclothorax / subternus / ♂ #40 / det. J.K. Liebherr 2014 // HOLOTYPE / Mecyclothorax / subternus / Liebherr / det. J.K. Liebherr 2015 (black-margined red label).

##### Paratypes.

Same data as holotype (BPBM, 2; CUIC, 3; NMNH, 5).

##### Etymology.

The adjectival species epithet subternus, meaning that which is underneath (Jaeger 1955), signifies the geographical range of this species being at the lower elevational bounds of its closely related, and much more broadly distributed relative, *Mecyclothorax mauiae* (Fig. 71).

##### Distribution and habitat.

*Mecyclothorax subternus* is known only from a single locality along the lower elevational limit of the range of its much more extensively distributed cryptic sibling species, *Mecyclothorax mauiae* (Fig. 71). All 10 specimens of this species came from a single day of collecting on mossy ‘ōhi‘a trunks and logs. The type locality in Kuhiwa Valley is extremely wet both due to precipitation but also due to the extensive rainwater runoff from the upper elevations of Kuhiwa Valley. Yearly rainfall at 871 m elevation in Kuhiwa Valley averaged 9.75 m from 1934–1941 (Stearns and McDonald 1942); the highest average total for any station in East Maui during those years.

#### (053) Mecyclothorax flaviventris sp. n.

Taxon classificationAnimaliaColeopteraCarabidae

http://zoobank.org/EBE87BAF-89FE-48CE-B36A-E62C8A2FF878

**Figure 72.**
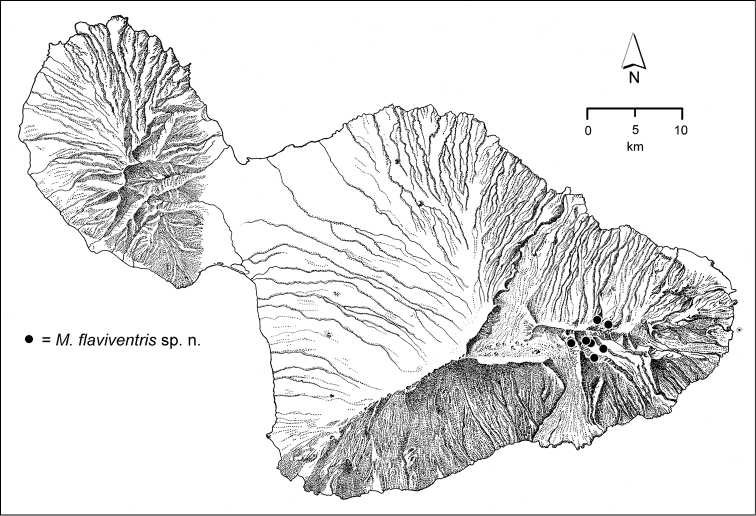
Recorded geographic distribution of *Mecyclothorax flaviventris*.

Figs 62G , 63F , 68C–D , 70E–I , 72

##### Diagnosis.

This species is the most gracile-bodied *Mecyclothorax ovipennis* group species with cordate pronotum, narrowed elytral humeri, and punctate discal elytral intervals (Fig. 68C). In body conformation it is most similar to *Mecyclothorax ovipennis* (Fig. 65A), and both species share the setal formula 2 2 2 1[ae]. However individuals of this species lack the parascutellar seta. Also, this species is uniquely characterized among species in the group by the apically flavous abdomen, with the flavous ventrites 4–6 contrasted to the rufobrunneous basal ventrites (Fig. 68D). Standardized body length 3.7–4.1 mm.

##### Description

(n = 5). *Head capsule* with frontal grooves broad near clypeus, lateral carina to supraorbital seta; dorsal surface of neck flat to slightly concave; eyes large and moderately convex, ocular ratio = 1.51–1.56, ocular lobe ratio = 0.82–0.88; labral anterior margin deeply angulate medially, emarginated 1/3 of length; antennae filiform; antennomeres 2–3 with only a few short setae along shafts; mentum tooth with sides acute, apex tightly rounded. *Pronotum* quadrisetose, distinctly constricted basally, MPW/BPW = 1.49–1.58, with lateral margins convergent before the right to slightly acute, projected hind angles; pronotum appearing narrow, but actually slightly transverse, MPW/PL = 1.13–1.21; median base broadly depressed relative to disc, rugose medially, longitudinally strigose laterally; basal margin very slightly convex between laterobasal depressions; median longitudinal impression evident, finely incised, continued onto median base; anterior transverse impression broad, surface with granulate microsculpture and deep, dense longitudinal wrinkles; anterior callosity depressed, covered with longitudinal wrinkles from transverse impression; front angles not projected, tightly rounded; pronotal apical width greater than basal width, APW/BPW = 1.06–1.09; lateral marginal depression moderately narrow, edge upturned, slightly broader inside front angle; laterobasal depression slightly transversely wrinkled, continuous with lateral depression. *Proepisternum* with 4–5 minute punctures along hind marginal groove; prosternal process with narrow median impression, lateral margins broadly beaded between coxae. *Elytra* distinctly ovoid with maximal width behind midlength, disc narrowly flat medially, sides steeply sloped; basal groove gently recurved to subangulate humerus that is defined by hitch at base of lateral marginal depression, MEW/HuW = 2.10–2.18; parascutellar striole very shallow, smooth, difficult to trace; sutural interval slightly more convex than lateral intervals, but depressed at suture; sutural and 2^nd^ striae of subequal depth from base to apex, striae 2–6 progressively shallower on disc, stria 7 discontinuous, obsolete, discal striae 1–4 with small punctures restricted to deepest parts of striae; 8^th^ interval slightly more convex than apical fused portion of striae 5 + 7; 2 dorsal elytral setae at 0.30–0.31× and 0.61–0.66× elytral length, setal impressions shallow, spanning ½ width of interval 3; apical elytral seta present near medial apex of interval 3, subapical seta absent; lateral elytral setae arranged in anterior series of 7 setae and posterior series of 6 setae; elytral marginal depression moderately narrow, edge upraised until beaded at subapical sinuation; subapical sinuation shallow, more abruptly incurved anteriorly. *Mesepisternum* with ~8 punctures in 1–2 rows; metepisternal width to length ratio = 0.71; metepisternum/metepimeron suture distinct. *Abdomen* with irregular lateral wrinkles on ventrites 1–5, lateral depressions on ventrites 3–6 (Fig. 68D); suture between ventrites 2 and 3 reduced laterally, effaced; apical male ventrite with 2 marginal setae, apical female ventrite with 4 equally spaced setae plus median trapezoid of 4 subequal, short setae. *Legs*-metatarsomere 1/metatibial length ratio = 0.21; metatarsomere 4 length along outer lobe 1.33× medial tarsomere length, apical and subapical setae present; metatarsal dorsolateral sulci narrow, shallow, median area broad. *Microsculpture* of vertex and pronotal disc a transverse mesh, sculpticell breadth 2–3× length; pronotal median base with granulate isodiametric and transverse mesh, sculpticell breadth 2× length; elytral disc with shallow isodiametric and transverse mesh, sculpticell breadth 2× length, in transverse rows; elytral apex with shallow isodiametric sculpticells in transverse rows; metasternum with upraised transverse mesh; laterobasal abdominal ventrites with swirling isodiametric and transverse microsculpture. *Coloration* of vertex rufobrunneous with piceous cast; antennomeres 1–3 flavous, 4–11 darker, more brunneous; pronotal disc rufopiceous, lateral margins, base, and apex narrowly rufoflavous; proepipleuron rufoflavous, proepisternum rufobrunneous; elytral disc dark rufobrunneous, sutural interval narrowly rufobrunneous basally, rufoflavous apically; elytral marginal depression and 9^th^ interval rufoflavous, elytral apex narrowly flavous; elytral epipleuron dorsally flavous, ventrally rufoflavous, metepisternum rufopiceous; abdomen with ventrites 1–2 rufobrunneous, ventrite 3 medially rufobrunneous, laterally and apically flavous, ventrites 3–6 flavous; metafemur flavous; metatibia flavous with a brunneous cast.

**Male genitalia** (n = 5). Aedeagal median lobe variably gracile, distance between parameral articulation and tip 3.7–4.2× depth at midlength (Fig. 70E, H, I); apex extended twice its depth beyond apex of ostial opening, tip slightly downturned, rounded; median lobe curved rightward toward apex in ventral view (Fig. 70F), right margin concave, left margin incurved to blunt tip; internal sac elongate, length from ostial margin to base of flagellar plate 0.79× parameral articulation-tip distance, sac surface unornamented but with microspicules more developed over entire ventral surface (Fig. 70G); flagellar plate moderately short, length 0.36× parameral articulation-tip distance. The male from Kīpahulu Valley, 1500 m elevation (Fig. 70H) exhibits a broader apex, though the internal sac in its uneverted condition looks identical to the configuration of the Kīpahulu Valley, 1845 m elevation male (Fig. 70E). All of these specimens exhibit the flavous abdominal ventrites that represent an autapomorphy for the species, and if the lower elevation Kīpahulu Valley population is determined to be distinct, the additionally recognized species will be the sister to *Mecyclothorax flaviventris*. A present, with only a single specimen from the lower Kīpahulu Valley locality, such recognition is deemed premature, and the aedeagal conformations are assumed to represent infraspecific variability.

**Female reproductive tract** (n = 1). Bursa copulatrix columnar with rounded, slightly expanded apex, length 0.68 mm, breadth 0.29 mm (Fig. 62G); bursal walls translucent with thin wrinkles; gonocoxite 1 with 0–1 apical fringe setae and 7–8 smaller setae on medial surface (Fig. 63F); gonocoxite 2 falcate with tightly rounded tip, base with long, thin lateral extension with 90° bend at terminus, 2 lateral ensiform setae, the apical seta longer and broader, apical nematiform setae on medial surface at 0.69× gonocoxite length.

##### Holotype.

Male (CUIC) dissected and labeled: HI:Maui Haleakala N.P. / Kipahulu Vy. 1500 m el. / 9-V-1991 sifting / leaf litter by day // S. Jessel / A.C. Medeiros, / Jr. collectors // Mecyclothorax / flaviventris / ♂ #4 / det. J.K. Liebherr 2014 // HOLOTYPE / Mecyclothorax / flaviventris / Liebherr / det. J.K. Liebherr 2015 (black-margined red label).

##### Paratypes.

35 specimens (see Appendix).

##### Etymology.

The Latin genitive singular flaviventris signifies the flavous abdominal ventrites 4–6 contrasted to the piceous abdominal base.

##### Distribution and habitat.

*Mecyclothorax flaviventris* has a distribution that is centered on the upper elevations of Kīpahulu Valley, extended northward into the Hāna Bogs, westward to the eastern mesic margin of Haleakalā Crater at Paliku, and southward to Kīpahulu’s west rim near Kuiki (Fig. 72). Collecting localities span 1500–2100 m elevation. The beetles have almost always been collected in association with mossy ‘ōhi‘a trunks and logs. Several beetles have been encountered while beating vegetation, and one was captured in a pitfall trap at Paliku.

#### (054) Mecyclothorax laetus

Taxon classificationAnimaliaColeopteraCarabidae

(Blackburn)

**Figure 73.**
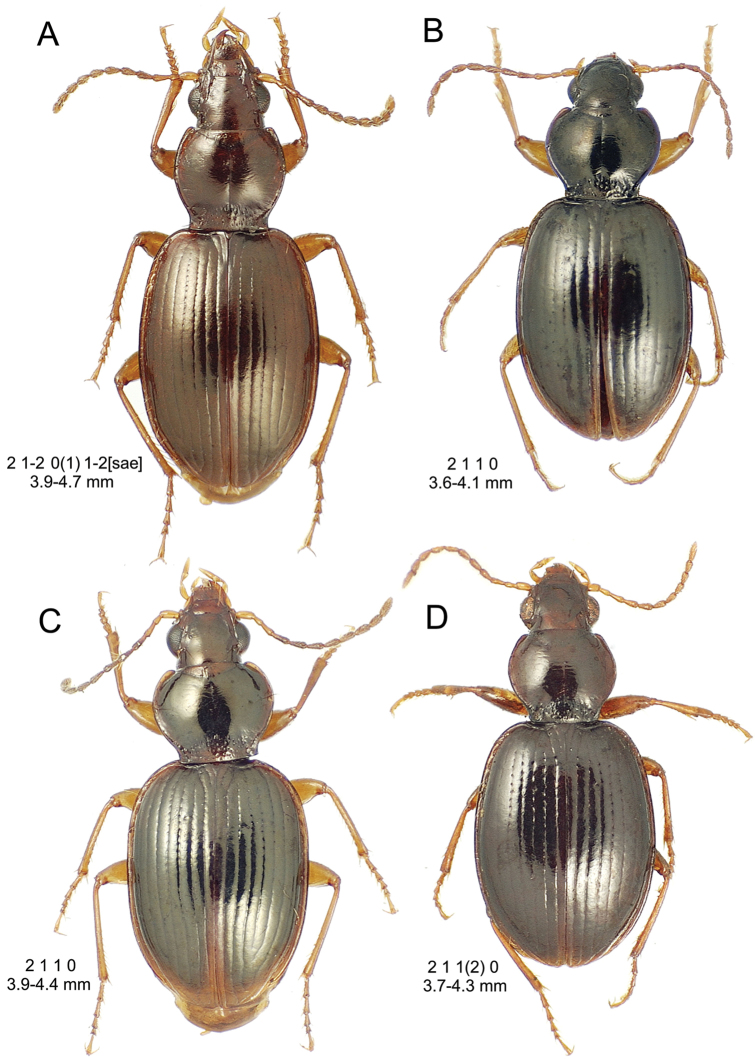
*Mecyclothorax ovipennis* group species, dorsal habitus view. **A** *Mecyclothorax laetus* (Kīpahulu, 1900 m) **B** *Mecyclothorax cordaticollis* (Olinda-Ukulele Camp Pipeline, 1210–1524 m) **C** *Mecyclothorax cordaticollaris* (Kaupō Gap, 1170 m) **D** *Mecyclothorax subconstrictus* (summit, 2895–3050 m).

**Figure 74.**
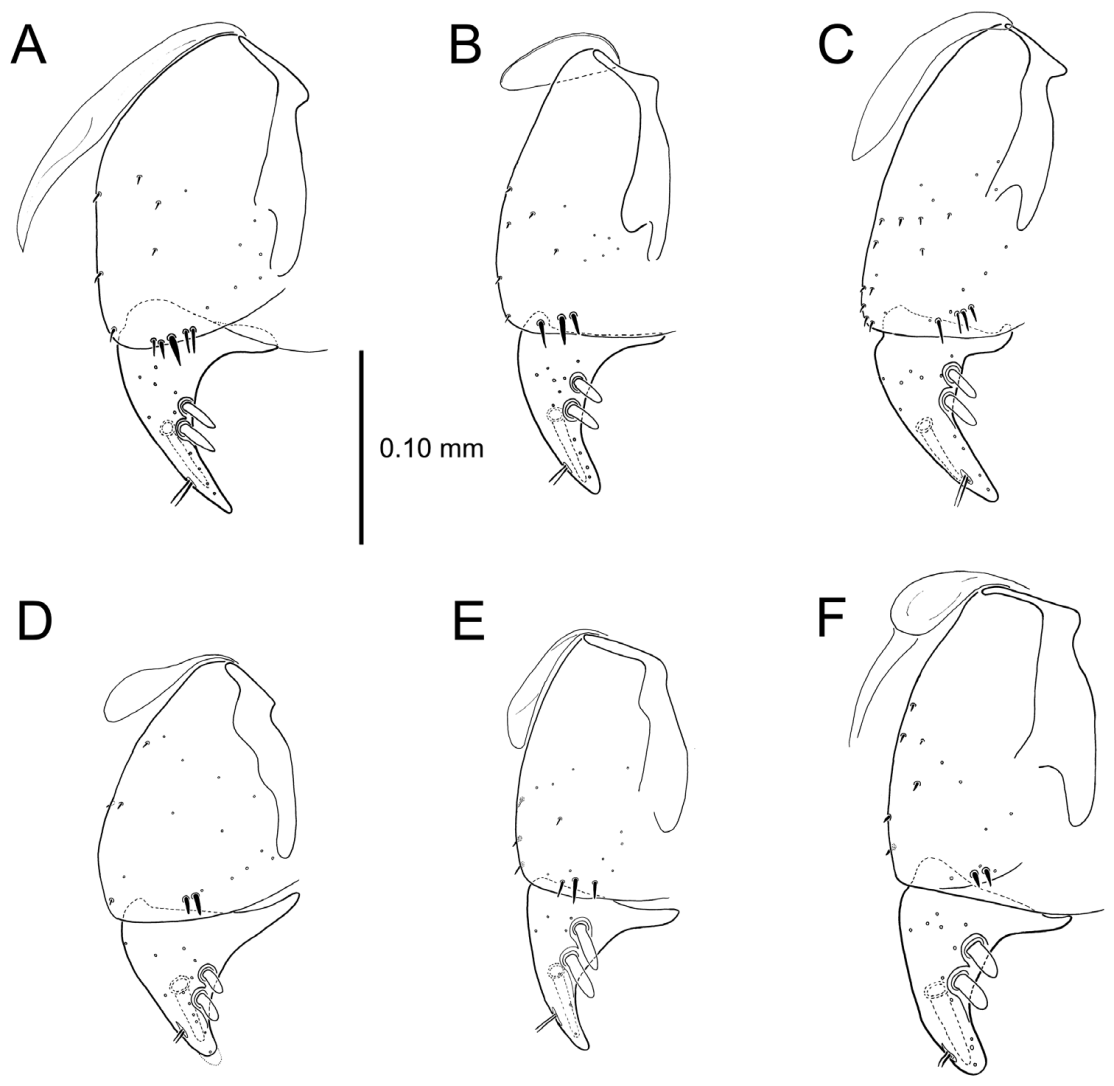
Left female gonocoxa, *Mecyclothorax ovipennis* group species, ventral view. **A** *Mecyclothorax laetus* (Ukulele Camp Pipeline, 1495–1525 m) **B** *Mecyclothorax cordaticollis* (nr. Ukulele Camp, 1525 m) **C** *Mecyclothorax cordaticollaris* (Kaupō Gap, 1495 m) **D** *Mecyclothorax subconstrictus* (summit, 2895–3050 m) **E** *Mecyclothorax pusillus* (summit, 2895–3050 m) **F** *Mecyclothorax rusticus* (summit, 2895–3050 m).

**Figure 75.**
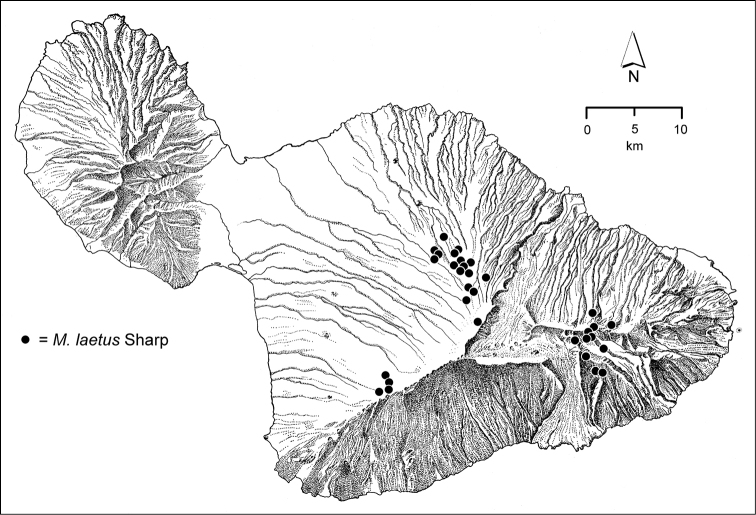
Recorded geographic distribution of *Mecyclothorax laetus*.

Figs 62H , 70J–M , 73A , 74A , 75

Cyclothorax laetus Blackburn 1881: 228; Blackburn and Sharp 1885: 216.
Thriscothorax laetus , Sharp 1903: 262.
Mecyclothorax laetus Sharp 1903: 247; Britton 1948b: 149 (synonymy, subsequent homonymy); Liebherr 2005b: 111.
Thriscothorax subconstrictus , Swezey 1954: 53, 60 (misidentification, *Cibotium* and rotten wood associate).

##### Diagnosis.

The rufobrunneous dorsal body color, narrow, cordate pronotum, and well-developed dorsal microsculpture–isodiametric on the elytra–will allow individuals of this species to be identified in the field using a hand lens (Fig. 73A). More microscopic characters amply diagnosis this species, including: 1, elytral parascutellar seta absent; 2, dorsal elytral setae absent, though a single seta may be present in rare instances; 3, vertex with shallow transverse mesh, sculpticell breadth 2× length, and pronotal disc with transverse mesh, sculpticell breadth 2–3× length. Setal conformation is highly variable among individuals of this species, with the basal pronotal setae present or absent, and either both apical and subapical elytral setae, or just the subapical seta present. Setal formula 2 1-2 0(1) 1-2[sae]. Standardized body length 3.9–4.6 mm.

##### Identification

(n = 5). The eyes are slightly to moderately convex, ocular ratio = 1.41–1.46, covering more than ¾ of the little protruded ocular lobe; ocular lobe ratio = 0.77–0.82. The antennae are submoniliform, with antennomeres 5–11 expanded apically. The pronotal lateral margin is distinctly concave anterad the right to acute hind angle, the basal sinuation extended for 1/7 the length of the pronotum. The pronotum is narrow to slightly transverse, MPW/PL = 1.13–1.23, with a somewhat variable basal constriction; MPW/BPW = 1.39–1.51. The narrow subquadrate elytra are flat medially, with discal striae 1–5 shallow and minutely punctate, stria 6 obsolete but traceable, and stria 7 absent. All intervals save the slightly convex sutural interval are only slightly convex to flat. When a single dorsal elytral seta is present, it is in the basal position; 0.24× elytral length.

**Male genitalia** (n = 3). Aedeagal median lobe moderately robust, distance between parameral articulation and tip 3.4× depth at midlength (Fig. 70J, L); apex sinuously extended beyond ostial opening, apex dorsoventrally expanded, either more dorsally than ventrally (Fig. 70J), or broadly both ventrally and dorsally (Fig. 70L–M); median lobe straight in ventral view, but thin elongate apex offset toward right side of shaft, with right margin concave basad apex, and left margin distinctly incurved to meet apex, tip appearing tightly rounded from ventral aspect (Fig. 70K); internal sac broad, with broad, diffuse ventral ostial microtrichial field, otherwise covered only with fine microspicules (Fig. 70M); flagellar plate moderately large, length 0.49× parameral articulation-tip distance. That the variably expanded apex represents infraspecific variation is supported by both narrower and broader apices (Fig. 70J, L) being found in males from Kīpahulu Valley, West Camp, 1900–1960 m elevation.

**Female reproductive tract** (n = 1). Bursa copulatrix columnar, apical lobe set off by constriction, reminiscent of a ginger jar with small lid; overall length 0.83 mm, apical lobe 0.25 mm long × 0.45 mm broad, shaft breadth 0.44 mm, and basal constriction 0.26 mm broad at vagina (Fig. 62H); bursal walls translucent, thinly wrinkled basally, apical lobe more wrinkled and less stained; gonocoxite 1 with 5 apical fringe setae, 6 smaller setae—1 at medioapical angle—along medial surface (Fig. 74A); gonocoxite 2 falcate with pointed apex, base broadly extended laterally, 2 short lateral ensiform setae, apical nematiform setae on medial surface at 0.77× gonocoxite length.

##### Lectotypes.

For *Cyclothorax laetus* Blackburn, male designated by Liebherr (2005b: 113). For *Mecyclothorax laetus* Sharp, female designated by Liebherr (2005b: 114). Type locality for the former is Haleakala, Maui, ~4000 ft.; i.e. the Waikamoi area; type locality for the latter is Haleakala, Maui, 5000 ft. (R.C.L.P. lot 661; Anonymous N D), i.e. near Ukulele Camp.

##### Distribution and habitat.

*Mecyclothorax laetus* exhibits a very broad geographic distribution (Fig. 75), though the species is extensively absent from the Ke‘anae Valley and Hanawī face of Haleakalā. This absence is made the more peculiar by the very broad ecological preference suggested for this species by the disparate arrays of collecting situations. Consistent with occupation of the forests west of Waikamoi Gulch, this species has been found on mossy ‘ōhi‘a trunks, on koa trunks, and associated with *Cibotium* (hāpu‘u) ferns. But it has also been collected by sifting soil around *Deschampsia* (hairgrass) clumps in open shrubland. More exotically, it has been collected in large numbers under the loose bark of downed alien *Pinus ponderosa* in a disturbed grassland on the northwest slope. And in the alien afforested areas of Polipoli Springs it has been found by grubbing in deep pine needle litter. The presence of free moisture is a constant in all of these situations, though the degree of solar insolation varies dramatically.

#### (055) Mecyclothorax cordaticollis

Taxon classificationAnimaliaColeopteraCarabidae

(Blackburn)

**Figure 76.**
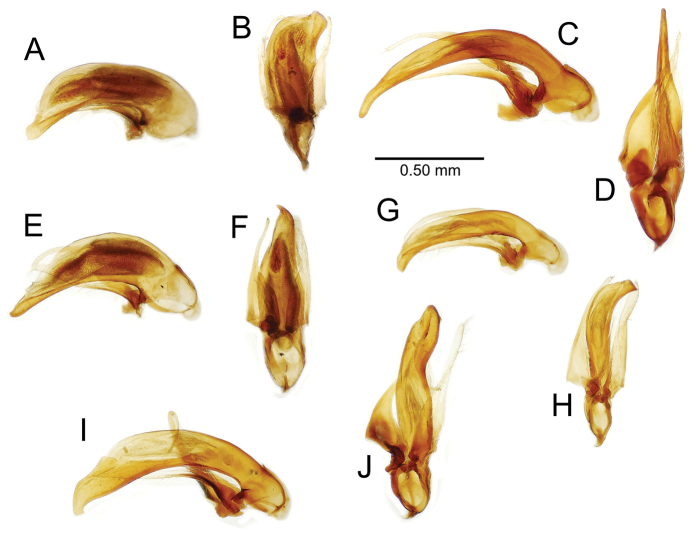
Male aedeagus, *Mecyclothorax ovipennis* group species. **A–B** *Mecyclothorax cordaticollis*, right and ventral views (Olinda-Ukulele Camp Pipeline, 1210–1524 m) **C–D** *Mecyclothorax cordaticollaris*, right and ventral views (Kaupō Gap, 1495 m) **E–F** *Mecyclothorax subconstrictus*, right and ventral views (summit, 2895–3050 m) **G–H** *Mecyclothorax pusillus*, right and ventral views (summit, 2895–3050 m) **I–J** *Mecyclothorax rusticus*, right and ventral views (summit, 2895–3050 m).

**Figure 77.**
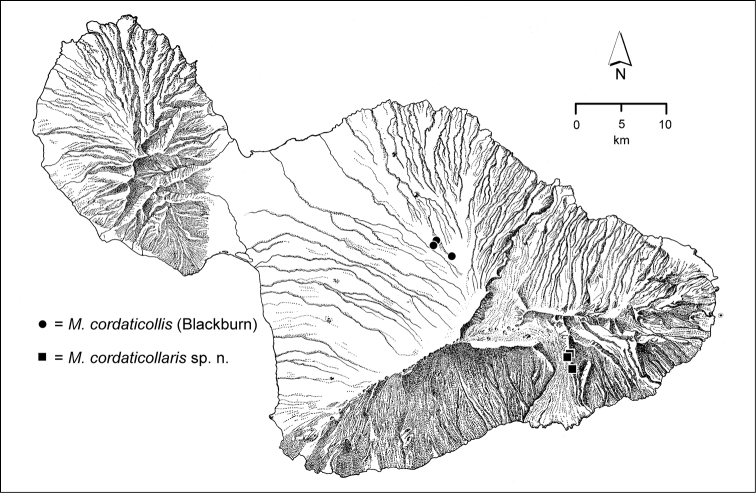
Recorded geographic distributions of *Mecyclothorax ovipennis* group species.

Figs 62I , 73B , 74B , 76A–B , 77

Cyclothorax cordaticollis Blackburn 1878b: 156; Blackburn and Sharp 1885: 215.
Thriscothorax cordaticollis , Sharp 1903: 259.
Mecyclothorax cordaticollis , Britton 1948b: 148.
Thriscothorax modestus Sharp 1903: 259; Britton 1948b: 148 (synonymy).

##### Diagnosis.

This species (Fig. 73B), *Mecyclothorax cordaticollaris* (Fig. 73C), and *Mecyclothorax subconstrictus* (Fig. 73D) are the only three *Mecyclothorax ovipennis* group species to exhibit the 2 1 1 0 setal formula (at least for some of the individuals of the latter species). *Mecyclothorax cordaticollis* and *Mecyclothorax subconstrictus* share shallow, minutely punctate discal elytral intervals and associated slightly convex intervals, plus a narrowly constricted pronotum; MPW/BPW = 1.55–1.61 in this species. The discal elytral striae are much more punctate in *Mecyclothorax cordaticollaris*, and the associated intervals are much more convex. *Mecyclothorax cordaticollis* and *Mecyclothorax subconstrictus* can be definitively diagnosed using dorsal microsculpture. The pronotal disc of *Mecyclothorax cordaticollis* bears transverse-mesh microsculpture, sculpticell breadth 2–3× length, and the elytral disc is covered with a transverse mesh, sculpticell breadth 2–4× length. For *Mecyclothorax subconstrictus*, the pronotal disc is glossy medially, and laterally covered with an obsolete transverse mesh, whereas the elytral disc bears a mixture of isodiametric and transverse sculpticells. Standardized body length 3.6–4.1 mm.

##### Identification

(n = 4). The eyes are moderately convex, ocular ratio = 1.42–1.49, but they cover much of the ocular lobe, ocular lobe ratio = 0.81–0.86. The pronotal lateral marginal depression is narrow, the edge upturned anterad lateral seta, slightly broader near front angle, and thin and beadlike along basal sinuation. The pronotal apical width is greater than the basal width; APW/BPW = 1.01–1.06. The elytra are subquadrate, with the tightly rounded humeral angles connected medially to only slightly recurved basal grooves. Though all the striae are shallow, they are all of similar depth on the elytral apex.

**Male genitalia** (n = 1). Aedeagal median lobe short, squat, distance between parameral articulation and tip 2.5× depth at midlength (Fig. 76A); apex extended beyond ostial opening equal to its depth, tip broadly rounded; median lobe curved rightward apically in ventral view (Fig. 76B), right margin slightly concave near tip, left margin broadly convex; internal sac with ventral ostial microtrichial patch indicated in uneverted specimen, shadow of flagellar plate also visible, the plate quite elongate, length 0.67× parameral articulation-tip distance.

**Female reproductive tract** (n = 1). Bursa copulatrix columnar, length 0.84 mm, breadth 0.34–0.38 mm (Fig. 62I); bursal walls translucent, thinly wrinkled; gonocoxite 1 with 3 apical fringe setae, middle seta larger, 1 small seta at apicomedial angle, and 4–5 setae on medial surface (Fig. 74B); gonocoxite 2 broadly subtriangular, apex tightly rounded, base moderately extended laterally, 2 short lateral ensiform setae, apical nematiform setae on medial surface at 0.75× gonocoxite length.

##### Lectotypes.

For *Cyclothorax cordaticollis*, female (BMMH) hereby designated, labeled: mounting platen with Blackburn Maui label (Zimmerman 1957: 210), cord (on reverse) // Type // Hawaiian Is. Rev. T. Blackburn 1888-30. // LECTOTYPE Cyclothorax cordaticollis Blackburn J.K. Liebherr 1998 (black-margined red label). For *Thriscothorax modestus*, male (BMNH) hereby designated, labeled: Thriscothorax modestus Type D.S. Haleakala 350 // Type // Hawaiian Is. Perkins 1904-336. // LECTOTYPE Thriscothorax modestus J.K. Liebherr 1998 (black-margined red label).

##### Distribution and habitat.

*Mecyclothorax cordaticollis* is known from only three definitive localities in the leeward forests west of Waikamoi Gulch (Fig. 77). Ukulele Camp (Site) is the most likely locality given Perkins’ (1894, 1896b) field notes. There no collecting records since Perkins collected two specimens in 1902.

#### (056) Mecyclothorax cordaticollaris sp. n.

Taxon classificationAnimaliaColeopteraCarabidae

http://zoobank.org/FC1D5329-71E8-4C07-81AF-A6F974CB832B

Figs 62J , 73C , 74C , 76C–D , 77

##### Diagnosis.

Like *Mecyclothorax cordaticollis* (Fig. 73B) or *Mecyclothorax subconstrictus* (Fig. 73D) in the setal formula—2 2 1 0—but with much deeper elytral striae, the discal striae 1–5 distinctly punctate with the punctures expanding strial breadth (Fig. 73C). The pronotal median base is minutely punctate, ~15 distinct punctures each side, with the punctures elongate at the juncture with the disc. The pronotal disc is glossy, with obsolete transverse-mesh microsculpture, sculpticell breadth 3× length, and the median base is glossy medially, and with an isodiametric mesh present laterally between the punctures. The elytra are relatively broader basally than in *Mecyclothorax cordaticollis* or *Mecyclothorax subconstrictus*, with the basal groove distinctly recurved to meet the angulate humerus, the angle defined by a hitch at the base of the lateral marginal depression. Standardized body length 3.9–4.4 mm.

##### Description

(n = 4). *Head capsule* with frontal grooves broad near clypeus, straight, lateral carina to anterior supraorbital seta; dorsal surface of neck flat to slightly concave; eyes moderately convex, ocular ratio = 1.48–1.50, ocular lobe ratio = 0.78–0.82; labral anterior margin very shallow emarginate medially; antennae filiform, antennomeres 2–3 with sparse pelage of short setae; mentum tooth with sides right, apex rounded. *Pronotum* transverse, MPW/PL = 1.24–1.27, constricted basally, MPW/BPW = 1.47–1.55; hind angle right, lateral margins subparallel to slightly convergent anterad projected angles; basal margin slightly, evenly convex between laterobasal depressions; median longitudinal impression shallow, finely incised; anterior transverse impression moderately deep, smooth, finely incised; anterior callosity slightly convex, smooth, glossy; front angles very slightly produced, broadly rounded; apical and basal pronotal widths subequal, APW/BPW = 0.95–1.0; lateral marginal depression narrow, edge upturned anterad seta, slightly broader at front angle, beadlike margin from midlength to basal sinuation; laterobasal depression smooth laterad median base, broadly raised in explanate lateral margin. *Proepisternum* with 5 minute punctures along hind margin; prosternal process with narrow median impression, lateral margins broadly beaded between coxae. *Elytra* broadly ovoid, disc moderately convex, sides more so; MEW/HuW = 2.11–2.16; parascutellar seta present; parascutellar striole with 4 punctures, continuous between punctures; sutural interval only slightly more convex than lateral intervals in basal half, more convex apically; sutural and 2^nd^ striae of subequal depth from base to apex; elytral intervals 2–4 moderately convex, lateral intervals flatter; 8^th^ interval slightly more convex than fused apical portion of striae 5 + 7; one dorsal elytral seta at 0.23–0.28× elytral length, setal impression small, spanning ½ width of interval 3; apical and subapical setae absent; lateral elytral setae arranged in anterior series of 7 setae and posterior series of 6 setae; elytral marginal depression narrow, margin upturned, beadlike near subapical sinuation; subapical sinuation shallow, more abruptly incurved anteriorly. *Mesepisternum* with ~10 punctures in 2–3 rows; metepisternal width to length ratio = 0.65; metepisternum/metepimeron suture distinct. *Abdomen* with irregular lateral wrinkles on ventrites 1–5 and lateral depressions on ventrites 3–6; suture between ventrites 2 and 3 reduced laterally, effaced; apical male ventrite with 2 marginal setae, apical female ventrite with 4 equally spaced marginal setae plus median trapezoid of 4 subequal, short setae. *Legs*-metatarsomere 1/metatibial length ratio = 0.21; metatarsomere 4 length along outer lobe 1.25× medial tarsomere length, apical and subapical setae present; metatarsal dorsolateral sulci shallow, narrow, median area broad. *Microsculpture* of vertex distinct, transversely stretched, sculpticell breadth 2–3× length; elytral disc with shallow transverse mesh, sculpticell breadth 3–4× length, apex with more developed transverse mesh of same dimensions; metasternum with shallow transverse mesh; laterobasal abdominal ventrites with swirling isodiametric and transverse microsculpture. *Coloration* of vertex rufobrunneous; antennomeres 1–3 flavous, 4–11 darker, more brunneous; pronotal disc rufobrunneous with piceous cast, lateral margins, base, and apex rufoflavous; proepipleuron rufoflavous, proepisternum rufobrunneous with piceous cast; elytral disc rufobrunneous, sutural interval paler rufous basally, rufoflavous apically; elytral lateral marginal depression narrowly rufoflavous, apex contrastedly flavous from apical terminus of interval 4; elytral epipleuron rufoflavous, metepisternum rufopiceous; abdominal ventrites 1–2 rufopiceous, ventrites 3–5 medially rufopiceous, laterally paler, apical ventrite with apical half flavous; metafemur flavous; metatibia flavous with brunneous cast.

**Male genitalia** (n = 1). Aedeagal median lobe extremely slender, apically narrowed, needlelike, distance from parameral articulation to tip 5.9× depth at midlength (Fig. 76C); apex elongate, very narrow, angled slightly downward about half way along apical extension, with tip narrowly rounded; median lobe straight in ventral view, right and left margins approaching each other for 1/3 lobe length in this view, tip narrowly pointed (Fig. 76D); internal sac without apparent microtrichial fields in uneverted specimen, flagellar plate length estimated to be 0.35× parameral articulation-tip distance.

**Female reproductive tract** (n = 1). Bursa copulatrix broad basally at vagina, with elongate, digitiform apical lobe, overall bursal length 0.74 mm, with apical lobe 0.51 mm long × 0.19 mm broad, and basal bulb at vagina 0.23 mm long × 0.41 mm broad (Fig. 62J); bursal walls smooth, only lightly wrinkled, the walls of apical lobe thinner, less stained than broad base with darker staining and thicker wrinkles; gonocoxite 1 with 4 apical fringe setae, medial surface lined with 7–10 smaller setae (Fig. 74C); gonocoxite 2 falcate with subacuminate apex, base broadly extended by short panhandle with curved terminus, 2 short lateral ensiform setae with apical seta longer and broader, apical nematiform setae on medial surface at 0.68× gonocoxite length.

##### Holotype.

Male (CUIC) dissected and labeled: HI: Maui Haleakala N.P. / Kaupo Gap el. 1160 m / N20°40'43", W156°08'09" / 17-V-2001 lot 05 beating / ferns J.K. Liebherr // 1 // HOLOTYPE / Mecyclothorax / cordaticollaris / Liebherr / det. J.K. Liebherr 2015 (black-margined red label).

##### Paratypes.

HI: Maui, Kaupo Gap Tr., beating *Pipturus*, 1340 m el., 31-viii-1996 lot 01, Ewing (CUIC, 1), sifting *Acacia koa*/fern/moss litter, 1495 m el., 17–18-v-2001 lot 03, Liebherr (CUIC, 1), same data as holotype (CUIC, 1).

##### Etymology.

This species’ great similarity to *Mecyclothorax cordaticollis* leads to use of the similar epithet cordaticollaris. As in the former name, this adjectival epithet is meant to signify the cordate pronotum.

##### Distribution and habitat.

*Mecyclothorax cordaticollaris* is distributed in the Koa Mesic Forest lining the eastern margin of Kaupō Gap (Fig. 77). Specimens have been collected from 1170–1495 m elevation in litter including fern and moss humus plus koa leaves and phyllodes, as well as by beating low soft ferns and *Pipturus* (māmaki).

#### (057) Mecyclothorax subconstrictus

Taxon classificationAnimaliaColeopteraCarabidae

(Sharp)

Figs 62K , 73D , 74D , 76E–F , 79

Thriscothorax subconstrictus Sharp 1903: 259.
Mecyclothorax subconstrictus , Britton 1948b: 147.

##### Diagnosis.

This, the third species of the group to exhibit setal formula 2 1 1 0 for at least some individuals, can be diagnosed from the other two—*Mecyclothorax cordaticollis* (Fig. 73B) and *Mecyclothorax cordaticollaris* (Fig. 73C)—by the following combination: 1, pronotal hind angles obtuse, lateral margin divergent from angle (Fig. 73D), not parallel before angle; 2, elytra broader relative to pronotal width, MEW/MPW = 1.53–1.66, versus values of 1.47–1.52 observed in specimens of the other two species; 3, elytral disc with mixture of isodiametric and transverse sculpticells, sculpticell breadth 2× length, versus more transverse meshes in the other two species. Setal formula 2 1 1(2) 0; individuals have a posterior dorsal elytral seta present unilaterally in rare instances. Standardized body length 3.7–4.3 mm.

##### Identification

(n = 5). The eyes are slightly larger and more convex than observed in *Mecyclothorax cordaticollis* and *Mecyclothorax cordaticollaris*—ocular ratio = 1.46–1.52, ocular lobe ratio = 0.81–0.88—though the ratios for the three species overlap. In aggregate the pronotal apex is broader relative to the base; APW/BPW = 1.03–1.09. The impressions of the anterior dorsal elytral setae span 2/3 of interval 3, whereas the impression of a posterior seta, if present, is small, spanning half or less of interval 3. The body coloration overall is also darker in specimens of this species versus those of the former two; 1, vertex and pronotal disc rufobrunneous with piceous cast; 2, pronotal lateral margins dark and concolorous with disc, though base and apex paler, rufoflavous.

**Male genitalia** (n = 1). Aedeagal median lobe robust, distance between parameral articulation and tip 2.9× depth at midlength (Fig. 76E); apex extended twice its depth beyond ostial opening, tip tightly rounded but with minute denticle along dorsal margin; median lobe slightly curved rightward in ventral view, left and right sides subparallel as they moderately converge to blunt-appearing tip (Fig. 76F); internal sac covered with dark microspicules, flagellar plate elongate, length 0.50× parameral articulation-tip distance.

**Female reproductive tract** (n = 1). Bursa copulatrix broadly ellipsoid with constricted base, length 0.88 mm, breadth 0.44 mm, basal constriction at vagina 0.31 mm broad (Fig. 62K); bursal walls translucent with thin wrinkles; gonocoxite 1 with 2 short apical fringe setae and 4–6 small setae along medial surface (Fig. 74D); gonocoxite 2 broadly triangular with broadly rounded apex (apex worn), 2 lateral ensiform setae with apical seta longer and broader, apical nematiform setae on medial surface at 0.72× gonocoxite length.

##### Lectotype.

(BMNH) hereby designated, left specimen on mounting platen, labeled: Thriscothorax subconstrictus Type D.S. Haleakala Perkins // Type // Hawaiian Is. Perkins 1904-336. // LECTOTYPE Thriscothorax subconstrictus Sharp J.K. Liebherr 1998 (black-margined red label).

##### Distribution and habitat.

*Mecyclothorax subconstrictus* is one of four species in the *Mecyclothorax ovipennis* group that occupy the highest elevations at the summit of Haleakalā (Figs 79, 80). Most specimens of these species were collected 9-iv-1894 during Perkins’ ascent of Haleakalā from Ukulele Camp. In Perkins’ words: “April 9^th^. Started with rug, food and gun etc. for Blackburn’s cave at about 9000 feet. I got there about 10 a.m. although I went down to nearly Olinda before ascending, as I wished to go on the usual trail. Worked from 9000 ft. to the summit. Got 3 or 4 species of Cyclothorax, the large punctured species as below, [i.e. Mecyclothorax montivagus, common from 4000–10000 ft.] the small ones all new to me apparently.” He returned to the summit two days later, collecting many more *Mecyclothorax* specimens. Besides *Mecyclothorax subconstrictus*, the species new to Perkins were *Mecyclothorax pusillus* and *Mecyclothorax rusticus*. These two collecting hikes occurred “a few weeks after the disappearance of the snow-cap (Perkins 1913, cxl).” *Mecyclothorax subconstrictus* was collected again in May and October 1896, the latter time at Holua in Haleakalā Crater. It has not been recollected since.

#### (058) Mecyclothorax nubicola

Taxon classificationAnimaliaColeopteraCarabidae

(Blackburn)

Figs 78A , 79

Cyclothorax nubicola Blackburn 1878b: 156.
Mecyclothorax nubicola , Sharp 1903: 244; Britton 1948b: 150.
Cyclothorax rupicola (lapsus calami), Blackburn and Sharp 1885: 216 (Sharp 1903: 244).

##### Diagnosis.

**Figure 78.**
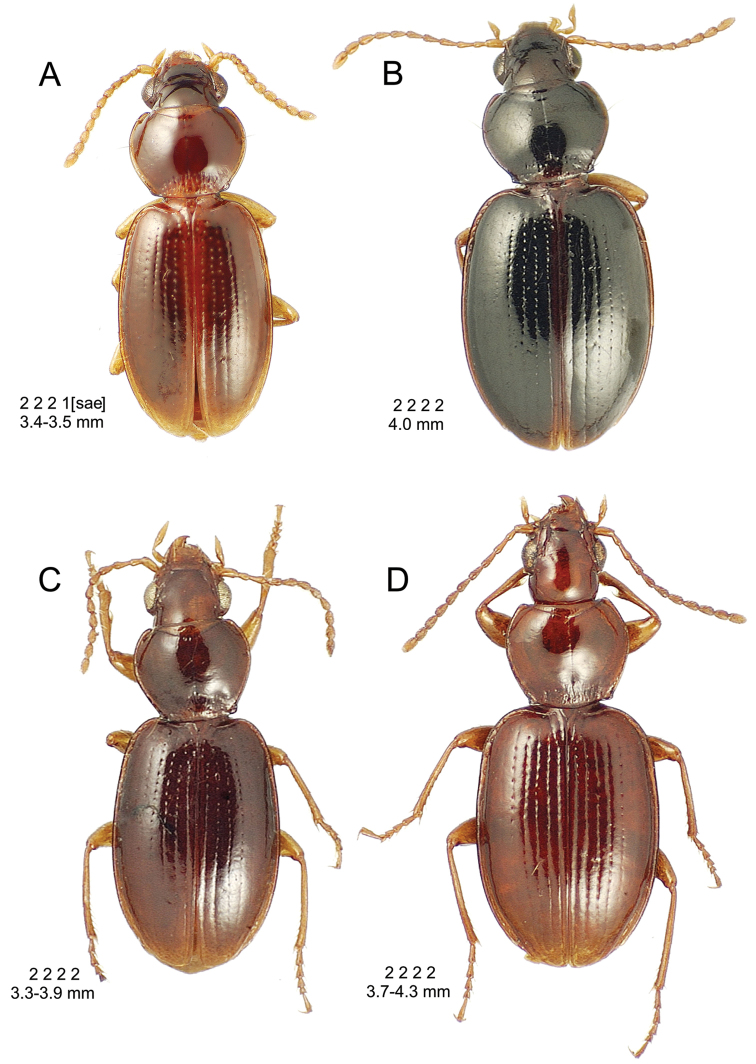
*Mecyclothorax ovipennis* group species, dorsal habitus view. **A** *Mecyclothorax nubicola* (Leleiwi, 2650 m) **B** *Mecyclothorax krushelnyckyi* (Kahikinui, 2400 m) **C** *Mecyclothorax pusillus* (summit, 2895–3050 m) **D** *Mecyclothorax rusticus* (summit, 2895–3050 m).

This species is uniquely characterized by the very broad pronotum and the remarkably narrow, subparallel elytra (Fig. 78A); MEW/MPW = 1.31–1.36. The only other Haleakalā species to approach this conformation is *Mecyclothorax pusillus* (Fig. 78C). The pronotum of *Mecyclothorax nubicola* also distinctively diagnoses the species, with the hind angles represented by a slight widening of the lateral marginal bead, with the lateral margin only slightly sinuate anterad the very obtuse angle. Also, this species is composed of small beetles; standardized body length 3.4–3.5 mm. The setal formula of 2 2 2 2 is shared with the following three species; *Mecyclothorax krushelnyckyi*, *Mecyclothorax pusillus*, and *Mecyclothorax rusticus*. All four species also lack the parascutellar seta.

##### Identification

(n = 3). The eyes are moderately convex; ocular ratio = 1.46–1.52, ocular lobe ratio 0.81–0.82. The pronotal disc is smooth, with a fine median longitudinal impression and moderately deep, finely incised anterior transverse impression. The median base contrasts to the disc as it is rugose, and is covered with ~10 densely distributed, elongate punctures each side, with fine longitudinal wrinkles at the base-disc juncture. The elytral basal groove is distinctly recurved laterally, with the humeral angle defined by a hitch at the base of the lateral marginal depression; MEW/HuW = 1.80–1.94. Discal elytral striae 1–3 are continuous, punctate, whereas stria 4 is interrupted along its length, and stria 5 is a series of punctures. Microsculpture is reduced in this species, with the frons and vertex glossy and covered with an obsolete transverse mesh. The pronotal disc has transverse lines in part, the cuticle glossy between these areas of microsculpture, whereas the pronotal median base is glossy medially, with irregular sculpticells laterally. The elytral disc bears very shallow isodiametric sculpticells in transverse rows.

##### Holotype.

Female (BMNH): mounting platen with Blackburn Maui label (Zimmerman 1957: 210), Cyc nubicola (on reverse) // Type // Hawaiian Is. Rev. T. Blackburn 1888-30 // HOLOTYPE Cyclothorax nubicola Blackburn J.K. Liebherr 1998 (black-margined red label).

##### Distribution and habitat.

**Figure 79.**
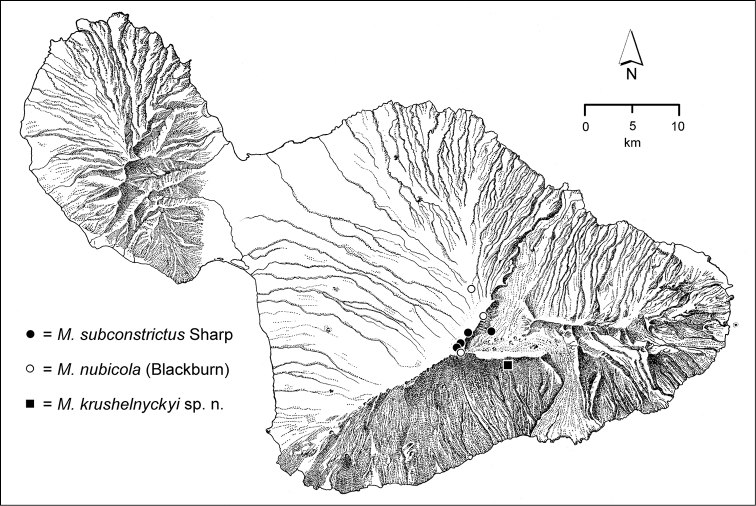
Recorded geographic distributions of *Mecyclothorax ovipennis* group species.

*Mecyclothorax nubicola* is a fourth summit-dwelling *Mecyclothorax* species, but it was not collected by Perkins in 1894 or 1896. The species is known from only four specimens and three localities (Fig. 79); the holotype described by Blackburn (1878b) from 10,000 ft. (3040 m), a Timberlake specimen (UCRC) from “gulch near Puu Nianiau, 6000 ft.” (1820 m; most probably the head of Waikamoi Gulch), and two specimens from Leleiwi Overlook, 2650 m elevation (P.D. Krushelnycky, CUIC, UHIM). The latter two specimens were collected in a pitfall trap in subalpine shrubland, though the summit record suggests also historical occupation of the alpine zone by this species.

#### (059) Mecyclothorax krushelnyckyi sp. n.

Taxon classificationAnimaliaColeopteraCarabidae

http://zoobank.org/B39CA7E3-9DA2-48FF-9254-C1D2729AE40B

Figs 78B , 79

##### Diagnosis.

This species (Fig. 78B) can be diagnosed from the other three small-bodied *Mecyclothorax ovipennis* group species with setal formula 2 2 2 2—*Mecyclothorax nubicola* (Fig. 78A), *Mecyclothorax pusillus* (Fig. 78C), and *Mecyclothorax rusticus* (Fig. 78D)–by: 1, the more narrowly constricted pronotal base; MPW/BPW = 1.54; 2, subellipsoid elytra, with the margin broadly rounded posterad the humerus, and the lateral margins evenly convex laterally; 3, elytra broad relative to forebody, with MEW/MPW = 1.51 and MEW/MHW = 2.15; and 4, the glossy upper body surface with little-developed microsculpture. The vertex is glossy, with obsolete transverse sculpticells, breadth 2× length in depressions. The pronotal disc is glossy, with obsolete transverse microsculpture visible in angled light; the pronotal base with obsolete transverse microsculpture over portions of the cuticle. The elytral disc is also glossy, with a transverse mesh, sculpticell breadth 2–3× length, restricted to the lateral and apical areas. Standardized body length 3.95 mm.

##### Description

(n = 1). *Head capsule* with frontal grooves broad near clypeus, lateral carina to anterior supraorbital seta; dorsal surface of neck slightly concave; eyes moderately developed, ocular ratio = 1.46, ocular lobe ratio = 0.78; labral anterior margin very shallowly emarginate apically; antennae filiform, antennomeres 2–3 with only 1–2 setae each along shafts; mentum tooth with sides acute, apex rounded. *Pronotum* transverse, MPW/PL = 1.28; hind angle sharply obtuse, lateral margin subparallel for short distance anterad angle; median base slightly depressed, ~10 punctures each side, punctures more elongate at juncture with disc; basal margin convexly expanded between laterobasal depressions; median longitudinal impression shallow, finely incised; anterior transverse impression deep, finely incised, area behind impression depressed relative to disc; anterior callosity convexly upraised, glossy; front angles slightly projected, rounded; pronotal apical width slightly greater than basal width, APW/BPW = 1.04; lateral marginal depression very narrow, edge appearing beaded except at front angle where margin is slightly broader; laterobasal depression narrow, continuous with lateral depression. *Proepisternum* with 5 minute punctures along hind marginal groove; prosternal process with narrow median impression, lateral margins broadly beaded between coxae. *Elytra* with disc flat, sides distinctly sloped; basal groove distinctly recurved to tightly rounded humeri, base relatively broad, MEW/HuW = 1.98; parascutellar seta absent; parascutellar striole with 4 punctures, striole shallow between punctures; sutural interval coplanar with lateral intervals basally, upraised apically; sutural and 2^nd^ striae of subequal depth from base to apex; discal striae 1–5 minutely punctate, the punctures joined by depressed stria medially, striae 4–5 composed of isolated punctures, inner intervals slightly convex, lateral intervals flat; 8^th^ interval slightly more convex than fused apical portion of striae 5 + 7; 2 dorsal elytral setae at 0.24–0.30× and 0.57× elytral length, setal impressions moderate, spanning 2/3 of interval 3; apical and subapical setae present; lateral elytral setae arranged in anterior series of 7 setae and posterior series of 6 setae; elytral marginal depression narrow from humerus to midlength, gradually reduced to beadlike margin at subapical sinuation; subapical sinuation shallow, more abruptly incurved anteriorly. *Mesepisternum* smooth; metepisternal width to length ratio 0.70; metepisternum/metepimeron suture distinct. *Abdomen* with irregular lateral wrinkles on ventrites 1–5; suture between ventrites 2 and 3 complete; apical female ventrite with 4 equally spaced setae plus a median trapezoid of 4 subequal, short setae. *Legs*-metatarsomere 1/metatibial length ratio = 0.19; metatarsomere 4 length along outer lobe 1.33× medial tarsomere length, apical and subapical setae present; metatarsal dorsolateral sulci shallow, narrow, median area broad. *Microsculpture* of metasternum a shallow transverse mesh; laterobasal abdominal ventrites with swirling isodiametric and transverse microsculpture. *Coloration* of vertex rufobrunneous with piceous cast; antennomeres 1–3 flavous, 4–11 darker, more brunneous; pronotal disc rufopiceous; pronotal lateral margins very narrowly and base and apex rufoflavous; proepipleuron flavous, proepisternum rufopiceous; elytral disc rufopiceous, sutural interval rufous in basal half, rufoflavous in apical half, lateral marginal depression and apex of interval 9 rufoflavous; elytral epipleuron dorsally flavous, ventrally rufoflavous, metepisternum rufopiceous; abdomen with ventral ventrites 1–5 and base of 6 rufopiceous, apical 1/3 of ventrite 6 flavous; metafemur flavous; metatibia flavous, only tibial spines brunneous.

**Female reproductive tract.** The lone female holotype was not dissected.

##### Holotype.

Female (UHIM) labeled: HI:Maui I. Haleakala / Kahikinui F.R. 2408 m el. / 20°41.93'N, W156°12.40', W / 24-XI-2008 P. Krushelnycky / Berlese shrubland litter // coll PDJ 627 / spec/lot# PKSP1463 // Mecyclothorax apicalis ? (PDK handwriting) // HOLOTYPE / Mecyclothorax / krushelnyckyi / Liebherr / det. J.K. Liebherr 2015 (black-margined red label).

##### Etymology.

This species is named to honor Dr. Paul Krushelnycky’s numerous, important discoveries of *Mecyclothorax* and *Blackburnia* species in the high elevation habitats of Haleakalā.

##### Distribution and habitat.

*Mecyclothorax krushelnyckyi* is one of the few species known to occupy Haleakalā’s south slope (Fig. 79); the others known to do so being *Mecyclothorax giffardi* (Fig. 56), *Mecyclothorax cordithorax* (Fig. 89), and *Mecyclothorax iteratus* (Fig. 106). The single specimen was collected in a sift sample of shrubland litter at 2408 m elevation.

#### (060) Mecyclothorax pusillus

Taxon classificationAnimaliaColeopteraCarabidae

Sharp

**Figure 80.**
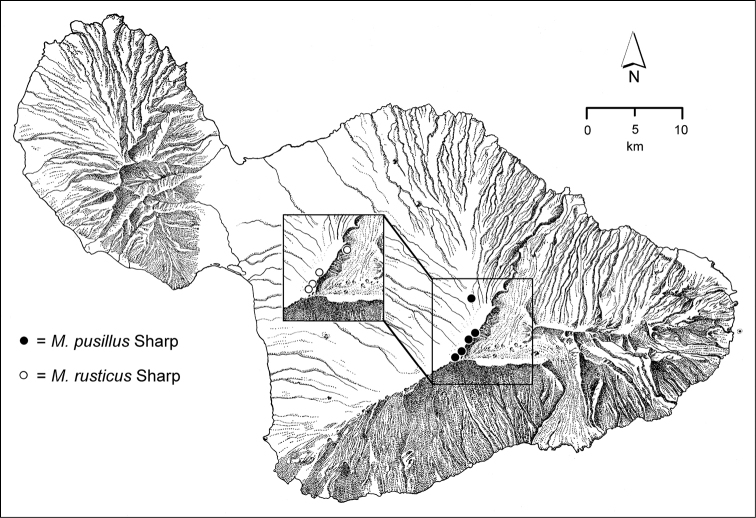
Recorded geographic distributions of *Mecyclothorax ovipennis* group species.

Figs 62L , 74E , 76G–H , 78C , 80

Mecyclothorax pusillus Sharp 1903: 243; Britton 1948b: 147.

##### Diagnosis.

Of the four *Mecyclothorax ovipennis* group species from Haleakalā with setal formula 2 2 2 2—*Mecyclothorax nubicola* (Fig. 78A), *Mecyclothorax krushelnyckyi* (Fig. 78B), *Mecyclothorax rusticus* (Fig. 78D), and this species (Fig. 78C)–*Mecyclothorax pusillus* can be diagnosed by : 1, pronotal base moderately broad, MPW/BPW = 1.39–1.48; 2, pronotal hind angles projected, obtuse, the lateral margin subparallel for twice the distance of the basal articulatory socket; 3, elytra narrow relative to head, MEW/MHW = 1.85–1.93, with sides subparallel, MEW/HUW = 1.84–1.90, the humerus tightly rounded; 4, frons and vertex with evident shallow transverse-mesh microsculpture, sculpticell breadth 2–3× length. Standardized body length 3.3–3.9 mm.

##### Identification

(n = 5). The head is broad with large eyes that cover much of the ocular lobe, ocular lobe ratio = 0.84–0.88, though the broad frons results in an ocular ratio lower than might be expected based on the eye size; ocular ratio = 1.45–1.48. Antennomeres 5–11 are stout, relatively short, of moniliform conformation similar to observed in *Mecyclothorax nubicola*. The pronotum is slightly transverse, MPW/PL = 1.19–1.29, with the base moderately constricted; MPW/BPW = 1.43–1.51. The pronotal median base is glossy due to the lack of microsculpture, but irregularly punctate with ~20 punctures each side, the punctures more elongate at juncture with disc. Elytral intervals 2–4 are nearly flat on the disc, though interval 2 is convex to the elytral apex, the sutural and 2^nd^ striae of subequal depth. The discal striae 2–4 are discontinuous, with their punctures isolated for portions of the strial length. The pronotal and elytral microsculpture are extremely similar to that observed in *Mecyclothorax rusticus*: 1, pronotal disc with obsolete transverse mesh, glossy medially, with sculpticell breadth 2–3× length laterally; 2, elytral disc with shallow isodiametric and transverse sculpticells in transverse rows, the elytral apex with an isodiametric mesh.

**Male genitalia** (n = 1). Aedeagal median lobe slender, distance between parameral articulation and tip 5.5× depth at midlength (Fig. 76G), lobe angled basally with median shaft straight; apex distinctly downturned, tip rounded; median lobe distinctly curved rightward toward apex in ventral view (Fig. 76H), right and left margins slightly convergent to blunt tip in this view; internal sac with fine spicules only, flagellar plate (visible in ventral view, Fig. 76H) short, length 0.34× parameral articulation-tip distance.

**Female reproductive tract** (n = 1). Bursa copulatrix columnar with rounded apex, length 0.46 mm, breadth 0.17 mm (Fig. 62L); bursal walls translucent with thin wrinkles, apex thinner, more diaphanous; gonocoxite 1 with 3 apical fringe setae, the middle seta larger, and 3–4 smaller setae along medial surface (Fig. 74E); gonocoxite 2 falcate with tightly rounded apex, base broadly extended laterally, 2 lateral ensiform setae with apical seta broader, apical nematiform setae on medial surface at 0.69× gonocoxite length.

##### Lectotype.

Male (BMNH) hereby designated, labeled: Thriscothorax pusillus Type D.S. Haleakala Perkins 254 // Type // Hawaiian Is. Perkins 1904-336. //LECTOTYPE Thriscothorax pusillus Sharp J.K. Liebherr 1998 (black-margined red label).

##### Distribution and habitat.

*Mecyclothorax pusillus* is the second of Perkin’s 19^th^ Century high-elevation *Mecyclothorax* triplet, with all of his records from his collecting activities made on his trips to the summit; 1830–3050 m elevation (Fig. 80). A single recent record (Kalahaku Overlook, 2870 m elevation, P.D. Krushelnycky, BPBM) places a lower-elevation population of this species in sympatry with Argentine Ant (Liebherr and Krushelnycky 2007).

#### (061) Mecyclothorax rusticus

Taxon classificationAnimaliaColeopteraCarabidae

Sharp

Figs 74F , 76I–J , 78D , 80

Mecyclothorax rusticus Sharp 1903: 244; Britton 1948b: 151.

##### Diagnosis.

Individuals of this species (Fig. 78D) are most like those of *Mecyclothorax pusillus* (Fig. 78C), and they are best diagnosed by the broader, more convexly margined elytra. The basal groove is recurved evenly on the rounded humeri, not tightly rounded to subangulate as in *Mecyclothorax pusillus*. Also, the head is narrow relative to the elytra; MEW/MHW = 2.02–2.07, with slightly more elongate antennomeres. The discal elytral striae are deeper and more continuous than in *Mecyclothorax pusillus*, though the lateral striae are more similar between the species; striae 5–6 discontinuous, stria 7 a series of minute punctures. The pronotal median base is little depressed relative to the disc, and is covered with ~10 isolated, elongate punctures each side, the surface glossy between the punctures. Finally, the vertex is glossy, with only an obsolete transverse mesh visible in angled light, sculpticell breadth 2–3× length. Setal formula 2 2 2 2. Standardized body length 3.7–4.3 mm.

##### Identification

(n = 5). The eyes are less convex than in individuals of *Mecyclothorax pusillus*, but due to the narrower head, the ocular ratio = 1.50–1.53; slightly greater than measured for that species. The pronotum is moderately constricted basally; MPW/BPW = 1.42–1.49. However the base is relatively broad, APW/BPW = 0.91–0.97, versus the subequal apical and basal pronotal widths in *Mecyclothorax pusillus*. The pronotal disc is glossy, with an obsolete transverse mesh, sculpticell breadth 2–3× length. The pronotal median base also glossy with shallow isodiametric sculpticells laterally between the punctures. The elytral disc bears shallow isodiametric and transverse sculpticells, breadth 2× length, in transverse rows, whereas the elytral apex is covered with shallow isodiametric sculpticells in transverse rows.

**Male genitalia** (n = 1). Aedeagal median lobe slender, distance between parameral articulation and tip 5.2× depth at midlength (Fig. 76I); apex nearly as broad as median shaft until it curves ventrally to narrow, blunt tip; median lobe sinuously curved apically in ventral view, right margin distinctly and briefly concave, left margin with incurved hitch near bluntly rounded tip (Fig. 76J); internal sac without ornamentation, flagellar plate short (visible in ventral view, Fig. 76J), length 0.29× parameral articulation-tip distance.

**Female reproductive tract** (n = 1). Bursa copulatrix columnar, length 0.46 mm, breadth 0.22 mm basally at vagina, 0.11 mm near rounded apex (a slightly broader version of *Mecyclothorax pusillus* bursa, Fig. 62L); bursal wall translucent with thin wrinkles; gonocoxite 1 with 2 short apical fringe setae and 4–6 shorter setae on medial surface (Fig. 74F); gonocoxite 2 falcate with broadly rounded apex (worn?), 2 short, broad lateral ensiform setae (also worn?), apical nematiform setae on medial surface at 0.73× gonocoxite length.

##### Lectotype.

Male (BMNH) hereby designated, labeled: Mecyclothorax rusticus Type D.S. Haleakala Perkins 254 // Type // Hawaiian Is. Perkins 1904-336. // Haleakala Maui 9½–10000 ft. Perkins IV-1894 // LECTOTYPE Mecyclothorax rusticus Sharp J.K. Liebherr 1998 (black-margined red label).

##### Distribution and habitat.

*Mecyclothorax rusticus* has been collected only by R.C.L. Perkins, with localities ranging 2895–3050 m elevation along his trips to the summit, and also at Holua, 2100–2200 m elevation at the western margin of Haleakalā Crater (Fig. 80).

### *Mecyclothorax argutor* species group

**Figure 81.**
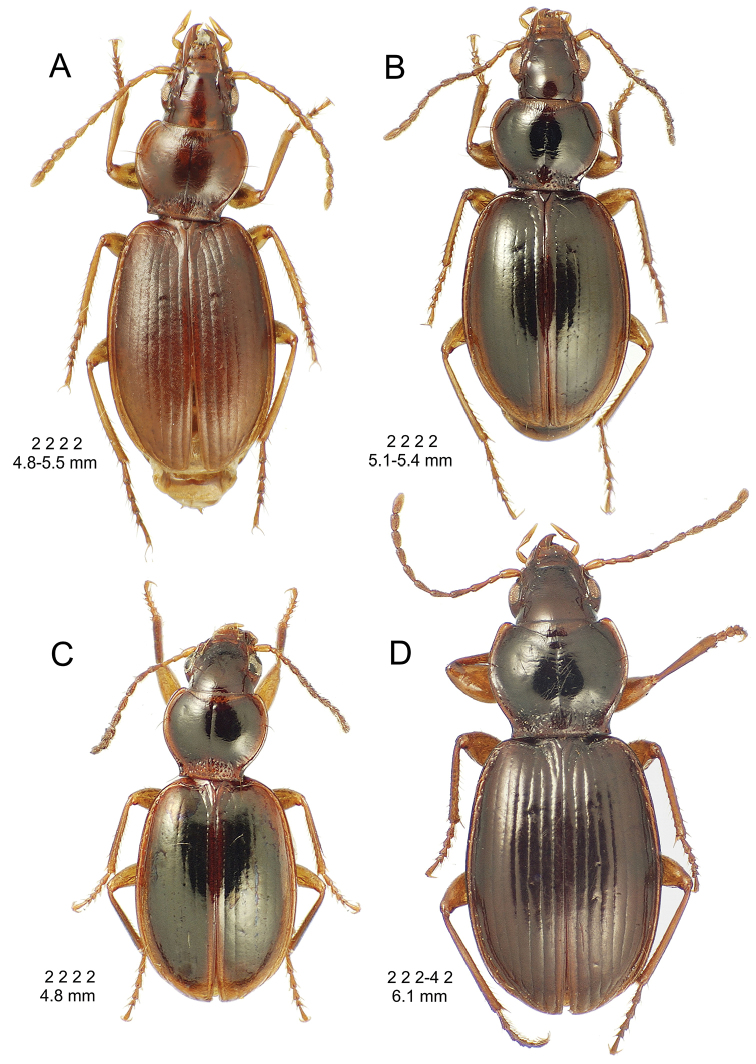
*Mecyclothorax argutor* group species, dorsal habitus view. **A** *Mecyclothorax ommatoplax* (Kīpahulu, 1500 m) **B** *Mecyclothorax semistriatus* (Honomanu, 1950 m) **C** *Mecyclothorax refulgens* (Kīpahulu, 1860 m) **D** *Mecyclothorax argutulus* (Kuhiwa, 1615 m).

**Diagnosis.** These are large-bodied species, nearly all individuals with standardized body length > 4.75 mm (only very few individuals of the very abundant and geographically widespread *Mecyclothorax cordithorax* with body length 4.5–4.7 mm). All species are characterized by presence of all major setae; 1, both anterior and posterior supraorbital setae; 2, lateral and basal pronotal setae; 3, parascutellar seta; 4, both anterior and posterior dorsal elytral setae, and in two species extra setae in 3^rd^ interval; 5, both apical and subapical elytral setae; and 6, the full complement of lateral elytral setae, i.e. an anterior series of seven setae and a posterior series of six setae. Thus the base setal formula for species in the group is 2 2 2 2. The eyes may be very small, flat, and covering little of the ocular lobe (Figs 81A, D, 87C), or moderately developed (81B–C, 87A–B, 87D), although in the latter set of species there may be substantial infraspecific variation in eye development, with some of the individuals exhibiting smaller, flatter eyes.

**Membership and distribution.** This group exhibits the greatest diversity on Haleakalā with the eight species treated below. Moloka‘i supports six species (Liebherr 2007), and West Maui (Liebherr 2011) and the island of Hawai‘i (Liebherr 2008b) each host two species.

#### Key to adults of the *Mecyclothorax argutor* species group, Haleakalā volcano, Maui, Hawai‘i

**Table:** 

1	Eyes moderately small to large, outer surface convex or positioned on ocular lobe so that inner dorsal margins converge on frons (Figs 81B–D, 87), ocular ratio = 1.39–1.53; elytra with shallow to well-developed isodiametric to transverse microsculpture, surface reflective; dorsal body coloration medially dark brunneous to piceous	**2**
1’	Eyes small, flat, ocular ratio = 1.29–1.35 (Fig. 81A); elytra with granulate isodiametric microsculpture, surface appearing rough, matte; dorsal body coloration pale brunneous	(062) ***Mecyclothorax ommatoplax* sp. n.**
2(1)	Elytra paler marginally, intervals 8–9 flavous, contrasted with piceous intervals 2–7 assessed on disc (Fig. 81B–C); discal elytral intervals flat to slightly convex	**3**
2’	Elytra concolorous except for possibly paler lateral marginal depression (Figs 81D, 87); discal elytral intervals moderately convex, associated striae well defined, moderately to distinctly punctate	**4**
3(2)	Elytral disc with evident isodiametric sculpticells in transverse rows; pronotal lateral margins narrowly flavous, depressed median base concolorous with disc; standardized body length 5.1–5.4 mm	(063) ***Mecyclothorax semistriatus* sp. n.**
3’	Elytral disc with distinctly transverse-mesh microsculpture (some sculpticells may be of subequal breadth and length near midline, but all are broader laterally on intervals 3–5 where they may be 4× broad as long); pronotal lateral margins broadly flavous, depressed median base distinctly paler than disc; standardized body length 4.8 mm	(064) ***Mecyclothorax refulgens* sp. n.**
4(2)	Pronotal lateral margins subparallel to briefly convergent anterad atmost slightly projected hind angles, lateral marginal depression narrow at midlength, front angle moderately projected anteriorly (Fig. 87); eyes larger, dorsal inner margins not convergent on frons	**5**
4’	Pronotal lateral margins distinctly convergent anterad acute, projected hind angles, pronotal lateral margins broad, front angle explanately projected anteriorly (Fig. 81D); eyes small, dorsal inner margins convergent on frons	(065) ***Mecyclothorax argutulus* sp. n.**
5(4)	Sutural interval elevated into a callus near elytral midlength, intervals 2–4 distinctly, broadly depressed near anterior dorsal seta (Fig. 87A–B)	**6**
5’	Sutural interval of similar convexity to other intervals near elytral midlength, intervals 2-4 at same elevation as sutural interval near anterior dorsal seta, or discal surface slightly depressed in vicinity of anterior seta (Fig. 87C–D)	**7**
6(5)	Pronotal apex and base of subequal width (Fig. 87A), APW/BPW = 1.02–1.05; male aedeagal median lobe with pointed apex (Figs 82E, G–H)	(066) ***Mecyclothorax planipennis* sp. n.**
6’	Pronotal apex broader than base (Fig. 87B), APW/BPW = 1.06–1.15; male aedeagal median lobe with rounded apex (Fig. 82I, K–L)	(067) ***Mecyclothorax planatus* sp. n.**
7(5)	Body narrower, MPW/PL = 1.19–1.22 (Fig. 87C); pronotal anterior transverse impression deep, smooth, defining a smooth, broadly convex anterior callosity; male aedeagal median lobe with narrow, elongate apex with tightly rounded, downturned tip (Fig. 88A–B)	(068) ***Mecyclothorax argutuloides* sp. n.**
7’	Body broader, MPW/PL = 1.25–1.38 (Fig. 87D); pronotal anterior transverse impression shallow, broad, the impression and anterior callosity crossed by fine longitudinal wrinkles; male aedeagal median lobe with short, broad apex that terminates in a pointed or tightly rounded tip (Fig. 88C–I)	(069) ***Mecyclothorax cordithorax* Liebherr**

#### (062) Mecyclothorax ommatoplax sp. n.

Taxon classificationAnimaliaColeopteraCarabidae

http://zoobank.org/E16F0AD3-9ACF-472E-9D4F-BB4A869C0601

Figs 81A , 85

##### Diagnosis.

This species is easily distinguished by the very small, little convex eyes; ocular ratio = 1.29–1.35, ocular lobe ratio = 0.68–0.69, in combination with the granulate isodiametric elytral microsculpture (Fig. 81A). Setal formula 2 2 2 2. Among species of the *Mecyclothorax argutor* group, the beetles are of moderate size, standardized body length 4.8–5.5 mm.

##### Description

(n = 2). *Head capsule* with frontal grooves broad near clypeus, curved laterally near clypeal juncture, a straight ridgelike lateral carina extended from dorsal mandibular articulation to mesad anterior supraorbital seta; dorsal surface of neck flat to slightly convex; compound eye with approximately 12–13 ommatidia across the maximal horizontal diameter; labral anterior margin medially emarginate 0.2× length; antennae filiform, antennomere 3 with sparse, short setae; mentum tooth with sides acute, apex pointed. *Pronotum* slightly transverse, MPW/PL = 1.17–1.27, constricted basally, MPW/BPW = 1.51–1.58; hind angle right, apex rounded, lateral margin parallel anterad angle; median base depressed relative to disc, covered with rugose wrinkles and round punctures; basal margin broadly, medially extended between laterobasal depressions; median longitudinal impression fine, shallow, crossed by shallow wrinkles; anterior transverse impression broad, shallow, crossed by longitudinal wrinkles; anterior callosity slightly convex, crossed by longitudinal wrinkles from anterior impression; front angles not projected, rounded; pronotal anterior width slightly greater than basal width, APW/BPW = 1.02–1.05; lateral marginal depression narrow, margin upturned to beaded; laterobasal depression moderately broad with low oblique ridge terminated at hind angle. *Proepisternum* with 6 punctures along hind marginal groove; prosternal process medially concave, margin smoothly upraised. *Elytra* subquadrate, narrow basally, the basal groove meeting lateral marginal depression at subangulate humerus, MEW/HuW = 1.81–1.94; disc flat, sides moderately depressed; parascutellar seta present; parascutellar striole shallow, smooth, continued onto base; sutural interval slightly more convex than lateral intervals; sutural and 2^nd^ striae of subequal depth from base to apex; discal striae 1–5 continuous, slightly irregular but impunctate, deeper and smooth apically, stria 6 reduced, stria 7 traceable, very shallow, associated intervals broadly, slightly convex; 8^th^ interval slightly convex laterad 7^th^ stria, principally due to deep apical portion of 7^th^ stria; 2 dorsal elytral setae at 0.30× and 0.59–0.60× elytral length, setal impressions shallow, about breadth of interval 3; apical and subapical setae present; lateral elytral setae arranged as anterior series of 7 setae and posterior series of 6 setae; elytral marginal depression moderately broad, lined with sculpticells, the margin upturned; subapical sinuation evident, abrupt and brief. *Mesepisternum* with ~14 punctures in 2–3 rows, metepisternal width to length ratio = 0.67; metepisternum/metepimeron suture distinct. *Abdomen* with shallow indistinct wrinkles on ventrites 1–3, suture between ventrites 2 and 3 complete; apical female ventrite with 4 equally spaced setae and median trapezoid of 4(–5 unilaterally) subequal, short setae. *Legs*-metatarsomere 1/metatibial length ratio = 0.19; metatarsomere 4 length along outer lobe 1.4× medial tarsomere length, with apical and subapical setae; metatarsal dorsolateral sulci broad, shallow. *Microsculpture* of vertex an upraised isodiametric mesh in transverse rows; pronotal disc with transverse mesh, median base with granulate isodiametric mesh; elytral apex with distinct transverse mesh; metasternum with distinct transverse mesh; laterobasal abdominal ventrites with swirling isodiametric and transverse microsculpture. *Coloration* of vertex rufobrunneous; antennomere 1 flavous, 2–11 rufoflavous; pronotal disc rufobrunneous, lateral margins narrowly paler in lateral depression, rufoflavous; proepipleuron rufoflavous, proepisternum rufobrunneous with piceous cast; elytral disc reflective rufobrunneous, sutural interval basally rufous, apically flavous, elytral margins broadly rufoflavous, apex broadly rufoflavous to flavous; elytral epipleuron flavous, metepisternum rufobrunneous with piceous cast; abdomen rufobrunneous with piceous cast, apical half of ventrite 6 broadly paler, flavous; metafemur flavous; metatibia rufoflavous.

**Female reproductive tract.** The holotype and paratype females were not dissected.

##### Holotype.

Female (CUIC) labeled: HI:Maui Haleakala N.P. / Kipahulu Vy.1500 m el. / 9-V-1991 sifting / leaf litter by day // S. Jessel / A.C. Medeiros, / Jr. collectors // Mecyclothorax / ommatoplax / ♀ photo / det. J.K. Liebherr 2014 // HOLOTYPE / Mecyclothorax / ommatoplax / Liebherr / det. J.K. Liebherr 2015 (black-margined red label).

##### Paratype.

HI: Maui, Kipahulu Vy., West Camp at Dead Pig Bog, 2010 m el., 18-iii-1998, Takumi (BPBM, 1).

##### Etymology.

The species epithet compounds the Greek words plax, flat round plate, and ommatos, the genitive of omma, i.e. eye. As Greek, ommatoplax is treated as a noun. The epithet refers to the very flat eyes characteristic of this species.

##### Distribution and habitat.

*Mecyclothorax ommatoplax* is distributed in the upper reaches of Kīpahulu Valley (Fig. 85). Specimens have been collected in sift samples of leaf and humus litter.

#### (063) Mecyclothorax semistriatus sp. n.

Taxon classificationAnimaliaColeopteraCarabidae

http://zoobank.org/47580371-0677-4518-BAF3-D6036D49425B

**Figure 82.**
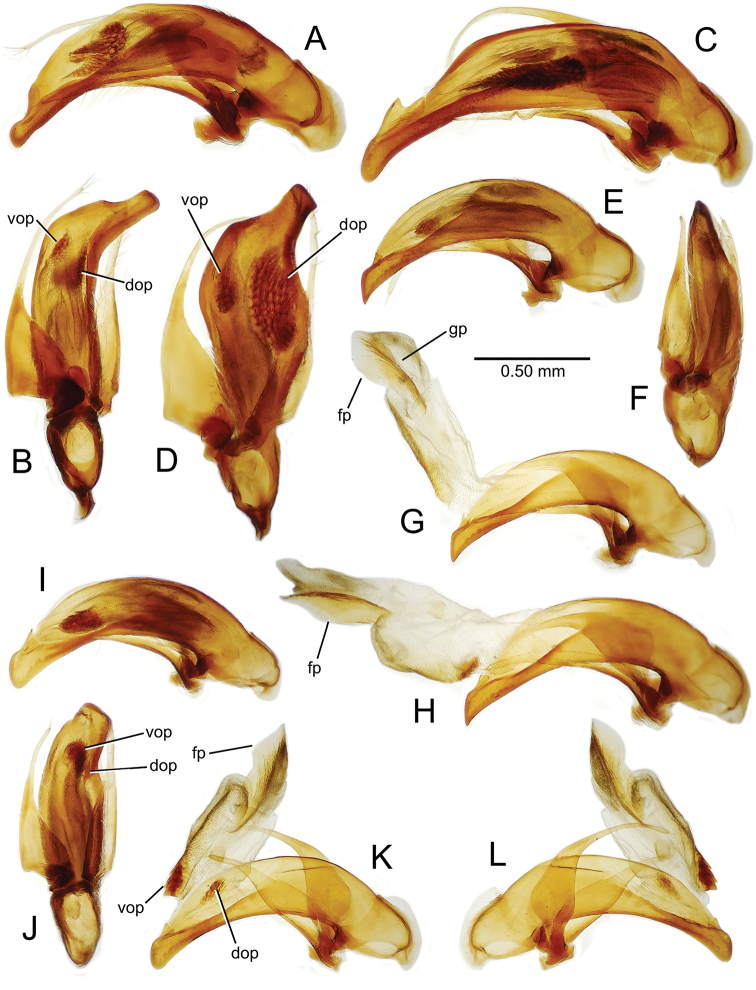
Male aedeagus, *Mecyclothorax argutor* group species (for abbreviations see Table 2, p. 23). **A–B** *Mecyclothorax semistriatus*, right and ventral views (Honomanu, 1950 m) **C–D** *Mecyclothorax argutulus*, right and ventral views (Kuhiwa, 1615 m) **E–H** *Mecyclothorax planipennis*. **E–F** Right and ventral views (Kīpahulu, 1960 m). **G–H** Right view, sac everted. **G** (ESE Kuiki, 2145 m) **H** (Kīpahulu, 1960 M) **I–L** *Mecyclothorax planatus* **I–J** Right and ventral views (Honomanu, 1820 m) **K–L** Right and left views, sac everted (Waikamoi, 1470 m).

**Figure 83.**
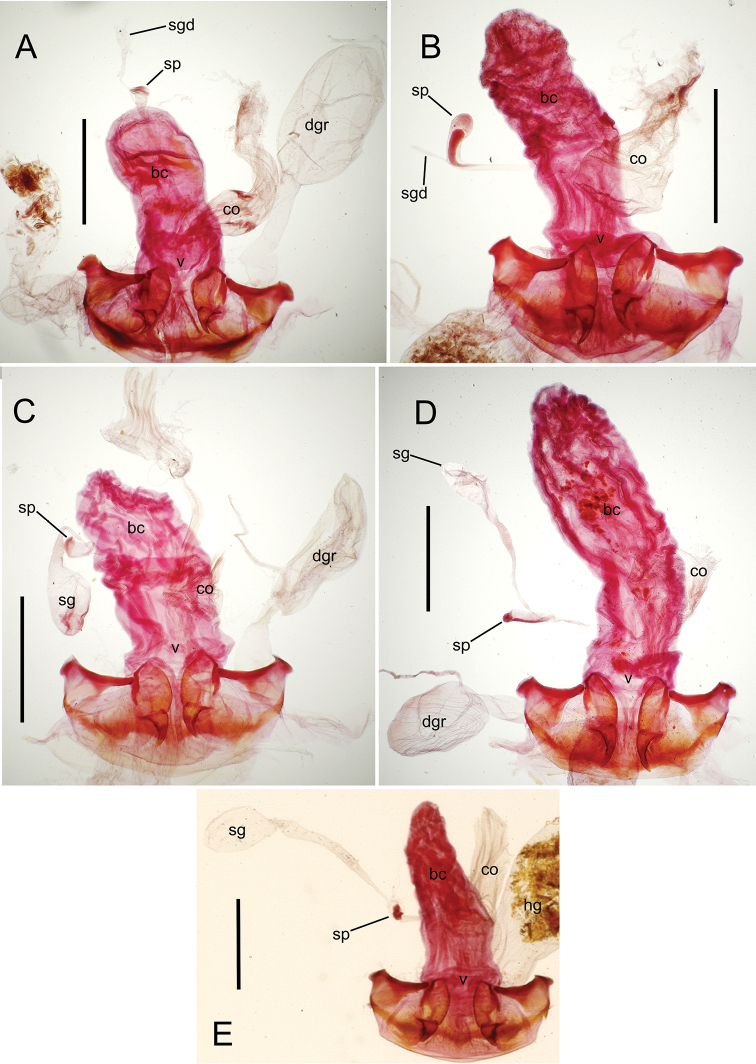
Female bursa copulatrix and associated reproductive structures, *Mecyclothorax argutor* group species, ventral view (for abbreviations see Table 2, p. 23). **A** *Mecyclothorax semistriatus* (Honomanu, 1950 m) **B** *Mecyclothorax planipennis* (ESE Kuiki, 2145 m) **C** *Mecyclothorax planatus* (Honomanu, 1700 m) **D** *Mecyclothorax argutuloides* (Kīpahulu, 2100 m) **E** *Mecyclothorax cordithorax* (Kaupō Gap, 1736 m). Scale bar = 0.50 mm.

**Figure 84.**
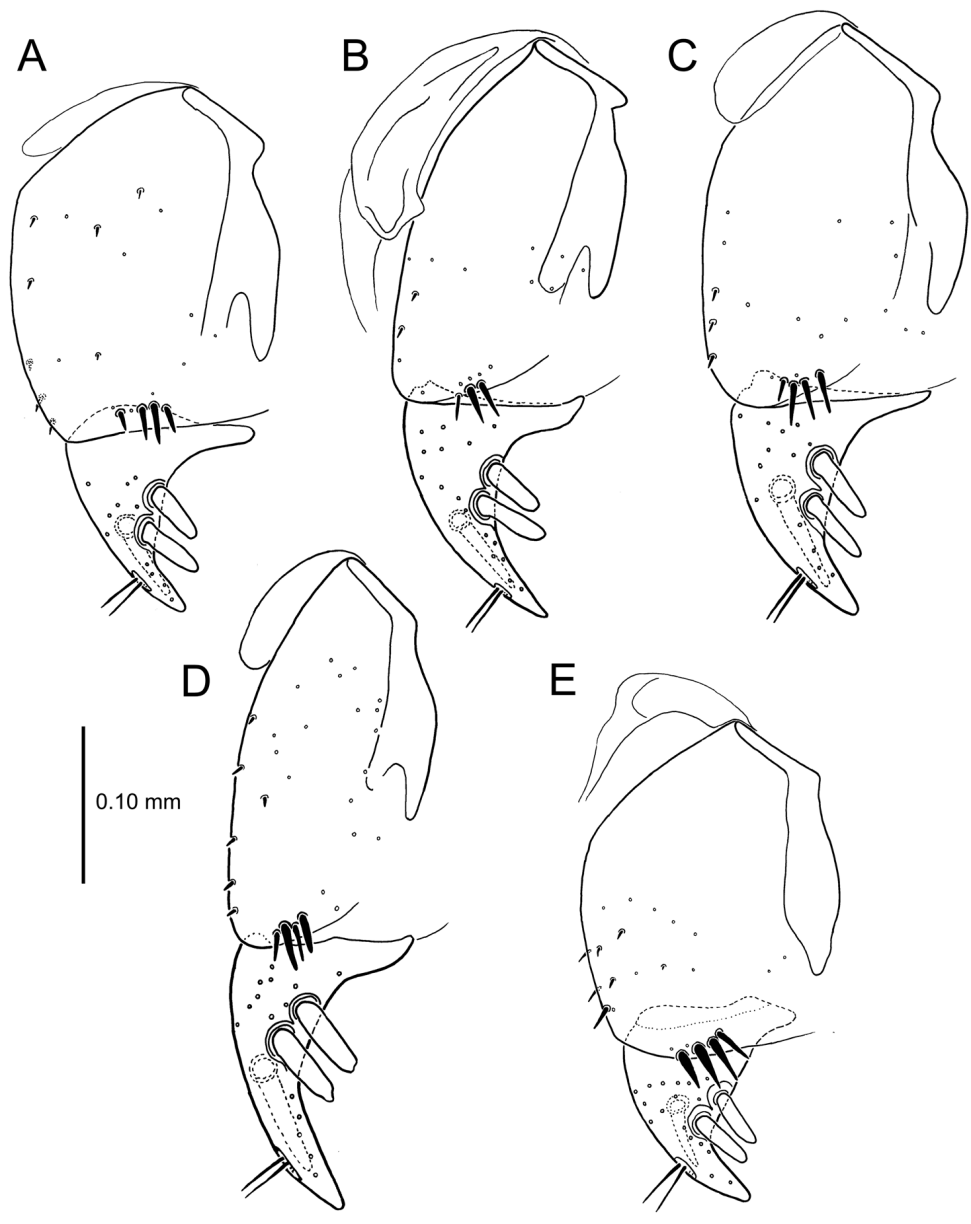
Left female gonocoxa, *Mecyclothorax argutor* group species, ventral view. **A** *Mecyclothorax semistriatus* (Honomanu, 1950 m) **B** *Mecyclothorax planipennis* (ESE Kuiki, 2145 m) **C** *Mecyclothorax planatus* (Honomanu, 1700 m) **D** *Mecyclothorax argutuloides* (Kīpahulu, 2100 m) **E** *Mecyclothorax cordithorax* (Kaupō Gap, 1736 m).

**Figure 85.**
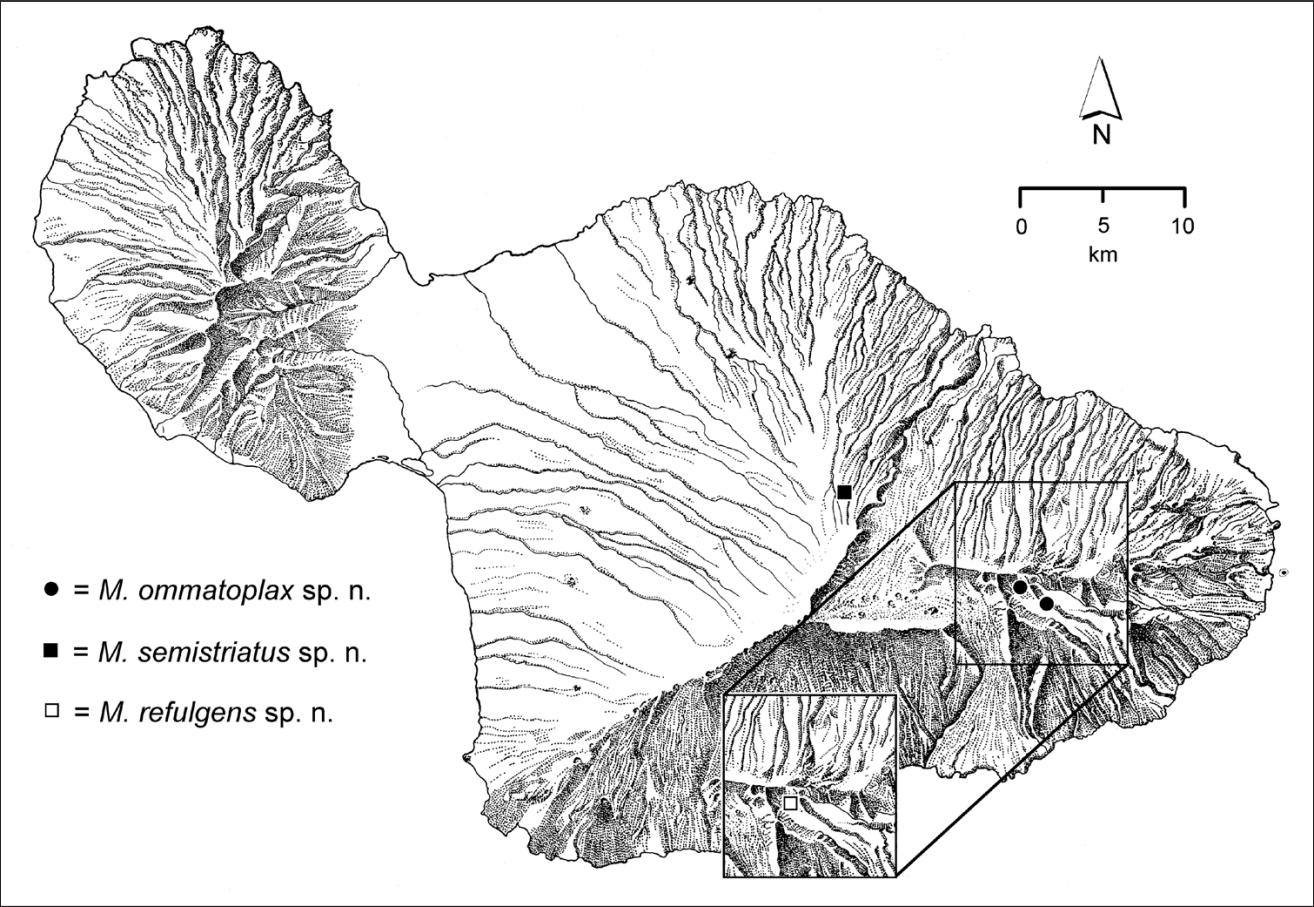
Recorded geographic distributions of *Mecyclothorax argutor* group species.

Figs 81B , 82A–B , 83A , 84A , 85

##### Diagnosis.

This (Fig. 81B) and the following *Mecyclothorax refulgens* (Fig. 81C) synapomorphously exhibit pale, flavous elytral margins that contrast with the piceous elytral disc. Beetles of this species have broader bodies, with the pronotum more transverse, MPW/PL = 1.21–1.32, and the elytra more broadly ellipsoid, the greatest width just behind midlength. Cuticular microsculpture is less developed in this species, the vertex covered with shallow isodiametric sculpticells in transverse rows, the elytral disc covered with a shallow, elongate transverse mesh, sculpticell breadth 2–3× length. Also, beetles of this species are larger than those of *Mecyclothorax refulgens*; standardized body length 5.1–5.4 mm. Setal formula 2 2 2 2.

##### Description

(n = 5). *Head capsule* with frontal grooves broad and moderately deep near clypeus, bordered laterally by broad carina to supraorbital seta; dorsal surface of neck flat to convex; eyes convex, of moderate size, ocular ratio = 1.39–1.47, ocular lobe ratio = 0.76–0.80; labral anterior margin broadly, shallowly emarginate; antennae filiform, antennomere 3 sparsely setose; mentum tooth with sides acute, apex tightly rounded. *Pronotum* cordate, lateral margins distinctly sinuate basally, hind angles acute, projected, MPW/BPW = 1.53–1.61; median base depressed relative to disc, with ~12 rounded punctures and sinuous depressions each side; basal margin slightly convex medially; median longitudinal impression fine, shallow, anterior transverse impression moderately deep, narrow, longitudinal wrinkles fore and aft; anterior callosity slightly convex, with indistinct longitudinal wrinkles; front angles slightly projected, tightly rounded; pronotal apex variably as broad as base, APW/BPW = 0.98–1.11; lateral marginal depression narrow throughout, margin upraised to beaded; laterobasal depression narrow, with low oblique ridge terminated at hind angle. *Proepisternum* with 6 punctures along hind marginal groove; prosternal process medially concave, margin smoothly upraised. *Elytra* moderately convex, disc narrowly flattened medially; basal groove recurved at tightly rounded humeral angle, MEW/HuW = 2.0–2.22; parascutellar seta present; parascutellar striole with 4 punctures, shallow between punctures; sutural interval of same convexity as intervals 2–3; sutural and 2^nd^ striae of subequal depth from base to apex; discal striae 1–3 shallow with small but evident punctures, lateral striae 4–6 shallower, traceable as isolated serial punctures, stria 7 obsolete except at elytral apex where it is broad and shallow; 8^th^ interval as convex as fused apical portion of striae 5 + 7; 2 dorsal elytral setae at 0.23–0.27× and 0.57–0.69× elytral width, setal impressions shallow, spanning ½ width of interval 3; apical and subapical setae present; lateral elytral setae arranged in anterior series of 7 setae and posterior series of 6 setae; elytral marginal depression moderately broad, lined with sculpticells, margin upturned; subapical sinuation shallow, nearly obsolete. *Mesepisternum* with ~9 punctures in 2–3 rows; metepisternal width to length ratio = 0.78; metepisternum/metepimeron suture distinct. *Abdomen* with shallow indistinct lateral wrinkles on ventrites 1–3; suture between ventrites 2 and 3 complete; apical male ventrite with 2 marginal setae and apical female ventrite with 4 equally spaced setae plus median trapezoid of 4 subequal, short setae. *Legs*-metatarsomere 1/metatibial length ratio = 0.18; metatarsomere 4 length along outer lobe 1.4× medial tarsomere length, apical and subapical setae present; metatarsal dorsolateral sulci broad, shallow. *Microsculpture* of pronotal median base of distinct isodiametric sculpticells in transverse rows; elytral apex with distinct transverse mesh; metasternum with shiny transverse mesh; laterobasal abdominal ventrites with swirling isodiametric and transverse microsculpture. *Coloration* of vertex rufobrunneous; antennomeres 1–4 flavous, 5–11 rufobrunneous; pronotal disc rufobrunneous, margins narrowly rufoflavous; proepipleuron rufoflavous, proepisternum rufobrunneous; elytral disc reflective rufopiceous, sutural interval rufous basally, flavous apically; elytral margins broadly paler, with intervals 7–9 or 8–9 rufoflavous near interval 6 to flavous laterally, apex broadly flavous apicad subapical sinuation; elytral epipleuron flavous with rufous cast, metepisternum rufobrunneous with piceous cast; abdomen with ventrites 1–2 piceous, 3–5 rufoflavous, 6 broadly flavous; metafemur flavous, metatibia rufoflavous.

**Male genitalia** (n = 1). Aedeagal median lobe robust, distance from parameral articulation to tip 3.2× depth at midlength (Fig. 82A); apex narrowed distad ostial opening, tip expanded dorsally, knoblike; median lobe distinctly curved rightward near apex in ventral view (Fig. 82B), left margin indented before parallel-sided, bluntly rounded tip; internal sac with small dorsal ostial microtrichial patch (left patch in Fig. 82B) and larger ventral ostial patch; flagellar plate of moderate size, length 0.38× parameral articulation-tip distance.

**Female reproductive tract** (n = 1). Bursa copulatrix columnar with rounded apex, length 0.82 mm, breadth 0.37 mm (Fig. 83A); bursal walls translucent, smooth with a few wrinkles; gonocoxite 1 with 4 apical fringe setae, the middle two setae larger, 1 small seta basad apicomedial angle, 5–7 smaller setae on medial surface (Fig. 84A); gonocoxite 2 broadly falcate, base broadly extended laterally, 2 stout, apically narrowed lateral ensiform setae, apical nematiform setae on medial surface at 0.80× gonocoxite length.

##### Holotype.

Male (CAS) labeled: U.S.A. Hawaii: Maui, / Haleakala nw. slope / Waikamoi Reserve / Transect 3, 1950 m, / 7 May 1991, Stop #91-10B / D.H. Kavanaugh collector // D.H. Kavanaugh / collection // 2 // HOLOTYPE / Mecyclothorax / semistriatus / Liebherr / det. J.K. Liebherr 2015 (black-margined red label).

##### Paratypes.

Same data as holotype (CAS, 2; CUIC, 2).

##### Etymology.

The adjectival species epithet semistriatus refers to the medially striate and laterally smooth elytra.

##### Distribution and habitat.

The type series of *Mecyclothorax semistriatus* was found in moss adhering to tall mastlike ‘ōhi‘a trees in the upper Honomanu drainage, 1950 m elevation (Fig. 85). The ‘Ōhi‘a Mesic Forest is quite open here, with the moss layers rather thin, though they were moist at the time of collecting.

#### (064) Mecyclothorax refulgens sp. n.

Taxon classificationAnimaliaColeopteraCarabidae

http://zoobank.org/958DC6DE-1BCE-4CEE-875C-8392177387DE

Figs 81C , 85

##### Diagnosis.

This and *Mecyclothorax semistriatus*, comprise the pair of *Mecyclothorax argutor* group species characterized by contrastingly pale elytral margins and shallow elytral striae. This species (Fig. 81C) can be told from *Mecyclothorax semistriatus* (Fig. 81B) by the broadly paler pronotal and elytral lateral margins, and the basally narrower elytra, the elytral shape distinctly ovoid with the greatest width well behind midlength. Also, beetles of this species exhibit more well-developed microsculpture, with the elytral disc subiridescent due to the well-developed transverse and isodiametric sculpticells. The discal elytral striae are less punctate in this species, with only the sutural stria punctate basally, striae 2–5 at most slightly irregular. The sutural and 2^nd^ striae are of subequal depth only at the extreme apex of the elytra. More basally the sutural stria is broader and deeper. Setal formula 2 2 2 2. Standardized body length 4.8 mm.

##### Description

(n = 1). *Head capsule* with frontal grooves broad near clypeus, a sinuous lateral carina extended to anterior supraorbital seta; dorsal surface of neck flat; eyes moderately convex, ocular lobe moderately protruded from gena, ocular ratio = 1.46, ocular lobe ratio = 0.79; labral anterior margin broadly, shallowly emarginate; antennae filiform, antennomere 3 sparsely setose; mentum tooth with sides acute, apex tightly rounded. *Pronotum* cordate, little transverse, MPW/PL = 1.19, lateral margins distinctly sinuate before very slightly acute, projected hind angles; pronotal base narrow, MPW/BPW = 1.63; median base depressed relative to disc, 12–14 punctures laterally plus wrinkles present along juncture with disc; basal margin straight; median longitudinal impression fine, very shallow; anterior transverse impression broad, shallow medially, crossed by longitudinal wrinkles, deep, continuous in lateral 2/3; anterior callosity slightly convex, longitudinal wrinkles indistinct; front angles slightly projected, tightly rounded; pronotal apical width greater than basal width, APW/BPW = 1.10; lateral marginal depression narrow throughout, margin upturned to beaded; laterobasal depression moderately broad, slightly raised medially. *Proepisternum* with 6 punctures along hind marginal groove; prosternal process medially concave, margin smoothly upraised. *Elytra* convex, sides depressed; basal groove meeting lateral marginal depression at subangulate humerus; elytra narrow basally, MEW/HuW = 2.10; parascutellar seta present; parascutellar striole shallow, smooth, with 1–2 punctures; sutural interval more convex than lateral intervals, the sutural juncture upraised; sutural stria deeper and more punctate than 2^nd^ stria on disc, discal striae 3–5 traceable, smooth, stria 6 obsolete, stria 7 absent, associated intervals broadly, slightly convex on disc, flat laterally; 8^th^ interval laterad position of 7^th^ stria not more convex than more medial elytral surface; 2 dorsal elytral setae at 0.23–0.29× and 0.66× elytral length, setal impressions shallow, broad, spanning interval 3; apical and subapical setae present; lateral elytral setae arranged in anterior series of 7 setae and posterior series of 6 setae; elytral marginal depression narrow, margin upturned; subapical sinuation shallow, broad. *Mesepisternum* with ~8 punctures in 2–3 rows; metepisternal width to length ratio = 0.79; metepisternum/metepimeron suture distinct. *Abdomen* with shallow, indistinct lateral wrinkles on ventrites 1–3; suture between ventrites 2 and 3 complete; apical female ventrite with 4 equally spaced setae and median pair of 2 small setae along ventrite margin. *Legs*-metatarsomere 1/metatibial length ratio = 0.18; metatarsomere 4 length along outer lobe 1.4× medial tarsomere length, apical and subapical setae present; metatarsal dorsolateral sulci narrow, canaliculated, median carina broad. *Microsculpture* of vertex isodiametric sculpticells in transverse rows; pronotal disc with transverse mesh and parallel lines, median base with distinct isodiametric sculpticells; elytral apex covered with reflective transverse mesh; metasternum with reflective transverse mesh; laterobasal abdominal ventrites with swirling isodiametric and transverse microsculpture. *Coloration* of vertex rufobrunneous with piceous cast; antennomeres 1–3 flavous, 4-11 rufobrunneous; pronotal disc rufopiceous, lateral margins and pronotal base broadly rufoflavous; proepipleuron rufoflavous, proepisternum rufobrunneous with piceous cast; elytral disc reflective rufopiceous, sutural interval rufous basally, flavous apically; elytral margins broadly paler, intervals 8–9 rufoflavous to flavous at marginal depression, elytral apex broadly rufoflavous to flavous apicad subapical sinuation; elytral epipleuron flavous, metepisternum rufobrunneous with piceous cast; abdomen with ventrites 1–2 and base of 3 piceous, apex of 3 rufoflavous, ventrites 4–6 flavous; metafemur flavous; metatibia rufoflavous.

**Female reproductive tract.** The lone female specimen was not dissected.

##### Holotype.

Female (BPBM) labeled: HAWAIIAN IS.: Maui I: / Kipahulu Valley: West Camp; / 12.vii.1983 // at night on tree trunks / F.G. Howarth, Col. / BISHOP Museum // HOLOTYPE / Mecyclothorax / refulgens / Liebherr / det. J.K. Liebherr 2015 (black-margined red label).

##### Etymology.

The dorsal body surface of these beetles is quite reflective, leading to use of the present participle of the Latin verb refulgeo—to shine—as the species epithet. The nominative singular participle refulgens maintains its ending regardless of gender.

##### Distribution and habitat.

*Mecyclothorax refulgens* is distributed in upper Kīpahulu Valley (Fig. 85), 1860 m elevation. The type specimen was collected from a tree trunk at night.

#### (065) Mecyclothorax argutulus sp. n.

Taxon classificationAnimaliaColeopteraCarabidae

http://zoobank.org/50B5E5DB-D050-4738-BABD-95B6B43857C8

**Figure 86.**
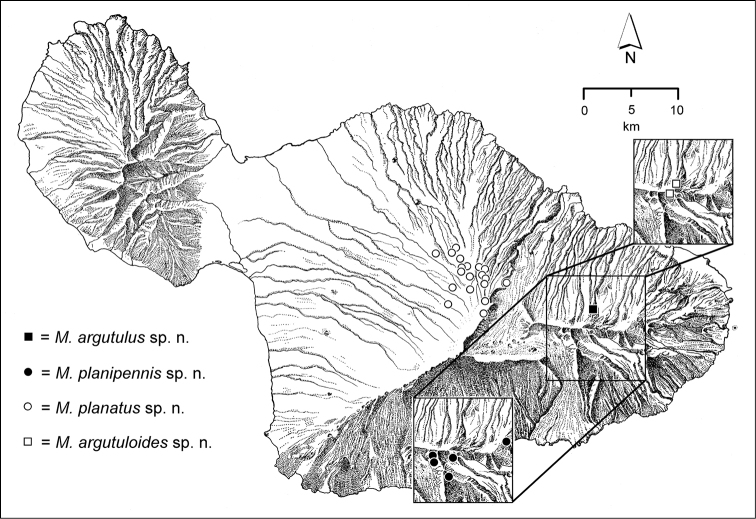
Recorded geographic distributions of *Mecyclothorax argutor* group species.

Figs 81D , 82C–D , 86

##### Diagnosis.

**Figure 87.**
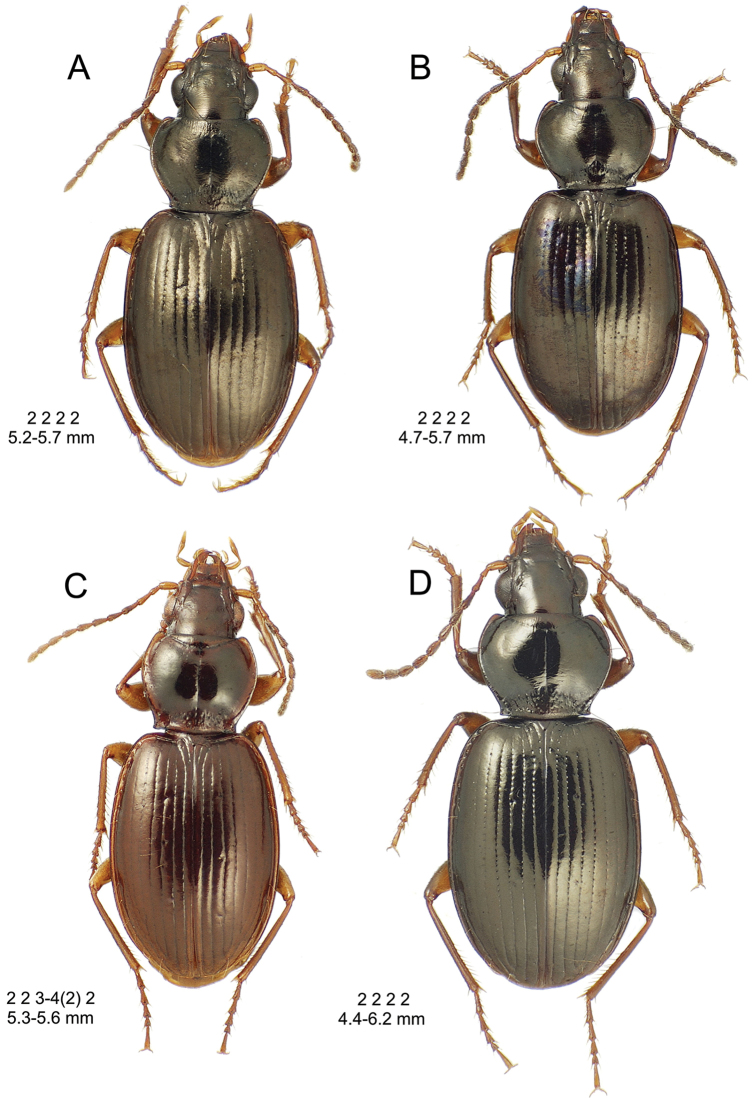
*Mecyclothorax argutor* group species, dorsal habitus view. **A** *Mecyclothorax planipennis* (ESE Kuiki, 2145 m) **B** *Mecyclothorax planatus* (Honomanu, 1700 m) **C** *Mecyclothorax argutuloides* (Kīpahulu, 2100 m) **D** *Mecyclothorax cordithorax* (Polipoli, 1890 m).

This species (Fig. 81D) and *Mecyclothorax argutuloides* (Fig. 87C) comprise a second sister-species pair within the Haleakalā *Mecyclothorax argutor* group. Both species can be told by the broad, robust pronotum that is little constricted basally, MPW/BPW = 1.38–1.40. Of the two, this species displays a more robust appearance, with the pronotal lateral margins broad, and elytra broadly expanded laterally with broad, translucent lateral marginal depressions. The eyes of *Mecyclothorax argutulus* beetles are also less convex, ocular ratio = 1.33, though covering about as much of the ocular lobe as observed in *Mecyclothorax argutuloides*; ocular lobe ratio for this species = 0.69, ratios of *Mecyclothorax argutuloides* individuals span 0.68–0.73. Setal formula 2 2 2-4 2; the single specimen has two dorsal elytral setae on the left elytron, four on the right. Standardized body length 6.1 mm.

##### Description

(n = 1). *Head capsule* with frontal grooves broad near clypeus, a broad, straight lateral carina extended to anterior supraorbital seta; dorsal surface of neck slightly concave; ocular lobe broadly expanded from gena, outer surface of eye not extended beyond curvature of lobe; horizontal diameter of eye crosses 18–19 ommatidia; labral anterior margin broadly, shallowly emarginate; antennae filiform, antennomere 3 sparsely setose; mentum tooth with sides acute, apex pointed. *Pronotum* slightly transverse, MPW/PL = 1.22, the lateral margins distinctly sinuate before the acute, very projected hind angles, the pronotal base broad so that MPW/BPW = 1.40; median base depressed relative to disc, rugose due to dense wrinkles and punctures; basal margin broadly extended medially between laterobasal depressions; median longitudinal impression fine, shallow, crossed by shallow transverse wrinkles; anterior transverse impression deeply incised, smooth, anterior callosity broadly convex, smooth; front angles well projected, rounded; pronotal apical width subequal to broad basal width, APW/BPW = 0.98; lateral marginal depression broad, margin broadly upraised; laterobasal depression broad, slightly upraised tubercle present. *Proepisternum* with 6 punctures along hind marginal groove; prosternal process medially concave, margin smoothly upraised. *Elytra* subquadrate, broadest behind middle, MEW/MPW = 1.38 elytral disc flat, sides moderately sloped; basal groove recurved at tightly rounded humerus, MEW/HuW = 1.83; parascutellar seta present; parascutellar striole deep, smooth, with 5 elongate punctures; sutural interval slightly convex, similar to lateral intervals; sutural and 2^nd^ striae of subequal depth from base to apex; discal striae 1–5 minutely punctate, continuous, stria 6 reduced, very shallow, stria 7 traceable only; 8^th^ interval laterad 7^th^ stria of similar convexity to more medial intervals; the 2 paired dorsal elytral setae at 0.24–0.27× and 0.54- 0.56× elytral length, extra dorsal setae on the right elytron at 0.45× and 0.82× elytral length, setal impressions shallow, spanning about ½ width of interval 3; apical and subapical setae present; lateral elytral setae arranged in anterior series of 7 setae and a posterior series of 6 setae; elytral marginal depression moderately broad, translucent and lined with sculpticells, margin upturned; subapical sinuation shallow, broad. *Mesepisternum* with ~6 shallow punctures on a glossy surface; metepisternal width to length ratio = 0.89; metepisternum/metepimeron suture a broad, incomplete depression; metathoracic flight wing a triangular flap, 1.7× long as broad, remnant R and M veins present, the flap extended to hind margin of metanotum. *Abdomen* with shallow indistinct lateral wrinkles on ventrites 1–3; suture between ventrites 2 and 3 complete; apical male ventrite with 2 marginal setae. *Legs*-metatarsomere 1/metatibial length ratio = 0.17; metatarsomere 4 length along outer lobe 1.25× medial tarsomere length, apical and subapical setae present; metatarsal dorsolateral sulci broad, shallow. *Microsculpture* of vertex shallow isodiametric sculpticells in transverse rows; pronotal disc with shallow transverse mesh, median base with distinct isodiametric sculpticells; elytral disc with an elongate transverse mesh, the sculpticells more distinct on the elytral apex; metasternum with distinct transverse mesh; laterobasal abdominal ventrites with swirling isodiametric and transverse microsculpture. *Coloration* of vertex brunneous with piceous cast; antennomeres 1–3 and base of 4 rufoflavous, apical portion with piceous cast; pronotal disc rufopiceous, lateral marginal depression broadly rufoflavous; proepipleuron rufoflavous with piceous cast, proepisternum rufopiceous; elytral disc reflective rufobrunneous with piceous cast, sutural interval concolorous basally, rufoflavous apically; elytral margins narrowly rufoflavous in lateral depression, apex slightly, broadly paler; elytral epipleuron rufoflavous, metepisternum rufopiceous; abdomen rufopiceous, slightly paler laterally, apical half of apical ventrite broadly paler, rufoflavous; metafemur and metatibia rufoflavous.

**Male genitalia** (n = 1). Aedeagal median lobe very robust, distance from parameral articulation to tip 3.6× depth at midlength (Fig. 82C); apex narrowly extended beyond ostial opening, tip angled downward, tightly rounded; median lobe curved rightward near apex in ventral view (Fig. 82D), median shaft very broad, expanded medially, maximum breadth 0.37× parameral articulation-tip distance; internal sac with very large dorsal ostial microtrichial patch (right patch in Fig. 82D), plus well-developed ventral ostial microtrichial patch; flagellar plate moderately short, length 0.33× parameral articulation-tip distance (estimated from shadow of sclerotized plate inside dorsal margin; Fig. 82C).

##### Holotype.

Male (CUIC) dissected and labeled: HI:Maui Haleakala / Hanawi N.A.R. Kuhiwa / Str. E Poouli Cabin 6-V- / 1998 lot 03 1615 m el. / under rocks along / stream C.P. Ewing // HOLOTYPE / Mecyclothorax / argutulus / Liebherr / det. J.K. Liebherr 2015 (black-margined red label).

##### Etymology.

The adjectival species epithet argutulus is the diminutive of argutus, clear, bright, or sharp (Brown 1956).

##### Distribution and habitat.

This species is known from Kuhiwa Stream, 1600 m elevation on the Hanawī windward face of Haleakalā (Fig. 86). The type specimen was collected from under a rock in the streambed; an unusual microhabitat for a *Mecyclothorax* beetle on Maui. However on the island of Moloka‘i where streams have smaller catchments and lower flow rates, several *Mecyclothorax* have been collected in streamside habitats, including *Mecyclothorax argutor* (Sharp), *Mecyclothorax palustris* (Sharp), and *Mecyclothorax cymindoides* Liebherr (2007).

#### (066) Mecyclothorax planipennis sp. n.

Taxon classificationAnimaliaColeopteraCarabidae

http://zoobank.org/2959BEC2-1EA6-43EF-9670-BF5D13E61EC4

Figs 82E–H , 83B , 84B , 86 , 87A

##### Diagnosis.

This species (Fig. 87A) and *Mecyclothorax planatus* (Fig. 87B) comprise the third sister-species pair within the Haleakalā *Mecyclothorax argutor* group fauna. Their adelphotaxon status is supported by the broad depressions of the elytra; an anterior depression centered each side on the anterior dorsal elytral seta, and a posterior depression implicating intervals 1–5 and lying posterolaterad of the posterior dorsal elytral seta. A second synapomorphy for the pair is the presence of four marginal setae on the male apical abdominal ventrite, a character not observed in any other Hawaiian *Mecyclothorax* species. *Mecyclothorax planipennis* can be differentiated from *Mecyclothorax planatus* by: 1, elytral intervals slightly convex near anterior dorsal elytral seta, not flat as in *Mecyclothorax planatus*; 2, pronotum tending to be relatively less constricted basally, MPW/BPW = 1.45–1.50 versus values of 1.49–1.60 in *Mecyclothorax planatus*, that basal constriction leading to a diagnostic difference in apical versus basal pronotal widths, APW/BPW = 1.02–1.05 for *Mecyclothorax planipennis* versus APW/BPW = 1.06–1.15 for *Mecyclothorax planatus*. Although these species are exceedingly similar in outward appearance, the male aedeagal median lobes differ distinctively. The median lobe tip of *Mecyclothorax planipennis* males is pointed (Fig. 82E–H), whereas the median lobe tip of *Mecyclothorax planatus* males is bluntly rounded (Fig. 82I–L). Setal formula 2 2 2 2. Standardized body length 5.2–5.7 mm.

##### Description

(n = 5). *Head capsule* with frontal grooves broad near clypeus, a broad lateral carina posterad to anterior supraorbital seta; dorsal surface of neck flat to convex; eyes moderately convex, ocular lobe obtusely extended from gena, ocular ratio = 1.41–1.51, ocular lobe ratio = 0.72–0.80; labral anterior margin broadly, shallowly emarginate; antennae filiform, antennomere 3 sparsely setose; mentum tooth with sides acute, apex tightly rounded. *Pronotum* transverse, MPW/PL = 1.30–1.39; hind angle right, margin behind rounded, lateral margin subparallel to slightly convergent for short distance anterad angle; median base depressed relative to disc, covered with rugose wrinkles and punctures; basal margin broadly convex medially; median longitudinal impression fine, shallow; anterior transverse impression broad, bordered anteriorly by depressed to slightly convex anterior callosity, the callosity crossed by numerous, densely packed longitudinal wrinkles; front angles slightly projected, rounded; lateral marginal depression moderately narrow, edge upturned; laterobasal depression broadly concave. *Proepisternum* with 6 punctures along hind marginal groove; prosternal process medially concave, margins broadly upraised. *Elytra* subquadrate, disc flattened due to anterior elytral depressions, sides moderately sloped; basal groove evenly curved to subangulate juncture with lateral marginal depression, the lateral marginal depression broader than basal groove, MEW/HuW = 1.83–1.92; parascutellar seta present; parascutellar striole shallow, with 6–7 punctures; sutural interval more convex than lateral intervals, sutural juncture upraised; sutural and 2^nd^ striae of subequal depth from base to apex; discal striae 1–4 punctate in basal 2/3 of length, stria 5 continuous, striae 6–7 obsolete laterally, striae 1–5 and 7 evident at elytral apex; 8^th^ interval convexly elevated immediately laterad 7^th^ stria; 2 dorsal elytral setae at 0.26–0.29× and 0.54–0.60× elytral length, setal impression spanning interval 3; apical and subapical setae present; lateral elytral setae arranged in anterior series of 7 setae and posterior series of 6 setae; elytral marginal depression moderately broad, lined with sculpticells, margin upturned; subapical sinuation shallow, broad. *Mesepisternum* with ~14 punctures in 2–3 rows; metepisternal width to length ratio = 0.75; metepisternum/metepimeron suture distinct. *Abdomen* with 1–2 longitudinal, lateral wrinkles on ventrites 1–4; suture between ventrites 2 and 3 complete; apical male ventrite with 4 equally spaced marginal setae (a 5^th^ unilateral seta in one individual), apical female ventrite with 4 equally space marginal setae and median trapezoid of 4 subequal, short setae. *Legs*-metatarsomere 1/metatibial length ratio = 0.19; metatarsomere 4 length along outer lobe 1.3× medial tarsomere length, apical and subapical setae present; metatarsal dorsolateral sulci narrow, canaliculated, median carina broad. *Microsculpture* of vertex upraised isodiametric sculpticells in transverse rows; pronotal disc with isodiametric sculpticells in transverse rows, median base with upraised isodiametric mesh; elytral disc with upraised isodiametric sculpticells in transverse rows, apex with upraised isodiametric sculpticells; metasternum with transverse mesh; laterobasal abdominal ventrites with swirling isodiametric and transverse microsculpture. *Coloration* of vertex brunneous with piceous cast; antennomeres 1–3 rufoflavous, 4–11 darker, with piceous cast; pronotal disc rufobrunneous with piceous cast, margins paler, rufoflavous in lateral depression; proepipleuron rufoflavous, proepisternum rufobrunneous with piceous cast; elytral disc reflective rufobrunneous, sutural interval rufous basally, rufoflavous apically; elytral margins narrowly rufoflavous in lateral depression, apex slightly, broadly paler; elytral epipleuron rufoflavous, metepisternum rufopiceous; abdomen uniformly dark, rufopiceous, apical abdominal ventrite very narrowly paler, rufoflavous; metafemur with basal half rufopiceous, apex flavous; metatibia rufobrunneous with piceous cast.

**Male genitalia** (n = 3). Aedeagal median lobe robust, distance from parameral articulation to tip 3.2× depth at midlength (Fig. 82E, G–H); apex briefly extended beyond ostial opening, apical face broadly flattened, tip acutely angulate, slightly downturned; median lobe straight in ventral view (Fig. 82F), right margin indented before rounded tip in this view, left margin slightly curved; internal sac with ventral surface covered with denser microspicules (Fig. 82G–H), a ventral ostial microtrichial patch either present (Fig. 82H) or absent (Fig. 82G) near sac base; flagellar plate large, length 0.56–0.61× parameral articulation-tip distance.

**Female reproductive tract** (n = 1). Bursa copulatrix columnar, elongate, length 0.97 mm, breadth 0.34 mm (Fig. 83B); bursal walls moderately opaque, thickly wrinkled; gonocoxite 1 with 2–3 apical fringe setae and 3–4 smaller setae on medial surface (Fig. 84B); gonocoxite 2 falcate, tip pointed, base broadly extended laterally, 2 stout, apically narrowed lateral ensiform setae, apical nematiform setae on medial surface at 0.73× gonocoxite length.

##### Holotype.

Female (BPBM) labeled: Paliku, Haleakala / Crater, Maui 6500', / near Kaupo Trail / VII-22-65 // J.W. Beardsley / Collector // HOLOTYPE / Mecyclothorax / planipennis / Liebherr / det. J.K. Liebherr 2015 (black-margined red label).

##### Paratypes.

HI: Maui: Haleakala N.P., Haleakala Crater, Paliku, banana bait trap, 1960 m el., 25-vii-1962, Hardy (BPBM, 1), diphacinone bait station, 1830– 1950 m el., 21-x-1997, Takumi (BPBM, 1), 8-i-1998, Takumi (BPBM, 1), 01-v-2008, Kaholoa‘a (BPBM, 1), Kipahulu Vy., West Camp, pyrethrin fog *Metrosideros*/moss, 1960 m el., 19-v-1998 lot 01, Polhemus (NMNH, 3), below Kuiki, sift *Metrosideros* litter, 2145 m el., 16-v-2001 lot 02, Liebherr (CUIC, 6); Hana For. Res., Waihoi Vy., 1372 m el., 12-vii-1981, Montgomery (BPBM, 1).

##### Etymology.

The species epithet planipennis compounds the Latin planus, or even, flat, with the plural of penna, or wing; therefore planipennis, flat wings. The species epithet is treated as a noun in apposition.

##### Distribution and habitat.

*Mecyclothorax planipennis* is distributed from Paliku on the west to Waiho‘i Valley on the east, and across upper Kīpahulu Valley from West Camp on the north to Kuiki on the south (Fig. 86). Locality elevations range 1372–2145 m. Beetles have been found on mossy ‘ōhi‘a trunks and in ‘ōhi‘a leaf litter. They have also entered banana bait traps (Hardy, BPBM) and diphacinone rat traps (Takumi Kaholoa‘a, BPBM).

#### (067) Mecyclothorax planatus sp. n.

Taxon classificationAnimaliaColeopteraCarabidae

http://zoobank.org/547ECB73-B7B0-47D5-8CDD-4CE9EA083B5A

Figs 82I–L , 83C , 84C , 86 , 87B

##### Diagnosis.

This is the adelphotaxon to *Mecyclothorax planipennis*, treated immediately above, with the two species synapomorphically united by the anterior and posterior dorsal elytral depressions (Fig. 87A–B), and by the males possessing four marginal setae on the apical abdominal ventrite. The pronotal base exhibited by members of this species is narrower relative to maximum pronotal width; MPW/BPW = 1.49–1.60, and APW/BPW = 1.06–1.15. The discal elytral intervals within the anterior elytral depression are flatter in this species (Fig. 87B), and the male aedeagal median lobe is bluntly rounded (Figs 82I–L). Setal formula 2 2 2 2. Standardized body length 5.2–5.7 mm.

##### Description

(n = 5). As in the taxonomic treatments of other cryptic sibling species pairs in this revision, this description provides only those attributes and measurements that deviate from the description provided above for the sibling species, *Mecyclothorax planipennis*. Eyes tending to be more convex than in *Mecyclothorax planipennis*; ocular ratio = 1.47–1.53, ocular lobe ratio = 0.72–0.80. *Pronotum* transverse, MPW/PL = 1.30–1.37. *Elytra* subquadrate, but narrower basally than in *Mecyclothorax planipennis*, MEW/HuW = 1.91–2.02; discal strial punctures small, little expanding strial breadth within anterior elytral depression; lateral marginal depression extremely narrow at humerus, the lateral margin depression and basal groove similarly upraised laterad and mesad humeral angle.

**Male genitalia** (n = 5). Aedeagal median lobe robust, distance from parameral articulation to tip 3.2× depth at midlength (Fig. 82I, K–L); apex little extended beyond ostial opening, flattened on apical face, tip acutely rounded; median lobe curved rightward near apex, right and left margins subparallel near (Fig. 82J); internal sac with small dorsal ostial microtrichial patch and larger, heavily spiculated and projected ventral ostial microtrichial patch (Fig. 82K); flagellar plate very large, length 0.67× parameral articulation-tip distance.

**Female reproductive tract** (n = 1). Bursa copulatrix columnar, length 0.84 mm, breadth 0.46 mm (Fig. 83C); bursal walls translucent, thickly wrinkled; gonocoxite 1 with 4 apical fringe setae and 3 smaller setae on medial surface (Fig. 84C); gonocoxite 2 falcate, base extended laterally, 2 stout, apically narrowed lateral ensiform setae, apical nematiform setae on medial surface at 0.75× length of gonocoxite.

##### Holotype.

Male (CUIC) labeled: HI: Maui Haleakala NW / slope Waikamoi Pres. / trans. 3 @ 1700 m el. / 8-V-1991 scraping / ohia w/ moss & dirt // J.K. Liebherr / collector // Mecyclothorax / nsp platysminus / ♂ #1 EM / J.E. Hayden 2005 // HOLOTYPE / Mecyclothorax / planatus / Liebherr / det. J.K. Liebherr 2015 (black-margined red label).

##### Paratypes.

91 specimens (see Appendix).

##### Etymology.

The adjectival species epithet planatus is derived from the Latin planus, flat or level, and signifies the flattened qualities of beetles of this species.

##### Distribution and habitat.

*Mecyclothorax planatus* is broadly distributed across the higher elevation forests in the Waikamoi area, with a population known from the western reaches of Ko‘olau Gap (Fig. 86). It can occupy open shrubland such as occurs at Halemau‘u Trailhead (2438 m), or lower-elevation Koa-‘Ōhi‘a Forest (1210–2438 m). It has been found on koa trunks by scraping the flaky bark, or in moss on ‘ōhi‘a trunks. It has been collected also in sifted litter from ‘Ōhi‘a Forest, as well as under rocks resting on bare soil. That is has been a repeatedly encountered species since G.C. Munro collected a specimen at Olinda in 1936, but was not seen earlier by either Blackburn or Perkins suggests a fundamental change in relative abundances of *Mecyclothorax* species in this area over the last century.

#### (068) Mecyclothorax argutuloides sp. n.

Taxon classificationAnimaliaColeopteraCarabidae

http://zoobank.org/1CAD0940-F022-4D82-BB4D-F9473BA664AC

**Figure 88.**
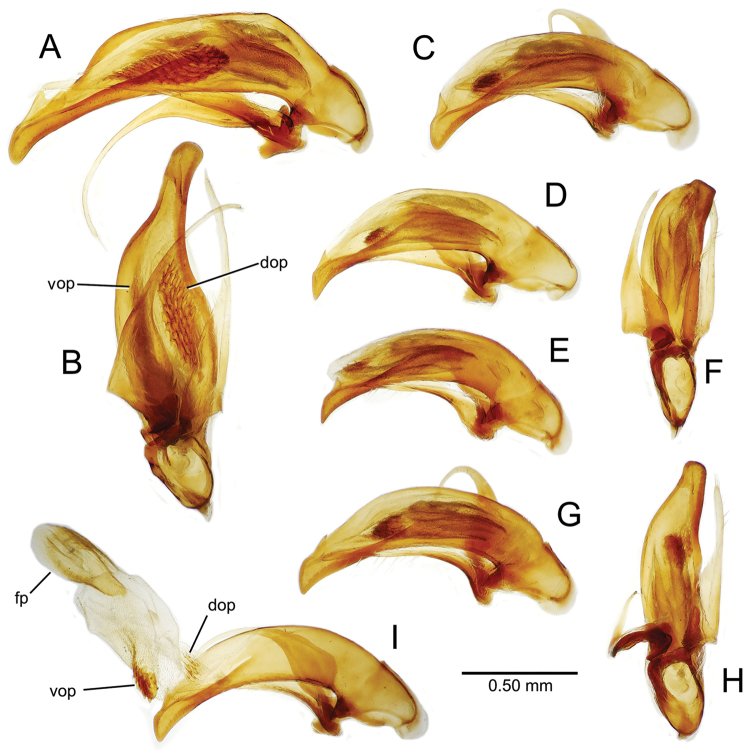
Male aedeagus, *Mecyclothorax argutor* group species (for abbreviations see Table 2, p. 23). **A–B** *Mecyclothorax argutuloides*, right and ventral views (Kīpahulu, 2100 m) **C–I** *Mecyclothorax cordithorax* **C** Right view (NW upper slope, 2065 m) **D** Right view (Kalapawili Ridge, 2475 m) **E–F** Right and ventral views (Kaupō Gap, 1736 m) **G–I** Right, ventral, and right view, sac everted (Polipoli, 1890 m).

Figs 83D , 84D , 86 , 87C , 88A–B

##### Diagnosis.

This species (Fig. 87C) looks like a smaller bodied, allometrically more conservative version of its adelphotaxon, *Mecyclothorax argutulus* (Fig. 81D). Both species share a little transverse, basally broad pronotum; MPW/PL = 1.19–1.22, MPW/BPW = 1.38–1.40 for this species; values that subsume the ratios derived from the unique *Mecyclothorax argutulus* holotype. However the body proportions of this species are narrower overall, leading to a more gracile appearance. The pronotal lateral marginal depression is narrow at midlength, and although the elytra are narrower in this species than in its robust relative, the elytra are still broader relative to the pronotum; MEW/MPW = 1.39–1.47 versus MEW/MPW = 1.38 in *Mecyclothorax argutulus*. The elytra tend to have more dorsal elytral setae present, though presence of these setae is also unstable in this species. Of the four specimens available, the setal counts for left plus right elytra are: 4 + 4, 4 + 3, 2 + 3, and 3 + 4; setal formula 2 2 3–4(2) 2. Standardized body length 5.3–5.6 mm, versus 6.2 mm for its closest relative.

##### Description

(n = 4). *Head capsule* with frontal grooves broad near clypeus, sinuous, broad convex carina to mesad anterior supraorbital seta; dorsal surface of neck slightly concave; eyes small, little convex, ocular lobe obtusely extended from ocular lobe, ocular ratio = 1.36–1.42, ocular lobe ratio 0.68–0.73; labral anterior margin broadly shallowly emarginate; antennomeres 2–3 with sparse pelage of short setae; mentum tooth with sides acute, apex rounded. *Pronotum* with lateral margin slightly convergent before acute hind angle, the angle acute even with convexly rounded margin behind angle; median base depressed relative to disc, rugose due to elongate wrinkles and punctures; basal margin broadly extended medially between laterobasal depressions, also convexly expanded posteriorly behind depressions to hind angle; median longitudinal impression finely incised, shallow; anterior transverse impression deeply incised, smooth, anterior callosity broadly convex, the callosity smooth with indistinct longitudinal wrinkles; front angles projected, tightly rounded; apical and basal pronotal widths subequal, APW/BPW = 0.96–1.02; lateral marginal depression narrow at midlength, broader at front angles, expanded in basal sinuation, margin upturned; laterobasal depression broadly concave, median tubercle present in some individuals. *Proepisternum* with 6 punctures along hind marginal groove; prosternal process medially concave, margin smoothly upraised. *Elytra* subquadrate, disc flat, sides moderately sloped; basal groove subangulate at base of sutural stria, extended to tightly angled humerus, the lateral marginal depression broader, its margin more upraised laterad humerus, MEW/HuW 1.75–1.85; parascutellar seta present; parascutellar striole deep, with 3–4 elongate punctures, smooth between punctures; sutural interval convexity only slightly more convex than lateral intervals, sutural juncture upraised at midlength, depressed basally and apically; sutural and 2^nd^ striae of subequal depth from base to apex; discal striae 1–5 present, the median 4 striae continuous, stria 5 partially interrupted, stria 6 reduced and stria 7 only traceable; discal strial punctures minute, only slightly expanding strial breadth and associated with strial irregularities; 8^th^ interval laterad 7^th^ stria of similar convexity to more mesal intervals; up to 4 dorsal elytral setae, at 0.22–0.24×, 0.38–0.46×, 0.52–0.52×, and 0.67–0.74× elytral length when all are present, setal impressions shallow, spanning about ½ width of interval 3; apical and subapical setae present; lateral elytral setae arranged in anterior series of 7 setae and posterior series of 6 setae; elytral marginal depression moderately broad, lined with sculpticells, margin upturned; subapical sinuation shallow, broad. *Mesepisternum* with ~7 shallow punctures on glossy surface; metepisternal width to length ratio = 0.80; metepisternum/metepimeron suture distinct; the metepisternal ratio is 0.88, and the suture indistinct in the robust adelphotaxon, *Mecyclothorax argutulus*. *Abdomen* with shallow indistinct lateral wrinkles on ventrites 1–3; suture between ventrites 2 and 3 complete; apical male ventrite with 2 setae, apical female ventrite with 4 equally spaced setae and median trapezoid of 4 subequal, short setae. *Legs*-metatarsomere 1/metatibial length ratio = 0.18; metatarsomere 4 length along outer lobe 1.4× medial tarsomere length, apical and subapical setae present; metatarsal dorsolateral sulci shallow, broad. *Microsculpture* of vertex a shallow isodiametric mesh in transverse rows; pronotal disc with shallow transverse mesh, median base with distinct isodiametric sculpticells; elytral disc with elongate transverse mesh, apex with reflective transverse mesh; metasternum with reflective transverse mesh; laterobasal abdominal ventrites with swirling isodiametric and transverse sculpticells. *Coloration* of vertex rufobrunneous; antennomeres 1–3 and base of 4 rufoflavous, balance of antennae with a piceous cast; pronotal disc rufobrunneous with a piceous cast, margins concolorous, depression rufoflavous at front angles; proepipleuron rufoflavous, proepisternum rufobrunneous with piceous cast; elytral disc reflective rufobrunneous, sutural interval concolorous basally, slightly paler apically; elytral margins narrowly rufoflavous in lateral depression, apex broadly paler, rufoflavous; elytral epipleuron rufoflavous, metepisternum rufobrunneous with piceous cast; abdomen rufobrunneous, slightly paler laterally, apical half of apical abdominal ventrite broadly paler, rufoflavous; metafemur rufoflavous; metatibia rufoflavous with brunneous cast.

**Male genitalia** (n = 1). Aedeagal median lobe robust, dorsal margin flat, distance from parameral articulation to tip 3.7× depth at midlength (Fig. 88A); apex elongate, with groove extended from ostial opening to downwardly curved, acutely rounded tip; median lobe apex slightly curved rightward in ventral view (Fig. 88B); median shaft broadly expanded medially, maximum breadth 0.32× parameral articulation-tip distance; internal sac with large dorsal ostial microtrichial patch (right patch in Fig. 88B), and smaller ventral ostial microtrichial patch; flagellar plate of moderate size, length 0.33× parameral articulation-tip distance.

**Female reproductive tract** (n = 1). Bursa copulatrix very elongate, slightly broader in apical half, length 1.37 mm, breadth 0.43 mm (Fig. 83D); bursal walls moderately opaque, thickly wrinkled; gonocoxite 1 with 3–4 apical fringe setae, 2 of the setae larger, the balance smaller (Fig. 84D), 7 smaller setae on medial surface; gonocoxite 2 narrowly falcate, base broadly, moderately extended laterally, 2 broad lateral ensiform setae (apices worn), apical nematiform setae on medial surface at 0.90× gonocoxite length.

##### Holotype.

Male (CUIC) labeled: HI:Maui Haleakala N.P. / Kipahulu Vy., 2100 m el. / 7-V-1991 sifting / leaf litter by day // S. Jessel / A.C. Medeiros, /Jr. collectors // Mecyclothorax / argutuloides / ♂ photo / det. J.K. Liebherr 2014 // HOLOTYPE / Mecyclothorax / argutuloides / Liebherr / det. J.K. Liebherr 2015 (black-margined red label).

##### Paratypes.

HI: Maui, Haleakala, Upper Hana For. Res., [= Wai‘anapanapa ], 2073 m el., 8-v-1973 (BPBM, 1); same data as holotype (CUIC, 2).

##### Etymology.

The adjectival species epithet argutuloides modifies the epithet argutulus proposed above for a closely related species, leading to a name that signifies “like the small shining” *Mecyclothorax argutulus*. Again Perrault’s (1984, 1986, 1988, 1989) convention of using a common stem for epithets of closely related species is employed.

##### Distribution and habitat.

*Mecyclothorax argutuloides* is known from Wai ‘anapanapa, 2070 m elevation in the Hāna Bogs area, and upper Kīpahulu Valley, 2100 m elevation (Fig. 86). The Kīpahulu specimen was collected in a sifted leaf litter sample.

#### (069) Mecyclothorax cordithorax

Taxon classificationAnimaliaColeopteraCarabidae

Liebherr

**Figure 89.**
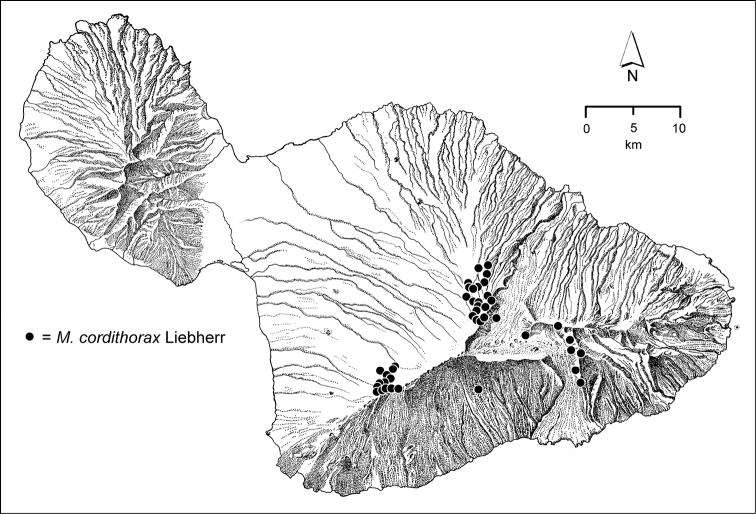
Recorded geographic distribution of *Mecyclothorax cordithorax*.

Figs 83E , 84E , 87D , 88C–I , 89

Mecyclothorax cordithorax Liebherr 2005b: 115.
Mecyclothorax robustus Sharp, Loope et al. 1988: 55, Cole et al. 1992: 1317 (misidentifications).

##### Diagnosis.

This, the most commonly encountered *Mecyclothorax* species on Haleakalā, can be diagnosed by the distinctly punctate elytral striae (Fig. 87D), little upraised sutural interval, and transverse pronotum with constricted base, MPW/PL = 1.25–1.38, MPW/BPW = 1.46–1.56. In body proportions this species approaches the *Mecyclothorax planipennis*-*Mecyclothorax planatus* species pair, but the cuticular microsculpture of *Mecyclothorax cordithorax* beetles is much smoother and more transverse, with the pronotal disc, for example, covered with a mixture of shallow transverse mesh and shallow transverse lines. Setal formula 2 2 2 2. Standardized body length 4.4–6.2 mm (vast majority of beetles > 4.6 mm length).

##### Identification

(n = 5). The eyes are moderately convex, ocular ratio = 1.40–1.48. The pronotal disc is smooth with shallow transverse wrinkles—looking like lap marks on a painted surface—with the distinctly depressed median base covered with isolated puncture and more rugose, longitudinal wrinkles. The elytral striation is well developed, with striae 1–4 continuous and deep to the elytral apex. Striae 5–6 are shallower laterally and apically, with 6 interrupted between some punctures, and stria 7 traceable only as a series of shallow, isolated elongate punctures. Stria 7 is deep on the elytral apex, with interval 8 medially convex laterad the deep stria. The microsculpture on the vertex comprises isodiametric sculpticells in transverse rows. The pronotal median base is covered with distinct isodiametric sculpticells resulting in a granulate surface. The elytral disc has upraised isodiametric sculpticells mixed with transversely stretched sculpticells, the resultant mesh with a reflective surface. The body is rufous to rufobrunneous with a variable piceous cast, contrasting with the flavous legs.

**Male genitalia** (n = 7). Aedeagal median lobe robust, distance from parameral articulation to tip 3.2× depth at midlength (Fig. 88D–E, G, I); apex little extended beyond ostial opening, flattened apically, tip acutely rounded (Fig. 88G, I) to acutely pointed (Fig. 88D–E); median lobe slightly curved rightward near apex in ventral view, left margin converging toward right to bluntly rounded tip (Fig. 88F, H); internal sac with small dorsal ostial microtrichial patch and larger, heavily spiculated and projected ventral ostial microtrichial patch (Fig. 88I); flagellar plate large, length 0.61× parameral articulation-tip distance.

**Female reproductive tract** (n = 3). Bursa copulatrix columnar, apically narrowed, length 0.97–1.03 mm, breadth 0.40–0.45 mm (Fig. 83E); bursal walls moderately opaque, thickly wrinkled; gonocoxite 1 with 4 apical fringe setae, 6–9 smaller setae on medial surface (Fig. 84E); gonocoxite 2 broad basally, subtriangular with angled apex, tip tightly rounded, 2 stout lateral ensiform setae, apical nematiform setae on medial surface at 0.75× gonocoxite length.

##### Holotype.

Female (CUIC) designated by Liebherr (2005b: 116). Type locality is HI: Maui, Haleakalā, Haleakalā N.P., Hosmer’s Grove, 2060 m el.

##### Distribution and habitat.

Of all of the open country, high-elevation species, *Mecyclothorax cordithorax* has a very broad distribution that is unique among Haleakalā *Mecyclothorax* (Fig. 89). It occupies shrubland habitats from 1600–2750 m elevation, but does not occur at Haleakalā summit. It occurs in Haleakalā Crater, but only where there is some topographic relief, such as along the margins at Paliku, Kaupō Gap, or Holua, or mid-crater at Pu‘u Māmane. Its native microhabitats include *Deschampsia nubigena* (hairgrass) tufts, leaf litter under *Sophora chrysophylla* (māmane), koa and ‘ōhi‘a trunks in mesic forest, as well as microhabitats associated with various other native plants: *Cheirodendron* (‘ōlapa), *Coprosma* (pilo), *Dryopteris* ferns, *Leptecophylla* (pūkiawe), *Pipturus* (māmaki), *Rubus* (‘ākala), and *Vaccinium* (‘ōhelo). It will occupy microhabitats on or near exotic species such as *Eucalyptus* or *Pinus ponderosa*. This exceedingly broad occurrence in shrubland habitats begs the question why neither Blackburn nor Perkins collected this species before Sharp’s (1903) revision of the Hawaiian Carabidae. The first specimen was collected by Perkins at 5000 ft. (1524 m) in 1913. Thereafter it was collected by a long line of Hawaiian entomologists and botanists; e.g. Giffard, Timberlake, Forbes, Swezey, Beardsley, Burkhart, Medeiros, Howarth, Stone, Kaholoa‘a, and Krushelnycky. The sudden appearance of this species leading to its status as the dominant shrubland carabid species speaks to an ecological resetting of this community during the early 20^th^ Century.

### *Mecyclothorax microps* species group

**Diagnosis.** Species assigned to this group are small bodied; standardized body length 3.2–4.5 mm. They exhibit outwardly flat eyes; ocular ratio = 1.28–1.39. The species are also characterized by absence of the parascutellar, and apical and subapical elytral setae. There may be 0, one, or two dorsal elytral setae, though there are always anterior and posterior supraorbital setae plus lateral and basal pronotal setae; setal formulae 2 2 2 0, 2 2 1 0, and 2 2 0 0.

**Membership and distribution.** Species assigned to this group are distributed on the vicariant portions of Maui Nui plus the island of Hawai‘i. In addition to the six Haleakalā species revised below, there are six species in West Maui (Liebherr 2011), one from Moloka‘i (Liebherr 2007), and two from Hawai‘i Island (Liebherr 2008b). Setal configuration varies substantially among the various island faunas. In contrast to the Haleakalā fauna, the Moloka‘i and West Maui species can be classified using formulae 2 2 2 2, 2 2 1 2, 2 2 2 1, and 2 2 1 1. The Hawai‘i Island species exemplify setal formulae 2 1 2 1 and 2 1 2 2. As the characters of flatter eyes and small body size currently define this group taxonomically, either setal evolution is very active within this group, or the “small eye + small body” syndrome has been polyphyletically derived on the various islands.

#### Key to adults of the *Mecyclothorax microps* species group, Haleakalā volcano, Maui, Hawai‘i

**Table:** 

1	Body size smaller, standardized body length 3.2–4.1 mm; elytra subquadrate to ellipsoid, lateral margins convex—slightly to distinctly—posterad humeri (Fig. 90B–F)	**2**
1’	Body size larger, standardized body length 4.5 mm; elytra quadrate, lateral margins straight, slightly divergent laterad anterior dorsal elytral setae (Fig. 90A)	(070) ***Mecyclothorax major* sp. n.**
2(1)	Pronotal base relatively broader (Fig. 90B–C), MPW/BPW = 1.30–1.35; elytra subquadrate, margins nearly straight posterad humerus, MEW/HuW = 1.74–1.81	**3**
2’	Pronotal base relatively narrower (Fig. 90D–F), MPW/BPW = 1.42–1.52; elytra more ellipsoid, lateral margins convex, MEW/HuW = 1.90–2.00	**4**
3(2)	Pronotum quadrate (Fig. 90B), MPW/PL = 1.18; elytra with transverse microsculpture, sculpticells 3–4× broad as long mixed with areas of transverse lines	(071) ***Mecyclothorax minor* Britton**
3’	Pronotum more transverse (Fig. 90C), MPW/PL = 1.21–1.30; elytral microsculpture a mixture of isodiametric and slightly transverse sculpticells arranged in transverse rows	(072) ***Mecyclothorax angusticollis* (Blackburn)**
4(2)	Elytral striae 1–6 impressed, punctate on disc (Fig. 90E–F); pronotal base broader, MPW/BPW = 1.42–1.49	**5**
4’	Elytral striae 1–2 impressed and continuous on disc, striae 3–6 discontinuous and progressively shallower toward lateral elytral margin (Fig. 90D); pronotal base narrower, MPW/BPW: basal pronotal width = 1.52	(073) ***Mecyclothorax xestos* sp. n.**
5(4)	Elytra with distinct, regular isodiametric mesh microsculpture, surface coriaceous; pronotum broader, more constricted basally, MPW/BPW = 1.49	(074) ***Mecyclothorax orbiculus* sp. n.**
5’	Elytra with distinct, regular transverse-mesh microsculpture, sculpticell breadth 2–4× length, lateral intervals covered with transverse lines; pronotum narrower, less constricted basally, MPW/BPW = 1.42–1.43	(075) ***Mecyclothorax contractus* sp. n.**

#### (070) Mecyclothorax major sp. n.

Taxon classificationAnimaliaColeopteraCarabidae

http://zoobank.org/14536137-E6A0-4750-85F8-DC1DF484B644

**Figure 90.**
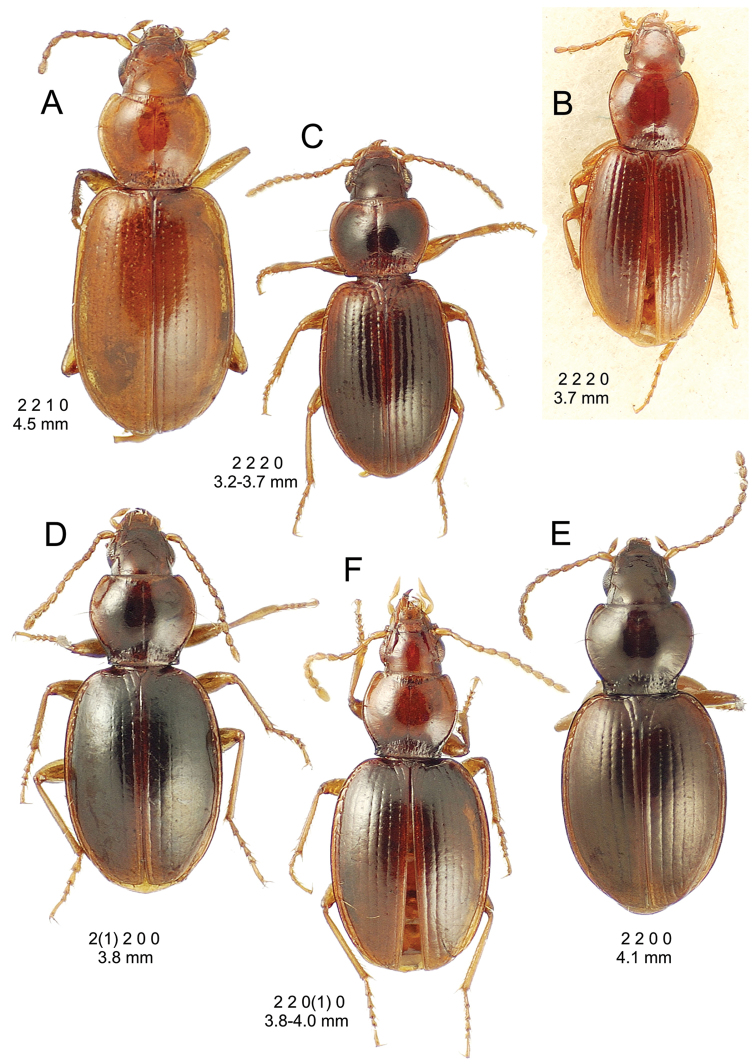
*Mecyclothorax microps* group species, dorsal habitus view. **A** *Mecyclothorax major* (Paliku, 1980 m) **B** *Mecyclothorax minor* (nr. Ukulele Camp, 1525 m) **C** *Mecyclothorax angusticollis* (below Ukulele Camp, 1372 m) **D** *Mecyclothorax xestos* (Kuhiwa, 1590 m) **E** *Mecyclothorax orbiculus* (Honomanu, 1750 m). **F** *Mecyclothorax contractus* (Kuhiwa, 1585 m).

**Figure 91.**
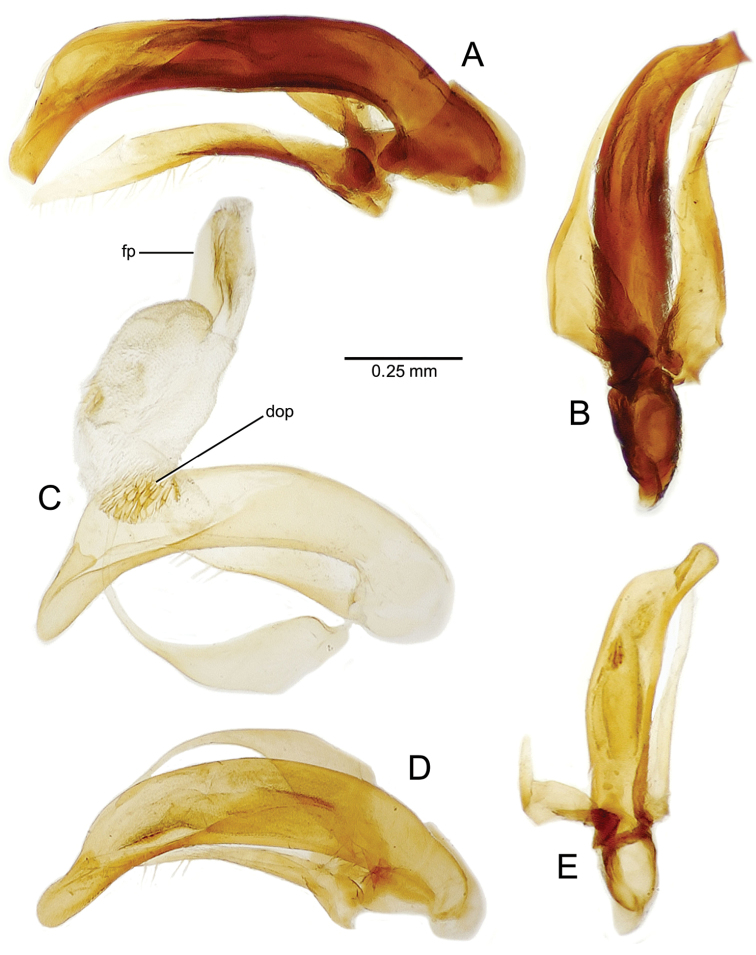
Male aedeagus, *Mecyclothorax microps* group species (for abbreviations see Table 2, p. 23). **A–B** *Mecyclothorax major*, right and ventral views (Paliku, 1981 m) **C** *Mecyclothorax minor* right view, sac everted (nr. Ukulele Camp, 1525 m) **D–E** *Mecyclothorax angusticollis*, right and ventral views (below Ukulele Camp, 1372 m).

Figs 90A , 91A–B , 94

##### Diagnosis.

With standardized body length 4.5 mm, this is the largest-bodied species of the *Mecyclothorax microps* group residing across all islands. Like *Mecyclothorax minor* (Fig. 90B) and *Mecyclothorax angusticollis* (Fig. 90C), the elytra are parallel sided and narrow relative to the pronotum (Fig. 90A); MEW/MPW = 1.42 for this species. The cuticle is more pallid than observed in *Mecyclothorax angusticollis*, and the pronotum is more constricted basally than in *Mecyclothorax minor*. Also, the elytra have a single dorsal elytral seta at the anterior position, resulting in the setal formula 2 2 1 0 unique to this species within the species group.

##### Description

(n = 1). *Head capsule* with frontal grooves convexly curved from clypeus to mesad anterior supraorbital seta, broad carina laterad convex groove; dorsal impression of neck slightly concave; eyes little convex, ocular lobe very obtusely extended from gena, ocular ratio = 1.31; ocular lobe ratio = 0.73; 10–11 ommatidia across horizontal diameter of eye; labral anterior margin angularly emarginate medially, impressed 1/7 length of labrum; antennomeres 2–3 with sparse pelage of short setae; antennae submoniliform; mentum tooth with sides acute, apex pointed. *Pronotum* little transverse, MPW/PL = 1.19, base moderately constricted, MPW/BPW = 1.49, with lateral margin straight to sinuate anterad obtuse hind angle; median base depressed relative to disc, covered with minute punctures basally and minute wrinkles at juncture with disc; basal margin slightly convex between laterobasal depressions; median longitudinal impression finely incised, shallow; anterior transverse impression broad, moderately deep laterally, obsolete medially; anterior callosity coplanar with disc medially, crossed by distinct wrinkles; front angles moderately produced, tightly rounded; pronotal apical and basal widths subequal, APW/BPW = 0.99; lateral marginal depression moderately broad with upturned margin before lateral seta, narrow with upraised margin to hind angle; laterobasal depression with irregular surface, continuous with lateral marginal depression. *Proepisternum* with 5–6 minute punctures along hind margin; prosternal process broadly depressed medially. *Elytra* subparallel, disc medially depressed, sides markedly depressed to margins; basal groove broadly recurved on rounded humeri, MEW/HuW = 1.84; parascutellar seta absent; parascutellar striole discontinuous, with 4–5 small punctures; sutural interval coplanar with lateral intervals basally, slightly upraised in apical half; sutural interval deeper and more steeply sided than 2^nd^ stria throughout most of length, striae of subequal depth at elytral apex; discal striae 1–4 shallow, punctate, striae 5–6 a series of isolated punctures, stria 7 absent near midlength; discal intervals 2–4 flat; 8^th^ interval laterad 7^th^ stria of same convexity as more mesal intervals; 1 dorsal elytral seta at 0.23–0.26× elytral length, setal impressions shallow, spanning ½ width of interval 3; apical and subapical setae absent; lateral elytral setae arranged in anterior series of 7 setae and posterior series of 5 setae; elytral marginal depression moderately broad with margin upraised laterad humerus, beadlike from midlength to subapical sinuation; subapical sinuation shallow, more abruptly incurved anteriorly. *Mesepisternum* smooth; metepisternal width to length ratio = 0.58; metepisternum/metepimeron suture distinct; metathoracic flight wing length 2.33× breadth, remnant R and M veins present, apex extended 0.75× wing remnant’s length beyond hind margin of metanotum (all estimated by viewing through elytra). *Abdomen* with indistinct lateral wrinkles on ventrites 1–5; suture between ventrites 2 and 3 reduced laterally, effaced; apical male ventrite with 2 marginal setae. *Legs*-metatarsomere 1/metatibial length ratio = 0.20; metatarsomere 4 length along outer lobe 1.5× medial tarsomere length, apical and subapical setae present; metatarsal dorsolateral sulci broad, basal tarsomeres medially subcarinate. *Microsculpture* of vertex an obsolete transverse mesh, sculpticell breadth 2× length; pronotal disc with obsolete transverse mesh, sculpticell breadth 2–4× length, portions of disc glossy, median base with mixture of isodiametric and transverse sculpticells, breadth 2× length; elytral disc with shallow isodiametric mesh in transverse rows, apex with same mesh obsolete; metasternum with distinct transverse mesh; laterobasal abdominal ventrites with swirling isodiametric and transverse microsculpture. *Coloration* of vertex rufoflavous; antennomere 1 flavous, 2–7 rufoflavous (outer antennomeres broken off); pronotal disc and margins rufoflavous; proepipleuron flavous, proepisternum rufoflavous; elytral disc and sutural interval rufoflavous, marginal depression narrowly flavous; elytral apex and apices of intervals 7 –9 flavous apicad terminus of interval 4; elytral epipleuron flavous dorsally, rufoflavous ventrally, metepisternum rufoflavous; abdomen rufoflavous, apical half of apical ventrite 6 flavous; metafemur rufoflavous; metatibia rufoflavous with brunneous cast.

**Male genitalia** (n = 1). Aedeagal median lobe slender, straight at midlength, shaft abruptly curved ventrally at base and apex (Fig. 91A); distance from parameral articulation to tip 5.4× depth at midlength; apex broadly extended beyond ostial opening, tip blunt with dorsal angle rounded and ventral angle denticulate; median lobe abruptly curved rightward ear apex in ventral view (Fig. 91B), blunt tip appearing concave in this view; internal sac with broad fields of dark microspicules but no apparent macrospicules (assessed in uneverted type specimen); right paramere parallel sided, broad nearly to apex (Fig. 91A).

##### Holotype.

Male (BPBM) dissected and labeled: Paliku, Haleakala / Crater, Maui 6500’ / VI–20-26–75 // R. Burkhart / Collector // pitfall trap // ACC. NO. 1990.009 / BISHOP Museum // HOLOTYPE / Mecyclothorax / major Liebherr / det. J.K. Liebherr 2015 (black-margined red label).

##### Etymology.

The Latin adjectival epithet major, i.e. larger, is used for this species in order to contrast it with the following *Mecyclothorax minor*.

##### Distribution and habitat.

*Mecyclothorax major* is known only from Paliku at the eastern margin of Haleakalā Crater (Fig. 94). The lone collecting event was in a pitfall trap, with the little developed eyes of this species also pointing to occupation of ground-level microhabitats.

#### (071) Mecyclothorax minor

Taxon classificationAnimaliaColeopteraCarabidae

Britton

Figs 90B , 91C , 94

Mecyclothorax minor Britton 1948b: 154.

##### Diagnosis.

This species (Fig. 90B) and *Mecyclothorax angusticollis* (Fig. 90C) represent the two species of the *Mecyclothorax microps* group characterized by: 1, a quadrate, basally broad pronotum, MPW/BPW = 1.30–1.35; 2, both anterior and posterior dorsal elytral setae present; and 3, elytra that are narrow relative to the forebody, MEW/MPW = 1.35–1.44, MEW/MHW = 1.93–2.12. Britton distinguished his *Mecyclothorax minor* from *Mecyclothorax angusticollis* based on the paler body color, and the broader pronotal base with non-sinuate lateral margins anterad the obtuse hind angles. Setal formula 2 2 2 0. Standardized body length 3.7 mm.

##### Identification

(n = 1). The eyes are small, ocular ratio = 1.36, but cover much of the non-protruded ocular lobe, ocular lobe ratio = 0.79. The labrum is medially emarginate to 1/5 labral length. The pronotal median base is nearly coplanar with disc, and covered with 8–10 minute, isolated punctures each side. The laterobasal depressions are not present, with that area broadly convex to the narrow lateral and basal margins. The elytra are narrow, with the basal groove distinctly recurved on the tightly rounded humeri, MEW/HuW = 1.80. The sutural and 2^nd^ striae are of equal development throughout their length, whereas striae 3–4 are of similar depth and punctation on the disc. Striae 5–6 are shallower and less punctate, and stria 7 shallower still and interrupted along its length. The 2 dorsal elytral setae are placed at 0.26–0.30× and 0.59–0.61× elytral length, with the setal impressions spanning half of interval 3. The vertex is covered with a shallow isodiametric and transverse mesh, sculpticell breadth 2× length; pronotal disc with transverse mesh, sculpticell breadth 2–4× length, the median base with mixture of isodiametric and transverse sculpticells; and elytral disc with transverse mesh, sculpticell breadth 3–4× length, and transverse lines, the apex with mixture of transverse sculpticells and parallel lines.

**Male genitalia** (n = 2). Aedeagal median lobe moderately gracile, distance from parameral articulation to tip 3.7× depth at midlength (Fig. 91C); apex extended for twice breadth beyond ostial opening, flattened on apical face, tip narrowly rounded; internal sac with a well-developed dorsal ostial microtrichial patch present, ventral surface broadly covered with microspicules; flagellar plate moderately large, length 0.45× parameral articulation-tip distance.

##### Holotype.

Male (BMNH), dissected and labeled: Type (round red-margined label) // Haleakala, / Maui 5000ft. / Perkins. V 1896 // near microps // Sharp Coll. / 1905-313. // Hawaiian Is. / R.C.L. Perkins. // Cyclothorax / minor sp. n. / E.B. Britton / det. 1939.

##### Distribution and habitat.

*Mecyclothorax minor* was collected in Perkins’ lot 600 (Anonymous N D) consistent with a collecting locality near Ukulele Camp (Fig. 94). It has not been collected since, though the mesic forest surrounding Ukulele Pipeline near that elevation has been visited on several occasions.

#### (072) Mecyclothorax angusticollis

Taxon classificationAnimaliaColeopteraCarabidae

(Blackburn)

**Figure 92.**
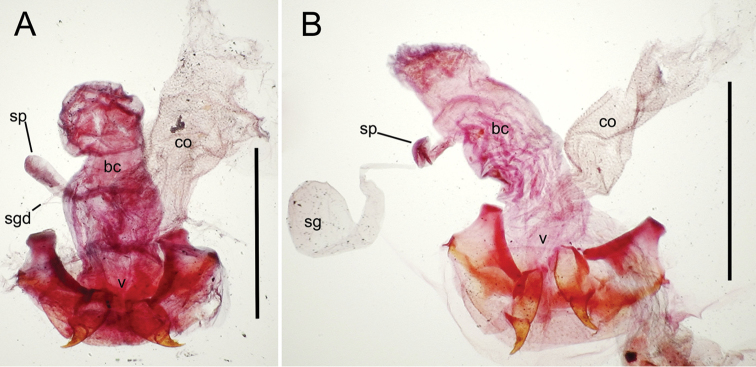
Female bursa copulatrix and associated reproductive structures, *Mecyclothorax microps* group species, ventral view (for abbreviations see Table 2, p. 23). **A** *Mecyclothorax angusticollis* (nr. Ukulele Camp, 1525 m) **B** *Mecyclothorax contractus* (Kuhiwa, 1585 m). Scale bar = 0.50 mm.

**Figure 93.**
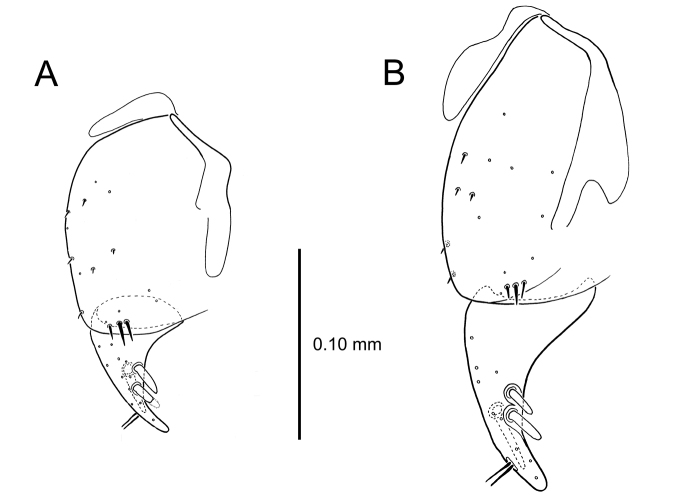
Left female gonocoxa, *Mecyclothorax microps* group species, ventral view. **A** *Mecyclothorax angusticollis* (nr. Ukulele Camp, 1525 m) **B** *Mecyclothorax contractus* (Kuhiwa, 1585 m).

**Figure 94.**
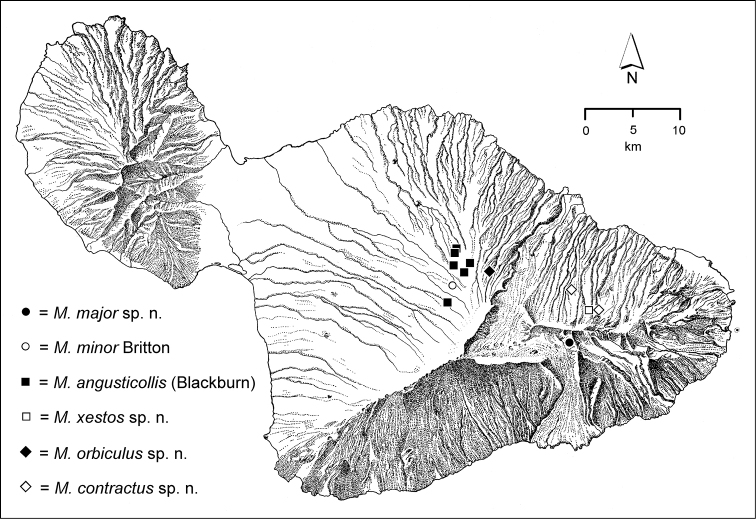
Recorded geographic distributions of *Mecyclothorax microps* group species.

Figs 90C , 91D–E , 92A , 93A , 94

Cyclothorax angusticollis Blackburn 1878b: 156; Blackburn and Sharp 1885: 216.
Mecyclothorax angusticollis , Sharp 1903: 246; Britton 1948b: 155.

##### Diagnosis.

This species is distinguished from all others in the group by the transverse pronotum (Fig. 90C), MPW/PL = 1.21–1.28, that is broad relative to the elytra, MEW/MPW = 1.35–1.39. Most similar to *Mecyclothorax minor*, *Mecyclothorax angusticollis* can also be distinguished from that species by the distinct isodiametric sculpticells in transverse rows on the elytral disc and apex. Body coloration is darker as well, with the pronotal disc rufobrunneous to rufopiceous, and the elytra rufobrunneous, in some instances with an apical rufopiceous cloud. Setal formula 2 2 2 0. Standardized body length 3.2–3.7 mm.

##### Identification

(n = 5). The eyes are relatively flat, ocular ratio = 1.32–1.39, and cover about ¾ of the ocular lobe, ocular lobe ratio = 0.75–0.79. The labrum is medially emarginate 1/5–1/6 of its length. The pronotal hind angles are obtuse rounded and little projected, the lateral margin only briefly subparallel before the angle. The pronotal median base is slightly depressed relative to the disc, but well set off by the dense elongate punctures and wrinkles, and the upraised isodiametric and transverse sculpticells over the surface. The elytral striae are well developed, with striae 1–6 and 8 complete and moderately deep on disc, stria 7 shallower. Striae 1–6 are distinctly punctate in basal ¾ of length. Sutural stria deep to apex, stria 2 shallower in apical half, except for deep portion parallel to deepened apex of stria 7. The 8^th^ interval is broadly convex, slightly more so than inner intervals. The lateral elytral setae are arranged in an anterior series of 6 setae and a posterior series of 4 setae.

**Male genitalia** (n = 2). Aedeagal median lobe gracile, distance from parameral articulation to tip 3.9× depth at midlength (Fig. 91D); apex extended for twice breadth beyond ostial opening, dorsal surface broadly expanded before evenly rounded tip; median lobe curved rightward near apex in ventral view, right and left margins parallel before bluntly rounded apex that is offset to the right (Fig. 91E); internal sac with small, well-sclerotized ventral ostial microtrichial patch (on left of sac, Fig. 91E); flagellar plate of moderate size, length 0.40× parameral articulation-tip distance (estimated by viewing ovoid sclerotized plate in ventral view, Fig. 91E).

**Female reproductive tract** (n = 1). Bursa copulatrix columnar with apical cap separated by constriction, overall length 0.67 mm, apical cap breadth 0.23 mm, medial shaft breadth 0.30 mm (Fig. 92A); bursal walls translucent with thin wrinkles; gonocoxite 1 with 3 small apical fringe setae and 6 very small setae on medial surface (Fig. 93A); gonocoxite 2 broadly subtriangular, apex evenly curved laterally, base evenly expanded laterally, 2 lateral ensiform setae, apical nematiform setae on medial surface at 0.67× gonocoxite length.

##### Lectotype.

Male (BMNH) hereby designated, labeled: mounting platen with Blackburn Maui code (Zimmerman 1957: 210), angustic (on reverse) // Type // Hawaiian Is. Rev. T. Blackburn 1888-30. // Lectotype Cyclothorax angusticollis Blackburn J.K.Liebherr 1998 (black-margined red label).

##### Distribution and habitat.

*Mecyclothorax angusticollis* was collected repeatedly by Blackburn and Perkins in the late 19^th^ Century (Perkins lots 112, 115, 120, 251, 350, 366, 367, 369, 371, 372, 597, 604, 605, 615, 680; Anonymous N D). Yet the only 20^th^ Century record was a single specimen collected by beating vegetation at 1830–1980 m elevation (E.C. Zimmerman, BPBM). This species appears to have been a species of the forest edge (Fig. 94), with the historical collecting sites now extensively disturbed, the old forest edge gone.

#### (073) Mecyclothorax xestos sp. n.

Taxon classificationAnimaliaColeopteraCarabidae

http://zoobank.org/073B775A-C8B2-496B-AE16-11DE11B2AB4D

Figs 90D , 94

##### Diagnosis.

This species (Fig. 90D) plus the following 2 species, *Mecyclothorax orbiculus* (Fig. 90E), and *Mecyclothorax contractus* (Fig. 90F), comprise the only species of the *Mecyclothorax microps* group that can be characterized by presence of both supraorbital setae and both pronotal setae, but much reduced elytral setation; no dorsal elytral setae or subapical and apical setae present; base setal formula 2 2 0 0. As the single known specimen of this species unilaterally lacks the anterior supraorbital seta on the right side, this species’ setal formula is scored 2(1) 2 0 0. As in all *Mecyclothorax microps* group species, the parascutellar seta is also absent. This setal configuration relegates the lateral elytral setae to be the sole setiform sensory organs of the dorsal hindbody. *Mecyclothorax xestos* exhibits the most reduced elytral striation of the three species, with: 1, only the sutural and 2^nd^ striae shallow, evident; 2, striae 3–4 traceable only as irregular, shallow grooves; 3, striae 5–6 very shallow, obsolete and interrupted; and 4, stria 7 absent basally, traceable only near elytral apex. Standardized body length 3.8 mm.

##### Description

(n = 1). *Head capsule* with frontal grooves narrow, a broad convexity laterad groove and fine carinae mesad eye; dorsal impression of neck broadly, slightly concave; eyes flat externally and small, ocular ratio = 1.28, ocular lobe ratio = 0.67; 9–10 ommatidia across horizontal diameter of eye; labral anterior margin angularly emarginate medially 1/7 of length; antennae nearly moniliform, antennomeres 2–3 with few very short setae; mentum tooth with sides acute, apex tightly rounded. *Pronotum* slightly transverse, MPW/PL = 1.20, basally constricted, MPW/BPW = 1.52; hind angle right to slightly acute, lateral margin subparallel to convergent for short distance anterad angle; median base depressed relative to disc, covered with longitudinal strigae; basal margin slightly convex between laterobasal depressions; median longitudinal impression finely incised, shallow but evident; anterior transverse impression broad, shallow, obsolete medially; anterior callosity coplanar with disc medially, slightly raised laterally, smooth, glossy; front angles not projected, tightly rounded; apical and basal pronotal widths subequal, APW/BPW = 0.98; lateral marginal depression narrow, edge upraised throughout length, slightly wider at front angle; laterobasal depression with irregular surface, continuous with lateral depression. *Proepisternum* with numerous small irregularities along hind marginal groove due to microsculpture; prosternal process broadly depressed medially. *Elytra* broadly subellipsoid, disc flat medially, moderately sloped at sides and apex; basal groove evenly curved anterad to angulate humeral juncture with lateral marginal depression, MEW/HuW = 1.90; parascutellar seta absent; parascutellar striole very shallow, smooth, hard to trace; sutural interval slightly upraised throughout length; discal intervals 2–4 flat; 8^th^ interval laterad 7^th^ stria more convex than more mesal intervals; elytral marginal depression moderately broad, margin upraised laterad humerus, beadlike from midlength to subapical sinuation; subapical sinuation shallow, more abruptly incurved anteriorly. *Mesepisternum* with ~9 punctures in 2–3 rows; metepisternal width to length ratio = 0.68; metepisternum/metepimeron suture distinct. *Abdomen* with indistinct lateral wrinkles on ventrites 1–3, circular depressions on ventrites 3–6; suture between ventrites 2 and 3 reduced laterally, effaced; apical female ventrite with 4 equally spaced marginal setae plus median trapezoid of 4 subequal, short setae. *Legs*-metatarsomere 1/metatibial length ratio = 0.20; metatarsomere 4 length along outer lobe 1.3× medial tarsomere length, apical and subapical setae present; metatarsal dorsolateral sulci broad, basal tarsomeres medially subcarinate. *Microsculpture* of vertex a very shallow isodiametric and transverse mesh, sculpticell breadth 2× length mesad eyes; pronotal disc with shallow transverse mesh, sculpticell breadth 2–4× length, median base with mixture of isodiametric and transverse sculpticells, breadth 2× length; elytral disc with regular transverse mesh, sculpticell breadth 2× length on disc, 3–4× length laterally, apex with transverse mesh, sculpticell breadth 2–3× length; metasternum with distinct transverse mesh; laterobasal abdominal ventrites with swirling isodiametric and transverse microsculpture. *Coloration* of vertex rufous, clypeus rufoflavous; antennomeres 1–3 flavous, 4–11 rufoflavous; pronotal disc rufous with brunneous cast, lateral margins, base, and apex broadly rufoflavous; proepipleuron flavous, proepisternum rufobrunneous; elytral disc rufobrunneous with reflective sheen, sutural interval rufoflavous basally, flavous apically; elytral lateral marginal depression and interval 9 rufoflavous, elytral apex and intervals 7–9 flavous apicad terminus of interval 4; elytral epipleuron flavous dorsally, rufoflavous ventrally, metepisternum rufobrunneous; abdominal ventrite 1 rufobrunneous, ventrites 2–3 medially rufopiceous, laterally rufoflavous, ventrites 4–5 and base of 6 rufoflavous, apical half of ventrite 6 flavous; metafemur flavous; metatibia flavous with rufoflavous cast.

**Female reproductive tract.** The lone female specimen was not dissected.

##### Holotype.

Female (CUIC) labeled: HI: Maui Haleakala / Hanawi N.A.R. Poouli / Cabin 5-V-1998 lot 01 / 1590m el. pyrethrum fog / mossy ohia J.K. Liebherr // HOLOTYPE / Mecyclothorax / xestos Liebherr / det. J.K. Liebherr 2015 (black-margined red label).

##### Etymology.

This species epithet is the Greek xestos, or scraped, planed, smoothed, or polished (Brown 1956).

##### Distribution and habitat.

The type specimen was collected near Kuhiwa Stream at 1590 m elevation in Hanawī (Fig. 94) in a pyrethrin fog samples of several moss-covered ‘ōhi‘a trees. Seven other *Mecyclothorax* spp. were also collected in this set of samples taken within a radius of 30 m: *Mecyclothorax crassuloides*, *Mecyclothorax kipwilli*, *Mecyclothorax mauiae*, *Mecyclothorax pau*, *Mecyclothorax perstriatus*, *Mecyclothorax poouli*, and *Mecyclothorax robustus*.

#### (074) Mecyclothorax orbiculus sp. n.

Taxon classificationAnimaliaColeopteraCarabidae

http://zoobank.org/2DCDA2D8-D039-489E-94CA-8FCD82303EB2

Figs 90E , 94

##### Diagnosis.

Among the triplet of *Mecyclothorax microps* group species without dorsal elytral setae, this species stands out due to the subellipsoid elytra, the lateral margins markedly convex from the narrowed humeri to the apex (Fig. 90E). The eyes are more convex than in either of the other species in the triplet, with ocular ratio = 1.38; versus 1.28–1.33 in the other two species. The elytral microsculpture also differs, being a distinct isodiametric mesh versus transverse mesh to transverse lines in the other two species. Setal formula 2 2 0 0. Standardized body length 4.1 mm.

##### Description

(n = 1). *Head capsule* with frontal grooves broad, shallow, a broad low convexity laterally before the eye and a very low, rounded carina mesad anterior supraorbital seta; dorsal impression of neck broadly, slightly concave; 14–15 ommatidia across horizontal diameter of eye; labral anterior margin angularly emarginate to 1/6 of labral length; antennae nearly moniliform, antennomeres 2–3 with sparse pelage of short setae; mentum tooth with sides acute, apex pointed. *Pronotum* slightly transverse, MPW/PL = 1.22, moderately constricted basally, MPW/BPW = 1.49; hind angle obtuse due to convex margin behind angle; lateral margins slightly convergent anterad sharply projected hind angles; median base moderately depressed relative to disc, ~14 punctures each side; basal margin slightly convex between laterobasal depressions; median longitudinal impression finely incised, shallow but evident; anterior transverse impression broad, shallow, obsolete medially; anterior callosity coplanar with disc medially, slightly raised laterally, smooth, glossy; front angles slightly projected, tightly rounded; apical pronotal width greater than basal width, APW/BPW = 1.05; lateral marginal depression narrow, the edge beadlike to upturned near broader area at front angle; laterobasal depression with irregular surface, continuous with lateral depression. *Proepisternum* with 5–6 minute punctures along hind marginal groove; prosternal process broadly depressed medially. *Elytra* with narrow humeri, basal groove distinctly curved to meet lateral marginal depression at subangulate humerus, MEW/HuW = 1.91; parascutellar striole shallow, with 4 punctures; sutural interval slightly upraised relative to lateral intervals throughout its length; sutural and 2^nd^ striae of subequal depth from base to apex; discal striae 1–6 progressively shallower, stria 7 traceable though interrupted repeatedly; discal striae 1–5 shallowly punctate, minute elongate punctulae in the base of stria 6, all striae smooth and complete on elytral apex; elytral intervals slightly convex medially, nearly flat laterally; 8^th^ interval laterad 7^th^ stria subcarinate, more convex than more mesal intervals; lateral elytral setae arranged in anterior series of 7 setae and posterior series of 6 setae; elytral marginal depression broader, margin slightly upraised at humerus, narrow, beadlike from midlength to subapical sinuation; subapical sinuation shallow, more abruptly incurved anteriorly. *Mesepisternum* with ~10 punctures in 2–3 rows; metepisternal width to length ratio = 0.72; metepisternum/metepimeron suture distinct. *Abdomen* with indistinct lateral wrinkles on ventrites 1–3 and circular lateral depressions on ventrites 3–6; suture between ventrites 2 and 3 reduced laterally, effaced; apical female ventrite with 4 equally spaced setae and median trapezoid of 4 subequal, short setae. *Legs*-metatarsomere 1/metatibial length ratio = 0.19; metatarsomere 4 length along outer lobe 1.3× medial tarsomere length, apical and subapical setae present; metatarsal dorsolateral sulci broad, basal tarsomeres medially subcarinate. *Microsculpture* of vertex a shallow isodiametric mesh, transverse sculpticells, breadth 2× length mesad eyes; pronotal disc with transverse mesh, sculpticell breadth 2–4× length, median base with mixture of isodiametric and transverse sculpticells, breadth 2× length; elytral apex with mixture of isodiametric and transverse sculpticells in transverse rows; metasternum with distinct transverse mesh; laterobasal abdominal ventrites with swirling isodiametric and transverse microsculpture. *Coloration* of vertex rufobrunneous with rufoflavous clypeus; antennomeres 1–3 flavous, 4–11 rufoflavous; pronotal disc rufous with brunneous cast, lateral margins, base, and apex broadly rufoflavous; proepipleuron rufoflavous, proepisternum rufobrunneous; elytral disc rufobrunneous with indistinct purplish sheen, sutural interval rufoflavous basally, flavous in apical 1/3, elytral lateral margin depression, 9^th^ interval and apex rufoflavous; elytral epipleuron flavous dorsally, rufoflavous ventrally, metepisternum rufobrunneous; abdomen with abdominal ventrite 1 rufobrunneous, ventrites 2–3 medially rufopiceous, ventrites 4–6 rufoflavous, with apical half of ventrite 6 flavous; metafemur flavous; metatibia flavous with rufoflavous cast.

**Female reproductive tract.** The lone female specimen was not dissected.

##### Holotype.

Female (BPBM) labeled: HI: Maui Is. Haleakala / Waikamoi N.C.P. 1750 m el. / 20°47.21'N, 156°13.82'W / 12-III-2002 R. Takumi / pyr. fog mossy ohia // HOLOTYPE / Mecyclothorax / orbiculus / Liebherr / det. J.K. Liebherr 2015 (black-margined red label).

##### Etymology.

The species epithet orbiculus is the diminutive of orbis, or circle, and signifies the small eyes characteristic of this species.

##### Distribution and habitat.

The type specimen of this species was collected at 1750 m elevation within the Honomanu drainage of the Waikamoi Nature Conservancy Preserve (Fig. 94). It was found in moss adhering to an ‘ōhi‘a trunk.

#### (075) Mecyclothorax contractus sp. n.

Taxon classificationAnimaliaColeopteraCarabidae

http://zoobank.org/F9362BDC-3674-45F6-B528-9FF0A233F9AF

Figs 90F , 92B , 93B , 94

##### Diagnosis.

This third species of the triplet lacking dorsal elytral setae can be diagnosed by the more quadrate pronotum (Fig. 90F); MPW/PL = 1.07–1.13. The pronotum is relatively less constricted basally in association with the quadrate shape; MPW/BPW = 1.42–1.43. The elytra are subquadrate, the lateral margins extended laterally outside the distinctly angulate humeri, and broader relative to the pronotum than in other *Mecyclothorax microps* group species; MEW/MPW = 1.56–1.59. Setal formula 2 2 0(1) 0; 1 of the 3 specimens has the anterior dorsal elytral seta present. Standardized body length 4.0 mm.

##### Description

(n = 3). *Head capsule* with frontal grooves broad at clypeus, sinuous, with broad convexity laterad groove and thin carina mesad supraorbital seta; dorsal impression of neck slightly concave; ocular lobe little projected, eyes small, little convex, vertical dimension greater than length, ocular ratio = 1.29–1.33, ocular lobe ratio = 0.73–0.78, about 10 ommatidia across horizontal diameter of eye; labral anterior margin angularly emarginate medially, impressed 1/6 length; antennomere 2 with sparse setae at apex, antennomere 3 sparsely setose along shaft; mentum tooth with sides acute, apex pointed. *Pronotum* with lateral margins broadly sinuate anterad slightly obtuse, projected hind angles, margin behind angle convex; median base depressed relative to disc, covered with punctures and longitudinal strigae; basal margin slightly convex between laterobasal depressions; median longitudinal impression finely incised, shallow but evident, continued onto median base; anterior transverse impression broad, shallow, surface slightly irregular; anterior callosity coplanar with disc medially, slightly raised laterally, smooth, glossy; front angles not projected, rounded; apical and basal pronotal widths subequal, APW/BPW = 0.97–1.04; lateral marginal depression broadest inside front angles, narrow with beadlike margins in anterior half, margin upraised basally; laterobasal depression with irregular surface, continuous with lateral depression. *Proepisternum* with 5–6 minute punctures along hind marginal groove; prosternal process broadly depressed medially. *Elytra* subquadrate, the disc flat medially, moderately sloped on sides and apex; the humeral angles are proximate relative to elytral width, MEW/HuW = 1.95–2.0; parascutellar striole with 3 punctures, the striole interrupted between punctures; sutural interval slightly more upraised throughout its length than lateral intervals; sutural striae slightly deeper than 2^nd^ stria basally, of similar depth apically; discal striae 1–4 moderately impressed, minutely punctate, intervals 5–6 shallow but evident with elongate irregularities along length, stria 7 obsolete, interrupted; 8^th^ interval laterad 7^th^ stria of same convexity as more mesal intervals; 1 dorsal elytral seta present in 1 of 3 specimens at 0.23–0.26× elytral length, setal impression shallow, spanning ½ width of interval 3; lateral elytral setae arranged in anterior series of 6(7) setae and posterior series of 5(6) setae; elytral marginal depression moderately broad with upraised margin outside humerus, beadlike from midlength to subapical sinuation; subapical sinuation shallow, more abruptly incurved anteriorly. *Mesepisternum* with ~7 punctures in 2 rows; metepisternal width to length ratio = 0.88; metepisternum/metepimeron suture distinct; metathoracic flight wing 2.33× long as broad, with remnant R and M veins, the vestigium extended for 0.75× its length beyond hind margin of metanotum. *Abdomen* with indistinct lateral wrinkles on ventrites 1–5; suture between ventrites 2 and 3 reduced laterally, effaced; apical female ventrite with 4 equally space marginal setae plus median trapezoid of 4 subequal, short setae. *Legs*-metatarsomere 1/metatibial length ratio = 0.19; metatarsomere 4 length along outer lobe 1.5× medial tarsomere length, apical and subapical setae present; metatarsal dorsolateral sulci broad, basal tarsomeres medially subcarinate. *Microsculpture* of vertex very shallow isodiametric and transverse mesh, sculpticell breadth 2× length mesad eyes; pronotal disc with shallow transverse mesh, sculpticell breadth 2–4× length, median base with mixture of isodiametric and transverse sculpticells, breadth 2× length, apex with transverse mesh, sculpticell breadth 4× length; metasternum with distinct transverse mesh; laterobasal abdominal ventrites with swirling isodiametric and transverse microsculpture. *Coloration* of vertex rufous; antennomeres 1–3 flavous, 4–11 rufoflavous; pronotal disc rufous, lateral margins, base, and apex broadly rufoflavous; proepipleuron rufoflavous, proepisternum rufobrunneous; elytral disc rufobrunneous with reflective sheen, sutural interval rufoflavous basally, flavous apically; elytral marginal depression and 9^th^ interval rufoflavous, intervals 7–9 and apex apicad terminus of interval 4 tending toward flavous; elytral epipleuron flavous dorsally, rufoflavous ventrally, metepisternum rufobrunneous; abdominal ventrite 1 and 4–6 rufoflavous, ventrites 2–3 rufobrunneous, apical half of apical ventrite 6 flavous; metafemur flavous; metatibia flavous with rufoflavous cast.

**Female reproductive tract** (n = 1). Bursa copulatrix columnar with rounded apex, length 0.67 mm, breadth 0.23 mm (Fig. 92B); bursal walls translucent with thin wrinkles; gonocoxite 1 with 3 small apical fringe setae and 5–6 smaller setae on medial surface (Fig. 93B); gonocoxite 2 narrowly subtriangular, apex rounded, base narrowly extended laterally, 2 lateral ensiform setae with apical seta broader and longer, apical nematiform setae on medial surface at 0.75× gonocoxite length.

##### Holotype.

Female (CUIC) labeled: HI: Maui Hanawi N.A.R. / Pig fence helipad sift / humus ex ohia 21-V- / 1993 lot 02 el. 1575 m // 2 // HOLOTYPE / Mecyclothorax / contractus / Liebherr / det. J.K. Liebherr 2015 (black-margined red label).

##### Paratypes.

HI: Maui, Haleakala, Hanawi N.A.R., Kopiliula Str., pyrethrin fog *Cibotium*+*Metrosideros*, 1137 m el., 4-v-1998 lot 02 (CUIC, 1), same data as holotype (CUIC, 1).

##### Etymology.

The adjectival species epithet contractus means drawn together, or made narrow, and refers to the basally constricted pronotum of beetles in this species.

##### Distribution and habitat.

This species is known from collecting sites in Hanawī near Kuhiwa and Kopili‘ula Streams, elevations 1137–1585 m (Fig. 94). Two beetles were found in a sift sample of humus associated with a shrublike ‘ōhi‘a tree at the former site, and a third was found in a pyrethrin fog sample of a *Cibotium* tree fern on a horizontal, mossy ‘ōhi‘a nurse log in the Kopili‘ula drainage.

### *Mecyclothorax scaritoides* species group

**Diagnosis.** Species classified in this group are characterized by much reduced elytral striation, with the 2^nd^ stria less developed basally than the sutural stria, and lateral elytral striae 3–7 very shallow to absent. The apical and subapical elytral setae are always absent from beetles comprising the Haleakalā, Moloka‘i and O‘ahu species placed in this group, though both dorsal elytral setae are always present, and the parascutellar seta is present in all species except *Mecyclothorax macrops*. Additionally, the narrow lateral margins of the pronotum are associated with absence of at least the basal seta in all Haleakalā species, and the absence of both lateral and basal setae in four of the nine Haleakalā species. The supraorbital setal condition is also variable, with the anterior supraorbital seta absent from beetles comprising three of the nine Haleakalā species. The dorsal microsculpture is reduced, and distinctly transverse when visible. The setal formula is 2 1 2 0 or 1 0 2 0 in the Haleakalā species. Most of the species comprise larger bodied beetles, standardized body length 4.5–7.4 mm, with only *Mecyclothorax gracilicollis* sp. n. deviating by its small size, 3.8 mm.

**Membership and distribution.** This group is among the most broadly distributed of all species groups in Hawai‘i, with the nine Haleakalā species complemented by seven species from O‘ahu (Liebherr 2009a), three from Moloka‘i (Liebherr 2007), one from West Maui (Liebherr 2011), and six from Hawai‘i Island (Liebherr 2008b). All species recorded from O‘ahu, Moloka‘i and Haleakalā are characterized by the derived states of a glabrous pronotum and absence of both apical and subapical elytral setae. The lone West Maui species, *Mecyclothorax crassus* (Sharp), is characterized by glabrous pronotum and a single supraorbital seta, but both apical and subapical elytral setae are present. More disparately, 5 of the 6 Hawai‘i Island species are characterized by having both apical and subapical elytral setae present, and either 1 or 2 pronotal setae present. Thus setal evolution patterns are more consistent with the majority of species-level diversification having taken place within islands, especially on the Big Island of Hawai‘i, than by rampant exchange of lineages among islands.

#### Key to adults of the *Mecyclothorax scaritoides* species group, Haleakalā volcano, Maui, Hawai‘i

**Table:** 

1	Pronotum glabrous, both lateral and basal setae absent, lateral margin carinate, without depression adjoining disc (Fig. 95)	**2**
1’	Pronotal lateral seta present, basal seta absent, lateral marginal depression present between disc and upturned edge (Fig. 100)	**5**
2(1)	Very large, standardized body length 6.1–7.4 mm; pronotal hind angles little projected, lateral margin little sinuate before angle (Fig. 95A–B)	**3**
2’	Smaller, standardized body length 3.8–5.8 mm; pronotal hind angles projected, lateral margin distinctly sinuate before angle (Fig. 95C–D)	**4**
3(2)	Pronotal hind angles evident though rounded apically, median base with single irregular transverse row of punctures, otherwise smooth (Fig. 95A); standardized body length 7.4 mm	(076) ***Mecyclothorax molops* (Sharp)**
3’	Pronotal hind angles distinctly obtuse, lateral margin distinctly, briefly sinuate before angle, median base defined anteriorly at disc by well-marked transverse row of elongate punctures, punctures also present more basally (Fig. 95B); standardized body length 6.1–6.4 mm	(077) ***Mecyclothorax macrops* (Sharp)**
4(2)	Elytral disc with distinct isodiametric microsculpture arranged in transverse rows, sculpticells upraised; male aedeagal median lobe with broadly rounded apex bearing a bluntly rounded dorsal projection (Fig. 96C)	(078) ***Mecyclothorax scaritoides* (Blackburn)**
4’	Elytral disc with transverse microsculpture, a mixture of transverse-mesh microsculpture with sculpticells 3–4× broad as long, and transverse lines not joined into a mesh; male aedeagal median lobe with elongate, clublike apex bearing a distinctly expanded dorsal projection (Fig. 96D)	(079) ***Mecyclothorax scarites* sp. n.**
5(1)	Elytral striae 2–5 obsolete, barely traceable, the elytral surface glossy (Fig. 100A–C); pronotum constricted basally, MPW/BPW = 1.63–1.72	**6**
5’	Elytral striae 2–5 indicated, shallow but traceable on disc, surface less uniformly glossy due to curvature of intervals (Fig. 100D–E); pronotum less constricted basally, MPW/BPW = 1.51–1.59	**8**
6(5)	Lateral pronotal margins convergent anterad hind angle for 0.100.12× pronotal length (Fig. 100B–C), pronotum broader, MPW/PL = 1.20–1.31; male aedeagal median lobe attenuate, recurved to pointed tip (Fig. 96I–J)	**7**
6’	Lateral pronotal margins parallel anterad hind angle for 0.2× pronotal length (Fig. 100A), pronotum narrower, MPW/PL = 1.16; male aedeagal median lobe narrowly elongate and evenly downturned apically (Fig. 96F)	(080) ***Mecyclothorax timberlakei* sp. n.**
7(6)	Elytra disc with indistinct isodiametric sculpticells in transverse rows, sculpticell margins not visible in reflected light, elytra apex glossy, microsculpture obsolete; male aedeagal median lobe apex robust, ventrally acuminate (Fig. 96I)	(081) ***Mecyclothorax crassuloides* sp. n.**
7’	Elytral disc with distinct isodiametric sculpticells, their margins visible in reflected light, elytral apex with evident isodiametric mesh; male aedeagal median lobe apex gracile, tip expanded both dorsally and ventrally (Fig. 96J)	(082) ***Mecyclothorax crassulus* sp. n.**
8(5)	Smaller, standardized body length 3.8 mm (Fig. 100D); discal elytral intervals with regular transverse-mesh microsculpture, sculpticell breadth 2–3× length; pronotal base narrower, MPW/BPW = 1.59	(083) ***Mecyclothorax gracilicollis* sp. n.**
8’	Larger, standardized body length 4.8 mm (Fig. 100E); discal elytral intervals glossy, indistinct transverse lines visible over portions of cuticle; pronotal base broader, MPW/BPW = 1.51	(084) ***Mecyclothorax dispar* sp. n.**

#### (076) Mecyclothorax molops

Taxon classificationAnimaliaColeopteraCarabidae

(Sharp)

Figs 95A , 99

Metrothorax molops Sharp 1903: 269.
Mecyclothorax molops , Britton 1948b: 119.

##### Diagnosis.

**Figure 95.**
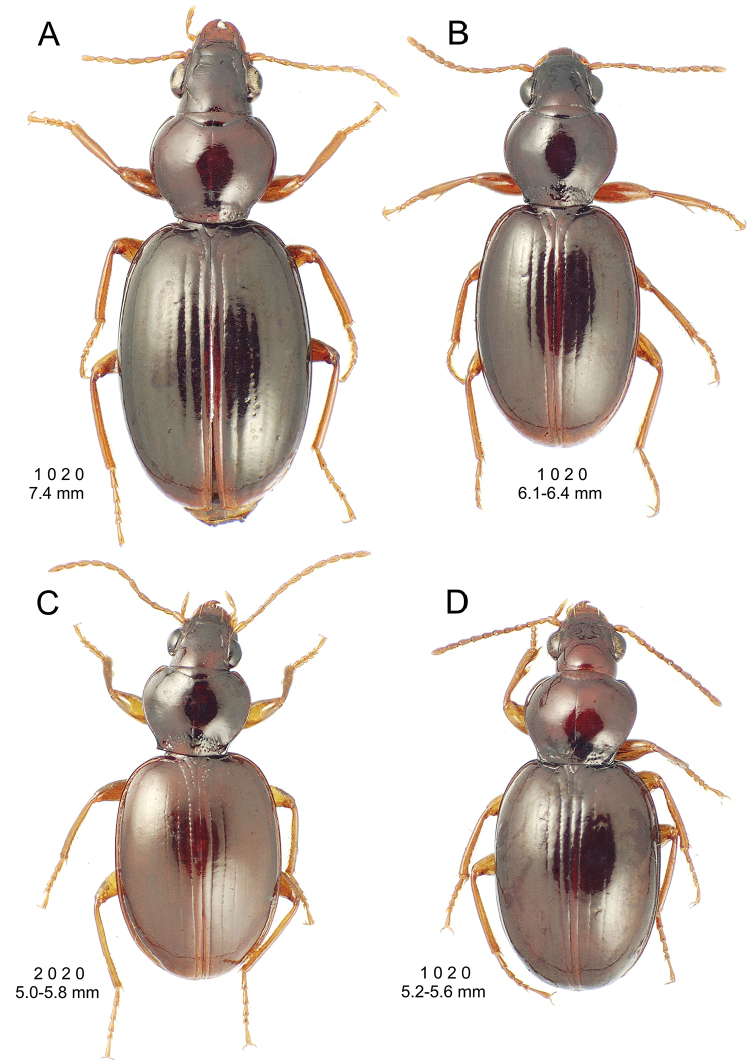
*Mecyclothorax scaritoides* group species, dorsal habitus view. **A** *Mecyclothorax molops* (nr. Ukulele Camp, 1525 m) **B** *Mecyclothorax macrops* (Olinda, 1210 m) **C** *Mecyclothorax scaritoides* (above Olinda, 1210–1365 m) **D** *Mecyclothorax scarites* (Olinda, 1280 m).

Although known only from two specimens collected by R.C.L. Perkins in 1894 and 1896, new specimens of this species would be instantly recognizable by the presence of only the posterior supraorbital seta, glabrous pronotum with carinate lateral margins, minimally punctate pronotal median base (Fig. 95A), and very large body size; standardized body length 7.4 mm (paratype female). Setal formula 1 0 2 0, and the parascutellar seta is present. Britton (1948b) records the body length as 7.2–7.8 mm based on his measurements of holotype and paratype females.

##### Identification

(n = 1). These are very robust, large-bodied beetles. The eyes are moderately convex, covering much of the protruded ocular lobes; ocular ratio = 1.48, ocular lobe ratio = 0.79. The pronotum is vase shaped, MPW/PL = 1.15, the base narrowly constricted, MPW/BPW = 1.73. The median base bears ~10 very shallow, isolated punctures each side, the cuticle glossy over the entire surface, and the anterior transverse impression is deep and very smooth, bordered anterior by the convex, smooth and glossy anterior callosity. The elytra are an elongate ovoid shape, with narrowly rounded base. The evenly curved basal grooves meet their respective lateral margins at the proximate, rounded to subangulate humeri; MEW/HuW = 2.30. The 2^nd^ stria is nearly as deep as the sutural stria near elytral midlength, but it is absent basally whereas the sutural stria can be traced basally by a series of fine punctures. The much reduced microsculpture includes: 1, vertex with obsolete transverse mesh, sculpticell breadth 2× length, the surface glossy; 2, pronotal disc with obsolete transverse mesh in part, glossy between areas of sculpticells; 3, pronotal median base glossy, indistinct transverse sculpticells laterally; 4, elytral disc with shallow transverse lines irregularly joined into a loose mesh, apex glossy, microsculpture obsolete there; 5, metasternum with shallow transverse mesh, sculpticell breadth 3–4× length; and 6, laterobasal abdominal ventrites with swirling isodiametric and transverse microsculpture.

**Female reproductive tract.** The female lectotype (BMNH) was not dissected.

##### Lectotype.

Female (BMNH) hereby designated, labeled: Metrothorax / molops / Type / D.S. / ♀ (pencil) Haleakala / Perkins 413 (ink on mounting platen) / Type (round red-margined label) / Haleakala / Maui 5000 ft. / Perkins. III 1894. // Hawaiian Is. / Perkins / 1904–336. // LECTOTYPE / Metrothorax / molops Sharp / J.K. Liebherr 1998 (black-margined red label).

##### Distribution and habitat.

*Mecyclothorax molops* is known from two specimens (Perkins lots 413, 612), which correspond to collections made near Ukulele Camp (Fig. 99) in iii-1894 and v-1896 (Perkins 1896a). The species has not been recollected since.

#### (077) Mecyclothorax macrops

Taxon classificationAnimaliaColeopteraCarabidae

(Sharp)

**Figure 96.**
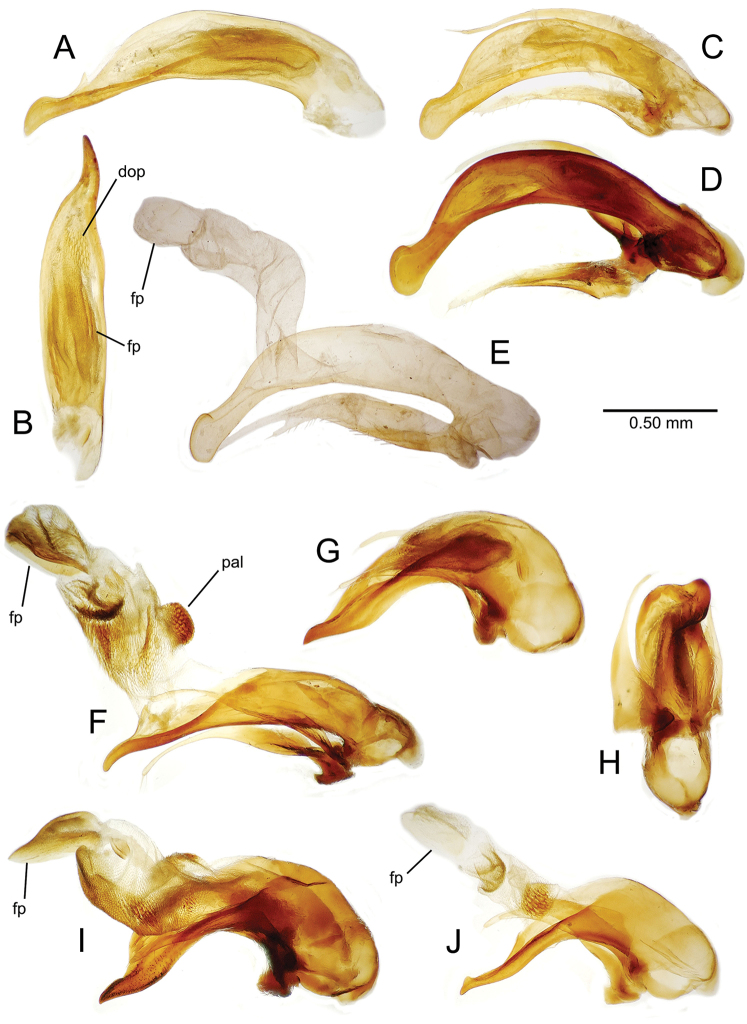
Male aedeagus, *Mecyclothorax scaritoides* group species (for abbreviations see Table 2, p. 23). **A–B** *Mecyclothorax macrops*, right and ventral views (nr. Ukulele Camp, 1372–1830 m) **C** *Mecyclothorax scaritoides* right view (Olinda, 1210 m) **D–E** *Mecyclothorax scarites* **D** Right view (Waikamoi, 975–1210 m) **E** Right view, sac everted (Olinda, 1280 m) **F** *Mecyclothorax timberlakei* (Ke‘anae Pali, 1525 m) **G–I** *Mecyclothorax crassuloides* (Kuhiwa E rim, 880–910 m) **G** Right view **H** Ventral view **I** Right view, sac everted **J** *Mecyclothorax crassulus*, right view, sac everted (Honomanu, 1700 m).

**Figure 97.**
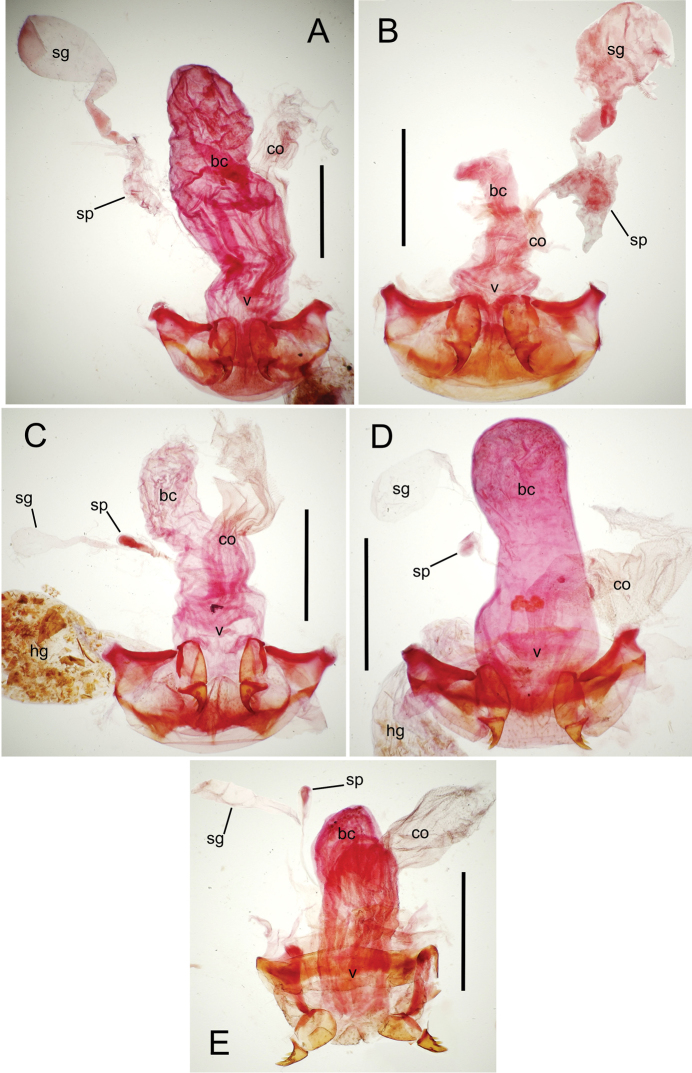
Female bursa copulatrix and associated reproductive structures, *Mecyclothorax scaritoides* group species, ventral view (for abbreviations see Table 2, p. 23). **A** *Mecyclothorax macrops* (Olinda, 1210 m) **B** *Mecyclothorax scaritoides* (nr. Ukulele Camp, 1525 m) **C** *Mecyclothorax scarites* (Olinda, 1280 m) **D** *Mecyclothorax crassuloides* (Midcamp Bog, 1665 m) **E** *Mecyclothorax crassulus* (Honomanu, 1680 m). Scale bar = 0.50 mm.

**Figure 98.**
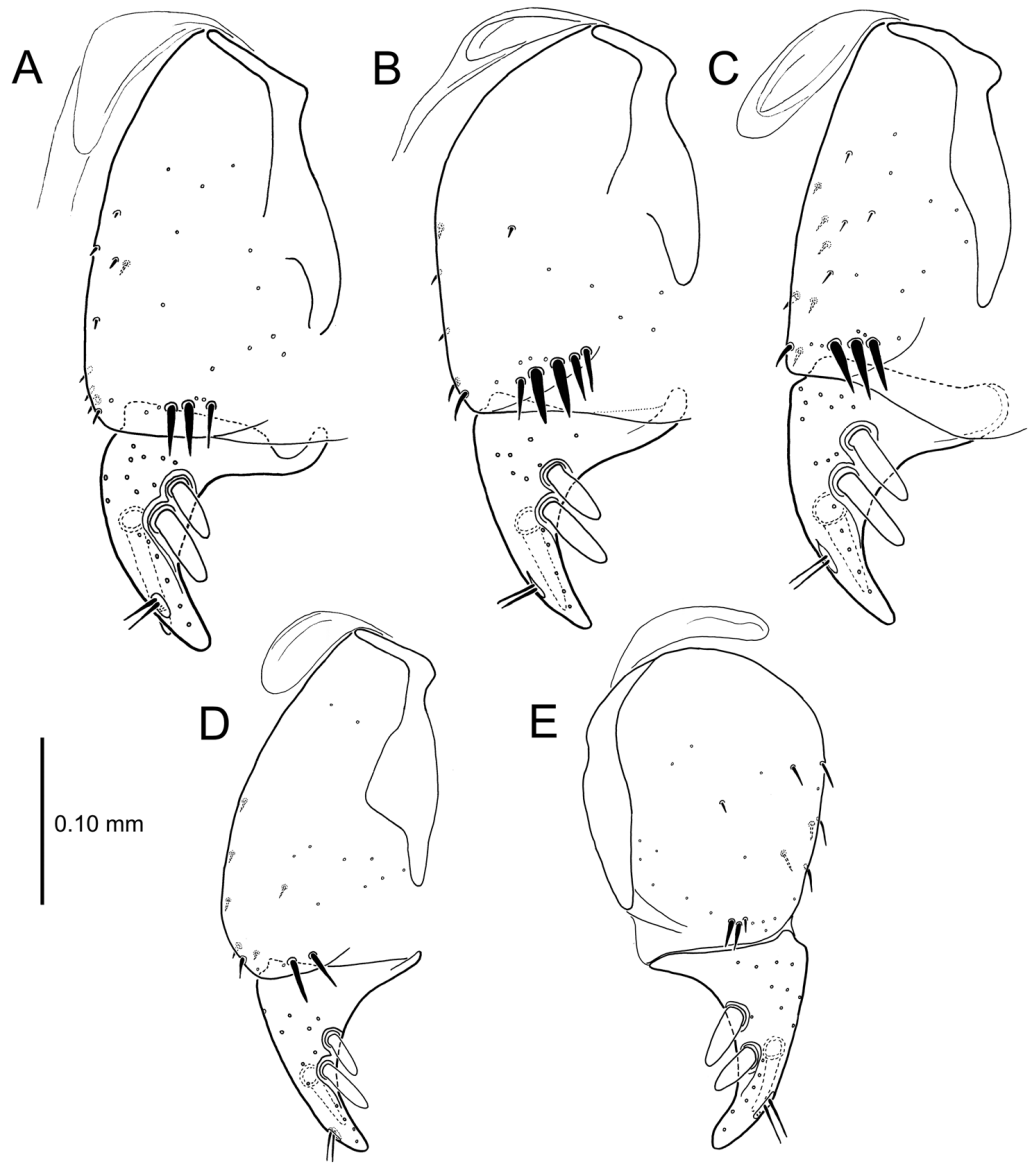
Female gonocoxa, *Mecyclothorax scaritoides* group species, ventral view. **A** *Mecyclothorax macrops*, left gonocoxa (Olinda, 1210 m) **B** *Mecyclothorax scaritoides*, left gonocoxa (nr. Ukulele Camp, 1525 m) **C** *Mecyclothorax scarites*, left gonocoxa (Olinda, 1280 m) **D** *Mecyclothorax crassuloides*, left gonocoxa (Midcamp Bog, 1665 m) **E** *Mecyclothorax crassulus*, right gonocoxa (Honomanu, 1680 m).

**Figure 99.**
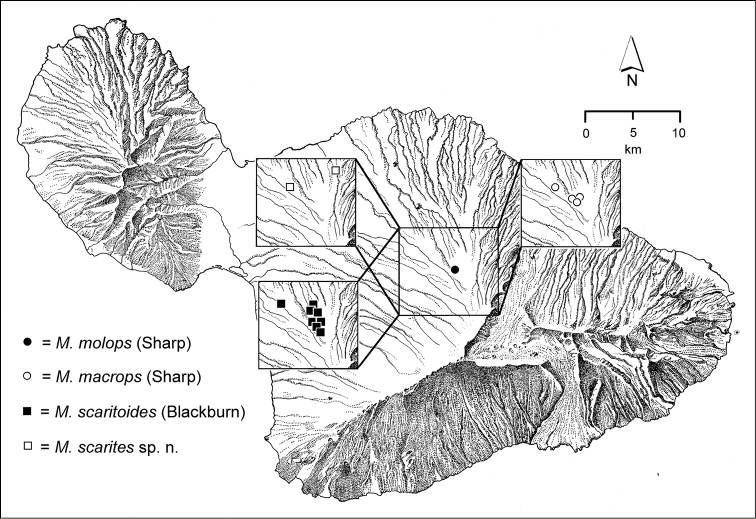
Recorded geographic distributions of *Mecyclothorax scaritoides* group species.

Figs 95B , 96A–B , 97A , 98A , 99

Metrothorax macrops Sharp 1903: 270.
Mecyclothorax macrops , Britton 1948b: 119.

##### Diagnosis.

This species (Fig. 95B) looks much like a smaller version of *Mecyclothorax molops* (Fig. 95A), but the pronotal hind angles are projected, distinctly obtuse, and the pronotal lateral margin clearly sinuate anterad the angle. The pronotal median base is more punctate, with 20 or more punctures each side that are isolated by glossy cuticle. The sutural stria is easily followed to the basal groove, though it is smooth near the elytral base. The parascutellar seta is absent. The setal formula is 1 0 2 0; anterior supraorbital seta absent. Among Haleakalā species of the group this formula is shared only with *Mecyclothorax macrops* and *Mecyclothorax scarites*. At standardized body length 6.1–6.4 mm, these beetles are diagnostically smaller than the former, and diagnostically larger than the latter.

##### Identification

(n = 4). The pronotum is vase shaped, little transverse, MPW/PL = 1.08–1.13, with the base less constricted than in *Mecyclothorax molops*; MPW/BPW = 1.53–1.65. The anterior transverse impression is deep, finely incised, with minute irregularities in the deepest portion. The anterior callosity is convex and crossed by fine longitudinal wrinkles. The elytra are slightly broader basally than in *Mecyclothorax macrops*, with the basal groove distinctly curved anterad to the subangulate humerus; MEW/HuW = 2.05–2.14. The elytral striae are very reduced apically, with only the sutural interval evident, striae 2–7 absent and traceable only by subsurface punctual remnants. The dorsal body surface bears microsculpture, if much reduced: 1, vertex with very shallow transverse mesh, sculpticell breadth 2× length; 2, pronotal disc with obsolete transverse lines in part, glossy areas between sculpticells; 3, pronotal median base glossy, with indistinct transverse cells laterally; 4, elytral disc with transverse lines irregularly joined into a loose mesh, apex with shallow transverse mesh, sculpticell breadth 2–3× length; 5, metasternum with shallow transverse mesh, sculpticell breadth 3–4× length; and 6, laterobasal abdominal ventrites with swirling isodiametric and transverse microsculpture.

**Male genitalia** (n = 1). Aedeagal median lobe elongate, gracile, distance from parameral articulation to tip 4.0× depth at midlength (Fig. 96A); apex broadly convex dorsally beyond ostial opening, tip acutely pointed, defined by juncture of convex apical face and downwardly curved ventral margin median lobe straight in ventral view, apex curved and slightly displaced to the right, with right and left margins convergent to pointed tip in this view (Fig. 96B); internal sac with lightly sclerotized macrospicules in position of dorsal ostial microtrichial patch (right side of lobe, Fig. 96B); flagellar plate of moderate size, length 0.37× parameral articulation-tip distance (estimated from shadow of sclerotized plate in ventral view, Fig. 96B).

**Female reproductive tract** (n = 1). Bursa copulatrix columnar, elongate, broadest at midlength, length 1.43 mm, midlength breadth 0.51 mm, apical breadth 0.46 mm (Fig. 97A); bursal walls translucent, thickly wrinkled; gonocoxite 1 with 3 apical fringe setae, a thick, curved seta at medioapical angle and 8–9 smaller setae along medial surface (Fig. 98A); gonocoxite 2 very falcate with narrowly rounded tip, base extended laterally in an elongate panhandle with curved terminus; 2 broad, moderately elongate lateral ensiform setae, apical nematiform setae on medioventral surface at 0.61× gonocoxite length.

##### Lectotype.

Male (BMNH) hereby designated, labeled: Metrothorax macrops Type D.S. Haleakala Maui 383 // Type // Hawaiian Is. Perkins 1904-336. // LECTOTYPE Metrothorax macrops Sharp J.K. Liebherr 1998 (black-margined red label).

##### Distribution and habitat.

*Mecyclothorax macrops* was collected by Perkins from various elevations near Ukulele Camp, however Perkins also collected a single specimen from Olinda from under the bark of a koa tree (Fig. 99), thus expanding the elevational range from 1210–1830 m. Several koa-associated Carabidae in the genus *Blackburnia* (Tribe Platynini) suffered catastrophic population declines near the turn of the 20^th^ Century, among them *Blackburnia octoocellata* (Karsch), *Blackburnia sharpi* (Blackburn), and *Blackburnia terebrata* (Blackburn) (Liebherr 2006). The suggested causes are koa logging and incursion of cattle into the Koa Forest edge—the latter compacting the soil and killing the koa roots—and invasion by alien isopods that occupied the cavities in mature koa trees that would have previously served as daytime refugia for night-foraging carabid beetles.

#### (078) Mecyclothorax scaritoides

Taxon classificationAnimaliaColeopteraCarabidae

(Blackburn)

Figs 95C , 96C , 97B , 98B , 99

Cyclothorax scaritoides Blackburn 1878b: 156; Blackburn and Sharp 1885: 215.
Metrothorax scaritoides , Sharp 1903: 272.
Mecyclothorax scaritoides , Britton 1948b: 120.
Acupalpus biseriatus Karsch 1881: 2 (new synonymy).

##### Diagnosis.

This species (Fig. 95C) shares a glabrous pronotum with three other members of the *Mecyclothorax scaritoides* group: *Mecyclothorax molops* (Fig. 95A), *Mecyclothorax macrops* (Fig. 95B), and *Mecyclothorax scarites* (Fig. 95D). Among them this species deviates by exhibiting two supraorbital setae; setal formula 2 0 2 0. The discal elytral striae are more distinctly punctate in this species, with both the sutural stria and parascutellar striole punctate—in some instances very indistinctly so—to the elytral basal groove. In beetles of the most similar species, *Mecyclothorax scarites*, the sutural stria is continued to the basal groove, but it is smooth, and the parascutellar striole is little impressed with the punctures taking the form of elongate irregularities. The elytra are also more broadly subellipsoid in this species, with the tightly rounded to subangulate humeri more proximate; MEW/HuW = 2.04–2.15 versus values of 1.93–2.0 for specimens of *Mecyclothorax scarites*. Standardized body length 5.0–5.8 mm.

##### Identification

(n = 5). The eyes are convex and large, ocular ratio = 1.50–1.53, ocular lobe ratio = 0.81–0.85. The pronotum is moderately transverse, MPW/PL = 1.16–1.25, with basally sinuate lateral margin, the hind angle obtuse with apex pointed. The pronotal median base is coplanar with the disc medially, depressed laterally, with ~20 small, isolated punctures each side. The median longitudinal impression is very shallow, traceable to obsolete, and the anterior transverse impression is broad and shallow medially, narrow and finely incised laterally. Microsculpture in this species deviates from other similar species in that the elytral disc is covered with a distinct isodiametric mesh that incorporates transverse sculpticells, breadth 2× length, and the elytral apex is covered with an isodiametric mesh in transverse rows. Microsculpture otherwise present includes: 1, vertex with obsolete transverse mesh, sculpticell breadth 2× length; 2, pronotal disc with obsolete transverse mesh, sculpticell breadth 3–4× length, most parts of cuticle glossy; 3, pronotal median base glossy, with indistinct transverse cells laterally; and 4, metasternum with obsolete transverse mesh, surface glossy.

**Male genitalia** (n = 1). Aedeagal median lobe gracile, distance from parameral articulation to tip 4.4× depth at midlength (Fig. 96C); apex broadly expanded on dorsal surface, slightly expanded ventrally, tip broadly rounded; internal sac appearing unornamented (uneverted specimen); flagellar plate moderately long, length 0.42× parameral articulation-tip distance (estimated from shadow of sclerotized plate, Fig. 96C).

**Female reproductive tract** (n = 1). Bursa copulatrix columnar with narrower apex, broader base, length 0.68 mm, apical breadth 0.15 mm, basal breadth 0.38 mm (Fig. 97B); bursal walls translucent, thinly wrinkled; gonocoxite 1 with 4–5 apical fringe setae with the medial seta smaller (Fig. 98B), a thick, curved seta at medioapical angle and 5–6 smaller setae along medial surface; gonocoxite 2 falcate with acuminate tip, base broadly extended laterally with curved terminus, 2 broad lateral ensiform setae, apical nematiform setae on medioventral surface at 0.76× gonocoxite length.

##### Lectotypes.

For *Cyclothorax scaritoides*, female (BMNH) hereby designated, labeled: mounting platen with Blackburn Maui label (Zimmerman 1957: 210), scaritoid (on reverse) // Type // Hawaiian Is. Rev. T. Blackburn 1888-30. // LECTOTYPE Cyclothorax scaritoides Blackburn J.K. Liebherr 1998 (black-margined red label). For *Acupalpus biseriatus*, female (MNHU) hereby designated, labeled: LECTOTYPE Acupalpus biseriatus Karsch det. J.K. Liebherr 2000 (black-margined red label) // Mecyclothorax scaritoides (Sharp) det. J.K. Liebherr 2000.

##### Distribution and habitat.

*Mecyclothorax scaritoides* was historically distributed along the leeward edge of the Waikamoi forest (Fig. 99). During the 1870s, Blackburn (BMNH) and Finsch (MNHU) collected specimens at elevations from 1085–1210 m near Olinda. By the time Perkins arrived in 1894, his collections were restricted to elevations of 1210–1525 m in the vicinity of Ukulele Camp. Perkins collected 187 specimens from 1894 to 1902, and nobody has seen this species in nature since. However, a closely related species, *Mecyclothorax scarites* (see below) was collected in the vicinity of Olinda from 1926–1935.

#### (079) Mecyclothorax scarites sp. n.

Taxon classificationAnimaliaColeopteraCarabidae

http://zoobank.org/3A931DB6-433E-4A1D-A333-31FE391055A6

Figs 95D , 96D–E , 97C , 98C , 99

Metrothorax perkinsianus Sharp, Swezey 1954: 53 (misidentification, *Cibotium* associate).

##### Diagnosis.

This species (Fig. 95D) is assigned to the setal formula 1 0 2 0 along with two other Haleakalā species—*Mecyclothorax molops* (Fig. 95A) and *Mecyclothorax macrops* (Fig. 95B)—yet it is more similar in pronotal configuration, overall body proportions, and elytral striation to *Mecyclothorax scaritoides* (Fig. 95C), a species characterized by presence of both supraorbital setae. In addition to the supraorbital setae, *Mecyclothorax scarites* can be distinguished from *Mecyclothorax scaritoides* by the shallow but distinct transverse microsculpture on the elytral disc that is composed of sculpticells 3–4× broad as long plus transverse lines not joined into a mesh. The discal elytral striae are not punctate, with the sutural stria irregularly impressed along its discal portion, and striae 2–4 variable impressed among individuals, but smooth to slightly irregular across the disc in all individuals. The elytra are slightly more ovoid than those of *Mecyclothorax scaritoides*, with the humeral angles more distant relative to overall elytral width; MEW/HuW = 1.93–2.0. Standardized body length 5.3–5.5 mm.

##### Description

(n = 5). *Head capsule* with frontal grooves broad, sinuous, a broad lateral convexity before eye, groove nearly joined to postocular groove anterad posterior supraorbital seta; dorsal impression of neck slightly concave; eyes moderately convex, covering much of ocular lobe, ocular ratio = 1.47–1.52, ocular lobe ratio = 0.84–0.88; labral anterior margin very shallowly emarginate medially; antennae filiform, antennomeres 1-3 glabrous; mentum tooth with sides acute, apex tightly rounded. *Pronotum* moderately transverse, MPW/PL = 1.20–1.22, base moderately constricted, MPW/BPW = 1.47–1.55, lateral margin sinuate for 0.1× pronotal length anterad obtuse hind angles, their apex rounded; median base coplanar with disc medially, ~14 small, isolated punctures each side; basal margin moderately convex between hind angles; median longitudinal impression very shallowly incised, traceable to obsolete; anterior transverse impression broad, shallow medially, narrow and finely incised laterally; anterior callosity slightly convex, smooth; front angles slightly projected, tightly rounded; pronotal apical and basal widths subequal, APW/BPW = 0.94–1.05; lateral marginal depression very narrow, edge beaded even at front angle, disc very convex; laterobasal depression smooth, convex with U-shaped groove along lateral and basal margins of convexity. *Proepisternum* with 5 minute punctures along hind marginal groove; prosternal process with very shallow depression medially. *Elytra* ovoid, greatest width slightly behind midlength; disc very convex, slightly depressed along suture, sides distinctly sloped to near vertical inside marginal depression; basal groove distinctly curved to subangulate humerus; parascutellar seta present; parascutellar striole shallow, with 3–4 elongate punctures, smooth between punctures; sutural interval slightly more convex than intervals 2–4 throughout their length; sutural stria narrow, moderately deep apically, 2^nd^ stria obsolete, traceable at apex; 8^th^ interval laterad 7^th^ stria of similar convexity to fused apical portion of striae 5 + 7; 2 dorsal elytral setae at 0.19–0.23× and 0.52–0.53× elytral length, setal impressions very small, crossing 1/3 of interval 3; lateral elytral setae arranged in anterior series of 7 setae and posterior series of 6 setae; elytral marginal depression very narrow throughout, a low bead present at humerus, a more distinct bead near subapical sinuation; subapical sinuation very shallow, less concave than width of marginal bead. *Mesepisternum* with ~10 punctures in 2–3 rows; metepisternal width to length ratio = 0.73; metepisternum/metepimeron suture distinct. *Abdomen* with irregular lateral wrinkles on ventrites 1–6, lateral depressions on ventrites 3–6; suture between ventrites 2 and 3 effaced; apical male ventrite with 2 marginal setae and apical female ventrite with 4 equally spaced setae plus median trapezoid of 4 subequal, short setae. *Legs*-metatarsomere 1/metatibial length ratio = 0.20; metatarsomere 4 length along outer lobe 1.5× medial tarsomere length, apical and subapical setae present, the lateral subapical seta short; metatarsal dorsolateral sulci broad, shallow, medially subcarinate on metatarsomere 1 only. *Microsculpture* of vertex obsolete transverse mesh, sculpticell breadth 2× length, surface glossy; pronotal disc with obsolete transverse mesh, sculpticell breadth 3–4× length, most parts glossy, median base glossy, indistinct transverse cells laterally; elytral apex with shallow transverse mesh, sculpticell breadth 2–3× length; metasternum with obsolete transverse mesh, glossy; laterobasal abdominal ventrites with swirling isodiametric and transverse microsculpture. *Coloration* of vertex rufous with a piceous cast; antennomeres 1–3 rufoflavous, 4–11 rufobrunneous; pronotal disc rufous, concolorous basally and laterally, apex slightly paler; proepipleuron pale rufobrunneous, proepisternum rufobrunneous; elytral disc rufous, sutural interval concolorous basally, rufoflavous apically; elytral marginal depression and 9^th^ interval rufoflavous apically; elytral epipleuron pale rufobrunneous, metepisternum rufobrunneous; abdomen rufobrunneous, ventrites 1–3 rufoflavous laterally, apical 1/6 of apical ventrite flavous; metafemur rufoflavous; metatibia rufoflavous with brunneous cast.

**Male genitalia** (n = 2). Aedeagal median lobe slender, distance from parameral articulation to tip 5.0× depth at midlength (Fig. 96D); apex distinctly expanded dorsally and broadly expanded ventrally producing an apical knob, the outline of the tip broadly rounded; internal sac elongate, sac length from ventral margin of ostial opening to base of flagellar plate 0.78× parameral articulation-tip distance, without ornamentation (Fig. 96E); flagellar plate very short, nearly as broad as long, length 0.24× parameral articulation-tip distance.

**Female reproductive tract** (n = 1). Bursa copulatrix columnar with narrower apex, broader base, length 0.91 mm, apical breadth 0.23 mm, basal breadth 0.41 mm (Fig. 97C); bursal walls translucent, thinly wrinkled; gonocoxite 1 with 3–4 apical fringe setae (Fig. 98C), a thick, curved seta at medioapical angle and 10–11 smaller setae along medial surface; gonocoxite 2 falcate with acuminate tip, base broadly extended laterally with curved terminus, 2 broad lateral ensiform setae, apical nematiform setae on medioventral surface at 0.66× gonocoxite length.

##### Holotype.

Male (BPBM) dissected and labeled: Kula Pipe / Line, Maui / 4200 ft. / Jan. 14, 1926 // R H VanZwal- / uwenburg // blue square // Mecyclothorax / scarites / ♂ #2 / det. J.K. Liebherr 2014 // 1 // HOLOTYPE / Mecyclothorax / scarites / Liebherr / det. J.K. Liebherr 2015 (black-margined red label).

##### Paratypes.

HI: Maui, Haleakala, Olinda, *Rubus*, 1280 m el., 27-ii-1926, Swezey (BPBM, 1), *Cheirodendron*, 1280 m el., 27-ii-1926, Swezey (BPBM, 1), *Acacia koa*, 1280 m el., 10-x-1926, Swezey (BPBM, 1; CUIC, 1); Koolau For. Res., Kula Pipeline Rd., *Cibotium menziesii*, 975–1210 m el., 27-ii-1935, Swezey (BPBM, 1).

##### Etymology.

The cylindrical body shape of *Mecyclothorax scaritoides* appears to be the basis for Blackburn (1878b) using a species epithet derived from the Greek skaritis, that stem used by Fabricius (1775) to name the large-bodied *Scarites* beetles of the Holarctic. This species is given the species epithet scarites to complete the cycle with the generic name *Scarites* F.

##### Distribution and habitat.

*Mecyclothorax scarites* is known from two localities peripheral to the known localities of *Mecyclothorax scaritoides* (Fig. 99) in the leeward forests uphill (mauka) from Olinda. Based on the careful ecological labeling of specimens by O.H. Swezey, this species is known to have occurred on koa, *Cheirodendron* (‘ōlapa), *Cibotium menziesii* (hāpu‘u), and *Rubus* (‘ākala). *Mecyclothorax scarites* is closely related yet clearly distinct from *Mecyclothorax scaritoides*. The two species were parapatrically distributed, with *Mecyclothorax scarites* appearing in the collecting record subsequent to the last appearance of any specimen of *Mecyclothorax scaritoides*. Their patchwork appearances in the scientific record are consistent with a haphazard pattern of habitat destruction along the leeward edge of the 19^th^ Century Koa-‘Ōhi‘a Mesic Forest.

#### (080) Mecyclothorax timberlakei sp. n.

Taxon classificationAnimaliaColeopteraCarabidae

http://zoobank.org/829BC0DD-75EE-4800-93DA-992842B40CE1

**Figure 100.**
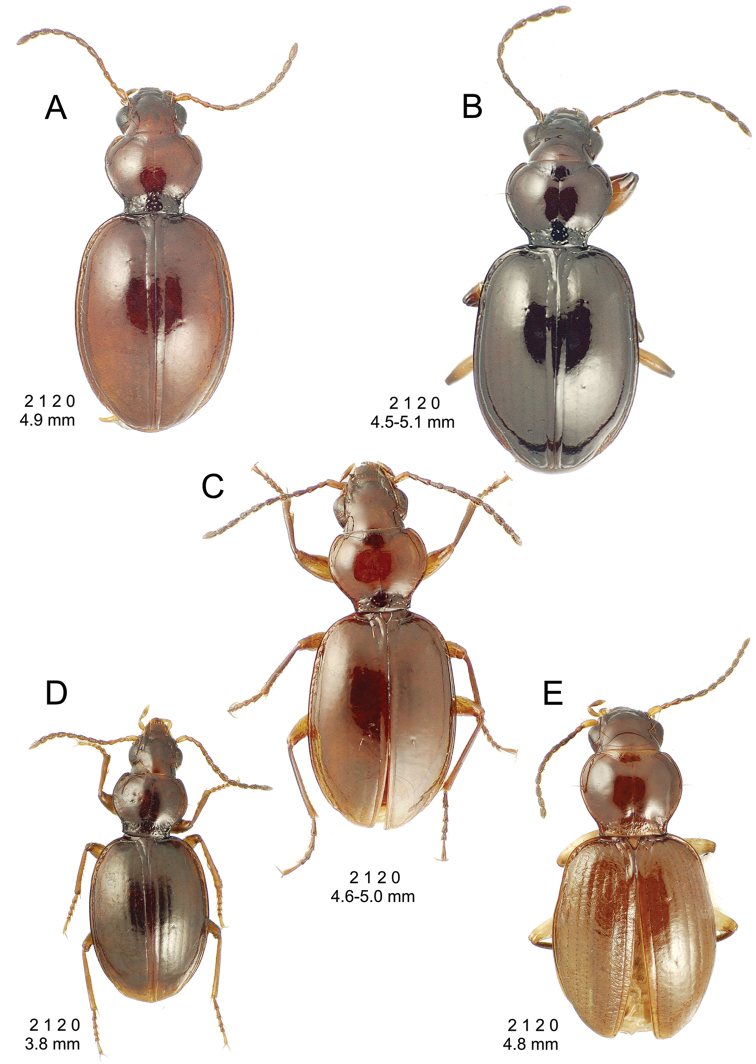
*Mecyclothorax scaritoides* group species, dorsal habitus view. **A** *Mecyclothorax timberlakei* (Ke‘anae Pali, 1525 m) **B** *Mecyclothorax crassuloides* (Kuhiwa, 880 m) **C** *Mecyclothorax crassulus* (Honomanu 1700 m) **D** *Mecyclothorax gracilicollis* (ESE Kuiki, 2090 m). **E** *Mecyclothorax dispar* (Kīpahulu, 1200 m).

**Figure 101.**
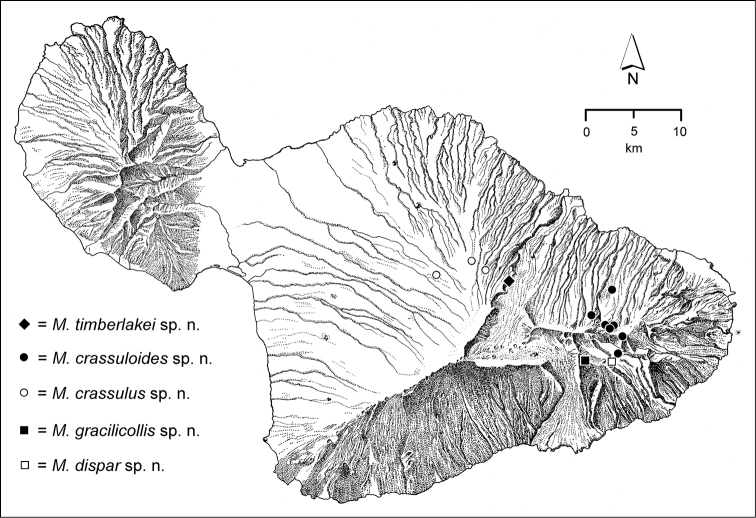
Recorded geographic distributions of *Mecyclothorax scaritoides* group species.

Figs 96F , 100A , 101

##### Diagnosis.

Of the five Haleakalā species of this group with the lateral pronotal seta present (Fig. 100), this species (Fig. 100A) can be diagnosed by the elongate convergence of the pronotal lateral margins anterad the acute hind angles. Like *Mecyclothorax crassuloides* (Fig. 100B) and *Mecyclothorax crassulus* (Fig. 100C), all discal striae save the sutural are reduced so much that they can at best be traced by very shallow serial punctures; the 8^th^ interval remains well developed, deep and broad along the lateral margin of the elytron. This species can be told from the above two by the relatively broader pronotal base; MPW/BPW = 1.63, versus values of 1.67–1.79. Setal formula 2 1 2 0. Standardized body length 4.9 mm.

##### Description

(n = 1). *Head capsule* with frontal grooves deep, directly divergent from clypeus to mesad anterior supraorbital seta, broad convexity laterally; dorsal impression of neck slightly concave; eyes large, moderately convex, ocular ratio = 1.54, ocular lobe ratio 0.85; labral anterior margin angularly emarginate medially to 1/6 of length; antennae filiform, antennomeres 2–3 with sparse pelage of short setae; mentum tooth with sides acute, apex tightly rounded. *Pronotum* slightly transverse, MPW/PL = 1.16; lateral margins convergent for 0.2× pronotal length anterad hind angles; median base depressed relative to disc, ~20 small, isolated punctures each side; basal margin straight medially, margin anteriorly curved laterad laterobasal depressions; median longitudinal impression very finely incised within slight depression of disc, crossed by indistinct transverse wrinkles; anterior transverse impression very shallow, broad, obsolete medially, smooth but crossed by minute longitudinal wrinkles; anterior callosity slightly convex, smooth, glossy; front angles moderately produced, tightly rounded; pronotal apical width slightly greater than basal width, APW/BPW = 1.05; lateral marginal depression narrow, edge upturned, slightly broader at front angle; laterobasal depression smooth, glossy surface continuous with lateral depression. *Proepisternum* with 5 minute punctures along hind marginal groove; prosternal process narrow, evenly depressed medially. *Elytra* ovoid, disc convex overall, slightly depressed along suture, distinctly sloped laterally to marginal depression; basal groove briefly, angularly curved to subangulate humerus, MEW/HuW = 2.42; parascutellar seta present; parascutellar striole brief with 3–4 punctures, deep and continuous between punctures; sutural interval convex throughout length, but crossing depressed medial portion of disc; discal intervals 2-4 and beyond laterally flat, difficult to demark; sutural stria continuous from base to apex, minutely punctate basally, smooth and deeper apically; 8^th^ interval laterad 7^th^ stria slightly more convex than fused apical portion of striae 5 + 7; 2 dorsal elytral setae at 0.20× and 0.45–0.48× elytral length, setal impressions shallow, spanning ½ width of interval 3; lateral elytral setae arranged in anterior series of 7 setae and posterior series of 6 setae; elytral marginal depression broadest at humeral angle, margin upturned in basal half, beaded near subapical sinuation; subapical sinuation shallow, more abruptly incurved anteriorly. *Mesepisternum* with ~10 large punctures in 2–3 rows; metepisternal width to length ratio = 0.68; metepisternum/metepimeron suture distinct. *Abdomen* with irregular lateral wrinkles on ventrites 1–5, lateral depressions on ventrites 3–6; suture between ventrites 2 and 3 partially effaced; apical male ventrite with 2 marginal setae. *Legs*-metatarsomere 1/metatibial length ratio = 0.20; metatarsomere 4 length along outer lobe 1.3× medial tarsomere length, apical and subapical setae present; metatarsal dorsolateral sulci broad, shallow, basal tarsomere medially subcarinate. *Microsculpture* of vertex a transverse mesh, sculpticell breadth 2–3× length; pronotal disc with shallow transverse mesh to transverse lines, the surface glossy in part, median base glossy, with indistinct transverse sculpticells laterally; elytral disc with shallow transverse mesh, sculpticell breadth 2× length, apex with such a mesh mixed with isodiametric sculpticells; metasternum with transverse mesh; laterobasal abdominal ventrites with swirling isodiametric and transverse microsculpture. Coloration of vertex rufous with a piceous cast; antennomeres 1–3 rufoflavous, 4–11 rufobrunneous; pronotal disc and margins rufous; proepipleuron rufoflavous, proepisternum rufoflavous dorsally, rufobrunneous ventrally; elytral disc and margins rufous, sutural interval concolorous basally, rufoflavous apically; elytral apex gradually paler, rufoflavous apicad subapical sinuation; elytral epipleuron and metepisternum rufoflavous; abdominal ventrites 1–3 rufobrunneous, ventrites 4–6 rufoflavous; metafemur rufoflavous; metatibia rufoflavous with brunneous cast.

**Male genitalia** (n = 1). Aedeagal median lobe gracile, distance from parameral articulation to tip 4.6× depth at midlength (Fig. 96F); apex narrowly and sinuously extended 3× its depth beyond ostial opening, the tip narrowly rounded; internal sac broad, with projected, pineapplelike lobe bearing sclerotized macrospicules at midlength on dorsal surface, and dense, brownish microspicules covering ventral surface; flagellar plate short, length 0.33× parameral articulation-tip distance.

##### Holotype.

Male (UCRC) dissected and labeled: Haleakala / Maui Jl 1919 // Keanae / Pali // above / 5000 ft. // Astelia / veratroides // Timberlake coll. // Univ. Calif. Riverside / Ent. Res. Museum / UCRC ENT 00039164 // HOLOTYPE / Mecyclothorax / timberlakei / Liebherr / det. J.K. Liebherr 2015 (black-margined red label).

##### Etymology.

This species honors the many entomological contributions of Phillip H. Timberlake, including the collection of the only known specimen of this species in 1919. Mr. Timberlake specialized in Hymenoptera, especially bees, and was an associate entomologist with the Hawaiian Sugar Planter’s Association from 1914–1924 before completing his long and distinguished career at the Citrus Experiment Station, Riverside, CA (Hurd et al. 1982).

##### Distribution and habitat.

*Mecyclothorax timberlakei* is known from a single specimen labeled “Keanae Pali, above 5000 ft.” This locality (Fig. 101) would have been most easily accessed from above timberline by walking down the western rim of Ko‘olau Gap-Ke‘anae Valley to the designated elevation. The labeled host plant substrate *Astelia veratroides* is now a junior synonym of *Astelia menziesiana* (painiu), an epiphytic lily found in mesic to wet forest.

#### (081) Mecyclothorax crassuloides sp. n.

Taxon classificationAnimaliaColeopteraCarabidae

http://zoobank.org/0B5C52B5-66DC-43ED-AFB8-5095A7FFE025

Figs 96G–I , 97D , 98D , 100B , 101

##### Diagnosis.

This species (Fig. 100B) and *Mecyclothorax crassulus* (Fig. 100C) are cryptic, sibling species, and based on synapomorphous configurations of the male aedeagal median lobes (Fig. 96G–J), they are sister species. They can be diagnosed externally by microsculpture only, with this species characterized by the elytra bearing indistinct isodiametric sculpticells in transverse rows on the disc, those sculpticells visible only outside any areas of reflected microscope light. On the elytral apex, the isodiametric sculpticells are more visible, but they are flat without upraised centers. The male aedeagal median lobe is much more robust in this species, broader dorsoventrally with a broader apex (Fig. 96I). The aedeagal internal sac is also larger, in keeping with the lobe dimensions, and more heavily spiculated. Setal formula 2 1 2 0. Standardized body length 4.5–5.1 mm.

##### Description

(n = 5). *Head capsule* with frontal grooves moderately deep, broad, the convexity laterad groove broad and low, groove terminated at short carina mesad anterior supraorbital seta; dorsal impression of neck slightly concave; eyes convex, ocular ratio = 1.54–1.58, moderately large, ocular lobe ratio = 0.74–0.80; labral anterior margin angularly emarginate 1/8 length; antennae filiform, antennomeres 2–3 with sparse pelage of short setae; mentum tooth with sides acute, apex tightly rounded. *Pronotum* moderately transverse, MPW/PL = 1.20–1.30, distinctly constricted basally, MPW/BPW = 1.67–1.80; hind angle acute, lateral margin subparallel for 0.1× pronotal length; median base depressed relative to disc, ~13 small, isolated punctures each side; basal margin straight, extended posteriorly near hind angles; median longitudinal impression very finely incised, within slight depression of disc, crossed by indistinct transverse wrinkles; anterior transverse impression broad, deep, may be crossed by distinct longitudinal wrinkles; anterior callosity slightly convex, smooth or with minute longitudinal wrinkles; front angles slightly produced, tightly rounded; apical pronotal width much greater than basal width, APW/BPW = 1.12–1.20; lateral marginal depression narrow, edge upturned, slightly broader at front angle; laterobasal depression with slightly irregular surface as on median base, continuous with lateral depression. *Proepisternum* with 5 minute punctures along hind marginal groove; prosternal process narrowly, evenly depressed medially. *Elytra* distinctly ovoid, widest behind midlength; disc slightly depressed along suture, sides distinctly sloped to marginal depression; basal groove briefly curved to subangulate humerus, the humeral angles proximate relative to elytral width, MEW/HuW = 2.31–2.43; parascutellar seta present; parascutellar striole brief, with 3–4 punctures, deep, continuous between punctures; sutural interval convex throughout length, disc depressed each side at sutural stria; discal striae 2–4 obsolete, partially traceable by very small punctulae or by cuticular pigment dots associate with strial development, lateral striae 5–7 absent (again their course visible only by serial dots of pigment associated with strial development); both 8^th^ interval laterad 7^th^ stria and fused apical portion of striae 5 + 7 slightly, broadly convex; 2 dorsal elytral setae at 0.26× and 0.54–0.65× elytral length, setal impressions very small, shallow, spanning ¼ of interval 3; lateral elytral setae arranged in anterior series of 7 setae and posterior series of 6 setae; elytral marginal depression broadest outside humerus, margin upturned in basal half, beaded near subapical sinuation; subapical sinuation shallow, more abruptly incurved anteriorly. *Mesepisternum* with ~10 large punctures in 2–3 rows; metepisternal width to length ratio = 0.63; metepisternum/metepimeron suture distinct. *Abdomen* with irregular lateral wrinkles on ventrites 1–5, lateral depressions on ventrites 3–6; suture between ventrites 2 and 3 partially effaced; apical male ventrite with 2 marginal setae and apical female ventrite with 4 equally spaced setae and median trapezoid of 4 subequal, short setae. *Legs*-metatarsomere 1/metatibial length ratio = 0.20; metatarsomere 4 length along outer lobe 1.3× medial tarsomere length, apical and subapical setae present; metatarsal dorsolateral sulci broad, shallow, basal tarsomere medially subcarinate. *Microsculpture* of vertex a transverse mesh, sculpticell breadth 2–3× length; pronotal disc with shallow transverse mesh to transverse lines, glossy in part, median base glossy with indistinct transverse sculpticells laterally; metasternum with transverse mesh; laterobasal abdominal ventrites with swirling isodiametric and transverse microsculpture. *Coloration* of vertex rufous with a piceous cast; antennomere 1 rufoflavous, 2–11 rufobrunneous in fully melanized individuals, antennomeres 1–3 rufoflavous in less melanized beetles; pronotal disc rufous, lateral margins concolorous, base and apex rufopiceous; proepipleuron rufoflavous, proepisternum rufoflavous dorsally, rufobrunneous ventrally; elytral disc rufous with a dark brunneous cast, sutural interval rufous basally, rufoflavous apically; elytral interval 9 rufopiceous, marginal depression rufoflavous; elytral apex rufoflavous only on interval 8 and along apical margin; elytral epipleuron flavous, metepisternum rufoflavous; abdomen rufoflavous; metafemur flavous with smoky brunneous apex; metatibia rufobrunneous with piceous cast.

**Male genitalia** (n = 2). Aedeagal median lobe very broad basally, attenuated apically with sinuous ventral surface, distance from parameral articulation to tip 2.4× basal depth (Fig. 96G, I), apex thick at distal end of ostial opening, sinuously narrowed to acutely pointed tip; base of median lobe nearly symmetrical in ventral outline (Fig. 96H) but left margin broadly curved 90° rightward before bluntly rounded tip in this view; internal sac with broadly convex dorsal lobe near base of sac, a suggested dorsal ostial microtrichial patch on right side at base, and ventral surface broadly covered with dark macro- and microspicules (Fig. 96I); flagellar plate elongate, length 0.50× parameral articulation-tip distance.

**Female reproductive tract** (n = 1). Bursa copulatrix columnar with rounded apex, broader base, length 1.0 mm, apical breadth 0.39 mm, basal breadth 0.48 mm (Fig. 97D); bursal walls translucent with shagreened surface and very fine wrinkles; gonocoxite 1 with 2–4 apical fringe setae, a thick, curved seta at medioapical angle and 6–8 smaller setae along medial surface (Fig. 98D); gonocoxite 2 subtriangular with subacuminate tip, 2 lateral ensiform setae, apical nematiform setae on medioventral surface at 0.78× gonocoxite length.

##### Holotype.

Female (CUIC) labeled: HI: Maui Haleakala N.P. / Kipahulu Vy. Central / Pali Tr. 1200 m el. / 29-IV-1991 sifting / moss & leaf litter // J.K. Liebherr / A.C. Medeiros, / Jr. collectors // HOLOTYPE / Mecyclothorax / crassuloides / Liebherr / det. J.K. Liebherr 2015 (black-margined red label).

##### Paratypes.

HI: Haleakala N.P., Northeast Rift, Midcamp Bog Cabin, sift *Metrosideros* humus, 1665 m el., 18-v-1993 lot 01, Liebherr/Medeiros (CUIC, 1), lot 05 Liebherr/Medeiros (CUIC, 1), New Greensword Bog, sift *Metrosideros* humus, 1850 m el., 17-v-1993 lot 05, Liebherr/Medeiros (CUIC, 1); Hana For. Res., Horseshoe Bog nr. Haleakala N.P., *Cheirodendron*/*Clermontia*, 1810 m el., 11-v-1998 lot 05, Ewing (CUIC, 1), Upper Hana, [= nr. Heleleikeoha State Fence Camp], 1730 m el., 14-xi-1973, Whittle (BPBM, 1); Koolau For. Res., Hanawi N.A.R., Kuhiwa Vy. E rim, pyrethrin fog *Metrosideros*, 880 m el., 09-vi-1999 lot 06, Polhemus (NMNH, 1), lot 09 Polhemus (NMNH, 1), pyrethrin fog steep streambank, 880 m el., 10-vi-1999 lot 01, Polhemus (NMNH, 1), Poouli Cabin, beat vegetation, 1590 m el., 05-v-1998 lot 04, Ewing (CUIC, 1), pyrethrin fog *Metrosideros*/moss, 1590 m el., 05-v-1998 lot 01, Liebherr (CUIC, 1).

##### Etymology.

The adjectival species epithet crassuloides is derived from the Latin crassus; thick, heavy (Jaeger 1955). This species and *Mecyclothorax crassulus* below are named to follow *Mecyclothorax crassus* of West Maui (Sharp 1903), a closely related species. As crassulus is the diminutive of crassus, then the epithet crassuloides used here means resembling a diminutive crassus.

##### Distribution and habitat.

*Mecyclothorax crassuloides* is distributed from Hanawī through the Hāna Bogs to Kīpahulu Valley (Fig. 101). It is known from 880–1590 m elevation in Kuhiwa Valley, and at 1200 m elevation within Kīpahulu Valley. The bog localities range in elevation 1665–1850 m. It has been collected in leaf litter, humus, and moss associated with ‘ōhi‘a, as well as associated with *Cheirodendron* (‘ōlapa), *Clermontia* (oha), and *Leptecophylla* (pūkiawe). The 11 separate collecting events of this species have each included only single specimens; unusual among Haleakalā *Mecyclothorax* spp.

#### (082) Mecyclothorax crassulus sp. n.

Taxon classificationAnimaliaColeopteraCarabidae

http://zoobank.org/0AE1AC1F-C2BC-4E97-A8AF-EE752F38D043

Figs 96J , 97E , 98E , 100C , 101

##### Diagnosis.

This adelphotaxon (Fig. 100C) to *Mecyclothorax crassuloides* (Fig. 100B) can be diagnosed externally by the elytral microsculpture, with the discal intervals covered with a shallow isodiametric mesh, and the lateral intervals with more transverse sculpticells, the microsculpture of all intervals visible in areas of reflected microscope light. The elytral apex bears a distinctly isodiametric mesh, with the sculpticell centers upraised, as opposed to the flat tiling of the isodiametric sculpticells on the elytral apex of *Mecyclothorax crassuloides*. As for this species’ sister taxon, the male aedeagal median lobe provides a certain diagnosis, with the lobe of *Mecyclothorax crassulus* more gracile throughout its length, and the apex narrower (Fig. 96J). The internal sac differs also, beings smaller and bearing less melanized spicules. Setal formula 2 1 2 0. Standardized body length 4.6–4.9 mm.

##### Description

(n = 5). As the microsculptural characters above are the only reliable external anatomical characters yet found to diagnoses these two species, the description of *Mecyclothorax crassuloides* can serve for this species as well, allowing the following substitutions involving non-diagnostic mensural and qualitative characters. *Head capsule* with eyes large, variably convex, ocular ratio = 1.47–1.62, ocular lobe ratio 0.77–0.85; labral anterior margin nearly straight; mentum tooth with sides right, apex rounded. *Pronotum* moderately transverse, MPW/PL = 1.22–1.31, distinctly constricted basally, MPW/BPW = 1.67–1.79; hind angle slightly obtuse, lateral margin subparallel for 0.12× pronotal length; median base depressed relative to disc, with ~16 small isolated punctures each side; basal margin slightly convex between hind angles, not extended posteriorly near angles; pronotal apical width nondiagnostically narrower relative to basal width in this species versus *Mecyclothorax crassulus*, APW/BPW = 1.08–1.17. *Elytra* tending to be narrower (Fig. 100C), more subellipsoid than in *Mecyclothorax crassulus* (Fig. 100B), MEW/HuW = 2.17–2.39; discal striae 2–4 often more evident, traceable as interrupted linear series of very small punctures. *Coloration* of available specimens extremely variable, ranging from sclerotized but not fully melanized individuals (as in Fig. 100C), to darker, more melanic individuals (e.g. *Mecyclothorax crassuloides* of Fig. 100B). Darker specimens share the smoky brunneous femoral apex on a flavous femoral base observed in specimens of *Mecyclothorax crassuloides*.

**Male genitalia** (n = 2). Aedeagal median lobe robust, broadest basally, distance from parameral articulation to tip 2.5× basal depth (Fig. 96J), apex elongate, sinuously extended 5× its depth to obliquely expanded tip with blunt dorsal projection, flattened apical face, and acutely rounded tip (median lobe appears much like a more slender version of that seen in *Mecyclothorax crassuloides*, Fig, 96I); internal sac with distinct field of macrospicules on right surface near base, otherwise covered with fine microspicules; flagellar plate very large, length 0.67× parameral articulation-tip distance.

**Female reproductive tract** (n = 1). Bursa copulatrix columnar with rounded apex, length 0.61 mm, breadth 0.30 mm, bursal walls translucent, thickly wrinkled (Fig. 97E); gonocoxite 1 with 2 apical fringe setae, the medial seta smaller, and 7 smaller setae along medial surface (Fig. 98E); gonocoxite 2 subtriangular with subacuminate tip, 2 lateral ensiform setae, apical nematiform setae on medioventral surface at 0.77× gonocoxite length.

##### Holotype.

Male (BPBM) labeled: Kula Pipeline / 8-25-27 Maui // O.H. Swezey / Collector // Ferns // 2 // HOLOTYPE / Mecyclothorax / crassulus / Liebherr / det. J.K. Liebherr 2015 (black-margined red label).

##### Paratypes.

HI: Maui, Haleakala, 1524 m el., 08-iii-1912, Rock (BPBM, 1); Olinda, 1207 m el., 13-vi-1918, Giffard/Fullaway (BPBM, 1); Waikamoi N.C.P., Honomanu drainage transect 3, sift litter, 1700 m el., 10-iv-1991 lot 01, Liebherr (CUIC, 2), sift moss and litter, 1680 m el., 08-v-1991 lot 07, Kavanaugh (CAS, 1).

##### Etymology.

This adjectival species epithet crassulus lies intermediate along the *Mecyclothorax crassus*-*Mecyclothorax crassuloides* linguistic cline. The adjectival crassulus is the diminutive of crassus—thick, heavy—used by Sharp (1903) for *Mecyclothorax crassus* of West Maui (Liebherr 2011).

##### Distribution and habitat.

*Mecyclothorax crassulus* is a species of the Waikamoi forest area (Fig. 101), with historically collected specimens from Olinda and Kula Pipeline (~1200 m elevation) complemented by more recent collections in the Honomanu drainage at ~1700 m elevation. Associated ecological collection data are limited to beating ferns, and sifting moss and leaf litter.

#### (083) Mecyclothorax gracilicollis sp. n.

Taxon classificationAnimaliaColeopteraCarabidae

http://zoobank.org/8CEA662E-D125-4419-8718-F95FCFDA454D

Figs 100D , 101

##### Diagnosis.

This is by far the smallest-bodied Haleakalā species in the *Mecyclothorax scaritoides* group; standardized body length 3.8 mm. The pronotum is basally constricted (Fig. 100D), MEW/BPW = 1.59, and the elytra are ellipsoid. These body proportions and size are similar to those of the O‘ahu *Mecyclothorax scaritoides* group species, *Mecyclothorax simiolus* (Blackburn) and *Mecyclothorax pelops* Liebherr (Liebherr 2009a). However, beetles of the O‘ahu species lack pronotal setae, thereby fitting setal formula 2 0 2 0, and *Mecyclothorax gracilicollis* is characterized by a bisetose pronotum and therefore this species matches the 2 1 2 0 setal formula of the other Haleakalā species in the group.

##### Description

(n = 1). *Head capsule* with frontal grooves broad near clypeus, only slightly sinuous, a broad convexity laterad groove before eye, and low carina mesad anterior supraorbital seta; dorsal impression impression of neck slightly concave concave; eyes little convex, ocular ratio = 1.39, but covering much of ocular lobe, ocular lobe ratio = 0.80; labral anterior margin angularly emarginate 1/6 labral length; antennae filiform, antennomere 2 with 1 short seta on shaft, antennomere 3 with 2 such setae; mentum tooth with sides acute, apex tightly rounded. *Pronotum* little transverse, MPW/PL = 1.19; hind angle sharp, obtuse due to curved basal margin inside hind angle, the lateral margin subparallel anterad angle; median base slightly depressed, ~18 small punctures each side; basal margin moderately convex between hind angles; median longitudinal impression shallow, narrowly defined, crossed by indistinct transverse wrinkles; anterior transverse impression very shallow, broad, obsolete medially, smooth; anterior callosity slightly convex, smooth, glossy; front angles slightly projected, tightly rounded; pronotal anterior width greater than basal width, APW/BPW = 1.05; lateral marginal depression narrow, edge upturned throughout length except beaded at front angle where depression is slightly broader; laterobasal depression with irregular surface continued from median base. *Proepisternum* with 5 minute punctures along hind marginal groove; prosternal process narrowly, evenly depressed medially. *Elytra* with disc slightly convex medially, side distinctly sloped to margin; basal groove curved to subangulate humerus, humeral angle defined by hitch in depression caused by juncture of narrow basal groove and broader marginal depression, MEW/HuW = 2. 17; parascutellar seta present; parascutellar striole with 4–5 punctures, deep, continuous between punctures; sutural interval more convex than slightly convex interval 2, sutural juncture upraised; sutural stria shallow, broad, minutely punctate basally, finely incised, deep, and smooth apically; striae 2–3 very shallow, striae 4–6 traceable and stria 7 obsolete on disc, apically striae 2–4 and 7 very shallow, incomplete, striae 5–6 obsolete; elytral intervals 2–3 slightly convex on disc, intervals 4–5 flat; 8^th^ interval laterad 7^th^ stria slightly more convex than fused apical portion of striae 5 + 7; 2 dorsal elytral setae at 0.29× and 0.56× elytral length, setal impressions very small, spanning ½ width of interval 3; lateral elytral setae arranged in anterior series of 7 setae and posterior series of 4–5 setae; elytral marginal depression broader laterad humerus to midlength, beadlike near subapical sinuation; subapical sinuation evident, more abruptly incurved anteriorly. *Mesepisternum* with ~10 punctures in 2–3 rows; metepisternal width to length ratio = 0.87; metepisternum/metepimeron suture distinct. *Abdomen* with irregular lateral wrinkles in ventrites 1–3, circular lateral depressions in ventrites 3–6; suture between ventrites 2 and 3 partially effaced; apical female ventrite with 4 equally spaced setae plus median trapezoid of 4 subequal, short setae. *Legs*-metatarsomere 1/metatibial length ratio = 0.19; metatarsomere 4 length along outer lobe 1.3× medial tarsomere length, apical and subapical setae present; metatarsal dorsolateral sulci shallow, broad, median area broad. *Microsculpture* of vertex very shallow, transverse, sculpticell breadth 2–3× length; pronotal disc with obsolete transverse lines in part, glossy between sculpticells, median base glossy, indistinct transverse cells laterally; elytral disc with regular, distinct transverse mesh, sculpticell breadth 2–4× length, apex with shallow transverse mesh, sculpticell breadth 2–4× length; metasternum with shallow transverse mesh, sculpticell breadth 3–4× length; laterobasal abdominal ventrites with swirling isodiametric and transverse microsculpture. *Coloration* of vertex rufobrunneous; antennomeres 1–3 rufoflavous, 4–11 rufobrunneous; pronotal disc rufobrunneous, lateral margins, base, and apex narrowly rufoflavous; proepipleuron rufoflavous, proepisternum rufobrunneous; elytral disc rufobrunneous with iridescent sheen, sutural interval rufoflavous basally and apically, rufous on disc; elytral lateral marginal depression narrowly rufoflavous, apex rufoflavous apicad subapical sinuation; elytral epipleuron rufoflavous, metepisternum rufobrunneous; abdominal ventrites 1–6 rufobrunneous, ventrites 2–3 slightly paler laterally, apical 1/3 of apical ventrite flavous; metafemur rufoflavous; metatibia rufobrunneous.

**Female reproductive tract.** The lone female specimen was not dissected.

##### Holotype.

Female (CUIC) labeled: HI: Maui Haleakala N.P. / Kipahulu west rim below / Kuiki sift humus ex ohia / 14-V-1993 lot 04 / el. 2090 m // J.K. Liebherr & / A.C. Medeiros / Collectors // HOLOTYPE / Mecyclothorax / gracilicollis / Liebherr / det. J.K. Liebherr 2015 (black-margined red label).

##### Etymology.

This species epithet combines the Latin adjective gracilis, slender or thin, with the Latin noun collis, or hill. The noun gracilicollis is used here to signify the basally constricted pronotum of beetles of this species.

##### Distribution and habitat.

The type specimen of *Mecyclothorax gracilicollis* was collected on the western rim of Kīpahulu Valley, 2090 m elevation (Fig. 101), in sifted humus from ‘ōhi‘a. Other *Mecyclothorax* species represented in the sample containing the type specimen are: *Mecyclothorax antaeus*, *Mecyclothorax consanguineus*, *Mecyclothorax iteratus*, *Mecyclothorax kuiki*, and *Mecyclothorax splendidus*.

#### (084) Mecyclothorax dispar sp. n.

Taxon classificationAnimaliaColeopteraCarabidae

http://zoobank.org/D0BEB167-F0CF-49D3-A455-4993237AAF53

Figs 100E , 101

##### Diagnosis.

Most similar (Fig. 100E) to the preceding *Mecyclothorax gracilicollis* (Fig. 100D) in elytral striation but larger bodied; standardized body length 4.75 mm. Also differing in the more broadly based pronotum—MPW/BPW = 1.51—and the glossy elytra with only patches of transverse-mesh microsculpture, sculpticell breadth 2–3× length, between the glossy portions. The unique holotype is slightly teneral–the left elytron failed to fully inflate–but the legs exhibit the smoky brunneous femoral apex that is shared with *Mecyclothorax crassuloides* (Fig. 100B) and *Mecyclothorax crassulus* (Fig. 100C). Setal formula 2 1 2 0.

##### Description

(n = 1). *Head capsule* with frontal grooves deep and broad near clypeus, sinuously curved toward eye and terminated mesad a thin, low carina at anterior supraorbital seta; dorsal impression of neck slightly concave; eyes moderately convex, ocular ratio = 1.47, large, covering much of ocular lobe, ocular lobe ratio = 0.88; labral anterior margin angularly emarginate medially to 1/8 of labral length; antennae filiform, antennomeres 2–3 with sparse pelage of short setae; mentum tooth with sides acute, apex rounded. *Pronotum* appearing longer than broad, but MPW/PL = 1.18; hind angle right, margin slightly rounded behind, lateral margin subparallel for 0.1× length before angle; median base slightly depressed relative to disc, ~8 isolated punctures each side and minute longitudinal wrinkles along margin; basal margin slightly convex between laterobasal depressions; median longitudinal impression very finely incised, situated in slight depression of disc, crossed by indistinct longitudinal wrinkles; anterior transverse impression broad, moderately deep, smooth, narrowly incised at front angles; anterior callosity slightly convex, smooth, glossy; front angles slightly produced, rounded; apical and basal pronotal widths equal; lateral marginal depression very narrow, edge beadlike, slightly broader at front angle; laterobasal depression with slightly irregular surface, broadest apically and narrowed to hind angle. *Proepisternum* with 5 minute punctures along hind marginal groove; prosternal process narrowly medially depressed. *Elytra* subquadrate to slightly ovoid (left elytron slightly deformed), disc slightly depressed along suture, sides distinctly sloped to lateral marginal depression; basal groove distinctly curved to angulate humerus at juncture with lateral marginal depression, MEW/HuW = 2.17; parascutellar seta present; parascutellar striole with 4 punctures, deep, continuous; sutural interval more convex than 2^nd^ interval throughout length, disc depressed at sutural interval; sutural stria broad, shallow, and smooth basally, deeper and more finely incised apically; discal striae 2–6 very shallow but traceable, associate intervals very slightly convex; 8^th^ interval laterad 7^th^ stria slightly more convex than apical fused portion of striae 5 + 7; 2 dorsal elytral setae at 0.30× and 0.59× elytral length, setal impressions very small, spanning ½ width of interval 3; lateral elytral setae arranged in anterior series of 7 setae and posterior series of 5–6 setae; elytral marginal depression broadest at humeral angle, margin upturned in basal half, beaded toward subapical sinuation; subapical sinuation deep, abruptly incurved anteriorly. *Mesepisternum* with ~9 punctures in 1–2 rows; metepisternal width to length ratio = 0.7; metepisternum/metepimeron suture distinct. *Abdomen* with irregular lateral wrinkles in ventrites 1–5, lateral depressions in ventrites 3–6; suture between ventrites 2 and 3 partially effaced; apical female ventrite with 4 equally spaced setae and median trapezoid of 4 subequal, short setae. *Legs*-metatarsomere 1/metatibial length ratio = 0.19; metatarsomere 4 length along outer lobe 1.3× medial tarsomere length, apical and subapical setae present; metatarsal dorsolateral sulci broad, shallow, median area subcarinate. *Microsculpture* of vertex an obsolete transverse mesh, sculpticell breadth 2× length, surface glossy; pronotal disc with obsolete microsculpture, surface glossy, median base glossy with indistinct transverse sculpticells laterally; elytral apex with shallow transverse mesh, sculpticell breadth 2–3× length; metasternum with shallow transverse mesh, sculpticell breadth 3–4× length; laterobasal abdominal ventrites with swirling isodiametric and transverse microsculpture. *Coloration* of vertex rufous with piceous cast (based on estimate from teneral type); antennomere 1 rufoflavous, antennomeres 2–3 rufobrunneous, 4–11 with piceous cast; pronotal disc rufous to rufobrunneous, lateral margins concolorous, base and apex rufoflavous; proepipleuron rufoflavous, proepisternum rufoflavous dorsally, rufobrunneous ventrally; elytral disc rufous with brunneous cast, sutural interval rufous basally, rufoflavous apically; elytral interval 9 rufopiceous, marginal depression rufoflavous; elytral apex rufoflavous only on interval 8 and along apical margin; elytral epipleuron flavous, metepisternum rufoflavous; abdominal ventrites 1–3 rufobrunneous, 4–6 rufoflavous, apical half of apical ventrite 6 flavous; metafemur flavous with smoky brunneous base and apex; metatibia rufobrunneous with piceous cast.

**Female reproductive tract.** The lone female specimen was not dissected.

##### Holotype.

Female (CUIC) labeled: HI: Maui Haleakala N.P. / Kipahulu Vy. Central / Pali Tr. 1200 m el. / 29-IV-1991 sifting / moss & leaf litter // J.K. Liebherr / A.C. Medeiros / Jr. collectors // HOLOTYPE / Mecyclothorax / dispar / Liebherr / det. J.K. Liebherr 2015 (black-margined red label).

##### Etymology.

The adjectival species epithet dispar means different, or unequal in Latin (Brown 1956), and is used here to represent the unusual body proportions that characterize this species.

##### Distribution and habitat.

*Mecyclothorax dispar* is known only from the type collected at 1200 m elevation along the Central Pali Trail, Kīpahulu Valley. The specimen was collected from the first set of sift samples completed by Liebherr and Medeiros in Kīpahulu Valley (Fig. 101). We collected four sift samples of about 2 l volume each, and collected beetles from the siftate as it lay spread on a beating sheet. The siftate included litter from ‘ōhi‘a and *Cibotium* (hāpu‘u), as well as moss from the drier undersides of downed logs. The litter was extremely wet from rain; e.g. 9 cm of rain fell the night after this daytime sampling (unpubl. data). Other *Mecyclothorax* spp. collected in these samples included: *Mecyclothorax aquilus*, *Mecyclothorax manducus*, *Mecyclothorax mauiae*, *Mecyclothorax ovipennis*, *Mecyclothorax pau*, *Mecyclothorax poouli*, and *Mecyclothorax simpulum*.

### *Mecyclothorax haleakalae* species group

**Figure 102.**
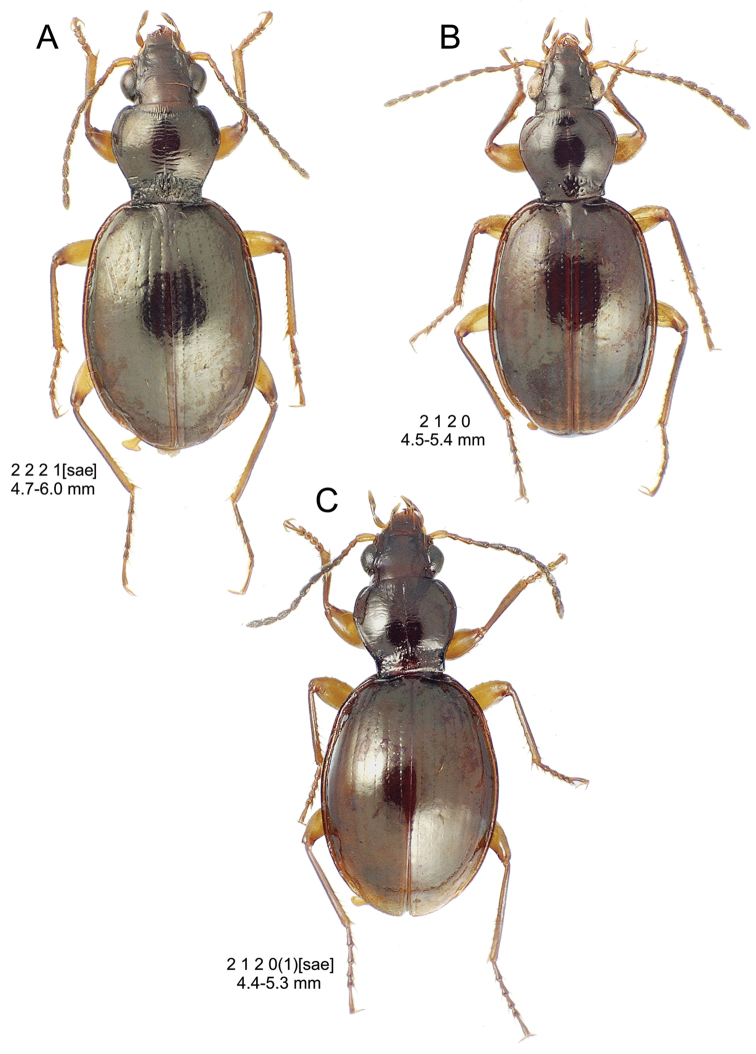
*Mecyclothorax haleakalae* group species, dorsal habitus view. **A** *Mecyclothorax iteratus* (ESE Kuiki, 2090 m) **B** *Mecyclothorax reiteratus* (Ke‘anae, 1325 m) **C** *Mecyclothorax splendidus* (ESE Kuiki, 2145 m).

**Diagnosis.** Members of this group are diagnosed by the reduced elytral striation, with discal striae reduced in depth—often only a linear series of isolated punctures—and the sutural stria of the same depth and striation as the more lateral discal striae. The eyes are very prominent, with ocular ratios spanning 1.55 to 1.71, and the eye covering much of the ocular lobe; ocular lobe ratio = 0.83–0.93. Beetles of this group are of moderate to large body size; standardized body length 4.4–6.3 mm. The elytral microsculpture is isodiametric, excepting *Mecyclothorax bacrionis* (Fig. 110A) which exhibits glossy elytra without perceivable sculpticells. That *Mecyclothorax bacrionis* belongs to the group can be shown by its lack of the anterior supraorbital seta plus its glabrous, vase-shaped pronotum (Fig. 110B), both characters shared with *Mecyclothorax haleakalae*. The six Haleakalā species may be divided into two groups of three, both centering on a species described by Sharp (1903). The first three (Fig. 102) including *Mecyclothorax iteratus* Sharp uniformly exhibit both supraorbital setae plus at least the lateral pronotal seta, with the subapical elytral seta present or absent; setal formulae 2221[sae], or 2120 (or in rare instances 2121[sae]). The pronotum is cordate, with prolonged sinuation of the lateral margins anterad the hind angles. The second three (Fig. 110) including *Mecyclothorax haleakalae* exhibit reduced setation, with the pronotum glabrous, apical and subapical elytral setae absent, and the anterior supraorbital seta absent in two of the three, and polymorphically present in the third; setal formulae 1 0 2 0 or 2(1) 0 2 0. The pronotum of individuals in this group is more vaselike, with the lateral sinuation less elongate, and the hind angles more obtuse.

**Membership and distribution.** This group is geographically restricted to the insular Maui Nui fragments of Moloka‘i, West Maui and Haleakalā, and ecologically restricted to montane rainforest. The two Moloka‘i species, *Mecyclothorax oculatus* Sharp and *Mecyclothorax dunbarorum* Liebherr (Liebherr 2007) are allied to *Mecyclothorax iteratus*, exhibiting the same setation—setal formula 2 2 2 1[sae]—and cordate pronotal shape. The two West Maui species (Liebherr 2011) separately join with each of the Haleakalā triplets; *Mecyclothorax ceteratus* Liebherr—setal formula 2 2 2 2—with the *Mecyclothorax iteratus* subgroup, and *Mecyclothorax kahalawaiae* Liebherr—setal formula 1 0 2 2—with the *Mecyclothorax haleakalae*-centered triplet.

#### Key to adults of the *Mecyclothorax haleakalae* species group, Haleakalā volcano, Maui, Hawai‘i

**Table:** 

1	Pronotum bisetose or quadrisetose, lateral seta present, basal seta present or absent; pronotal anterior transverse impression crossed by dense longitudinal strigae, the impression appearing zipperlike	**2**
1’	Pronotum glabrous, both lateral and basal setae absent; pronotal anterior impression shallow, finely to broadly incised, smooth	**4**
2(1)	Pronotum bisetose, lateral seta present, basal seta absent; elytra narrowly obovoid, humeri very narrow (Fig. 102B–C), MEW/HuW = 2.39–2.56	**3**
2’	Pronotum quadrisetose, both lateral and basal setae present; elytra obovoid, humeri moderately narrow (Fig. 102A), MEW/HuW = 2.23–2.38	(085) ***Mecyclothorax iteratus* Sharp**
3(2)	Discal elytral intervals with evident though shallow isodiametric to transverse-mesh microsculpture, sculpticell margins visible in reflected light; discal elytral intervals 2–4 flat to slightly concave, associated striae discontinuous linear series of isolated punctures; male aedeagal median lobe with dorsal hooked expansion, the apex not crossed by impression connected to ostium (Fig. 107)	(086) ***Mecyclothorax reiteratus* sp. n.**
3’	Discal elytral intervals glossy, obsolete isodiametric to transverse-mesh microsculpture visible only on portions of cuticle; discal intervals 2-4 slightly convex, at least in part, and associated striae continuously impressed though deeper at intermittent punctures; male aedeagal median lobe with expanded, rounded apex, the right face of apex incised by impression continuous with ostium (Fig. 108).. …	(087) ***Mecyclothorax splendidus* sp. n.**
4(1)	Pronotal median base densely and deeply punctate, 25–33 rounded to longitudinal punctures each side of midline (Fig. 110B–C); elytral surface duller, covered with evident isodiametric to transverse-mesh microsculpture; male aedeagal median lobe apex parallel sided, narrowed to a tightly rounded or subangulate tip (Fig. 113)	**5**
4’	Pronotal median base minutely punctate, 16 small isolated punctures each side of midline (Fig. 110A); elytra surface glossy, without evident microsculpture; male aedeagal median lobe apex with elongate, spoon-shaped dorsoventral expansion (Fig. 111)	(088) ***Mecyclothorax bacrionis* sp. n.**
5(4)	Elytra with dense isodiametric microsculpture, the sculpticells raised; male aedeagal median lobe apical face broad, flat, defining subangulate tip (Fig. 113A, C–D)	(089) ***Mecyclothorax haleakalae* (Sharp)**
5’	Elytra with shallow transverse-mesh microsculpture, sculpticells a mixture of isodiametric in transverse rows, and transverse with breadth up to 4× length; male aedeagal median lobe apex narrow, with convex apical face that defines tightly rounded tip (Fig. 113E, G)	(090) ***Mecyclothorax simpulum* sp. n.**

#### (085) Mecyclothorax iteratus

Taxon classificationAnimaliaColeopteraCarabidae

Sharp

**Figure 103.**
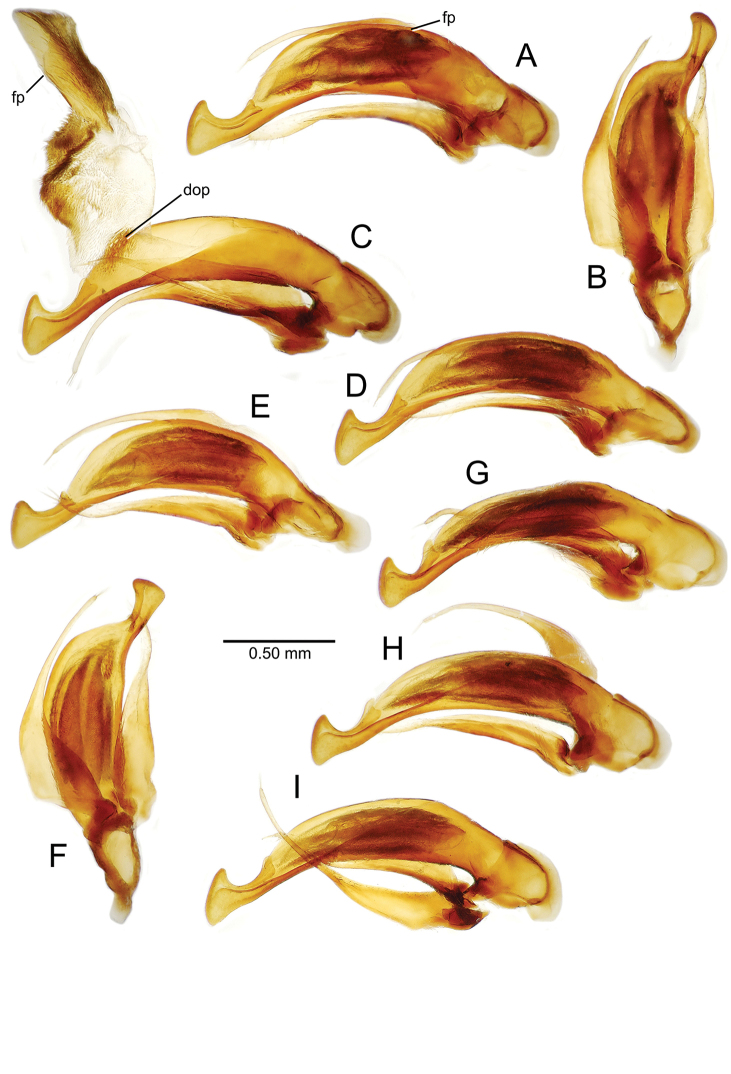
Male aedeagus, *Mecyclothorax iteratus* (for abbreviations see Table 2, p. 23). **A–B** Right and ventral views (Honomanu, 1830-1860 m) **C** Right view, sac everted (Honomanu, 1890 m) **D** Right view (Kuhiwa, 2070–2100 m) **E–F** Right and ventral views (New Greensword Bog, 1850 m) **G** Right view (Kīpahulu, 2100 m) **H–I** Right view **H** (Paliku, 1960 m). **I** (Kīpahulu W rim, 1850 m).

**Figure 104.**
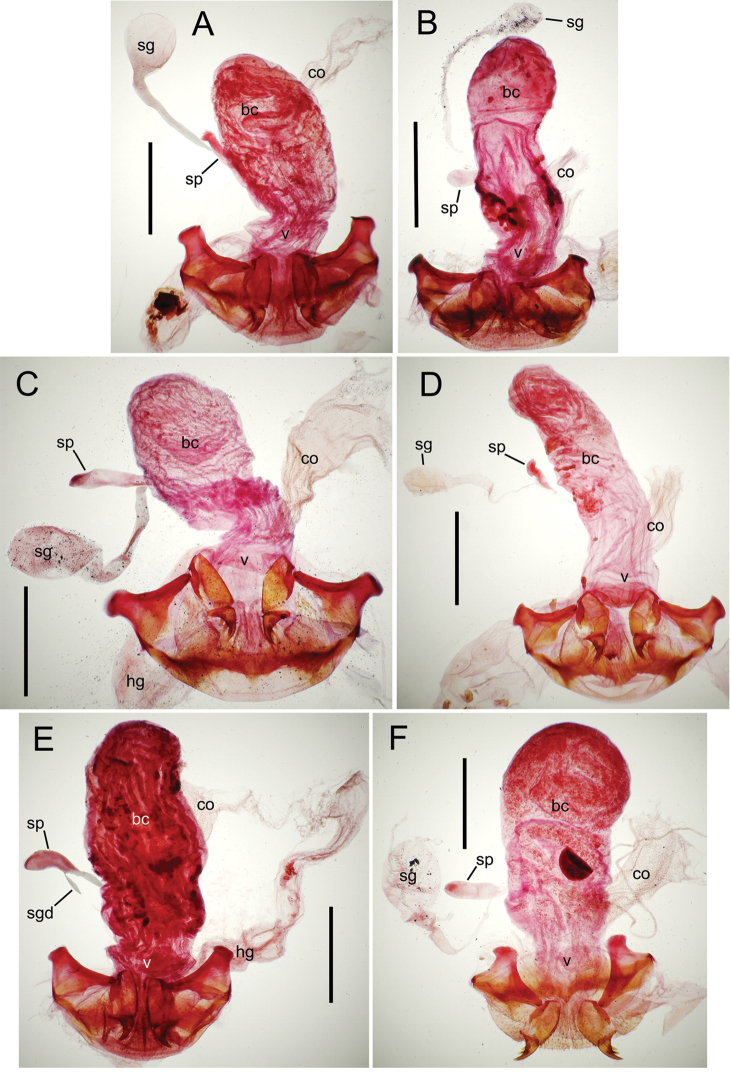
Female bursa copulatrix and associated reproductive structures, *Mecyclothorax haleakalae* group species, ventral view (for abbreviations see Table 2, p. 23). **A** *Mecyclothorax iteratus* (Kīpahulu, 2100 m) **B** *Mecyclothorax reiteratus* (Ke‘anae, 1325 m) **C** *Mecyclothorax splendidus* (New Greensword Bog, 1850 m) **D** *Mecyclothorax bacrionis* (Kuhiwa E rim, 880 m) **E** *Mecyclothorax haleakalae* (Ukulele Camp Pipeline, 1463 m) **F** *Mecyclothorax simpulum* (Kaupō Gap, 1736 m). Scale bar = 0.50 mm.

**Figure 105.**
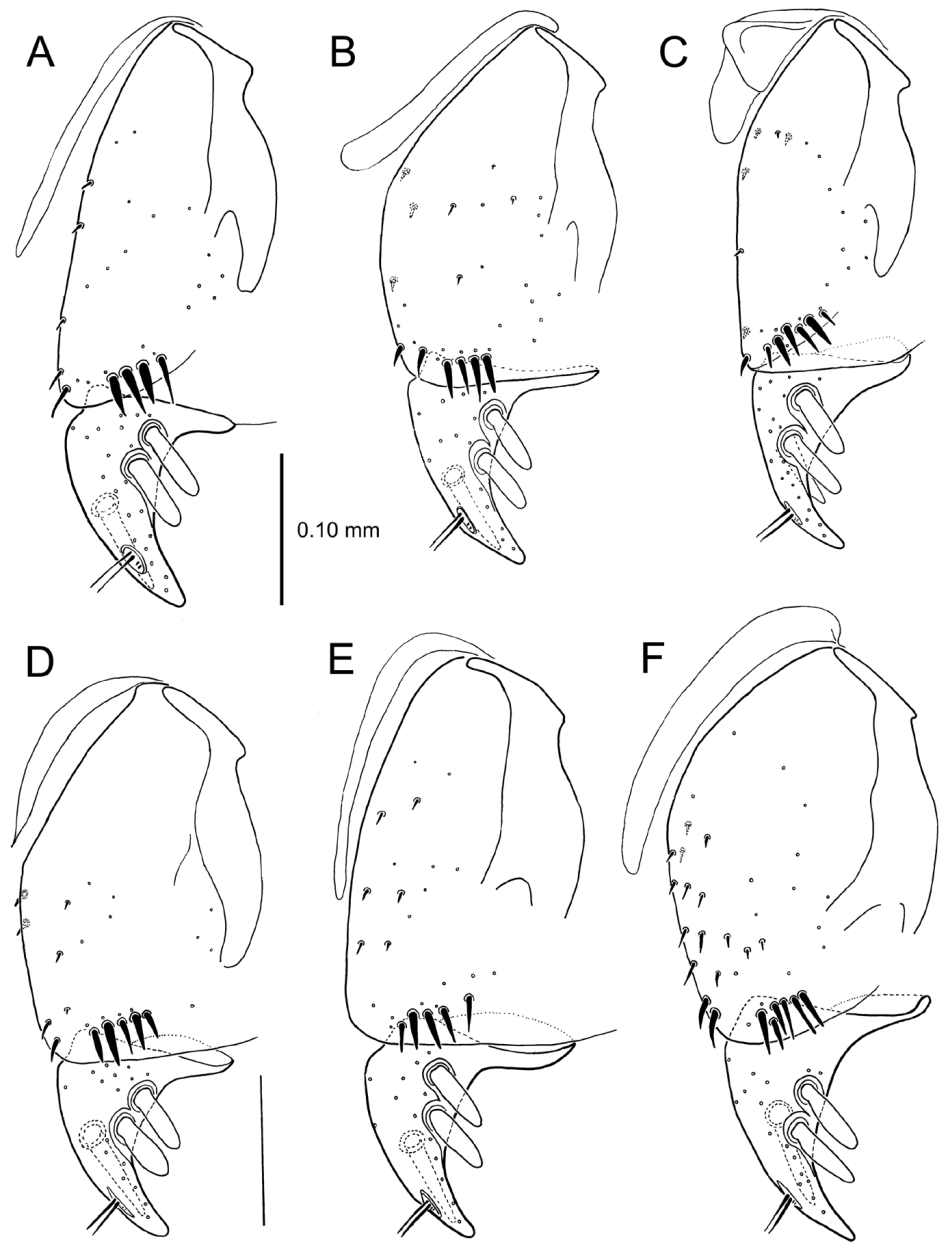
Left female gonocoxa, *Mecyclothorax haleakalae* group species, ventral view. **A** *Mecyclothorax iteratus* (Kīpahulu, 2100 m) **B** *Mecyclothorax reiteratus* (Ke‘anae, 1325 m) **C** *Mecyclothorax splendidus* (New Greensword Bog, 1850 m) **D** *Mecyclothorax bacrionis* (Kuhiwa E rim, 880 m) **E** *Mecyclothorax haleakalae* (Ukulele Camp Pipeline, 1463 m) **F** *Mecyclothorax simpulum* (Kaupō Gap, 1736 m).

**Figure 106.**
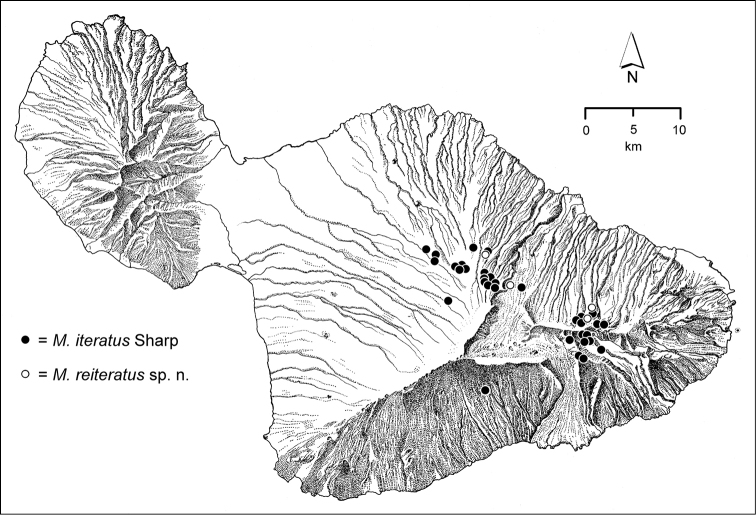
Recorded geographic distributions of *Mecyclothorax haleakalae* group species.

Figs 102A , 103 , 104A , 105A , 106

Mecyclothorax iteratus Sharp 1903: 250; Britton 1948b: 126.

##### Diagnosis.

**Figure 107.**
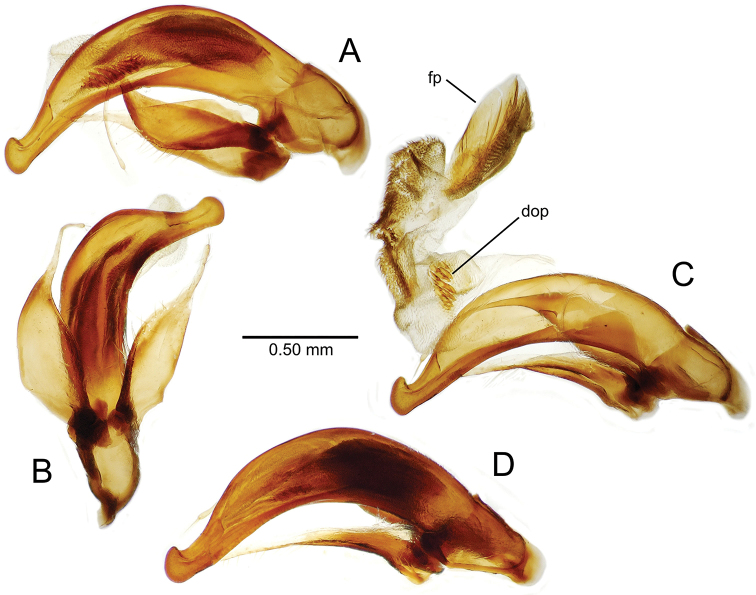
Male aedeagus, *Mecyclothorax reiteratus* (for abbreviations see Table 2, p. 23). **A–C** Right, ventral, and right with sac everted views (Ke‘anae, 1325 m) **D** Right view (Waikamoi, 1310 m).

**Figure 108.**
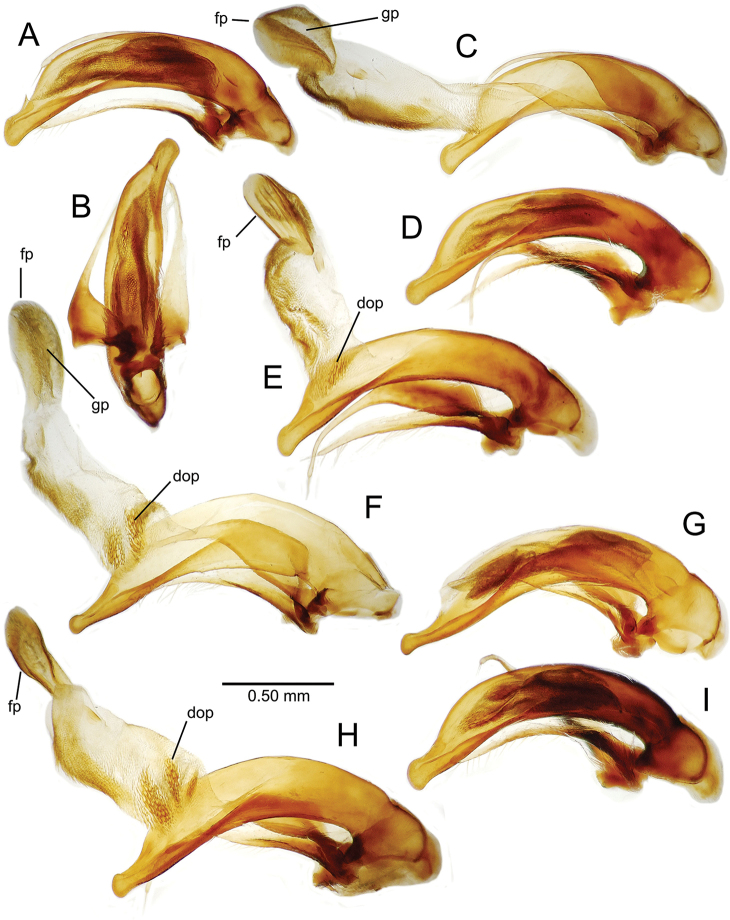
Male aedeagus, *Mecyclothorax splendidus* (for abbreviations see Table 2, p. 23). **A–B** Right and ventral views (Waikamoi, 1525 m) **C** Right view, sac everted (Honomanu, 1800 m) **D** Right view (New Greensword Bog, 1850 m) **E–F** Right view, sac everted **E** (Midcamp Bog, 1665 m) **F** (Helele‘ike‘oha, 1615 m) **G** Right view (Kīpahulu, 1200 m) **H** Right view, sac everted (Kaumakani, 1127 m) **I** Right view (ESE Kuiki, 2145 m).

The quadrisetose pronotum with deep transverse wrinkles crossing the disc and with the median base distinctly rugose (Fig. 102A) can diagnose this species from others in the group. The elytra are ovoid with narrow humeri, but the humeri are not as narrow relative to maximum elytral width as in the other two most similar species, *Mecyclothorax reiteratus* (Fig. 102B) and *Mecyclothorax splendidus* (Fig. 102C); MEW/HuW = 2.23–2.38 for this species versus values of 2.39–2.56 for measured individuals of the other two species. If the aedeagal median lobe projects even slightly from the abdominal apex of a male specimen, the broadened apex with dorsal projection (Fig. 103) can diagnose the specimen from those representing *Mecyclothorax reiteratus* (Fig. 107) or *Mecyclothorax splendidus* (Fig. 108). Setal formula 2 2 2 1[sae]. Standardized body length 4.7–6.0 mm.

##### Identification

(n = 5). The eyes are prominent and cover much of the ocular lobe, ocular lobe ratio = 0.90–0.93, but they are of variable convexity; ocular ratio = 1.54–1.74. The rugose pronotal surface involves deep, transverse discal wrinkles, a depressed, strigose median base with anastomosing punctures and elongate wrinkles and a deeply incised anterior transverse impression that is crossed by very distinct longitudinal wrinkles, the impression assuming a “zipperlike” appearance. Finally, the unique pattern of dorsal microsculpture can differentiate specimens of this species from *Mecyclothorax reiteratus* and *Mecyclothorax splendidus*; 1, vertex glossy, an indistinct transverse mesh, sculpticell breadth 2× length, in broad neck constriction; 2, pronotal disc glossy, indistinct transverse sculpticells restricted to the deep transverse wrinkles; 3, pronotal base with distinct transverse mesh, sculpticell breadth 2–3× length, between punctures and wrinkles; 4, elytral disc with regular isodiametric mesh, a few cells of transverse orientation mixed in; and 5, elytral apex with regular isodiametric mesh.

**Male genitalia** (n = 16). Aedeagal median lobe gracile, distance from parameral articulation to tip 4.7× depth at midlength (Fig. 103); apex dorsally curved beyond ostial opening, expanded dorsally as an acutely rounded tooth, the apical face slightly convex; median lobe curved rightward beyond ostial opening in ventral view, right margin narrowly concave, left margin sinuous—convex then concave—to join bluntly rounded tip (Fig. 103B); internal sac broad, with small dorsal ostial microtrichial patch on right side near base and ventral surface covered with diffuse field of melanic microspicules (Fig. 103C); flagellar plate substantial, elongate and robust, length 0.50× parameral articulation-tip distance.

**Female reproductive tract** (n = 1). Bursa copulatrix elongate, ovoid, with basal constriction distad the vagina, length 1.11 mm, maximum breadth 0.57 mm, basal constriction 0.31 mm (Fig. 104A); bursal wall translucent, thickly wrinkled; gonocoxite 1 with 3–4 apical fringe setae, a large seta at medioapical angle and 4–5 smaller setae on medial surface (Fig. 105A); gonocoxite 2 subtriangular, apex broad, base broadly, moderately extended laterally, 2 broad lateral ensiform setae with rounded apices, apical nematiform setae on medioventral surface at 0.70× gonocoxite length.

##### Lectotype.

Female (BMNH) hereby designated, labeled: Mecyclothorax iteratus Type D.S. Haleakala Perkins 354 // Type // Hawaiian Is. R.C.L. Perkins 1904 -336. // LECTOTYPE Mecyclothorax iteratus Sharp J.K. Liebherr 1998 (black-margined red label).

##### Distribution and habitat.

*Mecyclothorax iteratus* is broadly distributed around the entire forest belt of Haleakalā, with repeated collections in the Waikamoi forest, Hāna Bogs, Kīpahulu Valley, Manawainui Planeze and south face at Kahikinui (Fig. 106). Localities range 1210–2105 m elevation. Specimens have been collected on koa and ‘ōhi‘a trunks and associated mossmats, and from plant substrates including *Cheirodendron* (‘ōlapa), *Coprosma* (pilo), *Dubautia reticulata* (kupaoa), *Leptecophylla* (pūkiawe), *Myrsine* (kolea), *Rubus* (‘ākala), *Vaccinium* (‘ōhelo). It was even collected on *Deschampsia nubigena* (hairgrass) at Wai‘ele‘ele (Gagné, BPBM).

#### (086) Mecyclothorax reiteratus sp. n.

Taxon classificationAnimaliaColeopteraCarabidae

http://zoobank.org/C2276C55-5877-4460-8406-E4CBE785497C

Figs 102B , 104B , 105B , 106 , 107

##### Diagnosis.

This species (Fig. 102B) and *Mecyclothorax splendidus* (Fig. 102C) represent the only species in the group characterized by a bisetose pronotum, the lateral seta present and hind angles glabrous. In beetles of these species both supraorbital setae are present, and in this species both apical and subapical setae are absent; setal formula 2 1 2 0. The discal elytral intervals 2–4 are flat to even slightly concave, in agreement with *Mecyclothorax iteratus* but diagnostically different from *Mecyclothorax splendidus*, which exhibits slightly convex discal intervals. Elytral proportions vary among individuals, but the humeri—defined by the angle at the juncture of the basal groove and lateral marginal depression—tend to be more proximate in these beetles; MEW/HuW = 2.45–2.56 versus MEW/HuW = 2.39–2.49 in *Mecyclothorax splendidus*. The male aedeagal median lobe is very diagnostic, with males of this species exhibiting a rounded tip with a short “crochet hook” dorsal projection (Fig. 107), not the broadly expanded lobe apex of *Mecyclothorax iteratus* (Fig. 103) nor the slightly dorsoventrally expanded apex of *Mecyclothorax splendidus* (Fig. 108). Standardized body length 4.5–5.4 mm.

##### Description

(n = 5). *Head capsule* with frontal grooves slightly sinuous, broad medial face transversely wrinkled, narrow lateral carina mesad anterior supraorbital seta; dorsal impression of neck broadly concave, visible dorsally; eyes convex, ocular ratio = 1.56–1.71, covering much of protruded ocular lobe, ocular lobe ratio = 0.82–0.90; labral anterior margin shallowly emarginate to 1/7 labral length; antennae filiform, antennomeres 2–3 with sparse pelage of short setae; mentum tooth with sides acute, apex rounded, margins beaded. *Pronotum* appearing longer than broad, but MPW/PL = 1.08–1.11, base constricted, MPW/BPW = 1.53–1.62; hind angles slightly acute, sharply angled, lateral margins subparallel to convergent for 0.1× length; median base depressed relative to disc, ~25 elongate punctures each side separated by smooth cuticle; basal margin straight between hind angles; median longitudinal impression finely incised, broader on front of median base, crossed by indistinct transverse wrinkles; anterior transverse impression deeply incised, crossed by distinct longitudinal wrinkles medially, narrower and smoother mesad front angle; anterior callosity flat, crossed by dense wrinkles of bordering impression; front angles slightly projected, tightly rounded; apical pronotal width subequal to greater than basal width, APW/BPW = 0.99–1.08; lateral marginal depression very narrow, beaded laterally, slightly broader with upturned edge at front angle, upraised outside laterobasal depression; laterobasal depression an irregular continuation of lateral depression, margin beaded laterally. *Proepisternum* with 5 distinct punctures along hind marginal groove, small carinae between some punctures; prosternal process with very broad median depression, lateral margins broadly upraised. *Elytra* elongate obovoid, disc flat medially, sides distinctly sloped to margins; basal groove slightly curved to angulate humerus at juncture with lateral marginal depression, humeri proximate, lateral margins narrowly rounded, MEW/HuW = 2.45–2.56; parascutellar seta present; parascutellar striole with 5 isolated punctures, striole interrupted between punctures; sutural interval nearly coplanar with lateral intervals throughout length; sutural stria marked by proximate isolated punctures, very shallowly impressed, striae 2–5 with punctures progressively more isolated; discal intervals 2–4 flat to concave, intervals 5–8 following curvature of elytron; 7^th^ and 8^th^ intervals laterad apex of 7^th^ stria of same convexity; 2 dorsal elytral setae at 0.30–0.31× and 0.66–0.70× elytral length, setal impressions shallow, spanning ½ width of interval 3; apical and subapical setae absent; lateral elytral setae arranged in anterior series of 7 setae and posterior series of 6 setae; elytral marginal depression moderately narrow, edge upturned at humerus, depression lined with isodiametric sculpticells in anterior half, narrowed in apical 1/3 to subapical sinuation; subapical sinuation shallow, symmetrical. *Mesepisternum* with ~14 punctures in 2–3 rows; metepisternal width to length ratio = 0.75; metepisternum/metepimeron suture distinct. *Abdomen* with irregular longitudinal wrinkles laterally on ventrites 1–6, rounded lateral depressions on ventrites 3–6; suture between ventrites 2 and 3 effaced; apical male ventrite with 2 marginal setae and apical female ventrite with 4 equally spaced marginal setae and median trapezoid of 4 subequal, short setae. *Legs*-metatarsomere 1/metatibial length ratio = 0.20; metatarsomere 4 length along outer lobe 1.4× medial tarsomere length, apical and subapical setae present; metatarsal dorsolateral sulci narrow, lateral, median surface granulate. *Microsculpture* of vertex a transverse mesh, sculpticell breadth 2× length, on frons, sculpticells 3–4× broad as long on neck; pronotal disc with indistinct transverse mesh to transverse lines; pronotal median base with isodiametric sculpticells medially, transverse cells laterally; elytral disc with isodiametric mesh medially, transverse sculpticells on lateral intervals, apex with shallow isodiametric mesh; metasternum with transverse lines to transverse mesh, the sculpticell breadth 2× length; laterobasal abdominal ventrites with swirling isodiametric and transverse microsculpture. *Coloration* of vertex rufous with piceous cast in frontal grooves; antennomere 1 rufoflavous, antennomeres 2–11 rufobrunneous; pronotal disc rufopiceous, lateral margins narrowly, and base and apex broadly, rufous; proepipleuron rufoflavous, proepisternum rufobrunneous; elytral disc rufous with piceous cast, sutural interval pale rufous laterad scutellum to apex, elytral lateral margins slightly paler, rufobrunneous, apex broadly rufoflavous; elytral epipleuron rufoflavous, metepisternum rufobrunneous; abdominal ventrites 1-5 rufous, laterally and apically on ventrite 6 rufoflavous; metafemur flavous, apex with a rufobrunneous cast that matches rufobrunneous metatibia.

**Male genitalia** (n = 6). Aedeagal median lobe moderately robust, distance from parameral articulation to tip 3.4–3.8× depth at midlength (Fig. 107A, C–D); apex extended for 1.5× its depth beyond ostial opening, dorsal surface expanded into a bluntly acute tooth, surface of tip rounded apicad tooth; median lobe curved rightward nearly 90° apically in ventral view, right margin evenly concave, left margin sinuous—convex then slight concave—before rounded, slightly expanded tip (Fig. 107B); internal sac with discrete, ovoid dorsal ostial microtrichial patch comprised of macrospicules situated near sac base, and broadly diffuse field of dark microspicules covering ventral surface (Fig. 107C); flagellar plate large and robust, length 0.55× parameral articulation-tip distance.

**Female reproductive tract** (n = 1). Bursa copulatrix columnar, narrow and elongate, apex rounded, length 1.14 mm, rounded apex breadth 0.45 mm, basal breadth 0.34 mm (Fig. 104B); bursal walls translucent, thickly wrinkled; gonocoxite 1 with 3–5 apical fringe setae, a thick seta at medioapical angle and 5–6 setae along medial surface (Fig. 105B); gonocoxite 2 broadly subtriangular, tip tightly rounded, base thinly extended laterally, 2 elongate lateral ensiform setae, apical nematiform setae on medioventral surface at 0.73× gonocoxite length.

##### Holotype.

Male (CUIC) dissected and labeled: HI:East Maui Waikamoi / Flume 26-V-1997 lot06 / 1300 m el. beat ohia / in day J.K. Liebherr // Mecyclothorax / reiteratus / ♂ #21/ det. J.K. Liebherr 2014 // HOLOTYPE / Mecyclothorax / reiteratus / Liebherr / det. J.K. Liebherr 2015 (black-margined red label).

##### Paratypes.

HI: Maui: Koolau For. Res., Hanawi N.A.R., 1630 m el., vi-1997, Gruner (UHIM, 1), *Tetraplasandra dipyrena*, 1615 m el., 03-viii-1973, Gagné (BPBM, 1), Koolau Gap, Halehaku, beat ferns at night, 1325 m el., 13-v-1998 lot 09, Liebherr (CUIC, 5), beat *Metrosideros*/moss, 1325 n el., 13-v-1998 lot 11, Polhemus (NMNH, 5), beat *Rubus* (akala) at night, 1325 m el., 13-v-1998 lot 08, Liebherr (CUIC, 4), pyrethrin fog *Cibotium*/log, 1325 m el., 13-v-1998 lot 03, Liebherr (CUIC, 1), pyrethrin fog *Metrosideros*, 1325 m el., 13-v-1998 lot 04, Liebherr (CUIC, 2), pyrethrin fog *Metrosideros*/moss, 1325 m el., 13-v-1998 lot 01, Liebherr (CUIC, 3), lot 02, Liebherr (CUIC, 1), Waikamoi Gulch, Waikamoi Flume, beat/scrape *Metrosideros*, 1310 m el., 26-v-1997 lot 06, Liebherr (CUIC, 1).

##### Etymology.

Beetles of this species are similar to those of *Mecyclothorax iteratus*, and so use of the same stem plus a prefix can signify that similarity. The epithet reiteratus represents a redundancy of iteratus—to repeat—so this species repeats again a similar habitus with differences most pronounced in one setational and more male genitalic characters.

##### Distribution and habitat.

Distributional records for *Mecyclothorax reiteratus* lie at the lower elevational distributional margin of *Mecyclothorax iteratus*, with records from Waikamoi Flume, Ko‘olau Gap/Ke‘anae Valley, and Hanawī (Fig. 106). The localities span 1310–2065 m elevation. The predominant plant substrate is ‘ōhi‘a, but specimens have also been collected on *Cibotium* (hāpu‘u), *Rubus* (‘ākala), and *Polyscias* (‘ohe).

#### (087) Mecyclothorax splendidus sp. n.

Taxon classificationAnimaliaColeopteraCarabidae

http://zoobank.org/448F620A-EC1C-42BF-BB56-A92ABA2B1F8F

**Figure 109.**
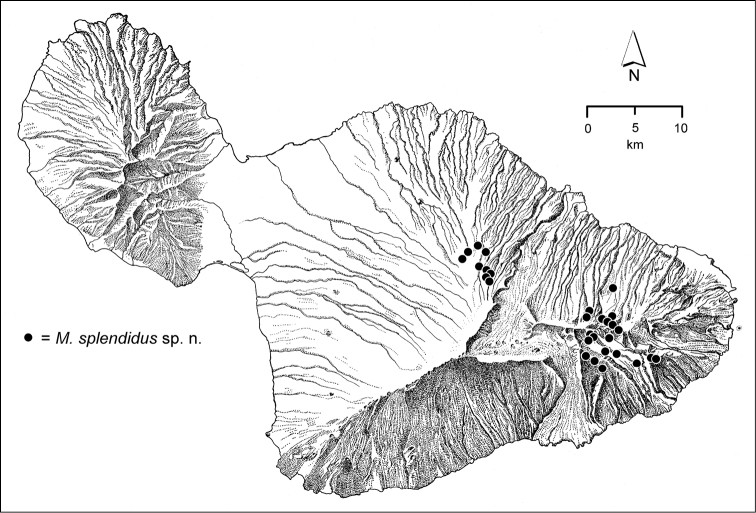
Recorded geographic distribution of *Mecyclothorax splendidus*.

Figs 102C , 104C , 105C , 108 , 109

##### Diagnosis.

Extremely similar to *Mecyclothorax reiteratus*, with bisetose pronotum and elytral humeri nearly as narrow; MEW/HuW = 2.39–2.49. The discal elytral intervals are more convex than those of *Mecyclothorax reiteratus* (Figs 102B–C), and the pronotal lateral margins are slightly more convergent before the hind angles; lateral margin convergent for 0.12× pronotal length. The dorsal microsculpture is more developed on beetles of this species, with the pronotal median base bearing a distinct isodiametric mesh across its width, and the elytral disc with a well-developed isodiametric mesh. The configuration of the male aedeagal median lobe is the best arbiter for identification, with the rounded lobe apex more or less symmetrically expanded dorsoventrally in males of this species, even given the infraspecific variation present in this structure (Fig. 108), versus a median lobe apex with a dorsal “crochet hook” expansion in males of *Mecyclothorax reiteratus* (Fig. 107). Setal formula 2 1 2 0(1)[sae]; the subapical elytral seta is most commonly absent, but present in rare instances. Standardized body length 4.4–5.3 mm.

##### Description

(n = 5). [The above description of *Mecyclothorax reiteratus* can serve to describe this species with the following substitutions.] *Head* with well-developed eyes, ocular ratio = 1.58–1.67, ocular lobe ratio = 0.84–0.90. *Pronotum* appearing longer than broad, but MPW/PL = 1.04–1.10, base moderately constricted, MPW/BPW = 1.46–1.54; median longitudinal impression finely incised, broadened on median base, crossed by moderately deep transverse wrinkles; anterior transverse impression shallow, broad, surface obscured by deep, dense longitudinal wrinkles; apical pronotal width slightly greater than basal width, APW/BPW = 1.02–1.05. *Microsculpture* of vertex an indistinct transverse mesh, sculpticell breadth 2–3× length, cuticle glossy in part; elytral apex with evident isodiametric microsculpture; metasternum with shallow transverse lines to transverse mesh, sculpticell breadth 2× length.

**Male genitalia** (n = 18). Aedeagal median lobe gracile, distance from parameral articulation to tip 4.2–4.7× depth at midlength (Fig. 108A, C–I); apex extended 2–3× depth beyond ostial opening, slightly expanded dorsoventrally at rounded tip; in ventral view, median lobe slightly curved rightward in apical half, the right and left margins convergent to rounded tip (Fig. 108B); internal sac with broad 1-part (Fig. 108E), or 2–3-part (Fig. 108F, H) dorsal ostial microtrichial patch on right side at base, a transverse band of macrospicules on left side of sac base also present in some individuals (Fig. 108H), ventral sac surface covered with shaggy pelage of microsetae; flagellar plate moderately large, length 0.40–0.44× parameral articulation-tip distance.

**Female reproductive tract** (n = 1). Bursa copulatrix columnar, elongate, apex rounded, length 1.03 mm, rounded apex breadth 0.50 mm, basal breadth 0.32 mm (Fig. 104C); bursal walls translucent, apex thinly wrinkled, base more thickly wrinkled; gonocoxite 1 with 6 apical fringe setae, a thick seta at medioapical angle and 5–6 setae along medial surface (Fig. 105C); gonocoxite 2 broadly subtriangular, tip subacuminate, base broadly extended laterally, 2 elongate lateral ensiform setae, apical nematiform setae on medioventral surface at 0.70× gonocoxite length.

##### Holotype.

Male (CUIC) dissected and labeled: HI:Maui Haleakala N.P. / Kipahulu Vy. Central / Pali Tr. 1200 m el. / 29-IV-1991 beating / vegetation at night // J.K. Liebherr / A.C. Medeiros, Jr. collectors // HOLOTYPE / Mecyclothorax / splendidus / Liebherr / det. J.K. Liebherr 2015 (black-margined red label).

##### Paratypes.

108 specimens (see Appendix).

##### Etymology.

Though hardly unique in its splendidness, this species is given the adjectival epithet splendidus to carry the flag for all species of this hyperdiverse Hawaiian radiation that evolved in splendid isolation (Simpson 1980, Zevon 1989).

##### Distribution and habitat.

*Mecyclothorax splendidus* exhibits a broad, bipartite distribution that includes the Waikamoi forest plus most of the windward face of Maui, including Hanawī, Hāna Bogs, Kīpahulu Valley, and the Manawainui Planeze (Fig. 109). Localities range from 915–2145 m elevation. Recorded forest plant substrates are predominantly ‘ōhi‘a and koa, but other recorded plants include *Cibotium menziesii* (hāpu‘u), *Dubautia* (kupoao), *Myrsine* (kolea), and *Pipturus* (māmaki). This species has been repeatedly collected by sifting humus, leaf litter, and moss.

#### (088) Mecyclothorax bacrionis sp. n.

Taxon classificationAnimaliaColeopteraCarabidae

http://zoobank.org/03F43A4E-9AE7-4E99-A804-21855D6BC9D8

**Figure 110.**
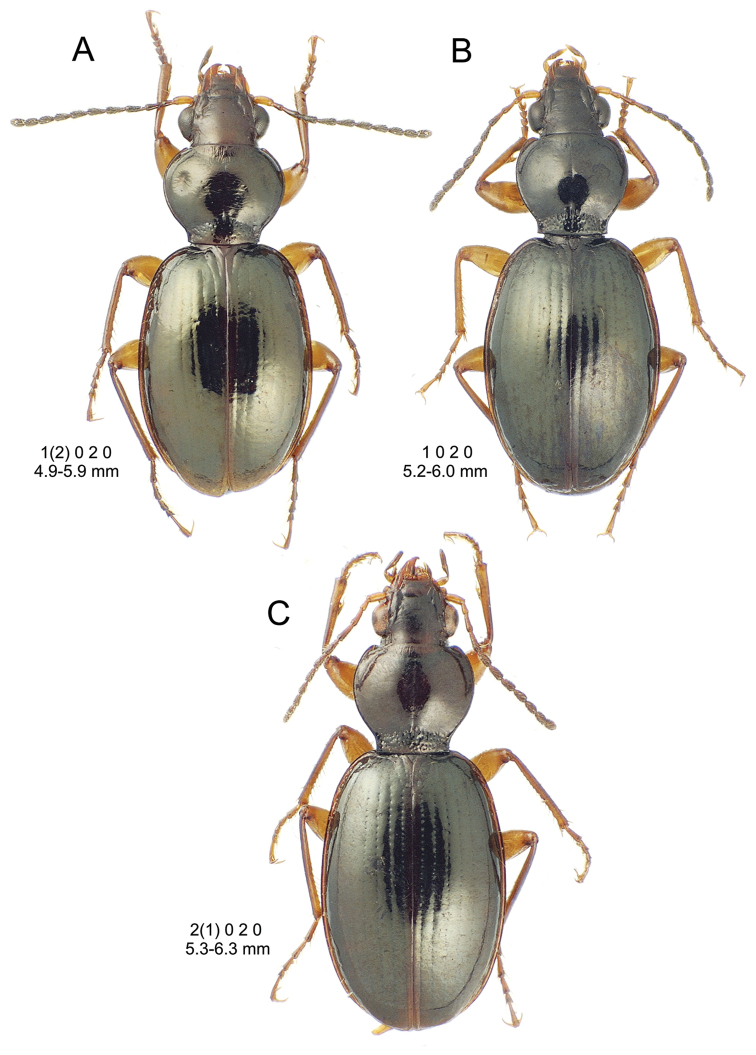
*Mecyclothorax haleakalae* group species, dorsal habitus view. **A** *Mecyclothorax bacrionis* (Ke‘anae, 1325 m) **B** *Mecyclothorax haleakalae* (Ukulele Camp Pipeline, 1463 m) **C** *Mecyclothorax simpulum* (Kaupō Gap, 1736 m).

**Figure 111.**
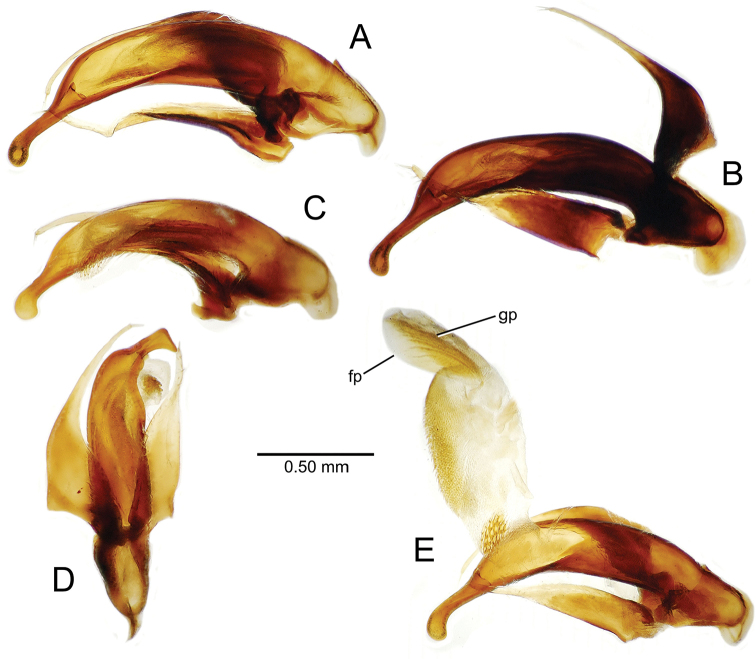
Male aedeagus, *Mecyclothorax bacrionis* (for abbreviations see Table 2, p. 23). **A–B** Right view **A** (Ke‘anae, 1325 m) **B** (Kopili‘ula, 1170 m) **C–D** Right and ventral views (Kuhiwa E rim, 910 m) **E** Right view, sac everted (Kuhiwa E rim, 915 m).

**Figure 112.**
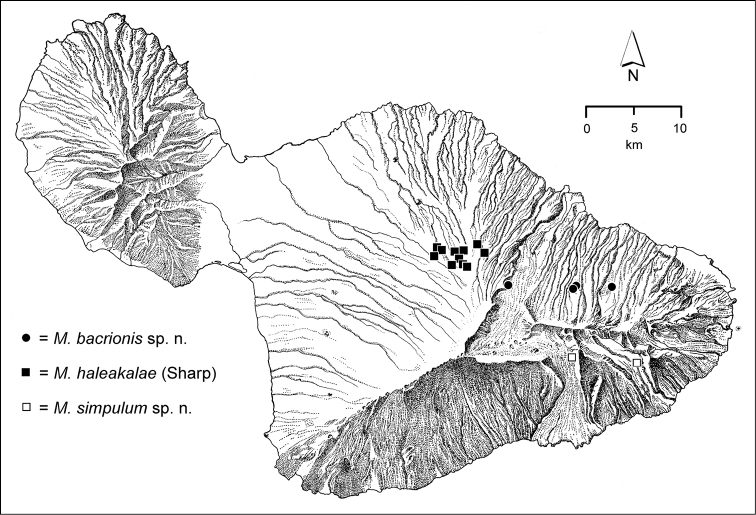
Recorded geographic distributions of *Mecyclothorax haleakalae* group species.

Figs 104D , 105D , 110A , 111 , 112

##### Diagnosis.

This species plus *Mecyclothorax haleakalae* and *Mecyclothorax simpulum* comprise the second triplet of Haleakalā’s *Mecyclothorax haleakalae* group species; this triplet characterized by the glabrous pronotum. In addition to the external key characters of: 1, minutely punctate pronotal median base; and 2, glossy elytral surface without evident microsculpture, this species (Fig. 110A) is easily diagnosed from *Mecyclothorax haleakalae* (Fig. 110B) and *Mecyclothorax simpulum* (Fig. 110C) by the foreshortened, more transverse pronotum, MPW/PL = 1.15–1.21. The elytral microsculpture is also less developed in beetles of this species, with the discal surface of the elytra glossy, the surface slightly irregular but without microsculpture. Setal formula 1(2) 0 2 0; the anterior supraorbital seta is present in rare instances. Standardized body length 4.9–5.9 mm.

##### Description

(n = 5). *Head capsule* with frontal groove sinuously curved laterally from deep juncture with clypeus to position mesad supraorbital seta; dorsal impression of neck broadly concave, visible dorsally; protruded ocular lobe largely covered by convex eye; ocular ratio = 1.51–1.61, ocular lobe ratio = 0.84–0.91; labral anterior margin shallowly emarginate to 1/8 labral length; antennae filiform, antennomeres 2–3 with extremely short setae to glabrous; mentum tooth narrow, sides acute, apex tightly rounded. *Pronotum* with basal margin slightly convex medially; median longitudinal impression very finely incised, obsolete on disc, continued deeply on front of median base; anterior transverse impression broad, shallow medially, well incised in lateral halves of each side to front angle; anterior callosity slightly convex, surface minutely irregular due to densely lined longitudinal wrinkles; front angles slightly projected, broadly expanded before tightly rounded margin; pronotal apical width greater than basal width, APW/BPW = 1.05–1.12; lateral marginal depression very narrow, beaded laterally, broader with margin upturned at front angle and outside laterobasal depression; laterobasal depression very narrow, surface irregular, continuation of lateral depression. *Proepisternum* with 5 distinct punctures along hind marginal groove, small carinae between some of the punctures; prosternal process with broad median depression, lateral margins broadly upraised. *Elytra* elongate subovoid, disc flat medially, sides distinctly sloped to margins; basal groove curved to angulate humerus defined by hitch at base of marginal depression, MEW/HuW = 2.26–2.44; parascutellar seta present; parascutellar striole with 7 isolated punctures, interrupted between punctures; sutural interval slightly elevated basally, more so apically; discal striae 1–2 shallow, punctate, striae 3–5 a series of punctures, and striae 6–7 absent, sutural stria deep and fine apically, stria 2 obsolete on apex; discal intervals slightly convex on inner intervals, flat laterally; 8^th^ interval laterad 7^th^ stria of same convexity as apical fused portion of striae 5 + 7; 2 dorsal elytral setae at 0.28–0.30× and 0.58–0.60× elytral length, setal impressions spanning ½ width of interval 3; lateral elytral setae arranged in anterior series of 7 setae and posterior series of 6 setae; elytral marginal depression moderately narrow with upturned edge at humerus, depression lined with isodiametric sculpticells in anterior half, narrowed in apical 1/3 to subapical sinuation; subapical sinuation shallow, symmetrical, internal plica visible from dorsal view. *Mesepisternum* with ~11 punctures in 2–3 rows; metepisternal width to length ratio = 0.70; metepisternum/metepimeron suture distinct. *Abdomen* with irregular longitudinal wrinkles laterally on ventrites 1–6, round lateral depressions on ventrites 4–6; suture between ventrites 2 and 3 effaced; apical male ventrite with 2 marginal setae and apical female ventrite with 4 equally spaced marginal setae plus median trapezoid of 4 subequal, short setae. *Legs*-metatarsomere 1/metatibial length ratio = 0.20; metatarsomere 4 length along outer lobe 1.3× medial tarsomere length, apical and subapical setae present; metatarsal dorsolateral sulci narrow, lateral, median surface granulate. *Microsculpture* reduced on vertex, surface glossy with indistinct transverse sculpticells in shallow depressions of the cuticle; pronotal disc glossy, indistinct transverse sculpticells over parts of the surface, median base glossy between the isolated punctures; elytral apex glossy, with indistinct transverse sculpticells along margin; metasternum with shallow transverse mesh; laterobasal abdominal ventrites with swirling isodiametric and transverse microsculpture. *Coloration* of vertex rufous with a piceous cast; antennomere 1 rufoflavous, 2–11 rufobrunneous; pronotum rufopiceous, marginal depression narrowly rufoflavous; proepipleuron rufobrunneous, proepisternum rufopiceous; elytral disc rufopiceous with cupreous reflection, sutural interval concolorous basally, rufoflavous apically; elytral marginal depression rufobrunneous, apex narrowly rufobrunneous apicad subapical sinuation; elytral epipleuron dorsally rufoflavous, ventrally rufobrunneous, metepisternum rufopiceous; abdominal ventrites 1–5 medially rufopiceous, ventrites 3–6 rufoflavous marginally, apical ventrite 6 rufoflavous in apical half; metafemur rufoflavous, apex with rufobrunneous cast; metatibia rufobrunneous.

**Male genitalia** (n = 5). Aedeagal median lobe gracile, distance from parameral articulation to tip 4.0–4.3× depth at midlength (Fig. 111A–C, E); apex elongate, extended 6× its minimum depth beyond ostial opening, terminated in a spoonlike expanded tip; median lobe apex distinctly curved rightward at 45° angle in ventral view (Fig. 111D), right margin distinctly concave, and left side slightly convex before apex which terminates in a chiseled tip in this view; internal sac with ovoid macrospicular field on right side near base, the ventral surface broadly and diffusely covered with a pelage of microspicules (Fig. 111E); flagellar plate moderately large, length 0.47× parameral articulation-tip distance.

**Female reproductive tract** (n = 1). Bursa copulatrix columnar, narrow and elongate, apex rounded, length 1.20 mm, breadth 0.34 mm (Fig. 104D); bursal walls translucent, thinly wrinkled; gonocoxite 1 with 4–5 apical fringe setae, a thicker seta at medioapical angle and 3–6 smaller setae along medial surface (Fig. 105D); gonocoxite 2 subtriangular, apex tightly rounded, base broadly extended laterally, 2 broad and elongate lateral ensiform setae, apical nematiform setae on medioventral surface at 0.75× gonocoxite length.

##### Holotype.

Male (CUIC) dissected and labeled: HI: Maui Haleakala Koolau / For. Res. Koolau Gap @ Halehaku 13-V-1998 / lot03 1325m el. pyr. fog / *Cibotium*+mossy nurse / log J.K. Liebherr // Mecyclothorax bacrionis / ♂ #4 / det. J.K. Liebherr 2014 // HOLOTYPE / Mecyclothorax / bacrionis / Liebherr / det. J.K. Liebherr 2015 (black-margined red label).

##### Paratypes.

HI: Maui: Koolau For. Res., Hanawi N.A.R., Kopiliula Str., pyrethrin fog *Acacia koa* trunk, 1127 m el., 03-v-1998 lot 02, Liebherr (CUIC, 3), pyrethrin fog *Metrosideros*/moss, 1127 m el., 03-v-1998 lot 03, Liebherr (CUIC, 1), beating *Metrosideros*, 1170 m el., 04-v-1998 lot 05, Ewing (CUIC, 1), Kuhiwa Vy. E rim, dead fronds *Cibotium chamissoi*, 900 m el., 10-vi-1999 lot 07, Ewing (CPEC, 1), pyrethrin fog *Cibotium*, 880 m el., 10-vi-1999 lot 05, Polhemus (NMNH, 3), 915 m el., 10-vi-1999 lot 02, Polhemus (NMNH, 5), lot 03, Polhemus (NMNH, 1), pyrethrin fog *Metrosideros*, 880 m el., 09-vi-1999 lot 04, Polhemus (NMNH, 1).

##### Etymology.

The species epithet bacrionis, or Latin for ladle with a long handle (Brown 1956), is here used as a noun in apposition. The epithet references the conformation of the male aedeagal median lobe (Fig. 111).

##### Distribution and habitat.

*Mecyclothorax bacrionis* is distributed across the lower elevations—880–1325 m—of Hanawī (Fig. 112). The relatively few records have been associated with ‘ōhi‘a, koa, *Cibotium* (hāpu‘u), or soft ferns.

#### (089) Mecyclothorax haleakalae

Taxon classificationAnimaliaColeopteraCarabidae

(Sharp)

**Figure 113.**
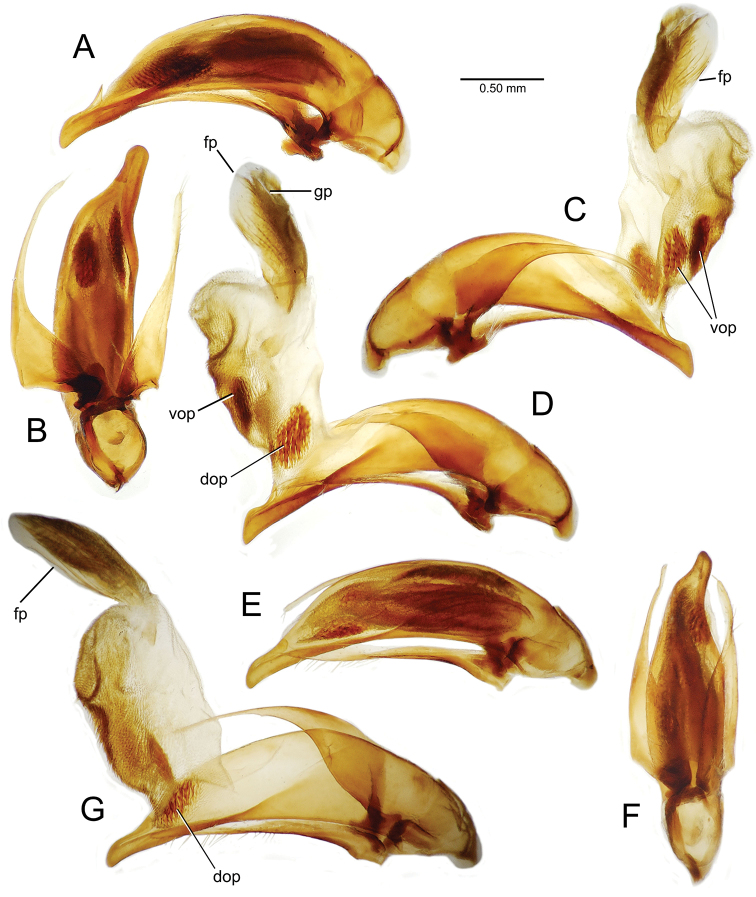
Male aedeagus, *Mecyclothorax haleakalae* group species (for abbreviations see Table 2, p. 23). **A–D** *Mecyclothorax haleakalae*. **A–B** Right and ventral views (Waikamoi, 1470 m) **C–D** Left and right views, sac everted (Ukulele Camp Pipeline, 1463 m) **E–G** *Mecyclothorax simpulum*, right, ventral, and right with sac everted views (Kaupō Gap, 1736 m).

**Figure 114.**
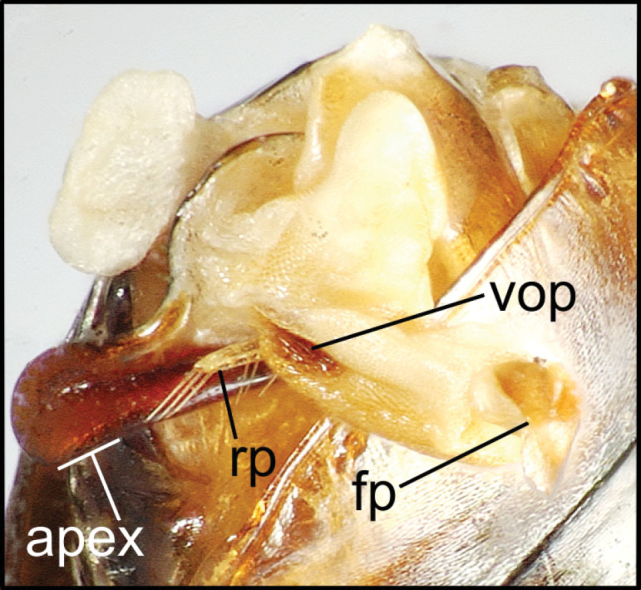
Male aedeagus of *Mecyclothorax haleakalae* in situ, ventral view, with internal sac partially everted (for abbreviations see Table 2, p. 23; apex = aedeagal apex, rp = right paramere). Aedeagus everts to left of body, and internal sac everts toward right side of aedeagal median lobe.

Figs 104E , 105E , 110B , 112 , 113A–D , 114

Metrothorax haleakalae Sharp 1903: 271; Swezey 1954: 8 (koa association).
Mecyclothorax haleakalae , Britton 1948b: 125.

##### Diagnosis.

This species (Fig. 110B) and *Mecyclothorax simpulum* (Fig. 110C) both are characterized by a glabrous quadrate pronotum—MPW/PL = 1.07–1.14 for this species—with a densely punctate median base. Specimens of *Mecyclothorax haleakalae* can be told by the well-developed and regular isodiametric mesh covering the elytral disc. The anterior supraorbital seta is absent from specimens of this species, whereas nearly all specimens examined of *Mecyclothorax simpulum* have both anterior and posterior pairs of these setae. The male aedeagal median lobe apex is broader dorsoventrally and flattened apically in *Mecyclothorax haleakalae* males (Figs 113A, C–D), and the internal sac bears two small ventral ostial microtrichial patches (Fig. 113C) which are absent from *Mecyclothorax simpulum* males (Fig. 113G). Setal formula 1 0 2 0. Standardized body length 5.2–6.0 mm.

##### Identification

(n = 5). The eyes are convex and large, ocular ratio = 1.51–1.61, ocular lobe ratio = 0.84–0.91. The pronotum is constricted basally, MPW/BPW = 1.56–1.70, with the lateral margin subparallel for 0.1× the pronotal length anterad the slightly acute, projected hind angles. The median base has ~30–33 punctures each side. The pronotum is broader apically than basally; APW/BPW = 1.11–1.16. The elytra are narrowed basally, with angulate humeri, MEW/HuW = 2.10–2.22. Discal elytral striae 1–2 are shallow and closely punctured, whereas striae 3–5 are progressively less impressed laterally, the more lateral striae indicated by linear series of isolated punctures. The associated intervals 2–4 are slightly convex to flat, and the sutural interval is upraised from the disc to the apex. The vertex is glossy, with indistinct transverse sculpticells, breadth 2× length, in small depressions. The pronotal disc is covered with an obsolete transverse microsculpture, with much of the surface glossy, and the pronotal base is glossy medially, with indistinct transverse sculpticells laterally.

**Male genitalia** (n = 5). Aedeagal median lobe robust, distance from parameral articulation to tip 3.5× depth at midlength (Fig. 113A, C–D); apex parallel sided to oblique tip formed by slightly convex apical face and slightly downturned ventral margin (Fig. 114); median lobe slightly curved rightward near apex, right margin shallowly concave, left margin sinuate to elongate, parallel-sided apex with rounded tip (Fig. 113B); internal sac broadest just before base of flagellar plate, narrow at base, with one ovoid dorsal ostial microtrichial patch on right dorsal face (Fig. 113D), and 2 distinct, ovoid ventral ostial microtrichial patches on left ventral face (Fig. 113C); flagellar plate large, robust, length 0.60× parameral articulation-tip distance (Figs 113C, G, 114).

**Female reproductive tract** (n = 1). Bursa copulatrix columnar with a basal constriction, length 1.37 mm, apical width 0.50 mm, basal constriction 0.4 mm (Fig. 104E); bursal walls opaque, thickly wrinkled; gonocoxite 1 with 5 apical fringe setae and 5–6 smaller setae on medial surface (Fig. 105E); gonocoxite 2 falcate with a broad lateral extension at base, 2 elongate lateral ensiform setae, apical nematiform setae on medioventral surface at 0.77× gonocoxite length.

##### Lectotype.

Male (BMNH) hereby designated, labeled: Metrothorax haleakalae Type D.S. Haleakala Perkins 354 // Type // Hawaiian Is. Perkins 1904-336 // LECTOTYPE Metrothorax haleakalae Sharp J.K. Liebherr 1998 (black-margined red label).

##### Distribution and habitat.

During the 19^th^ and early 20^th^ Centuries, *Mecyclothorax haleakalae* was collected across the Waikamoi forest from Olinda to Ukulele Camp at elevations 1210–1675 m (Fig. 112). More recently it has been collected at Ukulele Pipeline (1525 m el.), and in yellow-pan traps along Kula Pipeline Rd (1183–1280 m el., UHIM) and Makawao-Maile Rd. (1293–1426 m el., UHIM). Aside from the yellow-pan traps, recent records have all been on koa, with beetles found under bark, or by application of pyrethrin fog to trunks of larger trees.

#### (090) Mecyclothorax simpulum sp. n.

Taxon classificationAnimaliaColeopteraCarabidae

http://zoobank.org/9A54F54F-7663-4615-9D35-A458A19F7B45

Figs 104F , 105F , 110C , 112 , 113E–G

##### Diagnosis.

This species (Fig. 110C) can be easily diagnosed from the other two in this triplet—*Mecyclothorax bacrionis* (Fig. 110A) and *Mecyclothorax haleakalae* (Fig. 110B) by the presence of both anterior and posterior supraorbital setae (in 1 of 22 specimens, the anterior supraorbital setae is unilaterally absent; setal formula 2(1) 0 2 0). The pronotum of this species is of exceedingly similar to that of *Mecyclothorax haleakalae*, however there are fewer punctures on the median base; ~25 each side. The sutural stria is shallower also, though discal stria 2 is about as impressed as in individuals of *Mecyclothorax haleakalae*. The male aedeagal median lobe has a slightly narrower apex, assessed dorsoventrally, with a more rounded tip (Fig. 113E, G) versus the dorsoventrally broader, apically flattened median lobe apex of *Mecyclothorax haleakalae* males (Fig. 113A, C–D). The male aedeagal internal sac also lacks any discrete ventral ostial microtrichial patches (Fig. 113G), though a dorsal ostial microtichial patch is present. Standardized body length 5.3–6.3 mm.

##### Description

(n = 5). [The above description of *Mecyclothorax bacrionis* can serve to describe this species with the following substitutions.] Eyes large and convex, ocular ratio = 1.51–1.61, ocular lobe ratio = 0.84–0.91. *Pronotum* appearing elongate, but MPW/PL = 1.11–1.12, basally constricted, MPW/BPW = 1.59–1.72; hind angles slightly obtuse, apex sharp, projected; basal margin slightly convex between laterobasal depressions, margins posterad laterobasal depressions separately convex; anterior callosity slightly convex, crossed by dense though indistinct longitudinal wrinkles; pronotal apical width variably subequal to greater than basal width, APW/BPW = 1.01–1.14, laterobasal depression narrow, surface punctate, continuous with lateral depression. *Elytra* narrowly ovoid but humeral angles situated posterolaterad pronotal hind angles, therefore MEW/HuW = 2.19–2.38 (values marginally less than those recorded for *Mecyclothorax bacrionis*); discal striae 1–2 impressed, punctate, the punctures more closely spaced on sutural stria; striae 3–5 punctate but less impressed than inner striae, punctures therefore more isolated; 2 dorsal elytral setae at 0.22–0.27× and 0.66–0.69× elytral length. *Mesepisternum* with ~11 punctures in 2–3 rows. *Microsculpture* of vertex an indistinct transverse mesh, sculpticell breadth 2–3× length, surface glossy in parts; pronotal disc with indistinct transverse lines on a glossy surface, median base glossy medially, indistinct transverse sculpticells laterally; elytral disc with very small, indistinct isodiametric sculpticells in partially transverse rows, apex with shallow isodiametric mesh in transverse rows. *Coloration* of elytral disc rufopiceous, but without cupreous reflection associated with glossy elytral surface of *Mecyclothorax bacrionis*.

**Male genitalia** (n = 5). Aedeagal median lobe robust, distance from parameral articulation to tip 3.2× depth at midlength (Fig. 113E, G); apex narrowly extended 2–3× its depth beyond ostial opening, tip slightly oblique, dorsoapical face flattened, tip slightly expanded ventrally; median lobe parallel sided along shaft, right margin broadly concave before slightly offset parallel-sided apex with blunt tip (Fig. 113F); internal sac with transverse dorsal ostial microtrichial patch at base of right side (Fig. 113G), ventral surface covered with shaggy pelage of long microtrichia; flagellar plate large, robust, length 0.56× parameral articulation-tip distance.

**Female reproductive tract** (n = 1). Bursa copulatrix columnar with a rounded apical expansion, length 1.6 mm, apical lobe width 0.72 mm, basal constriction at vagina 0.36 mm broad (Fig. 104F); bursal walls translucent, thickly wrinkled; gonocoxite 1 with 5–6 apical fringe setae, 1–2 thick setae at medioapical angle and 12–14 smaller setae along medial surface (Fig. 105F); gonocoxite 2 subacuminate, lateral extension at base with curved terminus, 2 lateral ensiform setae, apical nematiform setae on medioventral surface at 0.75× gonocoxite length.

##### Holotype.

Male (CUIC) labeled: HI: Maui Haleakala N.P. / Kaupo Gap el. 1736 m / N20°42'27", W156°08'41" / 17-V-2001 lot 01 pyr. fog / *Acacia koa* J.K. Liebherr // HOLOTYPE / Mecyclothorax / simpulum / Liebherr / det. J.K. Liebherr 2015 (black-margined red label).

##### Paratypes.

HI: Maui: Haleakala N.P., Kaupo Gap, pyrethrin fog *Acacia koa*, 1736 m el., 17-v-2001 lot 01, Liebherr (CUIC, 22); Kipahulu Vy., Central Pali Tr., sift leaf/moss litter, 915 m el., 30-iv-1991 lot 03, Liebherr/Medeiros (CUIC, 1).

##### Etymology.

As in *Mecyclothorax bacrionis*, the conformation of the male aedeagal median lobe is the most reliable means to diagnose this species. Here the Latin simpulum, small ladle, is used as a noun in apposition to signify the shape of the male median lobe (Figs 113E, G).

##### Distribution and habitat.

*Mecyclothorax simpulum* is a species of koa forests in eastern Haleakalā, found in lower Kīpahulu Valley (915 m el.) and in Kaupō Gap at 1736 m elevation (Fig. 112). The Kīpahulu record was from sifted moss and leaf litter from under large koas, and the Kaupō Gap record was based on application of pyrethrin fog to the trunk of a large koa.

### *Mecyclothorax vitreus* species group

**Figure 115.**
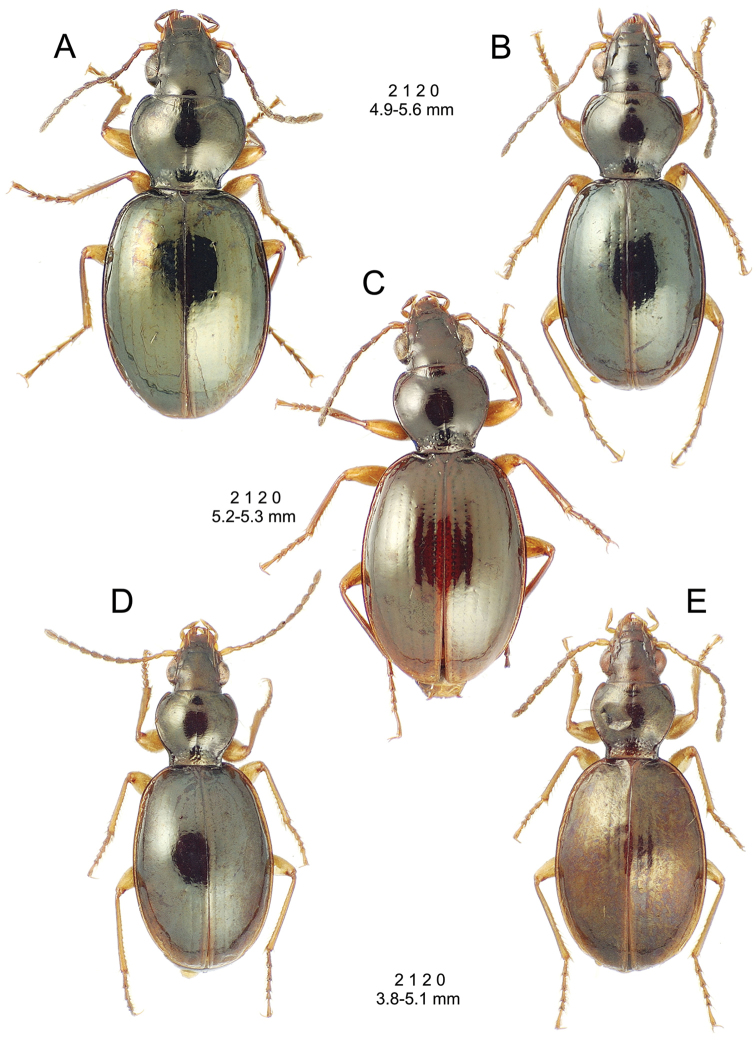
*Mecyclothorax vitreus* group species, dorsal habitus view. **A** *Mecyclothorax vitreus* (Ukulele Camp Pipeline, 1495 m) **B** *Mecyclothorax vitreus* (Waikamoi, 1420 m) **C** *Mecyclothorax perkinsianus* (“Haleakala, 1902”, RCLP) **D** *Mecyclothorax kipwilli* (Kuhiwa, 2070–2100 m) **E** *Mecyclothorax kipwilli* (Kuhiwa, 1590 m).

**Diagnosis.** Species in this group are superficially similar to those placed in the *Mecyclothorax haleakalae* group, but the dorsal microsculpture is much less developed, and the elytral striae are much less evident, with only the sutural and 2^nd^ striae impressed, at most, and the more lateral striae demarked by only very shallow impressions or undulations of the elytral surface (Figs 115, 122). The pronotum and elytral dorsal surfaces often take on a bluish metallic reflection. The eyes are less developed than in the *Mecyclothorax haleakalae* group species, ocular ratios spanning 1.46–1.59. All species in the group are characterized by presence of both supraorbital setae, two dorsal elytral setae and absence of the apical and subapical elytral setae. The pronotum may exhibit only the lateral seta, or be glabrous; setal formulae 2 1 2 0 and 2 0 2 0. The head capsule is transversely concave dorsally behind the eyes, resulting in a distinct “neck.” Standardized body length 3.8–5.6 mm.

**Membership and distribution.** This group’s diversity is centered squarely on Haleakalā, with six species occurring there. A single species, *Mecyclothorax deverilli* (Blackburn), is widespread on the Big Island of Hawai‘i (Liebherr 2008b), and *Mecyclothorax flavipes* Liebherr occurs on Lāna‘i (Liebherr 2009b). All Haleakalā species are characterized by setal formula 2 1 2 0 except *Mecyclothorax vitreus* which fits formula 2 0 2 0, that formula also fitting *Mecyclothorax deverilli* and *Mecyclothorax flavipes*.

#### Key to adults of the *Mecyclothorax vitreus* species group, Haleakalā volcano, Maui, Hawai‘i

**Table:** 

1	Vertex and elytra glossy, lacking any evident microsculpture	2
1’	Vertex and elytra with evident, transversely stretched microsculpture, sculpticell margins visible in reflected light	**3**
2(1)	Pronotum glabrous, broader, MPW/PL = 1.18–1.23; elytra ellipsoid, humeri broadly rounded, MEW/HuW = 2.12–2.30; dorsal body coloration piceous with blue reflection, head, pronotum and elytra concolorous (Fig. 115A–B)	(091) ***Mecyclothorax vitreus* Britton**
2’	Pronotum with lateral seta present, shape narrow, appearing elongate, MPW/PL = 1.08–1.10; elytra obovoid, humeri narrow, MEW/HuW = 2.42–2.53; dorsal body coloration dark brunneous to piceous, pronotum darkest (Fig. 115C)	(092) ***Mecyclothorax perkinsianus* (Sharp)**
3(1)	Elytra ellipsoid, narrow relative to forebody width, MEW/MHW = 1.87–2.18 (Fig. 122); male aedeagal median lobe apex narrow to moderately broad, tip evenly rounded or symmetrically blunted dorsoventrally (Figs 123, 125)	**4**
3’	Elytra broadly ovoid relative to width of forebody, maximum elytral width posterad midlength, MEW/MHW = 2.18–2.21 (Fig. 115D–E); male aedeagal median lobe apex very broad, apically flattened defining an angulate ventral tip (Fig. 120)	(093) ***Mecyclothorax kipwilli* sp. n.**
4(3)	Elytra with well-developed transverse microsculpture, sculpticells traceable in areas of reflected light; male aedeagal median lobe apex extended as broad or narrow, parallel-sided projection distad ostium (Figs 123F–K, 125)	**5**
4’	Elytra with shallow-margined transverse microsculpture, sculpticell margins not visible in areas of reflected light; male aedeagal median lobe apex extended as moderately short, apically rounded apex distad ostium (Fig. 123A, C–E)	(094) ***Mecyclothorax kipahulu* sp. n.**
5(4)	Pronotal base more punctate, 16–20 punctures each side of midline, pronotal basal margin smooth (Fig. 122B–C); elytra more narrowly ellipsoid, MEW/HuW = 2.10–2.27; male aedeagal median lobe apex broad, parallel sided, with slightly expanded, rounded tip, internal sac bilobate (Fig. 123F–K)	(095) ***Mecyclothorax kaumakani* sp. n.**
5’	Pronotal base less punctate medially, ~10–13 punctures each side of midline, basal margin lined with short longitudinal wrinkles (Fig. 122D–E); elytra more broadly ellipsoid, MEW/HuW = 2.30–2.39; male aedeagal median lobe apex narrow dorsoventrally, parallel sided, with tightly rounded, slightly downturned tip, internal sac unilobate (Fig. 125)	(096) ***Mecyclothorax kuiki* sp. n.**

#### (091) Mecyclothorax vitreus

Taxon classificationAnimaliaColeopteraCarabidae

Britton

**Figure 116.**
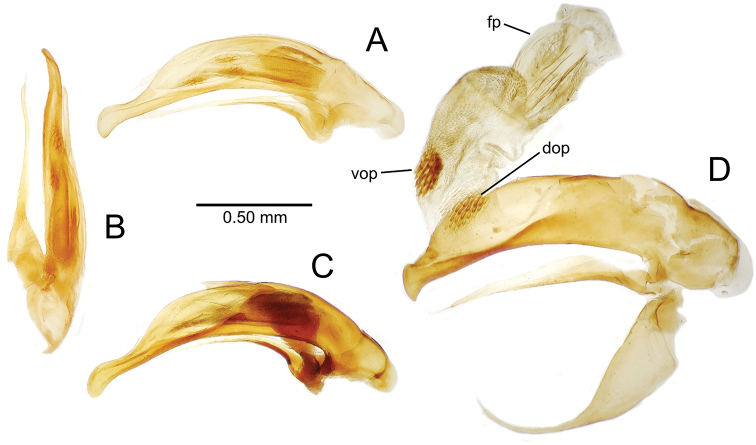
Male aedeagus, *Mecyclothorax vitreus* group species (for abbreviations see Table 2, p. 23). **A–B** *Mecyclothorax vitreus*, right and ventral views (Waikamoi, 1210 m) **C** *Mecyclothorax vitreus*, right view (Waikamoi, 1470 m) **D** *Mecyclothorax perkinsianus* (“Haleakala, 1902”, RCLP).

**Figure 117.**
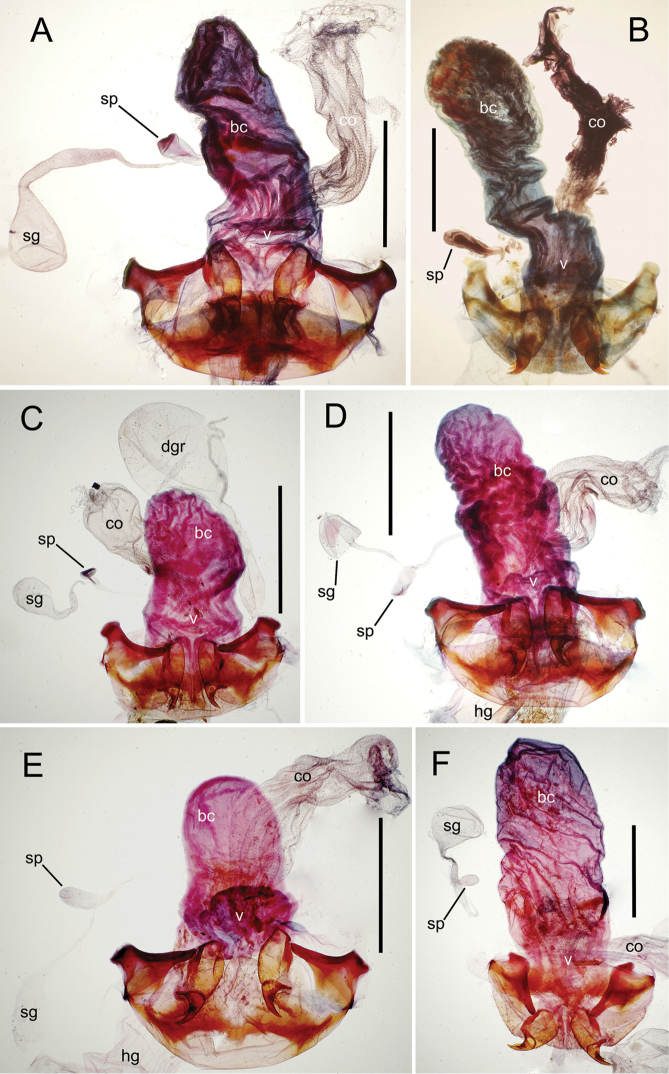
Female bursa copulatrix and associated reproductive structures, *Mecyclothorax vitreus* group species, ventral view (for abbreviations see Table 2, p. 23). **A** *Mecyclothorax vitreus* (Ukulele Camp Pipeline, 1495 m) **B** *Mecyclothorax perkinsianus* (“Haleakala, 1902”, RCLP) **C** *Mecyclothorax kipwilli* (Kuhiwa, 1590 m) **D** *Mecyclothorax kipahulu* (Kīpahulu, 1900 m) **E** *Mecyclothorax kaumakani* (Kīpahulu W rim, 1850 m) **F** *Mecyclothorax kuiki* (ESE Kuiki, 2105 m). Scale bar = 0.50 mm.

**Figure 118.**
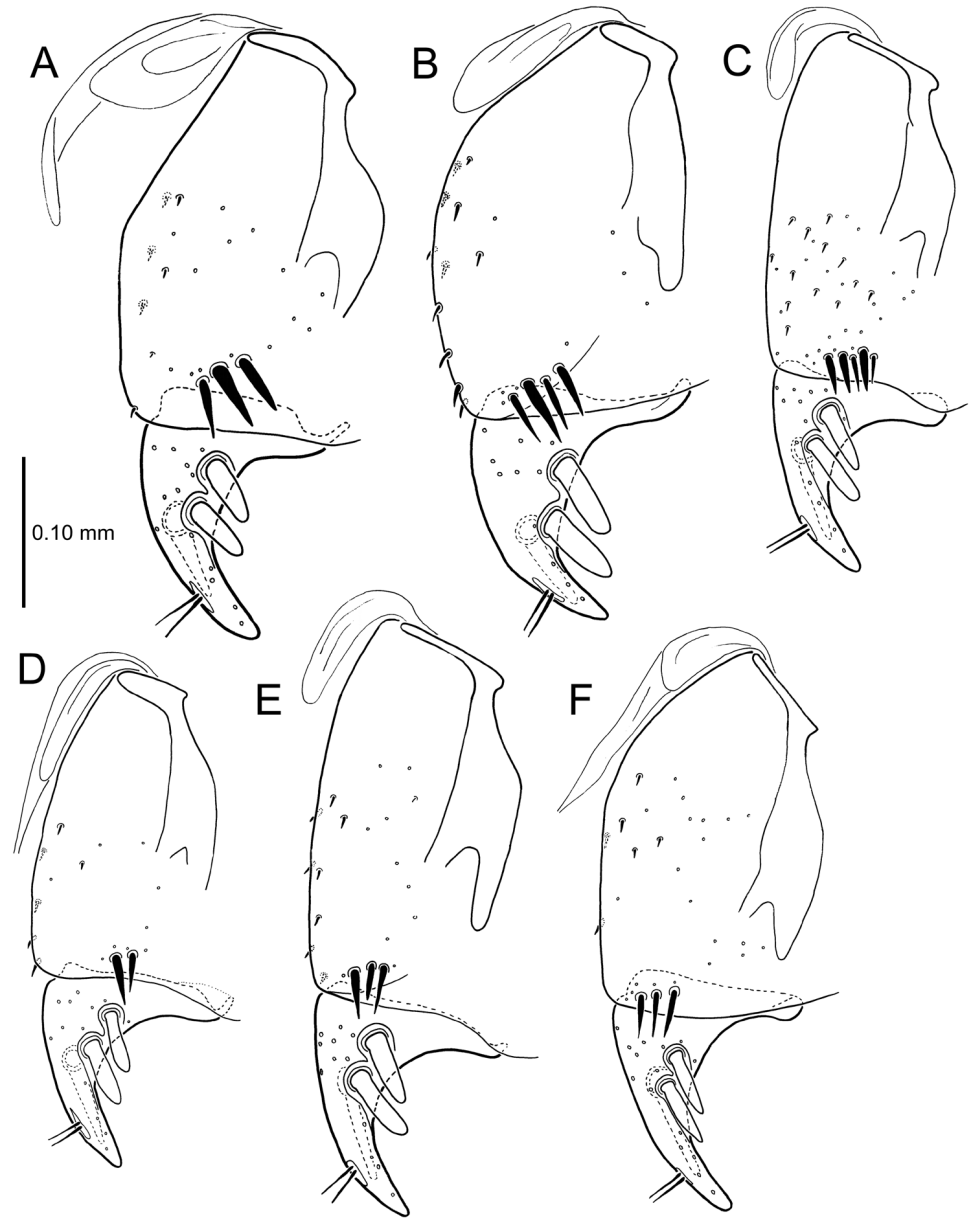
Left female gonocoxa, *Mecyclothorax vitreus* group species, ventral view. **A** *Mecyclothorax vitreus* (Ukulele Camp Pipeline, 1495 m) **B** *Mecyclothorax perkinsianus* (“Haleakala, 1902”, RCLP) **C** *Mecyclothorax kipwilli* (Kuhiwa, 1590 m) **D** *Mecyclothorax kipahulu* (Kīpahulu, 1900 m) **E** *Mecyclothorax kaumakani* (Kīpahulu W rim, 1850 m) **F** *Mecyclothorax kuiki* (ESE Kuiki, 2105 m).

**Figure 119.**
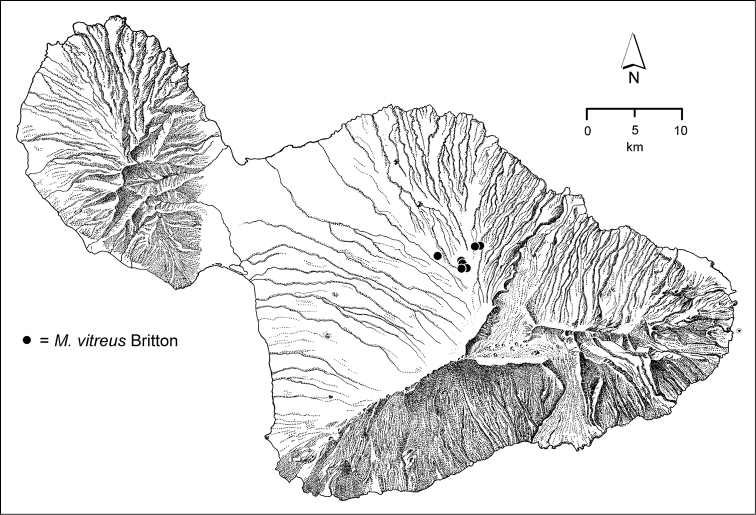
Recorded geographic distribution of *Mecyclothorax vitreus*.

Figs 115A–B , 116A–C , 117A , 118A , 119

Metrothorax laticollis Sharp 1903: 271 (junior homonym of *Mecyclothorax laticollis*Sloane 1900: 565).
Mecyclothorax vitreus Britton 1948b: 134.

##### Diagnosis.

Beetles of this species display the most vivid bluish metallic reflection of any species in the group (Fig. 115A–B). That characteristic in combination with the more transverse, glabrous pronotum, MPW/PL = 1.18–1.23 assures facile identification even in the field. The elytra vary in shape, with the humeral area broader (Fig. 115A) or narrower (Fig. 115B); MEW/HuW = 2.12–2.30. Setal formula 2 0 2 0. Standardized body length 4.9–5.6 mm.

##### Identification

(n = 5). The eyes are convex, ocular ratio = 1.46–1.57, and cover much of the protruded ocular lobe, ocular lobe ratio = 0.81–0.87. The pronotal hind angles are right to slightly obtuse, with the margin behind the angles convex, and the lateral margins anterad the angle variably convergent. The pronotal median base is minutely punctured, with ~23 punctures each side isolated by glossy cuticle. The pronotal anterior transverse impression is broad and shallow, and lined with dense longitudinal wrinkles that extend medially across the anterior callosity. The pronotal lateral marginal depression is extremely narrow, constituted only by the beaded margin adjacent to the pronotal disc. The pronotal apex is distinctly broader than the base; APW/BPW = 1.14–1.20. All elytral intervals are flat, though the sutural interval may be slightly convex on the middle of the disc (Fig. 115A), with the sutural stria the most punctate. The 2^nd^ stria may be serially punctate and slightly impressed on the disc, or indicated by a series of isolated punctures. The vertex is glossy, bearing only obsolete microsculpture that is difficult to discern. The pronotal disc and median base are also glossy, with obsolete transverse microsculpture over parts of the discal surface and obsolete isodiametric sculpticells in the laterobasal depression. The elytral disc is glossy with an obsolete, elongate transverse mesh; patches of elongate transverse sculpticells on the elytral apex.

**Male genitalia** (n = 2). Aedeagal median lobe gracile, distance from parameral articulation to tip 4.1× depth at midlength (Fig. 116A, C); apex narrowly extended beyond ostial opening 3× its minimum depth, tip curved ventrally with oblique apical face; median lobe straight in ventral view, though right and left margins are sinuously subparallel as they narrow to an acuminate tip (Fig. 116B); internal sac with evident, though small, dorsal and ventral ostial microtrichial patches (left and right patches in ventral view, Fig. 116B); flagellar plate apparently short based on uneverted specimen (Fig. 116A), length 0.29× parameral articulation-tip distance.

**Female reproductive tract** (n = 1). Bursa copulatrix columnar with rounded apex, length 0.94 mm, breadth 0.38 mm (Fig. 117A); bursal walls thickly wrinkled, base more translucent, apex more opaque; gonocoxite 1 with 3–4 apical fringe setae, a curved seta at medioapical angle and 6–7 smaller setae along medial surface (Fig. 118A); gonocoxite 2 narrowly falcate with tightly rounded tip and elongate basal extension with curved terminus, 2 lateral ensiform setae, apical nematiform setae on medioventral surface at 0.72× gonocoxite length.

##### Lectotype.

Female (BMNH) hereby designated, labeled: Metrothorax laticollis Type D.S. Hawaii Perkins 680 // Type // Hawaiian Is. Perkins 1904-336. // Mecyclothorax vitreus n.n. for Metrothorax laticollis Sharp E.B. Britton det. 1939 // LECTOTYPE Metrothorax laticollis Sharp J.K. Liebherr 1998 (black-margined red label). The type locality represented by R.C.L.P. lot 680 is HI: Maui, Haleakala, 4000+ ft. (Anonymous N D), in the Waikamoi area above Olinda.

##### Distribution and habitat.

*Mecyclothorax vitreus* is found at moderate elevations in the Waikamoi area, with all records spanning 1170–1830 m elevation (Fig. 119). More recently is has been collected in association with koa trunks, or by beating vegetation at night.

#### (092) Mecyclothorax perkinsianus

Taxon classificationAnimaliaColeopteraCarabidae

(Sharp)

Figs 115C , 116D , 117B , 118B

Metrothorax perkinsianus Sharp 1903: 270.
Mecyclothorax perkinsianus , Britton 1948b: 135.

##### Diagnosis.

Beetles of this species (Fig. 115C) exhibit a dorsal body surface that is as glossy as that of *Mecyclothorax vitreus* (Fig. 115A–B), but the pronotum is much less transverse, MPW/PL = 1.08–1.10, and the lateral pronotal seta is present. The sutural and 2^nd^ striae are slightly impressed on the disc in association with a slightly convex interval 2. The elytra are rufobrunneous, with the sutural interval paler, rufous basally and rufoflavous apically. In keeping with the narrow elytral base, the humeri are more proximate than in any other member of the group; MEW/HuW = 2.42–2.53. Setal formula 2 1 2 0. Standardized body length 5.2–5.3 mm.

##### Identification

(n = 2). The eyes are very convex, ocular ratio = 1.57–1.59, and cover much of the protruded ocular lobe, ocular lobe ratio = 0.91. The pronotal hind angles are slightly obtuse with tightly rounded apex, with the margin behind the angles convex, the basal margin continued in a trisinuate curve. The lateral margins are subparallel for only a short distance anterad the hind angles. The pronotal median base is smooth with ~16–17 large isolated punctures each side. The pronotal anterior transverse impression is shallow but distinct, crossed by some fine, shallow longitudinal wrinkles that extend medially across the anterior callosity. The pronotal lateral marginal depression is extremely narrow, constituted only by the beaded margin adjacent to the pronotal disc. The pronotal apex is broader than the base; APW/BPW = 1.07–1.12. The vertex is glossy, bearing only obsolete microsculpture that is difficult to discern. The pronotal disc and median base are also glossy, with obsolete transverse microsculpture over parts of the discal surface and obsolete transverse sculpticells in the laterobasal depression. The elytral disc is glossy with an obsolete, elongate transverse mesh; patches of indistinct isodiametric sculpticells on the elytral apex.

**Male genitalia** (n = 1). Aedeagal median lobe parallel sided along shaft, moderately robust, distance from parameral articulation to tip 3.8× depth at midlength (Fig. 116D); apex expanded dorsally a blunt projection, ventrally as acutely rounded tooth, the apical face between slightly convex; internal sac with dorsal ostial microtrichial patch near base, distinct, round ventral ostial microtrichial patch at 1/3 sac length; flagellar plate very large, robust, length 0.67× parameral articulation-tip distance.

**Female reproductive tract** (n = 1). Bursa copulatrix columnar, thin and elongate with apical expansion, length 1.42 mm, apical breadth 0.46 mm, midlength breadth 0.32 mm (Fig. 117B); bursal walls thickly wrinkled, base more translucent; gonocoxite 1 with 4 apical fringe setae, a thicker seta basad medioapical angle and 7–10 smaller setae along medial surface (Fig. 118B); gonocoxite 2 broad basally, with lateral margin straight near lateral ensiform setae, apex subacuminate, base broadly extended with curved terminus, 2 broad and elongate lateral ensiform setae, apical nematiform setae on medioventral surface at 0.76× gonocoxite length.

##### Lectotype.

Male (BMNH) hereby designated, labeled: Metrothorax perkinsianus Type D.S. Haleakala Perkins 1902 // Type // LECTOTYPE Metrothorax perkinsianus Sharp J.K. Liebherr 1998 (black-margined red label). Sharp (1903: 271) states “This species was discovered by the naturalist to whom we are indebted for *Atelothorax optatus.”* Under *Atelothorax optatus*, junior synonym of *Mecyclothorax cognatus*, Sharp writes: “The unique exponent was found on Haleakala last year by a friend of Mr. Perkins. I regret that I do not know the name (1903: 269).” Sharp (1896, 1903) did know and write about Dr. Albert Koebele and Brother Matthias Newell of Wailuku, Maui, but he did not mention George C. Munro, the noted ornithologist who emigrated to Hawai‘i in 1890 to collect birds with Henry C. Palmer for Lord Walter Rothschild (Amadon 1964). Munro provided specimens to Perkins; e.g., the types of *Mecyclothorax munroi* (Perkins 1937), junior synonym of *Mecyclothorax karschi* Blackburn (Liebherr 2011), and *Blackburnia munroi* (Perkins 1936). Specimens of *Mecyclothorax planatus* and *Mecyclothorax cognatus* included in this revision were collected in 1936 at Olinda by Munro. These later specimens do not prove that Munro was the “naturalist” in question who was the only person to see *Mecyclothorax perkinsianus* in nature, but Munro would have favored the Waikamoi collecting area—the range of *Mecyclothorax cognatus* (Fig. 31)—as it has always been well known for its native birds.

##### Distribution and habitat.

*Mecyclothorax perkinsianus* is the only Haleakalā *Mecyclothorax* species for which we have no authoritative locality information. Based on the forest habitats available to entomologists at the turn of the 20^th^ Century, bolstered by the best guess of G.C. Munro as the collector, the Waikamoi forest at 1300 m elevation seems the most likely collecting site, and so is designated the type locality.

#### (093) Mecyclothorax kipwilli sp. n.

Taxon classificationAnimaliaColeopteraCarabidae

http://zoobank.org/6B1AA074-AEC7-49D8-BF2D-C168D58514C0

**Figure 120.**
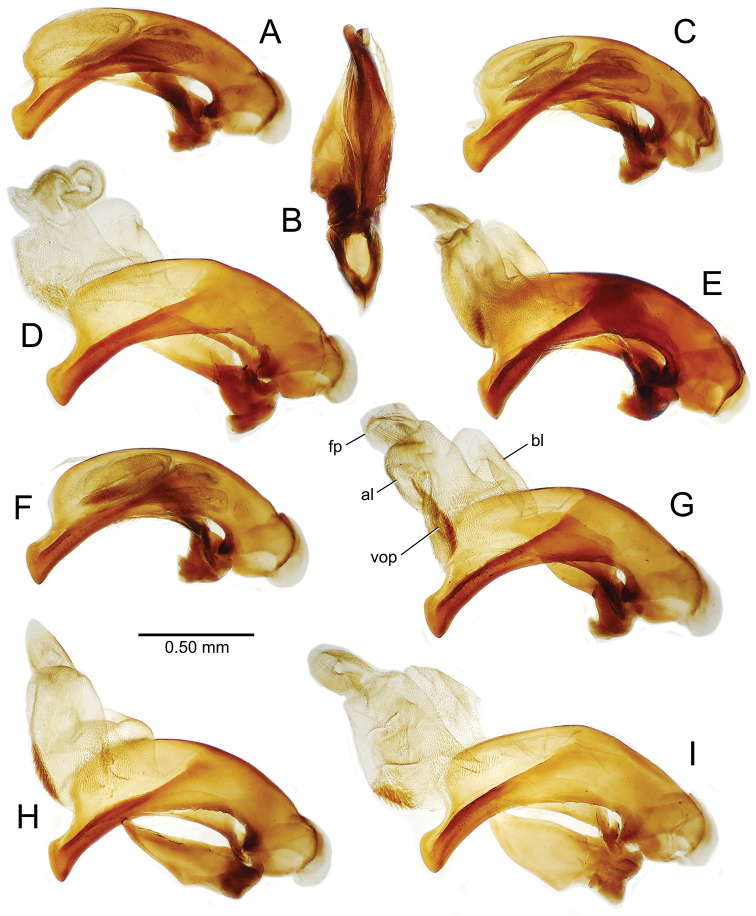
Male aedeagus, *Mecyclothorax kipwilli* (for abbreviations see Table 2, p. 23). **A-B** Right and ventral views (Honomanu, 1700 m) **C** Right view (Ke‘anae, 1325 m) **D** Right view, sac everted (Kuhiwa, 2070–2100 m) **E** Right view, sac everted. (Kuhiwa, 1590 m). **F** Right view (New Greensword Bog, 1850 m) **G–I** Right view, sac everted **G** (Horseshoe Bog, 1830 m) **H–I** (Helele‘ike‘oha, 1795 m).

**Figure 121.**
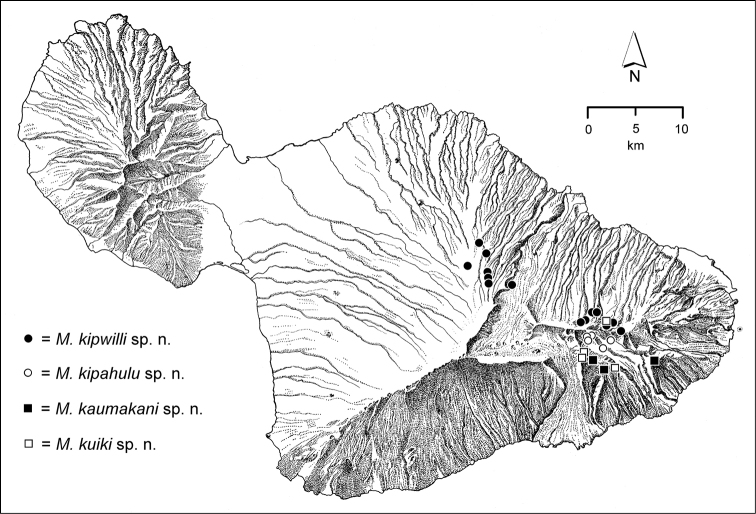
Recorded geographic distributions of *Mecyclothorax vitreus* group species.

Figs 115D–E , 117C , 118C , 120 , 121

##### Diagnosis.

**Figure 122.**
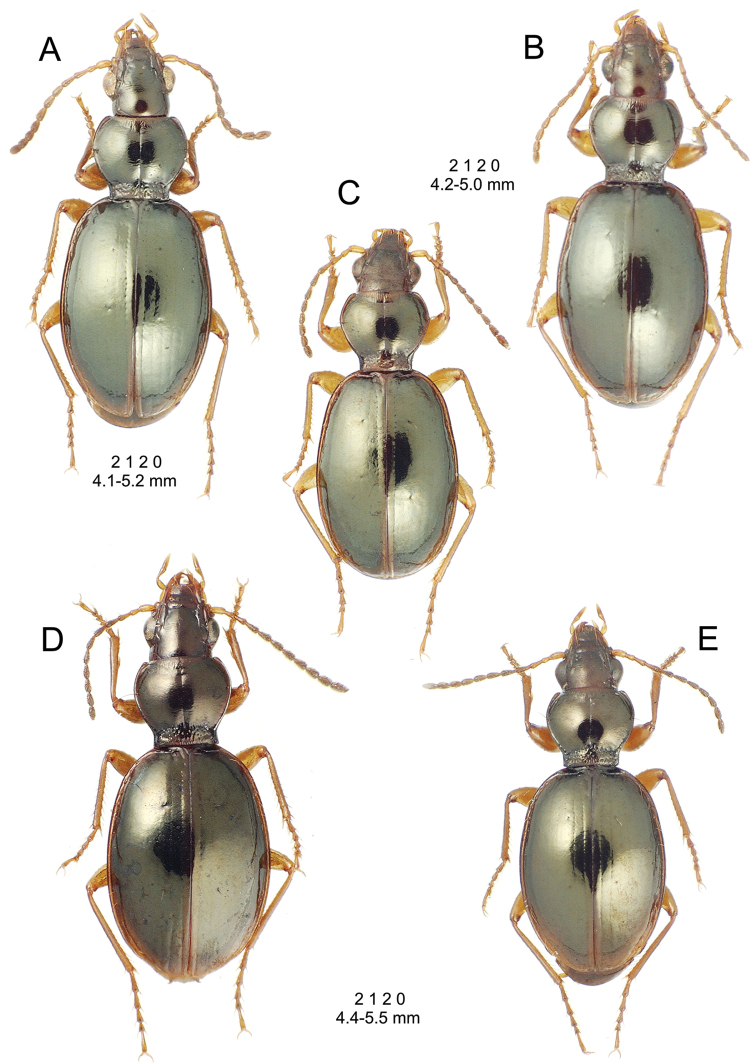
*Mecyclothorax vitreus* group species, dorsal habitus view. **A** *Mecyclothorax kipahulu* (Kīpahulu, 1800 m) **B** *Mecyclothorax kaumakani* (Kīpahulu W rim, 1850 m) **C** *Mecyclothorax kaumakani* (Pu‘u Ahulili, 1600 m) **D** *Mecyclothorax kuiki* (ESE Kuiki, 2105 m) **E** *Mecyclothorax kuiki* (New Greensword Bog, 1850 m).

The four newly described species of the *Mecyclothorax vitreus* group complex—this species (Fig. 115D–E), *Mecyclothorax kipahulu* (Fig. 122A), *Mecyclothorax kaumakani* (Fig. 122B–C), and *Mecyclothorax kuiki* (Fig. 122D–E)—were sorted for description principally by the configuration of the male aedeagus. Thus any external characters used here and in the key above are at best guideposts on the way to an identification, with a dissected male necessary for an authoritative determination, especially in the geographic area of extreme sympatry surrounding Kīpahulu Valley (Fig. 121). Nevertheless, based on available specimens, beetles of this species have the elytra more broadly ovoid relative to the width of the head across the eyes than observed in specimens of the other species; MEW/MHW = 2.18–2.21 (note: the span of these values for *Mecyclothorax kuiki* = 2.08–2.18). Pronotal shape, elytral striation, and microsculpture, usually of great help for species-level identification, are so variable infraspecifically that consulting the aedeagus is the only means to put a name on a specimen of this species with any confidence. That said, the male aedeagal median lobe is distinctive and relatively stable morphologically across this species’ range (Fig. 120), with the apex broad dorsoventrally and blunt apically. The aedeagal internal sac bears a ventral ostial microtrichial patch, and the lobe is only partially divided into an apical versus a basal lobe (Fig. 120G). Setal formula 2 1 2 0. Standardized body length 3.8–5.1 mm.

##### Description

(n = 5). *Head capsule* with frontal grooves deep apically near clypeus, sinuous laterally to mesad anterior supraorbital seta, separated from seta by low carina; dorsal impression of neck broad, shallow, visible dorsally; ocular lobe moderately protruded from gena, eyes moderately convex, ocular ratio = 1.46–1.53, ocular lobe ratio = 0.80–0.89; labral anterior margin broadly shallowly emarginate; antennae filiform, antennomeres 2–3 with sparse pelage of short setae; mentum tooth with sides slightly acute, apex tightly rounded. *Pronotum* slightly, variably transverse, MPW/PL = 1.07–1.20, variably constricted basally, MPW/BPW = 1.49–1.63; hind angle slightly acute, lateral margin slightly convergent to evenly concave (in the same specimen, Fig. 115E) anterad angle; median base with 9–14 isolated punctures each side; basal margin straight medially, slightly expanded posterad behind laterobasal depressions; median longitudinal impression very shallow, indistinct; front angles slightly projected, rounded; pronotal apex broader than base, APW/BPW = 1.05–1.14; lateral marginal depression moderately narrow, edge upturned; laterobasal depression narrow, smooth, laterally upraised to margin. *Proepisternum* with 6 minute punctures along hind marginal groove; prosternal process medially depressed with a low lateral marginal bead. *Elytra* convex, sides depressed; basal groove evenly and briefly curved to tightly rounded to obtusely angulate humerus at juncture of basal groove and lateral marginal depression; MEW/HuW = 2.32–2.44; parascutellar seta present; parascutellar striole with 4 isolated punctures; sutural interval slightly convex, lateral intervals 1–4 flat; sutural stria deep and distinct apically, whereas 2^nd^ stria shallow, broad there; 8^th^ interval laterad 7^th^ stria as convex as fused apical portion of striae 5 + 7; 2 dorsal elytral setae at 0.25× and 0.59–0.66× elytral length, setal impressions spanning ½ width of interval 3; lateral elytral setae arranged in anterior series of 7 setae and a posterior series of 6 setae; elytral marginal depression narrow, lateral margin upturned; subapical sinuation shallow, broad. *Mesepisternum* with ~16 isolated punctures in 3 rows; metepisternal width to length ratio = 0.72; metepisternum/metepimeron suture distinct; metathoracic flight wing a narrow strap 2.1× long as broad, remnant R and M veins present, strap not reaching hind margin of metanotum. *Abdomen* with irregular lateral wrinkles on ventrites 1–3; suture between ventrites 2 and 3 complete; apical male ventrite with 2 marginal setae and apical female ventrite with 4 equally spaced marginal setae plus median trapezoid of 4 short setae, the basal pair longer. *Legs*-metatarsomere 1/metatibial length ratio = 0.20; metatarsomere 4 length along outer lobe 1.5× medial tarsomere length, apical and subapical setae present; metatarsal dorsolateral sulci broad, shallow. *Microsculpture* of vertex a transversely stretched mesh; pronotal disc with indistinct elongate transverse mesh, median base with isodiametric sculpticells near punctures, glossy surface in spaces between punctures; elytral disc with indistinct to obsolete isodiametric mesh in transverse rows—sculpticells visible only in small patches; elytral apex glossy with patches of indistinct isodiametric sculpticells; metasternum covered with swirling transverse mesh; laterobasal abdominal ventrites with swirling isodiametric sculpticells. *Coloration* of vertex a glossy brunneous; antennomeres 1–3 flavous, 4–11 rufobrunneous; pronotal disc brunneous, lateral depression and edge of disc rufoflavous; proepipleuron flavous, proepisternum rufobrunneous with piceous cast; elytral disc glossy rufobrunneous, sutural interval concolorous basally, rufoflavous apically, margins narrowly rufoflavous in lateral depression, apex rufoflavous; elytral epipleuron flavous, metepisternum rufobrunneous with piceous cast; abdomen piceous basally, lateral margins of ventrites 3–6 flavous; metafemur flavous; metatibia flavous with brunneous cast.

**Male genitalia** (n = 38). Aedeagal median lobe squat, evenly curved, dorsal margin very convex along left margin of ostial opening, distance from parameral articulation to tip 2.4–2.8× depth at midlength (Fig. 120A, C–I); apex only briefly extended beyond ostial opening, obliquely blunt with apical face nearly flat to slightly convex, and ventral tip acutely rounded; median lobe straight overall in ventral view, apex curved slightly to left before rounded tip, convex dorsal margin visible to right of curved tip (Fig. 120B); internal sac broad and squat, a variably developed ventral ostial microtrichial patch (Fig. 120D–E, G–I), and a variably developed basal lobe (Fig. 120D, G) present; flagellar plate very small, length 0.20–0.30× parameral articulation-tip distance.

**Female reproductive tract** (n = 1). Bursa copulatrix broad, saclike with rounded apex, length 0.74 mm, breadth 0.46 mm (Fig. 117C); bursal walls translucent, thinly wrinkled; gonocoxite 1 with 5 apical fringe setae of 2 distinct sizes, 10–13 small setae across ventral surface (more may occur on dorsal surface but if so, they are impossible to discern) (Fig. 118C); gonocoxite 2 broadly falcate, apex subacuminate, base broadly extended laterally, 2 lateral narrow ensiform setae, apical nematiform setae on medioventral surface at 0.70× gonocoxite length.

##### Holotype.

Male (BPBM) labeled: HAWAIIAN IS: Maui (E): / Haleakala Nat. Park: / ridge E of Kipahulu / Valley, Greensword Bog: / 1859m, 22-25.VI.1981 // W.C. Gagné, Coll. / BISHOP Museum / Acc. #1981.284 // HOLOTYPE / Mecyclothorax / kipwilli / Liebherr / det. J.K. Liebherr 2015 (black-margined red label).

##### Paratypes.

256 specimens (see Appendix).

##### Etymology.

This species is named to commemorate the efforts of the first person who sorted to species the many Haleakalā *Mecyclothorax* specimens collected during the initial years of this project. Prof. Kipling W. Will was the first to separate specimens of this species now called *Mecyclothorax kipwilli*.

##### Distribution and habitat.

*Mecyclothorax kipwilli* has a bipartite Waikamoi plus Hanawī/Hāna Bogs distribution, but the eastern populations are restricted in occurrence by the speciation events that spawned the other closely related species in this complex; *Mecyclothorax kaumakani*, *Mecyclothorax kipahulu*, and *Mecyclothorax kuiki* (Fig. 121). Records of this species have been associated predominantly with ‘ōhi‘a, and secondarily with koa. Koa records lie in the upper elevations of the range (1800–1860 m el.), whereas ‘ōhi‘a records broadly range 1310–1860 m elevation. Other plant substrates include *Cheirodendron* (‘ōlapa), *Cibotium* (hāpu‘u), *Clermontia* (‘ōhā wai), *Cyanea* (haha), *Dubautia reticulata* (kupaoa), *Leptecophylla* (pūkiawe), *Melicope* (alani), and *Myrsine* (kolea).

#### (094) Mecyclothorax kipahulu sp. n.

Taxon classificationAnimaliaColeopteraCarabidae

http://zoobank.org/BE581D50-3084-4EC3-A510-0A59383095F0

**Figure 123.**
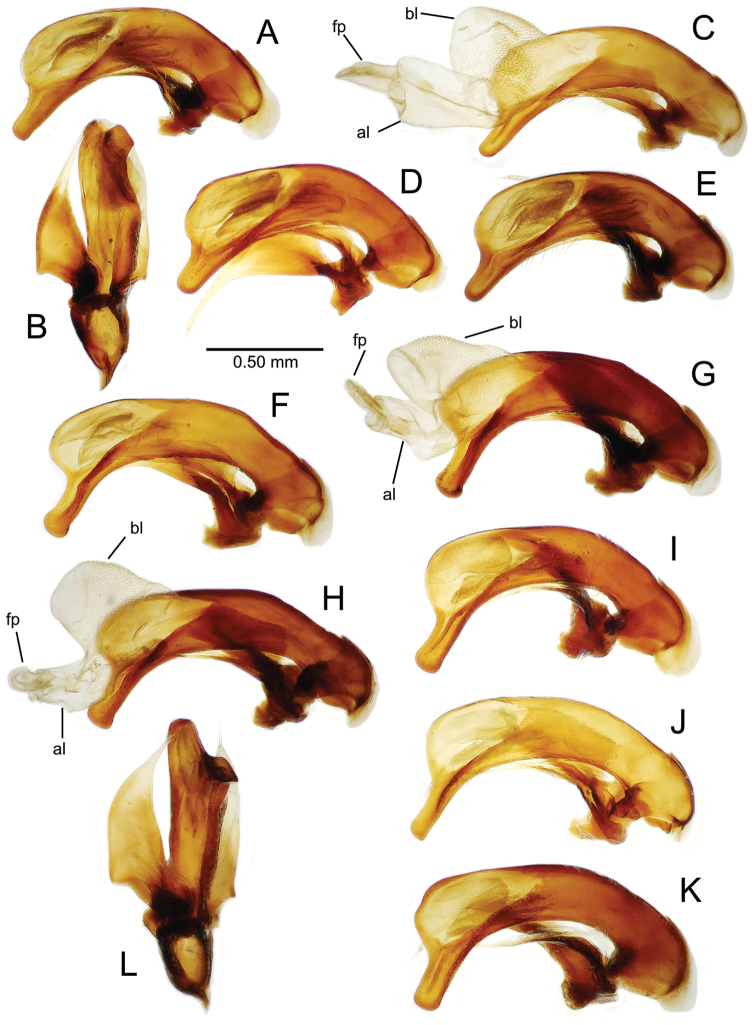
Male aedeagus, *Mecyclothorax vitreus* group species (for abbreviations see Table 2, p. 23). **A–E** *Mecyclothorax kipahulu* **A–C** Right, ventral, and right with sac everted views (Kīpahulu, 1950 m) **D** Right view (Kīpahulu, 1800 m) **E** Right view (Kīpahulu, 1500 m) **F–L** *Mecyclothorax kaumakani* **F–H** Right and right with sac everted views (Kaumakani, 1120–1150 m) **I–J** Right views. **I** (Kīpahulu W rim, 1850 m) **J** (Pu‘u Ahulili, 1600 m) **K–L** Right and ventral views (Pu‘u Ahulili, 1600 m).

**Figure 124.**
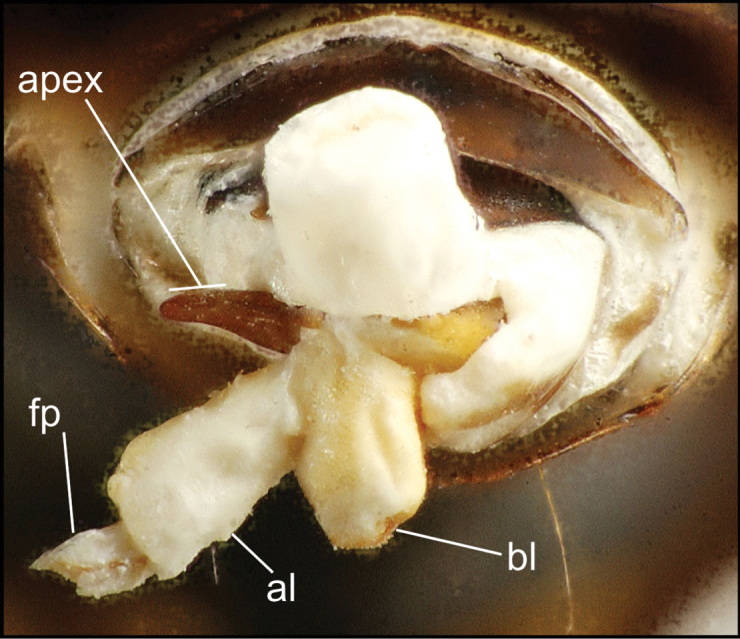
Male aedeagus of *Mecyclothorax kipahulu* in situ (Kīpahulu Vy., 1845m), ventral view, with internal sac partially everted (for abbreviations see Table 2, p. 23; apex = aedeagal apex). Aedeagus everts to left of body, and internal sac everts toward right side of aedeagal median lobe.

Figs 117D , 118D , 121 , 122A , 123A–E , 124

##### Diagnosis.

Individual of this species can be diagnosed from those of *Mecyclothorax kaumakani* and *Mecyclothorax kuiki* by the less developed elytral microsculpture. The elytral intervals are glossy, with only indistinct transverse lines, at most, visible over portions of the glossy cuticle. The male aedeagal median lobe has a short apex that is narrowed from the ostial opening to a rounded tip (Fig. 123A–E). The median lobe internal sac is divided into a shorter, broader basal lobe and a longer, thinner apical lobe (Fig. 123C). Setal formula 2 0 1 0. Standardized body length 4.1–5.2 mm.

##### Description

(n = 5). [The above description of *Mecyclothorax kipwilli* can serve to describe this species with the following substitutions.] *Eyes* more convex than in *Mecyclothorax kipwilli*; ocular ratio = 1.55–1.59, ocular lobe ratio = 0.79–0.85. *Pronotum* slightly transverse, MPW/PL = 1.16–1.19, variably constricted basally, MPW/BPW = 1.56–1.69; median base bearing 13–16 large, isolated punctures each side. *Elytra* narrowly ellipsoid, lateral margins somewhat projected laterad humeri, MEW/HuW = 2.17–2.36 (a conformation that coincides with the elytral shape of *Mecyclothorax kaumakani*, MEW/HuW = 2.10–2.27, and is broader basally, though not diagnostically so, than the elytra of *Mecyclothorax kipwilli*, MEW/HuW = 2.32–2.44, and *Mecyclothorax kuiki*, MEW/HuW = 2.30–2.39). *Mesepisternum* with ~8–10 punctures in 2 rows; metathoracic flight wing a narrow strap 1.9× long as broad, remnant M vein present; strap not reaching hind margin of metanotum. *Coloration* of vertex rufous with piceous cast; pronotal disc rufopiceous, margins narrowly paler, rufous; elytral disc glossy rufopiceous, sutural interval rufous basally and apically, concolorous on disc.

**Male genitalia** (n = 14). Aedeagal median lobe moderately robust, shaft curved, dorsal margin convex near apex of ostial opening, distance from parameral articulation to tip 3.0–3.4× depth at midlength (Fig. 123A, C–E); apical extension parallel sided, the tip evenly rounded; median lobe nearly straight in ventral view, the tip curved rightward, dorsal margin visible behind and to the left of curved tip (Fig. 123B), internal sac with large broad basal lobe, and narrower, more elongate apical lobe bearing the flagellar plate (Figs 123C, 124), sac surface covered with variously pigmented microspicules, but without distinct microtrichial patches; flagellar plate small, length 0.29× parameral articulation-tip distance.

Based on aedeagal conformation, *Mecyclothorax kipwilli* (Fig. 120), *Mecyclothorax kipahulu* (Fig. 123A–E), and *Mecyclothorax kaumakani* (Fig. 123F–L) comprise a triplet of closely related species. All have males with median lobes that are robust in breadth, distinctly curved, and have dorsoventrally broad, truncate apices. Males of all three species possess aedeagal internal sacs with both apical and basal lobes (Figs 120G, 123C, G), however in *Mecyclothorax kipahulu* and *Mecyclothorax kaumakani* males, the basal lobe approaches or exceeds the size of the plesiomorphically present apical lobe that bears the flagellar plate, supporting adelphotaxon status for these species.

**Female reproductive tract** (n = 1). Bursa copulatrix columnar with rounded apex, length 0.84 mm, apical breadth 0.34 mm, maximum breadth 0.43 mm (Fig. 117D); bursal walls thickly wrinkled at midlength, more finely wrinkled apically; gonocoxite 1 with 2–4 apical fringe setae, a curved seta at medioapical angle and 5–6 smaller setae along medial surface (Fig. 118D); gonocoxite 2 falcate, apex subacuminate, base broadly extended laterally with curved terminus, 2 elongate lateral ensiform setae, apical nematiform setae on medioventral surface at 0.69× gonocoxite length.

##### Holotype.

Male (BPBM) dissected and labeled: HAWAII: Maui I (E): / Haleakala Nat. Park / Kipahulu Valley / 1525 m, 25-XI-1980 // Sweeping // Rain forest understory // W.C. Gagné, Coll. / BISHOP Museum / Acc. #1980.545 // Mecyclothorax / kipahulu / ♂ #61 / det. J.K. Liebherr 2014 // HOLOTYPE / Mecyclothorax / kipahulu / Liebherr / det. J.K. Liebherr 2015 (black-margined red label).

##### Paratypes.

138 specimens (see Appendix).

##### Etymology.

This species is given the epithet kipahulu in reference to Kīpahulu Valley, home to Laka, a god of canoe makers (Pukui et al. 1974), and within whose boundaries this species lives (Fig. 121).

##### Distribution and habitat.

*Mecyclothorax kipahulu* is distributed within Kīpahulu Valley from 1300–2045 m elevation (Fig. 121), with its distribution parapatrically adjacent to those of *Mecyclothorax kaumakani*, *Mecyclothorax kipwilli*, and *Mecyclothorax kuiki*. The upper portion of the species distribution comprises older Kula volcanics (150-750 Ka), whereas the lower elevation portion of the distribution lies on Hāna Volcanic flow Qhn2, dated 11,000 years ago (Sherrod et al. 2007). Occurring at higher elevations in Kīpahulu, the host substrate records all involve ‘ōhi‘a, with other means of collecting including sifting leaf and moss litter, and beating vegetation at night.

#### (095) Mecyclothorax kaumakani sp. n.

Taxon classificationAnimaliaColeopteraCarabidae

http://zoobank.org/489E9B99-C79E-4C69-9D45-0459CEAB408C

Figs 117E , 118E , 121 , 122B–C , 123F–L

##### Diagnosis.

The pronotal median base is more punctate in this species versus *Mecyclothorax kipahulu* and *Mecyclothorax kuiki*, with ~16–20 punctures each side (Fig. 122B–C). The basal margin of the pronotum is smoothly curved, without dense longitudinal wrinkles as in *Mecyclothorax kuiki* (Fig. 122D–E). Also, the elytra are not so widened in their apical half, leading to a lower ratio of MEW/HuW = 2.10–2.27, versus higher ratios among individuals of *Mecyclothorax kuiki*. The male aedeagal median lobe is most like those of *Mecyclothorax kipahulu* males, but the dorsal and ventral margins are more parallel on the apex, and the tip is slightly expanded into a knob (Fig. 123F–L).

Setal formula 2 1 2 0. Standardized body length 4.6–5.0.

##### Description

(n = 5). [The above description of *Mecyclothorax kipwilli* can serve to describe this species with the following substitutions.] *Eyes* more convex than in *Mecyclothorax kipwilli*, though not diagnostically so, ocular ratio = 1.53–1.59, ocular lobe ratio = 0.79–0.87. *Pronotum* little transverse, MPW/PL = 1.10–1.15, variably constricted basally, MPW/BPW = 1.46–1.65. *Elytra* narrowly ellipsoid, lateral margins somewhat projected laterad humeri, MEW/HuW = 2.10–2.27. *Metathoracic flight wing* a narrow strap 1.3× long as broad, remnant M vein present; strap not reaching hind margin of metanotum. *Microsculpture* of pronotal median base an indistinct to distinct isodiametric mesh across the surface between punctures; elytral disc with shallow, evident transversely stretched isodiametric mesh in transverse rows. *Coloration* of vertex rufous with piceous cast; pronotal disc rufopiceous; elytral disc glossy rufopiceous, sutural interval concolorous basally rufoflavous apically; elytral epipleura rufoflavous, metepisternum rufopiceous.

**Male genitalia** (n = 11). Aedeagal median lobe moderately robust, curved, dorsal margin distinctly convex, bulging near apex of ostial opening, distance from parameral articulation to tip 3.2–3.5× depth at midlength (Fig. 123F–K); median lobe straight in ventral view, with apex extended from right side, and bulging dorsal surface visible behind and to the left of apex (Fig. 123L); internal sac with large, broad basal lobe, and narrow, short apical lobe bearing the flagellar plate, the sac surface covered with microspicules only; flagellar plate very small, length 0.19–0.20× parameral articulation-tip distance.

**Female reproductive tract** (n = 1). Bursa copulatrix broad, saclike, with basal lobe at vagina ventrad common oviduct, length 0.62 mm, breadth 0.36 mm (Fig. 117E); bursal walls more heavily stained and thickly wrinkled at basal lobe, more translucent and not wrinkled near apex; gonocoxite 1 with 3 apical fringe setae and 8 smaller setae along medial surface (Fig. 118E); gonocoxite 2 falcate, apex subacuminate, base broadly extended laterally with curved terminus, 2 lateral ensiform setae, apical nematiform setae on medioventral surface at 0.72× gonocoxite length.

##### Holotype.

Male (CUIC) dissected and labeled: HI: Maui Haleakala N.P. / Kipahulu west rim ESE Kuiki sift humus ex ohia / 15-V-1993 lot 03 / el. 1850 m // J.K. Liebherr & / A.C. Medeiros / Collectors // HOLOTYPE / Mecyclothorax / kaumakani / Liebherr / det. J.K. Liebherr 2015 (black-margined red label).

##### Paratypes.

42 specimens (see Appendix).

##### Etymology.

The distribution of this species embraces Kīpahulu Valley, with specimens collected on Kaumakani mountain to the east of Kīpahulu Valley, and along the western rim of Kīpahulu Valley ESE of Kuiki (Fig. 121). Kaumakani is taken as the species epithet for this species, allowing the next species of this complex to be named after Kuiki.

##### Distribution and habitat.

*Mecyclothorax kaumakani* is known from Kaumakani summit to the east of Kīpahulu Valley, and from near Pu‘u Ahulili on the Manawainui Planeze west of lower Kīpahulu Valley (Fig. 121); a distribution made disjunct by the presence of *Mecyclothorax kipahulu* in Kīpahulu Valley. The Kaumakani records are from relatively low elevations, 1127–1165 m—whereas the Manawainui records are from 1600–1850 m elevation. Both Kaumakani and the Manawainui Planeze comprise Kula Volcanics, dated 150-750 Ka, whereas the floor of lower Kīpahulu Valley is composed of Hāna Volcanic formation Qhn2, dated to 11,000 years ago (Sherrod et al. 2007). This history suggests that the Kaumakani and Manawainui populations of this species have not been in contact for the past 11,000 years, and that *Mecyclothorax kipahulu* colonized the lower valley floor from an upper Kīpahulu Valley Kula volcanic terrane during that time.

#### (096) Mecyclothorax kuiki sp. n.

Taxon classificationAnimaliaColeopteraCarabidae

http://zoobank.org/D00B7183-9F21-47C6-953B-44A5F935139F

**Figure 125.**
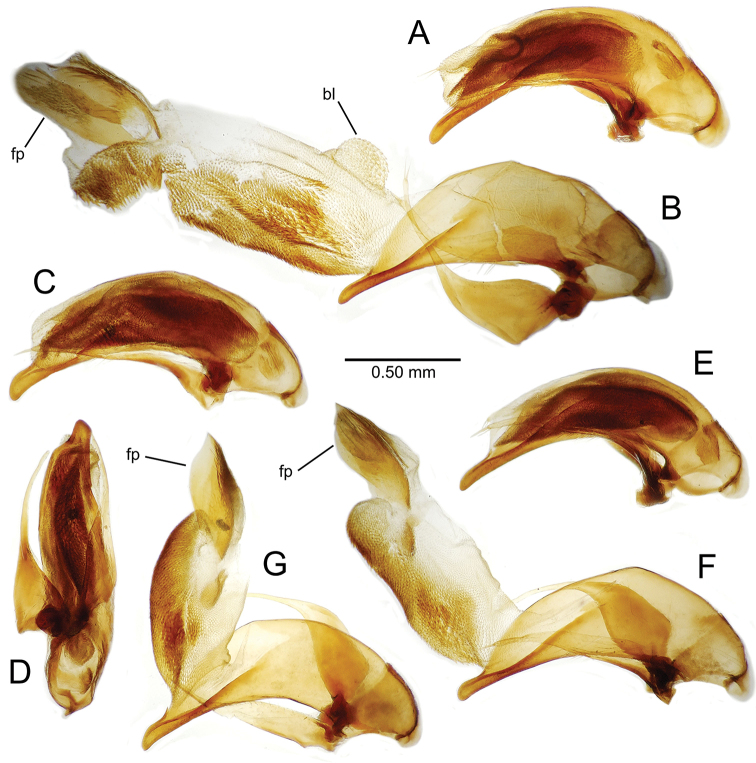
Male aedeagus, *Mecyclothorax kuiki* (for abbreviations see Table 2, p. 23). **A–B** Right, and right with sac everted views (New Greensword Bog, 1850 m) **C–E** Right and ventral views (ESE Kuiki, 2090 m) **F–G** Right view, sac everted **F** (ESE Kuiki, 2145 m) **G** (ESE Kuiki, 2105 m).

Figs 117F , 118F , 121 , 122D–E , 125

##### Diagnosis.

This fourth species of the *Mecyclothorax vitreus* group complex can be diagnosed by the pronotal median base with large punctures traversing the midline of the base, and small longitudinal wrinkles dissecting or at least disturbing the basal margin (Fig. 122D–E). The median base also has well-developed isodiametric microsculpture between the punctures. The elytra are ellipsoid, the base narrow with the margins laterad the humeri barely extended, resulting in high ratios of MEW/HuW = 2.30–2.39 that are mirrored in *Mecyclothorax kipahulu* (Fig. 122A) and *Mecyclothorax kipwilli* (Fig. 115D–E), but greater than those derived from *Mecyclothorax kaumakani* (Fig. 122B–C). The male aedeagal median lobe is of the most plesiomorphic configuration within this species complex, as the median lobe is gracile and moderately curved, and apex is narrowly extended beyond the ostial opening, the tip narrowly rounded and slightly downturned (Fig. 125A–C, F–G). The male aedeagal internal sac corroborates the median lobe’s relative plesiomorphy, as it is unilobate, long, and generally of the configuration observed across the genus (Fig. 125B, F, G). Setal formula 2 1 2 0. Standardized body length 4.4–5.5 mm.

##### Description

(n = 5). [The above description of *Mecyclothorax kipwilli* can serve to describe this species with the following substitutions.] *Eyes* of similar convexity to *Mecyclothorax kipwilli*; ocular ratio = 1.46–1.58, ocular lobe ratio = 0.76–0.81. *Pronotum* appearing elongate but slightly transverse, MPW/PL = 1.10–1.15, variably constricted basally, MPW/BPW = 1.46–1.65; median base bearing 10–13 isolated punctures across midlength of base, plus fine longitudinal wrinkles along the basal margin. *Metathoracic flight wing* an ellipsoid strap 1.4× long as broad, remnant R + M veins present; strap not reaching hind margin of metanotum. *Microsculpture* of elytral disc shallow, evident, comprising a transversely stretched isodiametric mesh arranged in transverse rows, elytral apex with evident isodiametric mesh. *Coloration* of vertex rufous with piceous cast; pronotal disc rufobrunneous with piceous cast, margins narrowly paler, rufous; elytral disc rufobrunneous, sutural interval rufous basally and apically, concolorous on disc.

**Male genitalia** (n = 11). Aedeagal median lobe robust, dorsal surface broadly convex, distance from parameral articulation to tip 2.3× depth at midlength (Fig. 125A–C, E–G); apex very narrowly extended, the tip obliquely rounded with dorsoapical face slightly flattened; median lobe sinuously curved in ventral view, right margin slightly concave, left margin briefly convex before narrow, bluntly rounded tip (Fig. 125D); internal sac very broad, cylindrical (Fig. 125B, F–G), length variable, from 1.0–1.2× parameral articulation-tip distance; a small basal lobe may (Fig. 125B) or may not (Fig. 125 F, G) be present on dorsal surface of sac; ventral sac surface covered with shaggy pelage of longer microtrichia; flagellar plate robust, very large, length 0.61–0.67× parameral articulation-tip distance (plate size is positively associated with sac length, Fig. 125B, F–G).

**Female reproductive tract** (n = 1). Bursa copulatrix columnar, broad and elongate, length 1.48 mm, breadth 0.57 mm (Fig. 117F); bursal walls translucent, surface more thickly wrinkled near base, apex with only thin wrinkles; gonocoxite 1 with 3–4 apical fringe setae and 6–9 smaller setae along medial surface (Fig. 118F); gonocoxite 2 falcate, apex subacuminate, basal extension elongate with curved terminus, 2 thin lateral ensiform setae, apical nematiform setae on medioventral surface at 0.71× gonocoxite length.

##### Holotype.

Male (CUIC) dissected and labeled: HI: Maui Haleakala N.P. / Kipahulu west rim below / Kuiki sift humus ex ohia / 14-V-1993 lot 02 / el. 2090 m // J.K. Liebherr & / A.C. Medeiros / Collectors // Mecyclothorax kuiki ♂ #80 // HOLOTYPE / Mecyclothorax / kuiki / Liebherr / det. J.K. Liebherr 2015 (black-margined red label).

##### Paratypes.

84 specimens (see Appendix).

##### Etymology.

As most specimens of this species have been found in proximity to Kuiki, the summit of Kīpahulu Valley’s west rim, that locality name is used as the epithet for this final species in the *Mecyclothorax kipwilli* + *Mecyclothorax kipahulu* + *Mecyclothorax kaumakani* + *Mecyclothorax kuiki* species quartet.

##### Distribution and habitat.

Analogous to the distribution of *Mecyclothorax kaumakani*, *Mecyclothorax kuiki* is disjunctly distributed north and south of Kīpahulu Valley (Fig. 121). In the Hāna Bogs region it is known only from New Greensword Bog, 1850 m elevation, whereas various Manawainui Planeze localities range 1525–2145 m elevation. Records are primarily associated with ‘ōhi‘a, with one collecting event associated with *Leptecophylla* (pūkiawe).

### *Mecyclothorax montivagus* species group

**Figure 126.**
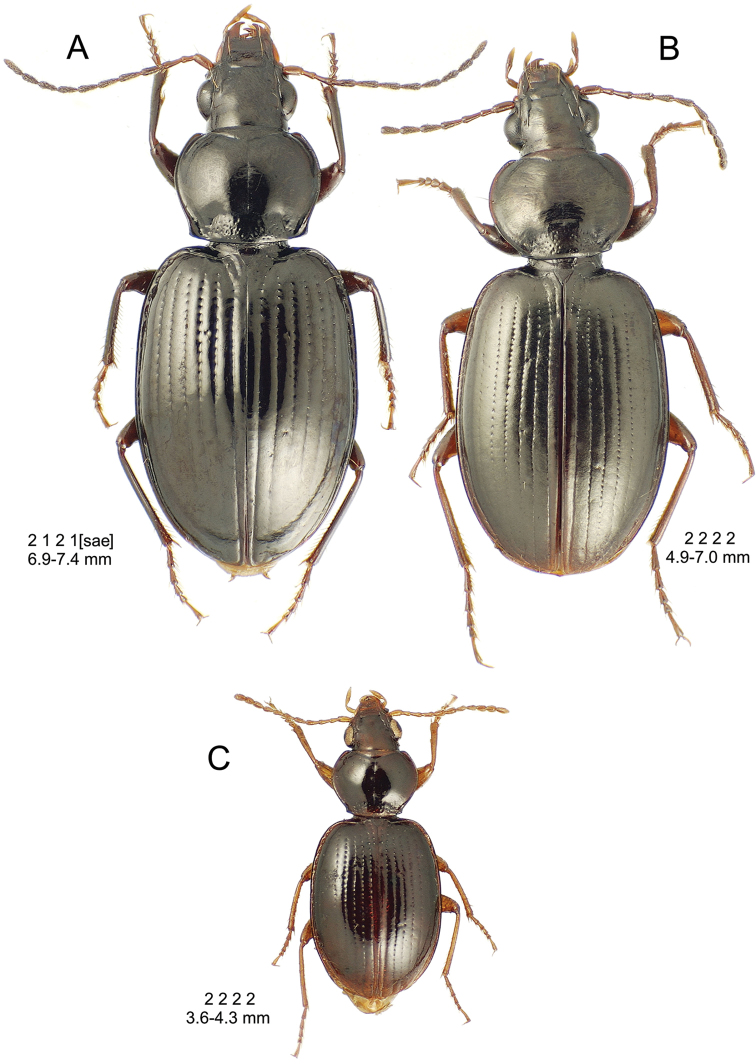
*Mecyclothorax montivagus* group species, dorsal habitus view. **A** *Mecyclothorax rex* (Waikamoi, 1300 m) **B** *Mecyclothorax montivagus* (NW upper slope, 2740 m) **C** *Mecyclothorax micans* (summit, 2895–3050 m).

**Diagnosis.** Looking in isolation at the Haleakalā species placed in this group, it is extremely difficult to understand how these three species might be members of a natural group. However admitting the additional seven member species from the Big Island of Hawai‘i into the discussion supports placement of all 10 species in the same species group. The first Haleakalā species, *Mecyclothorax rex* (Fig. 126A), is certainly the adelphotaxon—among currently known taxa—to *Mecyclothorax karschi* (Blackburn) of Hawai‘i Island (Liebherr 2008b). These two species comprise large-bodied beetles, standardized body length 6.2–7.4 mm, with very glossy, thick cuticle, and bisetose pronota with projected hind angles; setal formula 2 1 2 1[sae]. Two other Hawai‘i Island species placed in the group share that setal formula plus thick glossy cuticle; *Mecyclothorax variipes* (Sharp) and *Mecyclothorax perivariipes* Liebherr. A second subgroup of Big Island species—*Mecyclothorax kaukukini* Liebherr, *Mecyclothorax maunakukini* Liebherr, *Mecyclothorax pele* (Blackburn), and *Mecyclothorax punakukini* Liebherr—group with Haleakalā’s *Mecyclothorax montivagus* (Blackburn) and *Mecyclothorax micans* (Blackburn) based on: 1, pronotum with hind angles that project little from the convex lateral margin (Fig. 126B–C); 2, median elytral striae shallowly impressed and distinctly punctate, the lateral striae 6–7 much reduced to absent; 3, reduced dorsal microsculpture composed of transverse sculpticells; and 4, presence of all plesiomorphic setae, plus additional dorsal elytral setae in several taxa, setal formulae 2 2 2 2, 2 2 3 2, or 2 2 4 2. The two subgroups could have been separated taxonomically in this revision, but it is hypothesized that the former *Mecyclothorax karschi*-centered subgroup was derived from within the *Mecyclothorax montivagus*-centered subgroup. Such a relationship is supported by the shared pronotal configuration—hind angles little projected from convex lateral margins—observed in the former subgroup’s *Mecyclothorax variipes* and *Mecyclothorax perivariipes*, plus all taxa comprising the *Mecyclothorax montivagus* subgroup. Thus the above diagnosis of the *Mecyclothorax montivagus* subgroup represents the ground-plan for the species group, with *Mecyclothorax rex* and allies representing an offshoot derived from that plan.

**Membership and distribution.** Species placed in this group are restricted to the Big Island of Hawai‘i plus Haleakalā volcano of Maui Island. Britton (1948b) hypothesized that *Mecyclothorax montivagus* is the species phylogenetically closest to the Hawaiian ancestral colonist, as it appears most similar to the geographically widespread, extremely abundant Australian species *Mecyclothorax punctipennis* (MacLeay). Liebherr (2013) went further and hypothesized that propagules derived from Australian populations of *Mecyclothorax punctipennis* colonized both Tahiti and the Hawaiian Islands on the order of 1.4–1.8 million years ago (Ma). This hypothesis is supported by ecological data, as the *Mecyclothorax montivagus* subgroup species occupy open montane shrubland much like the open snow gum (*Eucalyptus pauciflora*) woodlands occupied by montane populations of *Mecyclothorax punctipennis* in Australia. Unlike all of the brachypterous Hawaiian and Tahitian species, *Mecyclothorax punctipennis* beetles are fully winged—macropterous—and they readily fly; e.g., they come in abundance to lights during warm Australian nights. Fitting with early divergence of *Mecyclothorax montivagus* from such a flighted ancestor, *Mecyclothorax montivagus* is characterized by the largest, most developed metathoracic flight wing vestigia of any Hawaiian *Mecyclothorax* species (see below). Paleontological data lend tangential support to this argument. Snidermann et al. (2009) discovered specimens of two species of *Mecyclothorax*—one very close to *Mecyclothorax moorei* (Baehr 2009) (N. Porch, pers. comm.), the other a member of the *Mecyclothorax cordicollis* (Sloane) complex that requires revision—in sclerophyllous vegetational deposits dated ~1.84– ~1.56 Ma from southeastern Australia. These extant Australian mainland *Mecyclothorax* species have persisted for the same length of time as the age of the oldest subaerial volcanic deposits that are found on Maui; the Wailuku Basalt from West Maui dated 1.8–2.0 Ma (Sherrod et al. 2007). Based on the above hypothesis, *Mecyclothorax montivagus* and *Mecyclothorax micans* represent taxa that diverged early on from the original Hawaiian colonist, whereas *Mecyclothorax rex* represents a more highly derived taxon that is more closely related to species from Hawai‘i Island. These morphologically more highly derived taxa—*Mecyclothorax rex*, *Mecyclothorax karschi*, *Mecyclothorax variipes*, and *Mecyclothorax perivariipes*—are all found in mesic to wet montane forest habitats demonstrating that they are also ecological specialists within this portion of the Hawaiian *Mecyclothorax* radiation.

#### Key to adults of the *Mecyclothorax montivagus* species group, Haleakalā volcano, Maui, Hawai‘i

**Table:** 

1	Pronotum quadrisetose, hind angles little protruded (Fig. 126B–C); elytra with both apical and subapical seta present, setal formula 2222	**2**
1’	Pronotum bisetose, glabrous hind angles distinctly protruded, lateral margins explanate inside angles (Fig. 126A); elytral subapical seta present, apical seta absent, setal formula 2 1 2 1[sae]	(097) ***Mecyclothorax rex* sp. n.**
2	Larger, standardized body length 5.1–7.0 mm; pronotal hind angles defining a small denticulate projection, pronotal lateral margins subparallel only laterad basal seta, anteriorly divergent (Fig. 126B); pronotal median base densely punctate	(098) ***Mecyclothorax montivagus* (Blackburn)**
2’	Smaller, standardized body length 3.6–4.3 mm; pronotal hind angles moderately projected, lateral margin distinctly sinuate anterad angle (Fig. 126C); pronotal median base minutely punctate	(099) ***Mecyclothorax micans* (Blackburn)**

#### (097) Mecyclothorax rex sp. n.

Taxon classificationAnimaliaColeopteraCarabidae

http://zoobank.org/7B328FF4-B352-40F4-81CA-3DFF861121E6

**Figure 127.**
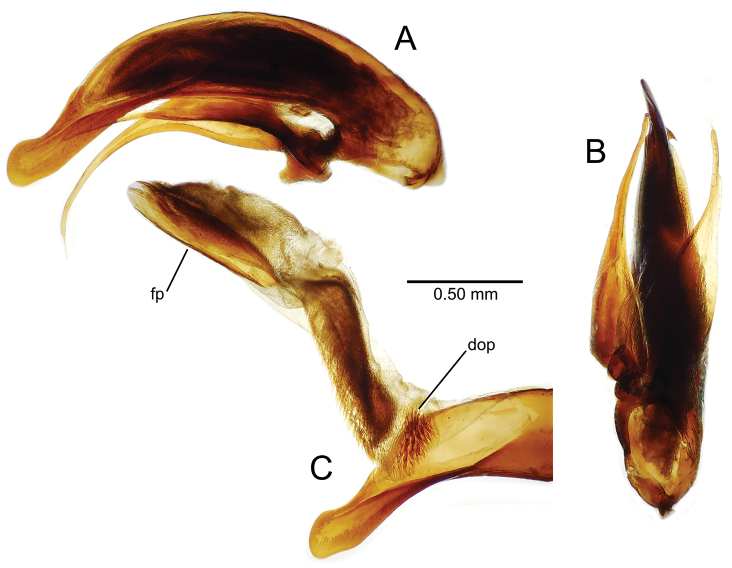
Male aedeagus, *Mecyclothorax rex* (for abbreviations see Table 2, p. 23). **A–B** Right, and ventral views (Waikamoi, 1310 m) **C** Right view of apex with internal sac everted (Waikamoi, 1310 m).

**Figure 128.**
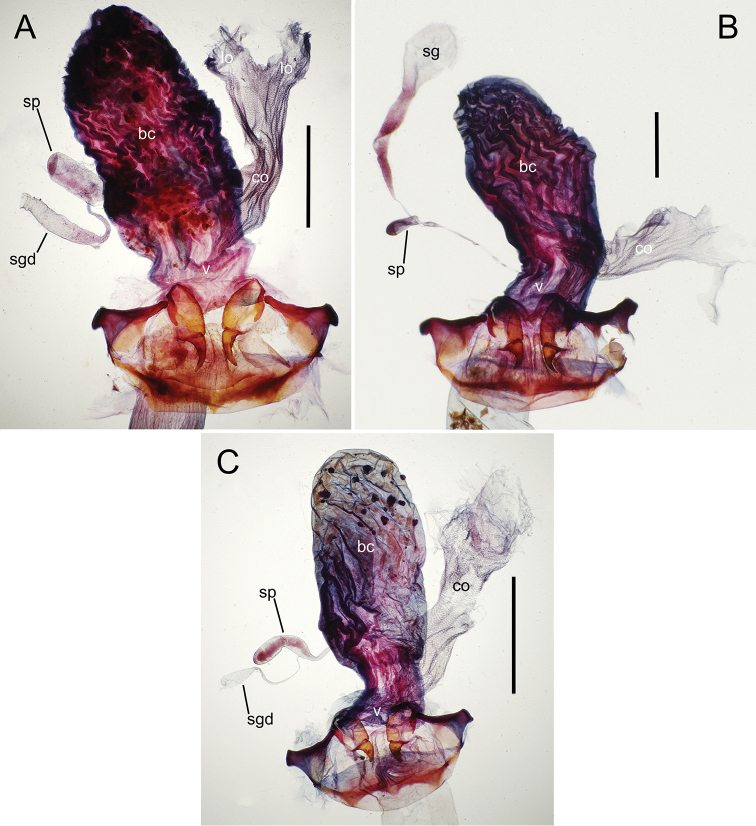
Female bursa copulatrix and associated reproductive structures, *Mecyclothorax montivagus* group species, ventral view (for abbreviations see Table 2, p. 23). **A** *Mecyclothorax rex* (Waikamoi, 1310 m) **B** *Mecyclothorax montivagus* (NW upper slope, 2740 m) **C** *Mecyclothorax micans* (Holua, 2103 m). Scale bar = 0.50 mm.

**Figure 129.**
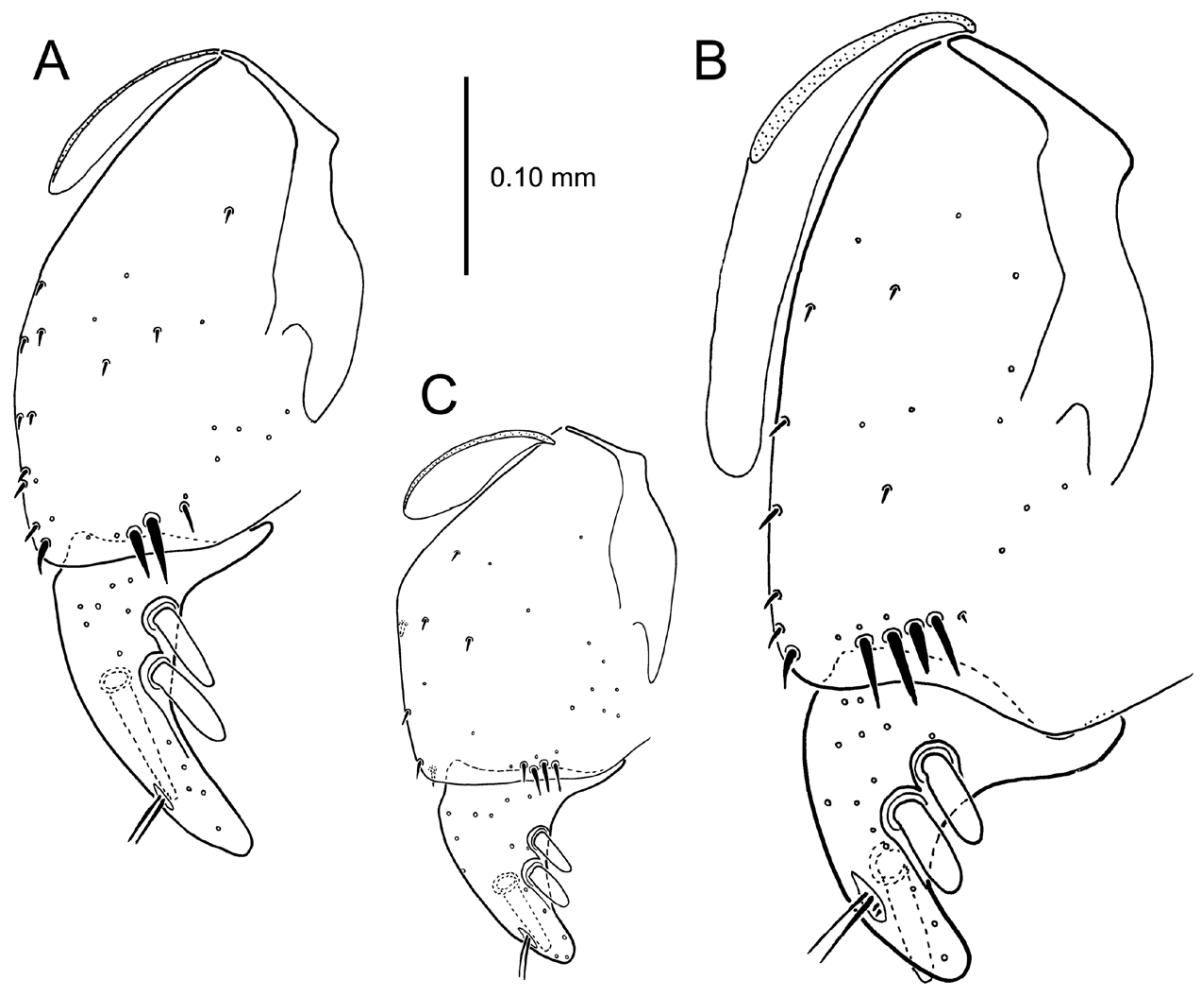
Left female gonocoxa, *Mecyclothorax montivagus* group species, ventral view. **A** *Mecyclothorax rex* (Waikamoi, 1310 m) **B** *Mecyclothorax montivagus* (NW upper slope, 2740 m) **C** *Mecyclothorax micans* (Holua, 2103 m).

**Figure 130.**
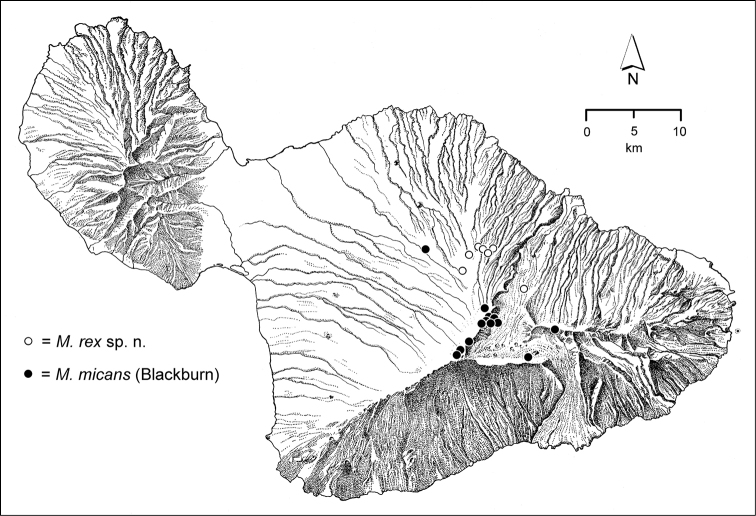
Recorded geographic distributions of *Mecyclothorax montivagus* group species.

Figs 126A , 127 , 128A , 129A , 130

##### Diagnosis.

These handsome beetles are instantly recognizable by: 1, the very large body size, standardized body length 6.9–7.4 mm; 2, dark, glossy cuticle; and 3, transverse pronotum—MPW/PL = 1.23–1.27—with projected, glabrous hind angles (Fig. 126A). The discal elytral striae are deep and distinctly punctate, the associated intervals moderately convex. The only other Hawaiian *Mecyclothorax* of similar body size is the very different-looking *Mecyclothorax molops* (Fig. 95A). Although only possible to test using first-hand experience, the elytral cuticle is so hard and brittle that use of an insect mounting pin to prepare a fully sclerotized specimen will shatter the elytron (much as would happen during such a pinning exercise for a specimen of *Clivina* Latreille). Setal formula 2 1 2 1[sae].

##### Description

(n = 4). *Head capsule* with frontal grooves broad near clypeus, shallow, lateral convexity very low; dorsal surface of neck flat to convex; ocular lobe obtusely projected from gena, ocular ratio = 1.48–1.56, ocular lobe ratio = 0.79–0.84; labral anterior margin broadly, shallowly emarginate; antennae filiform, antennomeres 2–3 glabrous except for apical setae; mentum tooth with sides acute, apex rounded. *Pronotum* broad, moderately constricted basally, MPW/BPW = 1.20–1.27; hind angle obtuse, margin behind angle convex, lateral margin anterad angle subparallel for short distance before diverging; median base smooth with minute, isolated punctures; basal margin convex between hind angles; median longitudinal impression shallow, finely incised; anterior transverse impression broad, deep, continuous medially, finely incised at front angles; anterior callosity broadly convex, smooth; front angles subangulate, not protruded; pronotal apex much narrower than basal width, APW/BPW = 0.79–0.83; lateral marginal depression narrow, edge beaded at midlength, slightly broader at front angle, broadly explanate at hind angle; laterobasal depression smooth, deep, continuous with lateral depression. *Proepisternum* and proepimeron with ~6 large punctures along hind marginal grooves; prosternal process broadly depressed on ventral surface, low marginal bead between coxae. *Elytra* subparallel with broadly convex apical margins, disc convex, sides depressed; basal groove absent from base of parascutellar interval, angled anteriorly at lateral edge of sutural interval and subangulate humerus at base of stria 6; elytral base broad, MEW/HuW = 1.63–1.70; parascutellar seta present; parascutellar striole shallow, 3–4 isolated punctures along length; sutural interval more convex than lateral intervals, sutural juncture upraised; sutural and 2^nd^ striae of subequal depth on disc, stria 2 evanescent at apex whereas sutural stria finely incised, deep, and smooth; all striae except sutural and 8^th^ absent from elytral apex, elytral surface evenly convex between those striae; 2 dorsal elytral setae at 0.25× and 0.55× elytral length, setal impressions small, spanning about 1/3 of interval 3; apical absent, subapical setae present in melanized track of reduced stria 7; lateral elytral setae arranged in anterior series of 7 setae and posterior series of 6 setae; elytral marginal depression narrow, lateral margin upturned; subapical sinuation very shallow, internal plica visible from dorsal viewpoint. *Mesepisternum* with ~20 punctures in 2–3 rows; metepisternal width to length ratio 0.83; metepisternum/metepimeron suture distinct; metathoracic flight wing an elongate strap, 3.7× long as broad, remnant R and M veins present, the strap extended beyond posterior margin of metanotum for 0.4× its length. *Abdomen* with anterior margin of abdominal ventrite 2 depressed along suture; abdominal ventrites 1–6 smooth; suture between ventrites 2 and 3 very shallow but traceable; apical male ventrite with 2 marginal setae and apical female ventrite with 4 equally spaced setae and median trapezoid of 4 subequal, short setae. *Legs*-metatarsomere 1/metatibial length ratio = 0.17; metatarsomere 4 length along outer lobe 1.2× medial tarsomere length, apical and subapical setae present; metatarsal dorsolateral sulci broad, shallow. *Microsculpture* of vertex, pronotal disc and base obsolete, surfaces glossy; elytral disc with obsolete, elongate transverse mesh, apex without microsculpture, surface glossy; metasternum and laterobasal abdominal ventrites with glossy surfaces. *Coloration* of vertex rufopiceous; antennomere 1 rufoflavous, antennomeres 2–11 with piceous cast; pronotal disc and margins rufopiceous; proepipleuron rufobrunneous with piceous upper margin, proepisternum rufobrunneous with piceous cast; elytral disc rufopiceous, sutural interval concolorous basally to rufobrunneous apically, margins concolorous with disc; elytral epipleuron and metepisternum rufous; abdomen rufous with a piceous cast, apex of apical ventrite only slightly paler; metafemur rufoflavous with median piceous cloud; metatibia rufopiceous with tibial setae golden.

**Male genitalia** (n = 2). Aedeagal median lobe large, moderately gracile, distance from parameral articulation to tip 3.9× depth at midlength (Fig. 127A); apex extended 2.5× depth beyond apex of ostial opening, curved dorsally near tip that is slightly expanded dorsally, and broadly convex apically; median lobe straight in ventral view, lateral margins evenly convergent to knifelike apex that is slightly curved toward left at tip (Fig. 127B); internal sac narrow, stalklike, with a broad dorsal ostial microtrichial patch at base, and ventral surface broadly covered with shaggy, melanic, microspicules (Fig. 127C); flagellar plate elongate, as long as membranous internal sac, and 0.64× as long as parameral articulation-tip distance.

**Female reproductive tract** (n = 1). Bursa copulatrix columnar with rounded apex and basal constriction, length 1.48 mm, breadth 0.74 mm, basal constriction 0.45 mm broad (Fig. 128A); bursal walls translucent, thickly wrinkled; gonocoxite 1 with 3 apical fringe setae, the middle seta of series largest, a curved seta at medioapical angle and 8–10 setae on medial surface (Fig. 129A); gonocoxite 2 narrowly subtriangular with tightly rounded apex, base only moderately extended laterally, 2 lateral ensiform setae with apical seta broader and longer than basal seta, apical nematiform setae on medioventral surface at 0.70× gonocoxite length.

##### Holotype.

Female (CUIC), right elytron cracked at pin, labeled: HI: Maui Haleakala NW / slope Waikamoi Flume / Waikamoi to Haipuaena / Gulches 11-IV-1991 / el 1300 m J. Liebherr // 3 // HOLOTYPE / Mecyclothorax / rex / Liebherr / det. J.K. Liebherr 2015 (black-margined red label).

##### Paratypes.

HI: Maui: Koolau For. Res., Flume Rd., wet forest, yellow pan trap, 1280 m el., vi-viii-2006, Leblanc (UHIM, 1), Kula Pipeline Rd., wet forest, yellow pan trap, 1183–1280 m el., vi-viii-2006, Leblanc (CUIC, 5; UHIM, 6), Waikamoi Gulch, 1210 m el., vii-1956, Namba (BPBM, 1), Waikamoi Flume, beat scrape *Metrosideros*, 1310 m el., 26-v-1997 lot 06, Liebherr (CUIC, 1), Waikamoi-Maile Tr., ecotone forest, yellow pan trap, 1426–1573 m el., vi-viii-2006, Leblanc (UHIM, 1); Waikamoi N.C.P., Keanae Gap, Camp 6, 1524 m el., 09-i-1998 lot 01, Haines (CUIC, 1).

##### Etymology.

At 7.4 mm, the largest individuals of this species are among the largest *Mecyclothorax* beetles in Hawai‘i, and so the species epithet rex—Latin for king—seems an appropriate epithet to apply.

##### Distribution and habitat.

*Mecyclothorax rex* is a species of wet forest in the Waikamoi area, with an easternmost limital record in Ke‘anae Valley (Fig. 130). Localities range 1210–1573 m elevation. Most specimens have been collected from ground level, either under boards or in yellow-pan traps within ‘Ōhi‘a Wet Forest. One specimen was found in scraped moss from the trunk of a larger ‘ōhi‘a tree.

#### (098) Mecyclothorax montivagus

Taxon classificationAnimaliaColeopteraCarabidae

(Blackburn)

**Figure 131.**
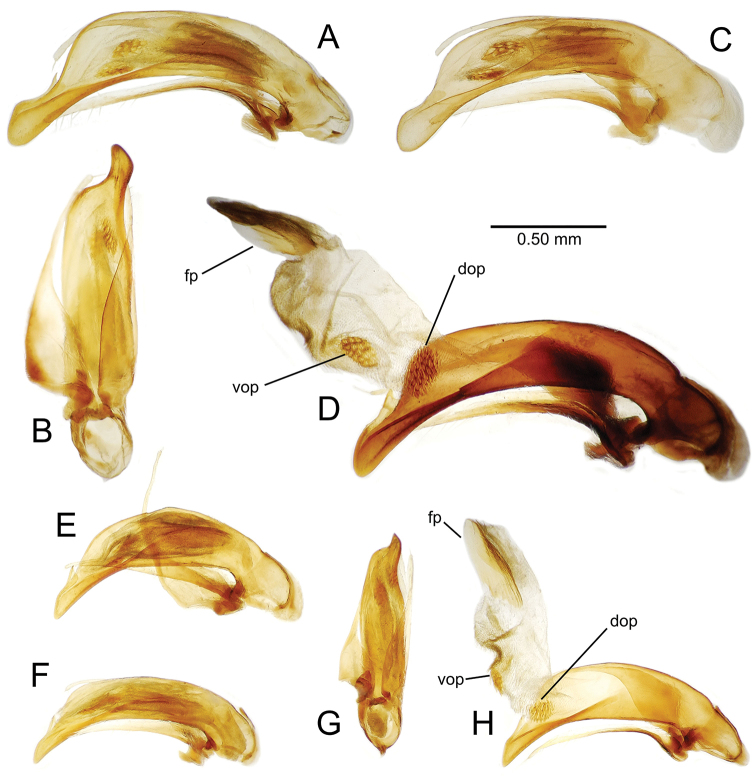
Male aedeagus, *Mecyclothorax montivagus* group species (for abbreviations see Table 2, p. 23). **A–D** *Mecyclothorax montivagus* **A–B** Right and ventral views (Leleiwi, 2438 m) **C** Right view (Kapalaoa Cabin, Haleakalā Crater, 2200 m) **D** Right view, sac everted (Holua, 2134 m) **E–H** *Mecyclothorax micans* **E** Right view (summit, 2895–3050 m) **F–G** Right and ventral views (Holua, 2134 m) **H** Right view, sac everted (NW upper slope, 2745 m).

**Figure 132.**
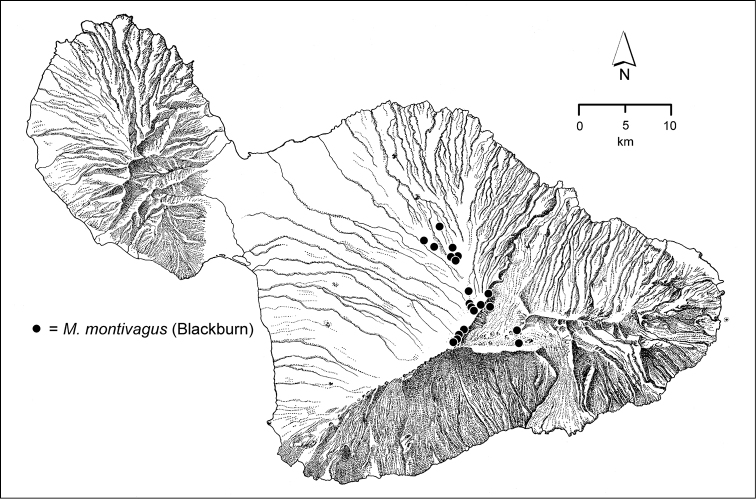
Recorded geographic distribution of *Mecyclothorax montivagus*.

Figs 126B , 128B , 129B , 131A–D , 132

Cyclothorax montivagus Blackburn 1878a: 122; Blackburn and Sharp 1885: 214.
Mecyclothorax montivagus , Sharp 1903: 253; Britton 1948b: 133.
Olisthopus insularis Motschulsky, Karsch 1881: 1 (misidentification).

##### Diagnosis.

This is one of two Haleakalā species (Fig. 126B–C) in this group that exhibit the full complement of supraorbital, pronotal, and dorsal and apical/subapical elytral setae; setal formula 2 2 2 2. However *Mecyclothorax montivagus* beetles are of moderate to large size for Hawaiian *Mecyclothorax*—standardized body length 4.9–6.9 mm—in contrast to those of *Mecyclothorax micans*; standardized body length 3.6–4.3 mm. If a qualitative character is desired to separate the species, one may use the presence of the parascutellar seta in individuals of this species, and the lack thereof in individuals of *Mecyclothorax micans*.

##### Identification

(n = 5). The frontal grooves are broad medially, with a slight hitch laterad near midlength, the groove terminated at a fine carina mesad the anterior supraorbital seta. The head capsule is transversely concave between the hind eye margins, resulting in a broad shallow neck that is visible in dorsal view. The pronotum is transverse, MPW/PL = 1.28–1.35, with the lateral margins sinuate for only a very short distance anterad the hardly projected hind angles, the angles themselves obtuse and appearing as little more than denticles at the terminus of the lateral marginal bead posterad the laterobasal depressions; MPW/BPW = 1.47–1.57. The median pronotal base is densely punctate with elongate wrinkles lining the juncture of the base and disc. The pronotal anterior transverse impression is smooth, shallow, with the anterior callosity upraised, flat to slightly convex, and smooth. The elytra are subquadrate with rounded lateral margins posterad the broadly rounded humeri; MEW/HuW = 1.97–2.05. Discal striae 1–4 are impressed, distinctly punctate, stria 5–6 are progressively shallower, with punctures more isolated along their length, and stria 7 is absent. The mesepisternum is less punctate than in *Mecyclothorax rex*, with ~9 minute punctures in 2 rows. The metathoracic flight wings are the least reduced—though still vestigial—of any Hawaiian *Mecyclothorax*; flight wing vestigium an apically narrowed strap, 3.3× long as broad, remnant C, R, M, and Cu veins present, and the strap extended for 0.3× its length beyond the hind margin of the metanotum. Microsculpture is reduced: 1, vertex and pronotal disc with obsolete transverse mesh; 2, pronotal median base with an obsolete transversely stretched isodiametric mesh; 3, elytral disc with obsolete elongate transverse mesh, apex glossy; 4, surfaces of metasternum and laterobasal abdominal ventrites mostly glossy, shallow transverse-mesh microsculpture over portions of cuticle.

**Male genitalia** (n = 8). Aedeagal median lobe large but gracile, distance from parameral articulation to top 4.5–4.9× depth at midlength (Fig. 131A, C–D); apex broadly extended for about 1.6× depth beyond ostial opening, tip variously convex dorsally (Fig. 131A), expanded dorsoventrally (Fig. 131C), or nearly parallel and narrowed to rounded tip (Fig. 131D); median lobe straight along median shaft in ventral view, apex slightly curved rightward with right margin concave and left margin sinuous before bluntly rounded tip (Fig. 131B); internal sac broad, with dorsal ostial microtrichial patch near base on right side, and ovoid ventral ostial microtrichial patch on left side further out on sac (Fig. 131D); flagellar plate moderately large, length 0.48× parameral articulation-tip distance.

**Female reproductive tract** (n = 3). Bursa copulatrix columnar with rounded apex, length 1.65 mm, breadth 0.80 mm, basal constriction at vagina 0.45 mm broad (Fig. 128B); bursal walls translucent with thick wrinkles; gonocoxite 1 with 4 apical fringe setae, a curved seta at apicomedial angle and ~7 setae on medial surface (Fig. 129B); gonocoxite 2 broadly subtriangular, apex broad, basally extended by a long panhandle with curved terminus, 2 broad lateral ensiform setae, apical nematiform setae on medioventral surface at 0.65× gonocoxite length.

##### Lectotype.

Male (BPBM) hereby designated, dissected, and labeled: mounting platen with Blackburn Maui label (Zimmerman 1957: 210), 5 mont (on reverse) // Type // Sandwich Is. 80-6 // LECTOTYPE Cyclothorax montivagus Blackburn J.K. Liebherr 1998 (black-margined red label).

##### Distribution and habitat.

*Mecyclothorax montivagus* is a species of *Deschampsia* grassland found along the upper Kula face and in Haleakalā Crater (Fig. 132). Present day elevations range 2134–2800 m elevation, though historical records from Finsch (Karsch 1881) and Perkins (lot 112; Anonymous N D) were from Olinda near 1210 m elevation. Specimens have also been found in leaf litter under *Sophora chrysophylla* (māmane) at 2750 m elevation, and under rocks in drier, less vegetated situations from 1830–2440 m elevation.

#### (099) Mecyclothorax micans

Taxon classificationAnimaliaColeopteraCarabidae

(Blackburn)

Figs 126C , 128C , 129C , 130 , 131E–H

Cyclothorax micans Blackburn 1878a: 122; Blackburn and Sharp 1885: 214.
Mecyclothorax micans , Sharp 1903: 244; Britton 1948b: 131.

##### Diagnosis.

This species (Fig. 126C) shares with *Mecyclothorax montivagus* (Fig. 126B): 1, a broad subquadrate elytra with broadly rounded humeri, MPW/HuW = 1.91–2.05; and 2, a transverse pronotum—MPW/PL = 1.36–1.32—with briefly sinuate lateral margins anterad the obtuse hind angles. The parascutellar seta is absent. The cuticular microsculpture is similarly to that of *Mecyclothorax montivagus*: 1, vertex and pronotal disc with obsolete transverse mesh; 2, pronotal median base glossy; 3, elytral disc with obsolete elongate transverse mesh, apex with isodiametric sculpticells in transverse rows; 4, metasternum glossy; and 5, laterobasal abdominal ventrites with swirling isodiametric and transverse sculpticells. Setal formula 2 2 2 2. Standardized body length 3.6–4.3 mm.

##### Identification

(n = 5). The eyes are convex and large, and are situated on ocular lobes protruded from the gena, ocular ratio = 1.52–1.58, ocular lobe ratio = 0.87–0.88. The dorsal surface of the head is convex. The pronotal median base is broadly smooth with minute punctures, and the pronotal lateral marginal depression is narrow with the edge beaded. The elytral discal striae 1–3 are impressed and distinctly punctate, stria 4 shallower with punctures more isolated, and stria 5 represented by a series of isolated punctures. Stria 6 may be represented by a few remnant punctures, or be absent as is stria 7. The mesepisternum bears ~6 shallow punctures in 2 rows. The metathoracic flight wings are a vestigial strap 2.5× long as broad, with remnant R and M veins present, and the strap apex extended to the hind margin of the metanotum.

**Male genitalia** (n = 7). Aedeagal median lobe gracile, distance from parameral articulation to tip 3.4–4.4× depth at midlength (Fig. 131E–H); apex obliquely narrowed along dorsal margin to acutely rounded tip; median lobe straight in ventral view, apex slightly curved rightward (Fig. 131G; compare to Fig. 131B); internal sac with dorsal ostial microtrichial patch near base on right side, and ventral ostial microtrichial patch at 1/3 sac length; flagellar plate large, length 0.55× parameral articulation-tip distance.

**Female reproductive tract** (n = 1). Bursa copulatrix elongate, ellipsoid with a basal constriction distad vagina, length 1.14 mm, breadth 0.45 mm, basal constriction 0.23 mm broad (Fig. 128C); bursal walls translucent, thinly wrinkled; gonocoxite 1 with 4 very short apical fringe setae, a curved seta at apicomedial angle and 6–7 setae on medial surface (Fig. 129C); gonocoxite 2 broadly subtriangular, apex broadly rounded, 2 lateral ensiform setae, apical nematiform setae on medioventral surface at 0.75× gonocoxite length.

##### Lectotype.

Male (BMNH) hereby designated, labeled: mounting platen with Blackburn Maui label (Zimmerman 1957: 210), Cyc micans (on reverse) // Type // Hawaiian Is. Rev. T. Blackburn 1888-30. // LECTOTYPE Cyclothorax micans Blackburn J.K. Liebherr 1998 (black-margined red label).

##### Distribution and habitat.

*Mecyclothorax micans* can be found in the same situations as *Mecyclothorax montivagus* (Figs 130, 132), with beetles of this species looking like miniature versions of the latter when encountered in the field. *Deschampsia* (hairgrass) clumps and *Sophora* (māmane) litter shelter individuals of this species. Unlike *Mecyclothorax montivagus*, which is not present at the summit of the Kula face (i.e. Pu‘u ‘Ula‘ula), the range of *Mecyclothorax micans* extends to the highest elevations, where the species keeps company with *Mecyclothorax nubicola*, *Mecyclothorax pusillus*, *Mecyclothorax rusticus*, and *Mecyclothorax subconstrictus* (Figs 79, 80).

### *Mecyclothorax ducalis* species group

**Diagnosis.** Species assigned to this group have the lateral elytral striae reduced relative to the discal striae. The sutural stria is much more impressed than stria 2 at the elytral apex, though they are of similar development on the elytral disc. And the microsculpture is reduced, with the elytral disc glossy, and any traceable sculpticells on the dorsal surface very transverse. In keeping with the glossy elytral surface, the apical and subapical elytral setae are consistently absent, however the parascutellar seta and both dorsal elytral setae are always present. The pronotum may be glabrous, or laterally setose; setal formulae 2 1 2 0 or 2 0 2 0. Beetles of the member species are of moderate to large body size; standardized body length 4.2–6.7 mm.

**Membership and distribution.** This group is known only from Moloka‘i with four species (Liebherr 2007) and Haleakalā with six species. The glossy body surface is associated with reduced setation on both islands, with the Moloka‘i species exhibiting setal formulae 2 1 2 0, 2 0 2 0, and 1 0 2 0. The beetles are denizens of wet montane forest, with host plant substrates including *Metrosideros*, *Cibotium*, *Cheirodendron*, *Tetraplasandra*, and *Acacia koa*. Beetles have been found by beating vegetation, though the most specific locations are under bark of woody trees and secondarily woody herbs such as lobelioids (Campanulaceae), where the rotting cambial layer establishes a semiaquatic microhabitat.

#### Key to the adults of the *Mecyclothorax ducalis* species group, Haleakalā volcano, Maui, Hawai‘i

**Table:** 

1	Pronotum glabrous, both lateral and basal setae absent	**2**
1’	Pronotum bisetose, lateral setae present, basal setae absent	**3**
2(1)	Body size smaller, standardized body length 5.1–5.6 mm; elytral apex broadly flavous, contrasted with rufopiceous disc (Fig. 133A)	(100) ***Mecyclothorax aquilus* sp. n.**
2’	Body size larger, standardized body length 6.0–6.7 mm; elytral apex concolorous with rufopiceous disc (Fig. 133D)	(103) ***Mecyclothorax ducalis* (Sharp)**
3(1)	Body size smaller, standardized body length 4.3–5.1 mm; elytral apex pale, flavous, contrasted with piceous disc (Fig. 133B–C)	**4**
3’	Body size larger, standardized body length 5.2–6.7 mm; elytral disc and apex concolorous, brunneous to piceous, though sutural interval may be paler, rufous (Figs 133D, 140)	**5**
4(3)	Elytra broader relative to forebody, humeri broadly rounded (Fig. 133B), MEW/MPW = 1.63–1.69; male aedeagal median lobe apically expanded both ventrally and dorsally into spatulate tip (Fig. 134B, D, F–G, I, K), apical quarter of lobe curved at nearly right angle to base (Fig. 134C, E, J)	(101) ***Mecyclothorax insolitus* (Sharp)**
4’	Elytra narrow relative to forebody, humeri narrowly rounded (Fig. 133C), MEW/MPW = 1.52–1.62; male aedeagal median lobe apically expanded only dorsally, producing smooth crochet-hook configuration to tip (Fig. 138A, C), apical quarter of lobe curved at 60°angle to base (Fig. 138B)	(102) ***Mecyclothorax invisitatus* sp. n.**
5(3)	Body more gracile, elytra narrowly ovoid, humeri narrowly rounded (Fig. 140), MEW/HuW = 2.20–2.49; femora bicolored, base and apex brunneous, middle flavous, tibiae with brunneous cast	**6**
5’	Body broad, robust, elytra broadly ovoid, humeri broadly rounded (Fig. 133D), MEW/HuW = 2.06–2.15; femora starkly bicolored, base and apex piceous versus flavous middle, tibiae with piceous cast	(103) ***Mecyclothorax ducalis* (Sharp)**
6(5)	Elytral surface glossy, sculpticells not visible; striae 1–4 impressed on elytral disc, striae 5–6 variably impressed or represented by series of isolated punctures, stria 7 shallow, smooth, traceable at midlength (Fig. 140A–B); male aedeagal median lobe with elongate, downturned and narrowly rounded apex (Fig. 141A, B, D–E) and bipartite dorsal ostial microtrichial patch (Fig. 141D–E)	(104) ***Mecyclothorax longidux* sp. n.**
6’	Elytral surface glossy in reflected light, but transverse sculpticells traceable adjacent to areas of reflected light; striae 1–2 impressed on elytral disc, striae 3–6 progressively shallower, stria 7 obsolete, traceable only as a series of very shallow impressions (Fig. 140C–D); male aedeagal median lobe with short, broad, and blunt apex (Fig. 141F–I), dorsal ostial microtrichial patch simple (Fig. 141I)	(105) ***Mecyclothorax brevidux* sp. n.**

#### (100) Mecyclothorax aquilus sp. n.

Taxon classificationAnimaliaColeopteraCarabidae

http://zoobank.org/2B2ACAB2-4A92-4D65-B01C-B643DA5DEF81

**Figure 133.**
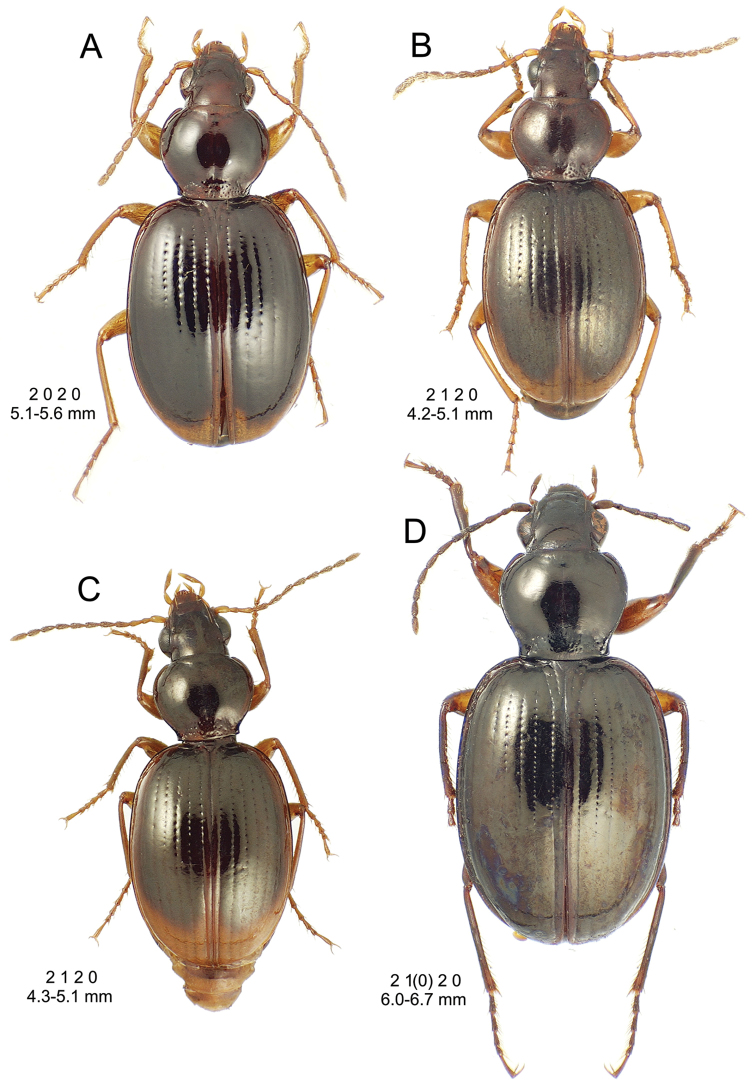
*Mecyclothorax ducalis* group species, dorsal habitus view. **A** *Mecyclothorax aquilus* (Kīpahulu, 915 m) **B** *Mecyclothorax insolitus* (Waikamoi, 1310 m) **C** *Mecyclothorax invisitatus* (Opana, 1293 m) **D** *Mecyclothorax ducalis* (Olinda to Ukulele Camp, 1210–1365 m).

**Figure 134.**
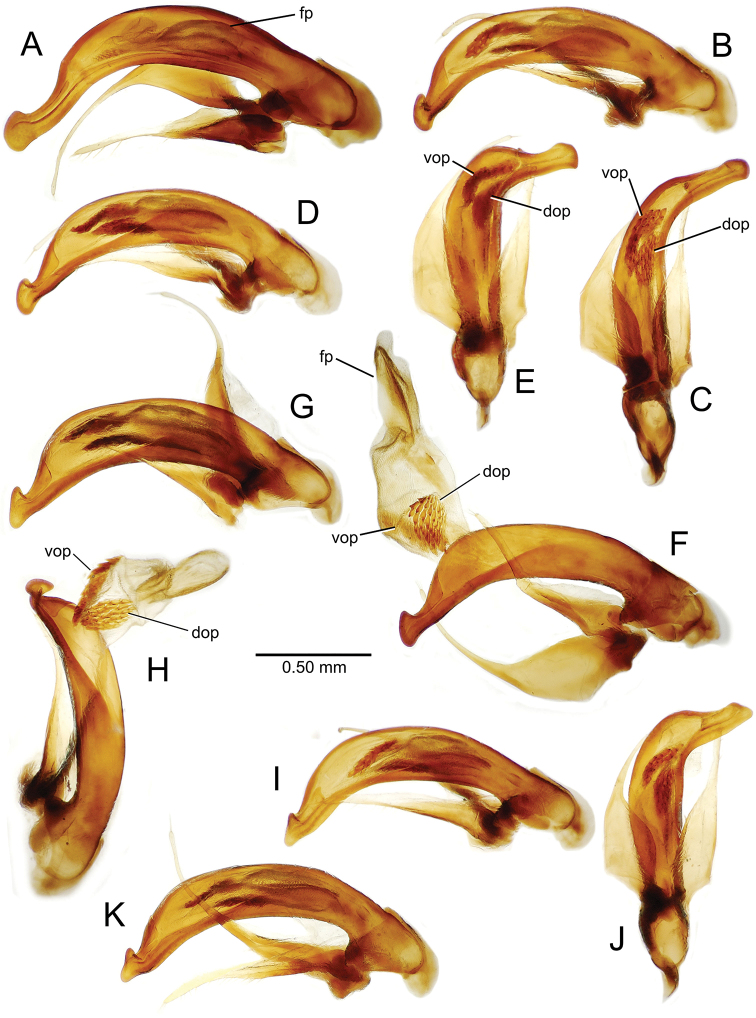
Male aedeagus, *Mecyclothorax ducalis* group species (for abbreviations see Table 2, p. 23). **A** *Mecyclothorax aquilus*, right view (Kīpahulu, 915 m). **B–K** *Mecyclothorax insolitus* **B–C** Right and ventral views (Waikamoi, 1310 m) **D–E** Right and ventral views (Waikamoi, 1265 m) **F** Right view, sac everted (Waikamoi, 1463 m) **G** Right view (Ke‘anae, 1325 m). **H** Right view, sac everted (Waikamoi, 1310 m) **I–K** Right and ventral views (Kuhiwa E rim, 880–915 m).

**Figure 135.**
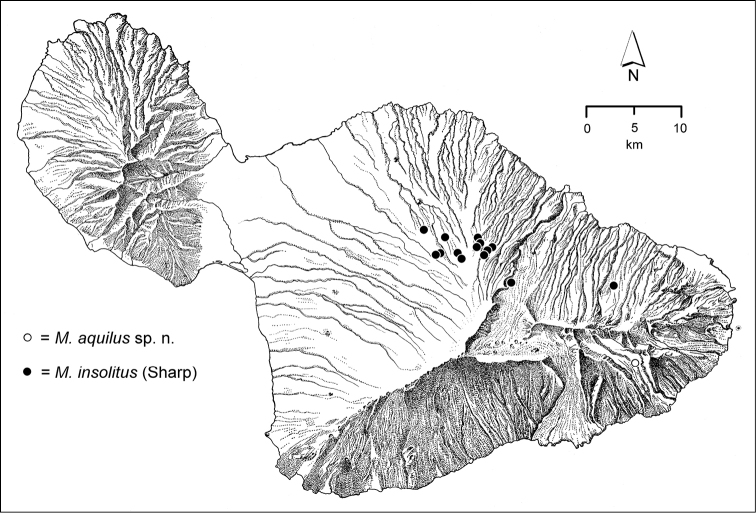
Recorded geographic distributions of *Mecyclothorax ducalis* group species.

Figs 133A , 134A , 135

##### Diagnosis.

This is the only Haleakalā species of the group that consistently exhibits a glabrous pronotum (Fig. 133A). *Mecyclothorax aquilus* is otherwise most similar in appearance to *Mecyclothorax insolitus* (Fig. 133B) and *Mecyclothorax invisitatus* (Fig. 133C) sharing the broadly flavous apical elytral margins. However beetles of this species are larger, standardized body length 5.1–5.6 mm, versus a range of 4.2–5.1 mm for the other two species. Setal formula 2 0 2 0.

##### Description

(n = 2). *Head capsule* with frontal grooves deep and broad near clypeus, a broad lateral convexity before eye that continues posterad enveloping anterior supraorbital seta; dorsal impression of neck slightly concave; ocular lobe moderately protruded from gena, eyes moderately convex, ocular ratio= 1.48–1.49, ocular lobe ratio = 0.80–0.81; labral anterior margin shallowly emarginate to 1/8 of labral length; antennae filiform, antennomeres 2–3 with sparse pelage of short setae; mentum tooth with sides acute, apex tightly rounded. *Pronotum* slightly transverse, MPW/PL = 1.11–1.16, the disc broadly convex; hind angles obtuse but sharp apically, lateral margins evenly divergent anterad, MPW/BPW = 1.52–1.56; median base slightly depressed relative to disc, ~20 minute, isolated punctures each side in glossy surface; basal margin distinctly convex between hind angles; median longitudinal impression very finely incised, broader in front of median base; anterior transverse impression very shallow, broad, incised only immediately inside front angle; anterior callosity flat medially, surface glossy; front angles slightly projected, tightly rounded; pronotal apical width variably subequal to basal width, APW/BPW = 0.97–1.06; lateral marginal depression obsolete, narrowly beaded laterally and basally, bead thicker only at hind angle, depression narrow only inside upraised margin of front angle; laterobasal depression deep, sloping from disc, margined by narrow U-shaped depression. *Proepisternum* with 5 minute punctures along hind marginal groove; prosternal process with broad median depression, lateral margins broadly upraised between coxae. *Elytra* subovoid, domed, the sides distinctly sloped to a vertical juncture with lateral marginal depression; basal groove evenly and briefly curved to angulate humerus at juncture with broader lateral marginal depression, MEW/HuW = 2.21–2.38; parascutellar striole with 3–5 isolated punctures, the striole shallow between punctures; sutural interval coplanar with lateral intervals basally, upraised in apical half; sutural and striae 2–4 of equal development on disc, but sutural stria the only one complete to base and apex, the basal part shallow and punctate, the apical portion finely incised, smooth and deep; stria 5 traceable only as a series of shallow punctures in basal half, striae 6–7 absent; elytral apex evenly and broadly convex, the lateral surface overlying the deep 8^th^ stria; 2 dorsal elytral setae at 0.33–0.36× and 0.54–0.60× elytral length, setal impressions small, spanning ¼ of interval 3; lateral elytral setae arranged in an anterior series of 7 setae and a posterior series of 6 setae; elytral marginal depression narrow throughout, edge upturned at humerus, beaded laterally to subapical sinuation; subapical sinuation shallow, symmetrical. *Mesepisternum* with ~11 punctures in 2–3 rows; metepisternal width to length ratio = 0.71; metepisternum/metepimeron suture distinct. *Abdomen* with smooth glossy ventrites, round lateral depressions on ventrites 3–6; suture between ventrites 2 and 3 effaced; apical male ventrite with 2 marginal setae and apical female ventrite with 4 equally spaced marginal setae plus trapezoid of 4 subequal, short setae. *Legs*-metatarsomere 1/metatibial length ratio = 0.20; metatarsomere 4 length along outer lobe 1.5× medial tarsomere length, apical and subapical setae present; metatarsal dorsolateral sulci narrow, lateral, upper surface granulate without carina. *Microsculpture* of vertex an obsolete transverse mesh, sculpticell breadth 2× length; pronotal disc and base glossy; elytral disc glossy, indistinct transverse lines visible through the reflective sheen; elytral apex glossy, surface irregular but no sculpticells visible; metasternum glossy; laterobasal abdominal ventrites glossy, indistinct sculpticells in lateral depressions. *Coloration* of vertex rufous with a piceous cast; antennomeres 1–4 rufoflavous, 5–11 slightly darker; pronotal disc rufous with piceous cast, lateral margins narrowly rufous, and base and apex broadly rufous; proepipleuron rufoflavous, proepisternum rufopiceous; elytral disc rufopiceous, sutural interval broadly rufous basally, rufoflavous to flavous apically; elytral margins with intervals 7–8 pale rufous basally, margin more broadly flavous in apical 1/3 where apex of interval 2 is also broadly flavous; elytral epipleuron flavous dorsally, darker ventrally, metepisternum rufopiceous; abdomen with ventrite 1–5 medially rufopiceous, ventrites 3–6 flavous marginally, the apical half of ventrite 6 flavous; metafemur flavous; metatibia flavous with brunneous cast.

**Male genitalia** (n = 1). Aedeagal median lobe gracile, distance from parameral articulation to tip 4.5× depth at midlength (Fig. 134A); apex sinuously extended to a dorsoventrally expanded knoblike tip; median lobe curved to right beyond ostial opening in ventral view; internal sac with small dorsal ostial microtrichial patch based on uneverted specimen; flagellar plate short, length 0.32× parameral articulation-tip distance.

**Female reproductive tract.** The female allotype was not dissected.

##### Holotype.

Male (CUIC) dissected and labeled: HI: Maui Haleakala N.P. / Kipahulu Vy. Central / Pali tr. 910 m el. / 30-IV-1991 beating / vegetation at night // J.K. Liebherr / A.C. Medeiros, / Jr. collectors // Mecyclothorax / aquilus / ♂1 / det. J.K. Liebherr 2014 // HOLOTYPE / Mecyclothorax / aquilus / Liebherr / det. J.K. Liebherr 2015 (black-margined red label).

##### Allotype.

Female (CUIC) with same data as holotype.

##### Etymology.

The Latin adjective aquilus—dark-colored, blackish, dun, swarthy (Brown 1956)—is used to signify the dark body coloration representative of this species.

##### Distribution and habitat.

*Mecyclothorax aquilus* is known only from two specimens collected at 915 m elevation in Kīpahulu Valley (Fig. 135). These were collected by sifting moss and leaf litter, and by beating vegetation at night. The 915 m elevation collecting site has large stature koa trees and a high diversity of understory vegetation.

#### (101) Mecyclothorax insolitus

Taxon classificationAnimaliaColeopteraCarabidae

(Sharp)

**Figure 136.**
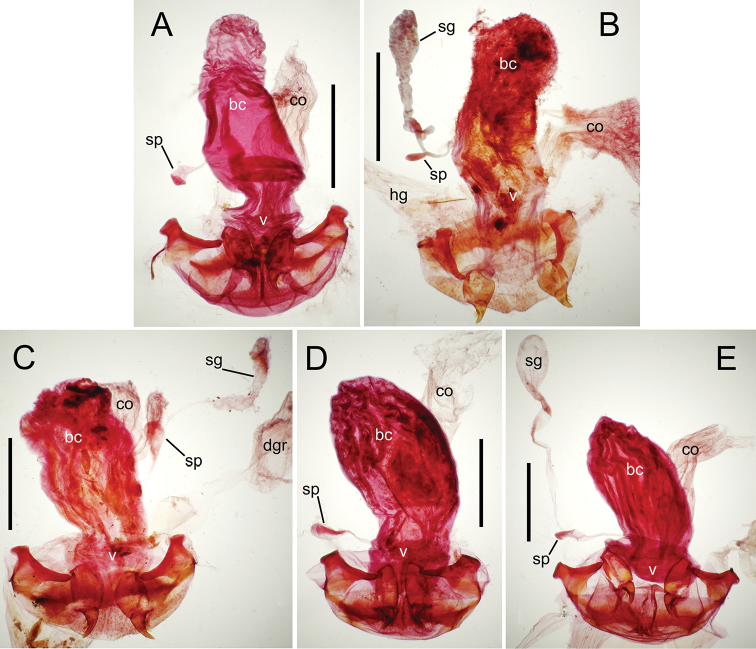
Female bursa copulatrix and associated reproductive structures, *Mecyclothorax ducalis* group species, ventral view (for abbreviations see Table 2, p. 23). **A** *Mecyclothorax insolitus* (Waikamoi, 1305 m) **B** *Mecyclothorax invisitatus* (Opana, 1265 m) **C** *Mecyclothorax ducalis* (Olinda, 1210 m) **D** *Mecyclothorax longidux* (Ke‘anae, 1325 m) **E** *Mecyclothorax brevidux* (Kīpahulu, 2045 m). Scale bar = 0.50 mm.

**Figure 137.**
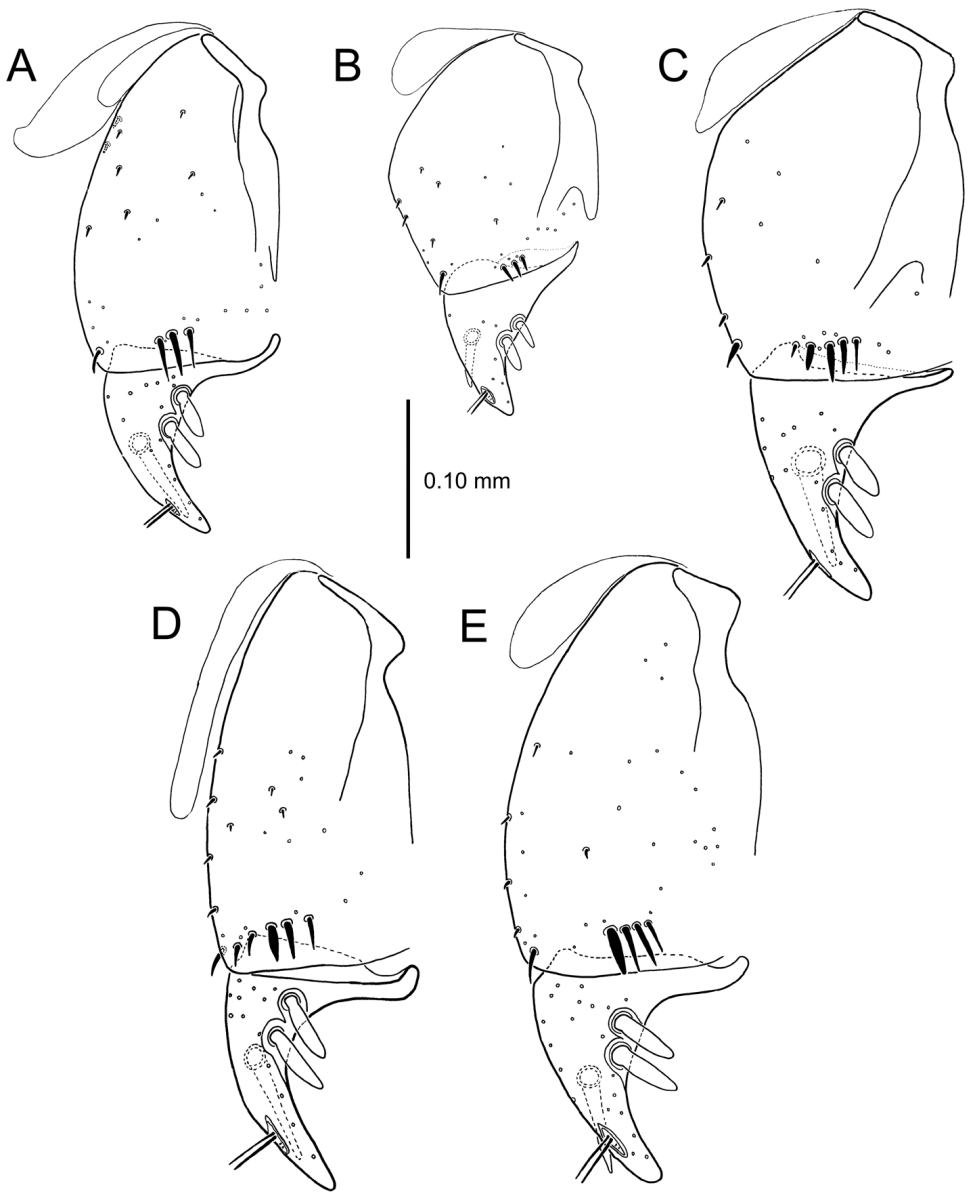
Left female gonocoxa, *Mecyclothorax ducalis* group species, ventral view. **A** *Mecyclothorax insolitus* (Waikamoi, 1305 m) **B** *Mecyclothorax invisitatus* (Opana, 1265 m) **C** *Mecyclothorax ducalis* (Olinda, 1210 m) **D** *Mecyclothorax longidux* (Ke‘anae, 1325 m) **E** *Mecyclothorax brevidux* (Kīpahulu, 2045 m).

Figs 133B , 134B–K , 135 , 136A , 137A

Thriscothorax insolitus Sharp 1903: 261.
Mecyclothorax insolitus , Britton 1948b: 141.

##### Diagnosis.

This species (Fig. 133B) and *Mecyclothorax invisitatus* (Fig. 133C) comprise a cryptic sibling species pair that can be best diagnosed externally by the breadth of elytra relative to the pronotum. In this species the elytra are broader, subovoid, with MEW/MPW = 1.63–1.69, whereas in *Mecyclothorax invisitatus* recorded values are 1.52–1.62. The pronotal disc bears obsolete transverse-mesh and transverse-line microsculpture in this species, whereas the disc surface is glossy in *Mecyclothorax invisitatus*. As there are no other reliable external characters that can separate these species, their recognition lies heavily on the differing configurations of the male aedeagal median lobe. Males of this species exhibit a median lobe with the apex expanded both dorsally and ventrally, and more flattened apically (Fig. 134F–K). The apex may be displaced dorsoventrally relative to the more basal portion of the shaft giving it a “prehensile proboscis” appearance. Conversely, the median lobe of *Mecyclothorax invisitatus* males is expanded only dorsally into a “crochet hook” apex, with the apical margin evenly rounded; a conformation also observed in males of *Mecyclothorax reiteratus* (Fig. 107). In the specimens available, the elytral apex is less broadly flavous in *Mecyclothorax insolitus* (Fig. 133B) compared to *Mecyclothorax invisitatus* (Fig. 133C), but the diagnostic value of this feature should be confirmed through examination of the male median lobe apex. Setal formula 2 1 2 0. Standardized body length 4.2–5.1 mm.

##### Identification

(n = 5). [Only non-diagnostic values for the various recorded ratios are presented for this species, as the qualitative attributes not in the above diagnosis are presented under *Mecyclothorax invisitatus*.] Eyes moderately convex, ocular lobe protruded obtusely from gena; ocular ratio = 1.52–1.56, ocular lobe ratio = 0.82–0.84. Pronotum moderately transverse, MPW/PL = 1.17–1.24, pronotal apical width variably subequal to basal width, APW/BPW = 0.92–1.07. Elytra broadly subovoid, MEW/HuW = 2.33–2.51.

**Male genitalia** (n = 13). Aedeagal median lobe gracile, distance from parameral articulation to tip 4.0–4.3× depth at midlength (Fig. 134B, D, F–G, I, K); median lobe apex variable, a very short knoblike tip (Fig. 134B, D), a more hooklike tip that includes a dorsal tooth (Fig. 134G, I, K), or a dorsoventrally expanded tip intermediate to those two configurations (Fig. 134F) (in all conformations the ventral margin is expanded at the tip); median lobe curved approximately 60° rightward apically in ventral view (Fig. 134C, E, J), the terminal knob or hook defining different profiles of the tip; internal sac with dorsal ostial microtrichial patch composed of stout macrospicules on right side (Fig. 134F, H), and ventral ostial microtrichial patch on left-ventral side (Fig. 134H), the sac otherwise covered with indistinct spicules; flagellar plate elongate, length 0.50× parameral articulation-tip distance. That the variation in tip configuration is infraspecific is attested to, in part, by sympatry of males exhibiting the knob configuration (Fig. 134D) and the dorsoventrally expanded tip (Fig. 134F) at Kula Pipeline Rd. near Waikamoi Gulch. The hooked aedeagal tip is observed in the eastern portion of the range, from Ko‘olau Gap/Ke‘anae Valley to Kuhiwa Valley, however it varies from exhibiting a straight ventral margin (Fig. 134G, I), to an angled margin (Fig. 134K). Similarly, the knob configuration varies within the Waikamoi sites (Fig. 134B, D, H). This instability in the aedeagal median lobe apex configuration within sites is coupled with lack of any diagnosable differences in external characters, aedeagal curvature (Fig. 134C, E, J), or spiculation of the internal sac (Fig. 134F, H) for specimens assigned to this species. This combination of observations supports circumscription of *Mecyclothorax insolitus* as a species that exhibits variability in the configuration of the aedeagal median lobe apex.

**Female reproductive tract** (n = 1). Bursa copulatrix cylindrical, with skirted basal constriction, smooth middle portion, and thinly wrinkled apex, length 1.08 mm, apical width 0.51 mm, skirted basal constriction 0.26 mm broad (Fig. 136A); bursal walls translucent, base and middle portion stained more intensely, thicker; gonocoxite 1 with 3 apical fringe setae, a curved seta at medioapical angle and 5–6 smaller setae on medial surface (Fig. 137A); gonocoxite 2 subacuminate with elongate, curved basal extension, 2 lateral ensiform setae, apical nematiform setae on medioventral surface at 0.70× gonocoxite length.

##### Holotype.

Female (BMNH) labeled: Thriscothorax insolitus Type D.S. Haleakala Perkins 845 // Type // Hawaiian Is. Perkins 1904-336. // HOLOTYPE Thriscothorax insolitus Sharp J.K. Liebherr 1998 (black-margined red label).

##### Distribution and habitat.

*Mecyclothorax insolitus* is distributed across windward Haleakalā from the Waikamoi area east to Kuhiwa Valley (Fig. 135). The species is known from low to moderate elevations; 880–1470 m. It has been collected in association with koa, ‘ōhi‘a, and *Cibotium* (hāpu‘u), though it has been also collected in leaf litter siftate, from a steep streambank, and under boards in ‘Ōhi‘a Wet Forest along Waikamoi Flume, 1310 m elevation.

#### (102) Mecyclothorax invisitatus sp. n.

Taxon classificationAnimaliaColeopteraCarabidae

http://zoobank.org/F361174B-0FCB-479F-B456-B7A06AE0C81F

**Figure 138.**
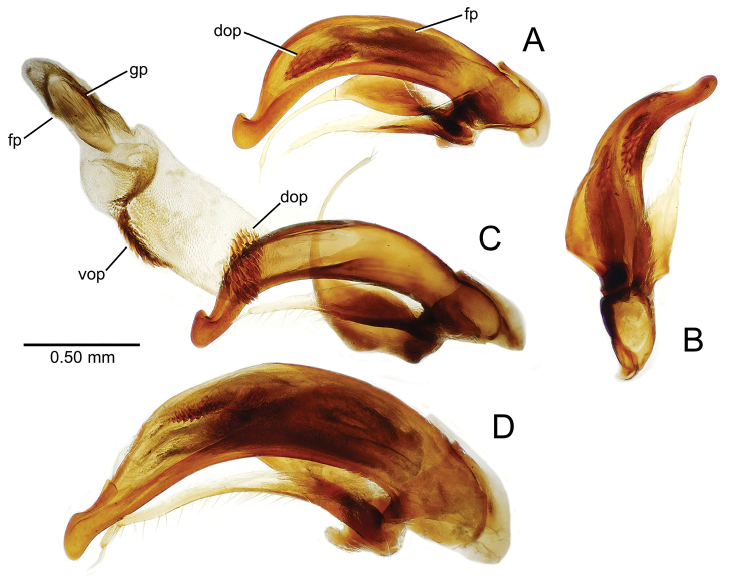
Male aedeagus, *Mecyclothorax ducalis* group species (for abbreviations see Table 2, p. 23). **A–C** *Mecyclothorax invisitatus*. **A–B** Right and ventral views (Opana, 1265) **C** Right view, sac everted (Waikamoi, 1305 m) **D** *Mecyclothorax ducalis*, right view (“Haleakala, 1902”, RCLP).

**Figure 139.**
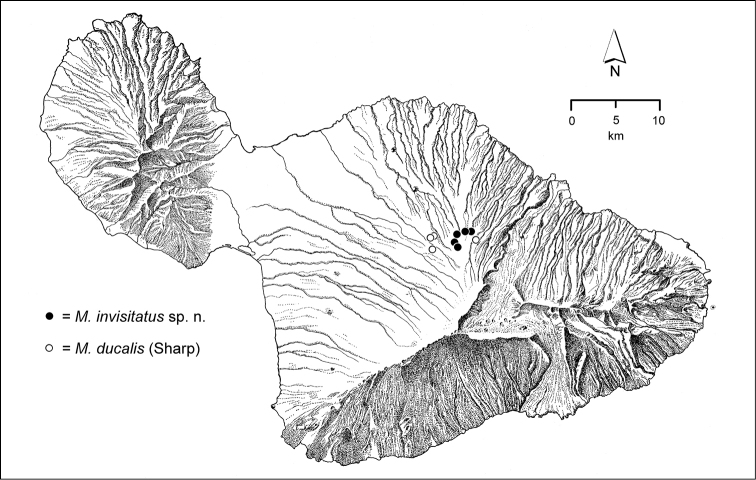
Recorded geographic distributions of *Mecyclothorax ducalis* group species.

Figs 133C , 136B , 137B , 138A–C , 139

##### Diagnosis.

Among the Haleakalā members of the *Mecyclothorax ducalis* group, this species is diagnosable by: 1, pronotum with lateral seta present; 2, elytra with apical margins flavous, contrasted to the rufopiceous disc; 3, pronotal disc glossy, microsculpture absent; 4, elytra narrowly subovoid, MEW/MPW = 1.52–1.62. Externally the cryptic sibling species, *Mecyclothorax insolitus*, differs in the last two criteria, with male aedeagal median lobe configuration offering an additional diagnostic difference between the species. Males of this species have the median lobe apex expanded into a dorsal hook, with the apex rounded ventrally (Fig. 138A–C), whereas *Mecyclothorax insolitus* males have the median lobe apex expanded both dorsally and ventrally, with the apical margin straighter (Fig. 134F–K). Setal formula 2 1 2 0. Standardized body length 4.3–5.1 mm.

##### Description

(n = 4). *Head capsule* with frontal grooves broad and deep near clypeus, a broad lateral convexity anterad eye, and low carina mesad anterior supraorbital seta; dorsal impression of neck broad, shallow, the concavity visible in dorsal view; ocular lobe protruded obtusely from gena, eyes moderately convex, ocular ratio = 1.50–1.55, ocular lobe ratio 0.80–0.84; labral anterior margin moderately, angularly emarginate 1/6 of labral length; antennae filiform, antennomeres 2–3 with sparse pelage of short setae; mentum tooth with sides acute, apex rounded. *Pronotum* moderately transverse, MPW/PL = 1.15–1.24, constricted basally with lateral margins briefly sinuate anterad right to obtuse hind angles, MPW/BPW = 1.53–1.62; median base slightly depressed medially relative to disc, more so laterally, ~20 punctures each side isolated in glossy surface; basal margin distinctly convex between hind angles; median longitudinal impression finely incised, crossed by indistinct transverse wrinkles; anterior transverse impression shallow, broad, smooth, incised only mesad front angle; anterior callosity slightly convex, smooth; front angles slightly projected, tightly rounded; pronotal apical width subequal to slightly broader than basal width, APW/BPW = 1.0–1.05; lateral marginal depression obsolete, narrowly beaded except slightly broader at front angle and evenly elevated from laterobasal depression to apex of projected hind angle; laterobasal depression deep, narrow, continuous with lateral depression. *Proepisternum* with 5 minute punctures along hind marginal groove; prosternal process slightly convex medially but with broad upraised margin. *Elytra* convex, sides distinctly sloped to vertical juncture with lateral marginal depression; basal groove briefly curved to proximate, subangulate humerus at juncture with broader lateral marginal depression, MEW/HuW = 2.22–2.50; parascutellar striole with 5 punctures, striole shallow between punctures; sutural interval slightly more convex than lateral intervals basally, more upraised apically; discal striae 1–6 distinctly punctate, progressively shallower laterally, stria 7 a series of shallow punctures at midlength; sutural stria deep and smooth on elytral apex, striae 2–3 broad and very shallow there, lateral striae not visible; discal elytral intervals 2–4 moderately convex, outer intervals follow curvature of elytron; 2 dorsal elytral setae at 0.25× and 0.51–0.53× elytral length, setal impressions shallow, crossing ¼ of interval 3; lateral elytral setae arranged in anterior series of 7 setae and posterior series of 6 setae; elytral marginal depression narrow throughout length, edge upturned at humerus, beaded laterally to subapical sinuation; subapical sinuation shallow, symmetrical. *Mesepisternum* with ~14 punctures in 2–3 rows; metepisternal width to length ratio = 0.79; metepisternum/metepimeron suture distinct. *Abdomen* with irregular longitudinal wrinkles on ventrites 1–6, round lateral impressions on ventrites 3–6; suture between ventrites 2 and 3 effaced; male apical ventrite with 2 marginal setae and female apical ventrite with 4 equally spaced setae and median trapezoid of 4 subequal, short setae. *Legs*-metatarsomere 1/metatibial length ratio = 0.20; metatarsomere 4 length along outer lobe 1.3× medial tarsomere length, apical and subapical setae present; metatarsal dorsolateral sulci broad, shallow, median area subcarinate. *Microsculpture* of vertex an obsolete transverse mesh, sculpticell breadth 2× length; pronotal median base glossy medially, indistinct transverse sculpticells present laterally; elytral disc and apex glossy, microsculpture obsolete; metasternum with a transverse mesh; laterobasal abdominal ventrites with swirling isodiametric and transverse microsculpture. *Coloration* of vertex rufous; antennomeres 1–2 rufoflavous, 3–11 darker, rufobrunneous; pronotal disc rufous, lateral margins, base, and apex narrowly rufoflavous; proepipleuron flavous, proepisternum rufous with piceous cast; elytral disc rufopiceous, sutural interval rufobrunneous basally, rufoflavous apically, elytral margins with intervals 7–9 or 8–9 contrastedly paler, rufoflavous to flavous, apex (up to apical 0.2× length) flavous; elytral epipleuron flavous dorsally, piceous ventrally, metepisternum rufopiceous; abdomen with all ventrites piceous mediobasally, flavous laterally, apical ventrite with apical ¾ flavous; metafemur flavous; metatibia rufoflavous with brunneous cast.

**Male genitalia** (n = 2). Aedeagal median lobe gracile, distance from parameral articulation to tip 3.8× depth at midlength (Fig. 138A, C); apex hooklike with blunt dorsal projection and evenly convex tip, the ventral margin not expanded ventrally as in *Mecyclothorax insolitus* (Fig. 134D, F, G, K); median lobe apically curved rightward at approximately 60° angle in ventral view (Fig. 138C, E, J), tip narrowly rounded in this view; internal sac with heavily spiculated dorsal ostial microtrichial patch at base of sac, and ventral ostial microtrichial patch at midlength (Fig. 138C, F, H); flagellar plate large, length 0.51× parameral articulation-tip distance.

**Female reproductive tract** (n = 1). Bursa copulatrix columnar, narrow with rounded apex, length 1.14 mm, apical width 0.40 mm, minimal width 0.30 mm (Fig. 136B); bursal walls more translucent basally, more thickly wrinkled apically; gonocoxite 1 with 3 short apical fringe setae, a curved seta at medioapical angle and 4–5 setae on medial surface (Fig. 137B); gonocoxite 2 short and broad basally, triangular with lateral margin evenly expanded to base, 2 small lateral ensiform setae, apical nematiform setae on medioventral surface at 0.80× gonocoxite length (short, broad apex may be due to wear).

##### Holotype.

Male (NMNH) labeled: HI: Maui Haleakala / Waikamoi N.C.P. Maile / Rd. 2-V-1998 lot05 / 1435 m el. pyrethrum fog / mossy log D.A. Polhemus // 1 // HOLOTYPE / Mecyclothorax / invisitatus / Liebherr / det. J.K. Liebherr 2015 (black-margined red label).

##### Paratypes.

HI: Maui: Koolau For. Res., Koolau Flume Rd., wet forest, yellow pan trap, 1280 m el., vi-viii-2006, Leblanc (CUIC, 2; UHIM, 2), Kula Pipeline Rd., pyrethrin fog log, 1305 m el., 18-v-2003 lot 09, Polhemus (NMNH, 1), Makawao Flume Rd., ecotone forest, yellow pan trap, 1293 m el., vi-viii-2006, Leblanc (UHIM, 2); Waikamoi N.C.P., Maile Rd., pyrethrin fog mossy log, 1435 m el., 02-v-1998 lot 05, Polhemus (NMNH, 1), Maile Rd., under logs on ground, 1494 m el., 16-v-2003 lot 06, Liebherr (CUIC, 1).

##### Etymology.

This close relative of *Mecyclothorax insolitus* is given the name *Mecyclothorax invisitatus* to signify the two species’ affinity. The adjective insolitus is translated as unusual, uncommon, or strange (Brown 1956), whereas invisitatus can be translated as strange, new, uncommon (Brown 1956). Thus the new species name proposed here owes its origin to David Sharp’s (1903) use of insolitus for the former species.

##### Distribution and habitat.

*Mecyclothorax invisitatus* is restricted to forests in the Waikamoi area from 1280–1500 m elevation (Fig. 139). All collections to date have been in association with ground-level microhabitats; under or in downed logs, or in yellow-pan traps.

#### (103) Mecyclothorax ducalis

Taxon classificationAnimaliaColeopteraCarabidae

(Sharp)

Figs 133D , 136C , 137C , 138D , 139

Thriscothorax ducalis Sharp 1903:266; Swezey 1954: 7 (koa associate).
Mecyclothorax ducalis , Britton 1948b: 140.

##### Diagnosis.

This large bodied species, standardized body length 6.0–6.7 mm, also stands out from other species in the group by the dark glossy, rufopiceous body color and contrasting flavous femora and infuscated tibiae. The pronotum is less constricted basally than in the other Haleakalā species of the group, MPW/BPW = 1.43–1.46, and the elytra are broadly subquadrate, with laterally extended basal margins outside the tightly rounded humeri (Fig. 133D); MEW/HuW = 2.06–2.15. Given these beetles’ large body size, the elytral striae are not deeply impressed, with only the sutural and 2^nd^ striae impressed on the disc, and lateral striae 3–5 indicated by increasingly isolated punctures. Setal formula 2 1(0) 2 0; the lectotype lacks pronotal lateral setae, a second specimen lacks the left seta, with the right present, and the other two examined specimens have both right and left lateral setae present.

##### Identification

(n = 4). The head is transversely impressed dorsally between the hind eye margins, the constriction visible in dorsal view, and the eyes moderately convex; ocular ratio = 1.54–1.56, ocular lobe ratio = 0.77–0.83. The pronotal lateral margins are subparallel for ~0.13× pronotal length anterad the obtuse, projected hind angles; median base only sparsely punctate with ~12 small punctures each side. The much reduced microsculpture is distributed as: 1, vertex with obsolete transverse mesh, sculpticell breadth 2× length; 2, pronotal disc mostly glossy, with obsolete transverse mesh, sculpticell breadth 2–3× length in places; 3, median base glossy medially, indistinct transverse sculpticells laterally; 4, elytral disc glossy; 5, elytral apex glossy, with patches of indistinct transverse sculpticells. The femoral apex is slightly infuscated, and the basal ¾ of the anterior femoral face is covered with a distinct piceous cloud, leaving a transverse femoral stripe across the apical half.

**Male genitalia** (n = 1). Aedeagal median lobe large, gracile, distance from parameral articulation to tip 3.7× depth at midlength (Fig. 138D); apex narrowly expanded, with dorsal projection basad expanded ventral margin producing an oblique apex with rounded ventral tip; the internal sac exhibits both a dorsal and a ventral ostial microtrichial patch (uneverted specimen); flagellar plate elongate, length estimated as 0.5× parameral articulation-tip distance (uneverted specimen).

**Female reproductive tract** (n = 1). Bursa copulatrix vase shaped, broader apically, length 1.09 mm, apical breadth 0.50 mm, basal breadth 0.34 mm (Fig. 136C); bursal walls thickly wrinkled; gonocoxite 1 with 4–5 apical fringe setae, a moderate seta at apicomedial angle and 3–4 setae on medial surface (Fig. 137C); gonocoxite 2 subtriangular, apex tightly rounded, lateral margin straight near ensiform setae, base moderately extended laterally, 2 lateral ensiform setae, apical nematiform setae on medial surface at 0.75× gonocoxite length.

##### Lectotype.

Male (BPBM) hereby designated, labeled: Thriscothorax ducalis Type D.S. Haleakala Perkins 620 (♂ in pencil) // Type // Hawaiian Is. Perkins 1904-336 // LECTOTYPE Thriscothorax ducalis Sharp J.K. Liebherr (black-margined red label).

##### Distribution and habitat.

*Mecyclothorax ducalis* has a distribution restricted to the Waikamoi area in habitats ranging 1210–1525 m elevation (Fig. 139). It was collected during six different collecting dates, starting v-1896 with R.C.L. Perkins, and finishing 14-i-1926 with R.H. Van Zwaluwenberg. None of the collecting events have associated ecological data.

#### (104) Mecyclothorax longidux sp. n.

Taxon classificationAnimaliaColeopteraCarabidae

http://zoobank.org/691389E5-0ABC-44DA-B8F8-DA4EBEC48F9D

**Figure 140.**
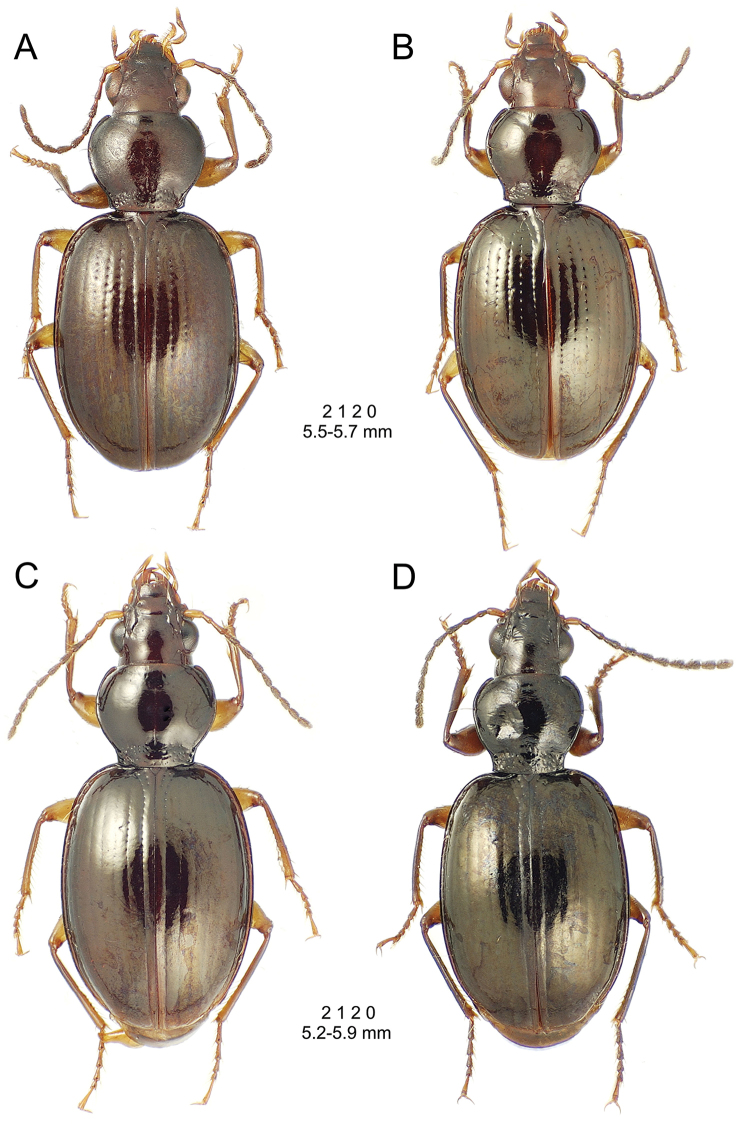
*Mecyclothorax ducalis* group species, dorsal habitus view. **A–B** *Mecyclothorax longidux*. A (Waikamoi, 1310 m) **B** (Kuhiwa, 1585 m) **C–D** *Mecyclothorax brevidux* **C** (Pu‘u Ahulili, 1600 m) **D** (Kīpahulu, 1800 m).

**Figure 141.**
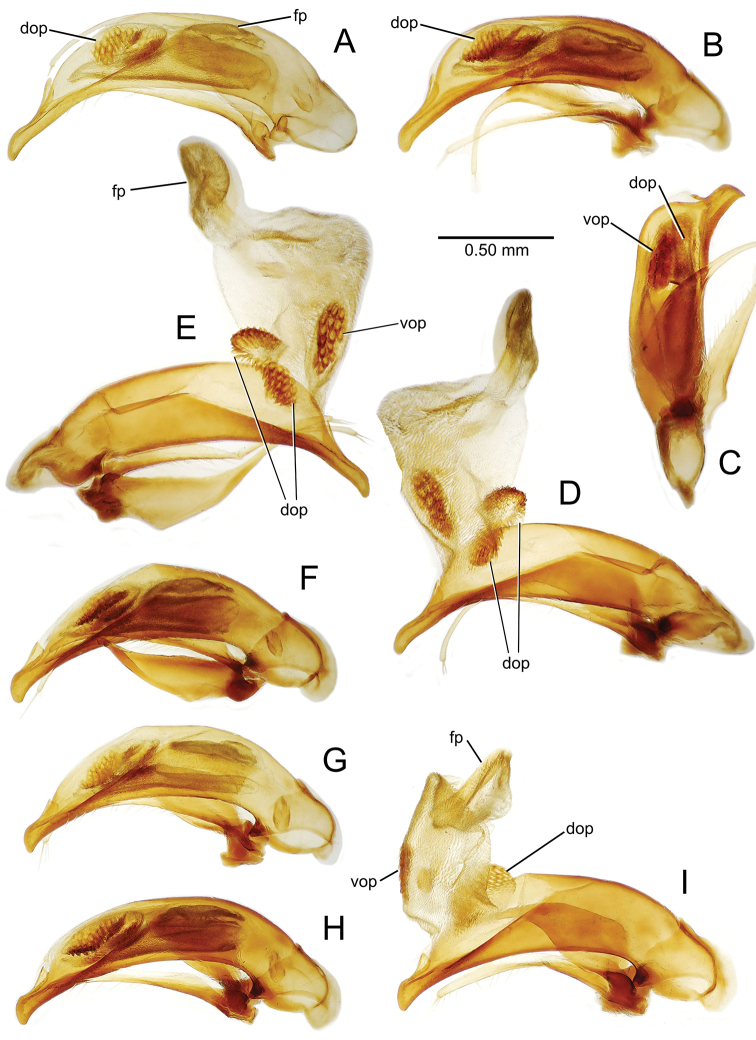
Male aedeagus, *Mecyclothorax ducalis* group species (for abbreviations see Table 2, p. 23). **A-E** *Mecyclothorax longidux*. **A** Right view (Waikamoi, 1310 m) **B–C** Right and ventral views (Kopili‘ula, 1127 m) **D–E** Right and left views, sac everted (Ke‘anae, 1325 m) **F–I** *Mecyclothorax brevidux* **F–H** Right views **F** (Kīpahulu, 1900 m) **G** (Kīpahulu W rim, 1850 m) **H** (Pu‘u Ahulili, 1600 m). **I** Right view, sac everted (Pu‘u Ahulili, 1600 m).

**Figure 142.**
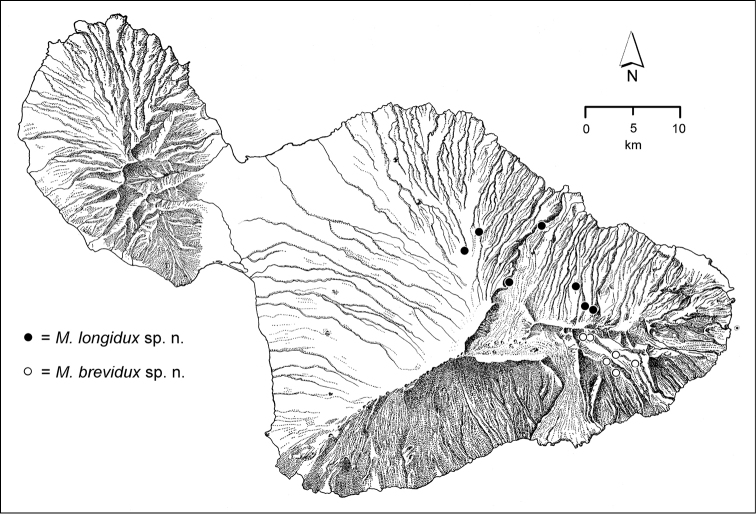
Recorded geographic distributions of *Mecyclothorax ducalis* group species.

Figs 136D , 137D , 140A–B , 141A–E , 142

##### Diagnosis.

This species (Fig. 140A–B) and the following, *Mecyclothorax brevidux* (Fig. 140C–D) are together diagnosed among Haleakalā species of the *Mecyclothorax ducalis* group by: 1, the bisetose pronotum; 2, dark rufous to rufopiceous elytra with concolorous apex; 3, basally constricted pronotum, MPW/BPW = 1.40–1.55 in this species; and 4, moderate body size, standardized body length 5.5–5.7 mm for this species. The elytral striae in individuals of this species are much more distinctly punctate, with the punctures distributed across the discal striae 1–5 where they expand strial breadth. The metafemur has the basal half of the anterior face covered with a piceous cloud, contrasted to the flavous medial coloration of the femur. In contrast, the metafemur in *Mecyclothorax brevidux* has only the basal 1/5 to 1/3 with piceous infuscation. In both species the femoral apex is darkened to match the rufobrunneous to rufopiceous tibial coloration. The male aedeagal median lobe confirms the value of these characters for diagnosis, with the lobe apex of *Mecyclothorax longidux* males narrow and elongate, with a slightly downturned tip (Fig. 141A–B, D), versus a shorter broader lobe apex in males of *Mecyclothorax brevidux* (Fig. 141F–I). The spiculation of the internal sac is also more developed in males of this species, with a bipartite dorsal ostial microtrichial patch present as well as a large, heavily spiculated ventral ostial microtrichical patch (Fig. 141D–E). Setal formula 2 1 2 0.

##### Description

(n = 5). *Head capsule* with frontal grooves deep, broad near clypeus, low broad lateral convexity anterad eye, terminated posteriorly mesad low ridge inside anterior supraorbital seta; dorsal impression of neck broad, shallow, visible in dorsal view; eyes large, moderately convex, ocular ratio = 1.53–1.63, ocular lobe ratio = 0.81–0.84; labral anterior margin angularly emarginate to 0.2× labral length; antennae filiform, antennomeres 2–3 with sparse pelage of short setae; mentum tooth with sides acute, apex tightly rounded. *Pronotum* little transverse, MPW/PL = 1.08–1.20; lateral margins subparallel to slightly convergent for 0.1× pronotal length anterad slightly obtuse, projected hind angles; median base moderately depressed relative to disc, ~25 minute, isolated punctures each side; basal margin distinctly convex between hind angles; median longitudinal impression finely incised, crossed by indistinct longitudinal wrinkles; anterior transverse impression shallow, broad, finely incised immediately mesad front angles; anterior callosity flat medially, smooth; front angles very slightly produced, tightly rounded; apical width subequal to basal width, APW/BPW = 0.99–1.02; lateral marginal depression obsolete, narrowly beaded laterally, broadly beaded basally, narrowly expanded inside front angle; laterobasal depression with irregularly punctured surface, margined by narrow U-shaped depression. *Proepisternum* with 5 minute punctures along hind marginal groove; prosternal process with broad median depression, lateral margins broadly upraised. *Elytra* subovoid, disc convex, slides sloped to nearly vertical juncture with lateral marginal depression; basal groove slightly curved to subangulate humerus at juncture with broader lateral marginal depression, MEW/HuW = 2.23–2.43; parascutellar striole with 4–5 elongate punctures, striole continuous between punctures; sutural interval more convex than lateral intervals 2–4, sutural juncture upraised; sutural and 2^nd^ striae of subequal depth and similar punctation on disc, 2^nd^ stria reduced in depth and punctation both basally and in apical 1/3 of length; discal striae 2–7 progressively shallower, impressed portions shorter, striae 6–7 reduced to a series of punctures, or stria 7 absent; striae 3–7 absent from elytral apex, surface evenly convex between striae 2 and 8; 2 dorsal elytral setae at 0.26× and 0.50–0.53× elytral length, setal impressions moderate, shallow, spanning ½ width of interval 3; lateral elytral setae arranged in anterior series of 7 setae and posterior series of 6 setae; elytral marginal depression; broad, explanate laterad humerus, narrowed and beaded laterally to subapical sinuation; subapical sinuation very shallow, nearly obsolete. *Mesepisternum* with ~11 punctures in 2–3 rows; metepisternal width to length ratio = 0.74; metepisternum/metepimeron suture distinct. *Abdomen* with irregular lateral wrinkles on ventrites 1–6, round lateral depressions on ventrites 3–6; suture between ventrites 2 and 3 effaced; apical male ventrite with 2 marginal setae and apical female ventrite with 4 equally spaced setae plus median trapezoid of 4 subequal, short setae. *Legs*-metatarsomere 1/metatibial length ratio = 0.19; metatarsomere 4 length along outer lobe 1.3× medial tarsomere length, apical and subapical setae present; metatarsal dorsolateral sulci narrow, lateral, upper surface granulate, broadly convex. *Microsculpture* of vertex an obsolete transverse mesh, sculpticell breadth 2× length, sculpticells most visible in depressed areas of cuticle; pronotal disc an obsolete transverse mesh, sculpticell breadth 2–3× length traceable over depressed portions of disc but surface mostly glossy; elytral disc and apex glossy, microsculpture obsolete; metasternum with shallow transverse mesh; laterobasal abdominal ventrites with swirling isodiametric and transverse microsculpture. *Coloration* of vertex rufous with a piceous cast; antennomere 1 rufoflavous, antennomeres 2–11 rufobrunneous; pronotal disc dark rufous with piceous cast, lateral margins, base and apex rufopiceous; proepipleuron rufobrunneous, proepisternum rufous; elytral disc dark rufous, sutural interval rufous basally, rufoflavous apically; elytral lateral marginal depression piceous basally, rufoflavous toward apex; elytral apex rufoflavous apicad subapical sinuation; elytral epipleuron rufoflavous, metepisternum rufobrunneous; abdominal ventrites 1–6 medially rufous with piceous cast, laterally rufoflavous, apical ¼ of ventrite 6 paler, rufoflavous.

**Male genitalia** (n = 4). Aedeagal median lobe gracile, distance from parameral articulation to tip 3.5–3.8× depth at midlength (Fig. 141A–B); apex narrowly extended 3× its depth beyond ostial opening, tip slightly downturned with apical face variably flattened (Fig. 141A–B, D); median lobe straight at midlength along shaft, apex offset toward right so that convex left margin is apical in ventral view (Fig. 141C); internal sac broadly expanded ventrally, a bipartite dorsal ostial microtrichial patch and ovoid ventral ostial microtrichial patch, each composed of heavily sclerotized macrospicules (Fig. 141D–E); flagellar plate of moderate size, length 0.35× parameral articulation-tip distance.

**Female reproductive tract** (n = 1). Bursa copulatrix sac-shaped, slightly constricted basally, length 1.14 mm, maximum breadth 0.64 mm, basal constriction 0.43 mm broad (Fig. 136D); bursal walls thick, broadly wrinkled; gonocoxite 1 with 3–5 apical fringe setae, a curved seta at medioapical angle and 5–7 setae on medial surface (Fig. 137D); gonocoxite 2 falcate with long basal panhandle, apex subacuminate, 2 narrow, moderately elongate lateral ensiform setae, apical nematiform setae on medioventral surface at 0.64× gonocoxite length.

##### Holotype.

Male (CUIC) dissected and labeled: HI: Maui Hanawi N.A.R. / Pig fence helipad sift / humus ex ohia 21-V- / 1993 lot 04 el. 1575 m // J.K. Liebherr & / A.C. Medeiros / Collectors // HOLOTYPE / Mecyclothorax / longidux / Liebherr / det. J.K. Liebherr 2015 (black-margined red label).

##### Paratypes.

HI: Maui: Hanawi N.A.R., Kopiliula Str., pyrethrin fog *Acacia koa* trunk, 1127 m el., 03-v-1998 lot 02, Liebherr (CUIC, 2), Kopiliula Str., uluhe fern under tent, 1125 m el., 03-v-1998 lot 01, Liebherr (CUIC, 1), Kuhiwa Vy., Poouli Cabin, beat vegetation, 1590 m el., 05-v-1998 lot 04, Ewing (CUIC, 2), southeast Keanae, Piinaau Road, 27-vi-1920, Bryan (BPBM, 1); Koolau For. Res. [= Hanawi N.A.R.], *Tetraplasandra dipyrena*, 1740 m el., 09-viii-1973, Gagné (BPBM, 1), Koolau Gap, Halehaku [= Ke‘anae Valley], beat ferns at night, 1325 m el., 13-v-1998 lot 09, Liebherr (CUIC, 2), beat *Rubus* (akala) beat at night, 1325 m el., 13-v-1998 lot 08, Liebherr (CUIC, 1), pyrethrin fog *Cibotium*/log, 1325 m el., 13-v-1998 lot 03, Liebherr (CUIC, 1), Kula Pipeline Rd., 975–1210 m el., 13-vi-1927, Swezey (BPBM, 1); Makawao For. Res., Maile Rd., scrape bark *Metrosideros*, 1310 m el., 26-v-1997 lot 08, Liebherr (CUIC, 1).

##### Etymology.

This and the following *Mecyclothorax brevidux* represent cryptic sibling species best diagnosed by the length of the male aedeagal median lobe (Fig. 141). As both are members of the *Mecyclothorax ducalis* species group, the Latin stem dux—a leader—is used for both species. In this species dux is combined with the Latin longus to form the noun longidux.

##### Distribution and habitat.

*Mecyclothorax longidux* is distributed across the windward face of Haleakalā from upper Kuhiwa Valley on the east to the Waikamoi forest on the west (Fig. 142), within an elevational range of 975–1740 m. To date all collecting series are small; either one or two specimens. The beetles have been collected in association with koa, ‘ōhi‘a, *Cibotium* (hāpu‘u), *Polyscias kavaiensis* (‘ohe‘ohe), and *Rubus* (‘ākala). Two specimens were found by beating ferns at night, and one was found in the uluhe fern (*Dicranopteris linearis*) platform mound built under my tent near Kopili‘ula Stream in the very wet Hanawī Natural Area Reserve (a technique of Perkins; see Evenhuis 2007: 34).

#### (105) Mecyclothorax brevidux sp. n.

Taxon classificationAnimaliaColeopteraCarabidae

http://zoobank.org/02338C86-031F-4248-997E-C1950C2FDD36

Figs 136E , 137E , 140C–D , 141F–I , 142

##### Diagnosis.

This species is extremely similar to *Mecyclothorax longidux* (Fig. 140A–B), but can be diagnosed from that species by the less punctate discal striae, with small punctures present in impressed striae 1–2 to 1–3, and striae 4–5 reduced to linear series of small punctures (Fig. 140C–D). The discal elytral intervals are less convex than in *Mecyclothorax longidux*, though the sutural interval is as upraised as in that species. The metafemur is darkened in its basal 1/5 to 1/3 of length, with the middle of the femur flavous, and apex narrowly piceous to match the darkened metatibia. The male aedeagal median lobe apex is shorter and broader (Fig. 141F–I) than in males of *Mecyclothorax longidux* (Fig. 141A–B, D). Setal formula 2 1 2 0. Standardized body length 5.2–5.9 mm.

##### Description

(n = 5). [The above description of *Mecyclothorax longidux* can serve to describe this species with the following substitutions.] *Eyes* with ocular ratio = 1.50–1.58, ocular lobe ratio = 0.81–0.84. *Pronotum* with lateral margins subparallel to slightly divergent for 0.1× pronotal length anterad slightly obtuse, projected hind angles; MPW/PL = 1.15–1.21, MPW/BPW = 1.51–1.63, APW/BPW = 1.0–1.07. *Elytra* subovoid, MEW/HuW = 2.20–2.49. *Microsculpture* of elytral disc and apex minimally developed, with indistinct isodiametric and transverse sculpticells visible through the glossy reflection.

**Male genitalia** (n = 4). Aedeagal median lobe gracile, distance from parameral articulation to tip 3.3–3.6× depth at midlength (Figs 141F–H); apex broad, extended twice maximum depth beyond ostial opening, apical face flattened, tip ventrally expanded so that ventral margin of lobe curves downward; internal sac short, broad, with lightly melanized dorsal ostial microtrichial patch and ventral ostial microtrichial patch (Fig. 141I); flagellar plate of moderate size, length 0.39× parameral articulation-tip distance (larger ratio of plate size relative to *Mecyclothorax longidux* due to shorter median lobe in *Mecyclothorax brevidux*; compare Fig. 141D, I)

**Female reproductive tract** (n = 1). Bursa copulatrix short, sac-shaped, length 1.0 mm, maximum width 0.51 mm, basal constriction 0.40 mm broad (Fig. 136E); bursal walls thick, broadly wrinkled; gonocoxite 1 with 4 apical fringe setae, a curved seta at medioapical angle and 5 setae on medial surface (Fig. 137E); gonocoxite 2 subacuminate, apex tightly rounded, basal extension curved at lateral terminus, 2 moderately elongate lateral ensiform setae, apical nematiform setae on medioventral surface at 0.74× gonocoxite length.

##### Holotype.

Female (BPBM) labeled: Kipahulu Valley / Maui, Camp 1 / 945 m, 6-12.VIII.67 // N. Wilson / Collector / BISHOP // 4 // HOLOTYPE / Mecyclothorax / brevidux / Liebherr / det. J.K. Liebherr 2015 (black-margined red label).

##### Paratypes.

HI: Maui: Haleakala N.P., Kekuewa Hill, 0.7 km N Puu Ahulili, sift humus/moss, 1600 m el., 16-v-1993 lot 02, Liebherr/Medeiros (CUIC, 4), lot 04 Liebherr/Medeiros (CUIC, 1), vegetation at night, 1600 m el., 16-v-1993 lot 08, Liebherr/Medeiros (CUIC, 1), Kipahulu Vy., sift litter, 1500 m el., 09-v-1991 lot 03, Jessel/Medeiros (CUIC, 1), 1800 m el., 08-v-1991 lot 04, Jessel/Medeiros (CUIC, 1), Mauka Ridge, pyrethrin fog *Metrosideros*/moss, 2045 m el., 21-v-1998 lot 01, Polhemus (NMNH, 2), Kipahulu west rim ESE Kuiki, sift *Metrosideros* humus, 1850 m el., 15-v-1993 lot 03, Liebherr/Medeiros (CUIC, 1).

##### Etymology.

This cryptic sister sibling species of *Mecyclothorax longidux* is given the epithet brevidux—short leader—in reference to the foreshortened aedeagal median lobe apex (Figs 141F–I).

##### Distribution and habitat.

*Mecyclothorax brevidux* is distributed in Kīpahulu Valley and on the Manawainui Planeze from 945–2045 m elevation (Fig. 142); a distribution allopatrically complementing that of *Mecyclothorax longidux* so that both species occupy most of Haleakalā’s windward forest. Most specimens of these beetles have been collected in moss, humus, or leaf litter in association with ‘ōhi‘a.

### *Mecyclothorax palustris* species group

**Figure 143.**
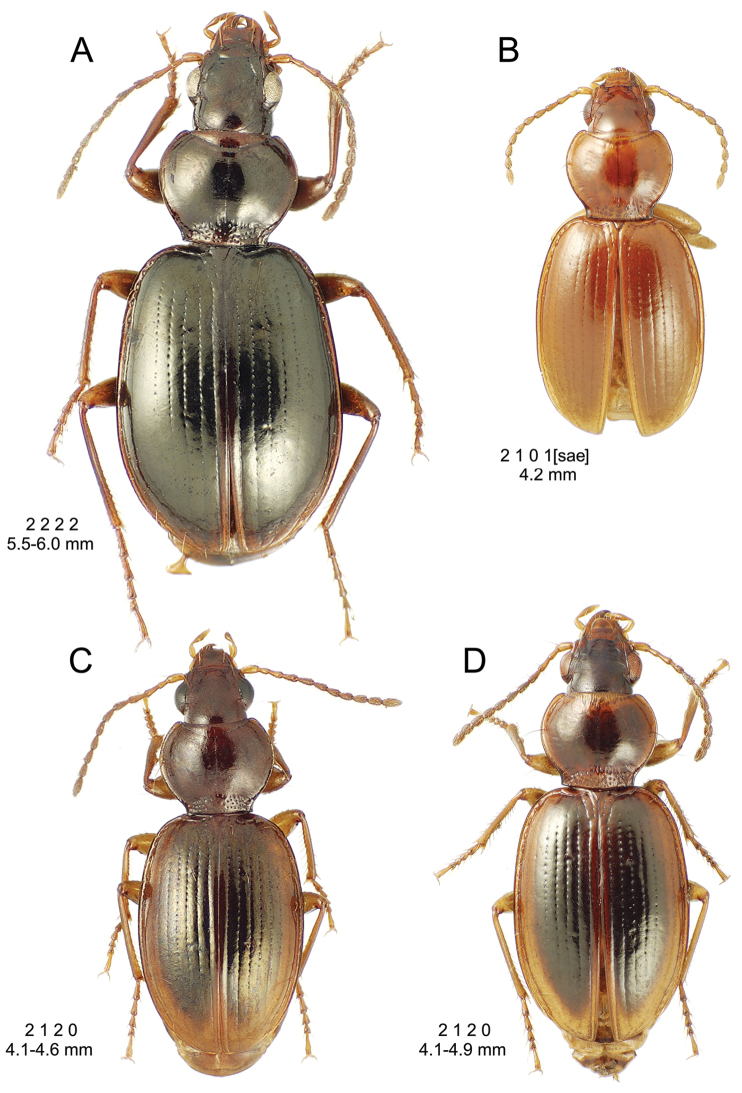
*Mecyclothorax palustris* group species, dorsal habitus view. **A** *Mecyclothorax hephaestoides* (Halemau‘u Tr., 2315 m) **B** *Mecyclothorax oculellus* (Honomanu, 1750 m) **C** *Mecyclothorax bicoloris*. (Ukulele Camp Pipeline, 1510 m) **D** *Mecyclothorax bicoloratus* (Helele‘ike‘oha, 1615 m).

**Diagnosis.** Analogous to the broadly based definition of the *Mecyclothorax ovipennis* group, species placed in the *Mecyclothorax palustris* species group are a disparate set of taxa united by a combination of broadly distributed characters. They all have: 1, lateral elytral striae 6–7 less developed than the medial elytral striae 1–5; 2, striae 1–3 subequally impressed and punctate on the disc; 3, the sutural stria deeper than stria 2 at the elytral apex; 4, elytral microsculpture evident and transverse; and 5, the pronotum basally constricted with the lateral margins sinuate before the well-developed hind angles. This array of taxa exhibits setal formulae 2 2 2 2, 2 1 2 1[sae], 2 1 2 0, and 2 1 0 1[sae]. Body size ranges from small to large, with standardized body lengths spanning 3.8–6.0 mm. Four of the species are characterized by marginally paler elytra (Figs 143C–D, 149A–B), and strial development ranges from shallow and impunctate (Fig. 149A) to deep and punctate (Fig. 143A, C–D). Even given this diversity of form within the group, several sets of cryptic sibling species are present, though the male aedeagal median lobe provides certain diagnosis in each instance.

**Membership and distribution.** There are 11 Haleakalā species assigned to the group. As a whole the species group attains the entire *Mecyclothorax* generic distribution across the Hawaiian archipelago, being known from O‘ahu to the Big Island of Hawai‘i. There are single species recorded from O‘ahu and Lāna‘i (Liebherr 2009a, b), three species from Moloka‘i (Liebherr 2007), and four each from West Maui (Liebherr 2011) and Hawai‘i Island (Liebherr 2008b). Haleakalā’s *Mecyclothorax hephaestoides* shares the full complement of supraorbital, pronotal and elytral setae with all four Big Island species plus *Mecyclothorax vulcanoides* Liebherr of West Maui. The Moloka‘i and O‘ahu species are characterized by reduced setation, with both apical and subapical elytral setae absent and the pronotum either glabrous or bisetose. This condition is shared with eight Haleakalā species. Based on these preliminary data, it would appear that this group diversified across a variously contiguous Maui Nui and O‘ahu Nui (Price and Elliott-Fisk 2004), with a single colonization event populating Hawai‘i Island. The species represent a diversity of ways of life, with Big Island’s *Mecyclothorax aa* Liebherr being the sole troglophilic Hawaiian *Mecyclothorax*. On Haleakalā, *Mecyclothorax hephaestoides* (Fig. 143A) has been found only in terrestrial microhabitats, either on open ground or along streams, whereas *Mecyclothorax bicoloris* and *Mecyclothorax bicoloratus* (Fig. 143C–D) have been recorded from terrestrial leaf litter and humus as well as from semiarboreal mossmats of larger trees.

#### Key to adults of the *Mecyclothorax palustris* species group, Haleakalā volcano, Maui, Hawai‘i

**Table:** 

1.	Body size smaller, standardized body length 3.9–5.5 mm; pronotum bisetose, lateral setae present and basal setae absent; elytral disc moderately to distinctly convex (Figs 143B–D, 149, 158); male aedeagal median lobe apex parallel sided, apically rounded (Figs 144D–I, 150, 152–153, 159, 161–162)	**2**
1’.	Body size larger, standardized body length 5.7–6.0 mm; pronotum quadrisetose, both lateral and basal setae present; elytral disc flattened (Fig. 143A); male aedeagal median lobe with broadly expanded, dorsally spinose apex (Fig. 144A–C)	(106) ***Mecyclothorax hephaestoides* sp. n.**
2(1).	Two dorsal elytra setae each side and parascutellar seta present; eyes larger, more convex, ocular ratio = 1.41–1.60 (smallest ratios in smallest individuals of *Mecyclothorax nanunctus* sp. n.; Fig. 149D)	**3**
2’.	Dorsal elytral setae and parascutellar seta absent (Fig. 143B); eyes reduced, ocular ratio = 1.43	(107) ***Mecyclothorax oculellus* sp. n.**
3(2)	Elytra distinctly bicolored, lateral intervals 7–9 and apex broadly flavous, pale margin contrasted with dark brunneous to piceous disc (Figs 143C–D, 149A–B)	**4**
3’	Elytral coloration more uniform, at most apex very narrowly paler and lateral margins gradually paler relative to disc (Figs 149C–D, 158)	**7**
4(3)	Elytral striae 1–5 impressed and distinctly punctate, associated intervals moderately convex (Fig. 143C–D)	**5**
4’	Elytral striae 1–2 to 1–4 impressed, but never distinctly punctate, the associated intervals flat (Fig. 149A–B)	**6**
5(4)	Eighth elytral interval apically expanded as a broad callus dorsad distal end of subapical sinuation; elytral disc with transversely stretched isodiametric microsculpture, the nearly isodiametric sculpticells arranged in transverse rows; male aedeagal median lobe apex with pointed tip (Fig. 144D, F–G)	(108) ***Mecyclothorax bicoloris* sp. n.**
5’.	Eighth elytral interval little convex dorsad distal end of subapical sinuation; elytral disc with distinctly transverse microsculpture, sculpticell breadth 2–3× breadth; male aedeagal median lobe with rounded tip (Fig. 144H–I)	(109) ***Mecyclothorax bicoloratus* sp. n.**
6(4)	Elytral striae 1 and 2 traceable on disc, stria 1 deep and punctured basally, continuous apically, stria 2 a shallow groove or series of isolated punctures basally, striae 3–6 intermittently traceable as isolated shallow grooves (Fig. 149A); elytral disc with papillate isodiametric microsculpture, the sculpticells uniformly isodiametric; male aedeagal median lobe with pointed tip (Fig. 150), internal sac bilobed (Fig. 150E–F)	(110) ***Mecyclothorax bilobatus* sp. n.**
6’	Elytral striae 1–6 traceable on disc, striae 1–2 continuous throughout length, minutely punctate in basal half, striae 3–6 traceable as shallow wavering lines or as isolated linear impressions (Fig. 149B); elytral disc with distinctly transverse microsculpture, the sculpticells isodiametric in transverse rows or with breadth 2× length; male aedeagal median lobe with rounded apex (Fig. 152A, C–D), internal sac with single lobe (Fig. 152G–H)	(111) ***Mecyclothorax palustroides* sp. n.** (in part)
7(3)	Pronotum and elytra similarly microsculptured, both covered with transverse-mesh microsculpture of varying development; elytral striae 1–3 to 1–6 evident, impressed and punctate, though punctures shallower and less regular on lateral striae (Figs 149D, 158)	**8**
7’	Pronotal and elytral microsculpture greatly contrasting, pronotum glossy with fine transverse lines traceable outside area of reflected light, elytra with well-developed isodiametric mesh, cuticle coriaceous, surface appearing dull (Fig. 149C); elytral striae 1–2 evident, punctate, striae 3–6 traceable as series of isolated linear impressions	(112) ***Mecyclothorax filipoides* sp. n.**
8(7)	Body coloration darker, head, pronotum and elytral disc dark brunneous to piceous, antennomeres 1–3 flavous, distinctly contrasted to smoky brunneous antennomeres 4–11 (Fig. 158)	**9**
8’	Body coloration paler, head, pronotum and elytral disc brunneous to rufous, pronotal and elytral lateromarginal depressions flavous, antennomeres 1–3 flavous, little contrasted with rufoflavous antennomeres 4–11 (Fig. 149D)	(113) ***Mecyclothorax nanunctus* sp. n.**
9(8)	Elytra narrowly ellipsoid to ovoid, humeri narrowly subangulate to angulate (Fig. 158C–E); pronotum narrower, MPW/PL = 1.08–1.21	**10**
9’	Elytra broadly ellipsoid, humeri broadly rounded (Fig. 158A–B); pronotum broader, MPW/PL = 1.22–1.33	(114) ***Mecyclothorax unctus* (Blackburn)**
10(9)	Pronotum median base distinctly depressed relative to disc, 19–24 punctures each side of midline (Figs 149B, 158E); male aedeagal median lobe extended beyond ostium (Figs 152, 161–162)	**11**
10’	Pronotal median base moderately depressed relative to disc, 12 minute punctures surrounded by glossy cuticle each side of midline (Fig. 158C–D); male aedeagal median lobe apex broadly rounded and little extended beyond ostium (Fig. 161A, C–D)	(115) ***Mecyclothorax tauberorum* sp. n.**
11(10)	Elytral disc (intervals 1–3) with upraised transverse microsculpture, sculpticells a mixture of isodiametric in transverse rows and transverse, breadth 2× length, individual sculpticells partially upraised, surface alutaceous; elytral intervals 8–9 posterad humerus variably flavous and much paler than brunneous disc, to flavobrunneous and only slightly paler than discal intervals; male aedeagal median lobe apex broad, with blunt dorsal expansion and rounded tip (Fig. 152)	(111) ***Mecyclothorax palustroides* sp. n.** (in part)
11’	Elytral disc (intervals 1–3) with shallow transverse microsculpture, sculpticells a mixture of isodiametric in transverse rows and transverse, breadth 2× length, adjacent sculpticells tiled, their surfaces flat, surface glossier; elytral intervals 8–9 posterad humerus only slightly paler, rufous, versus rufobrunneous disc, to concolorous with only ninth interval and lateral marginal depression paler; male aedeagal median lobe apex narrow, with slight dorsal expansion and tightly rounded tip (Figs 161–162)	(116) ***Mecyclothorax pau* sp. n.**

#### (106) Mecyclothorax hephaestoides sp. n.

Taxon classificationAnimaliaColeopteraCarabidae

http://zoobank.org/4794D8F3-BEC5-4CE4-9A9F-4CEE7686A4C0

**Figure 144.**
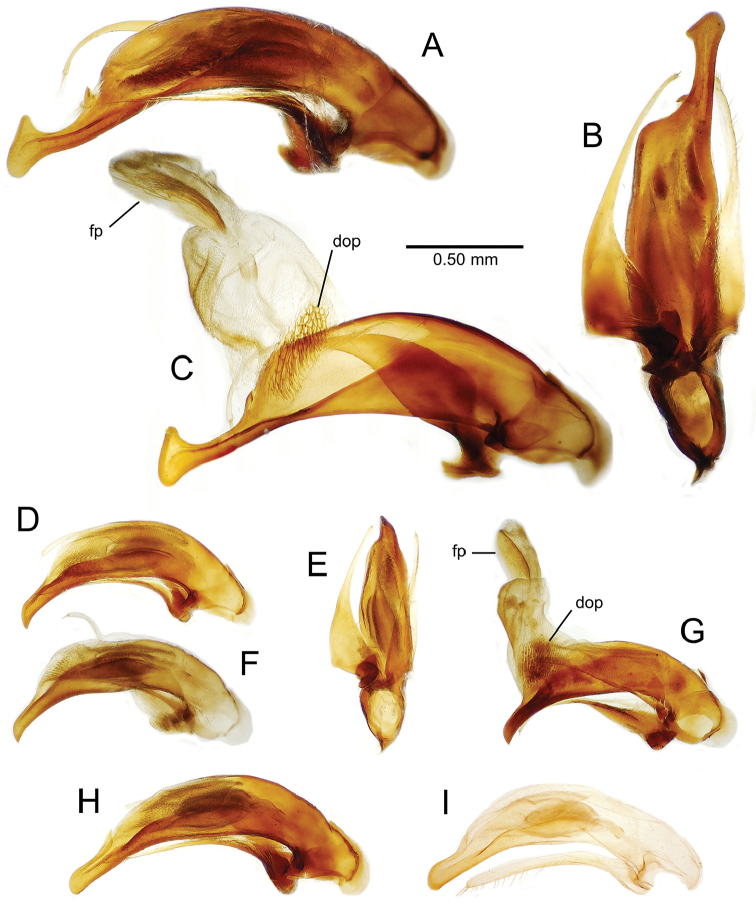
Male aedeagus, *Mecyclothorax palustris* group species (for abbreviations see Table 2, p. 23). **A–C** *Mecyclothorax hephaestoides* **A–B** Right and ventral views (Halemau‘u Tr., 2315 m) **C** Right view, sac everted (ESE Kuiki, 2145 m) **D–G** *Mecyclothorax bicoloris* **D–E** Right and ventral views (Ukulele Camp Pipeline, 1510 m) **F** Right view (Honomanu, 1700 m). **G** Right view, sac everted (Ukulele Camp Pipeline, 1510 m) **H–I** *Mecyclothorax bicoloratus*, right views (Helele‘ike‘oha, 1615 m).

**Figure 145.**
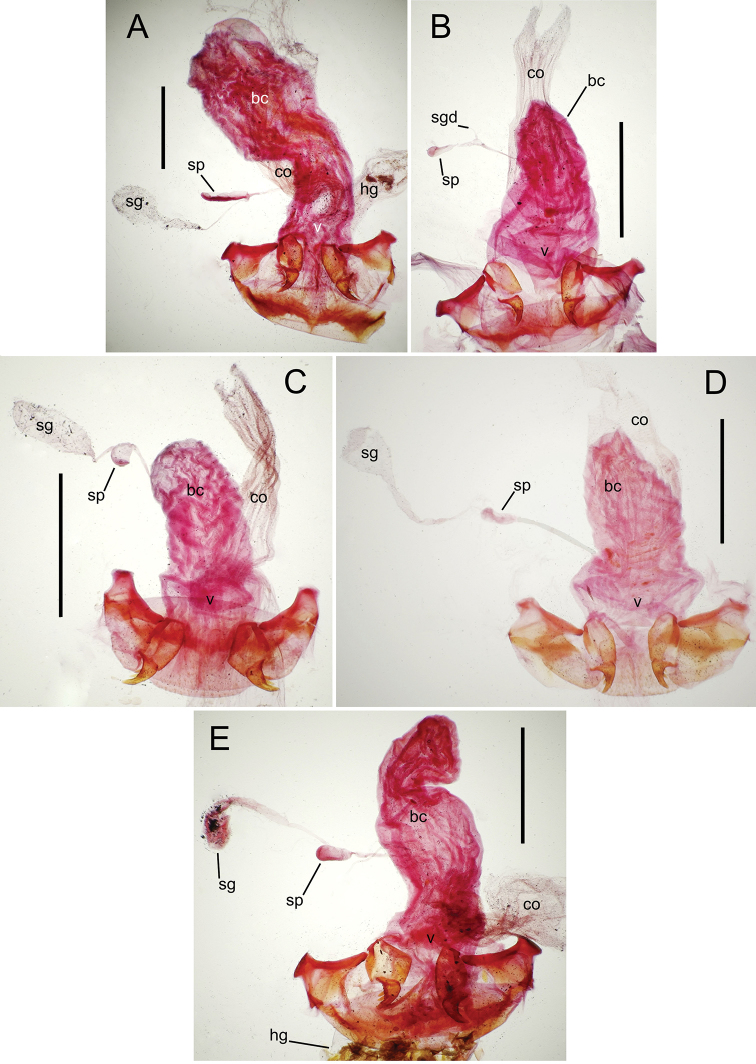
Female bursa copulatrix and associated reproductive structures, *Mecyclothorax palustris* group species, ventral view (for abbreviations see Table 2, p. 23). **A** *Mecyclothorax hephaestoides* (ESE Kuiki, 2145 m) **B** *Mecyclothorax bicoloris* (Honomanu, 1700 m) **C** *Mecyclothorax bicoloratus* (Helele‘ike‘oha, 1615 m) **D** *Mecyclothorax bilobatus* (Helele‘ike‘oha, 1615 m) **E** *Mecyclothorax palustroides* (Kīpahulu, 1960 m). Scale bar = 0.50 mm.

**Figure 146.**
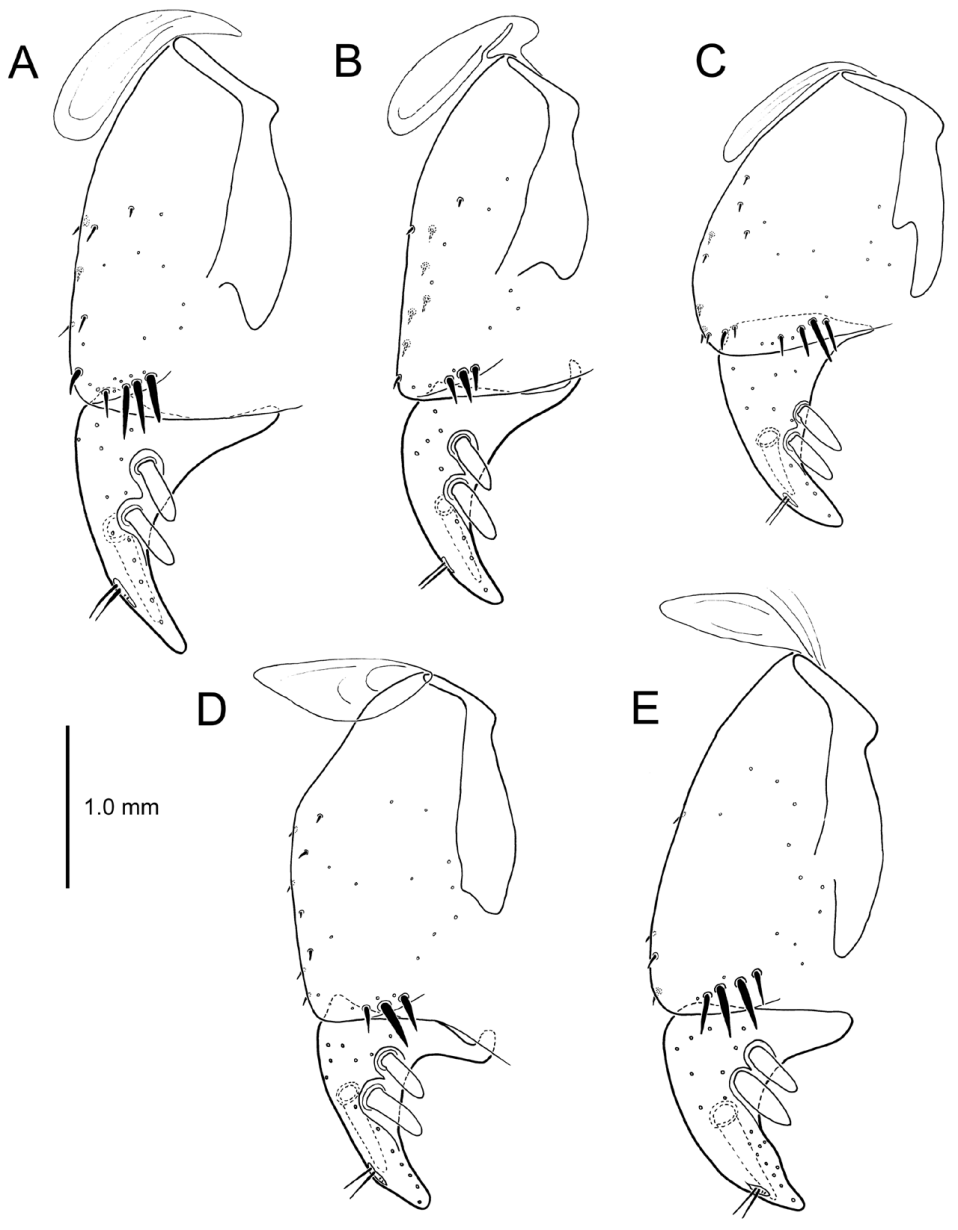
Left female gonocoxa, *Mecyclothorax palustris* group species, ventral view. **A** *Mecyclothorax hephaestoides* (ESE Kuiki, 2145 m) **B** *Mecyclothorax bicoloris* (Honomanu, 1700 m) **C** *Mecyclothorax bicoloratus* (Helele‘ike‘oha, 1615 m) **D** *Mecyclothorax bilobatus* (Helele‘ike‘oha, 1615 m) **E** *Mecyclothorax palustroides* (Kīpahulu, 1960 m).

**Figure 147.**
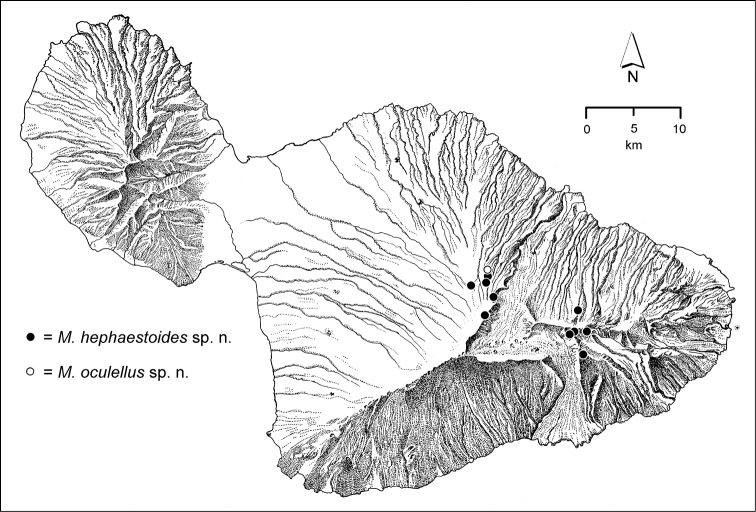
Recorded geographic distributions of *Mecyclothorax palustris* group species.

Figs 143A , 144A–C , 145A , 146A , 147

##### Diagnosis.

Beetles of this species are larger than all others assigned to this species group, standardized body length 5.7–6.0 mm (Fig. 143A), and are the only ones with a quadrisetose pronotum. Body coloration is uniformly dark, rufopiceous to rufobrunneous, with the elytral margins only narrowly rufoflavous. The discal elytral striae are distinctly punctate, with striae 1–3 impressed on the disc, and striae 4–5 indicated by more isolated punctures. Both the apical and subapical elytral setae are present. Setal formula 2 2 2 2.

##### Description

(n = 5). *Head capsule* with frontal grooves deep near clypeus, divergent mesad broad lateral convexity anterad eye, parallel mesad anterior supraorbital seta; dorsal impression of neck distinct, concave, visible in dorsal view; ocular lobe obtusely protruded from gena, eyes moderately convex, ocular ratio = 1.44–1.51, not covering posterior portion of ocular lobe, ocular lobe ratio = 0.76–0.81; labral anterior margin broadly emarginate to 1/6 of labral length; antennae filiform, antennomeres 2–3 with only a few setae basad apical rings; mentum tooth with sides acute, apex tightly rounded. *Pronotum* moderately transverse, MPW/PL = 1.20–1.25, lateral margins subparallel laterad basal pronotal setal articulatory sockets, hind angles obtuse, apex tightly rounded; median base depressed relative to disc, >20 distinct punctures each side, the punctures elongate along juncture with disc; basal margin broadly convex between laterobasal depressions; median longitudinal impression very fine, obsolete on disc, distinct just anterad median base; anterior transverse impression finely incised, deep, small punctures in deepest part; anterior callosity convex, smooth; front angles not produced, rounded posterad front margin; pronotal apical width subequal to pronotal basal width, APW/BPW = 0.99–1.07; lateral marginal depression moderately broad, reflexed, broader with minute bead at front angle; laterobasal depression deep, surface irregular, continuous with lateral depression. *Proepisternum* with 5 distinct punctures along hind marginal groove; prosternal process with broad median depression, lateral margins broadly beaded between coxae. *Elytra* subquadrate, disc moderately convex, sides evenly sloped to lateral marginal depression; basal groove subangulate at sutural stria, extended laterally to rounded humerus defined by a hitch at the juncture of narrow basal groove and broader lateral depression, MEW/HuW ratio = 2.06–2.12; parascutellar seta present; parascutellar striole with 5–7 punctures, striole shallow to coplanar with disc between punctures; sutural interval more convex than lateral intervals throughout length; sutural stria traceable to basal groove, deep and narrow apically, 2^nd^ stria obsolete basally, shallow and broad apically; discal striae 3–5 progressively shallower and less extended apically, all absent from base, stria 6 represented by a few very shallow punctures or absent, stria 7 absent; 8^th^ interval convex 7^th^ stria at position of subapical elytral seta; 2 dorsal elytral setae at 0.29× and 0.62–0.64 elytral length, setal impressions evident but spanning only ½ width of interval 3; apical and subapical setae present; lateral elytral setae arranged in anterior series of 7 setae and posterior series of 5–7 setae; elytral marginal depression narrow, edge slightly upraised at humerus, narrowed to bead at subapical sinuation; subapical sinuation shallow, more abruptly incurved apically. *Mesepisternum* with ~12 punctures in 2–3 rows; metepisternal width to length ratio = 0.69; metepisternum/metepimeron suture distinct. *Abdomen* with irregular lateral wrinkles on ventrites 1–6, lateral depressions on ventrites 3–6; suture between ventrites 2 and 3 complete; apical male ventrite with 2 marginal setae and apical female ventrite with 4 equally spaced setae and median trapezoid of 4 subequal, short setae. *Legs*-metatarsomere 1/metatibial length ratio = 0.17; metatarsomere 4 length along outer lobe 1.3× medial tarsomere length, apical and subapical setae present; metatarsal dorsolateral sulci narrow, lateral, median area broad. *Microsculpture* of vertex a shallow transverse mesh, sculpticell breadth 2–3× length; pronotal disc with indistinct elongate transverse mesh to transverse lines, transverse sculpticell breadth 2–4× length, median base with distinct transverse mesh between punctures, sculpticell breadth 2–3× length; elytral disc and apex with distinct, regular transverse mesh, sculpticell breadth 2–3× length; metasternum with obsolete transverse mesh, the surface glossy; laterobasal abdominal ventrites with swirling isodiametric and transverse microsculpture. *Coloration* of vertex rufobrunneous with piceous cast; antennomeres 1–3 rufoflavous, 4–11 rufobrunneous; pronotal disc rufopiceous, lateral margins narrowly, and base and apex more broadly rufobrunneous; proepipleuron rufobrunneous dorsally, rufoflavous along ventral margin, proepisternum dark rufobrunneous; elytral disc rufopiceous, sutural interval narrowly rufous along suture in basal 2/3, narrowly rufoflavous apically; elytral lateral marginal depression narrowly rufoflavous, elytral apex narrowly rufoflavous apicad subapical sinuation; elytral epipleuron dark rufoflavous, ventrally rufopiceous, metepisternum rufopiceous with cupreous reflection; abdominal ventrites 1–6 rufobrunneous mediobasally, rufoflavous on lateral and apical margins except apical 1/3 of ventrite 6 paler, rufobrunneous; metafemur rufoflavous with basal piceous cloud; metatibia rufoflavous with brunneous cast.

**Male genitalia** (n = 4). Aedeagal median lobe elongate, gracile, distance from parameral articulation to tip 4.2× depth at midlength (Fig. 144A, C); apex elongate, narrowly extended 5× its minimum depth beyond ostial opening, the tip expanded dorsally as a blunt tooth, and ventrally as rounded tip with oblique, straight, apical face; median lobe straight in ventral view, but apex offset to right side, dorsal (left) margin straight in ventral view, but apex offset to right side, dorsal (left) margin angled to meet thin apex (Fig. 144B); internal sac broad, breadth about 0.67× distance from ventral ostial margin to base of flagellar plate, a broad, transverse dorsal ostial microtrichial patch defined by shingled macrospicules present near base; flagellar plate moderately long, length 0.42× parameral articulation-tip distance.

**Female reproductive tract** (n = 1). Bursa copulatrix columnar with expanded apex, length 1.50 mm, apical breadth 0.68 mm, basal breadth 0.34 mm (Fig. 145A); bursal walls translucent, thickly wrinkled; gonocoxite 1 with 4–5 apical fringe setae, the medial seta smaller, a curved seta at medioapical angle and 6–7 smaller setae on medial surface (Fig. 146A); gonocoxite 2 falcate with subacuminate apex, broad basal extension little curved at lateral terminus, 2 lateral ensiform setae, apical nematiform setae on medioventral surface at 0.71× gonocoxite length.

##### Holotype.

Male (CUIC) dissected and labeled: HI: Maui Haleakala N.P. / Halemau‘u Tr. 5-V-1991 / el. 2270-2300 m / at night on ground // J.K. Liebherr / A.C. Medeiros, / Jr. collectors // 3 // Mecyclothorax / hephaestoides / ♂ #2 / det. J.K. Liebherr 2014 // HOLOTYPE / Mecyclothorax / hephaestoides / Liebherr / det. J.K. Liebherr 2015 (black-margined red label).

##### Paratypes.

HI: Maui: Haleakala N.P., Kalapawili Ridge nr. Pohaku Polaha, base bunch grass, 2505 m el., 11-x-2009 lot 01, Krushelnycky (UHIM, 1), Kipahulu Vy., sifting litter by day, 2100 m el., 07-v-1991 lot 05, Jessel/Medeiros (CUIC, 2), below Kuiki, pyrethrin fog mossy rockface, 2145 m el., 16-v-2001 lot 03, Liebherr (CUIC, 2), sift *Metrosideros* litter, 2145 m el., 16-v-2001 lot 02, Liebherr (CUIC, 1), Leleiwi overlook, under stone on ground, 2010–2100 m el., 11-vii-1919, Timberlake (UCRC, 1), NW upper slope, Halemauu Tr., on ground at night 2270–2300 m el., 05-v-1991 lot 03, Liebherr/Medeiros (CUIC, 1), under rocks nr. roots, 2285–2315 m el., 05-v-1991 lot 02, Liebherr (CUIC, 1), Waikamoi Gulch headwaters, moss along stream, 2030 m el., 06-v-1991 lot 01, Liebherr (CUIC, 1); Hana For. Res., Hanawi N.A.R., Kuhiwa Str. E Poouli Cabin, under rocks in streambed, 1615 m el., 06-v-1998 lot 03, Ewing (CUIC, 1); Koolau For. Res.; Waikamoi N.C.P., Honomanu drainage transect 3, ex mossy trunk *Metrosideros*, 1830–1860 m el., 07-v-1991 lot 07, Liebherr (CUIC, 5), upper arm Honomanu drainage, treading streamside vegetation, 1950 m el., 07-v-1991 lot 06, Liebherr (CUIC, 2).

##### Etymology.

This species resembles *Mecyclothorax vulcanus* (Blackburn) and *Mecyclothorax hephaestus* Liebherr of Hawai‘i Island (Liebherr 2008b), leading to use of hephaestoides as the species epithet.

##### Distribution and habitat.

*Mecyclothorax hephaestoides* exhibits a bipartite distribution with apparently disjunct populations occupying the Waikamoi area, and high elevations surrounding the head of Kīpahulu Valley (Fig. 147). This species occupies higher elevation forest and open subalpine habitats above timberline; i.e., 1615–2505 m elevation. Beetles of this species have been found under rocks in open rocky habitat within the fog zone, in moss along a stream, by treading emergent vegetation along a stream, in *Deschampsia* (hairgrass) tufts, and on a mossy rockface. The common element in all of these situations was the presence of free moisture, often at or just below the trade wind inversion layer on the volcano.

#### (107) Mecyclothorax oculellus sp. n.

Taxon classificationAnimaliaColeopteraCarabidae

http://zoobank.org/D3471827-6C31-4E9A-AD4C-4E4BCF112C35

Figs 143B , 147

##### Diagnosis.

This species is one of the few Hawaiian *Mecyclothorax* that lacks dorsal elytral setae. Among the Haleakalā fauna only *Mecyclothorax xestos* (Fig. 90D) of the *Mecyclothorax microps* group shares this feature. The parascutellar seta is absent, a unique occurrence within the *Mecyclothorax palustris* group, and the pronotum is broader relative to the elytra (Fig. 143B), MEW/MPW = 1.44, than in any other species with concolorous elytral disc and margins in the group. The bicolored *Mecyclothorax bicoloris* and *Mecyclothorax bicoloratus* exhibit similar somite proportions (Fig. 143C–D). The elytral microsculpture is a well-developed mesh of isodiametric sculpticells arranged in transverse rows on the inner intervals, the sculpticells more transverse on intervals 7 and 8. Setal formula 2 1 0 1[sae]. Standardized body length 4.2 mm.

##### Description

(n = 1). *Head capsule* with frontal grooves broadest near clypeus, expanded medially inside broad convexity anterad eyes, terminated posteriorly at thin carina mesad supraorbital seta; dorsal impression of neck slightly concave; ocular lobe barely protruded from gena, eyes relatively flat, ocular ratio = 1.43, ocular lobe ratio = 0.81; labral anterior margin angularly emarginate 1/6 labral length; antennae filiform, antennomeres 2–3 with sparse pelage of short setae; mentum tooth with sides acute, apex tightly rounded. *Pronotum* with lateral margins convex anterad a deep sinuation at the right hind angles, the lateral margins parallel for 1/6 pronotal length, MPW/PL = 1.21, MPW/BPW = 1.61; median base nearly coplanar with disc, shallow strigose wrinkles each side; basal margin very slightly convex between laterobasal depressions; median longitudinal impression very shallow, middle of disc flat; anterior transverse impression broad, shallow medially, more marked laterally, finely incised mesad front angles; anterior callosity moderately convex, smooth; front angles slightly projected, tightly rounded; pronotal apex broader than base, APW/BPW = 1.07; lateral marginal depression narrow, beaded laterally, edge upturned and broader at front angles; laterobasal depression smooth, continuous with lateral depression. *Proepisternum* with 5 minute punctures along hind marginal groove; prosternal process with broad median depression, lateral margins broadly beaded between coxae. *Elytra* subquadrate, moderately broad basally, disc flat, sides moderately sloped; basal groove angulate at sutural stria, extended to angulate humerus defined by juncture of basal groove and much broader lateral marginal depression, MEW/HuW = 1.95; parascutellar striole with 3–4 punctures, striole very shallow between punctures; sutural interval more convex than lateral intervals, sutural juncture upraised; sutural and 2^nd^ striae of subequal depth and punctation on disc, sutural stria deeper and more finely incised at apex; discal intervals 2–5 slightly convex, striae 2–5 progressively shallower and less punctate laterally, stria 6 a series of punctures, and stria 7 marked by sparse elongate irregularities at midlength, shallowly continuous apically; 8^th^ interval of similar convexity to apical fused portion of striae 5 + 7; subapical seta present in apical portion of stria 7 laterad apex of stria 2; lateral elytral setae arranged in anterior series of 7 setae and posterior series of 6 setae; elytral marginal depression moderately narrow posterad humerus, edge slightly upturned there, narrowed to a bead at subapical sinuation; subapical sinuation shallow, more abruptly incurved anteriorly. *Mesepisternum* with ~13 punctures in 2–3 rows; metepisternal width to length ratio = 0.76; metepisternum/metepimeron suture distinct. *Abdomen* with irregular lateral wrinkles on ventrites 1–5 and lateral depressions on ventrites 3–6; suture between ventrites 2 and 3 complete; apical female ventrite with 4 equally spaced setae and median trapezoid of 4 subequal, short setae; *Legs*-metatarsomere 1/metatibial length ratio = 0.19; metatarsomere 4 length along outer lobe 1.3× medial tarsomere length, apical and subapical setae present; metatarsal dorsolateral sulci broad, shallow, basal tarsomere medially subcarinate. *Microsculpture* of vertex a shallow transverse mesh, sculpticell breadth 2–3× length; pronotal disc with shallow transverse mesh, sculpticell breadth 2–4× length, median base with distinct isodiametric mesh; metasternum with a shallow transverse mesh; laterobasal abdominal ventrites with swirling isodiametric and transverse microsculpture. *Coloration* (lone holotype specimen is teneral) of vertex rufoflavous; antenna rufoflavous basally, dusky rufoflavous apically; pronotal disc rufoflavous, lateral margins narrowly, and base and apex flavous; proepipleuron flavous, proepisternum rufobrunneous with rufoflavous margins; elytral disc rufoflavous, sutural interval flavous basally and apically, concolorous on disc; elytral marginal depression narrowly flavous, apex gradually paler, flavous from apical terminus of interval 4; elytral epipleuron flavous, metepisternum rufobrunneous; abdominal ventrites 1–5 rufoflavous, apical half of ventrite 6 flavous; metafemur flavous; metatibia flavous with rufoflavous cast.

**Female reproductive tract.** The lone female specimen was not dissected.

##### Holotype.

Female (BPBM) labeled: HI: Maui Is. Haleakala / Waikamoi N.C.P. 1750 m el. / 20°47.21'N, 156°13.82'W / 12-III-2002 R. Takumi / pyr. fog mossy ohia // HOLOTYPE / Mecyclothorax / oculellus / Liebherr / det. J.K. Liebherr 2015 (black-margined red label).

##### Etymology.

The very small eyes characterizing this species are the basis for using oculellus—small eye—as the species epithet. The diminutive oculellus was used instead of ocellus due to the very specific meaning of the latter term in entomology.

##### Distribution and habitat.

*Mecyclothorax oculellus* is known only from a specimen collected at 1750 m elevation in the Honomanu drainage of Waikamoi Nature Conservancy Preserve (Fig. 147). The type specimen was collected in pyrethrin fog samples taken from mossy ‘ōhi‘a, along with specimens of 12 other *Mecyclothorax* spp.: *Mecyclothorax bicoloris*, *Mecyclothorax cognatus*, *Mecyclothorax filipoides*, *Mecyclothorax kipwilli*, *Mecyclothorax laetus*, *Mecyclothorax mauiae*, *Mecyclothorax orbiculus*, *Mecyclothorax ovipennis*, *Mecyclothorax perstriatus*, *Mecyclothorax planatus*, *Mecyclothorax sobrinus*, and *Mecyclothorax unctus*.

#### (108) Mecyclothorax bicoloris sp. n.

Taxon classificationAnimaliaColeopteraCarabidae

http://zoobank.org/255CB16D-17DD-4FBB-8053-AE92EC946A03

**Figure 148.**
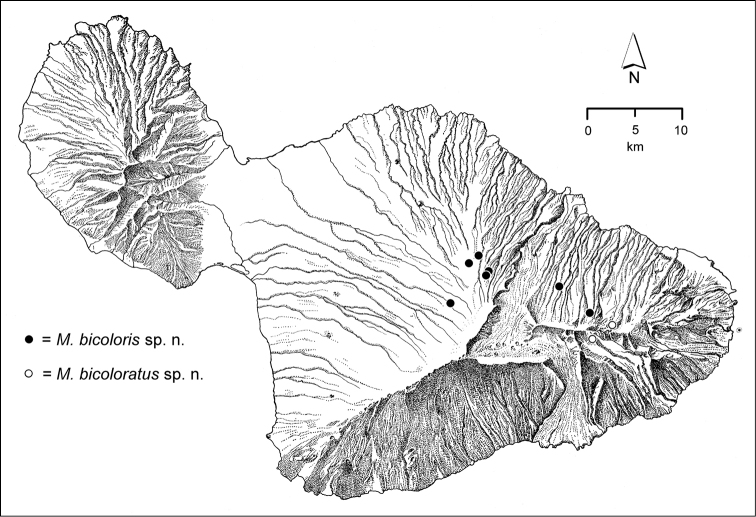
Recorded geographic distributions of *Mecyclothorax palustris* group species.

Figs 143C , 144D–G , 145B , 146B , 148

##### Diagnosis.

This species (Fig. 143C) and *Mecyclothorax bicoloratus* (Fig. 143D) comprise a cryptic sibling species pair diagnosed by: 1, broadly flavous elytral margins contrasted to a rufopiceous disc; and 2, punctate discal elytral striae 1–5. The strial punctures vary in size (Fig. 143C–D), though unfortunately for the taxonomist, not in tandem with the diagnostic male aedeagal median lobe. The median lobe apex for *Mecyclothorax bicoloris* males is ventrally pointed (Fig. 144D, F–G), whereas the lobe apex in *Mecyclothorax bicoloratus* males is broadly rounded (Fig. 144H–I). Two subtle external characters allow these two species to be diagnosed, though not with the confidence based on a male dissection. Firstly, the microsculpture of the vertex of *Mecyclothorax bicoloris* individuals comprises an indistinct transverse mesh, sculpticell breadth 2–3× length, with areas of microsculpture interspersed with glossy areas. The sculpticell margins are not visible in areas of reflected microscope light. *Mecyclothorax bicoloratus* individuals, in contrast, exhibit a well-developed transverse mesh on the vertex, with the convex surfaces of individual sculpticells discernible, and the entire surface less glossy. Secondly, the elytral lateral marginal groove is narrower basally just laterad the humeral angle in *Mecyclothorax bicoloris* (Fig. 143C), whereas it is broader with a more elevated margin in *Mecyclothorax bicoloratus* (Fig. 143D). Setal formula 2 1 2 0. Standardized body length 4.1–4.6 mm.

##### Description

(n = 5). *Head capsule* with frontal grooves deepest in anterior half of length where they are parallel on the frons, grooves narrower and shallower posterad a lateral jog in their direction, grooves terminated mesad narrow carina inside anterior supraorbital seta; dorsal impression of neck slightly concave; ocular lobe distinctly protruded from gena, eyes moderately convex and large, ocular ratio = 1.48–1.53, ocular lobe ratio = 0.85–0.91; labral anterior margin angularly emarginate 1/8 of labral length; antennae filiform; antennomeres 2–3 with sparse pelage of short setae; mentum tooth with sides acute, apex tightly rounded. *Pronotum* transverse, MPW/PL = 1.15–1.29, lateral margins distinctly sinuate for short distance anterad right to slightly acute hind angles, MPW/BPW = 1.56–1.70; median base distinctly depressed relative to disc, with ~20 small, isolated punctures each side; basal margin slightly convex medially, straight behind laterobasal depressions; median longitudinal impression very shallow, middle of disc flat, crossed by indistinct transverse wrinkles; anterior transverse impression broad, shallow, smooth, obsolete medially, incised immediately mesad front angles; anterior callosity nearly flat, slightly convex, smooth; front angles slightly projected, tightly rounded; pronotal apical width slightly greater than basal width, APW/BPW = 1.01–1.09; lateral marginal depression narrow, edge upturned anterad lateral seta, a bit broader at front angle; laterobasal depression narrow with irregular surface, continuous with lateral depression. *Proepisternum* with 5 minute punctures along hind marginal groove; prosternal process with narrow median impression, lateral margins broadly upraised. *Elytra* subquadrate, disc slightly convex, sides moderately sloped to margins; basal groove angulate at sutural stria, extended directly to angulate humerus at juncture of basal groove and broader lateral depression, MEW/HuW = 2.04–2.12; parascutellar seta present; parascutellar striole with 5 punctures, striole shallow but continuous between punctures; sutural interval coplanar basally, upraised apically; sutural and 2^nd^ striae of subequal depth and punctation on disc; sutural stria continued to base whereas striae 2–5 obsolete basally; at elytral apex sutural stria deep and smooth, striae 2–3 and 7 traceable apically but very shallow; discal striae 2–6 progressively shallower laterally, punctures more isolated and associated intervals flatter, stria 7 absent at elytral midlength; 8^th^ interval of similar convexity to apical fused portion of striae 5 + 7; 2 dorsal elytral setae at 0.26× and 0.65× elytral length, setal impressions small, shallow, spanning ½ width of interval 3; apical and subapical setae absent; lateral elytral setae arranged in anterior series of 7 setae and posterior series of 6 setae; elytral marginal depression narrow laterad humerus with upturned margin, narrower and beaded apically at subapical sinuation; subapical sinuation very shallow, symmetrical. *Mesepisternum* with ~9 punctures in 2–3 rows; metepisternal width to length ratio = 0.65; metepisternum/metepimeron suture distinct. *Abdomen* with irregular lateral wrinkles on ventrites 1–5, lateral depressions on ventrites 3–6; suture between ventrites 2 and 3 complete; apical male ventrite with 2 marginal setae and apical female ventrite with 4 equally spaced setae and median trapezoid of 4 subequal, short setae. *Legs*-metatarsomere 1/metatibial length ratio = 0.19; metatarsomere 4 length along outer lobe 1.3× medial tarsomere length, apical and subapical setae present; metatarsal dorsolateral sulci broad, shallow, basal tarsomere medially subcarinate. *Microsculpture* of pronotal disc shallow transverse mesh to transverse lines between areas of glossy cuticle, median base glossy between punctures, indistinct transverse sculpticells over parts of cuticle; elytral disc with transversely stretched isodiametric sculpticells, sculpticells up to 2× broad as long, apex with mixture of isodiametric and transverse sculpticells; metasternum with shallow transverse mesh; laterobasal abdominal ventrites with swirling isodiametric and transverse sculpticells. *Coloration* of vertex rufobrunneous; antennomeres 1–3 flavous, 4–11 rufoflavous; pronotal disc rufobrunneous, lateral margins, base, and apex rufoflavous; proepipleuron rufoflavous, proepisternum dorsally rufoflavous, ventrally rufobrunneous; elytral disc basally rufobrunneous to rufopiceous, lateral margins 7–9 contrastedly flavous, sutural interval rufoflavous basally, flavous apically; elytral apex broadly flavous, the paler area matching or exceeding the breadth of the pale lateral margin; elytral epipleuron flavous dorsally, rufoflavous ventrally, metepisternum rufobrunneous; abdomen with ventrites 1–3 rufobrunneous medially, rufoflavous laterally, ventrites 4–6 basally rufobrunneous, apically flavous; metafemur flavous; metatibia rufoflavous.

**Male genitalia** (n = 3). Aedeagal median lobe moderately stout, distance from parameral articulation to tip 3.0× depth at midlength (Fig. 144D, F); apex extended 2–3× its minimum depth beyond ostial opening, dorsal and ventral margins parallel until tip that is acutely angled and slightly downturned, the apical face convex; median lobe straight, right and left margins similarly convergent in ventral view, the apex with oblique, blunt tip in this view (Fig. 144E); internal sac about twice as long as broad, with broad, diffuse dorsal ostial microtrichial patch near base, and ventral sac surface covered with brownish microspicules (Fig. 144G); flagellar plate of moderate size, length 0.40× parameral articulation-tip distance.

**Female reproductive tract** (n = 1). Bursa copulatrix ovate—like an insect net in profile (Fig. 145B)—with length 0.72 mm, basal breadth 0.51 mm, midlength breadth 0.34 mm; bursal walls translucent, thickly wrinkled; gonocoxite 1 with 3–4 apical fringe setae, a curved seta at medioapical angle and 6–7 setae on medial surface (Fig. 146B); gonocoxite 2 falcate with subacuminate apex, basal extension curved at terminus, 2 lateral ensiform setae, apical nematiform setae on medioventral surface at 0.75× gonocoxite length.

##### Holotype.

Male (CUIC) labeled: HI: Maui Haleakala NW / slope Waikamoi Pres. / trans. 3 @ 1700 m el. / 10-IV-1991 sifting / litter J.K. Liebherr // 1 // HOLOTYPE / Mecyclothorax / bicoloris / Liebherr / det. J.K. Liebherr 2015 (black-margined red label).

##### Paratypes.

HI: Maui: Haleakala N.P., NW upper slope, beating, 1830–1980 m el., 18-viii-1937, Zimmerman (BPBM, 1); Hanawi N.A.R., Kopiliula Str., pyrethrin fog *Metrosideros*/moss/uluhe, 1127 m el., 03-v-1998 lot 08 Polhemus (CUIC, 1), Kuhiwa Vy., Poouli Cabin, pyrethrin fog *Metrosideros*/moss, 1590 m el., 06-v-1998 lot 06, Polhemus (NMNH, 1), pyrethrin fog *Metrosideros*/ roots/trunk, 1590 m el., 06-v-1998 lot 07, Polhemus (NMNH, 1); Koolau For. Res., Kula Pipeline Rd., pyrethrin fog *Acacia koa*, 1305 m el., 18-v-2003 lot 10, Polhemus (NMNH, 2); Waikamoi N.C.P., pyrethrin fog *Metrosideros*/moss, 1750 m el., 12-iii-2002, Takumi (BPBM, 1), Honomanu drainage, transect 3, sift moss and litter, 1680–1710 m el., 10-iv-1991 lot 01, Liebherr (CUIC, 1), 08-v-1991 lot 06, Kavanaugh (CAS, 3), scrape *Metrosideros* humus/moss, 1700 m el., 08-v-1991 lot 03, Liebherr (CUIC, 1), Ukulele Pipeline, pyrethrin fog *Metrosideros* mossy log, 1510 m el., 16-v-2003 lot 05, Liebherr (CUIC, 2).

##### Etymology.

The species epithet bicoloris refers to the bicolored elytra of members of this species. The epithet is the genitive singular form of bicolor, and its ending does not change with gender.

##### Distribution and habitat.

*Mecyclothorax bicoloris* is distributed on the windward face of Haleakalā from Kuhiwa Stream on the east to the Waikamoi forests on the west (Fig. 148). Localities range in elevation 1305–1980 m. The species was collected once in association with koa, but nearly all records are in association with ‘ōhi‘a; mossy trunks, mossy downed logs and exposed roots, or humus and leaf litter.

#### (109) Mecyclothorax bicoloratus sp. n.

Taxon classificationAnimaliaColeopteraCarabidae

http://zoobank.org/7AD61C28-DF55-4874-9ED6-A231A162D2CF

##### Diagnosis.

This species (Fig. 143D) shares the bicolored elytra, punctate discal elytral striae, and setal formula 2 1 2 0 with its cryptic sibling species, *Mecyclothorax bicoloris* (Fig. 143C). As detailed under that species’ treatment, the more intensely microsculptured vertex can externally diagnose individuals of this species, with the isodiametric sculpticells upraised, their individual reflections producing a textured look to the surface. Also, the elytral lateral marginal depression is broader laterad the humerus in this species (Fig. 143D). The aedeagus of this species has a broadly rounded median lobe apex (Fig. 144H–I) instead of the ventrally pointed apex of *Mecyclothorax bicoloris* males (Fig. 144D–G). Standardized body length 4.1–4.8 mm.

##### Description

(n = 5). [The above description of *Mecyclothorax bicoloris* can serve to describe this species. All diagnostic characters are presented above, so only the various recorded ratios are presented below.] *Eyes* moderately convex, covering much of the protruded ocular lobes, ocular ratio = 1.44–1.50, ocular lobe ratio = 0.79–0.84. *Pronotum* transverse, MPW/PL = 1.18–1.30, lateral margins distinctly sinuate for short distance anterad right to slightly acute hind angles, MPW/BPW = 1.54–1.64. Elytra narrowly subquadrate, MEW/HuW = 2.05–2.14.

**Male genitalia** (n = 2). Aedeagal median lobe gracile, distance from parameral articulation to tip 3.7× depth at midlength (Fig. 144H–I); apex parallel sided, of variable breadth, extended 1.5–2.7× minimum depth beyond ostial opening, tip with apical face less convex dorsad rounded juncture with ventral margin; internal sac with evident, broadly diffuse dorsal ostial microtrichial patch (uneverted specimen, Fig. 144H); flagellar plate of moderate size, length 0.35× parameral articulation-tip distance (uneverted specimens, Fig. 144H–I). Both figured specimens were collected at State Fence Camp on Helele‘ike‘oha Stream, and as there are no other features that diagnose these males, variation in the breadth of the median lobe and its apex is considered infraspecific.

**Female reproductive tract** (n = 1). Bursa copulatrix broad basally, with parallel-sided apical lobe with rounded apex, overall length 0.76 mm, apical lobe 0.60 mm long × 0.29 mm broad, base at vagina 0.38 mm broad (Fig. 145C); bursal walls translucent, thickly wrinkled; gonocoxite 1 with 3–4 apical fringe setae, the medial 1 or 2 smaller (Fig. 146C), a curved seta at apicomedial angle and 9–10 setae on medial surface; gonocoxite 2 narrowly subtriangular, with apex curved laterad, lateral margin straight near ensiform setae and base moderately extended laterally, 2 lateral ensiform setae, apical nematiform setae on medioventral surface at 0.76–0.79 gonocoxite length.

##### Holotype.

Female (CUIC) labeled: HI:Maui Haleakala N.P. / Kipahulu Vy. 1800m el. / 8-V-1991 sifting / leaf litter by day // S. Jessel / A.C. Medeiros / Jr. collectors // 2 // HOLOTYPE / Mecyclothorax / bicoloratus / Liebherr / det. J.K. Liebherr 2015 (black-margined red label).

##### Paratypes.

HI: Maui: Hana For. Res., Heleleikeoha Str. State Fence Camp, pyrethrin fog *Metrosideros*/moss, 1615 m el., 11-v-1998 lot 06, Polhemus (NMNH, 2), 12-v-1998 lot 03, Liebherr (CUIC, 1), lot 10 Polhemus (CUIC, 1; NMNH, 1).

##### Etymology.

The adjectival species epithet bicoloratus is used to name the second bicolored species in the *Mecyclothorax palustris* group, adding to the examples of sibling species named with the same word stem.

##### Distribution and habitat.

The distribution of *Mecyclothorax bicoloratus* lies to the east of that of *Mecyclothorax bicoloris*, with the known localities in upper Kīpahulu Valley (1800 m elevation) or the Helele‘ike‘oha Stream drainage at 1615 m elevation (Fig. 148). All specimens have been collected in association with ‘ōhi‘a, either in leaf litter or via pyrethrin fog application to mossy trunks and logs.

#### (110) Mecyclothorax bilobatus sp. n.

**Figure 149.**
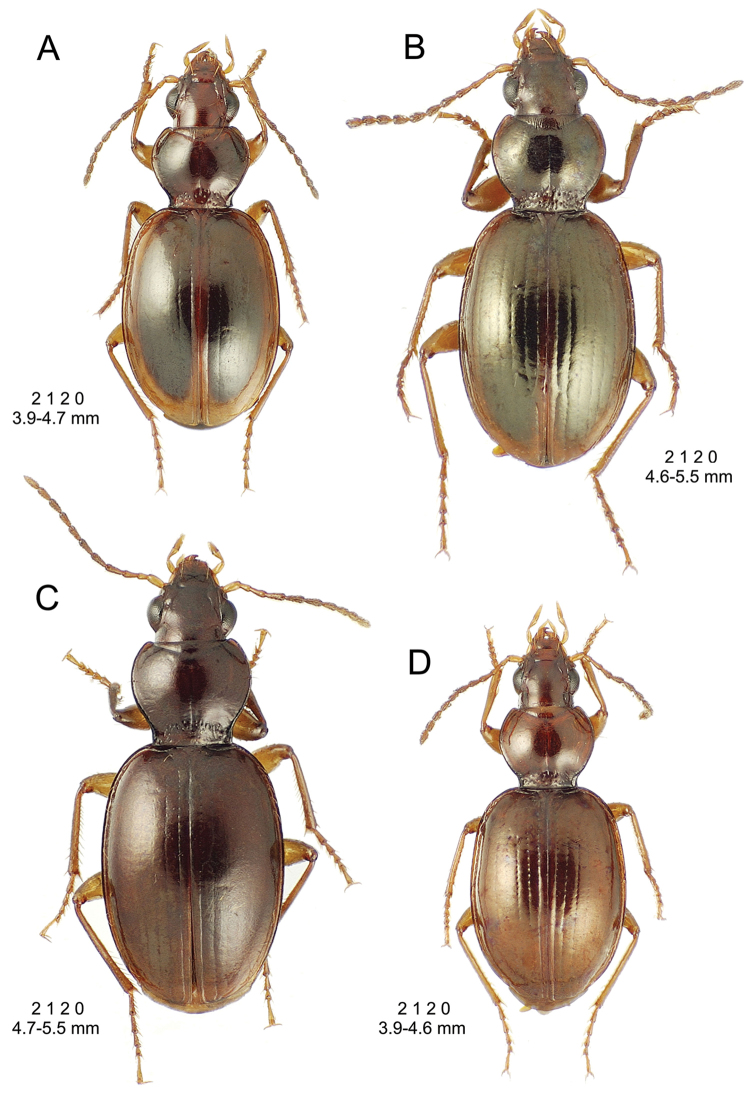
*Mecyclothorax palustris* group species, dorsal habitus view. **A** *Mecyclothorax bilobatus* (Helele‘ike‘oha, 1615 m) **B** *Mecyclothorax palustroides* (Honomanu, 1700 m) **C** *Mecyclothorax filipoides*. (Ukulele Camp Pipeline, 1525–1650 m) **D** *Mecyclothorax nanunctus* (Kīpahulu, 1800 m).

**Figure 150.**
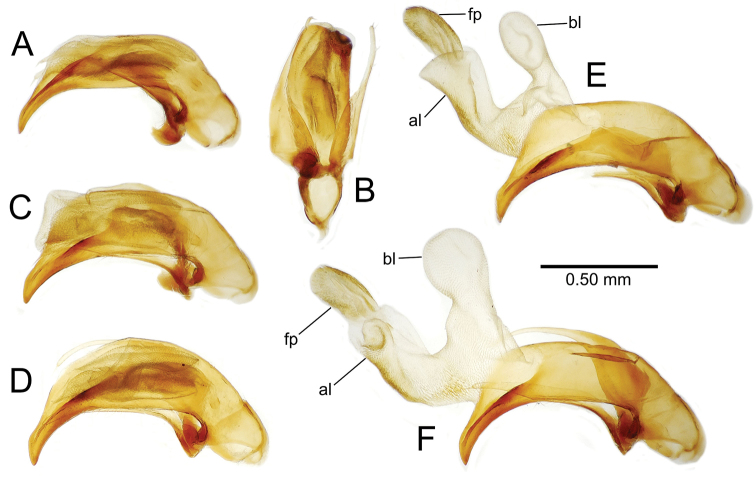
Male aedeagus, *Mecyclothorax bilobatus* (for abbreviations see Table 2, p. 23). **A–B** Right and ventral views (Kuhiwa, 1590 m) **C–D** Right views (Helele‘ike‘oha, 1615 m) **E–F** Right views, sac everted **E** (Helele‘ike‘oha, 1615 m) **F** (Helele‘ike‘oha, 1795 m).

**Figure 151.**
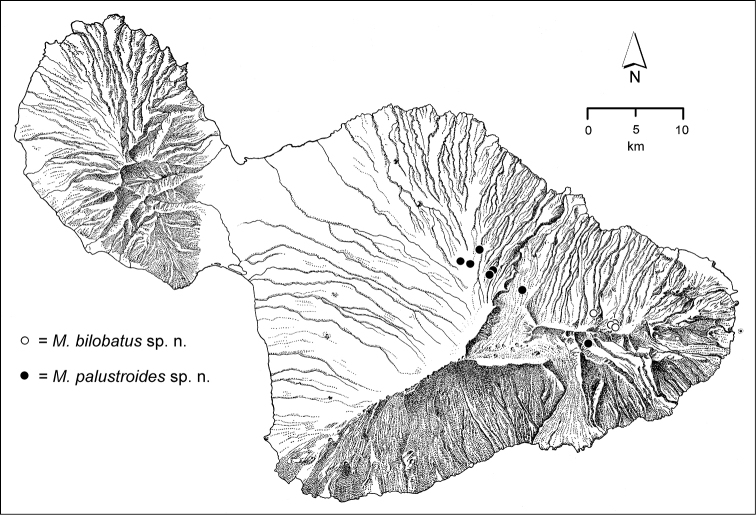
Recorded geographic distributions of *Mecyclothorax palustris* group species.

Taxon classificationAnimaliaColeopteraCarabidae

http://zoobank.org/ACE867F6-F299-40EE-A866-2300203EE3D0

(Figs 145D , 146D , 149A , 150 , 151

##### Diagnosis.

Among the four species in this group with individuals exhibiting contrastedly paler elytral margins, this species (Fig. 149A) is diagnosed by the very shallow elytral striae, with only the sutural and 2^nd^ striae impressed on the disc. The pronotal median base is less punctate than in the two bicolored species with punctate striae; *Mecyclothorax bicoloris* (Fig. 143C) and *Mecyclothorax bicoloratus* (Fig. 143D). In this species the pronotum has ~16 isolated punctures each side, and the surface of the pronotal laterobasal depression is smooth. The male aedeagal internal sac is bilobed in this species, with the basal lobe subequal in length to the apical lobe (Fig. 150E–F). Setal formula 2 1 2 0. Standardized body length 3.9–4.7 mm.

##### Description

(n = 5). *Head capsule* with frontal grooves broad and deep at clypeus, straight and subparallel, terminated at a low carina mesad anterior supraorbital seta; dorsal impression of neck slightly concave; ocular lobe obtusely protruded from gena, eyes convex, ocular ratio = 1.54–1.60, ocular lobe ratio = 0.78–0.81; labral anterior margin angularly emarginate 1/6 of labral length; antennae filiform, antennomeres 2–3 with sparse pelage of short setae; mentum tooth with sides acute, apex tightly rounded. *Pronotum* slightly transverse, MPW/PL = 1.15–1.20, basally constricted, MPW/BPW = 1.53–1.64, lateral margins briefly sinuate anterad acute, projected hind angles; median base depressed relative to disc; basal margin indistinctly trisinuate, slightly convex medially; median longitudinal impression very shallow, broad, crossed by indistinct transverse wrinkles; anterior transverse impression broad, very shallow medially, incised laterally mesad front angles; anterior callosity flat, slightly upraised, smooth; front angles not projected, rounded posterad curve of front margin; pronotal apical width variably subequal to broader than pronotal basal width, APW/BPW = 0.97–1.13; lateral marginal depression moderate, edge upturned at hind angle, beaded anterad inside basal sinuation, depression not wider at front angle. *Proepisternum* with 5 minute punctures along hind marginal groove; prosternal process with broad median depression, lateral margins broadly beaded between coxae. *Elytra* subovoid, disc convex, sides distinctly sloped to margins; basal groove evenly curved to angulate humerus, MEW/HuW = 2.14–2.36; parascutellar seta present; parascutellar striole with 4 isolated punctures; sutural interval coplanar with lateral intervals basally, upraised in apical half; sutural stria deeper than 2^nd^ stria throughout length, deeper and more punctate on disc, a series of isolated punctures near basal groove, and deep, narrow, and smooth at elytral apex; discal striae 2, or 2–3 traceable as linear series of shallow punctures, lateral striae traceable only as disturbances in the microsculpture, or melanic spots in the cuticle; striae 2, 3 and the apex of 7 shallow and traceable at elytral apex, area between striae 2 and 7 evenly convex there; 2 dorsal elytral setae at 0.26–0.28× and 0.49–0.57× elytral length, setal impressions small, shallow, spanning ½ width of interval 3; apical and subapical setae absent; lateral elytral setae arranged in anterior series of 7 setae and posterior series of 6 setae; elytral marginal depression narrow at humerus, margin upturned, beaded posteriorly near subapical sinuation; subapical sinuation shallow, more abruptly incurved anteriorly. *Mesepisternum* with 5 punctures in 1 row; metepisternal width to length ratio = 0.68; metepisternum/metepimeron suture distinct. *Abdomen* with irregular lateral wrinkles on ventrites 1–5, lateral depressions on ventrites 3–6; suture between ventrites 2 and 3 complete; apical male ventrite with 2 marginal setae and apical female ventrite with 4 equally spaced setae and median trapezoid of 4 subequal, short setae. *Legs*-metatarsomere 1/metatibial length ratio = 0.18; metatarsomere 4 length along outer lobe 1.3× medial tarsomere length, apical and subapical setae present; metatarsal dorsolateral sulci broad, shallow, median area broad. *Microsculpture* of vertex a shallow transverse mesh, sculpticell breadth 2–3× length; pronotal disc with obsolete transverse mesh, sculpticell breadth 2–4× length, median base glossy medially, indistinct isodiametric sculpticells laterally; elytral disc and apex with indistinct isodiametric sculpticells in transverse rows; metasternum with shallow transverse mesh; laterobasal abdominal ventrites with swirling isodiametric and transverse sculpticells. *Coloration* of vertex rufous; antennomeres 1–3 flavous, 4–11 rufoflavous; pronotal disc rufobrunneous, lateral margins, base, and anterior callosity rufoflavous; proepipleuron flavous, proepisternum rufobrunneous with rufoflavous margins; elytral disc rufopiceous, base rufobrunneous, sutural interval basally rufous, apical half rufoflavous, marginal intervals 7–9 contrastedly flavous, apex flavous apicad the posterior seta of the lateral setal series; elytral epipleuron flavous, metepisternum rufobrunneous; abdomen with ventrite 1 rufoflavous, ventrites 2–3 rufopiceous with flavous apex, ventrites 4–6 basally rufobrunneous, apical ¾ of apical ventrite 6 flavous; metafemur flavous; metatibia flavous with rufoflavous cast.

**Male genitalia** (n = 7). Aedeagal median lobe robust, distance from parameral articulation to tip 2.5–2.8× depth at midlength (Fig. 150A, C–F); apex a smoothly curved extension of ventral margin, the extension of variable depth (Fig. 150A versus C–F); median lobe broad, symmetrical in ventral view, the convex dorsal margin (Fig. 150A) visible as convex apical margin in this view (Fig. 150B); internal sac with well-developed basal lobe that is subequal in length to apical lobe (Fig. 150E–F), the lobe varying in breadth; sac with well-developed microspicules on ventral surface, a very diffuse patch of somewhat larger, lightly melanized macrospicules present near base (Fig. 150E); flagellar plate of moderate size, length 0.38–0.40× parameral articulation-tip distance.

**Female reproductive tract** (n = 1). Bursa copulatrix broad basally, with parallel-sided apical lobe with rounded apex, overall length 0.76 mm, apical lobe 0.56 mm long × 0.34 mm broad, base at vagina 0.55 mm broad (Fig. 145D); bursal walls translucent, thinly wrinkled; gonocoxite 1 with 3 apical fringe setae, 7–8 smaller setae on medial surface (146D); gonocoxite 2 falcate, with tightly rounded tip, base with long thick lateral extension that curves at terminus, 2 lateral ensiform setae, apical nematiform setae on medial surface at 0.76× gonocoxite length.

##### Holotype.

Male (CUIC) labeled: HI:Maui Haleakala / Hanawi N.A.R. Poouli / Cabin 6-V-1998 lot01 / 1590m el. pyrethrum fog / mossy ohia J.K. Liebherr // HOLOTYPE / Mecyclothorax / bilobatus / Liebherr / det. J.K. Liebherr 2015 (black-margined red label).

##### Paratypes.

103 specimens (see Appendix).

##### Etymology.

The adjectival species epithet bilobatus refers to the bilobed internal sac of the male aedeagus (Figs 150E–F).

##### Distribution and habitat.

*Mecyclothorax bilobatus* is known only from upper Kuhiwa and Helele‘ike‘oha Stream drainages, and Horseshoe Bog just mauka, i.e. uphill, from the Helele‘ike‘oha State Fence Camp (Fig. 151). These sites range 1590–1830 m elevation. All specimens of this species have been collected in association with mossy ‘ōhi‘a trunks and horizontal nurse logs.

#### (111) Mecyclothorax palustroides sp. n.

Taxon classificationAnimaliaColeopteraCarabidae

http://zoobank.org/E0DF2F5D-0617-4039-AE3B-4D08BA7015DD

**Figure 152.**
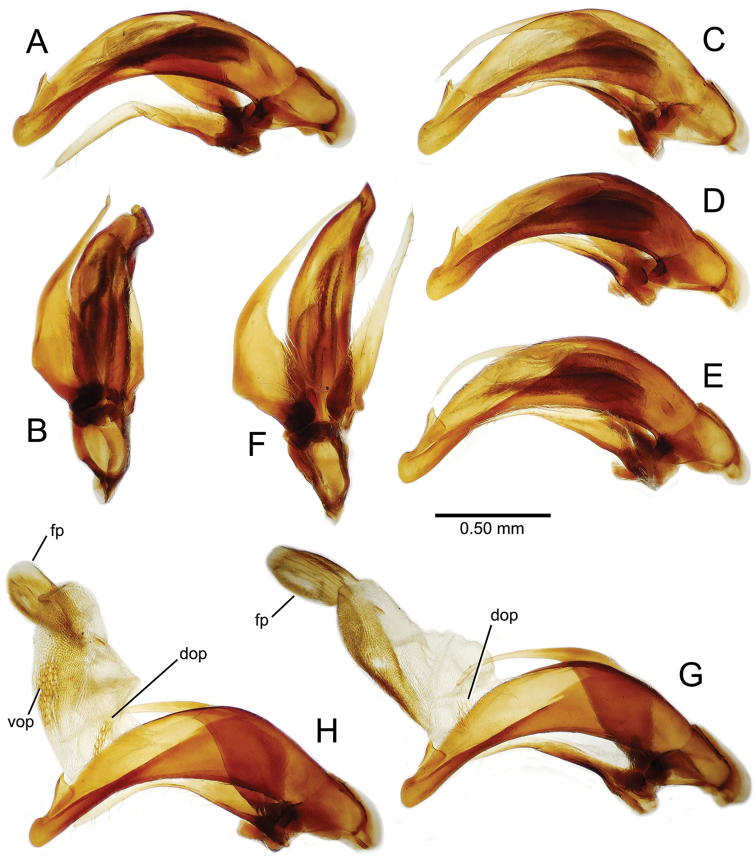
Male aedeagus, *Mecyclothorax palustroides* (for abbreviations see Table 2, p. 23). **A–B** Right and ventral views (Honomanu, 1830–1860 m) **C–D** Right views (Honomanu, 1700 m) **E–F** Right and ventral views (Kīpahulu, 1960 m) **G–H** Right views, sac everted **G** (Kīpahulu, 1960 m) **H** (Kīpahulu, 2055 m).

Figs 145E , 146E , 149B , 151 , 152

##### Diagnosis.

This species can be diagnosed by the impressed elytral striae 1–3 that are irregular along their length but not distinctly punctate, and the broadly flavous elytral apical, lateral, and sutural margins (Fig. 149B). The elytral coloration varies infraspecifically. The lateral intervals 7–9 are flavous in contrast to the rufopiceous discal intervals 2–6 in some individuals. Alternatively, the elytra may exhibit gradually paler lateral margins, with intervals 7 or 7–8 rufobrunneous mesad the more flavous 9^th^ interval and lateral marginal depression. The breadths of the flavous apical and lateral marginal bands are positively associated, with the apex variably pale from beyond the posterior seta of the lateral elytral setal series, or more narrowly pale beyond the apical fusion of striae 3 + 4 (Fig. 149B). Individuals with differing degrees of pale margination coöccur within the same collecting series. Individuals of this species are most similar to those of *Mecyclothorax tauberorum* and *Mecyclothorax pau*, though both of those species are characterized by darker, less contrasted lateral elytral margins. Individuals of *Mecyclothorax palustroides* are all larger than those of *Mecyclothorax tauberorum*; standardized body length for this species = 4.6–5.5 mm versus s.b.l. = 3.9–4.6 mm for *Mecyclothorax tauberorum*. *Mecyclothorax palustroides* can be diagnosed from *Mecyclothorax pau* by the more upraised sculpticells in the transverse discal elytral microsculpture, imparting an alutaceous sheen to the surface, and by the more broadly paler elytral intervals 8–9 posterad the humerus; that pale margin either rufobrunneous or flavous versus the rufopiceous disc. Also, the male aedeagal median lobe can definitively diagnose the 3 species: 1, *Mecyclothorax palustroides* with the lobe apex broadly rounded (Fig. 152); 2, *Mecyclothorax tauberorum* with the lobe apex short and bluntly rounded (Fig. 161A, C–D); and 3, *Mecyclothorax pau* with the lobe apex elongate, narrowly rounded (Fig. 161E–F, H–L). Setal formula 2 1 2 0.

##### Description

(n = 5). *Head capsule* with frontal grooves broad near clypeus, triangularly expanded medially, and divergent to terminate mesad fine carina inside anterior supraorbital seta; dorsal impression of neck slightly concave; ocular lobe obtusely protruded from gena, ocular ratio = 1.50–1.58, ocular lobe ratio = 0.81–0.84; labral anterior margin angularly emarginate to 1/9 labral length; antennae filiform, antennomeres 2–3 with sparse pelage of short setae; mentum tooth with sides acute, apex tightly rounded. *Pronotum* slightly transverse, MPW/PL = 1.08–1.21, variably constricted basally, MPW/BPW = 1.52–1.68; lateral margins convergent for short distance anterad right hind angles, the basal margin convex just mesad hind angle; median base distinctly depressed relative to disc, elongate punctures bordering disc, ~19 isolated punctures each side; basal margin trisinuate, slightly convex medially; median longitudinal impression very shallow, middle of disc flat; anterior transverse impression obsolete medially, finely incised laterally, fine longitudinal wrinkles extended from impression across flat anterior callosity; front angles slightly projected, tightly rounded; pronotal apical width variably broader than pronotal basal width, APW/BPW = 1.02–1.18; lateral marginal depression narrow, edge upturned laterally, slightly broader at front angle; laterobasal depression narrow, surface irregular, continuous with lateral depression. *Proepisternum* with 5 minute punctures along hind marginal groove; prosternal process with narrow median impression, lateral margins broadly upraised. *Elytra* subovoid, lateral margins rounded posterad humeral angles, disc convex, sides distinctly sloped to marginal depression; basal groove evenly curved to subangulate humerus, MEW/HuW = 2.21–2.33; parascutellar seta present; parascutellar striole with 4–5 punctures, striole very shallow between punctures; sutural interval coplanar with lateral intervals basally, upraised in apical half; sutural and 2^nd^ striae of similar depth on disc, sutural interval continued as isolated punctures at base, sutural stria deep, smooth and finely incised apically, 2^nd^ stria broader and irregularly interrupted apically; discal striae 2–5 progressively shallower, inner striae irregular, lateral striae represented by isolated punctures, striae 6–7 traceable only as series of very shallow punctures at midlength; mesal intervals slightly convex, lateral intervals flat; apex with striae 1, 2, 7, and 8 present, striae 3–6 obsolete, though intermittently traceable; 8^th^ interval slightly convex laterad 7^th^ stria mesad subapical sinuation; 2 dorsal elytral setae at 0.27× and 0.54–0.64× elytral length, setal impressions small, shallow, spanning ½ width of interval 3; lateral elytral setae arranged in anterior series of 7 setae and posterior series of 6 setae; elytral marginal depression moderately broad with edge upturned laterad humerus, narrowed to a beaded margin at subapical sinuation; subapical sinuation shallow, more abruptly incurved anteriorly. *Mesepisternum* with ~9-10 punctures in 2–3 rows; metepisternal width to length ratio = 0.71; metepisternum/metepimeron suture distinct. *Abdomen* with irregular lateral wrinkles on ventrites 1–5, lateral depressions on ventrites 3–6; suture between ventrites 2 and 3 complete; apical male ventrite with 2 marginal setae and apical female ventrite with 4 equally spaced setae and median trapezoid of 4 subequal, short setae. *Legs*-metatarsomere 1/metatibial length ratio = 0.20; metatarsomere 4 length along outer lobe 1.3× medial tarsomere length, apical and subapical setae present; metatarsal dorsolateral sulci broad, shallow, basal tarsomere medially subcarinate. *Microsculpture* of vertex a distinct transverse mesh, sculpticell breadth 2–3× length; pronotal disc with shallow transverse mesh, sculpticell breadth 3–4× length, median base with indistinct transverse mesh, sculpticell breadth 2× length; elytral apex with shallow isodiametric and transverse sculpticells, sculpticell breadth 2× length; metasternum with shallow transverse mesh; laterobasal abdominal ventrites with swirling isodiametric and transverse microsculpture. *Coloration* of vertex rufobrunneous with piceous cast; antennomeres 1–3 rufoflavous, 4–11 rufobrunneous; pronotal disc rufobrunneous with piceous cast, lateral margins narrowly, and base and apex rufoflavous; proepipleuron rufoflavous, proepisternum dorsally rufoflavous, ventrally rufobrunneous; elytral disc dark rufobrunneous with iridescent sheen, sutural interval rufous in basal half, rufoflavous in apical half; elytral epipleuron rufoflavous, metepisternum rufobrunneous; abdomen with ventrites 1–3 rufopiceous medially, rufoflavous laterally, ventrites 4–6 basally rufobrunneous, apically flavous, the apical ventrite flavous in apical 3/4; metafemur flavous; metatibia flavous with rufoflavous cast.

**Male genitalia** (n = 19). Aedeagal median lobe gracile to slightly robust, dorsal and ventral margins subparallel along median shaft, distance from parameral articulation to tip 3.2–4.1× depth at midlength (Fig. 152A, C–H); apex broadly and briefly extended, tip slightly curved apically along ventral margin, slightly expanded along dorsal margin, with obliquely convex apical face; median lobe broadly, evenly curved rightward apically in ventral view (Fig. 152B, F), the concave right margin and convex left margin convergent to blunt, oblique tip; internal sac with very pale, diffuse dorsal ostial microtrichial patch near base, and variably ornamented ventral surface, covered either with shaggy pelage of microspicules (Fig. 152G), or a ventral ostial microtrichial patch composed of overlapping scaly macrospicules (Fig. 152H); flagellar plate moderately sized, length 0.40× parameral articulation-tip distance.

**Female reproductive tract** (n = 1). Bursa copulatrix columnar with basally constricted apical lobe, length 1.08 mm, apical cap 0.40 mm long × 0.29 mm broad, width at midlength 0.40 mm (Fig. 145E); bursal walls translucent, thickly wrinkled; gonocoxite 1 with 4 apical fringe setae, a curved seta near medioapical angle and 3–5 smaller setae on medial surface (Fig. 146E); gonocoxite 2 falcate, apex broad with sensilla doubled along lateral margin, base broadly extended laterally, 2 lateral ensiform setae, apical nematiform setae on medioventral surface at 0.77× gonocoxite length.

##### Holotype.

Male (CUIC) labeled: HI: Maui Haleakala NW / slope Waikamoi Pres. / trans. 3 @ 1700 m el. / 10-IV-1991 sifting / litter J.K. Liebherr // HOLOTYPE / Mecyclothorax / palustroides / Liebherr / det. J.K. Liebherr 2015 (black-margined red label).

##### Paratypes.

61 specimens (see Appendix).

##### Etymology.

This species’ resemblance to *Mecyclothorax palustris* (Sharp) leads to the use of palustroides as the species epithet. The stem palustris describes marshy or swampy situations.

##### Distribution and habitat.

*Mecyclothorax palustroides* is known from the Waikamoi area, Ke‘anae Valley, and upper Kīpahulu Valley at 1265–2045 m elevation (Fig. 151). Its distribution is congruently disjunct across the Hanawī face of Haleakalā with several other widespread species in the *Mecyclothorax palustris* group; i.e. *Mecyclothorax nanunctus* (Fig. 157), *Mecyclothorax unctus* (Fig. 160), and *Mecyclothorax pau* (Fig. 163). The Kopili‘ula drainage at the center of this gap has experienced significant dieback of the ‘Ōhi‘a Forest (Holt 1983), with the area now characterized by open koa “savannah” standing in dense tangles of *Dicranopteris* (uluhe) fern. However this particular area of ‘ōhi‘a loss represents only a portion of the range disjunctions for the various species, likely requiring a more complicated solution to the explanation of this biogeographic pattern.

#### (112) Mecyclothorax filipoides sp. n.

Taxon classificationAnimaliaColeopteraCarabidae

http://zoobank.org/6ED46044-7B50-47E7-9463-06D400E1D6C1

**Figure 153.**
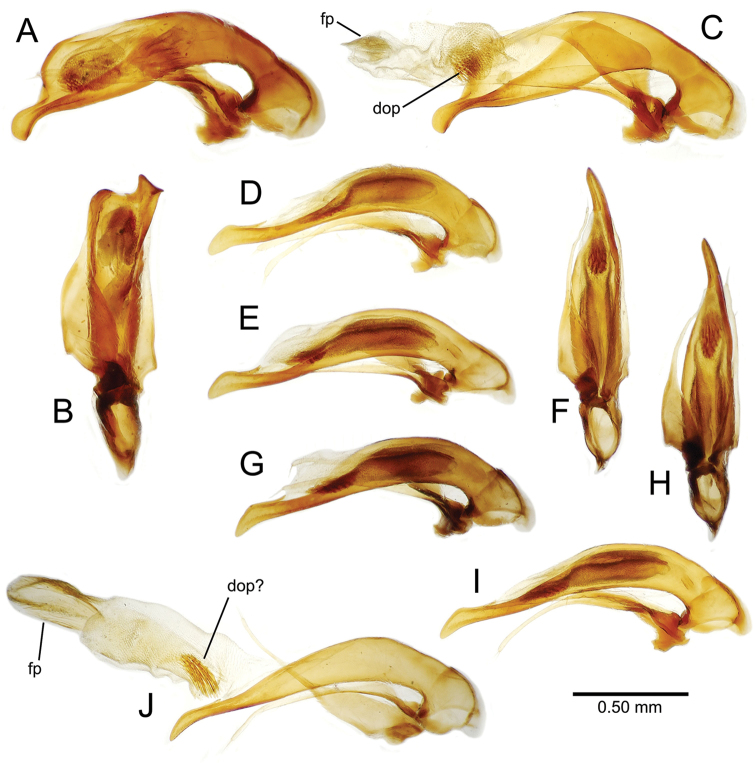
Male aedeagus, *Mecyclothorax palustris* group species (for abbreviations see Table 2, p. 23). **A–C** *Mecyclothorax filipoides* **A–B** Right and ventral views (Honomanu 1800–1860 m) **C** Right view, sac everted (Honomanu, 1750 m) **D–J** *Mecyclothorax nanunctus* **D** Right view (Kuhiwa, 1615 m) **E–F** Right and ventral views (Ukulele Camp Pipeline, 1510 m) **G–H** Right and ventral views (Kuhiwa, 1590 m) **I** Right view (Kīpahulu, 1800 m) **J** Right view, sac everted (Kuhiwa, 1590 m).

**Figure 154.**
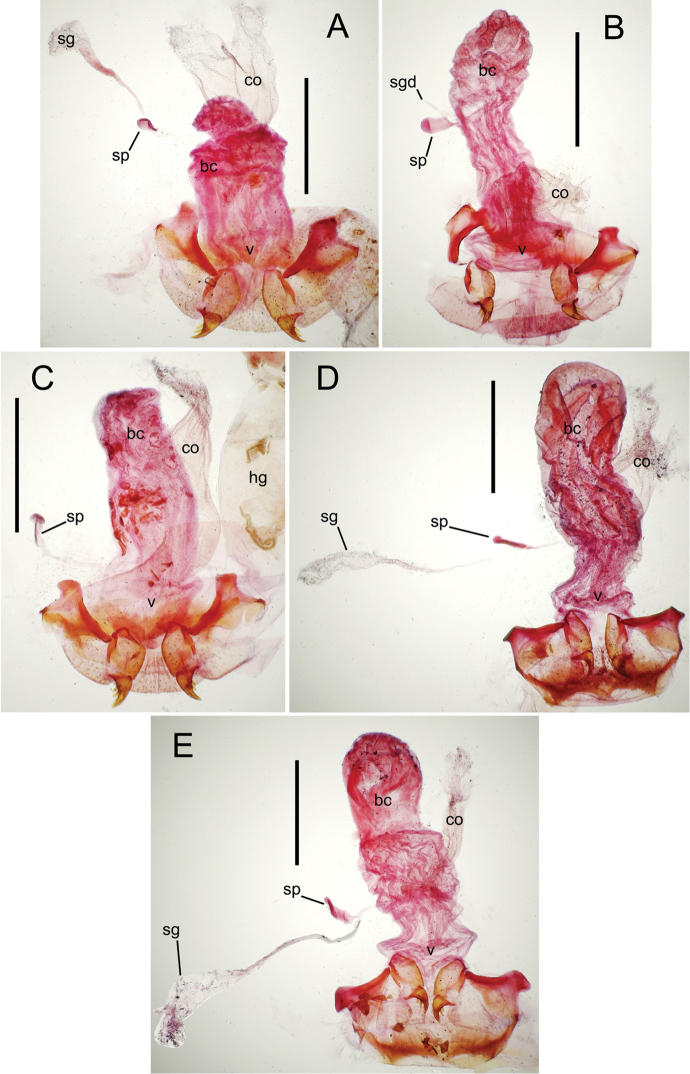
Female bursa copulatrix and associated reproductive structures, *Mecyclothorax palustris* group species, ventral view (for abbreviations see Table 2, p. 23). **A** *Mecyclothorax filipoides* (Ukulele Pipeline, 1525–1650 m) **B** *Mecyclothorax nanunctus* (Kuhiwa, 1590 m) **C** *Mecyclothorax unctus* (Paliku, 1950 m) **D** *Mecyclothorax tauberorum* (Waikamoi, 1275 m) **E** *Mecyclothorax pau* (Kīpahulu, 1200 m). Scale bar = 0.50 mm.

**Figure 155.**
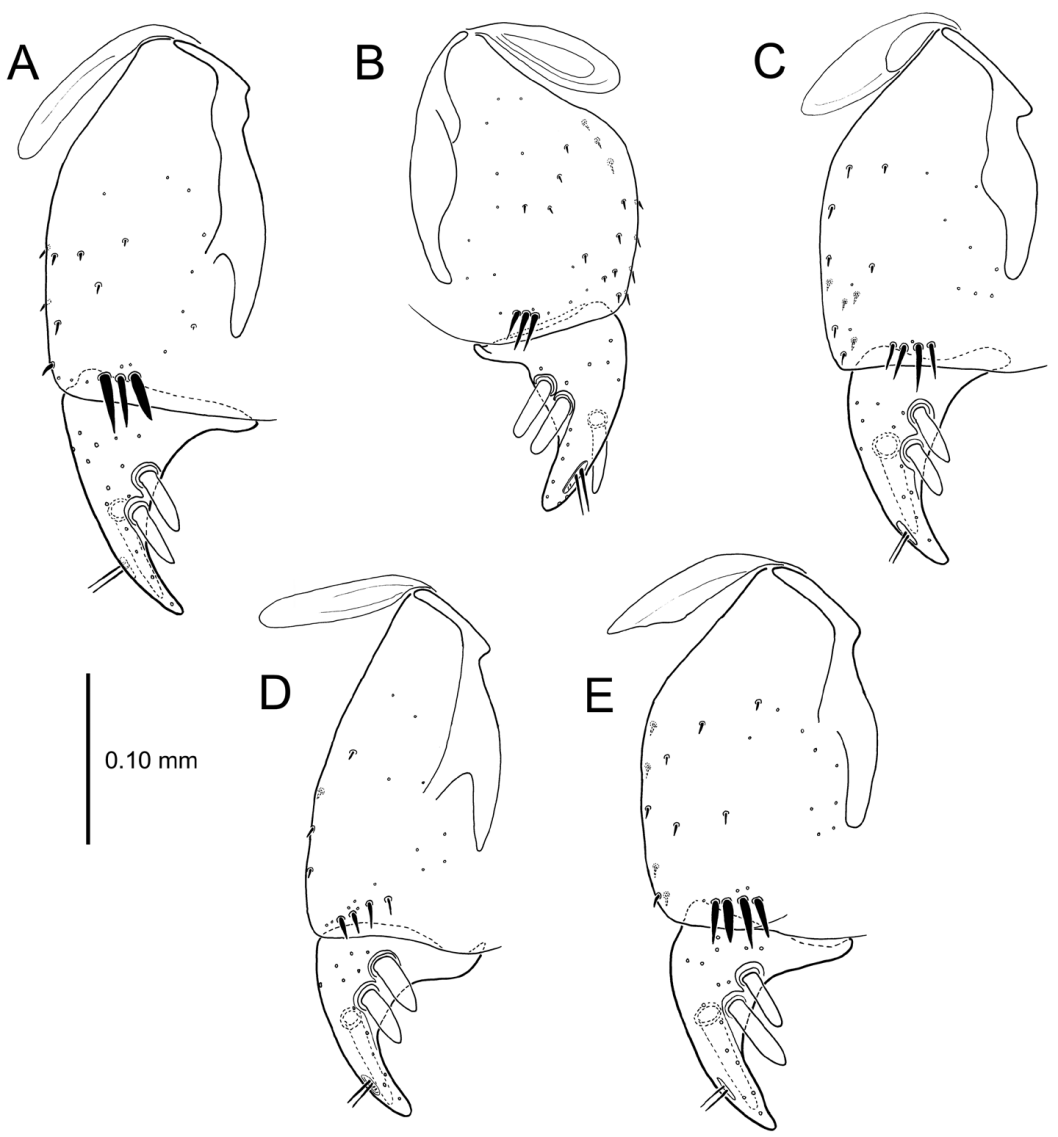
Female gonocoxa, *Mecyclothorax palustris* group species, ventral view. **A** *Mecyclothorax filipoides*, left gonocoxa (Ukulele Pipeline, 1525–1650 m) **B** *Mecyclothorax nanunctus*, right gonocoxa (Kuhiwa, 1590 m) **C** *Mecyclothorax unctus*, left gonocoxa (Paliku, 1950 m) **D** *Mecyclothorax tauberorum*, left gonocoxa (Waikamoi, 1275 m) **E** *Mecyclothorax pau*, left gonocoxa (Kīpahulu, 1200 m).

**Figure 156.**
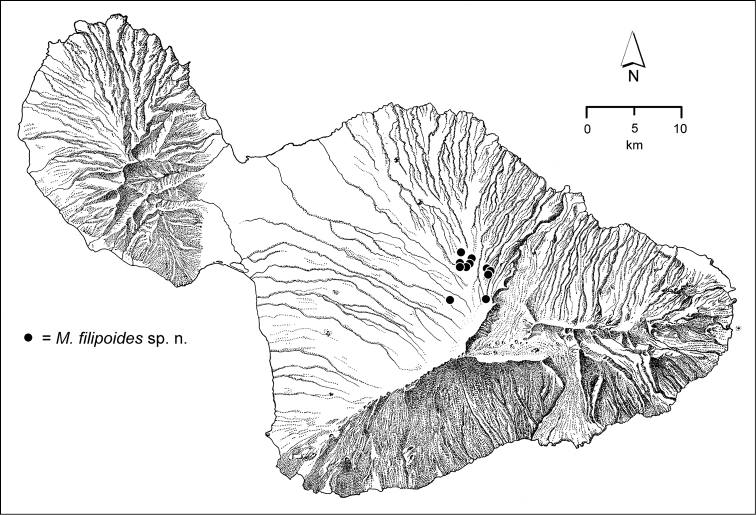
Recorded geographic distribution of *Mecyclothorax filipoides*.

Figs 149C , 153A–C , 154A , 155A , 156

##### Diagnosis.

Among Haleakalā’s *Mecyclothorax palustris* group species, *Mecyclothorax filipoides* stands out based on the disparate dorsal microsculpture: 1, head and pronotum with glossy surfaces, their microsculpture respectively a shallow transverse mesh, and obsolete transverse mesh to transverse lines; 2, elytral disc and apex with coriaceous surface comprised of isodiametric or slightly transversely stretched sculpticells arranged in irregular transverse rows. The elytral disc and lateral intervals are rufobrunneous with the elytral apex only indistinctly paler. The discal elytral striae are shallow, with the punctures of the inner striae isolated, and the sutural stria the most complete (Fig. 149C). Setal formula 2 1 2 0. Body size is large among species of the group; standardized body length 4.7–5.5 mm.

##### Description

(n = 5). *Head capsule* with frontal grooves narrow and deep anteriorly, sinuous laterally and shallower until terminated posteriorly mesad thin carina that borders depression surrounding anterior supraorbital seta; dorsal impression of neck slightly concave; ocular lobes obtusely protruded from gena, eyes large, ocular ratio = 1.52–1.59, ocular lobe ratio = 0.82–0.88; labral anterior margin angularly emarginate 1/7 labral length; antennae filiform, antennomeres 2–3 with sparse pelage of short setae; mentum tooth with sides acute, apex tightly rounded. *Pronotum* appearing quadrate, MPW/PL = 1.12–1.19, lateral margins slightly convergent anterad acute hind angles for 1/6 of pronotal length, base moderately constricted, MPW/BPW = 1.47–1.56; median base slightly depressed medially, more so laterally, ~25 isolated punctures each side, those along juncture of base and disc elongate; basal margin trisinuate, slightly convex medially and expanded posteriorly behind laterobasal depressions; median longitudinal impression shallow, finely incised, smooth, continued onto median base; anterior transverse impression broad, very shallow medially, well-marked laterally mesad front angles; anterior callosity flat, gradually elevated from transverse impression to front margin, crossed by minute longitudinal wrinkles; front angles projected, narrowly rounded; pronotal apical width slightly less than pronotal basal width, APW/BPW = 0.95–0.99; lateral marginal depression moderate, edge beaded laterally to basal sinuation, more broadly upraised outside laterobasal depressions; laterobasal depression minutely punctate, continuous with lateral depression. *Proepisternum* with 4 distinct punctures along hind marginal groove; prosternal process with broad median depression, lateral margins broadly margined between coxae. *Elytra* subquadrate, disc convex, sides distinctly sloped to margins; basal groove evenly curved to subangulate to angulate humerus defined by hitch in margin at juncture of narrow basal groove and broader lateral marginal depression, MEW/HuW = 2.09–2.24; parascutellar seta present; parascutellar striole with 5 large punctures, striole shallow to interrupted between punctures; sutural interval broadly domed to elevated sutural juncture; sutural stria punctate, deeper than 2^nd^ stria on disc, finely incised, deep, and smooth apically, where 2^nd^ stria is broader and shallower, and irregularly interrupted; discal striae 2–4 progressively shallower laterally with elongate punctures progressively isolated, associated intervals flat, appearing concave on middle of disc; striae 1, 2, and 7 present on elytral apex, striae indicated by series of isolated punctures, 8^th^ interval slightly more convex than 7^th^ mesad subapical sinuation; 2 dorsal elytral setae at 0.15–0.18× and 0.53–0.60× elytral length, setal impressions small, shallow, spanning ½ width of interval 3; lateral elytral setae arranged in anterior series of 7 setae and posterior series of 6 setae; elytral marginal depression moderately broad, margin little upraised even near subapical sinuation; subapical sinuation shallow, concavity symmetrical. *Mesepisternum* with ~18 punctures in 2–3 rows; metepisternal width to length ratio = 0.81; metepisternum/metepimeron suture distinct. *Abdomen* with irregular lateral wrinkles on ventrites 1–5, and lateral depressions on ventrites 3–6; suture between ventrites 2 and 3 reduced laterally, effaced; apical male ventrite with 2 marginal setae and apical female ventrite with 4 equally spaced setae and median trapezoid of 4 subequal, short setae. *Legs*-metatarsomere 1/metatibial length ratio = 0.20; metatarsomere 4 length along outer lobe 1.3× medial tarsomere length, apical and subapical setae present but short; metatarsal dorsolateral sulci broad, shallow, median area subcarinate. *Microsculpture* of vertex a shallow transverse mesh, sculpticell breadth 2–3× length; pronotal median base glossy, shallow transverse sculpticells laterally near punctures; metasternum with shallow transverse mesh; laterobasal abdominal ventrites with swirling isodiametric and transverse sculpticells. *Coloration* of vertex rufobrunneous with piceous cast; antennomeres 1–3 rufoflavous, 4–11 rufobrunneous; pronotal disc rufobrunneous, lateral margins narrowly, and base and apex rufoflavous; proepipleuron rufoflavous, proepisternum rufobrunneous with piceous cast; elytral disc rufobrunneous, sutural interval basally rufous, concolorous on disc, apically rufoflavous; elytral marginal depression rufoflavous outside humerus, concolorous with disc posteriorly; elytral epipleuron rufoflavous, metepisternum rufobrunneous; abdominal ventrites 1–5 rufopiceous medially, rufoflavous laterally, apical ¾ of apical ventrite 6 rufoflavous; metafemur flavous; metatibia rufoflavous.

**Male genitalia** (n = 3). Aedeagal median lobe very slender immediately distad basal bulb, greatly expanded along dorsal margin of ostial opening, distance from parameral articulation to tip 3.2× greatest depth (Fig. 153A, C), apex extended as an asymmetrical spoon with blunt ventral projection; median lobe with apex directed rightward in ventral view (Fig. 153B), bulging dorsal margin (Fig. 153A) visible as convex apical prominence in this view; internal sac short, narrow, with broad, diffuse dorsal ostial microtrichial patch near base (Fig. 153C); flagellar plate small, length 0.20× parameral articulation-tip distance (Fig. 153C).

**Female reproductive tract** (n = 1). Bursa copulatrix broadly columnar with small apical cap, overall length 0.78 mm, cap 0.29 mm broad × 0.17 mm long, shaft breadth 0.43 mm (Fig. 154A); apical cap finely wrinkled, shaft translucent with thick wrinkles; gonocoxite 1 with 3–4 apical fringe setae, a curved seta just basad medioapical angle and 7 smaller setae on medial surface (Fig. 155A); gonocoxite 2 falcate with acuminate tip, basal extension straight, 2 lateral ensiform setae, apical nematiform setae on medial surface at 0.68× gonocoxite length.

##### Holotype.

Male (BPBM) labeled: N.W. / Haleakala / VIII-18-37 Maui // 6000’–6500’ // Beating / ECZimmerman / Collector // HOLOTYPE / Mecyclothorax / filipoides / Liebherr / det. J.K. Liebherr 2015 (black-margined red label).

##### Paratypes.

54 specimens (see Appendix).

##### Etymology.

This species appears not unlike *Mecyclothorax filipes* (Sharp) of Lāna‘i (Liebherr 2009b), leading to filipoides as the species epithet. The adjectival stem filipes refers to the possession of threadlike feet (Jaeger 1955).

##### Distribution and habitat.

*Mecyclothorax filipoides* is distributed over a broad range of elevations—1310–1980 m—across the Waikamoi area (Fig. 156). Specimen collections are equally frequent from koa and ‘ōhi‘a, with beetles also associated with rotten *Cheirodendron* (‘ōlapa) trunks. Other collecting events involved beating vegetation at night, sifting litter, or capture in yellow-pan traps.

#### (113) Mecyclothorax nanunctus sp. n.

Taxon classificationAnimaliaColeopteraCarabidae

http://zoobank.org/418DC118-8C57-4092-BE75-D26F8BD0CF1F

**Figure 157.**
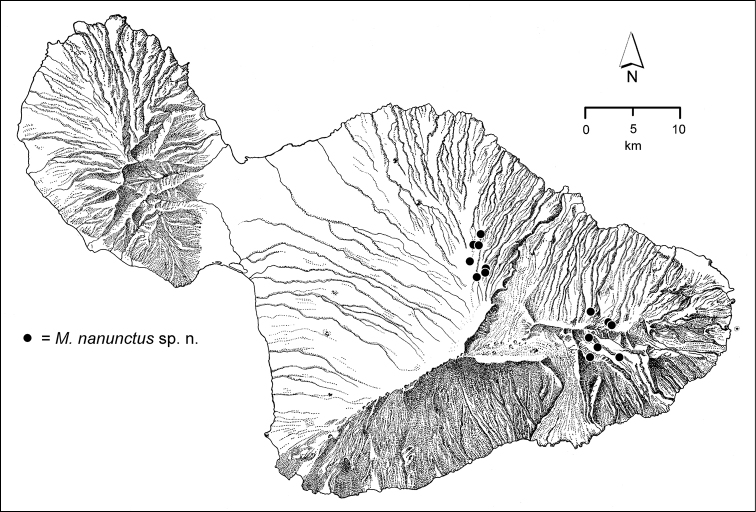
Recorded geographic distribution of *Mecyclothorax nanunctus*.

Figs 149D , 153D–J , 154B , 155B , 157

##### Diagnosis.

This species (Fig. 149D) can be separated from all other *Mecyclothorax palustris* group species based on: 1, uniformly pale body coloration without contrasted elytral lateral margins; 2, presence of two dorsal elytral setae, setal formula 2 1 2 0; and 3, relatively small size; standardized body length 3.9–4.6 mm. Were it to be confused with any other Haleakalā *Mecyclothorax*, the best candidate for confusion would be *Mecyclothorax mauiae* (Fig. 68A), as beetles of both species are pale bodied, and the size ranges overlap. Careful attention must be paid to the comparative depth of the sutural and 2^nd^ striae, as this is a major criterion for group membership. Also, the bodies of *Mecyclothorax mauiae* are broader, with the elytra slightly obovoid; i.e. with the greatest breadth in the anterior half. Conversely *Mecyclothorax nanunctus* beetles have the elytra more ovoid, with the greatest width in the apical half. The pronotum in *Mecyclothorax mauiae* is less transverse, though not diagnostically so, and the pronotal lateral depression is narrower with a less explanate margin. The surest arbiter given a male in the series is the male aedeagal median lobe, which in *Mecyclothorax nanunctus* males is gracile and elongate (Fig. 153D–J), and in *Mecyclothorax mauiae* males is short with a broadly rounded apex (Fig. 69). The two species are broadly sympatric (Figs 71, 157), but based on ecological records from the Waikamoi area, *Mecyclothorax nanunctus* is a species more at home in mesic forest situations dominated by *Acacia koa* and ‘ōhi‘a (*Metrosideros polymorpha*), whereas *Mecyclothorax mauiae* is found in wet forest situations that are dominated by ‘ōhi‘a but which also include ‘ōlapa (*Cheirodendron trigynum*).

##### Description

(n = 5). *Head capsule* with frontal grooves broad for most of length, terminated mesad low convexity mesad anterior supraorbital seta; dorsal impression of neck slightly concave; ocular lobe obtusely protruded from gena, eyes small and moderately convex, ocular ratio = 1.41–1.49, ocular lobe ratio = 0.79–0.84; labral anterior margin shallowly emarginate to 1/8 labral length; antennae filiform; antennomeres 2–3 with sparse pelage of short setae; mentum tooth with sides acute, apex tightly rounded. *Pronotum* moderately transverse, MPW/PL = 1.18–1.25, base narrowly constricted, MPW/BPW = 1.57–1.68, lateral margins subparallel for 0.1× pronotal length anterad right hind angles; median base distinctly depressed relative to convex disc, ~19 isolated punctures each side, half of them lining juncture between base and disc; basal margin slightly convex between laterobasal depressions; median longitudinal impression very shallow, middle of disc flat; anterior transverse impression broad, shallow, obsolete medially, crossed by indistinct wrinkles, incised only mesad front angles; anterior callosity slightly convex, crossed by indistinct longitudinal wrinkles; front angles slightly projected, tightly rounded; pronotal apical width greater than basal width, APW/BPW = 1.05–1.09; lateral marginal depression very narrow, slightly wider at front angle, thickly upraised outside laterobasal depression; laterobasal depression narrow, surface irregular, continuous with lateral depression. *Proepisternum* with 5 minute punctures along hind marginal groove; prosternal process with narrow lateral depression, lateral margins broadly upraised. *Elytra* convex, disc domed, sides distinctly sloped; basal groove evenly curved to tightly rounded humerus defined by juncture of basal groove and broader lateral marginal depression, MEW/HuW = 2.21–2.34; parascutellar seta present; parascutellar striole with 4 punctures, striole shallow or interrupted between punctures; sutural interval more convex than lateral intervals, sutural juncture upraised; sutural and 2^nd^ striae of similar depth and punctation on disc, sutural stria continuous to base, and finely incised, deep, and smooth apically, whereas 2^nd^ stria is absent from base, shallow and irregularly interrupted apically; discal striae 2–3 shallow, punctate, striae 4–5 very shallow to obsolete, striae 6–7 only shallow isolated punctures, or absent; elytral apex between 2^nd^ and 8^th^ striae uniformly convex; 2 dorsal elytral setae at 0.27–0.29× and 0.49–0.51× elytral length, setal impressions small, shallow, spanning ½ width of interval 3; lateral elytral setae arranged in anterior series of 6 setae and a posterior series of 5(6) setae; elytral marginal depression broader at humerus, edge little upturned there, gradually narrowed to bead at subapical sinuation; subapical sinuation shallow, more abruptly incurved anteriorly. *Mesepisternum* with ~10 punctures in 2–3 rows; metepisternal width to length ratio = 0.68; metepisternum/metepimeron suture distinct. *Abdomen* with irregular lateral wrinkles on ventrites 1–5, and lateral depressions on ventrites 3–6; suture between ventrites 2 and 3 complete; apical male ventrite with 2 marginal setae and apical female ventrite with 4 equally spaced setae and median trapezoid of 4 subequal, short setae. *Legs*-metatarsomere 1/metatibial length ratio = 0.19; metatarsomere 4 length along outer lobe 1.2× medial tarsomere length, apical and subapical setae present; metatarsal dorsolateral sulci broad, shallow, median area subcarinate. *Microsculpture* of vertex a transverse mesh, sculpticell breadth 2–3× length; pronotal disc with shallow transverse mesh to transverse lines, glossy in part, median base with shallow transverse mesh between glossy portions; elytral disc with distinct, regular transverse mesh, sculpticell breadth 2–3× length, apex with less-developed mesh of same dimensions; metasternum with shallow transverse mesh; laterobasal abdominal ventrites with swirling isodiametric and transverse microsculpture. *Coloration* of vertex rufous; antennomeres 1–3 flavous, 4–11 rufoflavous; pronotal disc rufoflavous, base slightly darker, rufobrunneous; proepipleuron flavous, proepisternum dorsally flavous, ventrally rufoflavous; elytral disc rufobrunneous, sutural interval rufoflavous basally, flavous apically, lateral depression and apex of 8^th^ interval flavous; elytral epipleuron flavous, metepisternum rufoflavous; abdomen with all ventrites medially rufoflavous, marginally flavous, apical half of apical ventrite flavous; metafemur flavous; metatibia flavous with rufoflavous cast.

**Male genitalia** (n = 9). Aedeagal median lobe slender, elongate, distance from parameral articulation to tip 5.6–6.0× maximum depth of shaft (Fig. 153D–E, G, I–J), apex narrowly extended 3.0–3.5× depth beyond ostial opening (Fig. 153E, J); median lobe nearly symmetrical in ventral view (Fig. 153F, H), with apex curved leftward to pointed tip in this view; internal sac with well-developed microtrichial field on right-ventral face near base (homologous with dorsal patch?), sac surface otherwise covered with fine microspicules (Fig. 153J); flagellar plate of moderate size, length 0.40× parameral articulation-tip distance.

**Female reproductive tract** (n = 1). Bursa copulatrix parallel sided, elongate, with slight apical expansion, length 1.06 mm, breadth 0.29 mm (Fig. 154B); bursal walls translucent, thinly wrinkled; gonocoxite 1 with 3–4 apical fringe setae, a small seta near medioapical angle and another ~16 small setae on medial surface (Fig. 155B); gonocoxite 2 falcate with subacuminate apex, lateral margin straight near ensiform setae, terminus of basal extension slightly curved, 2 elongate lateral ensiform setae, apical nematiform setae on medioventral surface at 0.69× gonocoxite length.

##### Holotype.

Male (BPBM) labeled: Kipahulu Valley / Maui Camp 2 / 1250 m, 13-17.VIII.67 // N. Wilson / Collector / BISHOP // HOLOTYPE / Mecyclothorax / nanunctus / Liebherr / det. J.K. Liebherr 2015 (black-margined red label).

##### Paratypes.

84 specimens (see Appendix).

##### Etymology.

During the initial sorting of specimens for this revision, this species was given the working name “little-unctus.” That name is validated here using the prefixed Latin participle nanunctus, or “small-anointed” (Brown 1956).

##### Distribution and habitat.

*Mecyclothorax nanunctus* exhibits a bipartite Waikamoi plus eastern Hanawī-Kīpahulu Valley distribution (Fig. 157), with collecting localities ranging 975–1850 m elevation. Nearly all specimens are associated with ‘ōhi‘a, though one specimen was obtaining scraping koa bark. Specimens of this species have also been found in association with *Astelia* (painiu), and *Cibotium* (hāpu‘u), in sifted litter, and by beating ferns and other vegetation at night.

#### (114) Mecyclothorax unctus

Taxon classificationAnimaliaColeopteraCarabidae

(Blackburn)

**Figure 158.**
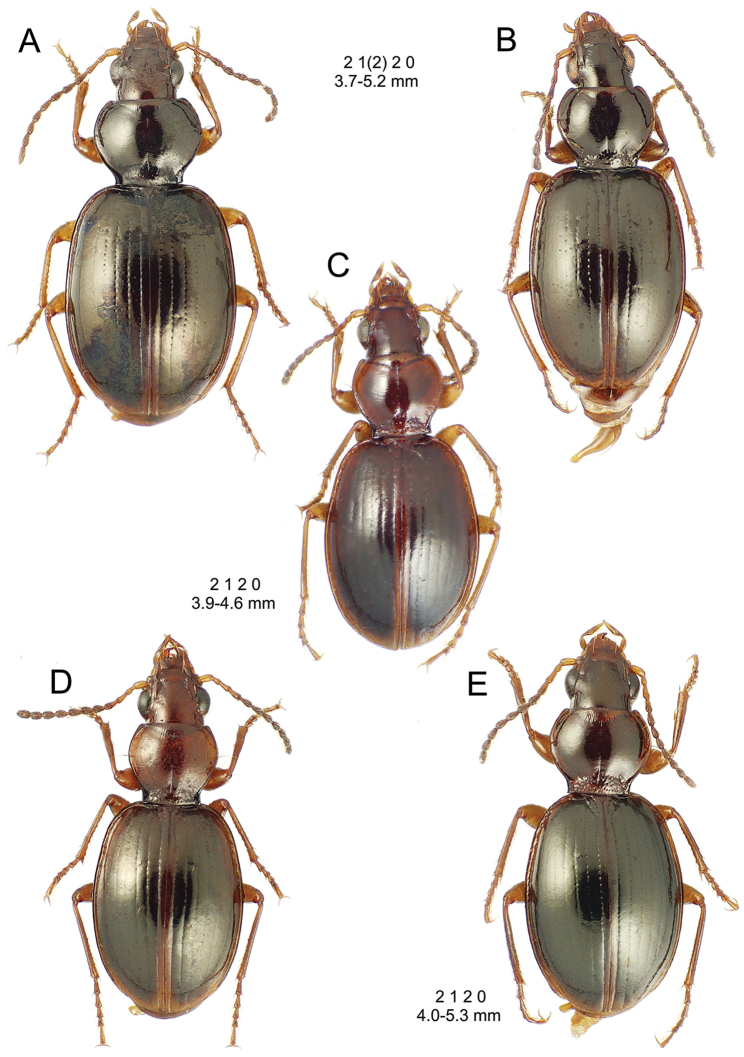
*Mecyclothorax palustris* group species, dorsal habitus view. **A–B** *Mecyclothorax unctus* **A** (Honomanu, 1950 m) **B** Specimen with setose hind pronotal angles (Kīpahulu, 2100 m) **C–D** *Mecyclothorax tauberorum* (Honomanu, 1700 m) **E** *Mecyclothorax pau* (Ke‘anae, 1325 m).

**Figure 159.**
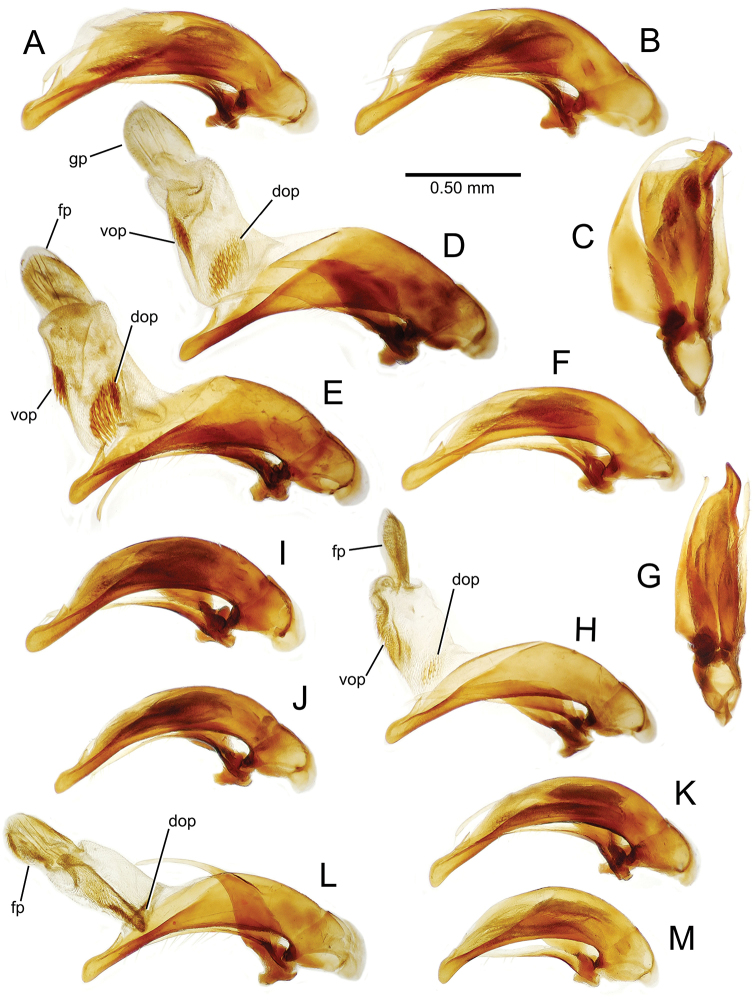
Male aedeagus, *Mecyclothorax unctus* (for abbreviations see Table 2, p. 23). **A** Right view (Leleiwi, 2072 m) **B–D** Right, ventral, and right with sac everted views (Honomanu, 19501980 m) **E** Right view, sac everted (Halemau‘u Tr., 2315 m) **F–H** Right, ventral, and right with sac everted views (Paliku, 1960 m) **I–K** Right views **I** (ESE Kuiki, 2120 m) **J** (Pu‘u Ahulili, 1600 m) **K** (Kīpahulu, 2100 m) **L** Right view sac everted (Kīpahulu, 2100 m) **M** Right view (Kīpahulu, 945 m).

**Figure 160.**
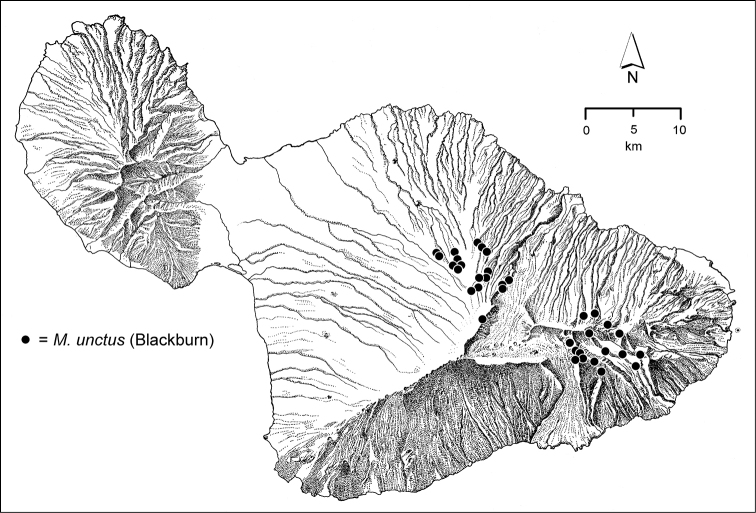
Recorded geographic distribution of *Mecyclothorax unctus*.

Figs 154C , 155C , 158A–B , 159 , 160

Cyclothorax unctus Blackburn 1881: 227.
Thriscothorax unctus , Sharp 1903: 257.
Mecyclothorax unctus , Britton 1948b: 138.

##### Diagnosis.

These beetles exhibit uniformly dark, rufobrunneous to rufopiceous body color (Fig. 158A–B), with the dorsal surface glossy due to the very transverse microsculpture. They are broad-bodied beetles with pronotum transverse, MPW/PL = 1.22–1.33, and elytra basally broad and subquadrate, MEW/HuW = 2.05–2.19. This breadth of body distinguishes them from the *Mecyclothorax palustroides* triad (Figs 149B, 158C–E). Nevertheless, this widely distributed species (Fig. 160) exhibits substantial variation in body size (standardized body length 3.7–5.2 mm), elytral breadth (Fig. 158A–B), and elytral striation. The discal striae 1–3, 1–4, 1–5, or 1–6 may be traceable by deeply impressed striae medially and linear series of isolated punctures laterally. Setal formula 2 1(2) 2 0; the basal pronotal setae may be present in rare instances (e.g. Fig. 158B).

##### Identification

(n = 5). The eyes are moderately convex, ocular ratio = 1.49–1.52, covering much of the moderately protruded ocular lobes, ocular lobe ratio = 0.77–0.83. The pronotum is distinctly constricted basally, MPW/BPW = 1.57–1.63, with the lateral margins subparallel for a short distance anterad the sharply obtuse hind angle, its obtuseness based on the curved basal margin inside the angle. The depressed pronotal median base is covered with >20 isolated punctures each side, the punctures more elongate at the juncture of base and disc. The elytral apex has striae 1, 2 and 8 always present, and an apical portion of stria 7 may also be present just laterad the apex of stria 2. Microsculpture includes: 1, vertex with indistinct transverse mesh, sculpticell breadth 2–3× length; 2, pronotal disc and median with indistinct elongate transverse mesh, sculpticell breadth 2–4× length, to transverse lines not joined into a mesh, the base with glossy areas between areas of microsculpture; 3 elytral disc and apex with distinct, regular transverse mesh, sculpticell breadth 2–3× length. The head and pronotal disc are rufobrunneous with a piceous cast, the elytral disc slightly darker rufopiceous with a cupreous reflection. The legs are contrastedly rufoflavous, and in some individuals the femora are covered medially with a piceous cloud.

**Male genitalia** (n = 24). Aedeagal median lobe variably robust, distance from parameral articulation to tip 3.0–4.2× depth at midlength (Fig. 159D, K); apex extended 1.3–2.0× depth beyond ostial opening (Fig. 159L, M); median lobe shaft symmetrical in ventral view, apex offset to right, with convex left margin sinuously adjoining obliquely blunt apex (Fig. 159C, G); internal sac broad, columnar, with variously developed dorsal ostial microtrichial patch (Fig. 159D–E, H, L), or distinct ventral ostial microtrichial patch (Fig. 159D–E); flagellar plate moderately sized, length 0.38–0.47× parameral articulation-tip distance. There is substantial aedeagal variation across the range of this widespread species (Fig. 160). Some of this variation occurs within regions of the mountain: 1, median lobe robustness and length in the Waikamoi area (Fig. 159A, B); 2, breadth of apical extension near Kuiki (Fig. 159 I–J); 3, length of apical extension in Kīpahulu Valley (Fig. 159K–M). The major pattern involves aedeagi with more well developed ventral ostial microtrichial patches in beetles from Waikamoi versus from localities to the east surrounding Kīpahulu Valley. Further studies of population structure within this geographically disjunct species (Fig. 160) are warranted.

**Female reproductive tract** (n = 1). Bursa copulatrix columnar, parallel sided, length 0.91 mm, breadth 0.34 mm (Fig. 154C); bursal walls translucent, thinly wrinkled; gonocoxite 1 with 3–4 apical fringe setae, 10–11 small setae on medial surface from medioapical angle to base (Fig. 155C); gonocoxite 2 subtriangular, apex subacuminate, base moderately extended laterally, 2 short lateral ensiform setae, apical nematiform setae on medioventral surface at 0.73× gonocoxite length.

##### Lectotype.

Female (BMNH) hereby designated, labeled: mounting platen with Blackburn Maui label (Zimmerman 1957: 210), unctus (on reverse) // Type // Hawaiian Is. Rev. T. Blackburn 1888-30 // LECTOTYPE Cyclothorax unctus Blackburn J.K. Liebherr 1998 (black-margined red label).

##### Distribution and habitat.

*Mecyclothorax unctus* exhibits a densely collected bipartite distribution, with specimens known from throughout the Waikamoi area, and disjunctly from Hanawī through the Hāna Bogs and Kīpahulu Valley, plus the Manawainui Planeze and the eastern margin of Haleakalā Crater (Fig. 160). The species exhibits a broad elevational distribution, with Kīpahulu Valley occupied from 915 m elevation to the valley rim at Kuiki at 2285 m elevation. Elevations of occupied habitat in Waikamoi are less disparate, ranging 1210–2060 m, perhaps due to the drier nature of the subalpine habitats along the leeward edge of the volcano. The species may be very abundant in disturbed ground-level situations, such as feral pig rootings, and may occur under rocks along stream margins. It has been recorded repeatedly from microhabitats associated with ‘ōhi‘a—mossy trunks, humus and leaf litter—but never from situations associated with koa. Other plant substrates from which it has been collected include *Athyrium* fern (‘akolea), *Leptecophylla* (pūkiawe), *Rubus* (‘ākala), and *Vaccinium* (‘ōhelo).

#### (115) Mecyclothorax tauberorum sp. n.

Taxon classificationAnimaliaColeopteraCarabidae

http://zoobank.org/02F96F05-57E2-4185-B598-E91D8229165B

**Figure 161.**
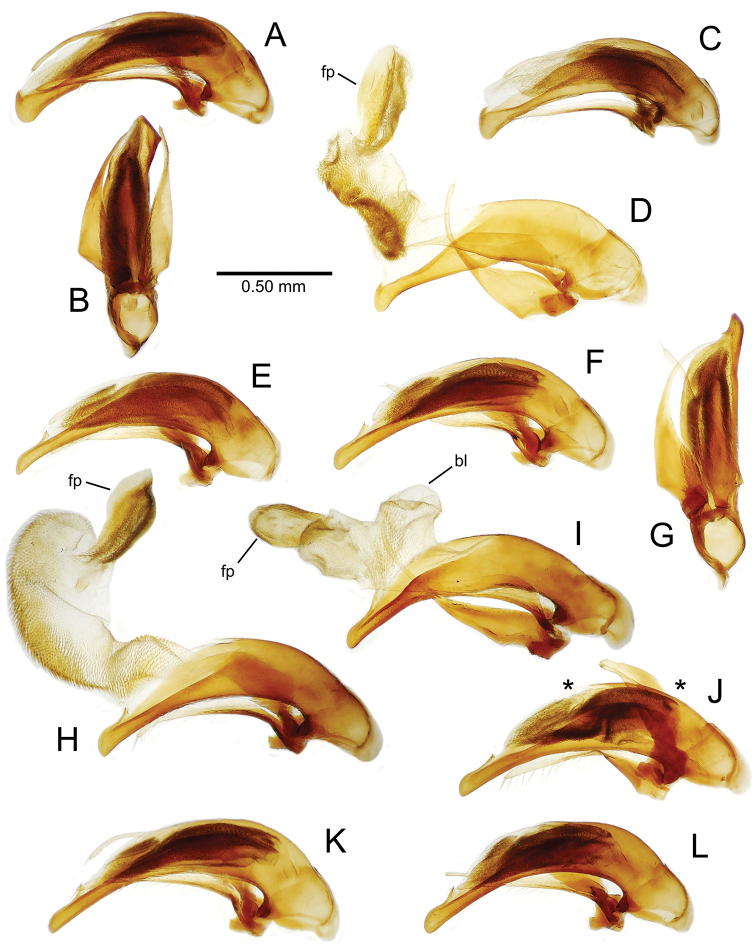
Male aedeagus, *Mecyclothorax palustris* group species (for abbreviations see Table 2, p. 23). **A–D** *Mecyclothorax tauberorum* **A–B** Right and ventral views (Honomanu 1830–1860 m) **C** Right view (Waikamoi, 1275 m) **D** Right view, sac everted (Honomanu, 1700 m) **E–K** *Mecyclothorax pau*. **E** Right view (Ke‘anae, 1325 m) **F–G** Right and ventral views (Kuhiwa, 1590 m) **H–I** Right view, sac everted **H** (Kuhiwa, 1590 m) **I** (Helele‘ike‘oha, 1615 m) **J–L** Right views. **J** (Helele‘ike‘oha, 1615 m; * = distal and basal margins of flagellar plate) **K** (New Greensword Bog, 1850 m) **L** (Pu‘u Ahulili, 1600 m).

Figs 154D , 155D , 158C–D , 161A–D , 163

##### Diagnosis.

This is the second species (Fig. 158C–D), along with *Mecyclothorax palustroides* (Fig. 149B) and *Mecyclothorax pau* (Fig. 158E), that comprise a taxonomic triplet characterized by extremely similar external appearance. Individuals of this species exhibit laterally darker elytra, with intervals 8–9 gradually paler to rufobrunneous compared to the rufopiceous disc; a condition shared with *Mecyclothorax pau*. There may be a broader pale area behind the humerus, but at midlength the lateral intervals 7–8 are indistinctly paler. The pale margin of the elytral apex is narrow, at most extended to a line traversing the subapical sinuations. The pronotal median base appears less depressed relative to the disc due to its less developed punctation, with ~12 small, isolated punctures each side and few or no elongate punctures along the juncture of disc and base. And the elytral microsculpture is an upraised transverse mesh comprising a mixture of isodiametric and transverse sculpticells, their individual surfaces partially upraised so that the surface is alutaceous; a condition shared with *Mecyclothorax palustroides*. The male aedeagal median lobe is bluntly rounded apically (Fig. 161A–D). Setal formula 2 1 2 0. Standardized body length 3.9–4.5 mm, diagnostically smaller than the body size of the sympatrically distributed *Mecyclothorax palustroides*; standardized body length 4.6–5.5 mm.

##### Description

(n = 5). [The above description of *Mecyclothorax palustroides* can serve to describe this species. All diagnostic characters are presented above, so only the various recorded ratios are presented below.]

*Eyes* moderately convex, ocular ratio = 1.48–1.54, ocular lobe ratio = 0.77–0.85. *Pronotum* slightly transverse, MPW/PL = 1.09–1.21, variably constricted basally, MPW/BPW = 1.52–1.71; pronotal apical width greater than basal width, APW/BPW = 1.07–1.15. *Elytra* subovoid, MEW/HuW = 2.13–2.35.

**Male genitalia** (n = 6). Aedeagal median lobe short, squat, distance from parameral articulation to tip 3.2–3.3× depth at midlength (Fig. 161A, C); apex bluntly rounded and not extended beyond ostial opening; median lobe slightly curved rightward near blunt apex, the right and left margins subparallel in ventral view (Fig. 161B); internal sac with ventral surface densely covered with pelage of shaggy microtrichia (Fig. 161D), the sac only 1.5× as long as flagellar plate; flagellar plate large, length 0.61× parameral articulation-tip distance.

**Female reproductive tract** (n = 1). Bursa copulatrix vase shaped, parallel sided with basal constriction, overall length 1.14 mm, midlength breadth 0.40 mm, basal constriction 0.22 mm broad (Fig. 154D): bursal walls translucent, thickly wrinkled; gonocoxite 1 with 4 apical fringe setae and 4 smaller setae on medial surface (Fig. 155D); gonocoxite 2 falcate with narrowly rounded apex, thick basal extension curved at terminus, 2 elongate lateral ensiform setae, apical nematiform setae on medioventral surface at 0.71× gonocoxite length.

##### Holotype.

Male (CUIC) dissected and labeled: HI: Maui Haleakala NW / slope Waikamoi Pres. / trans. 3 @ 1700 m el. / 8-V-1991 scraping / ohia w/ moss & dirt // J.K. Liebherr / collector // 1 // Mecyclothorax tauberorum ♂ #5 / det. J.K. Liebherr 2014 // HOLOTYPE / Mecyclothorax / tauberorum / Liebherr / det. J.K. Liebherr 2015 (black-margined red label).

##### Paratypes.

HI: Maui: Koolau For. Res., Waikamoi flume tanks [label Waikamoi N.C.P.], 1275 m el., 31-v-1993, Tauber/Tauber (CUIC, 11); Waikamoi N.C.P., Honomanu drainage, transect 3, *Metrosideros* with moss, 1700 m el., 13-v-1993 lot 01, Liebherr (CUIC, 1), moss on *Metrosideros* trunk, 1830–1860 m el., 7-v-1991 lot 09, Kavanaugh (CAS, 1), scraping *Metrosideros* bark/moss, 1820–1850, 07-v-1991 lot 02, Liebherr (CUIC, 1), scraping *Metrosideros* humus/moss, 1700 m el., 08-v-1991 lot 03 Liebherr (CUIC, 4).

##### Etymology.

This species is named to honor Dr. Catherine A. Tauber and Prof. Maurice J. Tauber for their collegiality and friendship, and for their contributions to Hawaiian entomology, evolutionary biology, and the study of Neuroptera worldwide.

##### Distribution and habitat.

*Mecyclothorax tauberorum* is known from only three localities; in the Honomanu drainage at 1700 m and 1820–1860 m elevations, and in the Waikamoi drainage at 1275 m elevation (Fig. 163). All specimens have been associated with mossy ‘ōhi‘a trunks, and were collected by scraping bark and moss, or by beating vegetation.

#### (116) Mecyclothorax pau sp. n.

Taxon classificationAnimaliaColeopteraCarabidae

http://zoobank.org/5BC492DA-3558-438B-97A8-28A685BF0186

**Figure 162.**
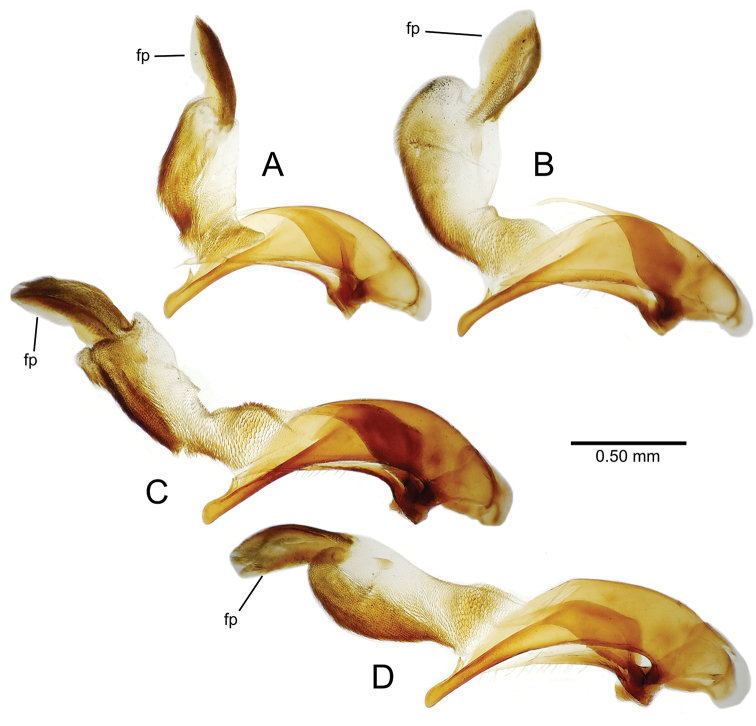
Male aedeagus, *Mecyclothorax pau* (for abbreviations see Table 2, p. 23). **A–D** Right view, sac everted **A** (Kīpahulu, 1800 m) **B** (Kīpahulu, 1200 m) **C** (Kīpahulu W rim, 1850 m) **D** (Pu‘u Ahulili, 1600 m).

**Figure 163.**
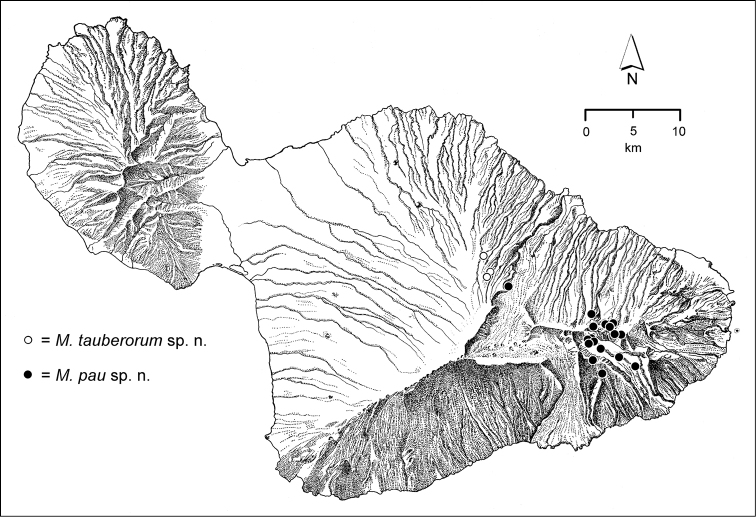
Recorded geographic distributions of *Mecyclothorax palustris* group species.

Figs 154E , 155E , 158E , 161E–K , 162 , 163

##### Diagnosis.

This (Fig. 158E), the third and last species of the *Mecyclothorax palustroides*-based triad to be taxonomically treated, is diagnosed by the following combination: 1, lateral elytral intervals as dark or only gradually paler laterally than the disc, a condition shared with *Mecyclothorax tauberorum* (Fig. 158C–D); 2, pronotal median base distinctly punctate, with ~19 punctures each side, the punctures along the juncture of disc and median base elongate, a condition shared with *Mecyclothorax palustroides* (Fig. 149B); and 3, elytral microsculpture a shallow transverse mesh, sculpticells a mixture of isodiametric in transverse rows and transverse, breadth 2× length, with adjacent sculpticells tiled, their surfaces flat, and thus the surface slightly glossy. Setal formula 2 1 2 0. Standardized body length 4.0–5.3 mm.

##### Description

(n = 5). [The above description of *Mecyclothorax palustroides* can serve to describe this species. All diagnostic characters are presented above, so only the various recorded ratios are presented below.] *Eyes* moderately convex, ocular ratio = 1.51–1.58, ocular lobe ratio = 0.7.8–0.86. *Pronotum* slightly transverse, MPW/PL = 1.16–1.21, variably constricted basally, MPW/BPW = 1.59–1.69; pronotal apical width subequal to greater than basal width, APW/BPW = 0.99–1.13. *Elytra* subovoid, MEW/HuW = 2.19–2.39.

**Male genitalia** (n = 43). Aedeagal median lobe variably robust, distance from parameral articulation to tip 4.4–4.1× depth at midlength (Fig. 161E, I); apex extended 1.6–2.5× depth beyond ostial opening, the extension narrow to moderately broad (Fig. 161E, H); median lobe shaft straight in ventral view, apex offset to right side with left (dorsal) margin convex (Fig. 161G) and tip bluntly rounded; internal sac variable, either a long curved cylinder (Figs 161H, 162A–D) or bilobed with a smaller basal lobe and larger apical lobe (Fig. 161I), the sac surface covered with microspicules only; flagellar plate moderately large, length 0.40× parameral articulation-tip distance in the individual with bilobed sac (Fig. 161I), or large, length 0.54–0.61× parameral articulation-tip distance in the balance of everted individuals with cylindrical sac (Figs 161H, 162). As the sac length can be assessed in uneverted specimens based on the plate’s shadow in backlit photographs (e.g. 161E, F, J–L), it is concluded that only one dissected male (Fig. 161I) has the bilobed sac condition with the associated small flagellar plate. The individual with the bilobed sac (Fig. 161I; lot 19980511.01, CUIC) was one of two males collected at State Camp on Helele‘ike‘oha Stream. A second male collected at that site (Fig. 161J; lot 19980512.11, NMNH) exhibits the long flagellar plate condition (i.e. plate length 0.55× parameral articulation-tip distance, Fig. 161J), Cylindrical-sac, large-plate males are also observed to the north of State Camp at Poouli Cabin (Fig. 161F, H), and to east and south in Hāna Bogs and Kīpahulu Valley (Figs 161K, 162A–B), and on the Manawainui Planeze (Figs 161L, 162C–D). Because both the cylindrical and bilobed conditions are observed sympatrically at State Camp, they are interpreted as two forms of an infraspecific polymorphism. Various hypotheses for the origin of this polymorphism might include: 1, the bilobed male condition is based on a rare allele primitively shared with the sympatrically distributed *Mecyclothorax bilobatus* (Fig. 150E, F); or 2, the bilobed male condition is the product of recent hybridization between the sympatric *Mecyclothorax pau* and *Mecyclothorax bilobatus*.

**Female reproductive tract** (n = 1). Bursa copulatrix vase shaped, parallel sided with basal constriction just distad vagina, overall length 1.05 mm, midlength breadth 0.43 mm, basal constriction 0.25 mm broad (Fig. 154E): bursal walls translucent, thickly wrinkled; gonocoxite 1 with 4 apical fringe setae, a curved seta at medioapical angle and 9–10 smaller setae on medial surface (Fig. 155E); gonocoxite 2 falcate with acuminate apex, the lateral margin slightly expanded near apex, thick basal extension curved at terminus, 2 elongate lateral ensiform setae, apical nematiform setae on medioventral surface at 0.71× gonocoxite length.

##### Holotype.

Male (BPBM) dissected and labeled: Wai Anapanapa / 6600’ // Maui, T.H. / VIII-45 // R.L. Mitchell / Coll. // H. St. John / Collector // HOLOTYPE / Mecyclothorax / pau / Liebherr / det. / J.K. Liebherr 2015 (black-margined red label).

##### Paratypes.

278 specimens (see Appendix).

##### Etymology.

The epithet pau given to this terminal species in the revision is the useful and definitive Hawaiian word for: finished, ended, completed, over, all done, final (Pukui et al. 1975). Given the intense endemism of Haleakalā *Mecyclothorax* species, there is no doubt that this epithet will be superseded in the future by names of presently undescribed species.

##### Distribution and habitat.

*Mecyclothorax pau* is known from an isolated population on the western margin of Ke‘anae Valley (1325 m elevation), and a main massing of populations from Kuhiwa Valley through the Hāna Bogs, Kīpahulu Valley, and the Manawainui Planeze (Fig. 163). The eastern populations occupy habitats 915–2045 m elevation. Nearly all specimens have been collected in association with mossy ‘ōhi‘a, either from trunks or horizontal logs, or by sifting humus and litter from around trees. In a small fraction of collecting events, *Cibotium* (hāpu‘u) tree ferns were living on ‘ōhi‘a nurse logs. The beetles may be beaten from ferns at night.

## Ecological and evolutionary synthesis

**Patterns of diversity.** The Haleakalā *Mecyclothorax* fauna is the most diverse in Hawai‘i at two taxonomic levels (Table 3). At 116 species precinctive to the volcano, the Haleakalā *Mecyclothorax* account for slightly less than 50% of all Hawaiian *Mecyclothorax* species. Moreover, 14 of the 16 species groups are represented in the Haleakalā fauna. Only Moloka‘i at 43 species and 13 species groups approaches the presently known diversity of Haleakalā. It must be remembered however that the current area and summit elevation of the East Moloka‘i volcano are certainly much less than at the volcano’s maximum, as the northern half of the island was lost to the sea in the prodigious Wailau Submarine Slide (Moore et al. 1989), and the entire island has subsided approximately 1500 m from its maximum elevation due to loading of the Pacific Plate by the products of subsequently erupted volcanoes, especially the Kahalawai and Haleakalā volcanoes of Maui (Price and Elliott-Fisk 2004). The biogeographic relationships of the Moloka‘i species preserve an artifact of the pre-landslide volcano, as numerous sister-species pairs precinctively populate the eastern and western portions of forests southeast and southwest of the former crater wall defined by the Pelekunu and Wailau Valley rims (Liebherr 2007).

The Haleakalā *Mecyclothorax* fauna includes all 10 species groups that contain at least one species that exhibits the plesiomorphic setal formula 2 2 2 2; i.e. two supraorbital setae each side, lateral and basal pronotal setae present, two dorsal elytral setae, and both subapical and apical elytral setae. Both Moloka‘i and West Maui lack any members of the *Mecyclothorax montivagus* and *Mecyclothorax sobrinus* groups (Table 3). Given that the flight wing vestigia of *Mecyclothorax montivagus* are the least reduced—i.e. longest and most venated—of any Hawaiian *Mecyclothorax* species, this species is corroborated as the most evolutionarily generalized of all Hawaiian *Mecyclothorax*. Britton (1948b) proposed that *Mecyclothorax montivagus* was very closely related to the Australian *Mecyclothorax punctipennis*, with that species the best candidate for the colonizing propagule reaching Hawai‘i giving rise to the present radiation. Liebherr (2013) concluded that *Mecyclothorax punctipennis* was also the most likely progenitor of the Society Islands *Mecyclothorax* radiation. Character distributions in the Tahitian fauna strongly suggest that this colonization was independent of the Hawaiian colonization event. Thirdly, it is clear that *Mecyclothorax punctipennis* is the closest relative of *Mecyclothorax sculptonotatus* (Enderlein), the single precinctive species that inhabits the St. Paul and Amsterdam Islands of the Indian Ocean (Jeannel 1940, Liebherr unpubl. data). Thus it appears, based on present knowledge, that the very abundant and geographically widespread Australian *Mecyclothorax punctipennis* has independently colonized three isolated island systems, two in the Pacific Ocean and one in the Indian Ocean.

**Figure 164.**
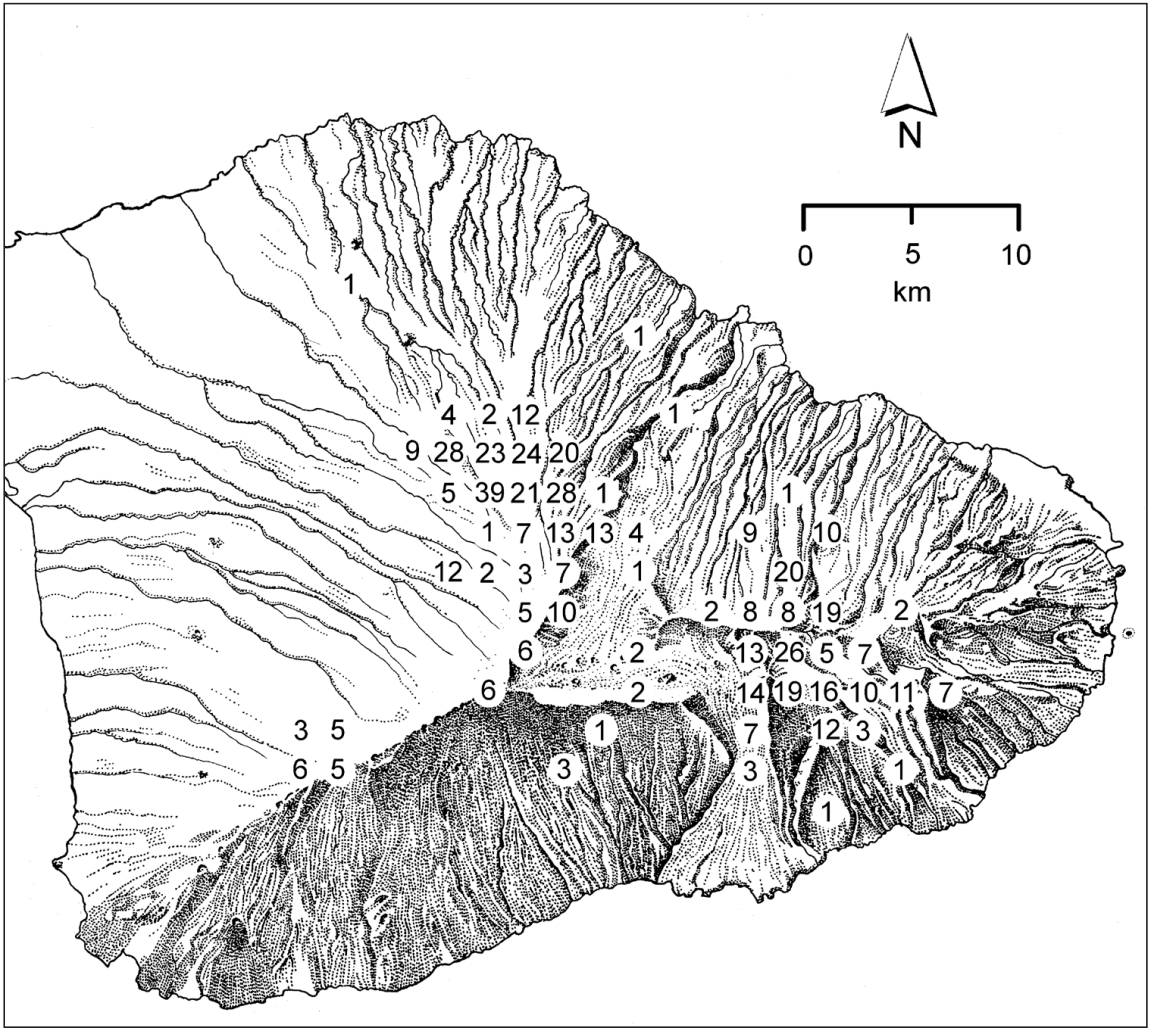
Biogeographic pattern of *Mecyclothorax* diversity on Haleakalā volcano illustrated by the number of *Mecyclothorax* spp. collected within each 1’ latitude × 1’ longitude cell.

Our knowledge of the distribution of species-level diversity within Haleakalā is by necessity constrained and biased by the history of entomological collection. Early access by 19^th^ Century entomologists to the forests of Waikamoi ensured both the maximal collecting effort for that area, as well as early findings of species that are now apparently extinct. Those areas of eastern Haleakalā that were intensively surveyed for the first time during fieldwork supporting this revision were accessed by foot trails, elevational bird-survey transects, and helicopter landings in isolated locales. Nevertheless, a general pattern of species-level diversity can be discerned. Based on information in hand, there are two diversity hotspots for *Mecyclothorax* beetles on Haleakalā. These include the forests of Waikamoi, and the upper elevations in and surrounding Kīpahulu Valley (Fig. 164). In the former, the diagonally adjacent 1’ latitude ×1’ longitude cells corresponding to the historical collecting sites of Olinda and Ukulele Camp have 28 and 39 recorded species respectively. The grid cell to their east that corresponds to mid-elevation Waikamoi Gulch has also had 28 recorded species. In eastern Haleakalā, even given the limited number of collecting days in Kīpahulu Valley where the first *Mecyclothorax* specimens were collected in 1967 by N. Wilson, there are 26 species recorded from the head of Kīpahulu Valley, and 19 from the vicinity of Kuiki. The area near the current Poo‘uli Cabin in the Hanawī Natural Area Reserve of the State of Hawai‘i has had 20 recorded species, and the cell encompassing part of the Hāna Bogs (Fig. 1) 19 species.

**Figure 165.**
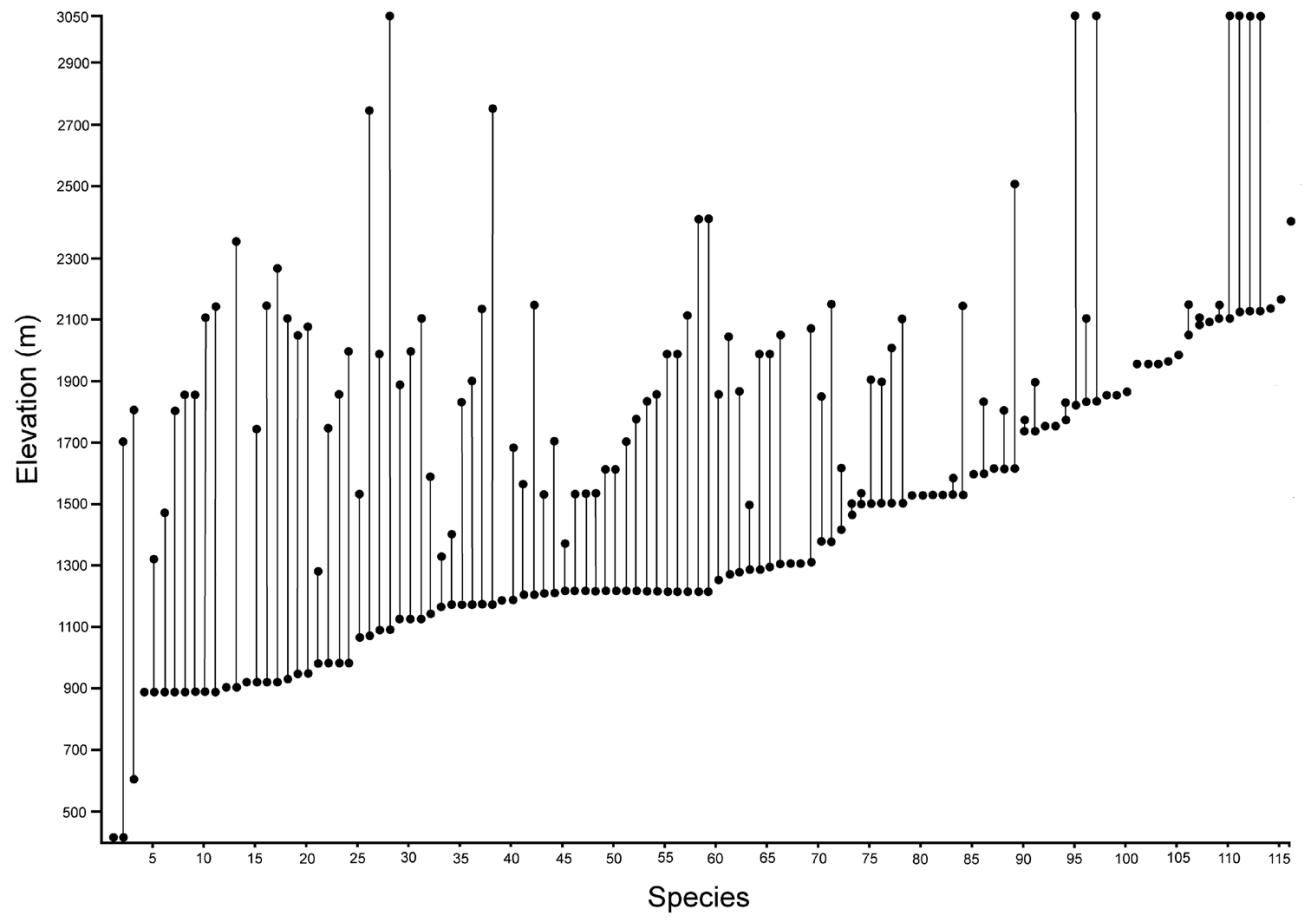
Elevational ranges of localities from which Haleakalā *Mecyclothorax* spp. have been collected, sequentially ordered by lowest then highest elevations of collection localities.

The spatial distribution of diversity can also be viewed by elevation regardless of locality (Fig. 165), with these aggregate data illustrating the general distribution of species up and down the mountain. There are very few species known from below 900 m elevation, and the single species restricted to that low zone—*Mecyclothorax ambulatus*—was collected by R.C.L. Perkins near the turn of the 20^th^ Century. Two other species are known from historical low-elevation sites—*Mecyclothorax notobscuricornis* and *Mecyclothorax manducus*—but have been collected more recently at higher elevations. Most species that are known from several to many localities exhibit substantial elevational ranges. The median vertical range size is approximately 500 m elevation, though 17 species are found at various localities that span more than 1000 m elevation. These vertically promiscuous species are found at various elevations, as their minimum elevation may range from 920 to 1830 m. The elevational ranges exhibited by many species show that it is not unusual for species to span elevationally adjacent plant formations (Gagné and Cuddihy 1990), such as ‘Ōhi‘a/Hapu‘u Tree Fern Forest upwards into Koa/‘Ōhi‘a Wet Montane Forest, and higher still into ‘Ōhi‘a Montane Wet Forest. On drier leeward aspects, species distributions may traverse Montane Dry Shrubland, Subalpine Dry Shrubland, entering the Alpine Dry Shrubland at the 3000 m summit. Species found at the highest elevations, with the exception of *Mecyclothorax krushelnyckyi* known from a single specimen, always occur 900–2100 m lower on the mountain as well, most often in shrublands along the Kula-Waikamoi frontier; e.g. *Mecyclothorax nubicola* (Fig. 79), *Mecyclothorax pusillus* (Fig. 80), and *Mecyclothorax montivagus* (Fig. 132). Other species found at the summit—*Mecyclothorax subconstrictus* (Fig. 79) and *Mecyclothorax rusticus* (Fig. 80)—have also been encountered in Haleakalā Crater. For these species then, climate change may have to drastically impact elevational zonation of habitats before it would cause extirpation of populations. The far greater threat is biotic; that being the distributional expansion of alien invasive social predators such as Argentine Ant, which has been shown to adversely impact native carabid beetles (Loope et al. 1988, Cole et al. 1992, Liebherr and Krushelnycky 2007).

**Table 3.** Diversity of species and species groups for *Mecyclothorax* of the Hawaiian Islands. The most generalized setal formula for any species in each group is presented in the second column. Species groups are arranged by setational formula, with groups at the top of the table including one or more species exhibiting the plesiomorphic setal formula 2 2 2 2 (see text), whereas groups at the bottom of the table contain species with the indicated setal formula, or a formula that is more derived; i.e., fewer setae are present. All 239 species are precinctive to single areas (island or volcano) as indicated in the table header.

Species group	Plesiomorphic setation	O‘ahu	Lāna‘i	Moloka‘i	West Maui	Haleakalā	Hawai‘i I.	Group spp. total
*Mecyclothorax argutor*	2 2 2 2	0	0	6	2	8	2	18
*Mecyclothorax interruptus*	2 2 2 2	0	0	4	2	9	0	15
*Mecyclothorax microps*	2 2 2 2	0	0	1	6	6	2	15
*Mecyclothorax montivagus*	2 2 2 2	0	0	0	0	3	7	10
*Mecyclothorax obscuricornis*	2 2 2 2	0	0	3	4	12	0	19
*Mecyclothorax ovipennis*	2 2 2 2	0	1	9	4	19	4	37
*Mecyclothorax palustris*	2 2 2 2	1	1	3	4	11	4	24
*Mecyclothorax robustus*	2 2 2 2	0	0	2	1	11	4	18
*Mecyclothorax scaritoides*	2 2 2 2	7	0	3	1	9	6	26
*Mecyclothorax sobrinus*	2 2 2 2	0	0	0	0	7	0	7
*Mecyclothorax haleakalae*	2 2 2 1	0	0	2	2	6	0	10
*Mecyclothorax constrictus*	2 1 2 2	0	0	4	0	3	0	7
*Mecyclothorax brevis*	2 1 2 0	5	0	1	0	0	0	6
*Mecyclothorax ducalis*	2 1 2 0	0	0	4	0	6	0	10
*Mecyclothorax vitreus*	2 1 2 0	0	1	0	0	6	1	8
*Mecyclothorax flavomarginatus*	2 1 2 0 or 2 0 2 2	7	0	1	1	0	0	9
**No. species represented on island**		**20**	**3**	**43**	**27**	**116**	**30**	**239**
**No. species groups represented on island**		**4**	**3**	**13**	**10**	**14**	**8**	

When viewing the species-diversity profile in relation to elevation, the greatest *Mecyclothorax* diversity lies between 1200 and 1700 m elevation (Table 4). Depending on soil and precipitation, timberline may occur at 1900 to 2200 m elevation, though isolated trees may support numerous individuals in native grasslands above the limits of closed forest. Nonetheless, diversity at or above timberline is half of that observed at lower elevations supporting closed forest habitats. Of course, elevations within the forested zone are also more subdivided by topographic barriers such as major gaps and deep valleys. Geological barriers such as recent Hana-aged volcanic flows in Kīpahulu Valley and Hanawī (Sherrod et al. 2007) have also vicariated forests at these elevations. Major geological events, such as the filling of Kīpahulu Valley or the opening of Ko‘olau and Kaupō Gaps, may have isolated *Mecyclothorax* populations leading to the numerous present-day species that are restricted to major volcanic blocks such as the Manawainui Planeze or Waikamoi versant (Figs 36, 51, 77, 101). Thus many more species occur across the mountain face at middle elevations, as related species replace each other on the different faces of the mountain (Figs 112, 121, 142).

**Speciation and biogeography.** All evidence points to Hawaiian *Mecyclothorax* having radiated after colonization of Maui Nui, necessitating speciation that resulted in 239 species in 1.9 million years or less (Price and Elliott-Fisk 2004, Sherrod et al 2007). Support for this scenario come from the disharmonic composition of the O‘ahu fauna, within which only 4 of 16 species groups are represented, with these groups all comprising species characterized by derived setal patterns (Table 3). Such a faunal composition is consistent with O‘ahu *Mecyclothorax* being derived from the Moloka‘i fauna (Liebherr and Krushelnycky 2011). Beyond those findings, Kauai lacks *Mecyclothorax* entirely, the ecological role of “small omnivorous mandibulate predator” being played by numerous species of *Bembidion* (Liebherr 2008a). Liebherr (2013) reviewed speciation rates among various rapidly evolving animal groups, estimating that both the Society Island and Hawaiian *Mecyclothorax* radiations have speciated at a rate of about 3.0–3.3 events per million years since their origins 1.4 and 1.8 Ma; greater both that of Hawaiian *Drosophila* (Drosophilidae) pomace flies (DeSalle 1992) or *Laupala* (Gryllidae) crickets (Mendelson and Shaw 2005). Hawaiian insects exhibiting the most rapid speciation are cave *Oliarus* planthoppers (Cixiidae; Wessel et al. 2013), where the speciation rate may be as rapid as 7–8 events per millions years. Liebherr’s (2013) analysis assumed that Hawaiian *Mecyclothorax* colonized the earliest high volcano of Maui Nui; East Moloka‘i dated at 1.9 Ma. That assumption requires that Moloka‘i housed the most generalized Hawaiian *Mecyclothorax* species, though currently it does not. The most generalized species is *Mecyclothorax montivagus* of Haleakalā. Thus the prior analysis assumed that an extremely generalized species existed on Moloka‘i when it was a much larger volcano, but that species subsequently became extinct on Moloka‘i or dispersed to Maui. Conversely, if we restrict our assumptions, and look only at the evidence in hand, the most generalized Hawaiian *Mecyclothorax* species—*Mecyclothorax montivagus*—occurs on a volcano—Haleakalā—that is 1.2 Myr old. Based on that age of origin for the radiation, and using the method of Coyne and Orr (2004), the speciation rate = ln N – ln N_0_ / t = ln 239 – ln 1 / 1.2 = 4.56 (where N = number of present day species, N_0_ = number of colonizing species, and t = time in million years; see Mendelson and Shaw 2005). Using these assumptions then, speciation has occurred about 4.5 times per million years, with the inverse species duration time between speciation events estimated to be only 220,000 years. This species duration is approximately 10% of that observed in Australian *Mecyclothorax*, where fossil deposits aged ~1.84– ~1.56 Ma contain putatively extant species (Snidermann et al. 2009). Such a rapid speciation rate also supports the hypothesis that the extant Australian species, *Mecyclothorax punctipennis*, colonized the Hawaiian Islands 1–2 Ma (Liebherr 2013), as this very abundant and geographically widespread mainland species is likely to have persisted since that time given the durational persistence of its less widespread mainland congeners. Future fossil discoveries of 1–2 Myr old *Mecyclothorax punctipennis* in Australia, or conversely *Mecyclothorax montivagus* or its adelphotaxon on Moloka‘i will, respectively, corroborate or refute the origin of Hawaiian *Mecyclothorax* on Haleakalā. These two alternate findings will therefore substantiate or contradict the current status of Haleakalā as keystone of the entire Hawaiian *Mecyclothorax* radiation.

Although a phylogenetic hypothesis for the Hawaiian *Mecyclothorax* is not at hand, the occurrence of numerous cryptic sibling species complexes on Haleakalā allows preliminary assessment of species-level historical biogeography, including an assessment of the geographical or ecological barriers that may have been associated with speciation. The biogeographical replacement of related species across the windward face of Haleakalā is a fundamental attribute of this radiation. Analyzing the distributions of only those species that could not be confidently diagnosed from each other until the internal male genitalic characters were evaluated, geographic distributions for 27 species representing 11 species complexes may be compared (Table 5). Viewing these various suites of closely related species, it is immediately apparent that speciation has proceeded in the different groups in response or non-response to very different barriers. In 6 of the 11 clades, a species precinctive to the Waikamoi versant stands separate from other species to the east. However in the balance of 5 species suites, populations in Waikamoi are conspecific with those from Hanawī (including Ko‘olau Gap) or the Hāna Bogs. Moving east, 2 species—*Mecyclothorax poouli* and *Mecyclothorax crassuloides*—are distributed across Hanawī and the Hāna Bogs into Kīpahulu Valley. Incongruent with this pattern, two other species pairs have their mutual boundaries situated between Hanawī and the Hāna Bogs. Finally, Manawainui may be populated by precinctive species—*Mecyclothorax ahulili*, *Mecyclothorax antaeus*, and *Mecyclothorax cordaticollaris*—or the species there may also be found in Kīpahulu Valley or the Hāna Bogs. Areas of sympatry, as observed in the *Mecyclothorax kipwilli* complex, are best explained as post-speciation secondary dispersal by one of the species. However aside from this complex, related species are allopatric, though the mutual borders between their adjacent distributions vary among species. These incongruities suggest that speciation has proceeded based on different factors in the different groups. These might include different rates of divergence in different taxa when populations are vicariated by volcanic lava flows. If speciation proceeds quickly after vicariance by, for example, a Hāna-aged volcanic flow (Sherrod et al. 2007), then the species boundary will be congruent with that geological feature. However if the populations diverge more slowly, they may secondarily disperse once forest is reestablished on a volcanic flow, and the historical vicariance disappears before speciation can occur. Divergence, under this scenario, could result in population-level phenomena such as clinal variation that is largely opaque to revisionary, morphologically based systematics.

Two instances of vicariant patterns observed among the cryptic species complexes are based on an ecological criterion; the restriction of particular *Mecyclothorax* species to *Acacia koa* forest habitats. Both *Mecyclothorax cordaticollis* and *Mecyclothorax cordaticollaris* are known only from areas of Koa/‘Ōhi‘a Mesic Forest. The former was collected only by Perkins near Ukulele Camp in Koa forest, the latter collected recently in Koa forest at Kaupō Gap (Fig. 3C). Similarly, *Mecyclothorax bacrionis*, *Mecyclothorax haleakalae*, and *Mecyclothorax simpulum* have all been collected in areas that include Koa trees, with specimens of the latter two species collected ***only*** on Koa trees. In both complexes, absence of any representative species from Hāna Bogs is strictly due to lack of suitable host plant substrate in that area.

A second type of biogeographic pattern is best explained as speciation of peripherally isolated populations in association with occupation of a different elevational habitat zone. *Mecyclothorax perseveratus* was discovered from only two sites among the lower elevation localities housing the geographically widespread *Mecyclothorax perstriatus* (Fig. 14). Analogously, *Mecyclothorax subternus* is known only one site in lower Kuhiwa Valley, along the lower elevational margin of its cryptic sibling species, the geographically widespread and numerically abundant *Mecyclothorax mauiae* (Fig. 71). A third example of such an elevational pattern involves the distribution of *Mecyclothorax reiteratus* among the lower-elevational populations of the more widespread *Mecyclothorax iteratus* (Fig. 106). This latter example requires either clinal speciation across a broad geographic front correlated with elevation, or secondary dispersal of *Mecyclothorax reiteratus* across lower elevation habitats. This species has been collected in ‘Ōhi‘a/Hapu‘u Montane Wet Forest, and so some ecological specialization may explain its distribution, however the presence of populations of the sister species *Mecyclothorax iteratus* surrounding those of the peripherally distributed species suggests that occupation of a particular type of forest cannot explain fully the relative distributions of these species.

**Table 4.** *Mecyclothorax* species-level diversity at different elevations of Haleakalā, summing species occurrences from windward and leeward aspects of the volcano.

Elevation (m)	No. of species
900	12
1100	27
1200	60
1300	61
1500	69
1700	57
1900	39
2100	28
2300	13
2500	10
3050	7

The *Mecyclothorax* fauna of the Southwest Rift at Polipoli Springs includes several examples of vicariant speciation associated with dry leeward habitats. In the *Mecyclothorax constrictus* group, *Mecyclothorax superstriatus* is precinctive to Polipoli whereas *Mecyclothorax perstriatus* is found across the windward face along with its Waikamoi peripherally isolated relative, *Mecyclothorax perseveratus* (Fig. 14). Two other precinctive Polipoli species—*Mecyclothorax aeneipennis* (Fig. 32) and *Mecyclothorax consobrinus* (Fig. 59)—are respectively, sister species of the Waikamoi endemics *Mecyclothorax aeneus* (Fig. 36) and *Mecyclothorax sobrinus*. A second *Mecyclothorax sobrinus* group species, *Mecyclothorax giffardi*, is distributed from Polipoli onto the south slope of Kahikinui (Fig. 56), whereas like-sized members of the group are all in Waikamoi. Thus endemic taxa on the Southwest Rift are consistently related to taxa from the Waikamoi versant. Pleistocene-aged climatic oscillations could be the basis for this vicariance, as the Pleistocene glacial periods were associated with cooler, drier conditions (Hotchkiss et al. 2000).

Intense sympatry within cryptic sibling species complexes is observed repeatedly across this radiation. Within the forests of Waikamoi, two pairs of exceedingly similar species in the *Mecyclothorax scaritoides* group—*Mecyclothorax macrops* and *Mecyclothorax molops*, and *Mecyclothorax scarites* and *Mecyclothorax scaritoides* (Fig. 99)—are all sympatric, or separated by very small geographic distances. At high elevations of the summit and Haleakalā Crater, similar levels of sympatry are observed among two other pairs of exceedingly similar species; *Mecyclothorax apicalis* and *Mecyclothorax parapicalis* (Fig. 67), and *Mecyclothorax pusillus* and *Mecyclothorax rusticus* (Fig. 80). In these instances any allopatry that might have been associated with speciation has been obscured by subsequent changes in the geographical distributions.

The overall pattern of species-level diversity for Haleakalā also includes numerous geographically widespread species that are found across the windward face, and in several instances also the leeward faces of the mountain. The most widespread species, *Mecyclothorax cordithorax* (Fig. 89) is found at the highest elevations surrounding Haleakalā Crater, at Polipoli Springs, and in the montane shrublands west of the windward forest. Three other species—*Mecyclothorax ovipennis* (Fig. 66), *Mecyclothorax laetus* (Fig. 75), and *Mecyclothorax iteratus* (Fig. 106)—are distributed across the windward areas from Waikamoi to Manawainui, but also in a leeward area such as Polipoli or Kahikinui. Six species distributions span the windward face from Waikamoi to Manawainui: 1, *Mecyclothorax perstriatus* (Fig. 14); 2, *Mecyclothorax consanguineus* (Fig. 35); 3, *Mecyclothorax mauiae* (Fig. 71); 4, *Mecyclothorax splendidus* (Fig. 109); 5, *Mecyclothorax nanunctus* (Fig. 157); and 6, *Mecyclothorax unctus* (Fig. 160). In all, 37 of the 116 species—or 32% of the fauna—are distributed in more than one of the areas of endemism defined for the cryptic sibling species complexes of Table 5. Thus for the Haleakalā *Mecyclothorax* fauna, speciation has proceeded in some lineages slower than in others, so that nearly a third of the fauna remains broadly cohesive at the level of species even given significant geographical features such as the Ko‘olau and Kaupō Gaps, Kīpahulu Valley, and Kula. This proportion of widespread species differs dramatically from geographic distributions observed in the Tahitian *Mecyclothorax* fauna, where only 5 of the 84 species of Tahiti Nui—6% of the fauna—are found in more than one area of endemism (Liebherr 2013), those areas defined as the massifs that radiate from the central peak Orohena. Tahitian *Mecyclothorax* diversity is distributed very differently from that of Haleakalā, as it exhibits very high beta diversity; i.e., few shared species across neighboring areas of endemism. In concert with this, single area alpha diversity is lower, with the maximal diversity from any one massif observed on Marau where 31 species are recorded from all habitable elevations. Conversely, a single 1’ latitude × 1’ longitude area in Waikamoi has 39 recorded species. The two types of biogeographical patterns have evolved over approximately the same amount of time; 1.4 Myr for Tahiti and 1.2 Myr for Haleakalā (Liebherr 2013). Moreover, the identical colonizing taxon—*Mecyclothorax punctipennis* of Australia—is hypothesized to be the common ancestor of both radiations. It seems clear that the biogeographic differences between the radiations are due to the much greater geographical subdivision of Tahiti, where the numerous deep, broad valleys isolate narrow ridgelike massifs, apparently limiting the numbers of *Mecyclothorax* species that can coexist on any one massif relative to Haleakalā. These major subdivisions are also associated with nearly complete species replacement from massif to massif.

**Table 5.** Geographical distributions of suites of cryptic sibling species from the windward face of Haleakalā, illustrating incongruent area relationships among various sets of most-closely related species. Areas of endemism into which species distributions are categorized are defined based on terminology of Fig. 1. For purposes of simplified illustration, peripheral features are united with several of the five recognized areas of endemism as follows: 1, Ko‘olau Gap, is united with Hanawī; 2, Kaumakani and Waiho‘i Valley are united with Kīpahulu Valley; 3, Kaupō Gap is united with Manawainui. Figures listed present the geographical distributions of the species. No phylogenetic relationship beyond unresolved monophyly for each suite of species is implied by species positions among the table cells.

Figures	Waikamoi	Hanawī	Hāna Bogs	Kīpahulu	Manawainui
25	*waikamoi*	*poouli*--------------*poouli*--------------*poouli*			*ahulili*
36, 42	*robustus*-----------*robustus*		*haydeni*------------*haydeni*		*antaeus*
40	*cymindicus*			*cymindulus*	
77	*cordaticollis*				*cordaticollaris*
86	*planatus*------------*planatus*			*planipennis*--------*planipennis*	
101	*crassulus*	*crassuloides*--------*crassuloides*--------*crassuloides*			
112	*haleakalae*	*bacrionis*		*simpulum*------------*simpulum*	
121	*kipwilli*------------*kipwilli*-------------*kipwilli*			*kaumakani*-----------*kaumakani*	
			*kuiki*	*kipahulu*	*kuiki*
142	*longidux*-----------*longidux*			*brevidux*--------------*brevidux*	
148	*bicoloris*------------*bicoloris*		*bicoloratus*--------*bicoloratus*		
163	*tauberorum*	*pau*------------------*pau*------------------*pau*-----------------*pau*			

**Conservation and future prospects.** Even though sites sampled in support of this revision (Fig. 6) greatly expanded the 19^th^ Century localities along the upper slope west of Haleakalā Crater and Ke‘anae Valley, much work remains to achieve a full understanding of the phylogenetic relationships and geographical distributions of Haleakalā *Mecyclothorax*. Fully 21 of the 116 species (or 18%) are known from single specimens, with another 5 species known from doubletons (4 of which are represented by 2 specimens of the same sex). Distributional information is necessarily limited for these 26 species, though knowledge of other more commonly encountered species will also benefit from additional field survey. Looking at present distributional knowledge (e.g. Table 5), we may predict that species or populations may be discovered that represent areas of endemism without any present species records. For example, will Koa Forests in Kīpahulu Valley support *Mecyclothorax cordaticollaris*, or another closely related representative species?

This revision establishes a 21^st^ Century baseline for the *Mecyclothorax* diversity of Haleakalā. Although extinction of native Hawaiian species is well documented, Howarth (1990) noted that “The Hawaiian fauna is not inherently fragile (p. 20).” Haleakalā *Mecyclothorax* beetles support this statement, as an assemblage of seven species remain resident at Polipoli Springs, an area originally covered with Koa Mesic Forest, but then denuded and subsequently afforested with alien forest tree species. How much human-mediated extinction has diminished the Hawaiian *Mecyclothorax* fauna remains an open question. Looking at the fauna of the Waikamoi versant during the entirety of entomological research conducted there, we now know that 17 species present in 19^th^ and early 20^th^ Century samples have not been recollected during recent fieldwork commencing in 1991. However we also know that 26 newly described species have been collected during fieldwork in Waikamoi that commenced in 1991. Only continued field survey will allow assessment of these competing views of Haleakalā *Mecyclothorax*. Given the present revision that supports accurate identification of the 116 described species recorded for Haleakalā, and the demonstrated persistence of nearly 100 of those species on the “gigantic mountain (Blackburn 1885)”, Hawaiian *Mecyclothorax* beetles represent a substantial and important source of information concerning the ecological condition of their island home.

## Appendix

Paratype records follow for species described based on more than 25 type specimens. Species sequence and identifying number follow textual presentation.

### (020) *Mecyclothorax consanguineus* sp. n.

261 paratypes. HI: Maui: Haleakala N.P., Haleakala Crater, Holua, 2134 m el., 1-ix-1945, St. John (BPBM, 1), Paliku, pitfall, 1900 m el., 14-15-iii-2002, Takumi (HALE, 1), Halemauu trailhead, pitfall trap, 2438 m el., 13-vi-1975, Burkhart (BPBM, 2), Kipahulu Vy., sifting litter, 1500 m el., 09-v-1991 lot 03, Jessel/Medeiros (CUIC, 17), sifting by day, 2100 m el., 07-v-1991 lot 05, Jessel/Medeiros (CUIC, 12), beating vegetation at night, 2100 m el., 07-v-1991 lot 13, Jessel/Medeiros (CUIC, 1), West Camp, pyrethrin fog *Metrosideros*/moss, 1960 m el., 19-v-1998 lot 01, Polhemus (NMNH, 12), 20-v-1998 lot 01, Polhemus (NMNH, 10), pit crater below West Camp, pyrethrin fog *Metrosideros*/moss, 1845 m el., 19-v-1998 lot 02, Polhemus (NMNH, 1); Kipahulu west rim ESE Kuiki, sift *Metrosideros* humus, 1850 m el., 15-v-1993 lot 02, Liebherr/Medeiros (CUIC, 2), lot 03, Liebherr/Medeiros (CUIC, 1), lot 05, Liebherr/Medeiros (CUIC, 5), 2090 m el., 14-v-1993 lot 04, Liebherr/Medeiros (CUIC, 1), sift moss at treebase, 2090 m el., 14-v-1993 lot 03, Liebherr/Medeiros (CUIC, 1), below Kuiki, sift *Metrosideros* litter, 2135 m el., 16-v-2001 lot 06, Ewing (CUIC, 2), 2145 m el., 16-v-2001 lot 02, Liebherr (CUIC, 6), 2164 m el., 16-v-2001 lot 05, Ewing (CUIC, 1), pyrethrin fog *Metrosideros*/moss, 2145 m el., 16-v-2001 lot 01, Liebherr (CUIC, 1), Haleakala NW upper slope, beating, 1830–1980 m el., 18-viii-1937, Zimmerman (BPBM, 4), Northeast Rift, New Greensword Bog, sifting ohia/styphelia, 1850 m el., 17-v-1993 lot 03, Liebherr/Medeiros (CUIC, 1); Hana For. Res., Heleleikeoha Str. fence camp, pyrethrin fog *Metrosideros*/moss, 1615 m el., 12-v-1998 lot 10, Polhemus (NMNH, 1); Koolau For. Res., Hanawi N.A.R., Frisbee Meadow Camp, woods below, sift *Dubautia* litter, 2072–2099 m el., 19-v-1993 lot 01, Liebherr/Medeiros (CUIC, 3), sift *Metrosideros* humus, 2072–2099 m el., 19-v-1993 lot 02, Liebherr/Medeiros (CUIC, 2), lot 03, Liebherr/Medeiros (CUIC, 2), Koolau Flume Rd., wet forest, yellow pan trap, 1280 m el., vi–viii–2006, Leblanc (UHIM, 2), Koolau Gap, Halehaku, beating *Metrosideros*/moss, 1325 m el., 13-v-1998 lot 11, Polhemus (NMNH, 8), Kula Pipeline Rd., pyrethrin fog *Acacia koa*, 1305 m el., 18-v-2003 lot 10, Polhemus (NMNH, 1), Kula Pipeline Rd., *Cibotium*, 1210 m el., 24-viii-1929, Whitten (BPBM, 2), pyrethrin fog downed log, 1305 m el., 18-v-2003 lot 09, Polhemus (NMNH, 1) pyrethrin fog ohia, 1265 m el., 18-v-2003 lot 08, Polhemus (NMNH, 1); Makawao Flume Rd., ecotone forest, yellow pan trap, 1293 m el., vi–viii–2006 lot 03, Leblanc (UHIM, 1), Waikamoi Flume, 1210 m el., vii-1956 lot 02, Namba (BPBM, 1), viii-1958, Hardy (BPBM, 1), 02—03–i-1986, Perreira (UHIM, 1), 1310 m el., 09-v-1991 lot 05, Kavanaugh (CAS, 1), Waikamoi Gulch to Haipuaena Gulch, under boards/wet ohia for., 1310 m el., 11-iv-1991 lot 01, Liebherr (CUIC, 1), Waikamoi Gulch/Flume, pyrethrin fog ohia/mossy log, 1265 m el., 18-v-2003 lot 07, Polhemus (NMNH, 2), Puu Luau, beating shrubbery, 1665 m el., 28-iv-1945, Zimmerman (BPBM, 1); Waikamoi N.C.P., 1525 m el., 13-viii-1991, Tauber/Tauber (CUIC, 1), 1830 m el., 12-iii-2002 lot 02, Takumi (HALE, 3), Honomanu drainage, Rain Gauge Tr., *Clermontia* flowers, 1615 m el., 24-vii-1965, Hardy (BPBM, 1), Honomanu drainage, transect 20, scraping bark *Acacia koa*, 1798–1860 m el., 13-v-1993 lot 03, Liebherr (CUIC, 1), scraping bark *Metrosideros*, 1859 m el., 13-v-1993 lot 02, Liebherr (CUIC, 1), transect 3, scraping *Acacia koa* bark, 1820–1850 m el., 07-v-1991 lot 03, Liebherr (CUIC, 10), scraping mossy trunk *Metrosideros*, 1820–1850 m el., 07-v-1991 lot 07, Liebherr (CUIC, 14), 1830–1860 m el., 07-v-1991 lot 09, Kavanaugh (CAS, 15), 1950 m el., 07-v-1991 lot 08, Kavanaugh (CAS, 6), sifting litter, 1700 m el., 10-iv-1991 lot 01, Liebherr (CUIC, 18), sifting moss and litter, 1680–1710 m el., 08-v-1991 lot 06, Kavanaugh (CAS, 1), 1680 m el., 08-v-1991 lot 07, Kavanaugh (CAS, 2), 1770–1830 m el., 08-v-1991 lot 08, Kavanaugh (CAS, 9), scraping ohia humus/moss, 1700 m el., 08-v-1991 lot 03, Liebherr (CUIC, 1), Maile Rd., pyrethrin fog mossy log, 1435 m el., 02-v-1998 lot 05, Polhemus (NMNH, 12), Ukulele Pipeline, *Metrosideros* mossy log, 1465 m el., 07-v-1998, lot 07, Polhemus (NMNH, 24), Ukulele Pipeline, above, beating vegetation, at night, 1534–1660 m el., 14-v-1998 lot 03, Liebherr (CUIC, 1), pyrethrin fog *Metrosideros*/moss, 1534 m el., 07-v-1998 lot 02, Liebherr (CUIC, 6), 1560m el., 07-v-1998 lot 05, Liebherr (CUIC, 9), pyrethrin fog mossy ohia log, 1510 m el., 16-v-2003 lot 05, Liebherr (CUIC, 4), on vegetation, 1510 m el., 16-v-2003 lot 07, Haines (CUIC, 6), under logs, 1495–1525 m el., 07-v-1998 lot 03, Liebherr (CUIC, 1), Waikamoi drainage, main access road below Hosmer, on ground in feral pig rooting, 1980 m el., 07-v-1991 lot 01, Liebherr (CUIC, 1).

### (022) *Mecyclothorax antaeus* sp. n.

39 paratypes. HI: Maui: Haleakala N.P., Kaapahu, Ohia camp, 1525 m el., 05-iv-2004, Kaholoa‘a (HALE, 1), Kekuewa Hill, 0.7 km N Puu Ahulili, sift humus/moss, 1600 m el., 16-v-1993 lot 01, Liebherr/Medeiros (CUIC, 2), lot 02, Liebherr/Medeiros (CUIC, 3), lot 03, Liebherr/Medeiros (CUIC, 1), lot 04, Liebherr/Medeiros (CUIC, 2), Kipahulu west rim ESE Kuiki, sift *Metrosideros* humus, 1850 m el., 15-v-1993 lot 02, Liebherr/Medeiros (CUIC, 1), lot 03, Liebherr/Medeiros (CUIC, 4), lot 04, Liebherr/Medeiros (CUIC, 3), lot 05, Liebherr/Medeiros (CUIC, 8), Kipahulu west rim below Kuiki, sift *Metrosideros* humus, 2090 m el., 14-v-1993 lot 02, Liebherr/Medeiros (CUIC, 1), lot 04, Liebherr/Medeiros (CUIC, 1), sift moss at tree base, 2090 m el., 14-v-1993 lot 03, Liebherr/Medeiros (CUIC, 2), on trunk of *Metrosideros*, 2135 m el., 16-v-2001 lot 07, Ewing (CUIC, 1), sift *Metrosideros* litter, 2145 m el., 16-v-2001 lot 02, Liebherr (CUIC, 8), under *Metrosideros* litter, 2135 m el., 16-v-2001 lot 09, Takumi (CUIC, 1). Non type specimen: HI: Maui: Haleakala N.P., Kaapahu, 1200 m el., 08-iv-2004, Kaholoa‘a (HALE, 1).

### (026) *Mecyclothorax haydeni* sp. n.

176 paratypes. HI: Maui: Haleakala N.P. Kipahulu Vy., beat vegetation at night, 1500 m el., 09-v-1991 lot 04, Jessel/Medeiros (CUIC, 1), sift litter, 1500 m el., 09-v-1991 lot 03, Jessel/Medeiros (CUIC, 14), sift litter by day, 1800 m el., 08-v-1991, lot 04, Jessel/Medeiros (CUIC, 9), 2100 m el., 07-v-1991 lot 05, Jessel/Medeiros (CUIC, 2), Central Pali Tr., beat vegetation in day, 1200 m el., 29-iv-1991 lot 01, Liebherr/Medeiros (CUIC, 1), beat vegetation at night, 1200 m el., 29-iv-1991 lot 04, Liebherr/Medeiros (CUIC, 1), sift leaf/moss litter, 1200 m el., 29-iv-1991 lot 03, Liebherr/Medeiros (CUIC, 2), Mauka Ridge, pyrethrin fog mossy ohia, 2045 m el., 21-v-1998 lot 01, Polhemus (NMNH, 3), Palikea Camp, 1300 m el., 20-iv-2000 lot 01, Takumi (HALE, 1), West Camp, pyrethrin fog *Metrosideros*/moss, 1960 m el., 19-v-1998 lot 01, Polhemus (NMNH, 16), 20-v-1998 lot 01, Polhemus (NMNH, 29), pit crater below West Camp, pyrethrin fog *Metrosideros*/moss, 1845 m el., 19-v-1998 lot 02, Polhemus (NMNH, 31), Northeast Rift, New Greensword Bog, sift *Metrosideros*/styphelia, 1850 m el., 17-v-1993 lot 02, Liebherr/Medeiros (CUIC, 1); Hana For. Res., Heleleikeoha Str. State Fence Camp, pyrethrin fog *Metrosideros*/*Astelia*, 1615 m el., 11-v-1998 lot 02, Liebherr (CUIC, 2), pyrethrin fog *Metrosideros*/moss, 1615 m el., 11-v-1998 lot 01, Liebherr (CUIC, 7), 11-v-1998 lot 06, Polhemus (NMNH, 22), 12-v-1998, lot 03, Liebherr (CUIC, 3), lot 04, Liebherr (CUIC, 3), lot 10, Polhemus (NMNH, 7), ridge E Heleleikeoha Str. State Fence Camp, under logs, 1740 m el., 11-v-1998 lot 04, Ewing (CUIC, 1), ridge S Heleleikeoha Str. State Fence Camp, pyrethrin fog *Metrosideros*/moss, 1795 m el., 12-v-1998 lot 11, Polhemus (NMNH, 11), Horseshoe Bog nr. Haleakala N.P., pyrethrin fog *Metrosideros*/moss, 1830 m el., 12-v-1998 lot 05, Liebherr (CUIC, 1), Kaumakani Peak, pyrethrin fog vegetation, 1127 m el., 07-vi-1999 lot 05, Polhemus (NMNH, 3), 08-vi-1999 lot 05, Polhemus (NMNH, 1), lot 07, Polhemus (NMNH, 3), 1135 m el., 07-vi-1999 lot 01, Polhemus (NMNH, 1).

### (051) *Mecyclothorax mauiae* sp. n.

817 paratypes. HI: Maui: Haleakala N.P., Kaapahu, 1200 m e., 08-iv-2004, Kaholoa‘a (HALE, 4), 1250 m el., 07-iv-2004, Kaholoa‘a (HALE, 9), Kekuewa Hill, 0.7 km N Puu Ahulili, sift humus/moss, 1600 m el., 16-v-1993 lot 01, Liebherr/Medeiros (CUIC, 7), lot 02, Liebherr/Medeiros (CUIC, 5), lot 03, Liebherr/Medeiros (CUIC, 9), lot 04, Liebherr/Medeiros (CUIC, 5), streambank, under rocks, 1600 m el., 16-v-1993 lot 05, Liebherr/Medeiros (CUIC, 3), on vegetation at night, 1600 m el., 16-v-1993 lot 06, Liebherr/Medeiros (CUIC, 1), Kipahulu Vy., sifting litter, 1500 m el., 09-v-1991 lot 03, Jessel/Medeiros (CUIC, 21), sifting litter, 1800 m el., 08-v-1991 lot 04, Jessel/Medeiros (CUIC, 9), sifting litter by day, 2100 m el., 07-v-1991 lot 05, Jessel/Medeiros (CUIC, 5), beat vegetation at night, 1500 m el., 09-v-1991 lot 04, Jessel/Medeiros (CUIC, 13), beat vegetation at night, 1900 m el., 08-v-1991 lot 05, Jessel/Medeiros (CUIC, 5), Camp 2, 1250 m el., 18-20-viii-1967, Wilson (BPBM, 1), Central Pali Tr., beating vegetation in day, 1200 m el., 29-iv-1991 lot 01, Liebherr/Medeiros (CUIC, 1), sift leaf/moss litter, 915 m el., 30-iv-1991 lot 03, Liebherr/Medeiros (CUIC, 15), sift leaf/moss litter, 1200 m el., 29-iv-1991 lot 03, Liebherr/Medeiros (CUIC, 13), beat vegetation at night, 1200 m el., 29-v-1991 lot 04, Liebherr/Medeiros (CUIC, 3), Delta Camp, 939 m el., 27-iv-2004 lot 01, Kaholoa‘a (HALE, 1), Mauka Ridge, pyrethrin fog mossy ohia, 2045 m el., 21-v-1998, Polhemus (NMNH, 13), Palikea Camp, 1250 m el., 23-iv-1997 lot 03, Takumi (HALE, 1), Transect 17, 1190 m el., 10-iv-2001 lot 01, Takumi (HALE, 1), West Camp, pyrethrin fog *Metrosideros*/moss, 1960 m el., 19-v-1998 lot 01, Polhemus (NMNH, 1), pit crater below West Camp, pyrethrin fog *Metrosideros*/moss, 1845 m el., 19-v-1998 lot 02, Polhemus (NMNH, 16), Kipahulu west rim ESE Kuiki, sift *Metrosideros* humus, 1850 m el., 15-v-1993 lot 02, Liebherr/Medeiros (CUIC, 2), lot 03, Liebherr/Medeiros (CUIC, 14), lot 04, Liebherr/Medeiros (CUIC, 1), lot 05, Liebherr/Medeiros (CUIC, 18), Northeast Rift, Big Bog, 1670 m el., 17-v-1991 lot 01, Mingard/Medeiros/White/Jessel (CUIC, 2), Midcamp Bog Cabin, sift *Metrosideros* humus, 1665 m el., 18-v-1993 lot 01, Liebherr/Medeiros (CUIC, 3), lot 04, Liebherr/Medeiros (CUIC, 2); Hana For. Res., Heleleikeoha Str. State Fence Camp, pyrethrin fog *Cibotium*, 1615 m el., 12-v-1998 lot 02, Liebherr (CUIC, 2), pyrethrin fog *Metrosideros*/*Astelia*, 1615 m el., 11-v-1998 lot 02, Liebherr (CUIC, 2), pyrethrin fog *Metrosideros*/moss, 1615 m el., 11-v-1998 lot 01, Liebherr (CUIC, 15), lot 06, Polhemus (NMNH, 26), 12-v-1998 lot 03, Liebherr (CUIC, 3), lot 10, Polhemus (NMNH, 7), under streamside vegetation, 1615 m el., 11-v-1998 lot 03, Liebherr (CUIC, 3), ridge S Heleleikeoha Str. State Fence Camp, pyrethrin fog *Metrosideros*/moss, 1795 m el., 12-v-1998 lot 11, Polhemus (NMNH, 33), Horseshoe Bog nr. Haleakala N.P., sift *Myrsine*/*Dubautia*, 1830 m el., 12-v-1998 lot 06, Liebherr (CUIC, 1), Kaumakani Peak, pyrethrin fog vegetation, 1127 m el., 07-vi-1999 lot 05, Polhemus (NMNH, 1), 1135 m el., 07-vi-1999 lot 01, Polhemus (NMNH, 1), 1160 m el., 08-vi-1999 lot 02, Polhemus (NMNH, 5), lot 03, Polhemus (NMNH, 1), 1165 m el., 08-vi-1999 lot 04, Polhemus (NMNH, 2); Koolau For. Res., Flume Rd., scraping *Acacia koa* bark, 1310 m el., 15-v-2003 lot 01, Liebherr (CUIC, 1), Hanawi N.A.R., Kopiliula Str., pyrethrin fog *Acacia koa* trunk, 1127 m el., 03-v-1998 lot 02, Liebherr (CUIC, 5), pyrethrin fog *Cibotium*, 1127 m el., 03-v-1998 lot 06, Liebherr (CUIC, 2), pyrethrin fog *Cibotium* + *Clermontia*, 1127 m el., 03-v-1998 lot 07, Polhemus (NMNH, 3), sift *Metrosideros* litter, 1137 m el., 04-v-1998 lot 01, Liebherr (CUIC, 2), pyrethrin fog *Metrosideros*/moss, 1170 m el., 04-v-1998 lot 07, Polhemus (NMNH, 5), pyrethrin fog ohia/moss/uluhe, 1127 m el., 03-v-1998 lot 08, Polhemus (NMNH, 10), beating uluhe fern, 1127 m el., 03-v-1998 lot 05, Liebherr (CUIC, 1), Kuhiwa Vy. E rim, dead fronds *Cibotium chamissoi*, 900 m el., 10-vi-1999 lot 07, Ewing (CPEC, 1), pyrethrin fog *Cibotium*, 880 m el., 10-vi-1999 lot 05, Polhemus (NMNH, 3), 915 m el., 10-vi-1999 lot 02, Polhemus (NMNH, 14), lot 03, Polhemus (NMNH, 12), lot 04, Polhemus (NMNH, 7), pyrethrin fog *Metrosideros*, 880 m el., 09-vi-1999 lot 01, Polhemus (NMNH, 2), lot 02, Polhemus (NMNH, 2), lot 04, Polhemus (NMNH, 7), lot 05, Polhemus (NMNH, 1), lot 07, Polhemus (NMNH, 27), lot 08, Polhemus (NMNH, 13), lot 09, Polhemus (NMNH, 9), pyrethrin fog steep streambank, 880 m el., 10-vi-1999 lot 01, Polhemus (NMNH, 8), Kuhiwa Vy., Poouli Cabin, beating vegetation, 1590 m el., 05-v-1998 lot 04, Ewing (CUIC, 1), pyrethrin fog *Metrosideros*/*Cibotium*, 1590 m el., 05-v-1998 lot 02, Liebherr (CUIC, 3), 06-v-1998 lot 02, Liebherr (CUIC, 4), pyrethrin fog *Metrosideros*/moss, 1590 m el., 05-v-1998 lot 01, Liebherr (CUIC, 24), lot 03, Polhemus (NMNH, 70), 06-v-1998 lot 01, Liebherr (CUIC, 20), lot 06, Polhemus (NMNH, 10), pyrethrin fog ohia roots/trunk, 1590 m el., 06-v-1998 lot 07, Polhemus (NMNH, 16), Kuhiwa Vy., pig fence helipad [=Poouli Cabin], sift *Metrosideros* humus, 1585 m el., 21-v-1993 lot 01, Liebherr/Medeiros (CUIC, 2), lot 02, Liebherr/Medeiros (CUIC, 3), lot 03, Liebherr/Medeiros (CUIC, 3), lot 04, Liebherr/Medeiros (CUIC, 1), middle Hanawi Str. Tributary, 880 m el., 12-xi-1992 lot 01, Polhemus (BPBM, 2), Ke‘anae Pali, *Astelia veratroides* 1524 m el., 19-vii-1919 lot 02, Timberlake (UCRC, 1), Koolau Gap, Halehaku, beating *Metrosideros*/moss, 1325 m el., 13-v-1998 lot 11, Polhemus (NMNH, 16), pyrethrin fog *Cibotium*/log, 1325 m el., 13-v-1998 lot 03, Liebherr (CUIC, 5), pyrethrin fog *Metrosideros*, 1325 m el., 13-v-1998 lot 04, Liebherr (CUIC, 3), lot 05, Liebherr (CUIC, 5), pyrethrin fog *Metrosideros*/moss, 1325 m el., 13-v-1998 lot 01, Liebherr (CUIC, 12), lot 02, Liebherr (CUIC, 11), Kula Pipeline Rd., 1270 m el., vii-1956, Hardy (BPBM, 1), ferns, 1210 m el., 25-viii-1929 lot 02, Swezey (BPBM, 2), pyrethrin fog *Acacia koa*, 1170 m el., 15-v-1998 lot 03, Liebherr (CUIC, 2), 1305 m el., 18-v-2003 lot 10, Polhemus (NMNH, 17), pyrethrin fog log, 1305 m el., 18-v-2003 lot 09, Polhemus (NMNH, 10), pyrethrin fog *Metrosideros*, 1265 m el., 18-v-2003 lot 08, Polhemus (NMNH, 33), pyrethrin fog *Metrosideros*/moss, 1170 m el., 15-v-1998 lot 06, Polhemus (NMNH, 24), pyrethrin fog trunk + log, 1170 m el., 15-v-1998 lot 04, Liebherr (CUIC, 15), wet forest, yellow pan trap, 1183–1280 m el., vi-viii-2006 lot 02, Leblanc (UHIM, 6), Kula Pipeline Rd. at Waikamoi Gulch, West side, pyrethrin fog *Metrosideros*/*Cibotium*, 975 m el., 15-v-1998 lot 01, Liebherr (CUIC, 1), pyrethrin fog *Metrosideros*/moss, 965 m el., 15-v-1998 lot 05, Polhemus (NMNH, 2), Waikamoi Gulch, 1210 m el., 10-viii-1958, Hardy (BPBM, 1), Waikamoi Flume at Waikamoi Gulch, beat/scrape *Metrosideros*, beat/scrape, 1310 m el., 26-v-1997 lot 06, Liebherr (CUIC, 5), pyrethrin fog *Metrosideros* mossy log, 1265 m el., 18-v-2003 lot 07, Polhemus (NMNH, 7), sift *Metrosideros*/moss, 1310 m el., 09-v-1991 lot 02, Liebherr (CUIC, 12), Waikamoi Flume from Waikamoi to Haipuaena Gulch, under boards/wet *Metrosideros* for., 1310 m el., 11-iv-1991 lot 01, Liebherr (CUIC, 6); Olinda, 1210 m el., 01-viii-1932, Krauss (BPBM, 1), Waikamoi Flume at Waikamoi Gulch, 1210 m el., 19-vii-1965, Beardsley (BPBM, 1), 1305 m el., 03-vi-1996 lot 01, Ewing (CPEC, 1), 1310 m el., 09-v-1991 lot 05, Kavanaugh (CAS, 6), beat/scrape *Metrosideros*, 1310 m el., ferns, 1372 m el., 14-i-1926, Swezey (BPBM, 1), under boards/wet *Metrosideros* for., 1310 m el., 09-v-1991 lot 01, Liebherr (CUIC, 1), Waikamoi Flume, Waikamoi Gulch to Haipuaena Gulch, under boards/wet *Metrosideros* for., 1310 m el.; Makawao For. Res., 1737 m el., 15-vi-1975, Burkhart (BPBM, 1); Waikamoi N.C.P., pyrethrin fog *Metrosideros*/moss, 1750 m el., 12-iii-2002, Takumi (HALE, 1), Honomanu drainage, transect 3, *Metrosideros*, mossy trunk, 1950 m el., 07-v-1991 lot 08, Kavanaugh (CAS, 1), sifting moss and litter, 1680 m el., 08-v-1991 lot 07, Kavanaugh (CAS, 4), Ukulele Pipeline, 1524 m el., 13-vii-1919, Timberlake (UCRC, 2).

### (053) *Mecyclothorax flaviventris* sp. n.

35 paratypes. HI: Maui: Haleakala N.P., Haleakala Crater, Paliku, 1850 m el., 17-iii-1998, Takumi (BPBM, 1), 27-v-1998, Takumi (BPBM, 1), 30-vi-1998, Takumi (BPBM, 1), pitfall trap, 1950 m el., 01-vii-1998, Takumi (BPBM, 1; HALE, 1), Paliku Cabin, beating vegetation, 1960 m el., 15-v-2001 lot 01, Liebherr (CUIC, 1), West Camp, 1859 m el., 17-vii-1983 lot 03, Howarth/Gagné (BPBM, 1), pyrethrin fog *Metrosideros*/moss, 1960 m el., 19-v-1998 lot 01, Polhemus (NMNH, 3), 20-v-1998 lot 01, Polhemus (CUIC, 1; NMNH, 7), pit crater below West Camp, pyrethrin fog *Metrosideros*/moss, 1845 m el., 19-v-1998 lot 02, Polhemus (NMNH, 1), Kipahulu west rim ESE Kuiki, sift *Metrosideros* humus, 1850 m el., 15-v-1993 lot 04, Liebherr/Medeiros (CUIC, 1), Northeast Rift, New Greensword Bog, sift *Metrosideros* humus, 1850 m el., 17-v-1993 lot 05, Liebherr/Medeiros (CUIC, 1); Koolau For. Res., Hanawi N.A.R., woods below Frisbee Meadow Camp, beat *Metrosideros* at night, 2072–2099 m el., 19-v-1993 lot 06, Liebherr/Medeiros (CUIC, 2), sift *Metrosideros* humus, 2072–2099 m el., 19-v-1993 lot 02, Liebherr/Medeiros (CUIC, 3), lot 03, Liebherr/Medeiros (CUIC, 5), lot 04, Liebherr/Medeiros (CUIC, 4).

### (067) *Mecyclothorax planatus* sp. n.

91 paratypes. HI: Maui: Haleakala N.P., Halemauu trailhead, pitfall trap 2438 m el., 13-vi-1975, Burkhart (BPBM, 2), Hosmer’s Grove, 2042 m el., 13-vi-1975, Burkhart (BPBM, 1), Hosmer’s Grove fence, 2050 m el., 17-xii-2004, Kaholoa‘a (HALE, 2), NW upper slope, beating, 1830–1980 m el., 18-viii-1937, Zimmerman (BPBM, 17); Koolau For. Res., Koolau Gap at Halehaku, pyrethrin fog *Cibotium* + mossy nurse log, 1325 m el., 13-v-1993 lot 03, Liebherr (CUIC, 1), pyrethrin fog mossy ohia, 1325 m el., 13-v-1993 lot 04, Liebherr (CUIC, 1), Waikamoi Gulch, 1524 m el., 13-viii-1991 lot 01, Tauber/Tauber (CUIC, 1), Waikamoi-Maile Tr., ecotone forest, yellow pan trap 1426–1573 m el., vi-viii-2006, Leblanc (UHIM, 5); Makawao For. Res., 1738 m el., 15-vi-1975, Burkhart (BPBM, 4), Maile Rd., scraping bark *Metrosideros*, 1310 m el., 26-v-1997 lot 08, Liebherr (CUIC, 1), Olinda, 1210 m el., 1936, Munro (BPBM, 1), Olinda-Makawao, 09-x-1968, Lokse (HNHM, 1), near Puu Luau, beating *Sadleria*, 1665 m el., 28-iv-1945, Zimmerman (BPBM, 4), beating *Suttonia*, 1665 m el., 28-iv-1945, Zimmerman (BPBM, 1), beating shrubbery, 1665 m el., 28-iv-1945, Zimmerman (BPBM, 2), under rocks, 1665 m el., 28-iv-1945, Zimmerman (BPBM, 2), 1830 m el., 27-iv-1945, Zimmerman (BPBM, 2), Puu Nianiau, 29-v-1956, Van Zwaluwenberg (BPBM, 2), near Puu Nianiau, 24-iv-1945, Zimmerman (BPBM, 2); Waikamoi N.C.P., pyrethrin fog *Metrosideros*/moss, 1750 m el., 12-iii-2002, Takumi (HALE, 5), sample 06, *Acacia koa*, bark trap, 20-v-2011, Peck (BPBM, 1), sample 06, *Metrosideros*, bark trap 21-v-2011 lot 04, Peck (BPBM, 4), sample 07, *Metrosideros*, bark trap, 21-v-2011 lot 05, Peck (BPBM, 1), sample 08, *Metrosideros*, bark trap, 20-v-2011 lot 06, Peck (BPBM, 1), sample 10, *Metrosideros*, bark trap, 20-v-2011 lot 08, Peck (BPBM, 1), Honomanu drainage, transect 3, scraping bark *Acacia koa*, 1820 m el., 07-v-1991 lot 03, Liebherr (CUIC, 1), *Metrosideros*/moss, 1700 m el., 13-v-1993 lot 01, Liebherr (CUIC, 1), *Metrosideros*/moss ex trunk 1830–1860 m el., 07-v-1991 lot 07, Liebherr (CUIC, 3), scraping *Metrosideros* bark, 1700 m el., 08-v-1991 lot 01, Liebherr (CUIC, 1), scraping *Metrosideros* bark/moss, 1820–1850 m el., 07-v-1991 lot 02, Liebherr (CUIC, 1), scraping *Metrosideros* humus/moss, 1700 m el., 08-v-1991 lot 03, Liebherr (CUIC, 3), sifting litter, 1700 m el., 10-iv-1991 lot 01, Liebherr (CUIC, 4), sifting moss and litter, 1680–1710 m el., 08-v-1991 lot 06, Kavanaugh (CAS, 2), 1770–1830 m el., 08-v-1991 lot 08, Kavanaugh (CAS, 1), Honomanu drainage, upper arm, treading streamside vegetation, 1950 m el., 07-v-1991 lot 06, Liebherr (CUIC, 1), Maile Road, above, pyrethrin fog mossy log, 1435 m el., 02-v-1998 lot 05, Polhemus (NMNH, 1), sifting litter, 1470 m el., 02-v-1998 lot 03, Ewing (CUIC, 3), under logs on ground, 1494 m el., 16-v-2003 lot 06, Liebherr (CUIC, 1), Ukulele Pipeline, above, beat vegetation at night, 1534–1660 m el., 14-v-1998 lot 03, Liebherr (CUIC, 3), mossy *Metrosideros* log, 1465 m el., 07-v-1998 lot 07, Polhemus (NMNH, 1), pyrethrin fog *Metrosideros*/moss, 1560 m el., 07-v-1998 lot 05, Liebherr (CUIC, 1).

### (087) *Mecyclothorax splendidus* sp. n.

108 paratypes. HI: Maui: Haleakala N.P., Kekuewa Hill, 0.7 km N Puu Ahulili, sift humus/moss, 1600 m el., 16-v-1993 lot 08, Liebherr/Medeiros (CUIC, 2), vegetation at night, 1600 m el., 16-v-1993 lot 06, Liebherr/Medeiros (CUIC, 4), Kipahulu Vy., beat vegetation at night, 1900 m el., 08-v-1991 lot 05, Jessel/Medeiros (CUIC, 2), sift litter, 1800 m el., 08-v-1991 lot 04, Jessel/Medeiros (CUIC, 1), Camp 3 to Rim, 1980–2260 m el., 21-25-viii-1967, Wilson (BPBM, 1), Central Pali Tr., beat vegetation at night, 915 m el., 30-iv-1991 lot 04, Liebherr/Medeiros (CUIC, 2), 1200 m el., 29-iv-1991 lot 04, Liebherr/Medeiros (CUIC, 1), Charlie Camp, mossy tree trunks at night, 1433 m el., 25-ii-1984, Gagné (BPBM, 1), Palikea Camp, 1300 m el., 20-iv-2000, Takumi (HALE, 1), West Camp, pyrethrin fog *Metrosideros*/moss, 1960 m el., 19-v-1998 lot 01, Polhemus (NMNH, 1), 20-v-1998, lot 01, Polhemus (NMNH, 3), Kipahulu west rim below Kuiki, beat *Metrosideros* at night, 2105 m el., 14-v-1993 lot 07, Liebherr/Medeiros (CUIC, 1), 15-v-1993 lot 01, Liebherr/Medeiros (CUIC, 1), pyrethrin fog *Metrosideros*/moss, 2145 m el., 16-v-2001 lot 01, Liebherr (CUIC, 1), sift *Metrosideros* humus, 2090 m el., 14-v-1993 lot 04, Liebherr/Medeiros (CUIC, 1), sift *Metrosideros* moss, 2090 m el., 14-v-1993 lot 01, Liebherr/Medeiros (CUIC, 2), ESE Kuiki, beating vegetation at night, 1850 m el., 15-v-1995 lot 06, Liebherr/Medeiros (CUIC, 1), Northeast Rift, Midcamp Bog Cabin, beat *Metrosideros*/styphelia at night, 1665 m el., 18-v-1993 lot 05, Liebherr/Medeiros (CUIC, 1), sift *Metrosideros* humus, 1665 m el., 18-v-1993 lot 01, Liebherr/Medeiros (CUIC, 1), lot 03, Liebherr/Medeiros (CUIC, 2), lot 04, Liebherr/Medeiros (CUIC, 1), New Greensword Bog, beat vegetation at night, 1850 m el., 17-v-1993 lot 06, Liebherr/Medeiros (CUIC, 6), sift *Metrosideros* humus, 1850 m el., 17-v-1993 lot 04, Liebherr/Medeiros (CUIC, 2), lot 05, Liebherr/Medeiros (CUIC, 2); Hana For. Res., Heleleikeoha Str. State Fence Camp, pyrethrin fog *Metrosideros*/moss, 1615 m el., 11-v-1998 lot 01, Liebherr (CUIC, 1), lot 06, Polhemus (NMNH, 2), 12-v-1998 lot 01, Liebherr (CUIC, 4), lot 03, Liebherr (CUIC, 1), lot 04, Liebherr (CUIC, 2), lot 10, Polhemus (NMNH, 4), Heleleikeoha Str., ridge S fence camp, pyrethrin fog *Metrosideros*/moss, 1795 m el., 12-v-1998 lot 11, Polhemus (NMNH, 10), Horseshoe Bog nr. Haleakala N.P., sift *Myrsine*/*Dubautia*, 1830 m el., 12-v-1998 lot 06, Liebherr (CUIC, 1), Kaumakani Peak, pyrethrin fog vegetation, 1127 m el., 07-vi-1999 lot 05, Polhemus (NMNH, 1), 08-vi-1999 lot 05, Polhemus (NMNH, 3), 1135 m el., 07-vi-1999 lot 02, Polhemus (NMNH, 1), 1160 m el., 08-vi-1999 lot 02, Polhemus (NMNH, 1), lot 03, Polhemus (NMNH, 3), 1165 m el., 08-vi-1999 lot 04, Polhemus (NMNH, 1); Koolau For. Res., Hanawi N.A.R., 1680–2065 m el., v-1997, Bruch (HALE, 3), 21-ix-1997, Takumi (HALE, 1, 1830 m el., 23-viii-1997 lot 01, Gruner (UHIM, 1), Poouli Cabin, nr., 1630 m el., vi-1997, Gruner (UHIM, 4), pig fence helipad [= future Poouli Cabin site], sift *Metrosideros* humus, 1585 m el., 21-v-1993 lot 02, Liebherr/Medeiros (CUIC, 1), Kuhiwa Vy. E rim, *Pipturus*, in gulch, 1030 m el., 09-vi-1999 lot 10, Ewing (CUIC, 1), Kula Pipeline Rd. 1270 m el., vii-1956, Namba (BPBM, 1), Waikamoi Flume, beat *Cibotium menziesii*, 1305 m el., 03-vi-1996 lot 02, Ewing (CUIC, 1), Waikamoi Gulch, 1524 m el., 13-viii-1991, Tauber/Tauber (CUIC, 3); Makawao For. Res., Maile Rd., scrape *Metrosideros* bark, 1310 m el., 26-v-1997 lot 08, Liebherr (CUIC, 1); Waikamoi N.C.P., 1540 m el., 16-viii-1991, Gambino (CUIC, 1), sample 08, *Acacia koa*, bark trap, 20-v-2011, Peck (BPBM, 1), Honomanu drainage, boardwalk, beat Ohelo + *Cibotium montana*, 1890 m el., 07-vi-1996 lot 01, Ewing (CUIC, 1), Honomanu drainage, transect 3, scraping *Acacia koa* bark, 1820–1850 m el., 07-v-1991 lot 03, Liebherr (CUIC, 2), scraping *Metrosideros* humus/moss, 1700 m el., 08-v-1991 lot 03, Liebherr (CUIC, 3), sift litter, 1700 m el., 10-iv-1991 lot 01, Liebherr (CUIC, 2), sift moss and litter, 1680–1710 m el., 08-v-1991 lot 06, Kavanaugh (CAS, 3), 1680 m el., 08-v-1991 lot 07, Kavanaugh (CAS, 1), 1770–1830 m el., 08-v-1991 lot 08, Kavanaugh (CAS, 1), Hosmer Grove, below, beat vegetation at night, 1875 m el., 02-v-1998 lot 04, Liebherr (CUIC, 1).

### (093) *Mecyclothorax kipwilli* sp. n.

256 paratypes. HI: Maui: Haleakala, vii-1919, Forbes (BPBM, 1); Haleakala N.P., NW upper slope, beating, 1830–1980 m el., 18-viii-1937, Zimmerman (BPBM, 1), Northeast Rift, Greensword Bog, 1859 m el., 22-25-vi-1981, Gagné (BPBM, 1), Midcamp Bog Cabin, beat *Metrosideros*/styphelia at night, 1665 m el., 18-v-1993 lot 05, Liebherr/Medeiros (CUIC, 1), sift *Metrosideros* humus, 1665 m el., 18-v-1993 lot 01, Liebherr/Medeiros (CUIC, 4), lot 02, Liebherr/Medeiros (CUIC, 9), lot 03, Liebherr/Medeiros (CUIC, 13), lot 04, Liebherr/Medeiros (CUIC, 9), New Greensword Bog, sift *Metrosideros*/styphelia, 1850 m el., 17-v-1993 lot 02, Liebherr/Medeiros (CUIC, 10), lot 03, Liebherr/Medeiros (CUIC, 1); Hana For. Res., Smith Camp [=Big Bog], *Metrosideros* at night, 1640 m el., 14-viii-2008, Kaholoa‘a (HALE, 1), Heleleikeoha Str. State Fence Camp, pyrethrin fog *Metrosideros*/*Astelia*, 1615 m el., 11-v-1998 lot 02, Liebherr (CUIC, 1), pyrethrin fog *Metrosideros*/moss, 1615 m el., 11-v-1998 lot 01, Liebherr (CUIC, 3), lot 06, Polhemus (NMNH, 10), 12-v-1998 lot 01, Liebherr (CUIC, 4), lot 03, Liebherr (CUIC, 1), lot 04, Liebherr (CUIC, 1), lot 10, Polhemus (NMNH, 5), Heleleikeoha Str., ridge S fence camp, pyrethrin fog *Metrosideros*/moss, 1795 m el., 1795 m el., 12-v-1998 lot 11, Polhemus (NMNH, 15), Horseshoe Bog nr. Haleakala N.P., pyrethrin fog *Metrosideros*/moss, 1830 m el., 12-v-1998 lot 05, Liebherr (CUIC, 24), sift *Myrsine*/*Dubautia*, 1830 m el., 12-v-1998 lot 06, Liebherr (CUIC, 4); Koolau For. Res., Hanawi N.A.R., sample 05, *Metrosideros* bark trap, 26-v-2011 lot 02, Peck (BPBM, 1), sample 09, *Metrosideros* branch clip, 15-iv-2011 lot 01, Peck (BPBM, 1), sample 13, *Metrosideros* bark trap, 28-v-2011 lot 01, Peck (BPBM, 1), sample 14, *Metrosideros* bark trap, 28-v-2011 lot 02, Peck (BPBM, 1), Frisbee Meadow Camp, woods below, beat *Metrosideros* at night, 2072–2099 m el., 19-v-1993 lot 06, Liebherr/Medeiros (CUIC, 1), sift *Dubautia*/tree litter, 2072–2099 m el., 19-v-1993 lot 01, Liebherr/Medeiros (CUIC, 4), sift *Metrosideros* humus, 2072–2099 m el., 19-v-1993 lot 02, Liebherr/Medeiros (CUIC, 5), lot 03, Liebherr/Medeiros (CUIC, 1), Poouli Cabin, nr., 1630 m el., vi-1997, Gruner (UHIM, 1), Kuhiwa Str. E Poouli Cabin, beat *Melicope*, 1590 m el., 06-v-1998 lot 04, Ewing (CUIC, 1), Kuhiwa Vy., Poouli Cabin, beat vegetation, 1590 m el., 05-v-1998 lot 04, Ewing (CUIC, 1), pyrethrin fog *Metrosideros*/*Cibotium*, 1590 m el., 05-v-1998 lot 02, Liebherr (CUIC, 9), 06-v-1998 lot 02, Liebherr (CUIC, 1), pyrethrin fog *Metrosideros*/moss, 1590 m el., 05-v-1998 lot 01, Liebherr (CUIC, 29), lot 03, Polhemus (NMNH, 9), 06-v-1998 lot 01, Liebherr (CUIC, 1), pyrethrin fog *Metrosideros* roots/trunk, 1590 m el., 06-v-1998 lot 07, Polhemus (NMNH, 7), pig fence helipad [=subsequent Poouli Cabin site], beat vegetation at night, 1585 m el., 20-v-1993 lot 02, Liebherr/Medeiros (CUIC, 1), sift *Metrosideros* humus, 1585 m el., 21-v-1993 lot 01, Liebherr/Medeiros (CUIC, 2), lot 04, Liebherr/Medeiros (CUIC, 3), sweeping *Cyanea*, 1524 m el., 09-v-1973, Gagné (BPBM, 1), Koolau Gap, Halehaku, beat *Cheirodendron* at night, 1325 m el., 13-v-1998 lot 10, Ewing (CUIC, 1), beat ferns at night, 1325 m el., 13-v-1998 lot 09, Liebherr (CUIC, 1), beat *Metrosideros*/moss, 1325 m el., 13-v-1998 lot 11, Polhemus (NMNH, 5), pyrethrin fog *Cibotium*/log, 1325 m el., 13-v-1998 lot 03, Liebherr (CUIC, 1), pyrethrin fog *Metrosideros*/moss, 1325 m el., 13-v-1998 lot 02, Liebherr (CUIC, 1), Kula Pipeline Rd., pyrethrin fog *Metrosideros*/moss, 1170 m el., 15-v-1998 lot 06, Polhemus (NMNH, 1), Waikamoi Gulch, Waikamoi Flume, beat/scrape *Metrosideros*, 1310 m el., 26-v-1997 lot 06, Liebherr (CUIC, 5), sift *Metrosideros*/moss, 1310 m el., 09-v-1991 lot 02, Liebherr (CUIC, 1), Waikamoi Flume W end, *Clermontia* bark, 1310 m el., 06-viii-1969, Montgomery (BPBM, 1); Waikamoi N.C.P., pyrethrin fog *Metrosideros*/moss, 1750 m el., 12-iii-2002 lot 01, Takumi (HALE, 1), sample 02, *Acacia koa* bark trap, 09-iv-2011 lot 02, Peck (BPBM, 1), sample 06, *Metrosideros* bark trap, 21-v-2011 lot 04, Peck (BPBM, 1), sample 07, *Acacia koa* bark trap, 20-v-2011 lot 02, Peck (BPBM, 1), sample 08, *Acacia koa* bark trap, 20-v-2011 lot 03, Peck (BPBM, 1), sample 10, *Metrosideros* bark trap, 20-v-2011 20110520.08 Peck (BPBM, 1), Honomanu drainage, transect 3, scraping *Acacia koa* bark, 1820–1850 m el., 07-v-1991 lot 03, Liebherr (CUIC, 3), *Metrosideros*/moss, 1700 m el., 13-v-1993 lot 01, Liebherr (CUIC, 1), *Metrosideros* moss ex trunk, 1830–1860 m el., 07-v-1991 lot 09, Kavanaugh (CAS, 4), scrape *Metrosideros* bark, 1700 m el., 08-v-1991 lot 01, Liebherr (CUIC, 1), sift litter, 1700 m el., 10-iv-1991 lot 01, Liebherr (CUIC, 1), sift moss and litter, 1680–1710 m el., 08-v-1991 lot 06, Kavanaugh (CAS, 5), scraping *Metrosideros* bark/moss, 1820–1850 m el., 07-v-1991 lot 02, Liebherr (CUIC, 2), scraping *Metrosideros* humus/moss, 1700 m el., 08-v-1991 lot 03, Liebherr (CUIC, 12), transect 20, scraping *Acacia koa* bark, 1798–1860 m el., 13-v-1993 lot 03, Liebherr (CUIC, 2), scrape *Metrosideros* bark, 1860 m el., 13-v-1993 lot 02, Liebherr (CUIC, 1), Ukulele Pipeline, above, beat *Cheirodendron* at night, 1534–1660 m el., 14-v-1998 lot 04, Ewing (CUIC, 1).

### (094) *Mecyclothorax kipahulu* sp. n.

138 paratypes. HI: Maui: Haleakala N.P., Kipahulu Vy., beat vegetation at night, 1500 m el., 09-v-1991 lot 04, Jessel/Medeiros (CUIC, 2), 1900 m el., 08-v-1991 lot 05, Jessel/Medeiros (CUIC, 17), sifting litter, 1500 m el., 09-v-1991 lot 03, Jessel/Medeiros (CUIC, 3), 1800 m el., 08-v-1991 04, Jessel/Medeiros (CUIC, 4), Mauka Ridge, pyrethrin fog *Metrosideros*/moss, 2045 m el., 21-v-1998 lot 01, Polhemus (NMNH, 23), Palikea Camp, 1300 m el., 20-iv-2000, Takumi (HALE, 2), West Camp, pyrethrin fog *Metrosideros*/moss, 1960 m el., 19-v-1998 lot 01, Polhemus (NMNH, 19), 20-v-1998 lot 01, Polhemus (NMNH, 51), on tree bark at night, 1859 m el., 17-vii-1983, Howarth (BPBM, 7), tree trunks at night, 1859 m el., 12-vii-1983, Howarth (BPBM, 1), pit crater below West Camp, pyrethrin fog *Metrosideros*/moss, 1845 m el., 19-v-1998 lot 02, Polhemus (NMNH, 9).

### (095) *Mecyclothorax kaumakani* sp. n.

42 paratypes. HI: Maui: Haleakala N.P., Kekuewa Hill 0.7 km N Puu Ahulili, sift humus/moss, 1600 m el., 16-v-1993 lot 01, Liebherr/Medeiros (CUIC, 3), lot 02, Liebherr/Medeiros (CUIC, 3), lot 03, Liebherr/Medeiros (CUIC, 3), lot 04, Liebherr/Medeiros (CUIC, 1), Kipahulu west rim ESE Kuiki, beat vegetation at night, 1850 m el., 15-v-1993 lot 06, Liebherr/Medeiros (CUIC, 2), sift *Metrosideros* humus, 1850 m el., 15-v-1993 lot 03, Liebherr/Medeiros (CUIC, 1), lot 04, Liebherr/Medeiros (CUIC, 4), lot 05, Liebherr/Medeiros (CUIC, 4); Hana For. Res., Kaumakani Peak, pyrethrin fog vegetation, 1127 m el., 08-vi-1999 lot 05, Polhemus (NMNH, 3), lot 07, Polhemus (NMNH, 7), 1160 m el., 08-vi-1999 lot 03, Polhemus (NMNH, 10), 1165 m el., 08-vi-1999 lot 04, Polhemus (NMNH, 1).

### (096) *Mecyclothorax kuiki* sp. n.

84 paratypes. HI: Maui: Haleakala N.P., Kaapahu, Ohia camp, 1525 m el., 05-iv-2004, Kaholoa‘a (BPBM, 1), below Kuiki, sift *Metrosideros* litter, 2145 m el., 16-v-2001 lot 02, Liebherr (CUIC, 1), pyrethrin fog *Metrosideros*/moss, 2145 m el., 16-v-2001 lot 01, Liebherr (CUIC, 16), Kipahulu west rim below Kuiki, beat *Metrosideros* at night, 2105 m el., 14-v-1993 lot 07, Liebherr/Medeiros (CUIC, 15), sift *Metrosideros* humus, 2090 m el., 14-v-1993 lot 02, Liebherr/Medeiros (CUIC, 8), lot 04, Liebherr/Medeiros (CUIC, 16), sift *Metrosideros*/moss, 2090 m el., 14-v-1993 lot 01, Liebherr/Medeiros (CUIC, 6), 2105 m el., 15-v-1993 lot 01, Liebherr/Medeiros (CUIC, 4), sift moss ex treebase, 2090 m el., 14-v-1993 lot 03, Liebherr/Medeiros (CUIC, 2), Northeast Rift, New Greensword Bog, beat vegetation at night, 1850 m el., 17-v-1993 lot 06, Liebherr/Medeiros (CUIC, 1), sift *Metrosideros* humus, 1850 m el., 17-v-1993 lot 04, Liebherr/Medeiros (CUIC, 7), lot 05, Liebherr/Medeiros (CUIC, 3), sift *Metrosideros*/styphelia, 1850 m el., 17-v-1993 lot 03, Liebherr/Medeiros (CUIC, 4).

### (110) *Mecyclothorax bilobatus* sp. n.

103 paratypes. HI Maui: Hana For. Res., Heleleikeoha Str. State Fence Camp, pyrethrin fog *Metrosideros*/*Astelia*, 1615 m el., 11-v-1998 lot 01, Liebherr (CUIC, 11), lot 02, Liebherr (CUIC, 4), pyrethrin fog *Metrosideros*/moss, 1615 m el., 11-v-1998 lot 06, Polhemus (NMNH, 33), 12-v-1998 lot 01, Liebherr (CUIC, 6), lot 03, Liebherr (CUIC, 3), lot 04, Liebherr (CUIC, 3), lot 10, Polhemus (NMNH, 11), Heleleikeoha Str., ridge S fence camp, pyrethrin fog *Metrosideros*/moss, 1795 m el., 12-v-1998 lot 11, Polhemus (NMNH, 23), Horseshoe Bog nr. Haleakala N.P., pyrethrin fog *Metrosideros*/moss, 1830 m el., 12-v-1998 lot 05, Liebherr (CUIC, 2); Koolau For. Res., Hanawi N.A.R., Kuhiwa Vy., Poouli Cabin, pyrethrin fog *Metrosideros*/moss, 1590 m el., 05-v-1998 lot 03, Polhemus (NMNH, 3), 06-v-1998, lot 06, Polhemus (NMNH, 2), pyrethrin fog *Metrosideros* roots/trunk, 1590 m el., 06-v-1998 lot 07, Polhemus (NMNH, 2).

### (111) *Mecyclothorax palustroides* sp. n.

61 paratypes. HI: Maui: Haleakala N.P., Kipahulu Vy., Mauka Ridge, pyrethrin fog *Metrosideros*/moss, 2045 m el., 21-v-1998 lot 01, Polhemus (NMNH, 4), West Camp, 1859 m el., 17-vii-1983, lot 03, Howarth/Gagné (BPBM, 4), pyrethrin fog *Metrosideros*/moss, 1960 m el., 19-v-1998 lot 01, Polhemus (NMNH, 5), 20-v-1998 lot 01, Polhemus (NMNH, 17), tree trunks at night, 1859 m el., 12-vii-1983 lot 02, Howarth (BPBM, 3), pit crater below West Camp, pyrethrin fog *Metrosideros*/moss, 1845 m el., 19-v-1998 lot 02, Polhemus (NMNH, 3); Koolau For. Res., Kula Pipeline Rd., beat vegetation at night, 1265 m el., 26-v-1997 lot 09, Liebherr (CUIC, 2); Waikamoi N.C.P., Honomanu drainage, transect 3, scrape bark *Acacia koa*, 1820–1850 m el., 07-v-1991 lot 03, Liebherr (CUIC, 2), moss on *Metrosideros* trunk, 1830–1860 m el., 07-v-1991 lot 09, Kavanaugh (CAS, 8), sift litter, 1700 m el., 10-iv-1991 lot 01, Liebherr (CUIC, 3), sift moss/litter, 1680–1710 m el., 08-v-1991 lot 06, Kavanaugh (CAS, 3), scrape *Metrosideros* humus/moss, 1700 m el., 08-v-1991 lot 03, Liebherr (CUIC, 3), Ke‘anae Gap Camp 6, 1524 m el., 09-i-1998, Haines (CUIC, 1), Ukulele Pipeline, pyrethrin fog mossy *Metrosideros* log, 1510 m el., 16-v-2003 lot 05, Liebherr (CUIC, 2); Makawao F.R., Maile Tr., beat *Cibotium menziesii*, 1423 m el., 02-v-1998 lot 08, Ewing (CUIC, 1).

### (112) *Mecyclothorax filipoides* sp. n.

54 paratypes. HI: Maui: Haleakala N.P., NW upper slope, beating, 1830–1980 m el., 18-viii-1937, Zimmerman (BPBM, 1). Koolau For. Res., Makawao-Maile Tr., ecotone forest, yellow pan trap, 1293–1426 m el., vi–viii-2006, Leblanc (UHIM, 2), 1426–1573 m el., 06-08-2006, Leblanc (UHIM, 2). Makawao For. Res., Maile Rd., scraping bark *Acacia koa*, 1310 m el., 26-v-1997 lot 07, Liebherr (CUIC, 1), scraping bark *Metrosideros*, 1310 m el., 26-v-1997 lot 08, Liebherr (CUIC, 2), ex *Clermontia grandiflora* flower, 26-v-1997 lot 10 (CUIC, 1); nr. Puu Luau, beating shrubberies, 1665 m el., 28-iv-1945 lot 02, Zimmerman (BPBM, 1); Waikamoi N.C.P., pyrethrin fog *Metrosideros*/moss, 1750 m el., 12-iii-2002, Takumi (HALE, 7), bark trap sample 02, *Acacia koa*, 09-iv-2011 lot 02, Peck (BPBM, 1), bark trap sample 06, *Metrosideros*, 21-v-2011 lot 04, Peck (BPBM, 1), Honomanu drainage, transect 20, scraping bark *Acacia koa*, 1798–1860 m el., 13-v-1993 lot 03, Liebherr (CUIC, 2), transect 3, scraping bark *Acacia koa*, 1820–1850 m el., 07-v-1991, lot 03, Liebherr (CUIC, 2), scraping bark/moss *Metrosideros*, 1820–1850 m el., 07-v-1991 lot 02, Liebherr (CUIC, 1), *Metrosideros* mossy trunk, 1830–1860 m el., 07-v-1991 lot 09, Kavanaugh (CAS, 2), Maile Rd., pyrethrin fog *Acacia koa* trunk, 1470 m el., 02-v-1998 lot 02, Liebherr (CUIC, 7), above Ukulele Pipeline, beat *Cheirodendron* at night, 1534–1660 m el., 14-v-1998 lot 04, Ewing (CUIC, 1), beat vegetation at night, 1534–1660 m el., 14-v-1998 lot 03, Liebherr (CUIC, 16), Ukulele Pipeline, rotten *Cheirodendron*, 1465–1495 m el., 07-v-1998 lot 06, Ewing (CUIC, 1), pyrethrin fog *Metrosideros*/moss, 1534 m el., 07-v-1998 lot 02, Liebherr (CUIC, 2), sift ground litter, 1495 m el., 18-v-2003 lot 04, Liebherr (CUIC, 1).

### (113) *Mecyclothorax nanunctus* sp. n.

84 paratypes. HI: Maui: Haleakala N.P., Kipahulu Vy., sift litter, 1500 m el., 09-v-1991 lot 03, Jessel/Medeiros (CUIC, 1), 1800 m el., 08-v-1991 lot 04, Jessel/Medeiros (CUIC, 2), Central Pali Tr., beat vegetation at night, 1200 m el., 29-iv-1991 lot 04, Liebherr/Medeiros (CUIC, 2), Kipahulu west rim ESE Kuiki, sift *Metrosideros* humus, 1850 m el., 15-v-1993 lot 04, Liebherr/Medeiros (CUIC, 1); Hana For. Res., Heleleikeoha Str. State Fence Camp, pyrethrin fog *Metrosideros*/moss, 1615 m el., 11-v-1998 lot 01, Liebherr (CUIC, 1), 12-v-1998 lot 04, Liebherr (CUIC, 1), S fence camp, pyrethrin fog *Metrosideros*/moss, 1795 m el., 12-v-1998 lot 11, Polhemus (NMNH, 2); Koolau For. Res., 1210 m el., vii-1956 lot 01, Namba (BPBM, 1), 1220 m el., 03-viii-1973 lot 02, Whittle (UHIM, 1), Flume Rd., scraping bark *Acacia koa*, 1310 m el., 15-v-2003, lot 01, Liebherr (CUIC, 1), Hanawi N.A.R., Kuhiwa Vy., Poouli Cabin, pyrethrin fog *Metrosideros*/*Cibotium*, 1590 m el., 05-v-1998 lot 02, Liebherr (CUIC, 4), 06-v-1998 lot 02, Liebherr (CUIC, 1), pyrethrin fog *Metrosideros*/moss, 1590 m el., 05-v-1998 lot 03, Polhemus (NMNH, 33), 06-v-1998 lot 01, Liebherr (CUIC, 8), lot 06, Polhemus (NMNH, 11), pyrethrin fog *Metrosideros* roots/trunk, 1590 m el., 06-v-1998 lot 07, Polhemus (NMNH, 5), Kula Pipeline Rd., Ohia lehua, 975–1220 m el., 30-v-1930, Swezey (BPBM, 1), pyrethrin fog *Metrosideros*, 1265 m el., 18-v-2003 lot 08, Polhemus (NMNH, 2); Waikamoi N.C.P., Honomanu drainage, transect 3, sift litter, 1700 m el., 10-iv-1991 lot 01, Liebherr (CUIC, 1), Ukulele Pipeline, *Astelia*, 1510 m el., 16-v-2003 lot 07, Haines (CUIC, 1), pyrethrin fog mossy *Metrosideros* log, 1510 m el., 16-v-2003 lot 05, Liebherr (CUIC, 3); Makawao F.R., Puu Luau, edge of forest, *Cyanea* sp., 1765 m el., 17-vii-1919 lot 03, Timberlake (UCRC, 1).

### (116) *Mecyclothorax pau* sp. n.

278 paratypes. HI: Maui: Haleakala N.P., Kekuewa Hill, 0.7 km N Puu Ahulili, sift humus/moss, 1600 m el., 16-v-1993 lot 01, Liebherr/Medeiros (CUIC, 10), lot 02, Liebherr/Medeiros (CUIC, 4), lot 03, Liebherr/Medeiros (CUIC, 2), lot 04, Liebherr/Medeiros (CUIC, 1), Kipahulu Vy., sift litter, 1500 m el., 09-v-1991 lot 03, Jessel/Medeiros (CUIC, 4), 1800 m el., 08-v-1991 lot 04, Jessel/Medeiros (CUIC, 6), beat vegetation at night, 1900 m el., 08-v-1991 lot 05, Jessel/Medeiros (CUIC, 2), Camp 3 to Rim, 1980-2260 m el., 21-25-viii-1967, Wilson (BPBM, 1), Central Pali Tr., sift leaf/moss litter, 915 m el., 30-iv-1991 lot 03, Liebherr/Medeiros (CUIC, 2), 1200 m el., 29-iv-1991 lot 03, Liebherr/Medeiros (CUIC, 13), under boards/logs/tarps, 915 m el., 30-iv-1991 lot 02, Liebherr/Medeiros (CUIC, 9), Mauka Ridge, pyrethrin fog *Metrosideros*/moss, 2045 m el., 21-v-1998 lot 01, Polhemus (NMNH, 4), West Camp, pyrethrin fog *Metrosideros*/moss, 1960 m el., 19-v-1998 lot 01, Polhemus (NMNH, 5), pit crater below West Camp, pyrethrin fog *Metrosideros*/moss, 1845 m el., 19-v-1998 lot 02, Polhemus (NMNH, 7), Kipahulu west rim ESE Kuiki, sift *Metrosideros* humus, 1850 m el., 15-v-1993 lot 02, Liebherr/Medeiros (CUIC, 2), lot 03, Liebherr/Medeiros (CUIC, 2), lot 04, Liebherr/Medeiros (CUIC, 2), lot 05, Liebherr/Medeiros (CUIC, 1), Northeast Rift, Big Bog, 1670 m el., 17-v-1991 lot 01, Mingard/Medeiros/White/Jessel, (CUIC, 3), Midcamp Bog Cabin, sift *Metrosideros* humus, 1665 m el., 18-v-1993 lot 02, Liebherr/Medeiros (CUIC, 2), lot 03, Liebherr/Medeiros (CUIC, 2), New Greensword Bog, sift *Metrosideros* humus, 1850 m el., 17-v-1993 lot 04, Liebherr/Medeiros (CUIC, 7) lot 05, Liebherr/Medeiros (CUIC, 1), sift *Metrosideros*/ styphelia, 1850 m el., 17-v-1993 lot 02, Liebherr/Medeiros (CUIC, 2); Hana For. Res., Heleleikeoha Str. State Fence Camp, pyrethrin fog *Metrosideros*/moss, 1615 m el., 12-v-1998 lot 04, Liebherr (CUIC, 1), lot 10, Polhemus (NMNH, 1), 11-v-1998 lot 01, Liebherr (CUIC, 1), ridge S fence camp, pyrethrin fog, *Metrosideros*/moss, 1795 m el., 12-v-1998 lot 11, Polhemus (NMNH, 1), Horseshoe Bog nr. Haleakala N.P., pyrethrin fog *Metrosideros*/moss, 1830 m el., 12-v-1998 lot 05, Liebherr (CUIC, 1); Hanawi N.A.R., Kuhiwa Vy., Poouli Cabin, pyrethrin fog *Metrosideros*/*Cibotium*, 1590 m el., 05-v-1998 lot 02, Liebherr (CUIC, 33), 06-v-1998, lot 02, Liebherr (CUIC, 3), pyrethrin fog *Metrosideros*/moss, 1590 m el., 05-v-1998 lot 01, Liebherr (CUIC, 11), lot 03, Polhemus (NMNH, 9), 06-v-1998 lot 01, Liebherr (CUIC, 9), lot 06, Polhemus (NMNH, 2), pyrethrin fog *Metrosideros* roots/trunk, 1590 m el., 06-v-1998 lot 07, Polhemus (NMNH, 6), pig fence helipad [= Poouli Cabin site], sift *Metrosideros* humus, 1585 m el., 21-v-1993 lot 01, Liebherr/Medeiros (CUIC, 1), lot 02, Liebherr/Medeiros (CUIC, 1); Koolau For. Res., Koolau Gap, Halehaku, beat ferns at night, 1325 m el., 13-v-1998 lot 09, Liebherr (CUIC, 1), beat *Metrosideros*/moss, 1325 m el., 13-v-1998 lot 11, Polhemus (NMNH, 34), pyrethrin fog *Cibotium*, 1325 m el., 13-v-1998 lot 06, Liebherr (CUIC, 14), pyrethrin fog *Cibotium*/log, 1325 m el., 13-v-1998 lot 03, Liebherr (CUIC, 1), pyrethrin fog *Metrosideros*, 1325 m el., 13-v-1998 lot 04, Liebherr (CUIC, 3), lot 05, Liebherr (CUIC, 10), pyrethrin fog *Metrosideros*/moss, 1325 m el., 13-v-1998 lot 01, Liebherr (CUIC, 23), lot 02, Liebherr (CUIC, 18).
